# Regioselective radical α-borylation of α,β-unsaturated carbonyl compounds for direct synthesis of α-borylcarbonyl molecules

**DOI:** 10.1038/s41467-019-09825-3

**Published:** 2019-04-29

**Authors:** Shi-Chao Ren, Feng-Lian Zhang, Ai-Qing Xu, Yinuo Yang, Min Zheng, Xiaoguo Zhou, Yao Fu, Yi-Feng Wang

**Affiliations:** 1 Hefei National Laboratory for Physical Sciences at the Microscale, Center for Excellence in Molecular Synthesis of CAS, Department of Chemistry, University of Science and Technology of China, 96 Jinzhai Road, Hefei, Anhui 230026 China; 2 Department of Chemical Physics, University of Science and Technology of China, 96 Jinzhai Road, Hefei, Anhui 230026 China; 3 State Key Laboratory of Elemento-Organic Chemistry, Nankai University, Tianjin, 300071 China

**Keywords:** Reactive precursors, Reaction mechanisms, Synthetic chemistry methodology

## Abstract

Organoboron compounds are highly valuable in synthetic chemistry. In particular, α-borylcarbonyl compounds have shown versatile synthetic applications, owing to fruitful chemistries of both the boryl and carbonyl moieties. However, the synthesis of these molecules still remains tedious and time-consuming. Here we report a straightforward and practical route to synthesize *α*-borylcarbonyl molecules based on a regioselective radical *α*-borylation of *α*,*β*-unsaturated carbonyl compounds. The reaction features unusual *α*-regioselectivity and high functional-group compatibility. Further synthetic applications of new *α*-borylated products were also demonstrated. DFT and kinetic studies implicated that the *α*-regioselectivity of *β*-aryl-*α*,*β*-unsaturated carbonyl compounds was determined by the thermodynamically more favorable radical *α*-addition step, whereas the formation of *α*-addition products from *β*-alkyl-*α*,*β*-unsaturated carbonyl compounds was driven by an energetically favored hydrogen atom transfer step. Given that *α*,*β*-unsaturated carbonyl compounds can be easily obtained in abundance and variety, this method enjoys great advantages in diverse and economical synthesis of valuable *α*-borylcarbonyl molecules.

## Abstract (web summary)

α-Borylcarbonyl compounds are versatile synthons in organic synthesis, however their preparation is often tedious. Here, the authors report a regioselective radical α-borylation of α,β-unsaturated carbonyl compounds to afford α-borylcarbonyl molecules with high selectivity and functional group compatibility.

## Introduction

Organoboron compounds have been substantially applied in chemical synthesis, medicinal chemistry, and material sciences^1^. Borylative functionalization of alkenes is among the most widely used methods to access these products^2–6^. In this context, great endeavors have been devoted to exploit borylation reactions of *α*,*β*-unsaturated carbonyl compounds, owing to their easy accessibility and intrinsically high electrophilicity. Over the past decades, many reagents and conditions have been well established to synthesize *β*-borylcarbonyl compounds through nucleophilic *β*-borylation^7–10^. In sharp contrast, the direct *α*-borylation reaction to access isolable *α*-borylcarbonyl molecules remains a formidable challenge^11^, mainly because the products undergo facile 1,3-boron shift to give thermodynamically more stable O-boron enolates^12^. Thus, developing practical and broadly applicable new *α*-borylation methods that can make stable and structurally diverse *α*-borylcarbonyl compounds is highly desirable.

**Fig. 1:**
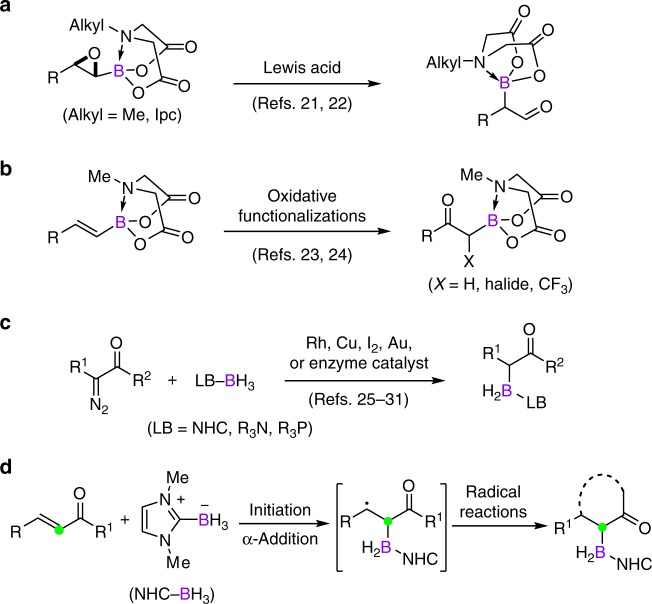
Synthesis of stable α-borylcarbonyl molecules. **a** Lewis acid-catalyzed 1,2-boryl migration of oxiranyl boronates. **b** Oxidative functionalization of borylated alkenes. **c** B−H Insertion reactions of Lewis base-boranes and α-diazocarbonyl compounds. **d** This work: radical α-borylation of *α*,*β*-unsaturated carbonyl compounds

α-Borylcarbonyl compounds with the boron center being quaternized by a Lewis base are air stable and easily handled^13–15^. In these molecules, both the boron and carbonyl moieties can be manipulated to install various functionalities of interest. This strategy has shown important applications in a variety of research fields and thus the synthesis of stable α-borylcarbonyl compounds has captured considerable attention in synthetic chemistry^13–20^. For example, He and Yudin^21^, and Li and Burke^22^ independently reported the synthesis of amphoteric α-boryl aldehydes from oxiranyl boronates by a Lewis acid-promoted 1,2-boryl migration (Fig. 1a). Wang and colleagues^23^, and Yudin and colleagues^24^ have developed oxidative functionalization reactions of alkenylboronates to make *α*-boryl ketones (Fig. 1b). In addition, Curran and colleagues^25,26^, Zhou and colleagues^27–29^, Xu and colleagues^30^, and Arnold and colleagues^31^ have described a number of catalytic B−H insertion reactions of Lewis base-boranes and *α*-diazocarbonyl compounds (Fig. 1c). Despite these advances, the preparation of these starting materials usually required multi-step synthesis, which limits the functionality and applicability. Various structurally diverse *α*,*β*-unsaturated carbonyl compounds are readily accessible. Arguably, the direct *α*-borylation of *α*,*β*-unsaturated carbonyl compounds to access value-added *α*-borylcarbonyl molecules is a more straightforward and economical strategy. However, this has rarely been documented. During the reviewing of this manuscript, Ingleson and colleagues^32^ reported an iodine-catalyzed ionic hydroboration of *α*,*β*-unsaturated esters. Herein, we report a conceptually distinct regioselective radical *α*-borylation protocol by taking advantages of the chemical reactivity of *N*-heterocyclic carbene (NHC)−boryl radical (Fig. 1d). Using radical cascade strategy, complex boron-substituted cyclic frameworks can be constructed as well.

## Results

### Reaction design

Recent years have witnessed the significant progress in NHC−boryl radical chemistry^33–40^, as the pioneering work of Curran and colleagues^41^. Our group has been interested in the exploration of new radical borylation methods to create C−B bonds from alkenes and alkynes^42,43^. Our previous studies revealed that the NHC−boryl radical addition to alkenes should be a reversible process and the reaction equilibrium is favored to the addition products when the resulted alkyl radicals are stabilized by an adjacent aryl or carbonyl group. In order to get more details of such reactivity, we intended to study the reaction with *β*-aryl-*α*,*β*-unsaturated carbonyl compounds. In this case, competitive *α*- and *β*-additions were envisioned to occur and the regioselectivity outcome would give us insightful information to rationalize the reaction profiles. This would further lead to the development of new radical borylation methods.

### Optimization of reaction conditions

We commenced our study by examining the radical hydroboration of ethyl cinnamate (**1a**) with 1,3-dimethylimidazol-2-ylidene borane (NHC−BH_3_, **2**) as the boryl radical precursor. Initially, *β*-addition was assumed to take place preferentially, owing to the strong inductive effect of the carbonyl group and the nucleophilic character of the NHC−boryl radical^44–48^. Unexpectedly, the reaction only gave *α*-borylated product **3a** in 49% yield in the presence of *tert*-dodecanethiol as a polarity reversal catalyst^45^ (Table 1, entry 1). The *β*-addition product was not detected at all. Such a specific *α*-regioselectivity has rarely been achieved in radical reaction of *α*,*β*-unsaturated carbonyl compounds, wherein *β*-regioselectivity usually predominated^49,50^. More importantly, this *α*-borylation reaction would offer a more straightforward and practical strategy to access valuable *α*-borylcarbonyl compounds from readily available starting materials. Thus, we decided to study the reaction further.

**Table 1:** Optimization of reaction conditions


Entry	LB-BH_3_	Initiator	RSH (*x* mol%)	3a Yield (%)^b^
1	**2a**	AIBN	*tert*-dodecanethiol (20)	49 (39)^d^
2	**2a**	AIBN	*tert*-dodecanethiol (50)	81^c^
3	**2a**	AIBN	PhSH (20)	77
4	**2a**	AIBN	4-MeOC_6_H_4_SH (20)	75
5	**2a**	AIBN	4-CO_2_MeC_6_H_4_SH (20)	73
6	**2a**	AIBN	MeO_2_CCH_2_SH (20)	80
7	**2b**	AIBN	PhSH (20)	89
8	**2c**	AIBN	PhSH (20)	70
9	**2d**	AIBN	PhSH (20)	67
10	**2e**	AIBN	PhSH (20)	0 (95)^e^
11	**2** **f**	AIBN	PhSH (20)	0 (98)^e^
12	**2a**	AIBN	--	0 (83)^d^
13^f^	**2a**	TBHN	*tert*-dodecanethiol (50)	67 (17)^d^
14^f^	**2a**	TBHN	PhSH (20)	60
15	**2a**	--	*tert*-dodecanethiol (50)	0 (98)^d^
16	**2a**	--	PhSH (20)	0 (88)^d^

^a^Reaction conditions: **2** (0.2–0.3 mmol), **1a** (1.2 equiv), initiator (20 mol%), RSH (*x* mol%), CH_3_CN (2 ml), 80 °C for 12 h

^b^NMR yield using tetrachloroethane as an internal standard

^c^Isolated yield

^d^Recovery yield of **2a** is shown in parentheses

^e^Recovery yield of **1a** is shown in parentheses

^f^The reaction was conducted at 50 °C

The effect of the thiol catalyst was first studied. Increasing the loading of *tert*-dodecanethiol to 50 mol% gave **3a** in an 81% isolated yield (entry 2). Using other thiol catalyst, such as benzenethiols (PhSH, 4-MeOC_6_H_4_SH, 4-CO_2_MeC_6_H_4_SH) and methyl thioglycolate (MeO_2_CCH_2_SH) as the catalyst also led to **3a** in comparable yields (entries 3–6). A range of Lewis base−BH_3_ complexes were tested as the boryl radical precursors. It was found that NHC−BH_3_ complexes, even including an electrophilic boryl radical precursor (**2d**)^51^ could participate in this radical hydroboration, producing the corresponding hydroboration products in good yields (entries 7–9). However, the use of pyridine−BH_3_ (**2e**) and Me_3_N−BH_3_ (**2f**) could not induce the desired boryl radical addition reaction and **1a** was fully recovered (entries 10 and 11). When the reaction was conducted without a thiol catalyst (entry 12), product **3a** was not detected, which suggested that the hydrogen atom transfer step might play an important role to control the reactivity and regioselectivity. Moderate yields of **3a** were obtained when di-*tert*-butyl hyponitrite was utilized as the radical initiator (entries 13 and 14). No reaction occurred in the absence of a radical initiator, indicating a radical mechanism is involved in this process (entries 15 and 16).

### Substrate scope of radical α-borylation reactions

The scope and generality of this *α*-borylation protocol was investigated (Table 2). A wide range of *α*,*β*-unsaturated carbonyl compounds were converted to *α*-boryl-*β*-aryl esters with aryl rings bearing a variety of functional groups (for **3a**–**3g**). The presence of additional simple alkene and alkyne motifs did not retard the desired borylation reaction (for **3h**–**3j**). Moreover, the intramolecular 6-*exo* cyclization of the resulted alkyl radicals with an alkyne or alkene tether did not occur for 1,6-enyne **1h** and 1,6-diene **1j**, probably due to a slower cyclization step than the hydrogen atom abstraction reaction. A range of *β*-heteroaryl rings, including indole (for **3k**), quinoline (for **3** **l**), and furan (for **3** **m**), could be installed. α-Borylation of cumarin proceeded to give the desired product **3n** in 72% yield. A mixture of diastereomers (for **3o**) was obtained for the reaction of **1o**. Increasing the steric congestion at the *α*-carbon (for **1p**) led to both *α*- and *β*-additions, affording **3p-*α*** and **3p-*β*** in 22% and 53% yields, respectively. The present method allowed for the construction of a series of *α*-borylated amides (for **3q**–**3t**). A gram scale synthesis of **3r** was also achieved in 79% yield. The reaction with amide **1t** bearing a chiral oxazolidinone unit gave product **3t** in a good yield but with low diastereoselectivity. When Lewis acids such as La(OTf)_3_, Zn(OTf)_2_, and MgBr_2_ were added to promote the diastereoselectivity^52,53^, the desired hydroboration product **3t** was not detected and hydrogenation of **1t** occurred instead (see Supplementary Table 1). Interestingly, the reaction of amide-bridged 1,6-enyne **1u** and 1,6-diene **1v**, both radical hydroboration and radical borylation/cyclization cascades proceeded, whereas the attempts to increase the yield of cyclized products **3u’** and **3v’** have been thus far proven unsuccessful. Remarkably, an intriguing 8-*endo* cyclization^54,55^ of intermediate **A** derived from *α*-borylation of *N*-(*o*-ethynylaryl)cinnamamides **1** proceeded smoothly to furnish boron-handled benzazocines (for **3w**–**3aa**). Notably, this protocol was also effective for the assembly of *α*-boryl ketones (for **3ab**–**3ad**) and even acid (for **3ae**). As for a limitation, the reaction of cinnamaldehyde resulted in a facile hydride reduction to give the corresponding alcohol and no hydroboration product was observed.

**Table 2:**
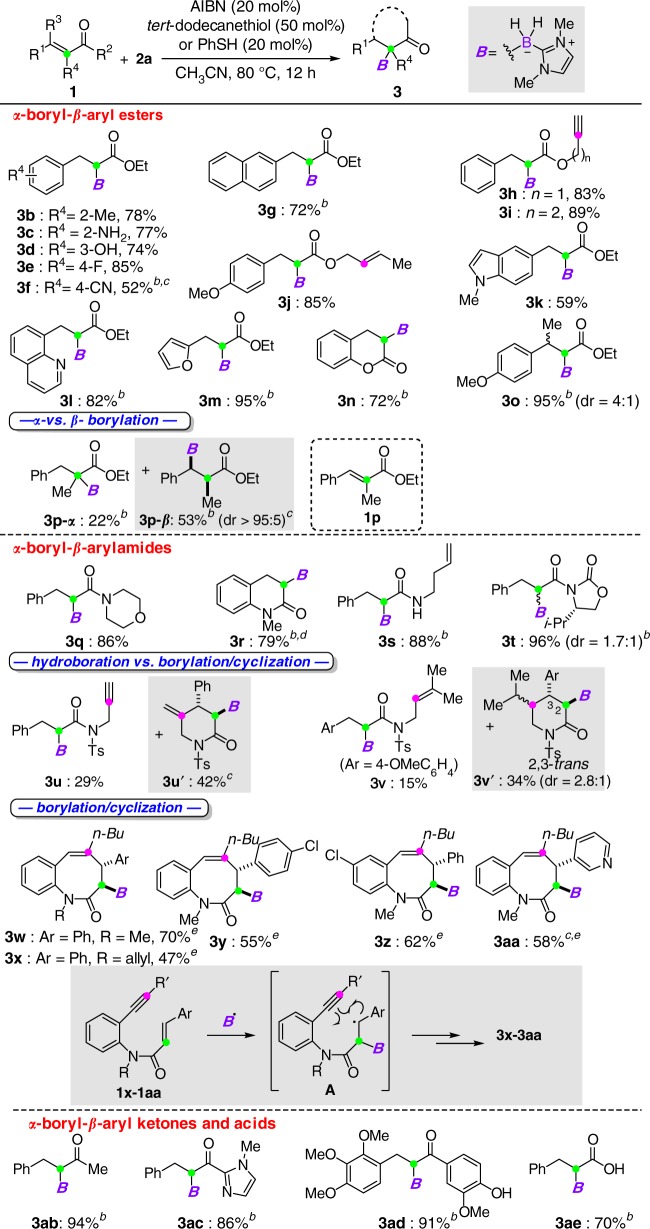
Scope of radical *α*-borylation of *β*-aryl-*α*,*β*-unsaturated carbonyl compounds

^a^Standard conditions: **2a** (0.2–0.3 mmol), **1** (1.2 equiv), AIBN (20 mol%), *tert*-dodecanethiol (50 mol%), CH_3_CN (2 ml), 80 °C for 12 h

^b^PhSH (20 mol%) was used

^c^The structures of **3f**, **3p-*β***, **3u’**, and **3aa** were secured by X-ray crystallographic analysis

^d^Isolated yield of a gram-scale synthesis

^e^Isooctyl thioglycolate (20 mol%) was used

**Table 3:**
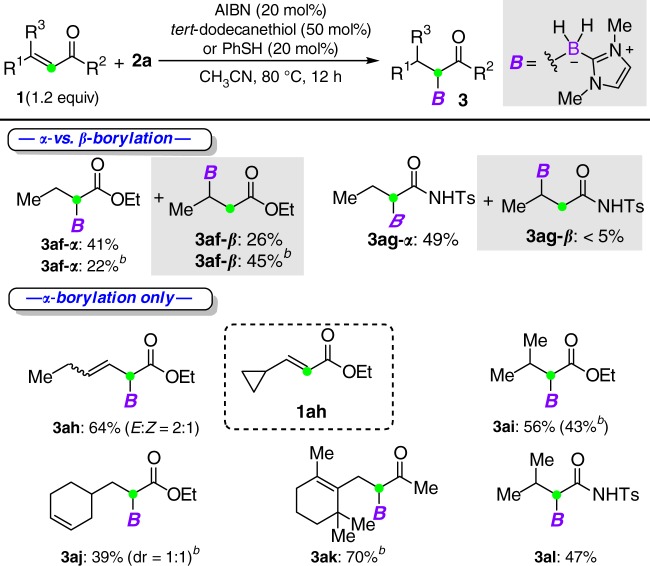
Scope of radical *α*-borylation of *β*-alkyl-*α*,*β*-unsaturated carbonyl compounds

^a^Standard conditions: **2a** (0.2–0.3 mmol), **1** (1.2 equiv), AIBN (20 mol%), *tert*-dodecanethiol (50 mol%), CH_3_CN (2 ml), 80 °C for 12 h

^b^PhSH (20 mol%) was used

The presence of a *β*-aryl group that can stabilize the resulting alkyl radical intermediates was envisioned to account for the above *α*-addition selectivity. Based on this assumption, we next turned our attention to study the reaction with *β*-alkyl-*α*,*β*-unsaturated carbonyl compounds (Table 3). The lack of aryl resonance stabilization would render the *β*-addition favorable. Surprisingly, when ethyl crotonate (**1af**) was subjected to the reaction conditions, *α*-borylation continued to be the major reaction pathway, giving **3af-*α*** in a 41% yield with 26% yield of the *β*-addition product **3af-*β***. When PhSH was used as the catalyst, **3af-*α*** and **3af-*β*** were formed in 22% and 45% yields, respectively. The reaction of crotonamide **1ag** resulted in superior *α*-selectivity, albeit with moderate yield. Notably, the introduction of a cyclopropane ring at the *β-*carbon (for **1ah**) led to only *α*-addition product **3ah**, which was probably driven by the facile ring-opening process. Furthermore, increasing the steric effect on the *β*-carbon gave solely *α*-borylated products (for **3ai**–**3al**). However, the reaction of ethyl acrylate under the optimized reaction conditions led to polymerization.

### Synthetic applications of α-borylcarbonyl compounds

**Fig. 2:**
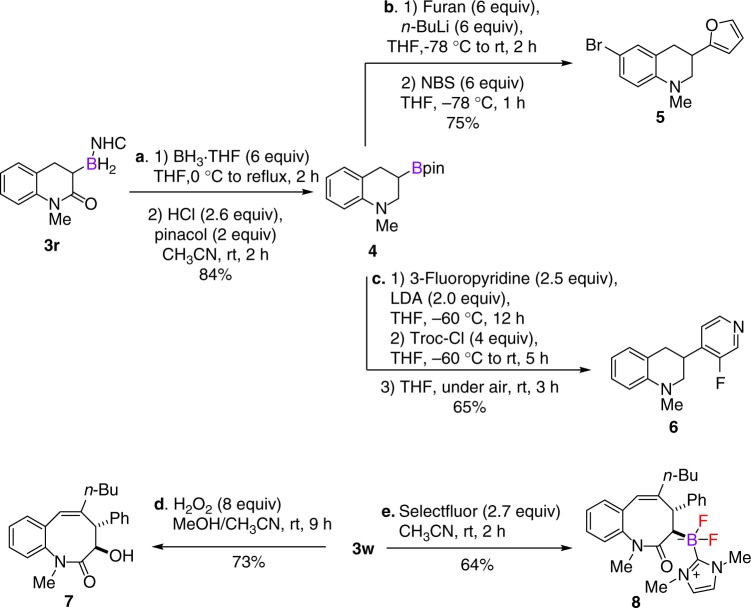
Diversification of *α*-borylcarbonyl compounds. **a** Synthesis of 3-boron-substituted tetrahydroquinoline **4** via a one-pot transformation from **3r**. **b** Synthesis of 3-furan-substituted tetrahydroquinoline **5**. **c**. Synthesis of 3-pyridine-substituted tetrahydroquinoline **6**. **d** Synthesis of *α*-hydroxy product **7** by the direct oxidation of the boron moiety. **e** Synthesis of NHC−difluoroboranes **8** from **3w**

The conversion of *α*-borylcarbonyl compounds to various functionalized borylated products have been well studied^13–15^. The present method allowed for the construction of a series of structurally new *α*-borylated molecules. The synthetic utility of some typical examples was demonstrated (Fig. 2). Reduction of the amide moiety of **3r** with BH_3_•THF afforded NHC−borane-substituted tetrahydroquinoline, which could be further converted to versatile pinacol boronic ester **4**. The subsequent coupling reactions with heteroaromatic compounds following Aggarwal’s protocols^56,57^ delivered furan- and pyridine-substituted tetrahydroquinolines **5** and **6**, respectively. In addition, the newly formed boron-handled benzazocines could be transformed to more useful building blocks. For example, oxidation of **3w** afforded *α*-hydroxy product **7** in 73% yield. Treatment of **3w** with Selectfluor furnished NHC−difluoroboranes **8**^58^, which represents a new class of stable *α*-borylcarbonyl molecules that may find potential synthetic applications.

## Discussion

**Fig. 3:**
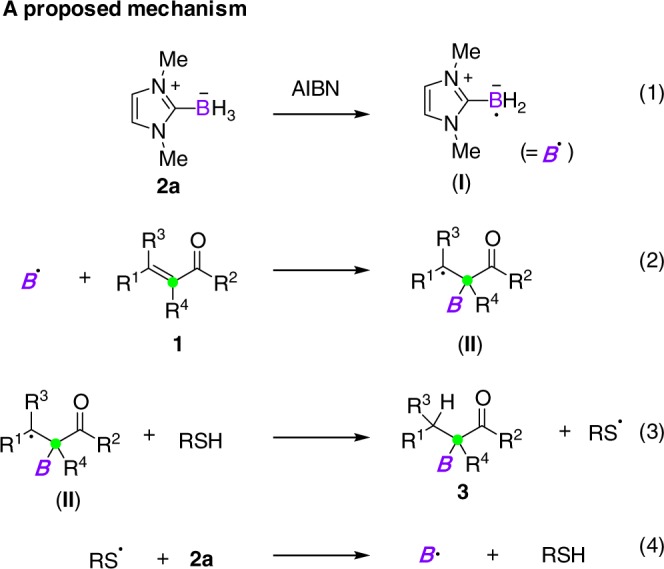
Mechanism. A proposed radical chain process for hydroboration of **1**

Based on the results obtained in this work and previous findings on NHC-boryl radicals^35^, a possible radical chain process was proposed for the hydroboration of *α*,*β*-unsaturated carbonyl compounds. As illustrated in Fig. 3, NHC-boryl radical (**I**) is first generated in the presence of AIBN as the radical initiator. After that, **I** undergoes addition to the *α*-carbon of **1**, giving alkyl radical intermediates **II**. The following hydrogen atom transfer from a thiol catalyst provides hydroboration products **3**. The resulting sulfur radical further abstracts a hydrogen atom from **2a** to regenerate **I** and RSH, thus propagating the radical chain process^45^.

**Fig. 4:**
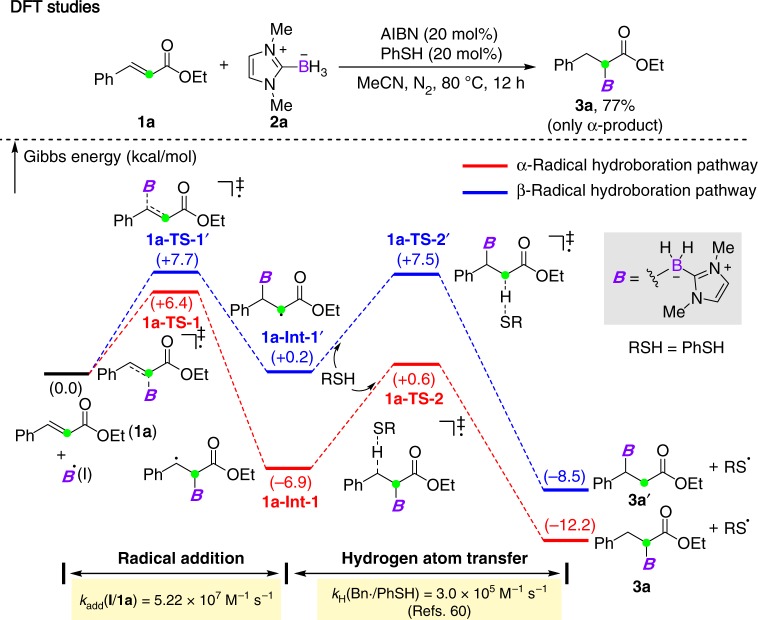
Theoretical calculations. DFT calculations of the radical hydroborylation of **1a**

**Fig. 5:**
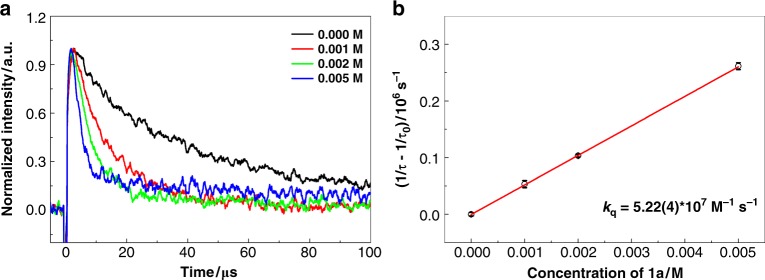
Kinetic studies. **a** Decay of **I** at 400 nm at 25 °C with increasing concentrations of **1a** from 0 M to 0.005 M. **b** Stern−Volmer plots generated from the fitted lifetime of **I** in the presence of **1a** in different concentrations at 25 °C

In this radical hydroboration process, an unusual *α*-regioselectivity was observed. To gain more insight into this selectivity, computational and kinetic studies were then performed. For the reaction of **1a**, as shown in Fig. 4, the Density Functional Theory (DFT) studies revealed that the energy barriers of *α*- and *β*-additions are comparable (+6.4 kcal mol^−1^ for *α*-addition and +7.7 kcal mol^−1^ for *β*-addition), while the resulting radical intermediates have significant energy difference (−6.9 kcal mol^−1^ for **1a-Int-1** and +0.2 kcal mol^−^^1^ for **1a-Int-1’**). This is probably attributed to more stabilization from the phenyl group than that from the ethoxycarbonyl motif^59^. The subsequent hydrogen atom transfer from the thiol catalyst to **1a-Int-1** and **1a-Int-1’** requires an almost same energy barrier (+7.5 kcal mol^−1^ for **1a-Int-1** and +7.3 kcal mol^−1^ for **1a-Int-1’**). Such a barrier renders the *β*-addition/elimination step reversible, thus accumulating the thermodynamically more favorable *α*-addition product **3a**. We next used laser flash photolysis (LFP) experiments^44^ to measure the rate constant for the addition reaction of NHC-boryl radical (**I**) to **1a**. Figure 5a shows the decay curves of absorption of **I** at 400 nm with increasing concentration of **1a**. The bimolecular reaction rate constant *k*_add_ = 5.22 × 10^7^ M^−1^ s^−1^ was determined by linear fitting the relationship between the reciprocal of the lifetime of **I** and the concentration of **1a** with the Stern–Volmer equation (Fig. 5b). It has been reported that the rate constant of hydrogen atom transfer from PhSH to a benzyl radical (*k*_H_) is 3.0 × 10^5^ M^−1^ s^−1 ^^60^, which is slower than the first radical addition step. Furthermore, the activation parameters ΔH^≠^ (−0.180 kcal mol^−1^) and ΔS^≠^ (−99.7 J mol^−1^) for the addition to **1a** were also determined, thereby giving the Gibbs free energy Δ*G*^≠^ = 6.92 kcal mol^−1^ (see Supplementary Fig. 11). This is comparable with the computational result.

**Fig. 6:**
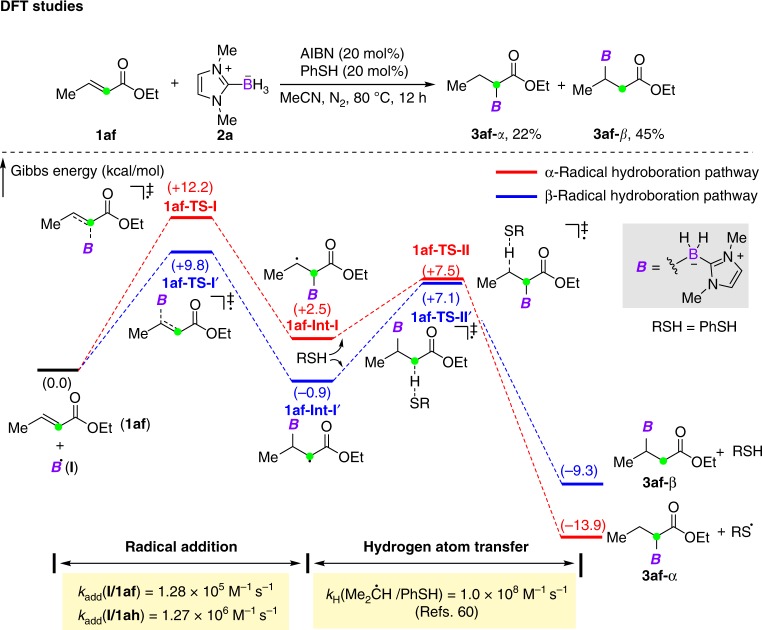
Theoretical calculations. DFT calculations of radical hydroborylation of **1af**

The reaction of **1af** gave a mixture of *α*- and *β*-addition products. It should be mentioned that the formation of *α*-product was quite unusual, because the *α*-addition intermediate has no *β*-aryl stabilization. To rationalize such reactivity, a computational study was performed. The free-energy profiles of the reaction between **1af** and NHC-boryl radical using PhSH as the catalyst are shown in Fig. 6. Indeed, in this reaction, the addition of NHC-boryl radical to the *α*-position of **1af** requires a higher activation energy (+12.2 kcal mol^−1^) than that to the *β*-carbon (+9.8 kcal mol^−1^). Moreover, the resulting *α*-addition intermediate **1af-Int-I** (+2.5 kcal mol^−1^) has higher energy than *β*-addition intermediate **1af-Int-I’** (−0.9 kcal mol^−1^). However, the subsequent hydrogen atom transfer step to **1af-Int-I** undergoes easily with a facile energy barrier of 5.0 kcal mol^−1^ (**1af-Int-I**→**1af-TS-II**) and an obvious energy decrease of 16.4 kcal mol^−1^ (**1af-Int-I**→**3af-α**). Such an energetically highly favorable hydrogen atom transfer process renders the *α*-addition step irreversible. On the other hand, a higher energy barrier of  8.0 kcal mol^−1^ (**1af-Int-I’**→**1af-TS-II’**) is required in the hydrogen atom transfer step because of the unmatched polarity. This calculation suggests that both *α*- and *β*-addition products are possible to form and the formation of *α*-addition product (**3af-*α***) is driven by the thermodynamically and kinetically more favorable hydrogen atom transfer step. The LFP experiments were carried out for the reaction of NHC-boryl radical (**I**) and **1af** (see Supplementary Fig. 4), giving the apparent rate constant *k*_add_ = 1.28 × 10^5^ M^−1^ s^−1^. As reported, the hydrogen atom transfer from PhSH to the isopropyl radical is very fast (1.0 × 10^8^ M^−1^ s^−1^). Therefore, it is obvious that the radical addition is much slower than that of hydrogen atom transfer step, which is in good agreement with the DFT studies. In addition, as mentioned above, the reaction of **1ah** bearing a *β*-cyclopropane moiety only gave *α*-addition product. The rate constant for radical addition to this substrate was also measured. As a result, a faster reaction (1.27 × 10^6^ M^−1^ s^−1^, see Supplementary Fig. 5) as compared with **1af** was observed. This suggests that the following facile cyclopropane ring-opening process (1.3 × 10^8^ s^−1^)^61^ may play a role to facilitate the *α*-addition.

The reaction of **1ai**, which has increased steric effect at the *β*-carbon, only provided *α*-addition product **3ai**. The computational study of this reaction shows that both the activation energies for *α*- and *β*-additions, and the energies of the resulting alkyl intermediates are similar (the free-energy profiles are shown in Supplementary Fig. 1). However, the following hydrogen atom transfer is energetically more favorable to the *α*-addition intermediate (+2.4 kcal mol^−1^) than to the *β*-addition intermediate (+7.3 kcal mol^−1^), thus leading to exclusive *α*-regioselectivity. The observed rate constant for this radical addition is 4.2 × 10^4^ M^−1^ s^−1^ (see Supplementary Fig. 6), which is much slower than the hydrogen atom transfer from PhSH to a tertiary alkyl radical (1.4 × 10^8^ M^−1^ s^−1^). Overall, the kinetic studies and computational results agree nicely.

In summary, we have developed a regioselective radical *α*-borylation of *α*,*β*-unsaturated carbonyl compounds. This method enables a general and practical synthesis of a wide range of valuable *α*-boryl esters, amides, ketones, and acids from readily available starting materials. This radical *α*-addition has been rarely achieved for *α*,*β*-unsaturated carbonyl compounds, especially for *β*-alkyl substituted ones. DFT calculations and kinetic studies have been performed to rationalize such regioselectivity. The results indicate that the thermodynamically more favorable *α*-addition step is responsible for the specific *α*-selectivity in *β*-aryl-*α*,*β*-unsaturated carbonyl compounds, and an energetically favored hydrogen atom transfer step accounts for the formation of *α*-borylated products from *β*-alky*-α*,*β*-unsaturated carbonyl compounds. The resulted *α*-borylated products will have potential applications in synthetic and medicinal chemistry. Exploration of the enantioselective radical *α*-borylation reactions is currently ongoing in our lab.

## Methods

### Radical *α*-borylation of *α*,*β*-unsaturated carbonyl compounds

To a 25 ml flame-dried Schlenk flask under nitrogen, NHC-BH_3_ (**2**, 0.500 mmol), substrate **1** (0.600 mmol), AIBN (0.100 mmol), *tert-*dodecanethiol (0.250 mmol), and CH_3_CN (5 ml) were added. The mixture was stirred at 80 °C for 12 h under nitrogen atmosphere. After evaporation of solvent, the resulting crude material was purified by flash column chromatography (silica gel; petroleum ether/ethyl acetate) to give the corresponding product.

### Computational methods

All the calculations were employed at B3LYP level of theory with an empirical dispersion term (Grimme-D3) as implemented in Gaussian 16 software packages. Geometry optimization was carried out with the 6–31 + G(d) basis set in acetonitrile solvent (using  Solvation Model based on Density). Frequency analysis was calculated at the same level of theory to verify the nature of stationary points. For each transition state, the intrinsic reaction coordinate analysis was conducted to ensure that it connects the right reactant and product. To obtain more accurate energies, single-point energy calculations were performed on all optimized structures applying the 6–311 + G(d, p) basis set. Standard state concentrations of 18.9 and 1.0 mol l^−1^ were used for MeCN and all the other species, respectively (see Supplementary References 18–25).

## Supplementary information

Supplentary Information

Description of Additional Supplementary Files

Supplementary Data 1

## Data Availability

The X-ray crystallographic coordinates for **3****f** (CCDC 1866488 [10.5517/ccdc.csd.cc20n797]), **3p-*****β*** (CCDC 1866489 [10.5517/ccdc.csd.cc20n7b8]), **3****u’** (CCDC 1866490 [10.5517/ccdc.csd.cc20n7c9]), and **3aa** (CCDC 1866487 [10.5517/ccdc.csd.cc20n786]) have been deposited at the Cambridge Crystallographic Data Centre (CCDC). These data can be obtained free of charge from the CCDC via www.ccdc.cam.ac.uk/data_request/cif. The experimental procedures, kinetic studies, computational results, and characterization of all new compounds are provided in the Supplementary Information.

## References

[1] Hall, D. G. *Boronic Acids: Preparation and Applications in Organic Synthesis, Medicine and Materials* 2nd edn (Wiley-VCH: Weinheim, 2011).

[2] Burgess K, Ohlmeyer MJ (1991). Transition-metal promoted hydroborations of alkenes, emerging methodology for organic transformations. Chem. Rev..

[3] Beletskaya I, Pelter A (1997). Hydroborations catalysed by transition metal complexes. Tetrahedron.

[4] Crudden CathleenM, Edwards D (2003). Catalytic asymmetric hydroboration: recent advances and applications in carbon−carbon bond-forming reactions. Eur. J. Org. Chem..

[5] Lei Z, Huang Z (2013). Iron-catalyzed alkene hydroboration with Pinacolborane. Synlett.

[6] Buñuel E, Cárdenas DJ (2016). Borylative cyclization reactions. Eur. J. Org. Chem..

[7] Schiffner JA, Müther K, Oestreich M (2010). Enantioselective conjugate borylation. Angew. Chem. Int. Ed..

[8] Mantilli L, Mazet C (2010). Copper-catalyzed asymmetric *β*-boration of *α*,*β*-unsaturated carbonyl derivatives. ChemCatChem.

[9] Calow ADJ, Whiting A (2012). Catalytic methodologies for the *β*-boration of conjugated electron deficient alkenes. Org. Biomol. Chem..

[10] Lee, S. & Yun, J. In *Synthesis and Application of Organoboron Compounds* (eds Fernandez E. & Whiting A.) (Springer, Cham, 2015).

[11] Ng EWH, Low KH, Chiu P (2018). Synthesis and applications of unquaternized C-bound boron enolates. J. Am. Chem. Soc..

[12] Ibrahim MR, Bühl M, Knab R, Schleyer PVR (1992). Vinyloxyborane and its isomers. An ab initio study of the C_2_H_5_BO potential energy surface, the barrier to 1,3-shifts in *β*-ketoboranes, and the mechanism of the carbonylation reaction of boranes. J. Comput. Chem..

[13] He Z, Zajdlik A, Yudin AK (2014). Air- and moisture-stable amphoteric molecules: enabling reagents in synthesis. Acc. Chem. Res..

[14] He Z, Zajdlik A, Yudin AK (2014). α-Borylcarbonyl compounds: from transient intermediates to robust building blocks. Dalton Trans..

[15] St. Denis JD, He Z, Yudin AK (2015). Amphoteric α-boryl aldehyde linchpins in the synthesis of heterocycles. ACS Catal..

[16] Ansorge A, Brauer DJ, Bürger H, Hagen T, Pawelke G (1993). BNC Isosteres of cyclopropane and a borylated diazoester from diazo compounds and (dimethylamino)bis(trifluoromethyl)borane. Angew. Chem. Int. Ed..

[17] Brauer, D. J., Bürger, H., Buchheim-Spiegel, S. & Pawelke, G. New azoniaboratacyclopropanes from (F_3_C)_2_BNMe_2_ and diazomethane derivatives - structure of *cyclo*-(F_3_C)_2_B-CPh_2_-NMe_2_ and HOB(CF_3_)_2_-CHC_6_F_5_-NHMe_2_. *Eur. J. Inorg. Chem*. **1999**, 255–261 (1999).

[18] Bell, N. J. et al. Platinum catalysed 3,4- and 1,4-diboration of *α*,*β*-unsaturated carbonyl compounds using *bis*-pinacolatodiboron. *Chem. Commun*. **10**, 1854–1855 (2004). 10.1039/b406052k 15306917

[19] Caskey SR, Stewart MH, Johnson MJA, Kampf JW (2006). Carbon–carbon bond formation at a neutral terminal carbido ligand: generation of cyclopropenylidene and vinylidene complexes. Angew. Chem. Int. Ed..

[20] Bai J, Burke LD, Shea KJ (2007). BH_3_-catalyzed oligomerization of ethyl diazoacetate: the role of C-boron enolates. J. Am. Chem. Soc..

[21] He Z, Yudin AK (2011). Amphoteric α-boryl aldehydes. J. Am. Chem. Soc..

[22] Li J, Burke MD (2011). Pinene-derived iminodiacetic acid (PIDA): a powerful ligand for stereoselective synthesis and iterative cross-coupling of C(sp^3^) boronate building blocks. J. Am. Chem. Soc..

[23] Lv WX (2016). Oxidative difunctionalization of alkenyl MIDA boronates: a versatile platform for halogenated and trifluoromethylated α-boryl ketones. Angew. Chem. Int. Ed..

[24] Corless VB (2018). Synthesis of *α*-borylated ketones by regioselective wacker oxidation of alkenylboronates. Org. Lett..

[25] Li X, Curran DP (2013). Insertion of reactive rhodium carbenes into boron–hydrogen bonds of stable *N*-heterocyclic carbene boranes. J. Am. Chem. Soc..

[26] Allen TH, Kawamoto T, Gardner S, Geib SJ, Curran DP (2017). N-Heterocyclic carbene boryl iodides catalyze insertion reactions of N-Heterocyclic carbene boranes and diazoesters. Org. Lett..

[27] Cheng QQ, Zhu SF, Zhang YZ, Xie XL, Zhou QL (2013). Copper-catalyzed B–H bond insertion reaction: a highly efficient and enantioselective C–B bond-forming reaction with amine–borane and phosphine–borane adducts. J. Am. Chem. Soc..

[28] Cheng QQ, Xu H, Zhu SF, Zhou QL (2015). Enantioselective copper-catalyzed B-H bond insertion reaction of *α*-Diazoketones. Acta Chim. Sin..

[29] Yang JM (2018). Gold-catalyzed oxidative coupling of terminal alkynes and borane adducts: efficient synthesis of α-boryl ketones. ACS Catal..

[30] Chen D, Zhang X, Qi WY, Xu B, Xu MH (2015). Rhodium(I)-catalyzed asymmetric carbene insertion into B–H bonds: highly enantioselective access to functionalized organoboranes. J. Am. Chem. Soc..

[31] Kan SBJ, Huang X, Gumulya Y, Chen K, Arnold FH (2017). Genetically programmed chiral organoborane synthesis. Nature.

[32] Radcliffe JE, Fasano V, Adams RW, You P, Ingleson MJ (2019). Reductive α-borylation of α,β-unsaturated esters using NHC–BH_3_ activated by I_2_ as a metal-free route to α-boryl esters. Chem. Sci..

[33] Walton JC (2009). Linking borane with N-heterocyclic carbenes: effective hydrogen-atom donors for radical reactions. Angew. Chem. Int. Ed..

[34] Curran DP (2011). Synthesis and reactions of N-heterocyclic carbene boranes. Angew. Chem. Int. Ed..

[35] Ueng SH (2009). N-Heterocyclic carbene boryl radicals: a new class of boron-centered radical. J. Am. Chem. Soc..

[36] Watanabe T, Hirose D, Curran DP, Taniguchi T (2017). Borylative radical cyclizations of benzo[3,4]cyclodec-3-ene-1,5-diynes and N-heterocyclic carbene-boranes. Chem. Eur. J..

[37] Pan X (2013). Mechanistic and preparative studies of radical chain homolytic substitution reactions of N-heterocyclic carbene boranes and disulfides. J. Am. Chem. Soc..

[38] Kawamoto T, Geib SJ, Curran DP (2015). Radical reactions of N-heterocyclic carbene boranes with organic nitriles: cyanation of NHC-boranes and reductive decyanation of malononitriles. J. Am. Chem. Soc..

[39] Shimoi M, Watanabe T, Maeda K, Curran DP, Taniguchi T (2018). Radical *trans*-hydroboration of alkynes with N-heterocyclic carbene boranes. Angew. Chem. Int. Ed..

[40] Zhou N, Yuan XA, Zhao Y, Xie J, Zhu C (2018). Synergistic photoredox catalysis and organocatalysis for inverse hydroboration of imines. Angew. Chem. Int. Ed..

[41] Ueng SH (2008). Complexes of borane and N-heterocyclic carbenes: a new class of radical hydrogen atom donor. J. Am. Chem. Soc..

[42] Ren SC (2017). Radical borylation/cyclization cascade of 1,6-enynes for the synthesis of boron-handled hetero- and carbocycles. J. Am. Chem. Soc..

[43] Qi J (2018). Radical borylative cyclization of 1,6-dienes: synthesis of boron-substituted six-membered heterocycles and carbocycles. Org. Lett..

[44] Tehfe MA (2010). N-Heterocyclic carbenes−borane complexes: a new class of initiators for radical photopolymerization. Macromolecules.

[45] Pan X, Lacôte E, Lalevée J, Curran DP (2012). Polarity reversal catalysis in radical reductions of halides by N-heterocyclic carbene boranes. J. Am. Chem. Soc..

[46] Lacôte E, Curran DP, Lalevée J (2012). NHC-Boranes: air- and water-tolerant co-initiators for type II photopolymerizations. Chimia.

[47] Telitel S (2015). Influence of electronic effects on the reactivity of triazolylidene-boryl Radicals: consequences for the use of N-heterocyclic carbene boranes in organic and polymer synthesis. Chem. Eur. J..

[48] Wu C, Hou X, Zheng Y, Li P, Lu D (2017). Electrophilicity and nucleophilicity of boryl radicals. J. Org. Chem..

[49] Srikanth GSC, Castle SL (2005). Advances in radical conjugate additions. Tetrahedron.

[50] Fischer H, Radom L (2001). Factors controlling the addition of carbon-centered radicals to alkenes—an experimental and theoretical perspective. Angew. Chem. Int. Ed..

[51] Subervie D (2018). Difluorination at boron leads to the first electrophilic ligated boryl radical (NHC-BF_2_•). Angew. Chem. Int. Ed..

[52] Renaud P, Gerster M (1998). Use of Lewis acids in free radical reactions. Angew. Chem. Int. Ed..

[53] Sibi MP, Manyem S, Zimmerman J (2003). Enantioselective radical processes. Chem. Rev..

[54] Srikrishna, A. in *Radicals in Organic Synthesis* (eds Renaud P. & Sibi M. P.) (Wiley-VCH, 2001).

[55] Liu L, Chen Q, Wu YD, Li C (2005). 8-Endo versus 7-exo cyclization of α-carbamoyl radicals. A combination of experimental and theoretical studies. J. Org. Chem..

[56] Bonet A, Odachowski M, Leonori D, Essafi S, Aggarwal VK (2014). Enantiospecific sp^2^–sp^3^ coupling of secondary and tertiary boronic esters. Nat. Chem..

[57] Llaveria J, Leonori D, Aggarwal VK (2015). Stereospecific coupling of boronic esters with N-heteroaromatic compounds. J. Am. Chem. Soc..

[58] Nerkar S, Curran DP (2015). Synthesis and suzuki reactions of N-heterocyclic carbene difluoro(aryl)-boranes. Org. Lett..

[59] Menon AS, Wood GPF, Moran D, Radom L (2007). Bond dissociation energies and radical stabilization energies: an assessment of contemporary theoretical procedures. J. Phys. Chem. A.

[60] Dénès F, Pichowicz M, Povie G, Renaud P (2014). Thiyl radicals in organic synthesis. Chem. Rev..

[61] Griller D, Ingold KU (1980). Free-radical clocks. Acc. Chem. Res..

