# Supplementary material for: Regioselective radical α-borylation of α,β-unsaturated carbonyl compounds for direct synthesis of α-borylcarbonyl molecules
Source: Nat Commun. 2019 Apr 29;10:1934. doi: 10.1038/s41467-019-09825-3 (PMC6488649; doi:10.1038/s41467-019-09825-3)
Supplement: Supplementary file 1 — Supplentary Information [file 41467_2019_9825_MOESM1_ESM.pdf]

Supplementary Information

Regioselective radical *α*-borylation of *α*,*β*-unsaturated carbonyl compounds for direct synthesis of *α*-borylcarbonyl molecules

Ren *et al*.

1. Supplementary Methods

General Information

^{1}H NMR (400 MHz) spectra were recorded on a Bruker Avance 400 spectrometer in $CDCl_3$ [using $CDCl_3$ (for ^{1}H, δ = 7.26). ^{13}C NMR (100 MHz) spectra on a Bruker Avance 400 spectrometer in $CDCl_3$ [using $CDCl_3$ (for ^{13}C, δ = 77.0) as internal standard]. The following abbreviations were used to explain the multiplicities: s = singlet, d = doublet, t = triplet, q = quartet, dd = doublet of doublet, ddd = doublet of doublet of doublet, dt = doublet of triplet, m = multiplet, s br = single broad. High-resolution mass spectra were obtained with a Water XEVO G2 Q-Tof (Waters Corporation). X-ray crystallography analysis was performed on Bruker X8 APEX X-ray diffractionmeter. Melting points were uncorrected and were recorded on a Buchi B-54 melting point apparatus. Flash column chromatography was performed using Merck silica gel 60 with distilled solvents. Commercially available reagents were purchased from Energy Chemical, J & K Scientific, Adamas-beta and Sigma-Aldrich Co., Inc.

NHC-BH_3 were prepared according to the reported procedures.[1] *α,β*-Unsaturated carbonyl compounds **1b**,[2] **1c**,[3] **1d**,[4] **1e**,[5] **1g**,[6] **1h**,[7] **1l**,[8] **1m**,[9] **1o**,[10] **1p**,[11] **1q**,[12] **1r**,[12] **1t**,[13] **1ac**,[14] **1ag**,[15] **1ah**,[16] **1ak**,[17] were known compounds and prepared according to the literature procedures. *α,β*-Unsaturated carbonyl compounds **1a**, **1f**, **1n**, **1ab**, **1ae**, **1af**, **1aj** were purchased and used directly without additional purification.

Computational methods

All the calculations were employed at B3LYP level of theory[18] with an empirical dispersion term (Grimme-D3)[19] as implemented in Gaussian 09 software packages.[20] Geometry optimization was carried out with the 6-31+G(d) basis set in acetonitrile solvent (using SMD solvation model[21]). Frequency analysis was calculated at the same level of theory to verify the nature of stationary points. For each transition state, the intrinsic reaction coordinate (IRC) analysis was conducted to ensure that it connects the right reactant and product.[22,23] To obtain more accurate energies, single-point energy calculations were performed on all optimized structures applying the 6-311+G(d,p) basis set.[24] Standard state concentrations of 18.9[25] and 1.0 mol/L were used for MeCN and all the other species, respectively.

Note: In the ^{1}H NMR spectral data, the protons on boron are not listed due to quadrupole broadening and spin–spin coupling with boron.

2. Synthesis of *α,β*-unsaturated carbonyl compounds
General procedure A:

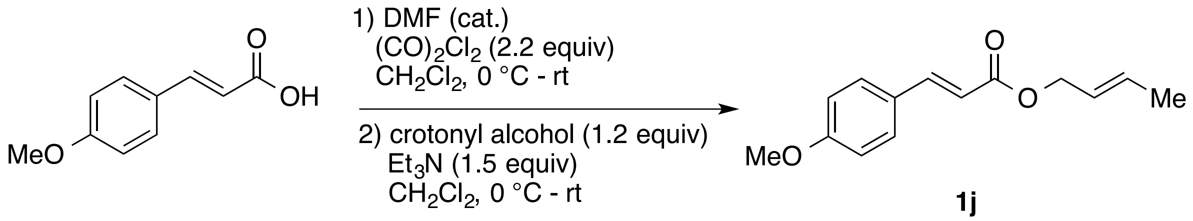

To a solution of (*E*)-3-(4-methoxyphenyl)acrylic acid (1.792 g, 10.05 mmol) and drops of DMF (cat.) in CH_2Cl_2 was slowly added $(CO)_2Cl_2$ (1.9 mL, 22.45 mmol) at 0 °C. The reaction mixture was warm to room temperature, and stirred under nitrogen atmosphere for 2 h. The solvent was removed under *vacuo* to afford a crude acyl chloride product. The crude product was dissolved in CH_2Cl_2, Et_3N (1.8 mL, 13.95 mmol) and crotonyl alcohol (0.85 mL, 9.96 mmol) was added. The reaction mixture was stirred at room temperature before it was cooled to 0 °C and quenched with water. The aqueous layer was extracted with CH_2Cl_2 three times. The combined organic extracts was washed with brine, dried over Na_2SO_4, and concentrated in *vacuo*. The crude residue was purified by flash column chromatography on silica gel (petroleum ether : ethyl acetate = 95:5) to give **1j** (1.649 g) in 71% yield as a colorless liquid. ^{1}H NMR (400 MHz, $CDCl_3$) δ 1.72-1.77 (m, 3H), 3.83 (s, 3H), 4.60-4.65 (m, 2H), 5.61-5.72 (m, 1H), 5.79-5.90 (m, 1H), 6.32 (d, *J* = 16.0 Hz, 1H), 6.90 (d, *J* = 8.8 Hz, 2H), 7.47 (d, *J* = 8.8 Hz, 2H), 7.65 (d, *J* = 16.0 Hz, 1H); ^{13}C NMR (100 MHz, $CDCl_3$) δ 17.8, 55.3, 65.1, 114.3, 115.6, 125.3, 127.2, 129.7, 131.3, 144.5, 161.4, 167.1; ESI-HRMS (*m/z*): $(M+Na)^+$ calcd for $C_{14}H_{16}NaO_3$, 255.0992; found: 255.0990.

But-3-yn-1-yl cinnamate (1i)

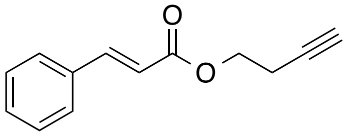

Following general procedure A, **1i** was obtained in 53% yield (530.0 mg, 2.65 mmol) from the reaction of cinnamic acid (740.0 mg, 4.99 mmol) and but-3-yn-1-ol (417 mg, 5.95 mmol); colorless liquid; ^{1}H NMR (400 MHz, $CDCl_3$) δ 2.04 (t, *J* = 2.8 Hz, 1H), 2.61 (dt, *J* = 2.8, 6.8 Hz, 2H), 4.32 (t, *J* = 6.8 Hz, 2H), 6.45 (d, *J* = 16.0 Hz, 1H), 7.33-7.41 (m, 3H), 7.48-7.57 (m, 2H), 7.71 (d, *J* = 16.0 Hz, 1H); ^{13}C NMR (100 MHz, $CDCl_3$) δ 19.0, 62.1, 69.9, 80.1, 117.6,

128.0, 128.8, 130.3, 134.2, 145.2, 166.5; ESIHRMS: Found: *m/z* 201.0912. Calcd for $C_{13}H_{13}O_2$: $(M+H)^+$ 201.0916.

***N*-(But-3-en-1-yl)cinnamamide (1s)**

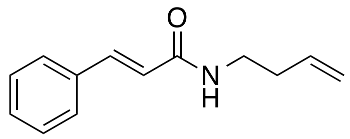

Following general procedure A, **1s** was obtained in 73% yield (733.7 mg, 3.64 mmol) from the reaction of cinnamic acid (745.0 mg, 5.03 mmol) and but-3-en-1-amine (707.9 mg, 9.95 mmol); yellow solid, mp: 63-64 °C; ^{1}H NMR (400 MHz, $CDCl_3$) δ 2.29-2.38 (m, 2H), 3.43-3.51 (m, 2H), 5.08-5.17 (m, 2H), 5.71 (s br, 1H), 5.78-5.88 (m, 1H), 6.38 (d, J = 15.6 Hz, 1H), 7.31-7.39 (m, 3H), 7.46-7.52 (m, 2H), 7.62 (d, J = 15.6 Hz, 1H); ^{13}C NMR (100 MHz, $CDCl_3$) δ 33.7, 38.6, 117.4, 120.6, 127.8, 128.8, 129.6, 134.8, 135.3, 150.0, 165.8; ESIHRMS: Found: *m/z* 224.1045. Calcd for $C_{13}H_{15}NNaO$: $(M+Na)^+$ 224.1046.

***N*-(Prop-2-yn-1-yl)-*N*-tosylcinnamamide (1u)**

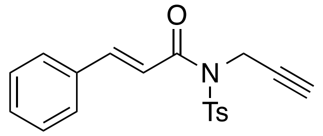

Following general procedure A, **1u** was obtained in 61% yield (1.032 g, 3.04 mmol) from the reaction of cinnamic acid (756.8 mg, 5.11 mmol) and 4-methyl-*N*-(prop-2-yn-1-yl)benzenesulfonamide (1.046 g, 5.00 mmol)[26]; white solid, mp: 75-76 °C; ^{1}H NMR (400 MHz, $CDCl_3$) δ 2.31 (t, J = 2.4 Hz, 1H), 2.40 (s, 3H), 4.75 (d, J = 2.4 Hz, 2H), 7.24-7.34 (m, 3H), 7.35-7.42 (m, 3H), 7.45-7.53 (m, 2H), 7.69 (d, J = 15.6 Hz, 1H), 7.93 (d, J = 8.4 Hz, 2H); ^{13}C NMR (100 MHz, $CDCl_3$) δ 21.6, 35.4, 72.5, 78.4, 117.4, 127.8, 128.4, 129.0, 129.8, 130.7, 134.3, 136.5, 145.1, 146.7, 165.2; ESIHRMS: Found: *m/z* 362.0818. Calcd for $C_{19}H_{17}NNaO_3S$: $(M+Na)^+$ 362.0814.

(*E*)-3-(4-Methoxyphenyl)-*N*-(3-methylbut-2-en-1-yl)-*N*-tosylacrylamide (1v)

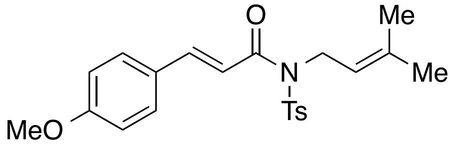

Following general procedure A, **1v** was obtained in 62% yield (1.992 g, 4.99 mmol) from the reaction of (*E*)-3-(4-methoxyphenyl)acrylic acid (1.430 g, 8.03 mmol) and

4-methyl-*N*-(3-methylbut-2-en-1-yl) benzenesulfonamide[27] (1.910 g, 7.98 mmol); white solid, mp: 90-91 °C; ^{1}H NMR (400 MHz, $CDCl_3$) δ 1.74 (s, 3H), 1.79 (s, 3H), 2.40 (s, 3H), 3.84 (s, 3H), 4.53 (d, *J* = 6.8 Hz, 2H), 5.22-5.32 (m, 1H), 6.89 (d, *J* = 8.4 Hz, 2H), 7.11 (d, *J* = 15.6 Hz, 1H), 7.29 (d, *J* = 8.4 Hz, 2H), 7.44 (d, *J* = 8.4 Hz, 2H), 7.63 (d, *J* = 15.6 Hz, 1H), 7.80 (d, *J* = 8.4 Hz, 2H); ^{13}C NMR (100 MHz, $CDCl_3$) δ 18.0, 21.6, 25.7, 44.7, 55.4, 114.4, 115.7, 120.0, 127.3, 127.6, 129.6, 130.0, 136.4, 137.4, 144.5, 145.5, 161.6, 166.2; ESIHRMS: Found: *m/z* 422.1391. Calcd for $C_{22}H_{25}NNaO_4S$: $(M+Na)^+$ 422.1397.

General procedure B:

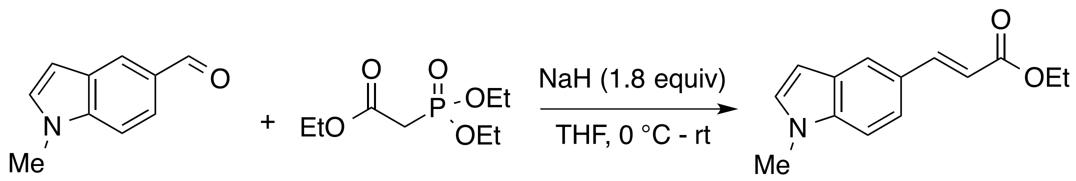

To a solution of ethyl 2-(diethoxyphosphoryl)acetate (1.5 mL, 7.56 mmol) in dry THF (30 mL) was added NaH (360 mg, 9.00 mmol) slowly in 5 minutes at 0 °C under nitrogen atmosphere. The reaction mixture was stirred at 0 °C for 30 minutes, then 1-methyl-1*H*-indole-5-carbaldehyde (796.0 mg, 5.00 mmol) was added and allowed warm to room temperature. The reaction mixture was stirred overnight before it was cooled to 0 °C and quenched with water. The aqueous layer was extracted with EA three times. The combined organic extracts was washed with brine, dried over Na_2SO_4, and concentrated in *vacuo*. The crude residue was purified by flash column chromatography on silica gel to give ethyl (*E*)-3-(1-methyl-1*H*-indol-5-yl)acrylate **1k** (972.6 mg) in 85% yield as a pale yellow liquid; ^{1}H NMR (400 MHz, $CDCl_3$) δ 1.35 (t, *J* = 7.2 Hz, 3H), 3.81 (s, 3H), 4.27 (q, *J* = 7.2 Hz, 2H), 6.41 (d, *J* = 16.0 Hz, 1H), 6.49-6.54 (m, 1H), 7.07 (d, *J* = 2.8 Hz, 1H), 7.31 (d, *J* = 8.4 Hz, 1H), 7.41-7.49 (m, 1H), 7.79 (s, 1H), 7.84 (d, *J* = 16.0 Hz, 1H); ^{13}C NMR (100 MHz, $CDCl_3$) δ 14.4, 33.0, 60.2, 102.0, 109.7, 114.9, 121.1, 122.5, 126.1, 128.7, 129.9, 137.8, 146.5, 167.7; ESIHRMS: Found: *m/z* 252.0992. Calcd for $C_{14}H_{15}NNaO_2$: $(M+Na)^+$ 252.0995.

Ethyl (*E*)-3-(cyclohex-3-en-1-yl)acrylate (1ai)

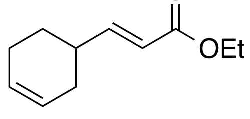

Following general procedure B, **1ai** was obtained in 85% yield (1.537 g, 8.53 mmol)) from the reaction of cyclohex-3-ene-1-carbaldehyde (1.034 g, 9.39 mmol); colorless liquid; ^{1}H

NMR (400 MHz, $CDCl_3$) δ 1.29 (t, *J* = 7.2 Hz, 3H), 1.40-1.52 (m, 1H), 1.77-1.98 (m, 2H), 2.04-2.21 (m, 3H), 2.37-2.49 (m, 1H), 4.19 (q, *J* = 6.8 Hz, 2H), 5.63-5.74 (m, 2H), 5.77-5.85 (m, 1H), 6.97 (dd, *J* = 7.2, 16.0 Hz, 1H); ^{13}C NMR (100 MHz, $CDCl_3$) δ 14.3, 24.4, 27.5, 30.1, 36.4, 60.2, 119.6, 125.3, 127.0, 153.2, 167.0; ESIHRMS: Found: *m/z* 203.1042. Calcd for $C_{11}H_{16}NaO_2$: $(M+Na)^+$ 203.1043.

General procedure C:

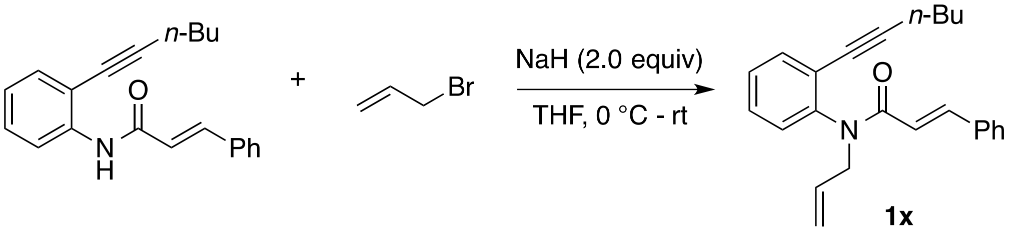

1x

To a solution of *N*-(2-(hex-1-yn-1-yl)phenyl)cinnamamide (641.2 mg, 2.11 mmol, prepared from 2-(hex-1-yn-1-yl)aniline[29] and cinnamoyl chloride following general procedure A and used without purification) in dry THF(10 mL) was added NaH (177.0 mg, 4.43 mmol) slowly at 0 °C under nitrogen atmosphere. The reaction mixture was stirred at 0 °C for 20 minutes, 3-bromoprop-1-ene (0.3 mL, 3.47 mmol) was then added and allowed warm to room temperature. The reaction mixture was stirred overnight before it was cooled to 0 °C and quenched with water. The aqueous layer was extracted with ethyl acetate three times. The combined organic extracts was washed with brine, dried over Na_2SO_4, and concentrated in *vacuo*. The crude residue was purified by flash column chromatography on silica gel to give **1x (**696.7 mg) in 96% yield as a pale yellow liquid; ^1H NMR (400 MHz, $CDCl_3$) δ 0.87 (t, *J* = 7.2 Hz, 3H), 1.34-1.45 (m, 2H), 1.46-1.57 (m, 2H), 2.36 (t, *J* = 6.8 Hz, 2H), 4.12 (tdd, *J* = 1.2, 6.8, 14.4 Hz, 1H), 4.77 (tdd, *J* = 1.6, 5.6, 14.4 Hz, 1H), 5.05-5.09 (m, 1H), 5.10-5.15 (m, 1H), 5.86-6.03 (m, 1H), 6.20 (d, *J* = 15.6 Hz, 1H), 7.13-7.20 (m, 1H), 7.23-7.38 (m, 7H), 7.47-7.56 (m, 1H), 7.68 (d, *J* = 15.6 Hz, 1H); ^{13}C NMR (100 MHz, $CDCl_3$) δ 13.5, 19.2, 21.9, 30.5, 51.6, 76.8, 96.8, 117.8, 119.0, 124.1, 127.8, 127.9, 128.3, 128.6, 129.3, 129.6, 133.2, 133.3, 135.4, 141.7, 142.9, 165.7; ESIHRMS: Found: *m/z* 344.2015. Calcd for $C_{24}H_{26}NO$: $(M+H)^+$ 344.2014.

***N*-(2-(Hex-1-yn-1-yl)phenyl)-*N*-methylcinnamamide (1w)**

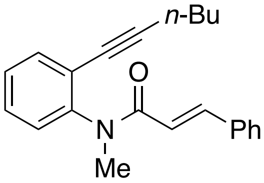

Following general procedure C, **1w** was obtained in 83% yield (835.3 mg, 2.63 mmol) from the reaction of *N*-(2-(hex-1-yn-1-yl)phenyl)cinnamamide (965.0 mg, 3.18 mmol) and iodomethane (684.0 mg, 4.82 mmol); white solid, mp: 58-59 °C; ^{1}H NMR (400 MHz, $CDCl_3$) δ 0.88 (t, *J* = 7.2 Hz, 3H), 1.34-1.45 (m, 2H), 1.47-1.57 (m, 2H), 2.36 (t, *J* = 6.8 Hz, 2H), 3.36 (s, 3H), 6.23 (d, *J* = 15.6 Hz, 1H), 7.18-7.23 (m, 1H), 7.25-7.37 (m, 7H), 7.48-7.53 (m, 1H), 7.67 (d, *J* = 15.6 Hz, 1H); ^{13}C NMR (100 MHz, $CDCl_3$) δ 13.5, 19.2, 21.9, 30.5, 36.4, 76.4, 96.6, 118.8, 123.5, 127.8, 127.9, 128.5, 128.6, 128.7, 129.3, 133.3, 135.4, 141.3, 144.7, 166.2; ESIHRMS: Found: *m/z* 318.1861. Calcd for $C_{22}H_{24}NO$: $(M+H)^+$ 318.1858.

(*E*)-3-(4-Chlorophenyl)-*N*-(2-(hex-1-yn-1-yl)phenyl)-*N*-methylacrylamide (1y)

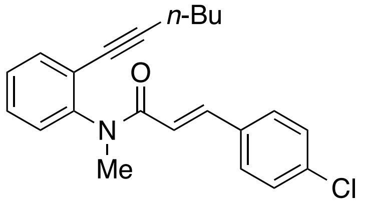

Following general procedure C, **1y** was obtained in 50% yield (972.7 mg, 2.64 mmol) from the reaction of (*E*)-3-(4-chlorophenyl)-*N*-(2-(hex-1-yn-1-yl)phenyl)acrylamide [prepared from 2-(hex-1-yn-1-yl)aniline[20] (955.0 mg, 5.51 mmol) and (*E*)-3-(4-chlorophenyl)acryloyl chloride following general procedure A and used without purification] and iodomethane (1.186 g, 8.35 mmol); pale yellow liquid; ^{1}H NMR (400 MHz, $CDCl_3$) δ 0.87 (t, *J* = 7.2 Hz, 3H), 1.33-1.44 (m, 2H), 1.45-1.55 (m, 2H), 2.35 (t, *J* = 6.8 Hz, 2H), 3.35 (s, 3H), 6.22 (d, *J* = 15.6 Hz, 1H), 7.18-7.25 (m, 5H), 7.28-7.36 (m, 2H), 7.48-7.53 (m, 1H), 7.60 (d, *J* = 15.6 Hz, 1H); ^{13}C NMR (100 MHz, $CDCl_3$) δ 13.5, 19.1, 21.9, 30.5, 36.5, 76.4, 96.6, 119.3, 123.5, 128.0, 128.4, 128.7, 128.8, 128.9, 133.3, 133.8, 135.1, 140.0, 144.5, 165.9; ESIHRMS: Found: *m/z* 352.1451. Calcd for $C_{22}H_{23}NOCl$: $(M+H)^+$ 352.1468.

***N*-(4-Chloro-2-(hex-1-yn-1-yl)phenyl)-*N*-methylcinnamamide (1z)**

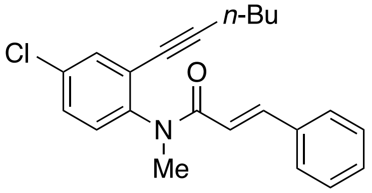

Following general procedure C, **1z** was obtained in 45% yield (1.930 g, 5.48 mmol) from the reaction of *N*-(4-chloro-2-(hex-1-yn-1-yl)phenyl)cinnamamide (prepared from 4-chloro-2-(hex-1-yn-1-yl)aniline[20] (2.520 g, 11.37 mmol) and cinnamoyl chloride following general procedure A and used without purification) and iodomethane (2.736 g, 19.28 mmol); pale yellow solid, mp: 67-68 °C; ^{1}H NMR (400 MHz, $CDCl_3$) δ 0.87 (t, *J* = 7.2 Hz, 3H),

1.32-1.43 (m, 2H), 1.42-1.55 (m, 2H), 2.35 (t, J = 6.8 Hz, 2H), 3.33 (s, 3H), 6.21 (d, J = 15.6 Hz, 1H), 7.14 (d, J = 8.4 Hz, 1H), 7.27-7.36 (m, 6H), 7.46-7.51 (m, 1H), 7.68 (d, J = 15.6 Hz, 1H); ^{13}C NMR (100 MHz, $CDCl_3$) δ 13.5, 19.1, 21.9, 30.3, 36.5, 75.4, 98.1, 118.3, 125.1, 127.8, 128.6, 128.9, 129.5, 129.7, 133.0, 133.5, 141.9 (overlapped), 143.3, 166.1; ESIHRMS: Found: *m/z* 352.1470. Calcd for $C_{22}H_{23}ClNO$: $(M+H)^+$ 352.1468.

(*E*)-*N*-(2-(Hex-1-yn-1-yl)phenyl)-*N*-methyl-3-(pyridin-3-yl)acrylamide (1aa)

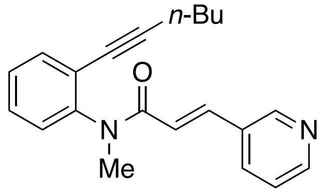

Following general procedure C, **1aa** was obtained in 16% yield (384.7 mg, 1.21 mmol) from the reaction of (*E*)-*N*-(2-(hex-1-yn-1-yl)phenyl)-3-(pyridin-3-yl)acrylamide (prepared from 2-(hex-1-yn-1-yl)aniline[20] (1.300 g, 7.51 mmol) and (*E*)-3-(pyridin-3-yl)acryloyl chloride following general procedure A and used without purification) and iodomethane (1.596 g, 11.24 mmol)); pale yellow liquid; ^{1}H NMR (400 MHz, $CDCl_3$) δ 0.88 (t, J = 7.2 Hz, 3H), 1.33-1.46 (m, 2H), 1.46-1.57 (m, 2H), 2.36 (t, J = 6.8 Hz, 2H), 3.38 (s, 3H), 6.32 (d, J = 15.6 Hz, 1H), 7.17-7.24 (m, 2H), 7.30-7.39 (m, 2H), 7.50-7.54 (m, 1H), 7.57 (td, J = 2.0, 8.0 Hz, 1H), 7.65 (d, J = 15.6 Hz, 1H), 8.48 (dd, J = 1.2, 4.8 Hz, 1H), 8.56 (d, J = 1.2 Hz, 1H); ^{13}C NMR (100 MHz, $CDCl_3$) δ 13.4, 19.0, 21.8, 30.4, 36.4, 76.2, 96.5, 120.8, 123.3, 123.4, 128.0, 128.2, 128.7, 131.0, 133.2, 134.0, 137.5, 144.2, 149.2, 149.9, 165.3; ESIHRMS: Found: *m/z* 319.1816. Calcd for $C_{21}H_{23}N_2O$: $(M+H)^+$ 319.1810.

(E)-1-(4-Hydroxy-3-methoxyphenyl)-3-(3,4,5-trimethoxyphenyl)prop-2-en-1-one (1ad)

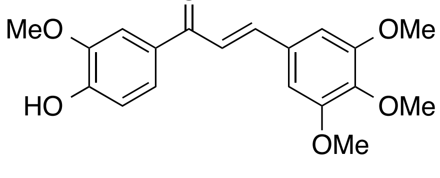

Following the reported literature;[28] **1ad** was obtained in 41% yield (697.8 mg, 2.03 mmol) from the reaction of 1-(4-hydroxy-3-methoxyphenyl)ethan-1-one (4.905 g, 29.52 mmol) and 3,4,5-trimethoxybenzaldehyde 830.1 mg, 4.23 mmol); pale yellow solid, mp: 179-180 °C; ^{1}H NMR (400 MHz, $CDCl_3$) δ 3.90 (s, 3H), 3.92 (s, 6H), 3.98 (s, 3H), 6.86 (s, 2H), 7.00 (d, J = 8.0 Hz, 1H), 7.43 (d, J = 15.6 Hz, 1H), 7.61-7.67 (m, 2H), 7.72 (d, J = 15.6 Hz, 1H); ^{13}C NMR (100 MHz, $CDCl_3$) δ 56.2, 56.3, 61.0, 105.6, 110.5, 113.7, 121.0, 123.7, 130.6, 131.1, 140.3, 144.1, 147.0, 150.4, 153.5, 188.5; ESIHRMS: Found: *m/z z* 345.1333. Calcd for

$C_{19}H_{21}O_6$: $(M+H)^+$ 345.1338.

3. Radical boryaltion of *α,β*-unsaturated compounds

General procedure:

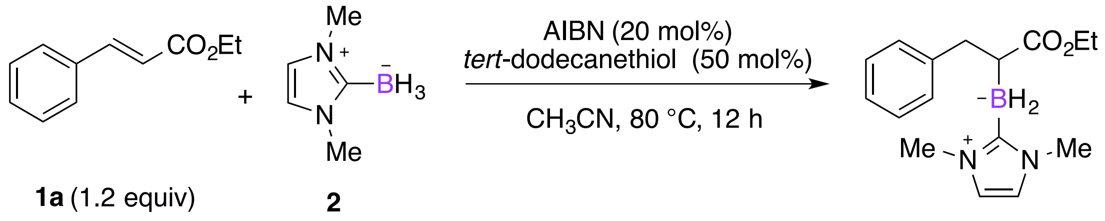

A solution of **1a** (105.9 mg, 0.601 mmol), **2** (55.1 mg, 0.501 mmol), AIBN (16.5 mg, 0.100 mmol) and *tert*-dodecanethiol (50.7 mg, 0.251 mmol) in CH_3CN (5 mL) was stirred at 80 °C for 12 h under nitrogen atmosphere. After evaporation of solvent, the resulting crude material was purified by flash column chromatography (silica gel; petroleum ether : ethyl acetate = 70 : 30) to give product **3a** (115.8 mg) in 81% yield as a colorless liquid.

(1,3-Dimethyl-1*H*-imidazol-3-ium-2-yl)(1-ethoxy-1-oxo-3-phenylpropan-2-yl)dihydroborate (3a)

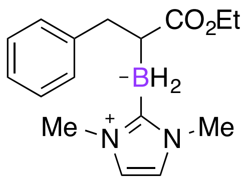

^{1}H NMR (400 MHz, $CDCl_3$) δ 0.98 (t, J = 7.2 Hz, 3H), 2.19 (m, BH_2CH, 1H), 2.72 (dd, J = 4.0, 14.4 Hz, 1H), 3.11 (dd, J = 10.8, 14.8 Hz, 1H), 3.74 (s, 6H), 3.76-3.86 (m, 2H), 6.80 (s, 2H), 7.1-7.13 (m, 1H), 7.16-7.24 (m, 4H); ^{13}C NMR (100 MHz, $CDCl_3$) δ 14.3, 36.0, 39.1, 58.5, 120.3, 125.0, 127.8, 128.5, 144.8, 181.6; ^{11}B NMR (128.4 MHz, $CDCl_3$): δ -25.0 (t, J = 90.4 Hz, 1B); ESIHRMS: Found: *m/z* 309.1747. Calcd for $C_{16}H_{23}{}^{11}BN_2NaO_2$: $(M+Na)^+$ 309.1750.

(1,4-Dimethyl-1*H*-1,2,4-triazol-4-ium-5-yl)(1-ethoxy-1-oxo-3-phenylpropan-2-yl)dihydroborate (3a-B)

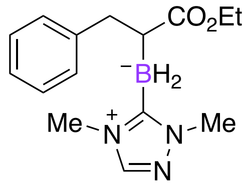

According to the general procedure, the reaction of **1a** (63.0 mg, 0.358 mmol), (1,4-dimethyl-4H-1,2,4-triazol-1-ium-5-yl)trihydroborate (33.2 mg, 0.299 mmol), AIBN (9.6 mg, 0.058 mmol), PhSH (6.4 mg, 0.058 mmol), and MeCN (3 mL), under nitrogen at 80 °C for 12 h after flash column chromatography on silica gel (ethyl acetate : MeCN = 60 : 40) to afford 76.5 mg (89%) of **3a-B** as colorless oil. ^{1}H NMR (400 MHz, $CDCl_3$) δ 0.89 (t, *J* = 7.2 Hz, 3H), 2.12-2.26 (m, 1H), 2.74 (dd, *J* = 4.8, 14.4 Hz, 1H), 3.11 (dd, *J* = 10.0, 14.4 Hz, 2H), 3.73 (s, 3H), 3.78-3.87 (m, 2H), 3.94 (s, 3H), 7.06-7.14 (m, 1H), 7.18-7.24 (m, 4H), 7.86 (s, 1H). ^{13}C NMR (100 MHz, $CDCl_3$) δ 14.2, 33.7, 38.2, 38.9, 58.7, 125.1, 127.8, 128.5, 141.3, 144.3, 181.1; ^{11}B NMR (128.4 MHz, $CDCl_3$): δ -25.5 (t, *J* = 92.6 Hz, 1B); ESIHRMS: Found: *m/z* 310.1695. Calcd for $C_{15}H_{22}{}^{11}BN_3NaO_2$: $(M+Na)^+$ 310.1703.

(1,3-Diisopropyl-1*H*-imidazol-3-ium-2-yl)(1-ethoxy-1-oxo-3-phenylpropan-2-yl)dihydro borate (3a-C)

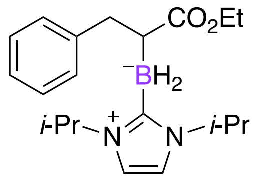

According to the general procedure, the reaction of **1a** (63.0 mg, 0.358 mmol), (1,3-diisopropyl-1H-imidazol-3-ium-2-yl)trihydroborate (49.7 mg, 0.299 mmol), AIBN (9.7 mg, 0.06 mmol), PhSH (6.4 mg, 0.058 mmol), and MeCN (3 mL), under nitrogen at 80 °C for 12 h after flash column chromatography on silica gel (ethyl acetate : MeCN = 80 : 20) to afford 71.6 mg (70%) of **3a-C** as white solid, mp: 90-91 °C; ^{1}H NMR (400 MHz, $CDCl_3$) δ 0.92 (t, *J* = 7.2 Hz, 3H), 1.39 (d, *J* = 6.8 Hz, 6H), 1.40 (d, *J* = 6.8 Hz, 6H), 2.10-2.34 (m, 1H), 2.68 (dd, *J* = 4.0, 14.4 Hz, 1H), 3.13 (dd, *J* = 10.4, 14.0 Hz, 1H), 3.70-3.86 (m, 2H), 5.11 (hept, *J* = 6.8 Hz, 2H), 6.95 (s, 2H), 7.06-7.12 (m, 1H), 7.17-7.24 (m, 4H); ^{13}C NMR (100 MHz, $CDCl_3$) δ 14.2, 22.9, 23.3, 39.3, 49.2, 58.2, 115.5, 124.9, 127.7, 128.5, 145.0, 181.3; ^{11}B NMR (128.4 MHz, $CDCl_3$): δ -24.9 (t, *J* = 89.1 Hz, 1B); ESIHRMS: Found: *m/z* 365.2367. Calcd for $C_{20}H_{31}{}^{11}BN_2NaO_2$: $(M+Na)^+$ 365.2376.

Cyano(1,3-dimethyl-1*H*-imidazol-3-ium-2-yl)(1-ethoxy-1-oxo-3-phenylpropan-2-yl)hydr oborate (3a-D)

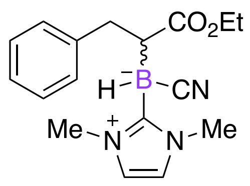

According to the general procedure, the reaction of **1a** (62.9 mg, 0.352 mmol), **2d** (41.2 mg, 0.305 mmol), AIBN (9.9 mg, 0.060 mmol) and PhSH (6.5 mg, 0.059 mmol) in CH_3CN (3 mL) was stirred at 80 °C for 12 h under nitrogen atmosphere after purification by flash column chromatography (silica gel; petroleum ether : ethyl acetate = 20 : 80) afforded product **3a-D** (63.5 mg) in 67% yield. Because there are two chiral centers (including a quaternized boron center) in the molecule, two diastereomers (dr = 1:1) are resulted. These two diastereomers could be separated by flash column chromatography, but the stereochemistry of each isomer was unable to be assigned.

Diasteroisomer **1**, colorless oil; ^{1}H NMR (400 MHz, $CDCl_3$) δ 0.85 (J = 7.2 Hz, 3H), 2.24-2.38 (m, BHCNC*H*, 1H), 2.99 (dd, J = 2.8, 14.4 Hz, 1H), 3.093 (dd, J = 11.2, 14.4 Hz, 1H), 3.56-3.72 (m, 2H), 3.85 (s, 6H), 6.90 (s, 2H), 7.08-7.14 (m, 1H), 7.16-7.25 (m, 1H); ^{13}C NMR (100 MHz, $CDCl_3$) δ 14.0, 36.6, 37.6, 58.9, 121.7, 125.4, 128.0, 128.4, 143.5, 178.6; ^{11}B NMR (128.4 MHz, $CDCl_3$): δ -26.6 (d, J = 95.9 Hz, 1B); ESIHRMS: Found: *m/z* 334.1718. Calcd for $C_{17}H_{22}{}^{11}BN_3NaO_2$: $(M+Na)^+$ 334.1703.

Diasteroisomer **2**, colorless oil; ^{1}H NMR (400 MHz, $CDCl_3$) δ 1.11 (J = 7.2 Hz, 3H), 2.14-2.25 (m, BHCNC*H*, 1H), 2.65 (dd, J = 5.2, 14.0 Hz, 1H), 3.14 (dd, J = 10.0, 14.4 Hz, 1H), 3.78 (s, 6H), 3.92-4.01 (m, 2H), 6.88 (s, 2H), 7.06-7.23 (m, 5H); ^{13}C NMR (100 MHz, $CDCl_3$) δ 14.3, 36.6, 37.1, 59.4, 121.8, 125.4, 127.9, 128.4, 142.9, 178.9; ^{11}B NMR (128.4 MHz, $CDCl_3$): δ -26.7 (d, J = 92.2 Hz, 1B); ESIHRMS: Found: *m/z* 334.1702. Calcd for $C_{17}H_{22}{}^{11}BN_3NaO_2$: $(M+Na)^+$ 334.1703.

(1,3-Dimethyl-1*H*-imidazol-3-ium-2-yl)(1-ethoxy-1-oxo-3-(o-tolyl)propan-2-yl)dihydroborate (3b)

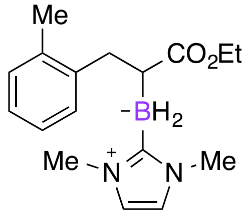

According to the general procedure, the reaction of ethyl (*E*)-3-(o-tolyl)acrylate (114.5 mg, 0.602 mmol), NHC-BH_3 (55.1 mg, 0.501 mmol), AIBN (16.4 mg, 0.100 mmol), *tert*-dodecanethiol (50.7 mg, 0.251 mmol), and MeCN (5 mL), under nitrogen at 80 °C for 12 h after flash column chromatography on silica gel (petroleum ether : ethyl acetate = 60 : 40) afforded 117.6 mg (78%) **3b** as a colorless liquid; ^{1}H NMR (400 MHz, $CDCl_3$) δ 0.98 (t, *J* = 7.2 Hz, 3H), 2.12-2.23 (m, BH_2CH, 1H), 2.32 (s, 3H), 2.72 (dd, *J* = 2.0, 14.8 Hz, 1H), 3.10 (dd, *J* = 10.4, 14.8 Hz, 1H), 3.75 (s, 6H), 3.77-3.86 (m, 2H), 6.82 (s, 2H), 6.98-7.08 (m, 3H), 7.20 (d, *J* = 7.2 Hz, 1H); ^{13}C NMR (100 MHz, $CDCl_3$) δ 14.2, 19.5, 35.92, 35.95, 58.5, 120.3, 125.0, 125.3, 128.5, 129.6, 135.9, 142.8, 181.6; ^{11}B NMR (128.4 MHz, $CDCl_3$): δ -25.0 (t, *J* = 88.7 Hz, 1B); ESIHRMS: Found: *m/z* 323.1909. Calcd for $C_{17}H_{25}{}^{11}BN_2NaO_2$: $(M+Na)^+$ 323.1907.

(3-(2-Aminophenyl)-1-ethoxy-1-oxopropan-2-yl)(1,3-dimethyl-1*H*-imidazol-3-ium-2-yl)dihydroborate (3c)

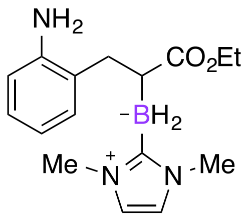

According to the general procedure, the reaction of ethyl (*E*)-3-(2-aminophenyl)acrylate (114.9 mg, 0.600 mmol), NHC-BH_3 (55.3 mg, 0.503 mmol), AIBN (16.4 mg, 0.100 mmol), *tert*-dodecanethiol (50.7 mg, 0.251 mmol), and MeCN (5 mL), under nitrogen at 80 °C for 12 h after flash column chromatography on silica gel (petroleum ether : ethyl acetate = 40 : 60) afforded 116.3 mg (77%) **3c** as a pale yellow liquid; ^{1}H NMR (400 MHz, $CDCl_3$) δ 0.94 (t, *J* = 7.2 Hz, 3H); 2.20 (m, BH_2CH, 1H), 2.54 (d, *J* = 14.8 Hz, 1H), 3.06 (dd, *J* = 10.4, 14.8 Hz, 1H), 3.73 (s, 6H), 3.75-3.88 (m, 2H), 6.61 (d, *J* = 8.0 Hz, 1H), 6.64 (dd, *J* = 7.6, 7.6 Hz, 1H), 6.81 (s, 2H), 6.93 (dd, *J* = 7.6, 8.0 Hz, 1H), 7.03-7.04 (d, *J* = 7.6 Hz, 1H); ^{13}C NMR (100 MHz, $CDCl_3$) δ 14.1, 33.9, 35.8, 58.5, 115.3, 118.1, 120.3, 126.0, 129.53, 129.67, 144.5, 182.3; ^{11}B NMR (128.4 MHz, $CDCl_3$): δ -24.8 (t, *J* = 90.4 Hz, 1B); ESIHRMS: Found: *m/z* 324.1859. Calcd for $C_{16}H_{24}{}^{11}BN_2NaO_2$: $(M+Na)^+$ 324.1859.

(1,3-Dimethyl-1*H*-imidazol-3-ium-2-yl)(1-ethoxy-3-(3-hydroxyphenyl)-1-oxopropan-2-yl)dihydroborate (3d)

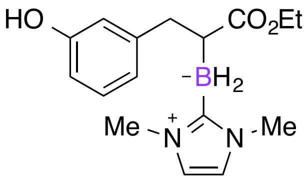

According to the general procedure, the reaction of ethyl (*E*)-3-(3-hydroxyphenyl)acrylate (117.0 mg, 0.609 mmol), NHC-BH_3 (55.2 mg, 0.502 mmol), AIBN (16.4 mg, 0.100 mmol), *tert*-dodecanethiol (50.7 mg, 0.251 mmol), and MeCN (5 mL), under nitrogen at 80 °C for 12 h after flash column chromatography on silica gel (petroleum ether : ethyl acetate = 40 : 60) afforded 116.3 mg (74%) **3d** as a pale yellow liquid; ^{1}H NMR (400 MHz, $CDCl_3$) δ 0.99 (t, *J* = 7.2 Hz, 3H), 2.22 (m, BH_2CH, 1H), 2.67 (dd, *J* = 3.6, 14.4 Hz, 1H), 3.05 (dd, *J* = 10.4, 14.4 Hz, 1H), 3.70 (s, 6H), 3.79-3.88 (m, 2H), 6.56 (d, *J* = 7.6 Hz, 1H), 6.68-6.75 (m, 2H), 6.76 (s, 2H), 7.03 (dd, *J* = 7.6, 7.6 Hz, 1H); ^{13}C NMR (100 MHz, $CDCl_3$) δ 14.2, 35.9, 38.9, 58.9, 112.2, 115.4, 120.3, 120.4, 128.8, 146.3, 155.9, 182.4; ^{11}B NMR (128.4 MHz, $CDCl_3$): δ -25.0 (t, *J* = 90.4 Hz, 1B). ESIHRMS: Found: *m/z* 325.1703. Calcd for $C_{16}H_{23}{}^{11}BN_3NaO_3$: $(M+Na)^+$ 325.1699.

(1,3-Dimethyl-1*H*-imidazol-3-ium-2-yl)(1-ethoxy-3-(4-fluorophenyl)-1-oxopropan-2-yl)dihydroborate (3e)

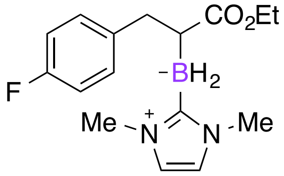

According to the general procedure, the reaction of ethyl (*E*)-3-(4-fluorophenyl)acrylate (116.7 mg, 0.601 mmol), NHC-BH_3 (55.4 mg, 0.504 mmol), AIBN (16.4 mg, 0.100 mmol), *tert*-dodecanethiol (50.7 mg, 0.251 mmol), and MeCN (5 mL), under nitrogen at 80 °C for 12 h after flash column chromatography on silica gel (petroleum ether : ethyl acetate = 60 : 40) afforded 116.3 mg (85%) **3e** as a white solid, mp: 81-82 °C; ^{1}H NMR (400 MHz, $CDCl_3$) δ 0.96 (t, *J* = 6.8 Hz, 3H), 2.13 (m, BH_2CH, 1H), 2.68 (dd, *J* = 3.2, 14.4 Hz, 1H), 3.06 (dd, *J* = 10.8, 14.4 Hz, 1H), 3.72 (s, 6H), 3.73-3.85 (m, 2H), 6.81 (s, 2H), 6.81-6.89 (m, 2H), 7.13-7.16 (m, 2H); ^{13}C NMR (100 MHz, $CDCl_3$) δ 14.2, 36.0, 38.2, 58.5, 114.3 (d, *J* = 20.6 Hz), 120.3, 129.8 (d, *J* = 7.6 Hz), 140.4 (d, *J* = 1.8 Hz), 160.8 (d, *J* = 240.1 Hz), 181.4; ^{11}B NMR (128.4 MHz, $CDCl_3$): δ -25.1 (t, *J* = 90.0 Hz, 1B); ^{19}F NMR (376 MHz, $CDCl_3$): δ -119.3 (1F, s); ESIHRMS: Found: *m/z* 327.1655. Calcd for $C_{16}H_{22}{}^{11}BFN_2NaO_2$: $(M+Na)^+$ 327.1656.

(3-(4-Cyanophenyl)-1-ethoxy-1-oxopropan-2-yl)(1,3-dimethyl-1*H*-imidazol-3-ium-2-yl)dihydroborate (3f)

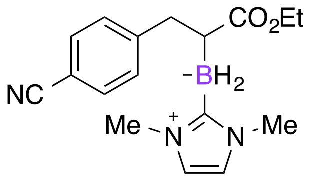

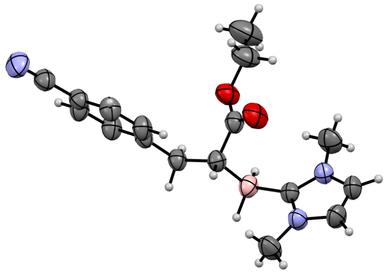

According to the general procedure, the reaction of ethyl (*E*)-3-(4-cyanophenyl)acrylate (120.1 mg, 0.600 mmol), NHC-BH_3 (55.0 mg, 0.500 mmol), AIBN (16.4 mg, 0.100 mmol), PhSH (10.8 mg, 0.098 mmol), and MeCN (5 mL), under nitrogen at 80 °C for 12 h after flash column chromatography on silica gel (petroleum ether : ethyl acetate = 50 : 50) afforded 80.7 mg (52%) **3f** as a white solid, mp: 119-120 °C; recrystallization from petroleum ether/ethyl acetate gave colorless crystals (CCDC: 1866488); ^{1}H NMR (400 MHz, $CDCl_3$) δ 0.92 (t, *J* = 7.2 Hz, 3H), 2.14 (m, BH_2CH, 1H), 2.76 (dd, *J* = 3.2, 14.4 Hz, 1H), 3.13 (dd, *J* = 10.8, 14.4 Hz, 1H), 3.67-3.84 (m, 8H), 6.83 (s, 2H), 7.30 (d, *J* = 7.6 Hz, 2H), 7.47 (d, *J* = 7.6 Hz, 2H); ^{13}C NMR (100 MHz, $CDCl_3$) δ 14.2, 35.9, 39.3, 58.6, 108.6, 119.5, 120.4, 129.3, 131.6, 150.7, 180.9; ^{11}B NMR (128.4 MHz, $CDCl_3$): δ -25.1 (t, *J* = 91.2 Hz, 1B); ESIHRMS: Found: *m/z* 334.1698. Calcd for $C_{17}H_{22}{}^{11}BN_3NaO_2$: $(M+Na)^+$ 334.1703.

Supplementary Table 1. Crystal data and structure refinement for **3f**.

Empirical formula	$C_{17}H_{22}BN_3O_2$
Formula weight	311.18
Temperature/K	291(2)
Crystal system	monoclinic
Space group	$P2_1/n$
a/Å	11.7108(3)
b/Å	5.83140(10)
c/Å	26.0680(6)
α/°	90
β/°	99.834(2)
γ/°	90
Volume/Å^3	1754.04(7)
Z	4
ρ_{calc}g/cm^3	1.178
μ/mm^{-1}	0.618

F(000)	664.0
Crystal size/mm^3	0.220 × 0.200 × 0.170
Radiation	CuKα (λ = 1.54184)
2Θ range for data collection/°	6.882 to 142.648
Index ranges	-10 ≤ h ≤ 14, -6 ≤ k ≤ 4, -31 ≤ l ≤ 28
Reflections collected	5867
Independent reflections	3274 [R_{int} = 0.0165, R_{sigma} = 0.0194]
Data/restraints/parameters	3274/0/219
Goodness-of-fit on F^2	1.038
Final R indexes [I>=2σ (I)]	R_1 = 0.0478, wR_2 = 0.1355
Final R indexes [all data]	R_1 = 0.0554, wR_2 = 0.1444
Largest diff. peak/hole / e Å^{-3}	0.18/-0.17

(1,3-Dimethyl-1*H*-imidazol-3-ium-2-yl)(1-ethoxy-3-(naphthalen-2-yl)-1-oxopropan-2-yl) dihydroborate (3g)

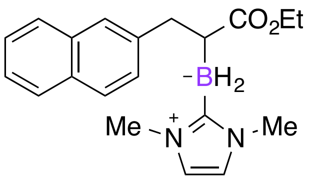

According to the general procedure, the reaction of ethyl (*E*)-3-(naphthalen-2-yl)acrylate (138.0 mg, 0.610 mmol), NHC-BH_3 (55.0 mg, 0.500 mmol), AIBN (16.4 mg, 0.100 mmol), PhSH (10.8 mg, 0.098 mmol), and MeCN (5 mL), under nitrogen at 80 ° C for 12 h after flash column chromatography on silica gel (petroleum ether : ethyl acetate = 60 : 40) afforded 121.7 mg (72%) **3g** as a white solid, mp: 76-77 °C; ^{1}H NMR (400 MHz, $CDCl_3$) δ 0.97 (t, *J* = 7.2 Hz, 3H), 2.24-2.35 (m, BH_2CH, 1H), 2.90 (dd, *J* = 3.6, 14.4 Hz, 1H), 3.29 (dd, *J* = 10.4, 14.4 Hz, 1H), 3.72 (s, 6H), 3.75-3.88 (m, 2H), 6.74 (s, 2H), 7.33-7.44 (m, 3H), 7.65 (s, 1H), 7.69 (d, *J* = 8.4 Hz, 1H), 7.73-7.78 (m, 2H); ^{13}C NMR (100 MHz, $CDCl_3$) δ 14.2, 35.9, 39.2, 58.5, 120.2, 124.4, 125.3, 126.1, 127.1, 127.35, 127.38, 127.9, 131.7, 133.5, 142.4, 181.5; ^{11}B NMR (128.4 MHz, $CDCl_3$): δ -24.9 (t, *J* = 89.4 Hz, 1B). ESIHRMS: Found: *m/z* 359.1904. Calcd for $C_{20}H_{25}{}^{11}BN_2NaO_2$: $(M+Na)^+$ 359.1907.

(1,3-Dimethyl-1*H*-imidazol-3-ium-2-yl)(1-oxo-3-phenyl-1-(prop-2-yn-1-yloxy)propan-2-yl)dihydroborate (3h)

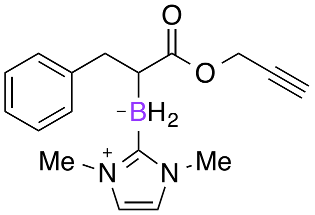

According to the general procedure, the reaction of prop-2-yn-1-yl cinnamate (84.1 mg, 0.452 mmol), NHC-BH_3 (33.3 mg, 0.303 mmol), AIBN (10.3 mg, 0.063 mmol), *tert*-dodecanethiol (31.0 mg, 0.153 mmol), and MeCN (3 mL), under nitrogen at 80 °C for 12 h after flash column chromatography on silica gel (petroleum ether : ethyl acetate = 70 : 30) afforded 74.1 mg (83%) **3h** as a colorless liquid; ^{1}H NMR (400 MHz, $CDCl_3$) δ 2.18-2.27 (m, BH_2*CH*, 1H), 2.29 (t, *J* = 2.0 Hz, 1H), 2.74 (dd, *J* = 3.6, 14.0 Hz, 1H), 3.13 (dd, *J* = 10.0, 14.0 Hz, 1H), 3.73 (s, 6H), 4.33 (dd, *J* = 2.0, 16.0 Hz, 1H), 4.44 (dd, *J* = 2.0, 16.0 Hz, 1H), 6.80 (s, 2H), 7.05-7.13 (m, 1H), 7.15-7.20 (m, 4H); ^{13}C NMR (100 MHz, $CDCl_3$) δ 36.0, 39.0, 50.2, 73.3, 79.0, 120.4, 125.0, 127.8, 128.4, 144.4, 180.4; ^{11}B NMR (128.4 MHz, $CDCl_3$): δ -25.0 (t, *J* = 90.4 Hz, 1B). ESIHRMS: Found: *m/z* 319.1585. Calcd for $C_{17}H_{21}{}^{11}BN_2NaO_2$: $(M+Na)^+$ 319.1588.

(1-(But-3-yn-1-yloxy)-1-oxo-3-phenylpropan-2-yl)(1,3-dimethyl-1*H*-imidazol-3-ium-2-yl) dihydroborate (3i)

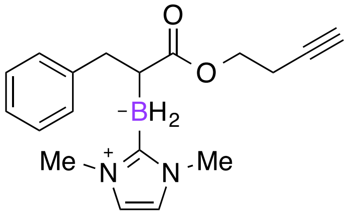

According to the general procedure, the reaction of but-3-yn-1-yl cinnamate (72.4 mg, 0.362 mmol), NHC-BH_3 (33.4 mg, 0.304 mmol), AIBN (9.5 mg, 0.058 mmol), *tert*-dodecanethiol (31.0 mg, 0.153 mmol), and MeCN (3 mL), under nitrogen at 80 °C for 12 h after flash column chromatography on silica gel (petroleum ether : ethyl acetate = 70 : 30) afforded 83.7 mg (89%) **3i** as a colorless liquid; ^{1}H NMR (400 MHz, $CDCl_3$) δ 1.90 (t, *J* = 2.4 Hz, 1H), 2.17-2.27 (m, 3H), 2.72 (dd, *J* = 4.4, 14.4 Hz, 1H), 3.11 (dd, *J* = 10.0, 14.4 Hz, 1H), 3.74 (s, 6H), 3.78-3.87 (m, 1H), 3.88-3.97 (m, 1H), 6.81 (s, 2H), 7.05-7.13 (m, 1H), 7.15-7.23 (m, 4H); ^{13}C NMR (100 MHz, $CDCl_3$) δ 18.9, 36.0, 39.0, 60.6, 69.3, 80.7, 120.4, 125.0, 127.8, 128.4, 144.5, 181.2; ^{11}B NMR (128.4 MHz, $CDCl_3$): δ -25.0 (t, *J* = 89.9 Hz, 1B); ESIHRMS: Found: *m/z* 333.1756. Calcd for $C_{18}H_{23}{}^{11}BN_2NaO_2$: $(M+Na)^+$ 333.1750.

(*E*)-(1-(But-2-en-1-yloxy)-3-(4-methoxyphenyl)-1-oxopropan-2-yl)(1,3-dimethyl-1*H*-imidazol-3-ium-2-yl)dihydroborate (3j)

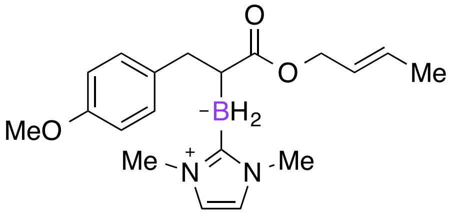

According to the general procedure, the reaction of (*E*)-but-2-en-1-yl (*E*)-3-(4-methoxyphenyl)acrylate (121.5 mg, 0.601 mmol), NHC-BH_3 (55.0 mg, 0.500 mmol), AIBN (16.4mg, 0.100 mmol), *tert*-dodecanethiol (50.7 mg, 0.251 mmol), and MeCN (5 mL), under nitrogen at 80 °C for 12 h after flash column chromatography on silica gel (petroleum ether : ethyl acetate = 60 : 40) afforded 145.1 mg (85%) **3j** as a colorless liquid; ^{1}H NMR (400 MHz, $CDCl_3$) δ 1.60 (d, J = 6.4 Hz), 2.09-2.20 (m, BH_2CH, 1H), 2.64 (dd, J = 3.6, 14.0 Hz, 1H), 3.03 (dd, J = 10.8, 14.0 Hz, 1H), 3.68 (s, 6H), 3.71 (s, 3H), 4.15-4.27 (m, 2H), 5.33 (td, J = 6.0, 15.2 Hz, 1H), 5.50 (qd, J = 6.4, 15.2 Hz, 1H), 6.72 (d, J = 8.0 Hz, 2H), 6.77 (s, 2H), 7.10 (d, J = 8.0 Hz, 2H); ^{13}C NMR (100 MHz, $CDCl_3$) δ 17.7, 36.0, 38.2, 55.1, 63.4, 113.2, 120.2, 126.2, 129.1, 129.3, 136.9, 157.1, 181.3; ^{11}B NMR (128.4 MHz, $CDCl_3$): δ -25.1 (t, J = 90.3 Hz, 1B); ESIHRMS: Found: *m/z* 365.2010. Calcd for $C_{19}H_{27}{}^{11}BN_2NaO_3$: $(M+Na)^+$ 365.2012.

(1,3-Dimethyl-1*H*-imidazol-3-ium-2-yl)(1-ethoxy-3-(1-methyl-1*H*-indol-5-yl)-1-oxopropan-2-yl)dihydroborate (3k)

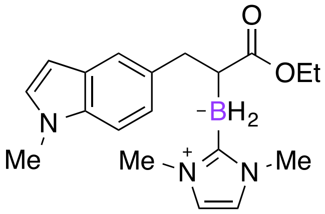

According to the general procedure, the reaction of ethyl (*E*)-3-(1-methyl-1*H*-indol-5-yl)acrylate (138.0 mg, 0.602 mmol), NHC-BH_3 (55.3 mg, 0.503 mmol), AIBN (16.4mg, 0.100mmol), *tert*-dodecanethiol (50.7 mg, 0.251 mmol), and MeCN (5 mL), under nitrogen at 80 °C for 12 h after flash column chromatography on silica gel (petroleum ether : ethyl acetate = 60 : 40) afforded 100.5 mg (59%) **3k** as a colorless liquid; ^{1}H NMR (400 MHz, $CDCl_3$) δ 0.98 (t, J = 7.2 Hz, 3H), 2.21-2.30 (m, BH_2CH, 1H), 2.81 (dd, J = 3.2, 14.0 Hz, 1H), 3.22 (dd, J = 10.4, 14.0 Hz, 1H), 3.73 (s, 3H), 3.74 (s, 6H), 3.77-3.84 (m, 2H), 6.36 (d, J = 2.4 Hz, 1H), 6.78 (s, 2H), 6.96 (d, J = 2.4 Hz, 1H), 7.12 (d, J = 8.4 Hz,

1H), 7.16 (d, J = 8.4 Hz, 1H), 7.44 (s, 1H); ^{13}C NMR (100 MHz, $CDCl_3$) δ 14.3, 32.7, 36.0, 39.1, 58.4, 100.4, 108.3, 119.9, 120.2, 122.9, 128.2, 128.4, 135.1, 135.7, 181.8; ^{11}B NMR (128.4 MHz, $CDCl_3$): δ -24.9 (t, J = 89.8 Hz, 1B); ESIHRMS: Found: m/z 362.2017. Calcd for $C_{19}H_{26}{}^{11}BN_3NaO_2$: $(M+Na)^+$ 362.2016.

(1,3-Dimethyl-1*H*-imidazol-3-ium-2-yl)(1-ethoxy-1-oxo-3-(quinolin-8-yl)propan-2-yl)dihydroborate (3l)

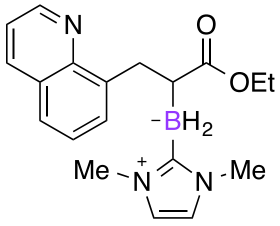

According to the general procedure, the reaction of ethyl (*E*)-3-(quinolin-8-yl)acrylate (136.5 mg, 0.600 mmol), NHC-BH_3 (55.2 mg, 0.502 mmol), AIBN (16.4mg, 0.100mmol), PhSH (10.8 mg, 0.098 mmol), and MeCN (5 mL), under nitrogen at 80 °C for 12 h after flash column chromatography on silica gel (petroleum ether : ethyl acetate = 50 : 50) afforded 139.3 mg (82%) **3l** as a white solid, mp: 77-78 °C; ^{1}H NMR (400 MHz, $CDCl_3$) δ 0.97 (t, J = 7.2 Hz, 3H), 2.52 (m, BH_2CH, 1H), 3.53 (d, J = 6.8 Hz, 2H), 3.75 (s, 6H), 3.83 (q, J = 7.2 Hz, 2H), 6.78 (s, 2H), 7.29 (dd, J = 4.0, 8.0 Hz, 1H), 7.38 (dd, J = 7.6, 7.6 Hz, 1H), 7.55 (d, J = 8.0 Hz, 1H), 7.65 (d, J = 7.0 Hz, 1H), 8.04 (d, J = 8.0 Hz, 1H), 8.79-8.87 (m, 1H); ^{13}C NMR (100 MHz, $CDCl_3$) δ 14.2, 34.5, 35.8, 58.2, 120.1 (overlapped), 125.0, 126.0, 128.0, 128.7, 135.9, 142.9, 146.9, 148.5, 181.8; ^{11}B NMR (128.4 MHz, $CDCl_3$): δ -24.8 (t, J = 88.5 Hz, 1B); ESIHRMS: Found: m/z 360.1856. Calcd for $C_{19}H_{24}{}^{11}BN_3NaO_2$: $(M+Na)^+$ 360.1859.

(1,3-Dimethyl-1*H*-imidazol-3-ium-2-yl)(1-ethoxy-3-(furan-2-yl)-1-oxopropan-2-yl)dihydroborate (3m)

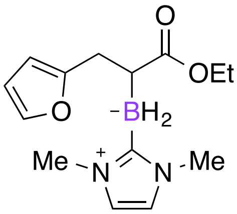

According to the general procedure, the reaction of ethyl (*E*)-3-(furan-2-yl)acrylate (101.6 mg, 0.610 mmol), NHC-BH_3 (55.0 mg, 0.500 mmol), AIBN (16.4mg, 0.100 mmol), PhSH (10.8 mg, 0.098 mmol), and MeCN (5 mL), under nitrogen at 80 °C for 12 h after flash column chromatography on silica gel (petroleum ether : ethyl acetate = 50 : 50) afforded

130.8 mg (95%) **3m** as a colorless liquid; ^{1}H NMR (400 MHz, $CDCl_3$) δ 1.04 (t, J = 7.2 Hz, 3H), 2.15-2.28 (m, BH_2CH, 1H), 2.68 (dd, J = 3.6, 16.0 Hz, 1H), 3.11 (dd, J = 10.8, 16.0 Hz, 1H), 3.75 (s, 6H), 3.82-3.95 (m, 2H), 5.90-5.93 (m, 1H), 6.17-6.23 (m, 1H), 6.81 (s, 2H), 7.20-7.24 (m, 1H); ^{13}C NMR (100 MHz, $CDCl_3$) δ 14.3, 31.3, 36.0, 58.6, 103.9, 109.9, 120.3, 140.0, 158.5, 181.4; ^{11}B NMR (128.4 MHz, $CDCl_3$): δ -25.2 (t, J = 90.9 Hz, 1B); ESIHRMS: Found: *m/z* 299.1543. Calcd for $C_{14}H_{21}{}^{11}BN_2NaO_3$: $(M+Na)^+$ 299.1543.

(1,3-Dimethyl-1*H*-imidazol-3-ium-2-yl)(2-oxochroman-3-yl)dihydroborate (3n)

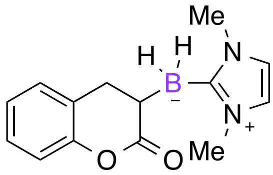

According to the general procedure, the reaction of 2*H*-chromen-2-one (87.7 mg, 0.600 mmol), NHC-BH_3 (55.1 mg, 0.501 mmol), AIBN (16.4 mg, 0.100 mmol), *tert*-dodecanethiol (50.7 mg, 0.251 mmol), and MeCN (5 mL), under nitrogen at 80 °C for 12 h after flash column chromatography on silica gel (petroleum ether : ethyl acetate = 30 : 70) afforded 92.9 mg (72%) **3n** as a white solid, mp: 123-124 °C; ^{1}H NMR (400 MHz, $CDCl_3$) δ 2.44 (m, BH_2CH, 1H), 2.69-2.78 (m, 1H), 3.19-3.32 (m, 1H), 3.68 (s, 6H), 6.78 (s, 2H), 6.90 (d, J = 7.6 Hz, 1H), 7.00 (dd, J = 7.2, 7.2 Hz, 1H), 7.09-7.18 (m, 2H); ^{13}C NMR (100 MHz, $CDCl_3$) δ 30.6, 35.9, 115.4, 120.6, 123.3, 124.0, 126.8, 128.4, 152.3, 178.2; ^{11}B NMR (128.4 MHz, $CDCl_3$): δ -26.1 (t, J = 91.2 Hz, 1B); ESIHRMS: Found: *m/z* 279.1282. Calcd for $C_{14}H_{27}{}^{11}BN_2NaO_2$: $(M+Na)^+$ 279.1281.

(1,3-Dimethyl-1*H*-imidazol-3-ium-2-yl)(1-ethoxy-3-(4-methoxyphenyl)-1-oxobutan-2-yl) dihydroborate (3o)

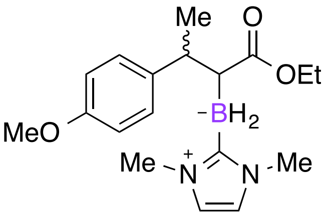

According to the general procedure, the reaction of ethyl 3-(4-methoxyphenyl)but-2-enoate (132.2 mg, 0.600 mmol), NHC-BH_3 (55.0 mg, 0.500 mmol), AIBN (16.4 mg, 0.100 mmol), PhSH (10.8 mg, 0.098 mmol), and MeCN (5 mL), under nitrogen at 80 °C for 12 h after flash column chromatography on silica gel (petroleum ether : ethyl acetate = 70 : 30) afforded 158.2 mg (95%) **3o** as a mixture of diastereoisomers (dr = 5.2:1). The stereochemistry of the

major isomer was not determined. ^{1}H NMR (400 MHz, $CDCl_3$) δ 0.77 (t, J = 6.8 Hz, 3Hx5.2), 1.13 (t, J = 7.2 Hz, 3Hx1), 1.19 (d, J = 6.8 Hz, 3Hx1), 1.30 (t, J = 7.2 Hz, 3Hx5.2), 2.03-2.17 (m, 1H+1Hx5.2), 2.98-3.13 (m, 1H+1Hx5.2), 3.44-3.62 (m, 6Hx1+2Hx5.2), 3.72-3.79 (m, 3Hx1+9Hx5.2), 3.89-4.02 (m, 2Hx1), 6.65 (s, 2Hx1), 6.70-6.78 (m, 2Hx1+2Hx5.2), 6.80 (s, 2Hx5.2), 7.06 (d, J = 8.4 Hz, 2Hx1), 7.16 (d, J = 8.0 Hz, 2Hx5.2); ^{13}C NMR (100 MHz, $CDCl_3$) δ 14.1, 14.4, 22.2, 24.2, 35.7, 36.0, 42.3, 42.6, 55.1, 55.2, 58.1, 58.6, 113.07, 113.1, 120.0, 120.3, 127.8, 128.2, 140.5, 141.2, 142.9, 157.0, 157.03, 181.0, 181.7; ^{11}B NMR (128.4 MHz, $CDCl_3$): δ -25.9 (t, J = 88.6 Hz, 1B); ESIHRMS: Found: *m/z* 353.2009. Calcd for $C_{18}H_{27}{}^{11}BN_2NaO_3$: $(M+Na)^+$ 353.2012.

(1,3-Dimethyl-1*H*-imidazol-3-ium-2-yl)(1-ethoxy-2-methyl-1-oxo-3-phenylpropan-2-yl)dihydroborate (3p-α)

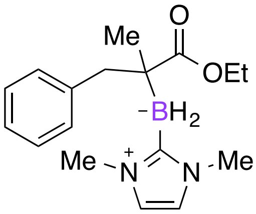

According to the general procedure, the reaction of ethyl (*E*)-2-methyl-3-phenylacrylate (454.7 mg, 2.39 mmol), NHC-BH_3 (220.0 mg, 2.00 mmol), AIBN (66.5 mg, 0.400 mmol), PhSH (43.1 mg, 0.390 mmol), and MeCN (10 mL), under nitrogen at 80 °C for 12 h after flash column chromatography on silica gel (petroleum ether : ethyl acetate = 70 : 30) afforded 129.5 mg (22%) **3p-α** as a colorless liquid; ^{1}H NMR (400 MHz, $CDCl_3$) δ 1.03 (s, 3H), 1.15 (t, J = 7.2 Hz, 3H), 2.60 (d, J = 13.6 Hz, 1H), 3.47 (d, J = 13.6 Hz, 1H), 3.78 (s, 6H), 3.89-4.01 (m, 2H), 6.86 (s, 2H), 7.08-7.14 (m, 1H), 7.16-7.21 (m, 4H); ^{13}C NMR (100 MHz, $CDCl_3$) δ 14.4, 22.8, 36.5, 46.1, 58.8, 120.7, 125.1, 127.4, 130.1, 142.3, 182.7; ^{11}B NMR (128.4 MHz, $CDCl_3$): δ -20.8 (t, J = 90.0 Hz, 1B); ESIHRMS: Found: *m/z* 323.1908. Calcd for $C_{17}H_{25}{}^{11}BN_2NaO_2$: $(M+Na)^+$ 323.1907.

In addition, **3p-*β*** was isolated in 53% yield (317.4 mg).

(1,3-Dimethyl-1*H*-imidazol-3-ium-2-yl)(3-ethoxy-2-methyl-3-oxo-1-phenylpropyl)dihydroborate (3p-*β*)

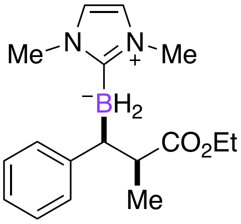

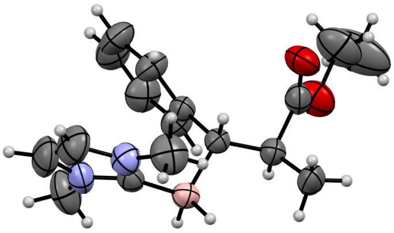

Recrystallization from petroleum ether/ethyl acetate gave colorless crystals (CCDC: 1866489), mp: 103-104 °C; ^{1}H NMR (400 MHz, $CDCl_3$) δ 0.85 (t, *J* = 7.2 Hz, 3H), 1.43 (d, *J* = 6.8 Hz, 3H), 1.94-2.06 (m, BH_2CH, 1H), 2.87-3.00 (m, 1H), 3.34 (s, 6H), 3.72-3.84 (m, 2H), 6.64 (s, 2H), 6.78 (d, *J* = 7.6 Hz, 2H), 6.83 (t, *J* = 7.2 Hz, 1H), 6.95 (dd, *J* = 7.2, 7.6 Hz, 2H); ^{13}C NMR (100 MHz, $CDCl_3$) δ 13.9, 18.1, 35.3, 45.9, 59.0, 119.9, 122.7, 126.9, 127.2, 152.2, 179.0; ^{11}B NMR (128.4 MHz, $CDCl_3$): δ -24.6 (1B, t, *J* = 86.9 Hz); ESIHRMS: Found: *m/z* 323.1909. Calcd for $C_{17}H_{25}{}^{11}BN_2NaO_2$: $(M+Na)^+$ 323.1907.

Supplementary Table 2. Crystal data and structure refinement for **3p-*β***.

Empirical formula	$C_{17}H_{25}BN_2O_2$
Formula weight	300.20
Temperature/K	291(2)
Crystal system	monoclinic
Space group	C2/c
a/Å	21.9318(4)
b/Å	9.2414(2)
c/Å	18.1299(3)
α/°	90
β/°	99.662(2)
γ/°	90
Volume/Å^3	3622.45(12)
Z	8
ρ_{calc}g/cm^3	1.101
μ/mm^{-1}	0.561
F(000)	1296.0
Crystal size/mm^3	0.240 × 0.220 × 0.190
Radiation	CuKα (λ = 1.54184)
2Θ range for data collection/°	8.178 to 142.628
Index ranges	-26 ≤ h ≤ 12, -11 ≤ k ≤ 9, -22 ≤ l ≤ 22
Reflections collected	6750
Independent reflections	3430 [R_{int} = 0.0155, R_{sigma} = 0.0170]
Data/restraints/parameters	3430/0/212
Goodness-of-fit on F^2	1.051
Final R indexes [I>=2σ (I)]	R_1 = 0.0640, wR_2 = 0.1907
Final R indexes [all data]	R_1 = 0.0687, wR_2 = 0.1976

Largest diff. peak/hole / e Å^{-3}	0.56/-0.31

(1,3-Dimethyl-1*H*-imidazol-3-ium-2-yl)(1-morpholino-1-oxo-3-phenylpropan-2-yl)dihydroborate (3q)

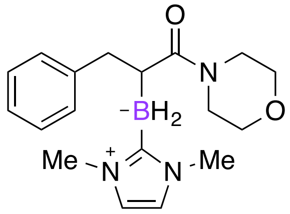

According to the general procedure, the reaction of (*E*)-1-morpholino-3-phenylprop-2-en-1-one (130.4 mg, 0.600 mmol), NHC-BH_3 (55.0 mg, 0.500 mmol), AIBN (16.4 mg, 0.100 mmol), *tert*-dodecanethiol (50.7 mg, 0.251 mmol), and MeCN (5 mL), under nitrogen at 80 °C for 12 h after flash column chromatography on silica gel (petroleum ether : ethyl acetate = 40 : 60) afforded 140.3 mg (86%) **3q** as a pale yellow liquid; ^{1}H NMR (400 MHz, $CDCl_3$) δ 2.48-2.57 (m, BH_2CH, 1H), 2.59 (dd, J = 3.6, 13.2 Hz, 1H), 3.01 (dd, J = 10.0, 13.2 Hz, 1H), 3.31-3.60 (m, 8H), 3.71 (s, 6H), 6.80 (s, 2H), 7.03-7.07 (m, 1H), 7.08-7.17 (m, 4H); ^{13}C NMR (100 MHz, $CDCl_3$) δ 36.2, 39.0, 41.6, 46.0, 66.9, 67.2, 120.6, 124.9, 127.7, 128.2, 144.9, 180.2; ^{11}B NMR (128.4 MHz, $CDCl_3$): δ -26.1 (t, J = 87.9 Hz, 1B); ESIHRMS: Found: m/z 350.2023. Calcd for $C_{18}H_{26}{}^{11}BN_3NaO_2$: $(M+Na)^+$ 350.2016.

(1,3-Dimethyl-1*H*-imidazol-3-ium-2-yl)(1-methyl-2-oxo-1,2,3,4-tetrahydroquinolin-3-yl) dihydroborate(3r)

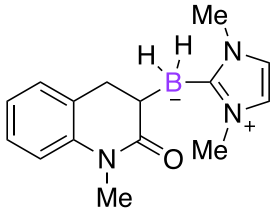

The procedure for the gram scale synthesis of 3r.

A solution of **1r** (2.545 g, 15.986 mmol), **2a** (1.924 g, 17.493 mmol), AIBN (537.7 mg, 3.275 mmol) and PhSH (0.32 mL, 3.198 mmol) in CH_3CN (50 mL) was stirred at 80 °C for 12 h under nitrogen atmosphere. After evaporation of solvent, the resulting crude material was purified by flash column chromatography (silica gel; petroleum ether : ethyl acetate = 60 : 40) to give product **3r** (3.402 g) in 79% yield. White solid, mp: 163-164 °C; ^{1}H NMR (400 MHz, $CDCl_3$) δ 2.17-2.31 (m, BH_2CH, 1H), 2.63 (d, J = 15.2 Hz, 1H), 3.16-3.27 (m, 4H), 3.62 (s,

6H), 6.74 (s, 2H), 6.83 (d, *J* = 8.0 Hz, 1H), 6.93 (td, *J* = 0.8, 7.2 Hz, 1H), 7.10-7.19 (m, 2H); ^{13}C NMR (100 MHz, $CDCl_3$) δ 29.1, 32.4, 35.8, 112.9, 120.2, 120.4, 121.7, 126.0, 127.9, 141.1, 180.1; ^{11}B NMR (128.4 MHz, $CDCl_3$): δ -26.8 (t, *J* = 88.7 Hz, 1B); ESIHRMS: Found: *m/z* 292.1588. Calcd for $C_{15}H_{20}{}^{11}BN_3NaO$: $(M+Na)^+$ 292.1597.

(1-(But-3-en-1-ylamino)-1-oxo-3-phenylpropan-2-yl)(1,3-dimethyl-1*H*-imidazol-3-ium-2-yl)dihydroborate (3s)

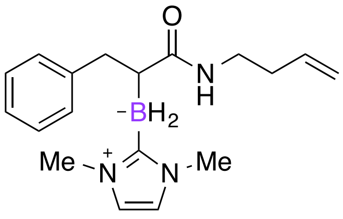

According to the general procedure, the reaction of *N*-(but-3-en-1-yl)cinnamamide (120.9 mg, 0.601 mmol), NHC-BH_3 (55.6 mg, 0.506 mmol), AIBN (16.5 mg, 0.100 mmol), PhSH (10.8 mg, 0.098 mmol), and MeCN (5 mL), under nitrogen at 80 °C for 12 h after flash column chromatography on silica gel (CH_2Cl_2 : MeOH : Et_3N = 97 : 3 : 1) afforded 138.8 mg (88%) **3s** as a white solid, mp: 85-86 °C; ^{1}H NMR (400 MHz, $CDCl_3$) δ 1.96-2.04 (m, BH_2CH, 1H), 2.04-2.11 (m, 2H), 2.72 (dd, *J* = 5.2, 14.4 Hz, 1H), 2.97 (dd, *J* = 9.6, 14.4 Hz, 1H), 3.08-3.22 (m, 2H), 3.72 (s, 6H), 4.92-4.96 (m, 1H), 4.97-4.99 (m, 1H), 5.27-5.31 (m br, 1H), 5.57-5.70 (m, 1H), 6.78 (s, 2H), 7.04-7.11 (m, 1H), 7.13-7.21 (m, 4H); ^{13}C NMR (100 MHz, $CDCl_3$) δ 34.0, 36.0, 38.1, 39.8, 116.5, 120.4, 125.0, 127.8, 128.4, 135.8, 144.2, 181.6; ^{11}B NMR (128.4 MHz, $CDCl_3$): δ -24.9 (t, *J* = 88.6 Hz, 1B); ESIHRMS: Found: *m/z* 310.2092. Calcd for $C_{18}H_{25}{}^{11}BN_3O$: $(M-H)^-$ 310.2091.

(1,3-Dimethyl-1*H*-imidazol-3-ium-2-yl)(1-((*S*)-4-isopropyl-2-oxooxazolidin-3-yl)-1-oxo-3-phenylpropan-2-yl)dihydroborate (3t)

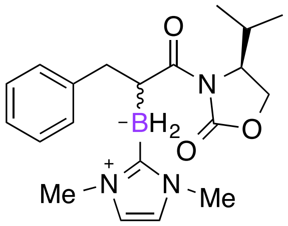

According to the general procedure, the reaction of (*S*)-3-cinnamoyl-4-isopropyloxazolidin-2-one (99.8 mg, 0.385 mmol), NHC-BH_3 (32.9 mg, 0.300 mmol), AIBN (9.8 mg, 0.060 mmol), PhSH (6.5 mg, 0.059 mmol), and MeCN (3 mL), under nitrogen at 80 °C for 12 h after flash column chromatography on silica gel (petroleum

ether : ethyl acetate = 50 : 50) afforded 106.6 mg (96%) **3t**. Two diastereomers (dr = 1.7:1) could be separated, but the stereochemistry of each isomer has not been assigned.

Diastereomer **1**, white solid, mp: 129-130 °C; ^{1}H NMR (400 MHz, $CDCl_3$) δ 0.88 (d, *J* = 7.2 Hz, 3H), 0.91 (d, *J* = 6.8 Hz, 3H), 2.28-2.40 (m, 2H), 3.20 (dd, *J* = 9.6, 14.4 Hz, 1H), 3.84 (s, 6H), 3.89-3.99 (m, 1H), 4.04-4.13 (m, 2H), 4.41-4.48 (m, 1H), 6.76 (m, 2H), 6.98-7.05 (m, 1H), 7.06-7.16 (m, 4H); ^{13}C NMR (100 MHz, $CDCl_3$) δ 14.8, 18.0, 28.6, 36.3, 38.0, 58.1, 62.5, 120.6, 125.0, 127.8, 128.2, 143.8, 154.4, 181.1; ^{11}B NMR (128.4 MHz, $CDCl_3$): δ -24.2 (t, *J* = 88.5 Hz, 1B); ESIHRMS: Found: *m/z* 392.2121. Calcd for $C_{20}H_{28}{}^{11}BN_3NaO_3$: $(M+Na)^+$ 392.2121.

Diastereomer **2**, colorless liquid; ^{1}H NMR (400 MHz, $CDCl_3$) δ 0.57 (d, *J* = 6.8 Hz, 3H), 0.76 (d, *J* = 7.2 Hz, 3H), 2.10-2.22 (m, 1H), 2.50 (dd, *J* = 4.4, 14.4 Hz, 1H), 3.22 (dd, *J* = 10.0, 14.0 Hz, 1H), 3.81 (s, 6H), 3.82-3.90 (m, 1H), 4.02-4.06 (m, 2H), 4.29-4.36 (m, 1H), 6.79 (s, 2H), 6.99-7.05 (m, 1H), 7.11-7.16 (m, 4H); ^{13}C NMR (100 MHz, $CDCl_3$) δ 14.3, 18.0, 28.7, 36.3, 38.5, 59.1, 62.5, 120.5, 124.9, 127.8, 128.3, 143.9, 154.3, 181.3; ^{11}B NMR (128.4 MHz, $CDCl_3$): δ -24.5 (t, *J* = 91.2 Hz, 1B); ESIHRMS: Found: *m/z* 392.2123. Calcd for $C_{20}H_{28}{}^{11}BN_3NaO_3$: $(M+Na)^+$ 392.2121.

In order to promote the diastereoselectivity, a range of Lewis acids, such as $La(OTf)_3$, $Zn(OTf)_2$, and $MgBr_2$, was added to the reaction mixture. However, the desired hydroboration product **3t** was not detected, and hydrogenation of **1t** occurred instead.

Supplementary Table 3.

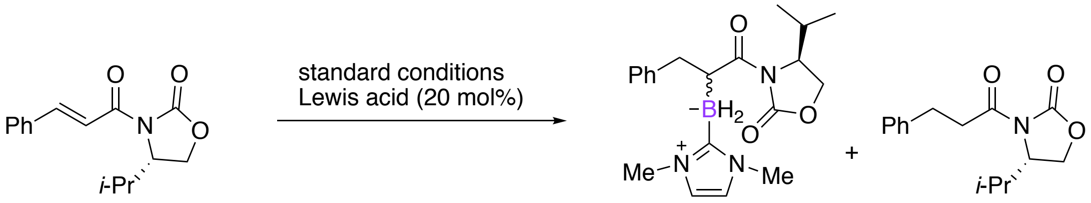

3t **3t'**

Lewis acid	3t	3t'
$La(OTf)_3$	0	62%
$Zn(OTf)_3$	0	66%
$MgBr_2$	0	87%

(1,3-Dimethyl-1*H*-imidazol-3-ium-2-yl)(1-((4-methyl-*N*-(prop-2-yn-1-yl)phenyl)sulfonamido)-1-oxo-3-phenylpropan-2-yl)dihydroborate (3u)

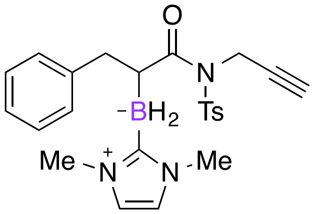

According to the general procedure, the reaction of *N*-(prop-2-yn-1-yl)-*N*-tosylcinnamamide (101.4 mg, 0.299 mmol), NHC-BH_3 (22.1 mg, 0.201 mmol), AIBN (6.7 mg, 0.041 mmol), *tert*-dodecanethiol (20.6 mg, 0.102 mmol), and MeCN (2 mL), under nitrogen at 80 °C for 12 h after flash column chromatography on silica gel (petroleum ether : ethyl acetate = 50 : 50) afforded 26.0 mg (29%) **3u** as a white solid, mp: 97-98 °C; ^{1}H NMR (400 MHz, $CDCl_3$) δ 2.10 (t, *J* = 2.4 Hz, 1H), 2.38 (s, 3H), 2.62 (dd, *J* = 3.2, 13.2 Hz, 1H), 2.72 (dd, *J* = 10.8, 13.2 Hz, 1H), 2.77-2.85 (m, 1H), 3.74 (s, 6H), 4.71 (dd, *J* = 2.4, 18.8 Hz, 1H), 4.77 (dd, *J* = 2.4, 18.8 Hz, 1H), 6.78-6.82 (m, 2H), 6.83 (s, 2H), 6.87-6.93 (m, 2H), 6.93-6.99 (m, 1H), 7.06-7.02 (m, 2H), 7.65 (d, *J* = 8.4 Hz, 2H); ^{13}C NMR (100 MHz, $CDCl_3$) δ 21.5, 35.0, 36.5, 38.6, 72.1, 79.4, 120.9, 124.6, 127.4, 128.0, 128.4, 128.8, 136.8, 1431, 143.5, 180.5; ^{11}B NMR (128.4 MHz, $CDCl_3$): δ -25.8 (t, *J* = 89.5 Hz, 1B); ESIHRMS: Found: *m/z* 472.1842. Calcd for $C_{24}H_{28}{}^{11}BN_3NaO_3S$: $(M+Na)^+$ 472.1840.

In addition, cyclic product **3u'** was isolated in 42% yield (37.7 mg). Recrystallization from petroleum ether/ethyl acetate gave colorless crystals.

1,3-Dimethyl-1*H*-imidazol-3-ium-2-yl)((3*R,4*S**)-5-methylene-2-oxo-4-phenyl-1-tosylpiperidin-3-yl)dihydroborate (3u')**

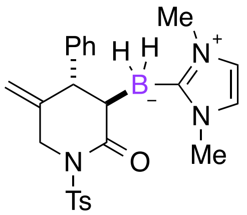

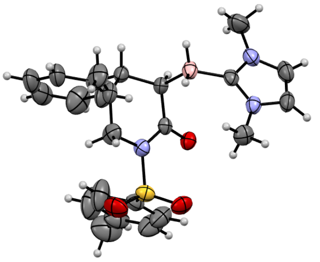

Colorless crystal (CCDC: 1866490), mp: 217-218 °C; ^{1}H NMR (400 MHz, $CDCl_3$) δ 2.12-2.20 (m, BH_2CH, 1H), 2.40 (s, 3H), 3.66 (s, 6H), 3.77 (s, 1H), 4.34 (d, *J* = 14.0 Hz, 1H), 4.65 (d, *J* = 14.0 Hz, 1H), 5.03 (s, 1H), 5.14 (s, 1H), 6.79 (s, 2H), 6.92-6.99 (m, 2H), 7.06-7.14 (m, 3H), 7.19 (d, *J* = 8.0 Hz, 2H), 7.67 (d, *J* = 8.0 Hz, 2H); ^{13}C NMR (100 MHz, $CDCl_3$) δ 21.5, 36.0, 50.1, 51.8, 112.1, 120.5, 126.0, 127.2, 128.1, 128.2, 129.0, 137.0, 142.2,

143.5, 145.0, 178.8; ^{11}B NMR (128.4 MHz, $CDCl_3$): δ -25.2 (t, *J* = 89.5 Hz, 1B); ESIHRMS: Found: *m/z* 472.1840. Calcd for $C_{24}H_{28}{}^{11}BN_3NaO_3S$: $(M+Na)^+$ 472.1842.

Supplementary Table 4. Crystal data and structure refinement for **3u'**.

Empirical formula	$C_{24}H_{28}BN_3O_3S$
Formula weight	449.36
Temperature/K	291(2)
Crystal system	monoclinic
Space group	$P2_1/n$
a/Å	12.96560(10)
b/Å	11.13370(10)
c/Å	17.1515(2)
α/°	90
β/°	105.1280(10)
γ/°	90
Volume/Å^3	2390.10(4)
Z	4
ϱ_{calc}g/cm^3	1.249
μ/mm^{-1}	1.442
F(000)	952.0
Crystal size/mm^3	0.220 × 0.200 × 0.120
Radiation	CuKα (λ = 1.54184)
2Θ range for data collection/°	7.662 to 142.65
Index ranges	$-15 \le h \le 14, -12 \le k \le 13, -18 \le l \le 20$
Reflections collected	8920
Independent reflections	4519 [R_{int} = 0.0196, R_{sigma} = 0.0261]
Data/restraints/parameters	4519/0/292
Goodness-of-fit on F^2	1.037
Final R indexes [I>=2σ (I)]	R_1 = 0.0428, wR_2 = 0.1182
Final R indexes [all data]	R_1 = 0.0476, wR_2 = 0.1234
Largest diff. peak/hole / e Å^{-3}	0.20/-0.36

(1,3-Dimethyl-1*H*-imidazol-3-ium-2-yl)(3-(4-methoxyphenyl)-1-((4-methyl-*N*-(3-methylbut-2-en-1-yl)phenyl)sulfonamido)-1-oxopropan-2-yl)dihydroborate (3v)

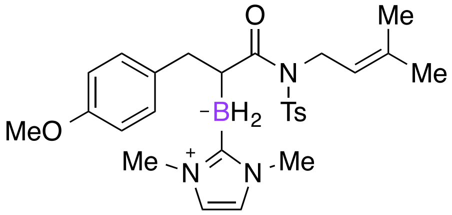

According to the general procedure, the reaction of (*E*)-3-(4-methoxyphenyl)-*N*-(3-methylbut-2-en-1-yl)-*N*-tosylacrylamide (143.2 mg, 0.360 mmol), NHC-BH_3 (33.3 mg, 0.303 mmol), AIBN (9.9 mg, 0.060 mmol), *tert*-dodecanethiol (31.0 mg, 0.153 mmol), and MeCN (3 mL), under nitrogen at 80 °C for 12 h after flash column chromatography on silica gel (petroleum ether : ethyl acetate = 70 : 30) afforded 24.4 mg (15%) **3v** as a white solid, mp: 83-84 °C; ^{1}H NMR (400 MHz, $CDCl_3$) δ 1.58 (s, 3H), 1.68 (s, 3H), 2.40 (s, 3H), 2.50-2.63 (m, 2H), 2.68-2.80 (m, 1H), 3.73 (s, 3H), 3.74 (s, 6H), 4.27-4.37 (m, 1H), 4.43-4.59 (m, 2H), 6.47 (d, *J* = 8.0 Hz, 2H), 6.69 (d, *J* = 8.0 Hz, 2H), 6.83 (s, 2H), 7.12 (d, *J* = 8.0 Hz, 2H), 7.53 (d, *J* = 8.0 Hz, 2H); ^{13}C NMR (100 MHz, $CDCl_3$) δ 17.9, 21.5, 25.4, 36.5, 38.4, 43.9, 55.0, 113.0, 120.8, 121.5, 128.1, 128.6, 129.2, 134.7, 136.6, 137.8, 142.8, 157.0, 181.4; ^{11}B NMR (128.4 MHz, $CDCl_3$): δ -25.8 (t, *J* = 89.2 Hz, 1B); ESIHRMS: Found: *m/z* 532.2422. Calcd for $C_{27}H_{36}{}^{11}BN_3NaO_4S$: $(M+Na)^+$ 532.2417.

In addition, cyclic product **3v’** was isolated in 34% yield (53.1 mg) as a mixture of diastereomers (dr = 2.8:1). After recrystallization from ethyl acetate/petroleum ether, a white solid (dr = 5.8:1) was obtained. NMR data described below were recorded for this solid.

(1,3-Dimethyl-1*H*-imidazol-3-ium-2-yl)((3*S,4*S**)-5-isopropyl-4-(4-methoxyphenyl)-2-oxo-1-tosylpiperidin-3-yl)dihydroborate (3v’)**

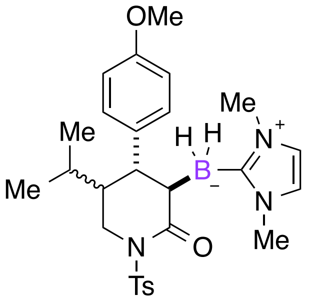

White solid, dr = 5.8:1; ^{1}H NMR (400 MHz, $CDCl_3$) δ 0.85 (d, *J* = 6.8 Hz, 3Hx5.8), 0.90 (d, *J* = 6.0 Hz, 3Hx1), 0.93 (d, *J* = 6.8 Hz, 3Hx5.8), 1.14-1.18 (d, *J* = 6.0 Hz, 3Hx1), 1.50-1.61 (m, 2Hx1+2Hx5.8), 1.71-1.76 (m, 1Hx5.8), 1.87-1.92 (m, 1Hx1), 2.41 (s, 3Hx5.8), 2.44 (s, 3Hx1), 2.80 (dd, *J* = 4.4, 11.2 Hz, 1Hx5.8), 3.04 (dd, *J* = 12.0, 12.0 Hz, 1Hx1), 3.27-3.30 (m, 1Hx1), 3.43 (s, 6Hx5.8), 3.65 (s, 6Hx1), 3.70 (dd, *J* = 11.6, 12.4 Hz, 1Hx5.8), 3.74 (s, 3Hx1), 3.76 (s, 3Hx5.8), 4.26-4.31 (m, 1Hx5.8+1Hx1), 6.57 (d, *J* = 8.8 Hz, 2Hx1), 6.62 (d, *J* = 8.8

Hz, 2Hx1), 6.70 (s, 2Hx5.8), 6.74 (d, *J* = 8.8 Hz, 2Hx5.8), 6.78 (s, 2Hx1), 6.99 (d, *J* = 8.8 Hz, 2Hx5.8), 7.25-7.32 (m, 2Hx5.8+2Hx1), 7.85-7.90 (m, 2Hx5.8+2Hx1); ^{13}C NMR (100 MHz, $CDCl_3$) δ 15.9, 20.5, 21.5, 21.7, 27.3, 27.7, 35.6, 39.8, 46.1, 48.7, 49.6, 55.1, 113.1, 113.4, 120.2, 120.4, 128.0, 128.5, 128.9, 129.1, 129.3, 137.9, 139.3, 143.5, 157.5, 179.7; ^{11}B NMR (128.4 MHz, $CDCl_3$): δ -25.4 (t, *J* = 89.6 Hz, 1B). ESIHRMS: Found: *m/z* 494.2649. Calcd for $C_{27}H_{37}{}^{11}BN_3O_3S$: $(M-H)^-$ 494.2649.

(*Z*)-(5-Butyl-1-methyl-2-oxo-4-phenyl-1,2,3,4-tetrahydrobenzo[*b*]azocin-3-yl)(1,3-dimethyl-1*H*-imidazol-3-ium-2-yl)dihydroborate (3w)

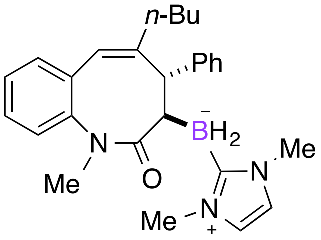

According to the general procedure, the reaction of *N*-(2-(hex-1-yn-1-yl)phenyl)-*N*-methylcinnamamide (150.2 mg, 0.473 mmol), NHC-BH_3 (62.4 mg, 0.567 mmol), AIBN (15.6 mg, 0.095 mmol), isooctyl thioglycolate (19.4 mg, 0.095 mmol), and MeCN (5 mL), under nitrogen at 80 °C for 12 h after flash column chromatography on silica gel (petroleum ether : ethyl acetate = 30 : 70) afforded 141.5 mg (70%) **3w** as a white solid, mp: 96-97 °C; ^{1}H NMR (400 MHz, $CDCl_3$) δ 0.84 (t, *J* = 7.2 Hz, 3H), 1.13-1.41 (m, 3H), 1.43-1.56 (m, 1H), 1.81-1.92 (m, 2H), 1.93-2.03 (m, 1H), 2.70-2.80 (m, 1H), 3.17 (s, 3H), 3.33 (s, 6H), 3.59 (d, *J* = 12.4 Hz, 1H), 6.09 (s, 1H), 6.52 (s, 2H), 6.97-7.05 (m, 2H), 7.06-7.10 (m, 4H), 7.18-7.25 (m, 2H), 7.26-7.29 (m, 1H); ^{13}C NMR (100 MHz, $CDCl_3$) δ 13.9, 22.3, 30.0, 35.6, 35.7, 38.5, 56.0, 119.5, 119.7, 124.4, 125.4, 125.8, 126.6, 127.5, 128.3, 130.3, 137.7, 143.8, 145.7, 151.6, 181.6; ^{11}B NMR (128.4 MHz, $CDCl_3$): δ -27.1 (t, *J* = 90.3 Hz, 1B); ESIHRMS: Found: *m/z* 450.2693. Calcd for $C_{27}H_{34}{}^{11}BN_3NaO_3$: $(M+Na)^+$ 450.2693.

(*Z*)-(1-Allyl-5-butyl-2-oxo-4-phenyl-1,2,3,4-tetrahydrobenzo[*b*]azocin-3-yl)(1,3-dimethyl-1*H*-imidazol-3-ium-2-yl)dihydroborate (3x)

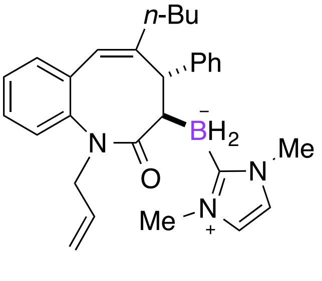

According to the general procedure, the reaction of *N*-allyl-*N*-(2-(hex-1-yn-1-yl)phenyl)cinnamamide (139.3 mg, 0.406 mmol), NHC-BH_3 (54.0 mg, 0.491 mmol), AIBN (14.1 mg, 0.086 mmol), isooctyl thioglycolate (16.5 mg, 0.081 mmol), and MeCN (5 mL), under nitrogen at 80 °C for 12 h after flash column chromatography on silica gel (petroleum ether : ethyl acetate = 30 : 70) afforded 86.5 mg (47%) **3x** as a white solid, mp: 115-116 °C; ^{1}H NMR (400 MHz, $CDCl_3$) δ 0.86 (t, *J* = 7.2 Hz, 3H), 1.16-1.42 (m, 3H), 1.46-1.58 (m, 1H), 1.84-2.03 (m, 2H), 2.65-2.79 (m, 1H), 3.39 (s, 6H), 3.61 (d, *J* = 12.4 Hz, 1H), 4.17-4.30 (m, 2H), 4.81-4.88 (tdd, *J* = 1.2, 1.2, 17.2 Hz, 1H), 4.89-4.93 (tdd, J = 1.2, 1.2, 10.4 Hz, 1H), 5.63-5.74 (m, 1H), 6.10 (s, 1H), 6.58 (s, 2H), 7.0-7.08 (m, 2H), 7.08-7.14 (m, 4H), 7.14-7.20 (m, 1H), 7.25-7.25 (m, 1H), 7.26-7.31 (m, 1H); ^{13}C NMR (100 MHz, $CDCl_3$) δ 13.9, 22.4, 30.2, 35.7, 38.6, 50.9, 55.9, 115.5, 119.8, 120.1, 124.2, 125.5, 125.8, 126.5, 127.5, 128.5, 130.2, 135.0, 138.2, 142.7, 145.7, 151.2, 180.9; ^{11}B NMR (128.4 MHz, $CDCl_3$): δ -26.9 (t, *J* = 87.8 Hz, 1B); ESIHRMS: Found: *m/z* 476.2851. Calcd for $C_{29}H_{36}{}^{11}BN_3NaO$: $(M+Na)^+$ 476.2849.

(*Z*)-(5-Butyl-4-(4-chlorophenyl)-1-methyl-2-oxo-1,2,3,4-tetrahydrobenzo[*b*]azocin-3-yl)(1,3-dimethyl-1*H*-imidazol-3-ium-2-yl)dihydroborate (3y)

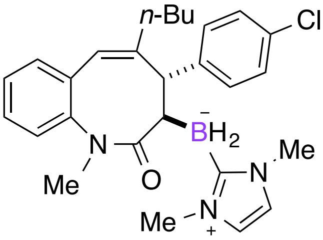

According to the general procedure, the reaction of (*E*)-3-(4-chlorophenyl)-*N*-(2-(hex-1-yn-1-yl)phenyl)-*N*-methylacrylamide (169.7 mg, 0.482 mmol), NHC-BH_3 (64.5 mg, 0.587 mmol), AIBN (16.4 mg, 0.100 mmol), isooctyl thioglycolate (19.4 mg, 0.095 mmol), and MeCN (5 mL), under nitrogen at 80 °C for 12 h after flash column chromatography on silica gel (petroleum ether : ethyl acetate = 30 : 70) afforded 122.4 mg (55%) **3y** as a white solid, mp: 155-156 °C; ^{1}H NMR (400 MHz, $CDCl_3$) δ 0.84 (t, *J* = 7.2 Hz, 3H), 1.13-1.40 (m, 3H), 1.41-1.54 (m, 1H), 1.81-2.00 (m, 2H), 2.61-2.71 (m, 1H), 3.12 (s, 3H), 3.37 (s, 6H), 3.57 (d, *J* = 12.4 Hz, 1H), 6.12 (s, 1H), 6.59 (s, 2H), 6.95-6.99 (m, 1H), 7.02-7.09 (m, 4H), 7.17-7.23 (m, 2H), 7.24-7.28 (m, 1H); ^{13}C NMR (100 MHz, $CDCl_3$) δ 13.8, 22.2, 30.0, 35.5, 35.7, 38.6, 55.4, 119.8, 119.9, 124.4, 125.9, 126.8, 127.6, 129.7, 130.2, 131.0, 137.4, 143.6, 144.3, 151.1, 181.2; ^{11}B NMR (128.4 MHz, $CDCl_3$): δ -27.1 (t, *J* = 87.8 Hz, 1B); ESIHRMS: Found: *m/z* 484.2313. Calcd for $C_{27}H_{33}{}^{11}BClN_3NaO$: $(M+Na)^+$ 484.2303.

(*Z*)-(5-Butyl-8-chloro-1-methyl-2-oxo-4-phenyl-1,2,3,4-tetrahydrobenzo[*b*]azocin-3-yl)(1,3-dimethyl-1*H*-imidazol-3-ium-2-yl)dihydroborate (3z)

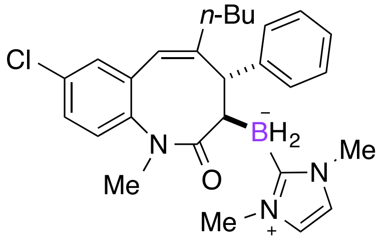

According to the general procedure, the reaction of *N*-(4-chloro-2-(hex-1-yn-1-yl)phenyl)-*N*-methylcinnamamide (144.6 mg, 0.411 mol), NHC-BH_3 (55.5 mg, 0.505 mmol), AIBN (13.7 mg, 0.083 mmol), isooctyl thioglycolate (16.8 mg, 0.082 mmol), and MeCN (5 mL), under nitrogen at 80 °C for 12 h after flash column chromatography on silica gel (petroleum ether : ethyl acetate = 30 : 70) afforded 111.7 mg (62%) **3z** as a white solid, mp: 167-168 °C; ^{1}H NMR (400 MHz, $CDCl_3$) δ 0.84 (t, *J* = 7.2 Hz, 3H), 1.12-1.39 (m, 3H), 1.42-1.54 (m, 1H), 1.81-1.91 (m, 1H), 1.93-2.03 (m, 1H), 2.65-2.76 (m, 1H), 3.14 (s, 3H), 3.35 (s, 6H), 3.60 (d, *J* = 12.4 Hz, 1H), 6.01 (s, 1H), 6.53 (s, 2H), 6.96 (d, *J* = 8.4 Hz, 1H), 7.00-7.08 (m, 3H), 7.09-7.13 (m, 2H), 7.15-7.19 (m, 1H), 7.26-7.28 (m, 1H); ^{13}C NMR (100 MHz, $CDCl_3$) δ 13.8, 22.2, 30.0, 35.65, 35.7, 38.5, 56.0, 118.4, 119.8, 125.58, 125.6, 126.7, 127.6, 128.3, 130.0, 131.0, 139.4, 142.5, 145.2, 153.1, 181.4; ^{11}B NMR (128.4 MHz, $CDCl_3$): δ -27.1 (t, *J* = 90.8 Hz, 1B); ESIHRMS: Found: *m/z* 484.2310. Calcd for $C_{27}H_{33}{}^{11}BN_3ONaCl$: $(M+Na)^+$ 484.2303.

(*Z*)-(5-Butyl-1-methyl-2-oxo-4-(pyridin-3-yl)-1,2,3,4-tetrahydrobenzo[*b*]azocin-3-yl)(1,3-dimethyl-1*H*-imidazol-3-ium-2-yl)dihydroborate (3aa)

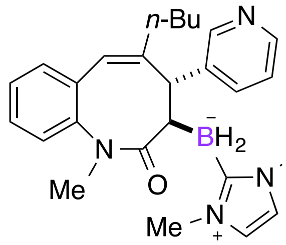

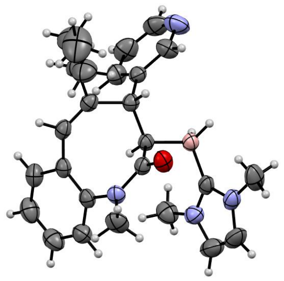

According to the general procedure, the reaction of (*E*)-*N*-(2-(hex-1-yn-1-yl)phenyl)-*N*-methyl-3-(pyridin-3-yl)acrylamide (150.4 mg, 0.472 mmol), NHC-BH_3 (62.1 mg, 0.565 mmol), AIBN (16.0 mg, 0.097 mmol), isooctyl thioglycolate (19.4 mg, 0.095 mmol), and MeCN (5 mL), under nitrogen at 80 °C for 12 h after flash column chromatography on silica gel (ethyl acetate) afforded 117.3 mg (58%) **3aa**

as a white solid, mp: 157-158 °C; recrystallization from petroleum ether/ethyl acetate gave colorless crystals (CCDC: 1866487); ^{1}H NMR (400 MHz, $CDCl_3$) δ 0.83 (t, *J* = 7.2 Hz, 3H), 1.13-1.39 (m, 3H), 1.41-1.54 (m, 1H), 1.81-1.99 (m, 2H), 2.62-2.77 (m, 1H), 3.11 (s, 3H), 3.38 (s, 6H), 3.59 (d, *J* = 12.4 Hz, 1H), 6.16 (s, 1H), 6.60 (s, 2H), 6.95 (dd, *J* = 2.0, 6.8 Hz, 1H), 7.03 (dd, *J* = 4.8, 7.6 Hz, 1H), 7.15-7.25 (m, 2H), 7.26-7.30 (m, 1H), 7.38-7.44 (m, 1H), 8.28 (dd, *J* = 1.6, 4.8 Hz, 1H), 8.40 (d, *J* = 1.6 Hz, 1H); ^{13}C NMR (100 MHz, $CDCl_3$) δ 13.8, 22.2, 30.0, 35.5, 35.7, 38.6, 53.6, 119.9, 120.5, 122.6, 124.4, 126.1, 126.9, 130.3, 135.9, 137.2, 141.0, 143.5, 146.9, 149.9, 150.3, 180.9; ^{11}B NMR (128.4 MHz, $CDCl_3$): δ -27.1 (t, *J* = 91.2 Hz, 1B); ESIHRMS: Found: *m/z* 429.2823. Calcd for $C_{26}H_{34}{}^{11}BN_4O$: $(M+Na)^+$ 429.2826.

Supplementary Table 5. Crystal data and structure refinement for **3aa**

Empirical formula	$C_{17.33}H_{22}B_{0.67}N_{2.67}O_{0.67}$
Formula weight	285.58
Temperature/K	291(2)
Crystal system	triclinic
Space group	P-1
a/Å	8.4651(2)
b/Å	9.9833(4)
c/Å	14.3752(5)
α/°	84.806(3)
β/°	88.032(3)
γ/°	88.416(3)
Volume/Å^3	1208.77(7)
Z	3
ϱ_{calc}g/cm^3	1.177
μ/mm^{-1}	0.562
F(000)	460.0
Crystal size/mm^3	0.200 × 0.190 × 0.170
Radiation	CuKα (λ = 1.54184)
2Θ range for data collection/°	8.896 to 142.402
Index ranges	-10 ≤ h ≤ 8, -12 ≤ k ≤ 12, -17 ≤ l ≤ 16
Reflections collected	7740
Independent reflections	4513 [R_{int} = 0.0174, R_{sigma} = 0.0247]
Data/restraints/parameters	4513/0/302
Goodness-of-fit on F^2	1.047

Final R indexes [I>=2σ (I)]	R_1 = 0.0533, wR_2 = 0.1538
Final R indexes [all data]	R_1 = 0.0588, wR_2 = 0.1600
Largest diff. peak/hole / e Å^{-3}	0.56/-0.27

(1,3-Dimethyl-1*H*-imidazol-3-ium-2-yl)(3-oxo-1-phenylbutan-2-yl)dihydroborate (3ab)

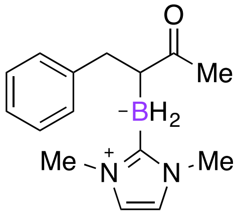

According to the general procedure, the reaction of (*E*)-4-phenylbut-3-en-2-one (87.8 mg, 0.600 mmol), NHC-BH_3 (55.1 mg, 0.501 mmol), AIBN (16.4mg, 0.100 mmol), PhSH (10.8 mg, 0.098 mmol), and MeCN (5 mL), under nitrogen at 80 °C for 12 h after flash column chromatography on silica gel (petroleum ether : ethyl acetate = 70 : 30) afforded 120.7 mg (94%) **3ab** as a pale yellow liquid; ^{1}H NMR (400 MHz, $CDCl_3$) δ 2.00 (s, 3H), 2.49-2.68 (m, 2H), 3.09 (dd, *J* = 9.6, 14.4 Hz, 1H), 3.70 (s, 6H), 6.79 (s, 2H), 7.06 (t, *J* = 7.2 Hz, 1H), 7.11 (d, *J* = 7.6 Hz, 2H), 7.17 (dd, *J* = 7.6, 7.6 Hz, 2H); ^{13}C NMR (100 MHz, $CDCl_3$) δ 28.2, 36.1, 38.3, 120.6, 125.0, 127.9, 128.2, 144.3, 217.5; ^{11}B NMR (128.4 MHz, $CDCl_3$): δ -25.3 (t, *J* = 90.3 Hz, 1B); ESIHRMS: Found: *m/z* 279.1648. Calcd for $C_{15}H_{21}{}^{11}BN_2NaO$: $(M+Na)^+$ 279.1645.

(1,3-Dimethyl-1*H*-imidazol-3-ium-2-yl)(1-(1-methyl-1*H*-imidazol-2-yl)-1-oxo-3-phenylpropan-2-yl)dihydroborate (3ac)

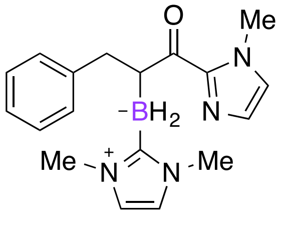

According to the general procedure, the reaction of (*E*)-1-(1-methyl-1*H*-imidazol-2-yl)-3-phenylprop-2-en-1-one (127.1 mg, 0.599 mmol), NHC-BH_3 (55.0 mg, 0.500 mmol), AIBN (16.4mg, 0.100 mmol), PhSH (10.8 mg, 0.098 mmol), and MeCN (5 mL), under nitrogen at 80 °C for 12 h after flash column chromatography on silica gel (petroleum ether : ethyl acetate = 20 : 80) afforded 138.4 mg (86%) **3ac** as a pale yellow solid, mp: 113-114 °C; ^{1}H NMR (400 MHz, $CDCl_3$) δ 2.76 (dd, *J* = 4.8, 14.4 Hz, 1H), 3.37 (dd, *J* = 9.2, 14.4 Hz, 1H), 3.59 (s, 6H), 3.79-3.88 (m, BH_2CH, 1H),

3.89 (s, 3H), 6.60 (s, 2H), 6.77 (d, J = 7.2 Hz, 2H), 7.01 (t, J = 7.2 Hz, 1H), 7.13 (dd, J = 7.2, 7.2 Hz, 2H), 7.24 (d, J = 7.2 Hz, 1H), 7.26 (d, J = 7.2 Hz, 1H); ^{13}C NMR (100 MHz, $CDCl_3$) δ 35.7, 35.8, 37.8, 120.0, 124.7, 124.8, 127.3, 127.6, 128.6, 144.56, 144.60, 200.6; ^{11}B NMR (128.4 MHz, $CDCl_3$): δ -24.5 (t, J = 90.1 Hz, 1B); ESIHRMS: Found: *m/z* 323.2039. Calcd for $C_{18}H_{24}{}^{11}BN_4NaO$: $(M+H)^+$ 323.2043.

(1,3-Dimethyl-1*H*-imidazol-3-ium-2-yl)(1-(4-hydroxy-3-methoxyphenyl)-1-oxo-3-(3,4,5-trimethoxyphenyl)propan-2-yl)dihydroborate (3ad)

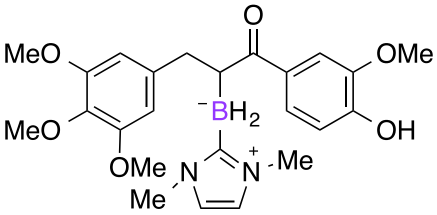

According to the general procedure, the reaction of (*E*)-1-(4-hydroxy-3-methoxyphenyl)-3-(3,4,5-trimethoxyphenyl)prop-2-en-1-one (83.2 mg, 0.242 mmol), NHC-BH_3 (22.3 mg, 0.203 mmol), AIBN (6.7 mg, 0.040 mmol), PhSH (4.3 mg, 0.039 mmol), and MeCN (3 mL), under nitrogen at 80 °C for 12 h after flash column chromatography on silica gel (ethyl acetate) afforded 83.6 mg (91%) **3ad** as a white solid, mp: 144-145 ° C; ^{1}H NMR (400 MHz, $CDCl_3$) δ 2.78 (dd, J = 3.6, 14.0 Hz, 1H), 3.33 (dd, J = 10.0, 14.0 Hz, 1H), 3.39-3.51 (m, BH_2CH, 1H), 3.60 (s, 6H), 3.71-3.77 (m, 9H), 3.88 (s, 3H), 5.93 (s br, 1H), 6.42 (s, 2H), 6.70 (s, 2H), 6.77 (d, J = 8.4 Hz, 1H), 7.36 (dd, J = 1.6, 8.4 Hz, 1H), 7.48 (d, J = 1.6 Hz, 1H); ^{13}C NMR (100 MHz, $CDCl_3$) δ 36.2, 39.3, 55.85, 55.9, 60.7, 105.3, 109.9, 113.1, 120.5, 122.0, 132.1, 135.3, 141.0, 146.1, 148.6, 152.5, 207.0; ^{11}B NMR (128.4 MHz, $CDCl_3$): δ -24.4 (t, J = 90.1 Hz, 1B). ESIHRMS: Found: *m/z* 477.2173. Calcd for $C_{24}H_{31}{}^{11}BN_2NaO_6$: $(M+Na)^+$ 477.2173.

(1-Carboxy-2-phenylethyl)(1,3-dimethyl-1*H*-imidazol-3-ium-2-yl)dihydroborate (3ae)

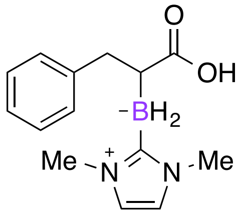

According to the general procedure, the reaction of cinnamic acid (88.8 mg, 0.608 mmol), NHC-BH_3 (55.2 mg, 0.502 mmol), AIBN (16.4 mg, 0.100 mmol), PhSH (10.8mg, 0.098 mmol), and MeCN (5 mL), under nitrogen at 80 °C for 12 h after flash column

chromatography on silica gel (petroleum ether : ethyl acetate = 40 : 60) afforded 90.6 mg (70%) **3ae** as a white solid, mp: 134-135 °C; ^{1}H NMR (400 MHz, $CDCl_3$) δ 2.16 (m, BH_2CH, 1H), 2.73 (dd, J = 3.6, 14.4 Hz, 1H), 3.05 (dd, J = 10.4, 14.4 Hz, 1H), 3.62 (s, 6H), 6.68 (s, 2H), 7.07-7.15 (m, 1H), 7.17-7.25 (m, 4H); ^{13}C NMR (100 MHz, $CDCl_3$) δ 35.8, 38.6, 120.4, 125.0, 127.8, 128.5, 144.7, 188.5; ^{11}B NMR (128.4 MHz, $CDCl_3$): δ -24.6 (t, J = 89.9 Hz, 1B); ESIHRMS: Found: m/z 281.1438. Calcd for $C_{14}H_{19}{}^{11}BN_2NaO_2$: $(M+Na)^+$ 281.1437.

(1,3-Dimethyl-1*H*-imidazol-3-ium-2-yl)(1-ethoxy-1-oxobutan-2-yl)dihydroborate (3af-α)

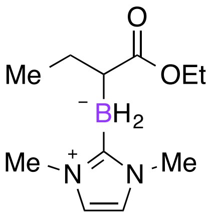

According to the general procedure, the reaction of ethyl (*E*)-but-2-enoate (68.9 mg, 0.603 mmol), NHC-BH_3 (55.0 mg, 0.500 mmol), AIBN (16.4 mg, 0.100 mmol), *tert*-dodecanethiol (50.7 mg, 0.251 mmol), and MeCN (5 mL), under nitrogen at 80 °C for 12 h after flash column chromatography on silica gel (petroleum ether : ethyl acetate = 70 : 30) afforded 45.9 mg (41%) α-addition product **3af-α** as a white solid, mp: 45-46 °C. ^{1}H NMR (400 MHz, $CDCl_3$) δ 0.86 (t, J = 6.8 Hz, 3H), 1.03 (t, J = 7.2 Hz, 3H), 1.33-1.42 (m, BH_2CH, 1H), 1.71-1.77 (m, 2H), 3.72 (s, 6H), 3.78-3.92 (m, 2H), 6.81 (s, 2H); ^{13}C NMR (100 MHz, $CDCl_3$) δ 14.4, 14.9, 26.3, 36.0, 58.3, 120.2, 182.5; ^{11}B NMR (128.4 MHz, $CDCl_3$): δ -25.3 (t, J = 89.1 Hz, 1B); ESIHRMS: Found: m/z 247.1592. Calcd for $C_{11}H_{21}{}^{11}BN_2NaO_2$: $(M+Na)^+$ 247.1594.

In addition, *β*-addition product **3af-β** was isolated in 26% yield (29.4 mg).

(1,3-Dimethyl-1*H*-imidazol-3-ium-2-yl)(4-ethoxy-4-oxobutan-2-yl)dihydroborate (3af-β)

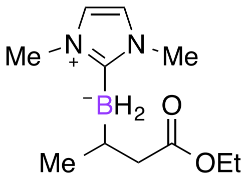

Colorless liquid; ^{1}H NMR (400 MHz, $CDCl_3$) δ 0.81 (d, J = 5.2 Hz, 3H), 1.13-1.24 (m, 4H), 2.09 (dd, J = 8.4, 13.6 Hz, 1H), 2.20 (dd, J = 6.4, 13.6 Hz, 1H), 3.76 (s, 6H), 4.02 (q, J = 7.2 Hz, 2H), 6.79 (s, 2H); ^{13}C NMR (100 MHz, $CDCl_3$) δ 14.4, 22.4, 36.0, 44.6, 59.3, 120.1, 176.4; ^{11}B NMR (128.4 MHz, $CDCl_3$): δ -24.1 (t, J = 85.3 Hz, 1B); ESIHRMS: Found: m/z 247.1595. Calcd for $C_{11}H_{21}{}^{11}BN_2NaO_2$: $(M+Na)^+$ 247.1594.

(1,3-Dimethyl-1*H*-imidazol-3-ium-2-yl)(1-((4-methylphenyl)sulfonamido)-1-oxobutan-2-yl)dihydroborate (3ag-α)

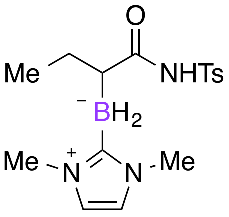

According to the general procedure, the reaction of (*E*)-*N*-tosylbut-2-enamide (143.9 mg, 0.601 mmol), NHC-BH_3 (55.4 mg, 0.504 mmol), AIBN (16.4 mg, 0.100 mmol), *tert*-dodecanethiol (50.7 mg, 0.251 mmol), and MeCN (5 mL), under nitrogen at 80 °C for 12 h after flash column chromatography on silica gel (petroleum ether : ethyl acetate = 50 : 50) afforded 86.8 mg (49%) **3ag-α** as a colorless liquid; the product contained an inseparable unknown impurity (this impurity was not the regioisomer); ^{1}H NMR (400 MHz, $CDCl_3$) δ 0.74 (t, *J* = 7.2 Hz, 3H), 1.26-1.40 (m, 1H), 1.46-1.56 (m, 1H), 1.57-1.65 (m, 1H), 2.40 (s, 3H), 3.66 (s, 6H), 6.81 (s, 2H), 7.27 (d, *J* = 8.0 Hz, 2H), 7.90 (d, *J* = 8.0 Hz, 2H), 8.04 (s br, 1H); ^{13}C NMR (100 MHz, $CDCl_3$) δ 14.4, 21.5, 26.0, 36.0, 120.7, 128.2, 129.2, 136.7, 144.0, 180.0; ^{11}B NMR (128.4 MHz, $CDCl_3$): δ -25.3 (t, *J* = 88.5 Hz, 1B); ESIHRMS: Found: *m/z* 372.1522. Calcd for $C_{16}H_{24}{}^{11}BN_3NaO_3S$: $(M+Na)^+$ 372.1524.

(1,3-Dimethyl-1H-imidazol-3-ium-2-yl)(1-ethoxy-1-oxohex-3-en-2-yl)dihydroborate (3ah)

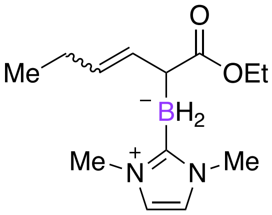

According to the general procedure, the reaction of ethyl (*E*)-3-cyclopropylacrylate (84.5 mg, 0.603 mmol), NHC-BH_3 (55.1 mg, 0.501 mmol), AIBN (16.4 mg, 0.100 mmol) and *tert*-dodecanethiol (50.7 mg, 0.251 mmol) in CH_3CN (5 mL), under nitrogen at 80 °C for 12 h after flash column chromatography on silica gel (petroleum ether : ethyl acetate = 70 : 30) afforded 79.8 mg (64%, *E*/*Z* = 2:1) **3ah** as a colorless liquid; ^{1}H NMR (400 MHz, $CDCl_3$) δ 0.84 (t, *J* = 7.2 Hz, 3Hx1), 0.88 (t, *J* = 7.2 Hz, 3Hx2), 1.12 (t, *J* = 7.2 Hz, 3Hx1+3Hx2), 1.61-1.74 (m, 2Hx1), 1.81-1.95 (m, 2Hx2), 2.56-2.69 (m, BH_2C*H,* 1Hx2), 2.87-2.99 (m, BH_2C*H,* 1Hx1), 3.73 (s, 6Hx2), 3.75 (s, 6Hx1), 3.85-4.04 (m, 2Hx1+2Hx2), 4.88-5.01 (m, 1Hx1+1Hx2), 5.7-5.84 (m, 1Hx1+1Hx2), 6.82 (s, 2Hx1+2Hx2); ^{13}C NMR (100 MHz, $CDCl_3$)

δ 14.1, 14.40, 14.44, 20.2, 25.6, 36.1, 36.2, 58.8, 58.9, 120.3, 120.4, 124.5, 125.4, 132.5, 132.7, 179.9, 180.1; ^{11}B NMR (128.4 MHz, $CDCl_3$): δ -25.1 (t, J = 91.5 Hz, 1Bx1), -24.6 (t, J = 91.9 Hz, 1Bx2); ESIHRMS: Found: *m/z* 273.1748. Calcd for $C_{13}H_{23}{}^{11}BN_2NaO_2$: $(M+Na)^+$ 273.1750.

(1,3-Dimethyl-1*H*-imidazol-3-ium-2-yl)(1-ethoxy-3-methyl-1-oxobutan-2-yl)dihydroborate (3ai)

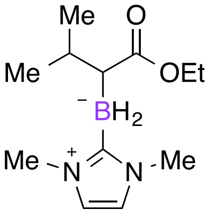

According to the general procedure, the reaction of ethyl 3-methylbut-2-enoate (77.4 mg, 0.604 mmol), NHC-BH_3 (55.0 mg, 0.500 mmol), AIBN (16.4 mg, 0.100 mmol), *tert*-dodecanethiol (50.7 mg, 0.251 mmol), and MeCN (5 mL), under nitrogen at 80 °C for 12 h after flash column chromatography on silica gel (petroleum ether : ethyl acetate = 70 : 30) afforded 67 mg (56%) **3ai** as a white solid, mp: 69-70 °C; ^{1}H NMR (400 MHz, $CDCl_3$) δ 0.92 (d, J = 6.4 Hz, 3H), 0.95-1.04 (m, 6H), 1.46-1.59 (m, BH_2CH, 1H), 1.89-1.99 (m, 1H), 3.64-3.86 (m, 8H), 6.80 (s, 2H); ^{13}C NMR (100 MHz, $CDCl_3$) δ 14.3, 21.9, 23.7, 31.3, 36.0, 58.1, 120.2, 182.2; ^{11}B NMR (128.4 MHz, $CDCl_3$): δ -26.3 (t, J = 89.4 Hz, 1B); ESIHRMS: Found: *m/z* 261.1751. Calcd for $C_{12}H_{23}{}^{11}BN_2NaO_2$: $(M+Na)^+$ 261.1750.

(3-(Cyclohex-3-en-1-yl)-1-ethoxy-1-oxopropan-2-yl)(1,3-dimethyl-1*H*-imidazol-3-ium-2-yl)dihydroborate (3aj)

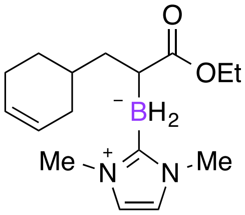

According to the general procedure, the reaction of ethyl (*E*)-3-(cyclohex-3-en-1-yl)acrylate (108.1 mg, 0.600 mmol), NHC-BH_3 (55.3 mg, 0.503 mmol), AIBN (16.4 mg, 0.100 mmol), PhSH (10.8 mg, 0.098 mmol), and MeCN (5 mL), under nitrogen at 80 °C for 12 h after flash column chromatography on silica gel (petroleum ether : ethyl acetate = 70 : 30) afforded 52.8 mg (36%) **3aj** (a mixture of diastereoisomers, dr = 1:1, determined by ^{13}C NMR) as a colorless liquid; ^{1}H and ^{11}B NMR spectra of two diastereomers were identical; ^{1}H NMR (400

MHz, $CDCl_3$) δ 1.02 (t, *J* = 7.2 Hz, 3H), 1.08-1.20 (m, 1H), 1.21-1.36 (m, 1H), 1.54-1.69 (m, 2H), 1.72-1.88 (m, 2H), 1.90-2.16 (m, 4H), 3.74 (s, 6H), 3.75-3.92 (m, 2H), 5.61 (m, 2H), 6.81 (s, 2H); ^{13}C NMR (100 MHz, $CDCl_3$) δ 14.3, 25.37, 25.4, 28.6, 29.6, 31.7, 32.4, 33.6, 33.9, 36.0, 40.1, 40.5, 58.3, 120.2, 126.8, 126.9, 127.0, 127.1, 182.4, 182.5; ^{11}B NMR (128.4 MHz, $CDCl_3$): δ -24.9 (t, *J* = -89.9 Hz, 1B); ESIHRMS: Found: *m/z* 313.2061. Calcd for $C_{16}H_{27}{}^{11}BN_2NaO_2$: $(M+Na)^+$ 313.2063.

(1,3-Dimethyl-1*H*-imidazol-3-ium-2-yl)(3-oxo-1-(2,6,6-trimethylcyclohex-1-en-1-yl)butan-2-yl)dihydroborate (3ak)

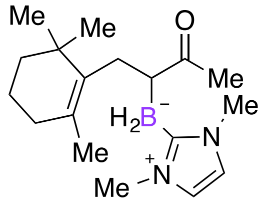

According to the general procedure, the reaction of (*E*)-4-(2,6,6-trimethylcyclohex-1-en-1-yl)but-3-en-2-one (115.3 mg, 0.600 mmol), NHC-BH_3 (55.1 mg, 0.501 mmol), AIBN (16.4 mg, 0.100 mmol), PhSH (10.8 mg, 0.098 mmol), and MeCN (5 mL), under nitrogen at 80 °C for 12 h after flash column chromatography on silica gel (petroleum ether : ethyl acetate = 70 : 30) afforded 106.0 mg (70%) **3ak** as a colorless liquid; ^1H NMR (400 MHz, $CDCl_3$) δ 0.84 (s, 3H), 0.93 (s, 3H), 1.27-1.33 (m, 2H), 1.42-1.53 (m, 5H), 1.83 (t, *J* = 6.4 Hz, 2H), 1.89 (s, 3H), 1.92-2.01 (m, 1H), 2.17-2.29 (m, BH_2CH, 1H), 2.66 (dd, *J* = 8.4, 14.4 Hz, 1H) 3.74 (s, 6H), 6.83 (s, 2H); ^{13}C NMR (100 MHz, $CDCl_3$) δ 19.4, 20.4, 28.6, 28.8, 29.3, 30.4, 33.0, 34.4, 36.1, 40.6, 120.6, 126.0, 140.7, 217.4; ^{11}B NMR (128.4 MHz, $CDCl_3$): δ -24.7 (t, *J* = 88.7 Hz, 1B); ESIHRMS: Found: *m/z* 325.2428. Calcd for $C_{18}H_{31}{}^{11}BN_2NaO$: $(M+Na)^+$ 325.2427.

(1,3-Dimethyl-1*H*-imidazol-3-ium-2-yl)(3-methyl-1-((4-methylphenyl)sulfonamido)-1-oxobutan-2-yl)dihydroborate (3al)

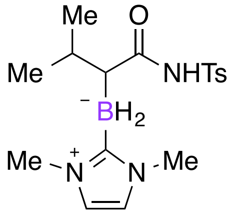

According to the general procedure, the reaction of ethyl (*E*)-*N*-tosylbut-2-enamide (91.7 mg, 0.362 mmol), NHC-BH_3 (33.1 mg, 0.301 mmol), AIBN (10.2 mg, 0.062 mmol), *tert*-dodecanethiol (31.0 mg, 0.153 mmol), and MeCN (3 mL), under nitrogen at 80 °C for 12

h after flash column chromatography on silica gel (petroleum ether : ethyl acetate = 50 : 50) afforded 51.8 mg (47%) **3al** as a white solid, mp: 74-75 °C; ^{1}H NMR (400 MHz, $CDCl_3$) δ 0.70 (d, *J* = 6.4 Hz, 3H), 0.97 (d, *J* = 6.4 Hz, 3H), 1.29-1.40 (m, 1H), 1.62-1.74 (m, 1H), 2.40 (s, 3H), 3.70 (s, 6H), 6.80 (s, 2H), 7.26 (d, *J* = 8.0 Hz, 2H), 7.88 (d, *J* = 8.0 Hz, 2H); ^{13}C NMR (100 MHz, $CDCl_3$) δ 21.5, 22.2, 23.2, 32.0, 36.0, 120.8, 128.2, 129.2, 136.5, 144.1, 179.7; ^{11}B NMR (128.4 MHz, $CDCl_3$): δ -26.1 (t, *J* = 92.8 Hz, 1B); ESIHRMS: Found: *m/z* 386.1676. Calcd for $C_{17}H_{26}{}^{11}BN_3NaO_3S$: $(M+Na)^+$ 386.1680.

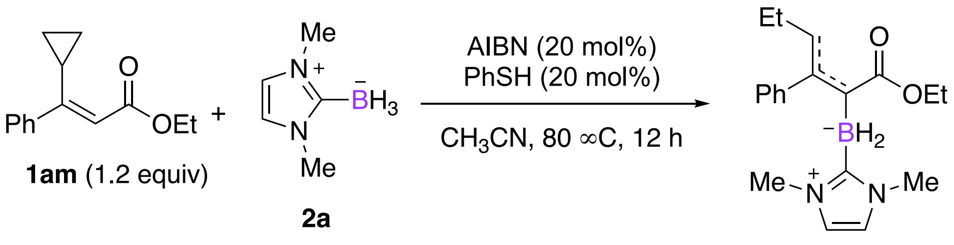

1am (1.2 equiv) **2a** **3am**: 78%

A solution of **1am** (130.3 mg, 0.602 mmol), **2a** (55.8 mg, 0.507 mmol), AIBN (16.6 mg, 0.100 mmol) and PhSH (10.8 mg, 0.098 mmol) in CH_3CN (5 mL) was stirred at 80 °C for 12 h under nitrogen atmosphere. After evaporation of solvent, the resulting crude material was subjected for ^{1}H NMR analysis. The spectrum showed that various isomers were obtained, owing to the easy migration of the C-C double bond. Despite the complexity of the spectrum, the presence of new vinyl and ethyl protons and their splitting patterns clearly confirmed the formation of ring opening products. Purification of the crude reaction mixture by flash column chromatography (silica gel; petroleum ether : ethyl acetate = 70 : 30) gave a pure single isomer and a mixture of other isomers in total 78% yield (129.3 mg). The characterization data were given for the pure single isomer. Colorless liquid; ^{1}H NMR (400 MHz, $CDCl_3$) δ 0.93 (t, *J* = 7.6 Hz, 3H), 1.03 (t, *J* = 7.2 Hz, 3H), 1.90-2.02 (m, 2H), 2.83-2.95 (m, 1H), 3.68 (s, 6H), 3.88 (q, *J* = 7.2 Hz, 2H), 5.90 (t, *J* = 7.2 Hz, 1H), 6.78 (s, 2H), 7.07-7.14 (m, 3H), 7.17-7.25 (m, 2H); ^{13}C NMR (100 MHz, $CDCl_3$) δ 14.3, 14.9, 22.8, 36.0, 58.8, 120.3, 125.5, 127.4, 128.5, 128.8, 141.0, 144.5, 179.9; ^{11}B NMR (128.4 MHz, $CDCl_3$): δ -25.3 (t, *J* = 91.5 Hz, 1B); ESIHRMS: Found: *m/z* 349.2053. Calcd for $C_{19}H_{27}{}^{11}BN_2NaO_2$: $(M+Na)^+$ 349.2058.

4. Synthetic applications

4.1 Synthesis of pinacol boronic ester 4

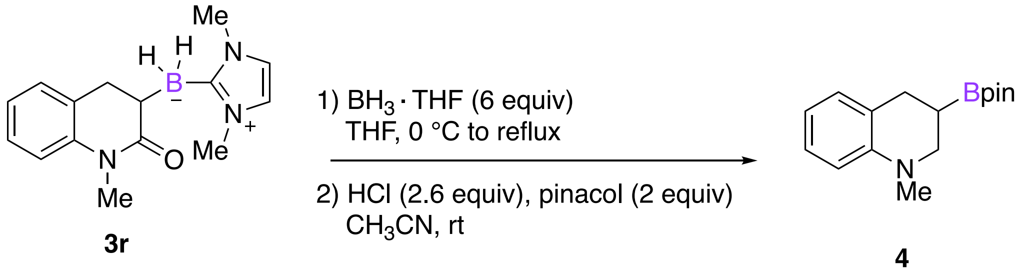

To a solution of **3r** (331.3 mg, 1.231 mmol) in anhydrous THF was slowly added a solution of BH_3•THF (7.4 mL, 7.4 mmol) at 0 °C. The reaction mixture was warm to reflux and stirred under nitrogen atmosphere for 2 hours. The solvent was removed under *vacuo,* follow by adding 30 mL of MeOH, and the mixture was heated to refluxed for 1 hour. After evaporation under *vacuo*, the residue was dissolved in 10 mL MeCN, HCl (1.6 mL, 2 M in water) and pinacol (292.9 mg, 2.479 mmol) were then added. The reaction mixture was stirred under nitrogen atmosphere for 2 hours at room temperature and quenched with saturated $NaHCO_3$ aqueous. The reaction mixture was extracted three times with ethyl acetate. The combined extracts were washed with brine, dried over Na_2SO_4, and concentrated in *vacuo*. The crude material was purified by flash column chromatography (silica gel; petroleum ether : ethyl acetate = 97 : 3) to give **4** (284.0 mg, 1.040 mmol) in 84% yield as a pale yellow solid; mp: 84-85 °C; ^{1}H NMR (400 MHz, $CDCl_3$) δ 1.25 (s, 12H), 1.60-1.72 (m, 1H), 2.71-2.86 (m, 2H), 2.88 (s, 3H), 3.21 (dd, J = 10.8, 10.8 Hz, 1H), 3.29 (ddd, J = 1.6, 4.4, 11.6 Hz, 1H), 6.54-6.62 (m, 2H), 6.92-6.98 (m, 1H), 7.02-7.10 (m, 1H); ^{13}C NMR (100 MHz, $CDCl_3$) δ 24.73, 29.34, 38.88, 52.51, 83.31, 110.73, 115.85, 123.28, 126.87, 128.52, 146.54; ^{11}B NMR (128.4 MHz, $CDCl_3$): δ 33.7 (s br, 1B); ESIHRMS: Found: *m/z* 273.1972. Calcd for $C_{16}H_{25}{}^{11}BNO_2$: $(M+H)^+$ 273.1978.

4.2 Synthesis of furan-substituted tetrahydroquinolines 5

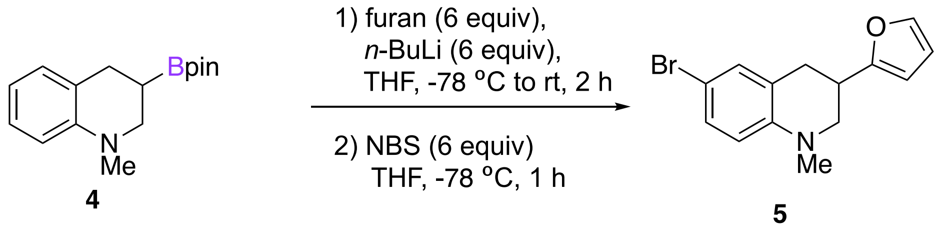

A solution of furan (63.9 mg, 0.939 mmol) in THF (1.5 mL) was cooled to –78 °C and treated with *n*-BuLi (0.6 mL, 1.6 M in hexanes). The cooling bath was removed and the

mixture was stirred at room temperature for 1 h. The mixture was cooled to –78 °C and a solution of boronic ester **4** (42.9 mg, 0.157 mmol) in THF (1.5 mL) was added by dropwise. The mixture was stirred at –78 °C for overnight. A solution of NBS (167.8 mg, 0.943 mmol) in THF (1.5 mL) was added by dropwise. After 1 h at –78 °C, the reaction was quenched with saturated $Na_2S_2O_3$ aqueous, and the reaction mixture was allowed to warm to room temperature. The reaction mixture was diluted with ethyl acetate and water. The layers were separated and the aqueous layer was extracted with ethyl acetate. The combined organic layers were dried anhydrous Na_2SO_4, filtered and concentrated under vacuum. The crude residue was purified by flash column chromatography on silica gel (petroleum ether : ethyl acetate = 95:5) to give **5** (34.4 mg) in 75% yield as a yellow oil. ^{1}H NMR (400 MHz, $CDCl_3$) δ 2.90 (s, 3H), 2.94-3.07 (m, 2H), 3.24-3.37 (m, 2H), 3.44-3.51 (m, 1H), 6.06-6.08 (m, 1H), 6.32 (dd, *J* = 2.0, 3.2 Hz, 1H), 6.47 (d, *J* =8.8 Hz, 1H), 7.08-7.12 (m, 1H), 7.17 (dd, *J* = 2.0, 8.4 Hz, 1H), 7.34-7.38 (m, 1H); ^{13}C NMR (100 MHz, $CDCl_3$) δ 32.26, 32.32, 39.05, 54.81, 104.59, 108.04, 110.15, 112.40, 123.42, 129.88, 131.31, 141.34, 144.93, 156.29; The structure was further confirmed by ^{1}H-^{1}H COSY interactions. ESIHRMS: Found: *m/z* 292.0330. Calcd for $C_{14}H_{14}{}^{79}BrNO$: $(M+H)^+$ 292.0337.

4.3 Synthesis of pyridine-substituted tetrahydroquinolines 6

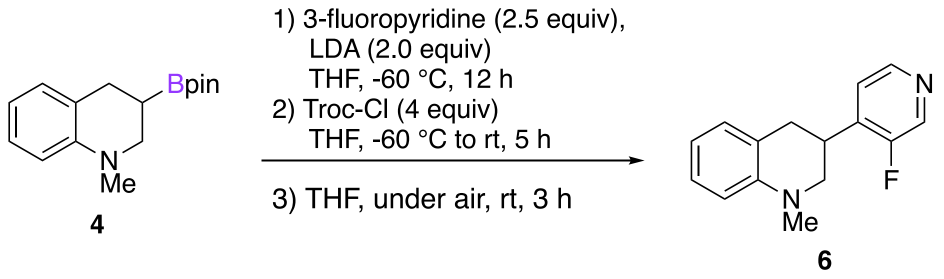

To a solution of $(i\text{-Pr})_2NH$ (82 μl, 0.584 mmol) in THF (1 mL) was cooled to -78 ºC was add *n*-BuLi (0.41 mL, 1.6 M in hexane, 0.643 mmol), and then stirred at -60 ºC for 1 hour. A solution of 3-fluoropyridine (63 μl, 0.730 mmol) in THF (0.5 mL) was added by dropwise and the mixturewas stirred at -60 ºC for 1 h. A solution of **4** (79.8 mg, 0.292 mmol) in THF (1 mL) was added and the miture was stirred for 12 hours at -60 ºC. Troc-Cl (161 μl, 1.168 mmol) was added and the mixture was stirredat -60 ºC for 5 hours and then gradually warmed to room temperature. The mixture was diluted with Et_2O and H_2O,the layers were separated and the aqueous phase was neutralized with saturated aqueous $NaHCO_3$ solution. The aqueous layer was extracted with Et_2O (x2), the combined organic layers were dried over

$MgSO_4$, filtered and evaporated. The residue was disolved in THF (2 mL) under air atomosphere at room temperature for 3 hours. After that, the reaction mixture was diluted with water, and the aqueous phase was extracted with Et_2O (x3). The organic layers were wahsed with brine, dried over $MgSO_4$, filtered and evaporated. Purification by flash chromatography on silica gel (hexanes:EtOAc, 9:1 to 8:2) gave **6** (46.2 mg) in 65% yield as a pale yellow liquid. ^{1}H NMR (400 MHz, $CDCl_3$) δ 2.94 (s, 3H), 3.00-3.11 (m, 2H), 3.30-3.44 (m, 2H), 3.58-3.66 (m, 1H), 6.63-6.72 (m, 2H), 7.02 (d, J = 7.2 Hz, 1H), 7.11-7.17 (m, 2H), 8.30-8.39 (m, 1H), 8.43 (s, 1H); ^{13}C NMR (100 MHz, $CDCl_3$) δ 31.2, 33.0, 39.1, 54.9, 111.2, 116.8, 121.1, 122.6, 127.6, 128.9, 137.9 (d, J = 25.1 Hz); 139.1 (d, J = 12.3 Hz), 145.8, 146.0 (d, J = 5 Hz), 157.8 (d, J = 253.9 Hz); ^{19}F NMR (376 MHz, $CDCl_3$): δ -132.9 (1F, s); ESIHRMS: Found: *m/z* 243.1290. Calcd for $C_{15}H_{16}FN_2$: $(M+H)^+$ 243.1298.

4.4 Synthesis of alcohol derivative 7

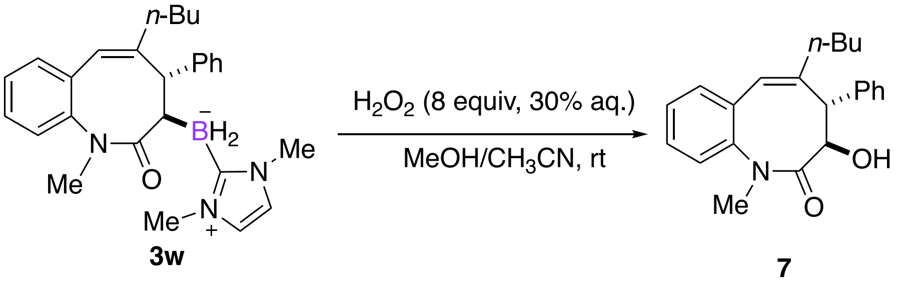

To a solution of **3w** (138.6 mg, 0.324 mmol) in MeOH/CH_3CN (1:1, 4 mL) was added H_2O_2 (30% aq, 0.3 ml) at room temperature. The reaction mixture was stirred for 9 h and quenched with saturated NH_4Cl (aq). The reaction mixture was extracted three times with dichloromethane. The combined extracts were washed with water brine, dried over Na_2SO_4, and concentrated in *vacuo*. The crude material was purified by flash column chromatography (silica gel; petroleum ether : ethyl acetate = 6 : 1) to give **7** (79.5 mg, 0.237 mmol) in 73% yield as a white solid, mp 135-136 °C; ^{1}H NMR (400 MHz, $CDCl_3$) δ 0.87 (t, J = 7.6 Hz, 3H), 1.15-1.40 (m, 3H), 1.41-1.53 (m, 1H), 1.94-2.03 (m, 2H), 3.13 (d, J = 9.2 Hz, 1H), 3.33 (d, J = 10.4 Hz, 1H), 3.35 (s, 3H), 4.33 (dd, J = 9.2, 10.4 Hz, 1H), 6.29 (s, 1H), 7.13-7.18 (m, 2H), 7.18-7.28 (m, 4H), 7.30-7.41 (m, 3H); ^{13}C NMR (100 MHz, $CDCl_3$) δ 13.8, 22.0, 30.1, 36.1, 38.4, 58.0, 71.1, 120.6, 125.7, 127.1, 127.8, 128.2, 128.3, 128.7, 130.2, 136.2, 139.3, 141.0, 146.4, 173.3; ESIHRMS: Found: *m/z* 336.1974. Calcd for $C_{22}H_{26}NO_2$ $(M+H)^+$ 336.1964.

4.5 Synthesis of NHC-difluoroborane 8

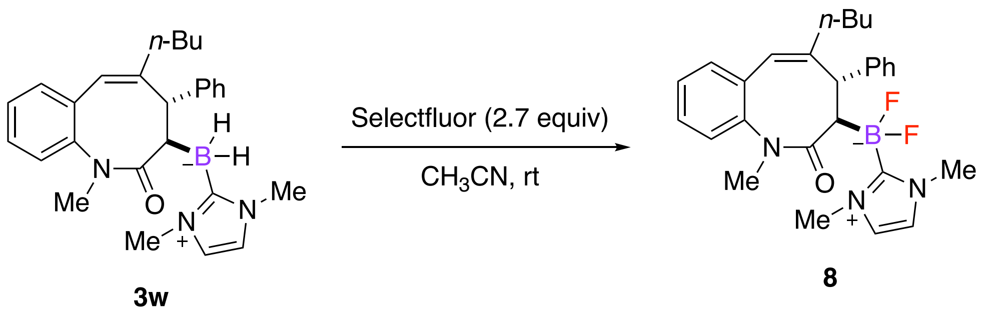

To a solution of **3w** (30.7 mg, 0.0718 mmol) in CH_3CN (1 mL) was added Selectfluor (68.7 mg, 0.1939 mol) at room temperature. The reaction mixture was stirred at room temperature for 2 h and then evaporated. The crude material was purified by flash column chromatography (silica gel; petroleum ether : ethyl acetate = 20 : 80) to give **8** (79.8 mg) in 64% yield as a colorless liquid; ^1H NMR (400 MHz, $CDCl_3$) δ 0.85 (3t, J = 7.2 Hz, 3H), 1.16-1.43 (m, 3H), 1.46-1.56 (m, 1H), 1.84-1.94 (m, 1H), 1.96-2.02 (m, 1H), 2.76-2.88 (m, 1H), 3.14 (s, 3H), 3.54 (s, 6H), 3.89 (d, J = 12.8 Hz, 1H), 6.10 (s, 1H), 6.57 (s, 2H), 7.01-7.07 (m, 1H), 7.08-7.18 (m, 5H), 7.22-7.32 (m, 3H); ^{13}C NMR (100 MHz, $CDCl_3$) δ 13.9, 22.3, 30.0, 35.6, 36.3 (t, J = 4.7 Hz), 38.3, 50.40, 50.44, 119.6, 121.1, 124.6, 125.8, 126.2, 126.9, 127.7, 128.6, 130.5, 137.5, 143.3, 144.7, 151.0, 178.3; ^{11}B NMR (128.4 MHz, $CDCl_3$): δ 4.0 (s br, 1B); ^{19}F NMR ($CDCl_3$, 376 MHz): δ -159.2 – -157.5 (1F, m), -154.9 – -153.4 (1F, m); ESIHRMS: Found: m/z 486.2492. Calcd for $C_{27}H_{32}N_3O^{11}BF_2Na$ $(M+Na)^+$ 486.2499.

5. Computational studies

5.1 DFT calculations of radical borylation of 1ai

The free energy profiles of the reaction between **1ai** and NHC-boryl radical are shown in Supplementary figure 1. The addition of boryl radical to the α-position of **1ai** requires 1.1 kcal mol^{-1} lower than the addition to the β-position (**1ai-TS-1** +11.5 kcal mol^{-1} versus **1ai-TS-1’** +12.6 kcal mol^{-1}). The corresponding **1ai-Int-1** and **1ai-Int-1’** are +3.0 kcal mol^{-1} and +4.0 kcal mol^{-1} in free energy relative to the initial reactants, respectively. The subsequent HAT from thiol to **1ai-Int-1** undergoes easily with a facile energy barrier of +2.4 kcal mol^{-1} (**1ai-Int-1 → 1ai-TS-2**) and an obvious energy decrease of +13.0 kcal mol^{-1} (**1ai-Int-1 → 3ai**). Such an energetically highly favorable HAT process renders the α-addition step irreversible, as well as promotes the α-hydroboration pathway vastly. On the other hand, HAT from thiol to **1ai-Int-1’** requires a much higher energy barrier of +7.3 kcal

mol^{-1} (**1ai-Int-1' →1ai-TS-2'**) and the corresponding product **3ai-*β*** is +4.8 kcal mol^{-1} in free energy above **3ai**. Over all, the α-addition pathway is much favored over the β-addition pathway in both kinetics and thermodynamics.

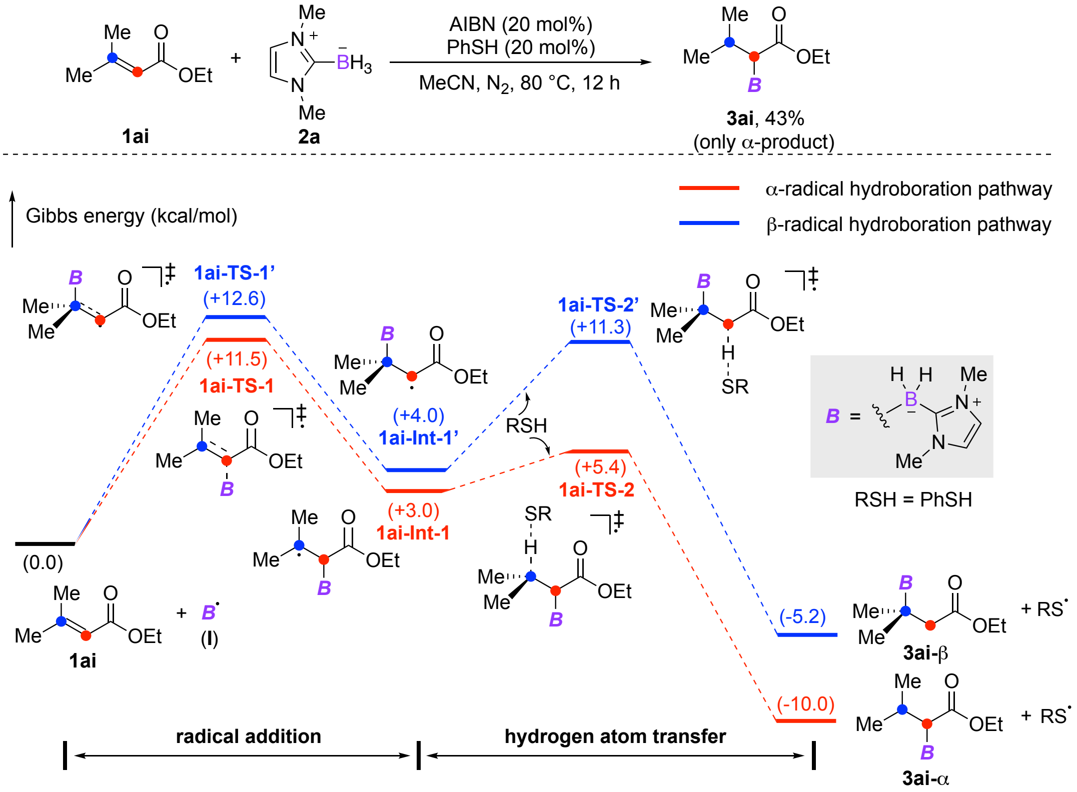

Supplementary Figure 1. DFT calculations of radical borylation of **1ai**

5.2 DFT calculations of radical borylation of 1ag

The reaction of **1ag** gave a better *α*-selectivity than that of **1af**. To rationalize such difference, DFT calculations were performed. The free energy profiles of the reaction between **1ag** and NHC-boryl radical are shown in the supplementary figure 2. The β-addition transition state is favored over its α-addition counterpart by 2.4 kcal mol^{-1} and the resulting **1ag-Int-1'** is much stable than **1ag-Int-1** (-2.7 kcal mol^{-1} versus +1.4 kcal mol^{-1}). This is possibly attributed to more stabilization from the amide motif than that from the methyl group. The subsequent HAT from thiol to **1ag-Int-1** undergoes easily with a facile energy barrier of +5.7 kcal mol^{-1} (**1ag-Int-1 →1ag-TS-2**) and an obvious energy decrease of +8.5 kcal mol^{-1} (**1ag-Int-1 → 3ag-*α***). Such an energetically favorable HAT process renders the *α*-addition step irreversible, as well as promotes the *α*-hydroboration pathway vastly. On the other hand, HAT step from thiol to **1ag-Int-1'** is endergonic by 1.5 kcal mol^{-1} (**1ag-Int-1' → 3ag-*β***) and requires an energy barrier of +12.2 kcal mol^{-1} (**1ag-Int-1' → 1ag-TS-2'**). As we mentioned before, this

is most likely due to the more electron withdrawing ability and steric bulkiness of the N-Ts group. The reverse HAT step from **3ag-*β*** to thiyl radical is exergonic by 1.5 kcal mol^{-1} and needs a barrier of only +8.3 kcal mol^{-1}. As a result, the reverse HAT step is much favored and **1ag-Int-1'** prefers to fragment back to **1ag** and NHC-boryl radical, thus accumulating the thermodynamically more favorable *α*-addition product **3ag-*α***.

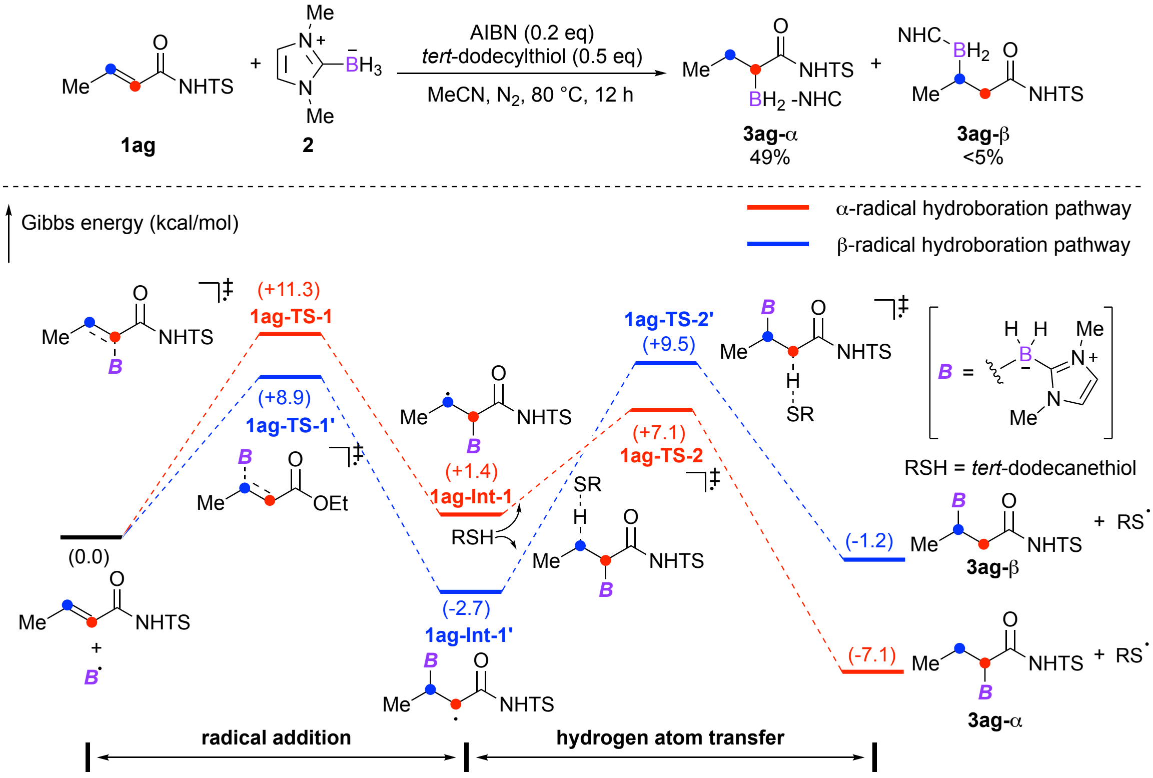

Supplementary Figure 2. DFT calculations of radical borylation of **1ag**

Supplementary Table 6. Thermal correction of Gibbs free energy (TCG, hartree) and total electronic energies (E, hartree) in CH_3CN solvent for all species involved in this study.

Compounds	TCG	E	Compounds	TCG	E
**1a**	0.164379	-576.8810125	**1ai**	0.143547	-424.4331365
**NHC-BH$_2$•**	0.116779	-330.8686518	**1ai-TS-1**	0.281042	-755.2803997
**1a-TS-1**	0.303717	-907.7364471	**1ai-Int-1**	0.282760	-755.2940691
**1a-Int-1**	0.305497	-907.7575858	**1ai-TS-2**	0.371481	-1385.762712
**PhSH**	0.069109	-630.4755875	**3ai**	0.299044	-755.9359891
**1a-TS-2**	0.392298	-1538.218347	**1ai-TS-1'**	0.282209	-755.2786687
**3a**	0.318940	-908.3873299	**1ai-Int-1'**	0.284795	-755.2924061
**PhS•**	0.060110	-629.8542935	**1ai-TS-2'**	0.372432	-1385.753421
**1a-TS-1'**	0.304220	-907.7344025	**3ai-*β***	0.297776	-755.9283952
**1a-Int-1'**	0.305723	-907.7463048	**1ag**	0.176866	-1105.650788

**1a-TS-2'**	0.393135	-1538.207316	**1ag-TS-1**	0.316812	-1436.498483
**3a'**	0.319220	-908.3814806	**1ag-Int-1**	0.317688	-1436.514145
**1af**	0.116903	-385.1297071	**RSH**	0.314842	-871.0269956
**1af-TS-1**	0.254706	-715.9758393	**1ag-TS-2**	0.658059	-2307.529102
**1af-Int-1**	0.255364	-715.9913755	**3ag-*α***	0.334790	-1437.156301
**1af-TS-2**	0.342421	-1346.456026	**1ag-TS-1'**	0.315971	-1436.502292
**3af-*α***	0.269644	-716.6388392	**1ag-Int-1'**	0.320562	-1436.520781
**1af-TS-1'**	0.254440	-715.9796629	**1ag-TS-2'**	0.658432	-2307.525287
**1af-Int-1'**	0.256697	-715.9968428	**3ag-*β***	0.335876	-1437.146872
**1af-TS-2'**	0.344572	-1346.456641	**RS•**	0.3067880	-870.3983985
**3af-*β***	0.271613	-716.6315371			

RSH = *tert*-dodecanethiol
RS• = *tert*-dodecane thiyl radical

6. Kinetic studies

Laser Flash Photolysis Experiments (LFP)

Nanosecond time-resolved transient absorption spectra were recorded using a home-built laser flash photolysis system.[30,31] The third harmonic (355 nm) of a Q-Switched Nd: YAG laser (Dawa-100, Beamtech) was utilized as the excitation source (pulse duration: 8 ns, repetition rate: 10 Hz, pulse energy ~10 mJ per pulse). The analyzing light from a 500 W xenon lamp passed through a flow quartz cuvette perpendicularly to the pulsed excitation laser. The optical absorption path length was 10 mm. A monochromator equipped with a photomultiplier (CR131, Hamamatsu) was used to record the transient absorption spectra within a wavelength range of 300–800 nm. The typical spectral resolution was less than 1 nm. A dynamic decay curve of the intermediate was averaged by multi-shots and recorded using an oscilloscope (TDS3052B, Tektronix). All the solutions were deoxygenated by purging with high purity argon (99.99%) for more than 20 minutes prior to measurements. The direct cleavage of di-*tert*-butyl peroxide by photolysis at 355 nm produced the *tert*-butoxyl radical, which abstracted the hydrogen of **2a** to form the corresponding NHC-boryl radical **I**.[32]

Transient absorption spectra of the NHC-boryl radical I derived from 2a

A LFP experiment at 355 nm was performed for the solution of **2a** acetonitrile/di-*tert*-butylperoxide. The transient absorption spectra for **I** exhibits two wide

absorptions for λ < 500 nm and λ > 700 nm (Supplementary Figure 3). Although the UV characteristic absorption is just located at ~355 nm, the shoulder at 400 nm was used to represent the concentration of **I** owing to avoiding the Rayleigh scattering of excitation laser. Thus, the corresponding lifetimes (τ) of **I** were determined by fitting the decay curve of the absorption at 400 nm.

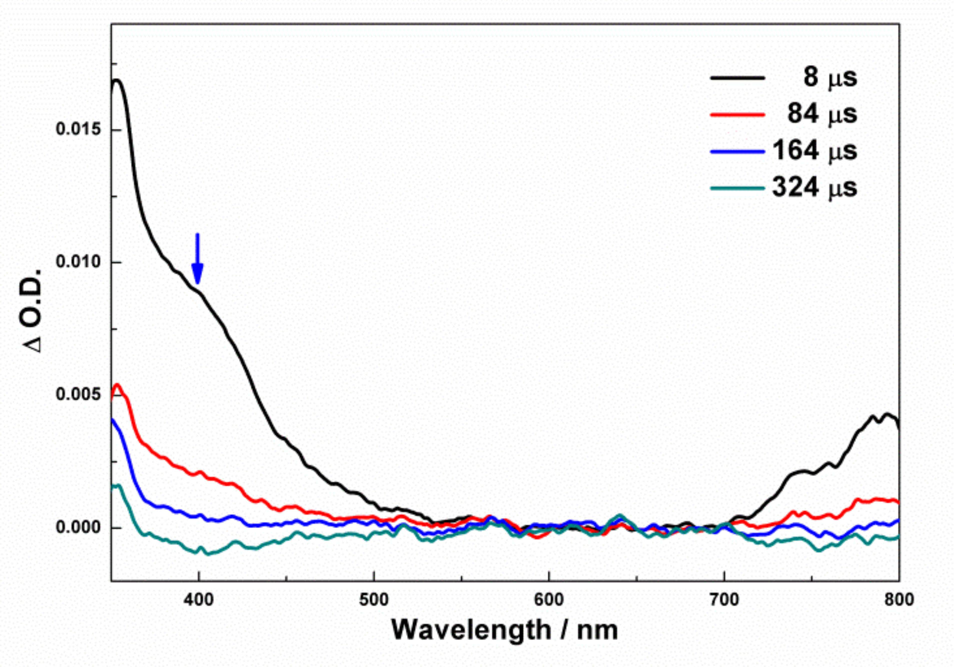

Supplementary figure 3. Transient absorption spectra of the NHC-boryl radical **I**

Rate constants for the bimolecular reactions between boryl radical I with typical substrates

For the different concentrations of typical substrates, the corresponding lifetimes (τ) of **I** were measured respectively. Based on these values, the rate constants for the bimolecular reactions between **I** with typical substrates were determined according to the classical Stern−Volmer equation.

$$\frac{1}{\tau} - \frac{1}{\tau_0} = k_q[\text{substrate}]$$

where τ_0 is the lifetime of **I** in the presence of the substrates, and [substrate] is the concentration.

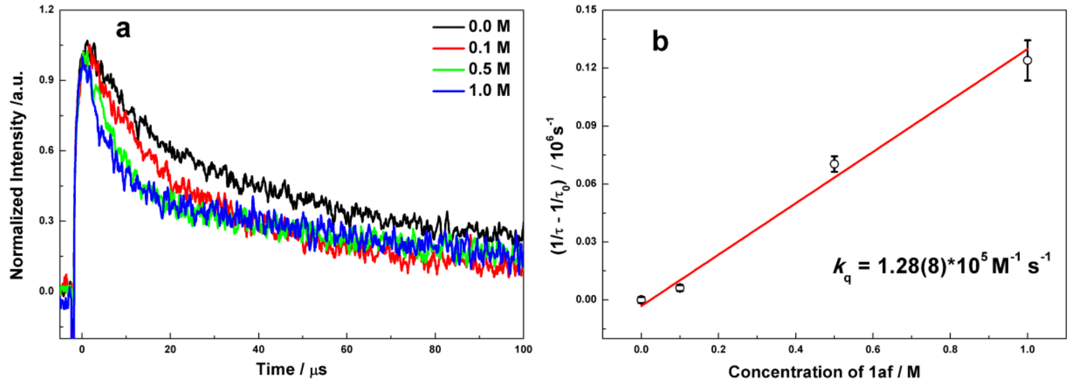

Supplementary figure 4. (a) Decay of **I** at 400 nm with increasing concentrations of **1af** from 0 M to 1 M. (b) Stern−Volmer plots describing the effect of the **1af** concentration on the lifetime quenching of **I**.

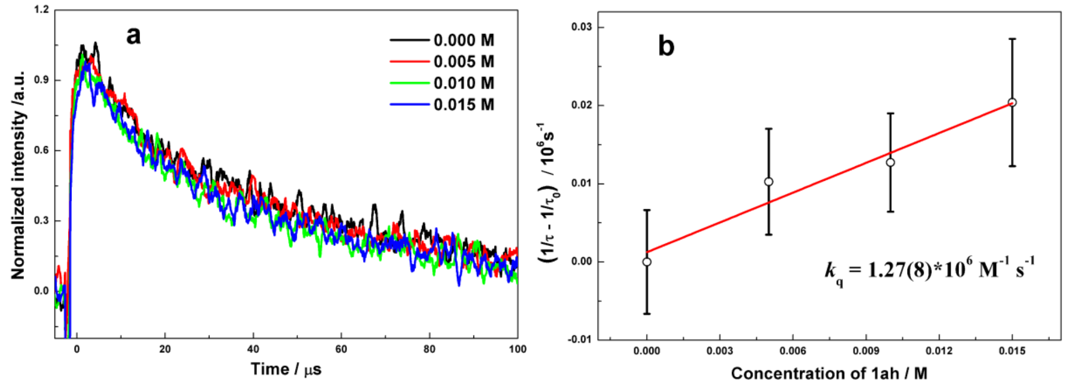

Supplementary figure 5. (a) Decay of **I** at 400 nm with increasing concentrations of **1ah** from 0 M to 1 M. (b) Stern−Volmer plots describing the effect of the **1ah** concentration on the lifetime quenching of **I**.

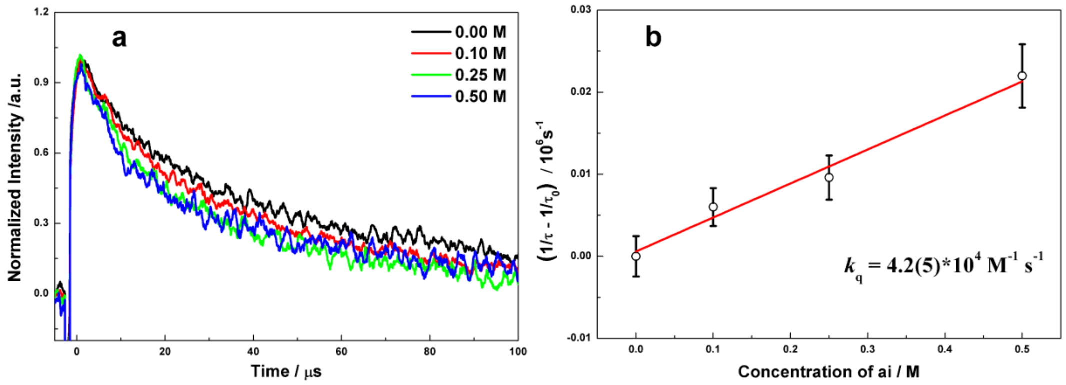

Supplementary figure 6. (a) Decay of **I** at 400 nm with increasing concentrations of **1ai** from 0 M to 1 M. (b) Stern−Volmer plots describing the effect of the **1ai** concentration on the lifetime quenching of **I**.

Activation parameters for the addition of I to 1a

At the different temperatures from 1 to 40°C, the rate constants for the bimolecular reactions between **I** with **1a** were determined respectively, and summarized in Supplementary Table 7.

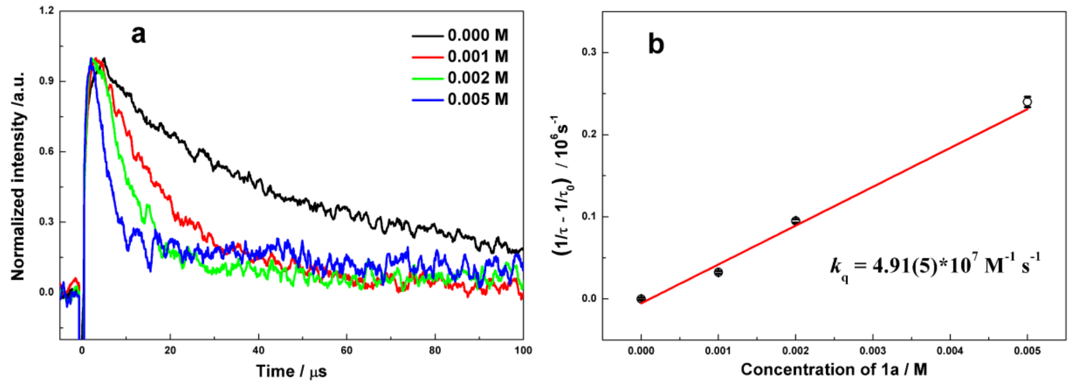

Supplementary figure 7. (**a**) Decay of **I** at 400 nm at 1 °C with increasing concentrations of **1a** from 0 M to 0.005 M. (**b**) Stern−Volmer plots generated from the fitted lifetime of **I** in the presence of **1a** in different concentrations at 1 °C.

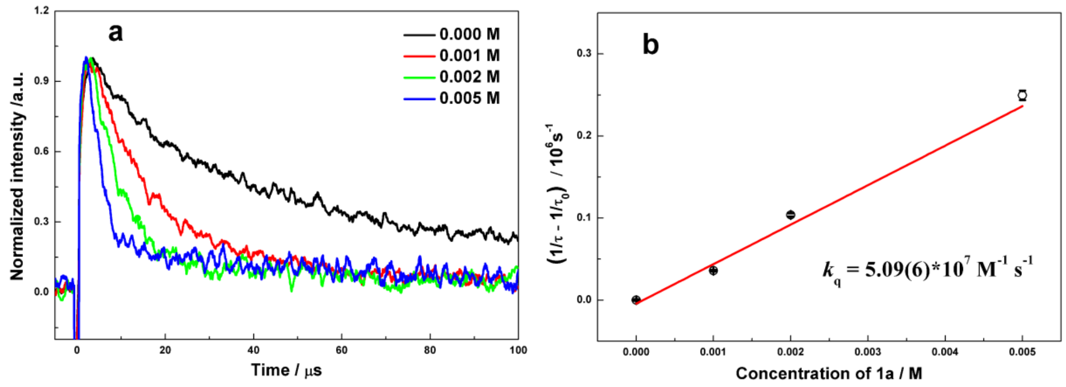

Supplementary figure 8. (**a**) Decay of **I** at 400 nm at 15 °C with increasing concentrations of **1a** from 0 M to 0.005 M. (**b**) Stern−Volmer plots generated from the fitted lifetime of **I** in the presence of **1a** in different concentrations at 15 °C.

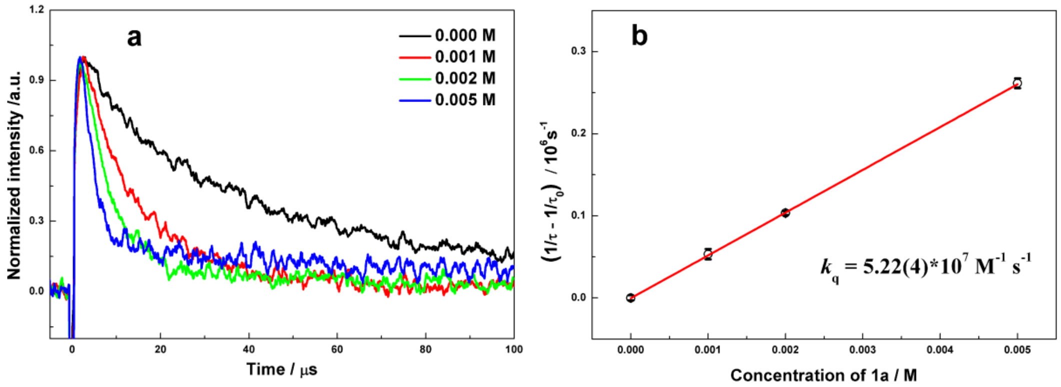

Supplementary figure 9. (**a**) Decay of **I** at 400 nm at 25 °C with increasing concentrations of **1a** from 0 M to 0.005 M. (**b**) Stern−Volmer plots generated from the fitted lifetime of **I** in the presence of **1a** in different concentrations at 25 °C.

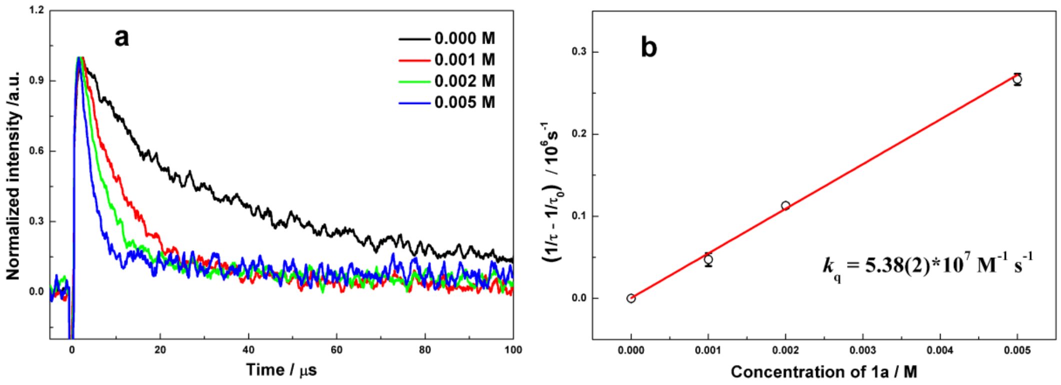

Supplementary figure 10. (**a**) Decay of **I** at 400 nm at 40 °C with increasing concentrations of **1a** from 0 M to 0.005 M. (**b**) Stern−Volmer plots generated from the fitted lifetime of **I** in the presence of **1a** in different concentrations at 40 °C.

Supplementary Table 7. Rate constants for the bimolecular reactions between **I** with **1a** at various temperatures.

T (K)	1/T (K^{-1})	k_q ($M^{-1}\cdot s^{-1}$)	$\ln(hk_q/k_BT)$
274	0.00365	4.91×10^7	-11.663
288	0.00347	5.09×10^7	-11.677
298	0.00336	5.22×10^7	-11.686
313	0.00319	5.38×10^7	-11.705

Using the transition state theory, the linear relationship between k_q/T with 1/T could be fitted as the following equations.

$$k_q = \frac{k_B T}{h} e^{-\frac{\Delta S^{\neq}}{R}} e^{-\frac{\Delta H^{\neq}}{RT}} \qquad (s1)$$

$$\ln\frac{h\cdot k_q}{k_B\cdot T} = \frac{\Delta S^{\neq}}{R} - \frac{\Delta H^{\neq}}{RT} \qquad \text{(s2)}$$

where *R* (ideal gas constant) = 8.314 J·mol^{-1}·K^{-1}, k_B (Boltzmann constant) = 1.38*10^{-23} J·K^{-1}, *h* (Planck constant) = 6.626*10^{-34} J·s. Supplementary Figure 11 shows the experimental data and the fitted relationship with the equation s2. Thus, the reaction enthalpy, reaction entropy, and Gibbs free energy were determined, $\Delta H^{\neq}$ = -0.18 Kcal·mol^{-1}, $\Delta S^{\neq}$ = -99.7 J·mol^{-1}, and $\Delta G^{\neq}$ = 6.92 Kcal·mol^{-1}.

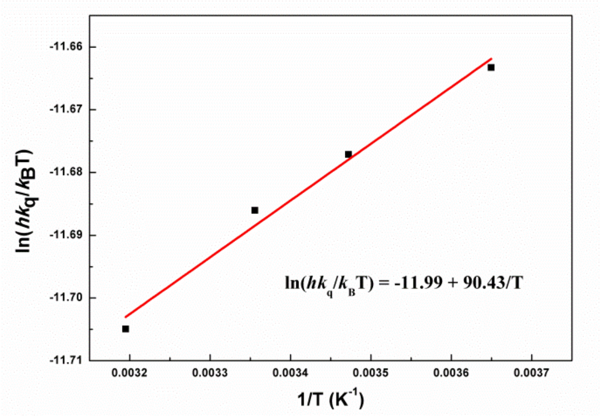

Supplementary Figure 11. Experimental and fitted relationship between k_q/T with 1/T.

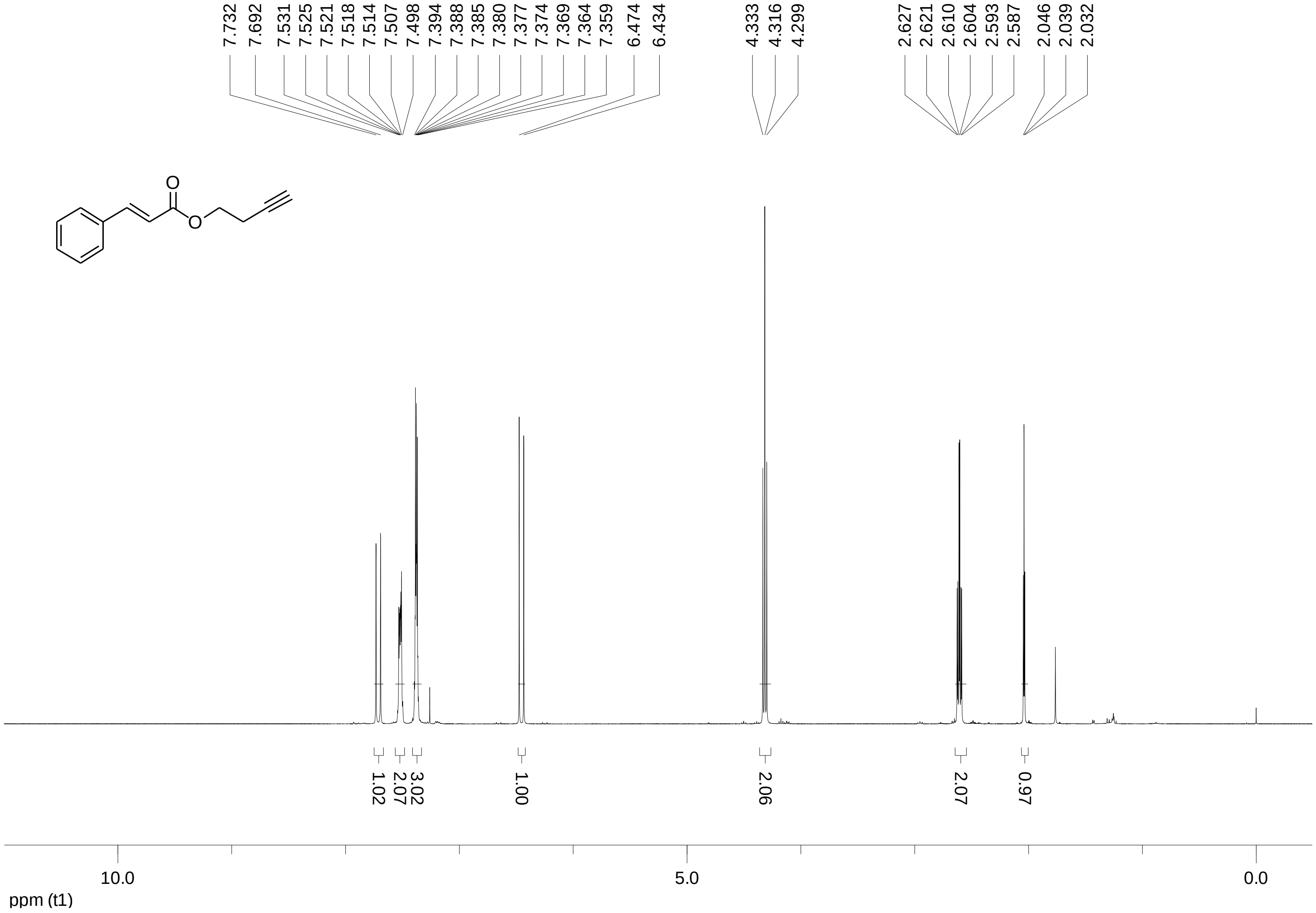

Supplementary Figure 12. ^{1}H NMR spectrum for 1i

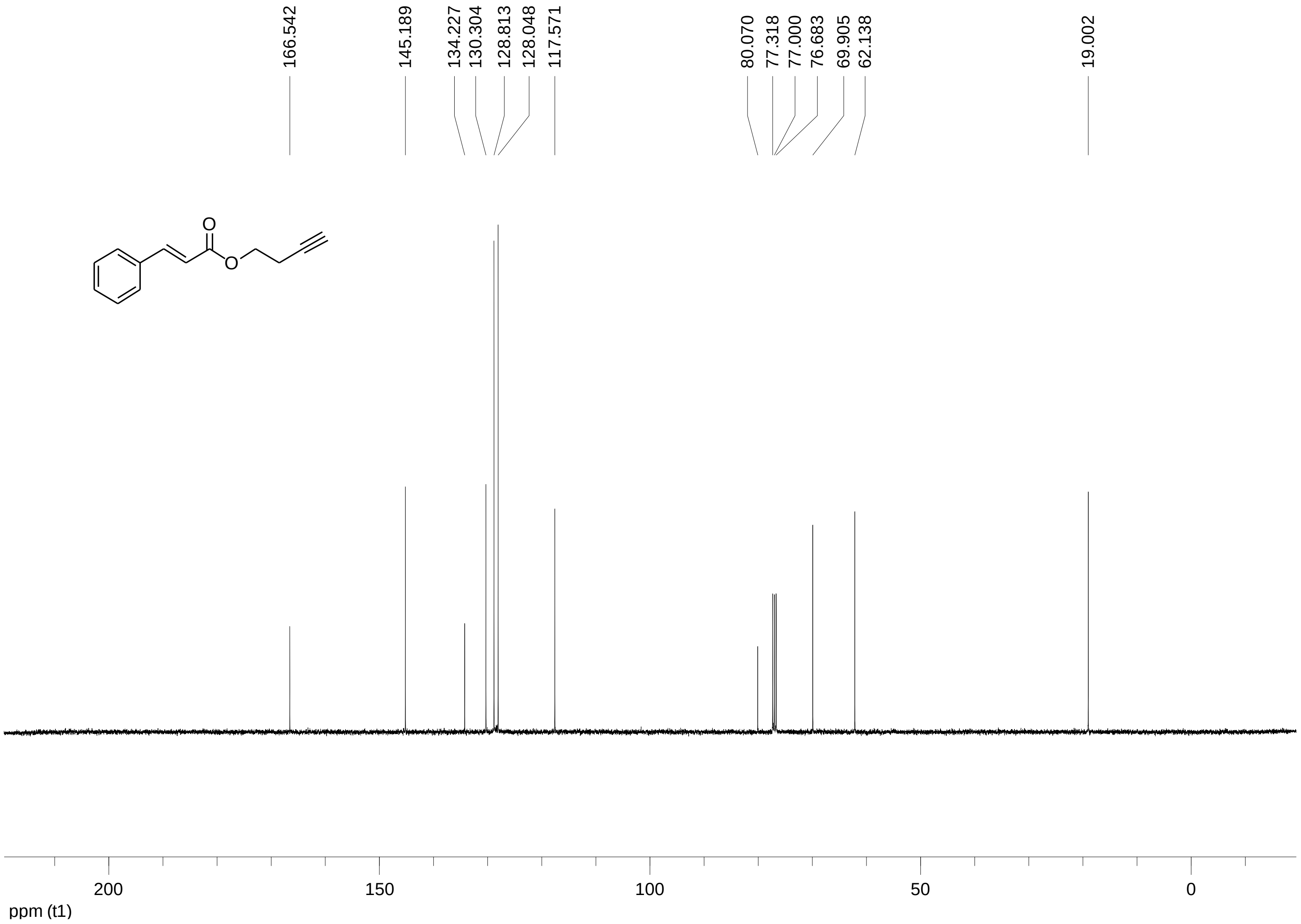

Supplementary Figure 13. ^{13}C NMR spectrum for 1i

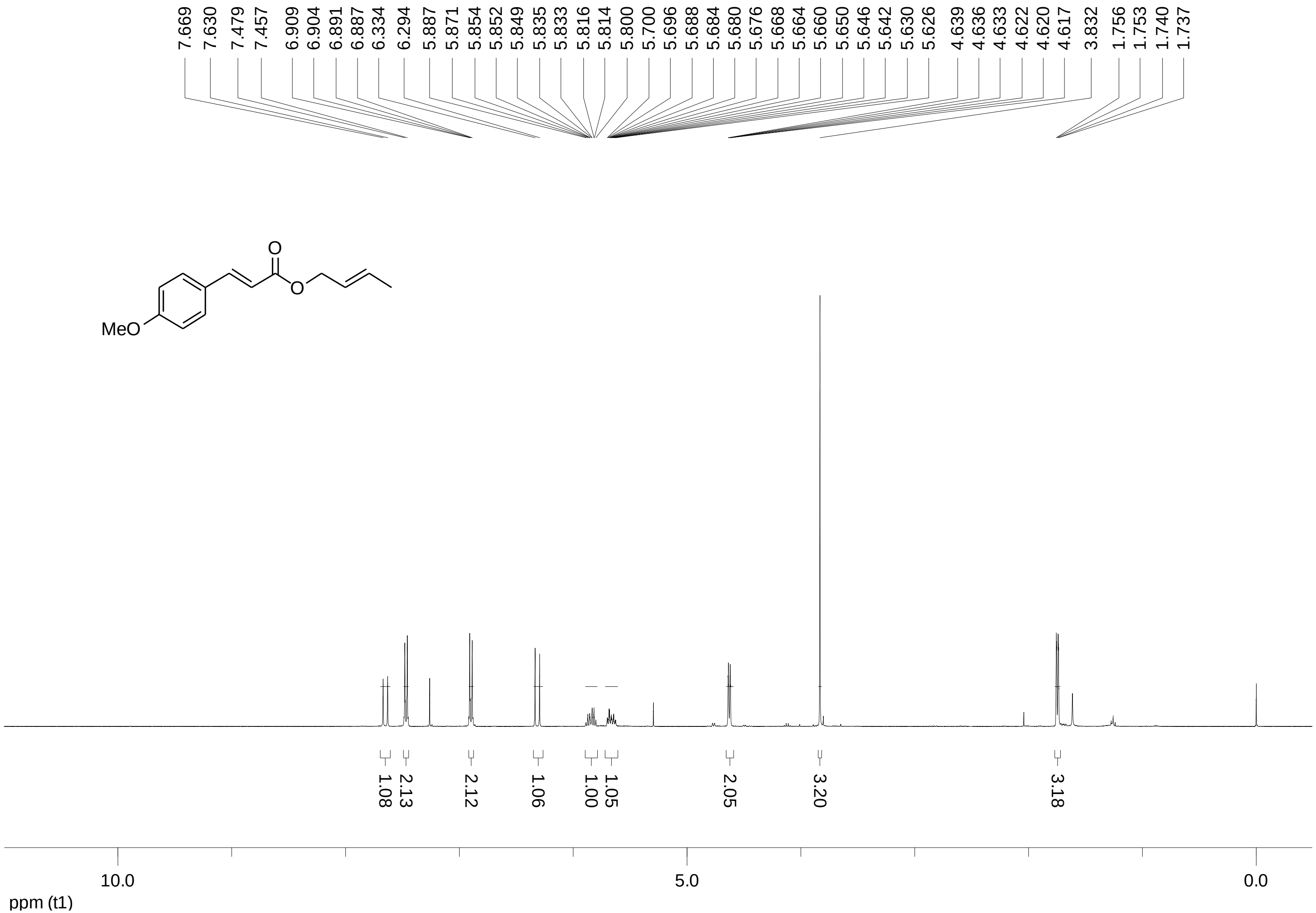

Supplementary Figure 14. ^{1}H NMR spectrum for 1j

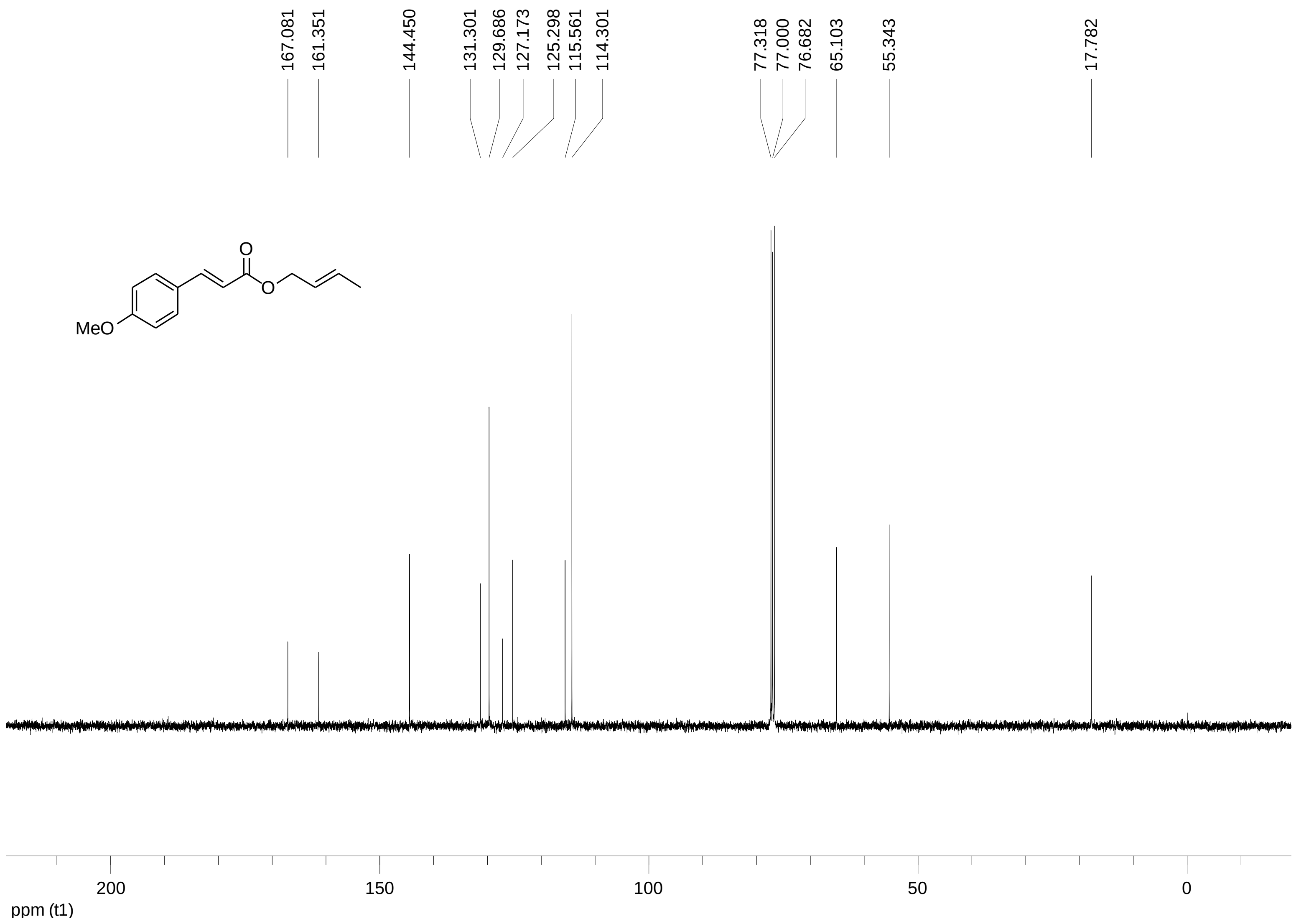

Supplementary Figure 15. ^{13}C NMR spectrum for 1j

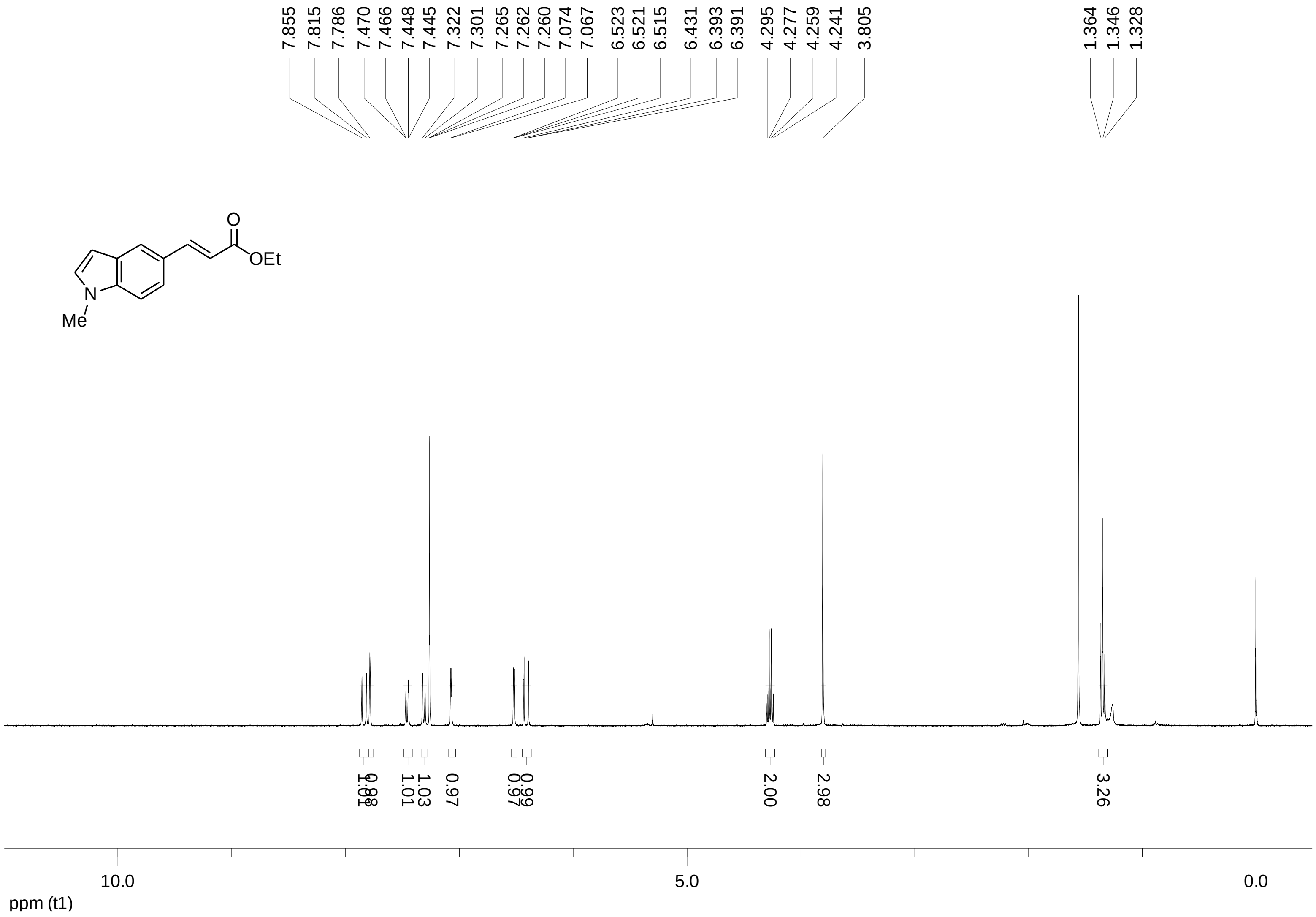

Supplementary Figure 16. ^{1}H NMR spectrum for 1k

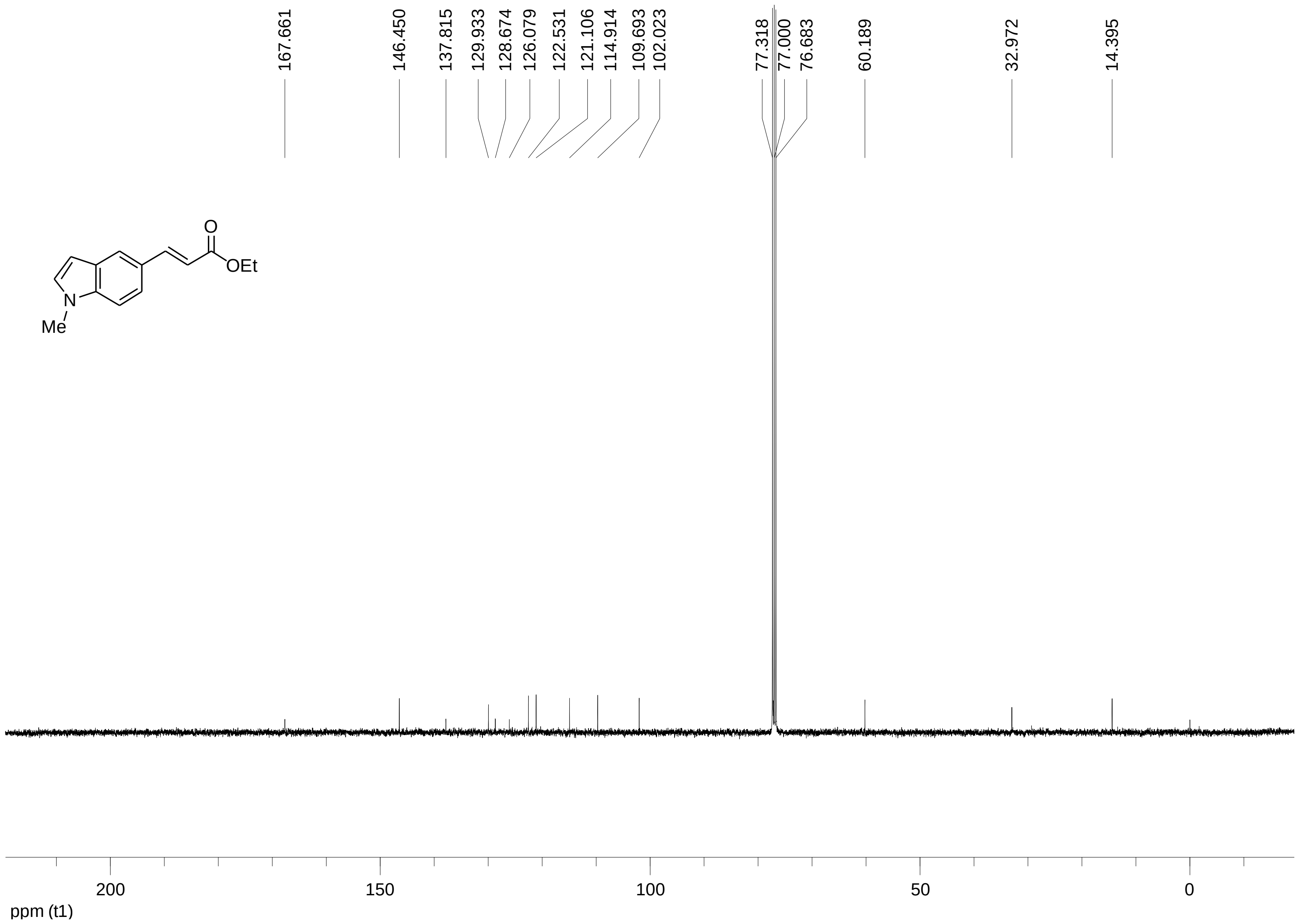

Supplementary Figure 17. ^{13}C NMR spectrum for 1k

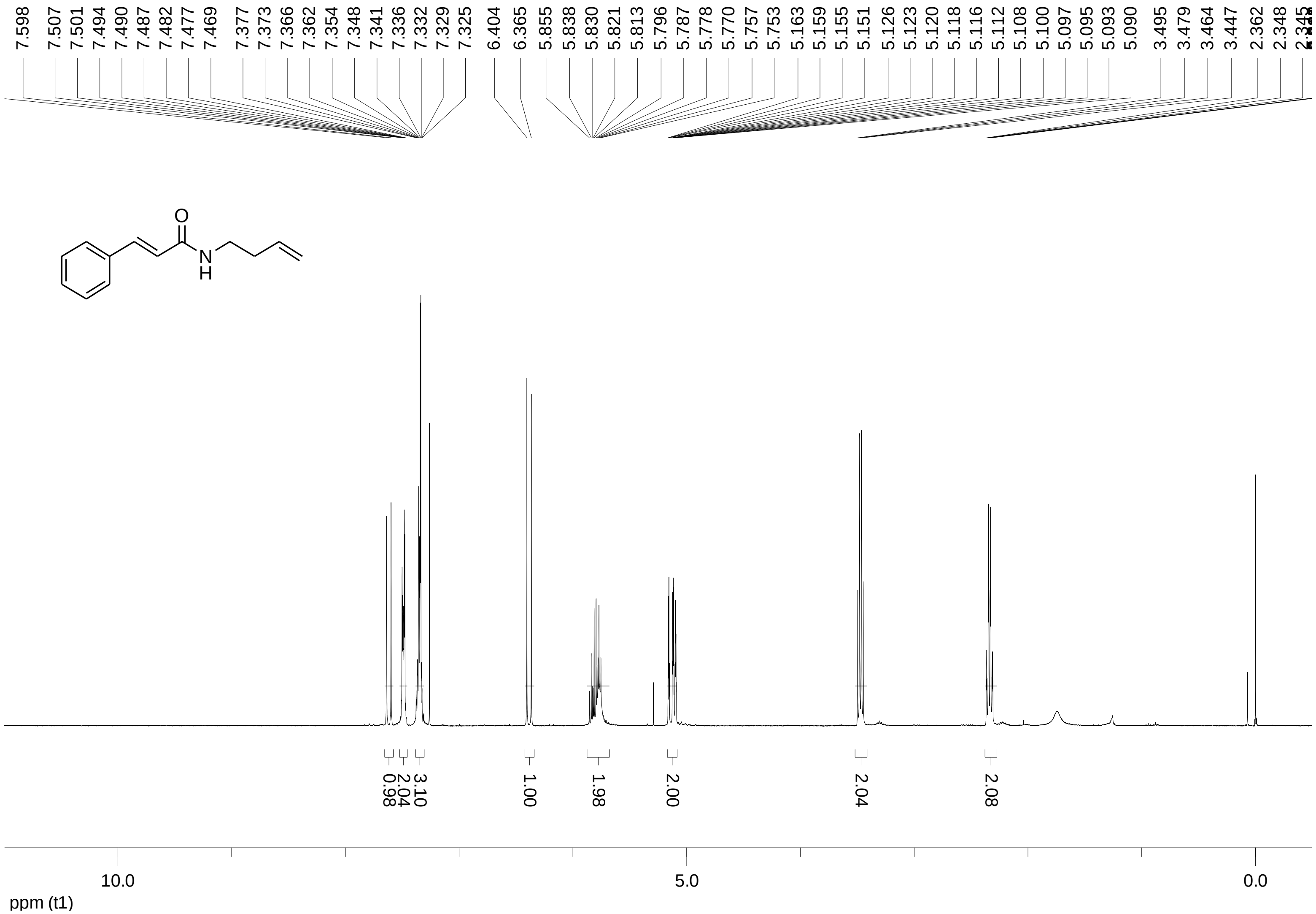

Supplementary Figure 18. ^{1}H NMR spectrum for 1s

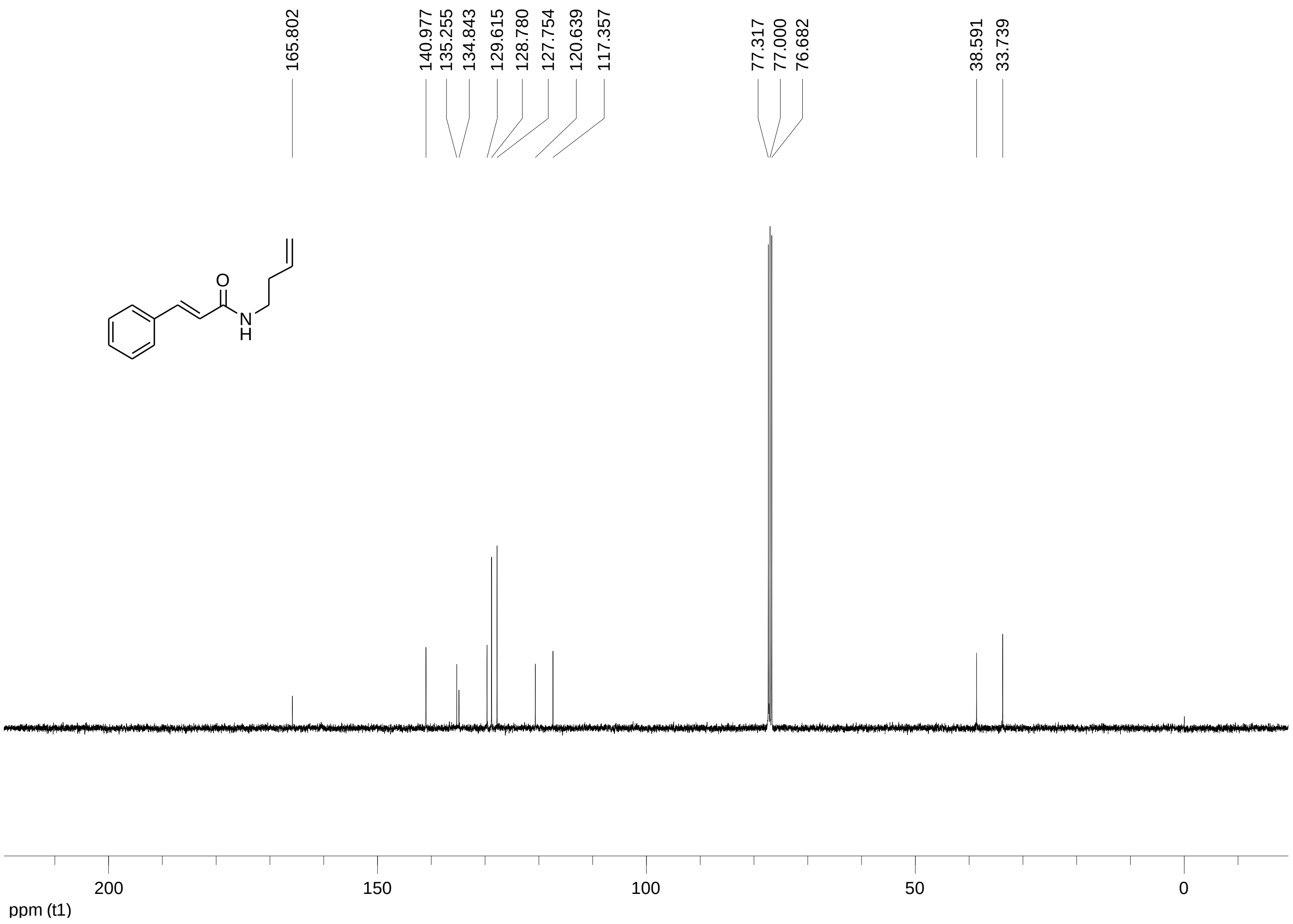

Supplementary Figure 19. ^{13}C NMR spectrum for 1s

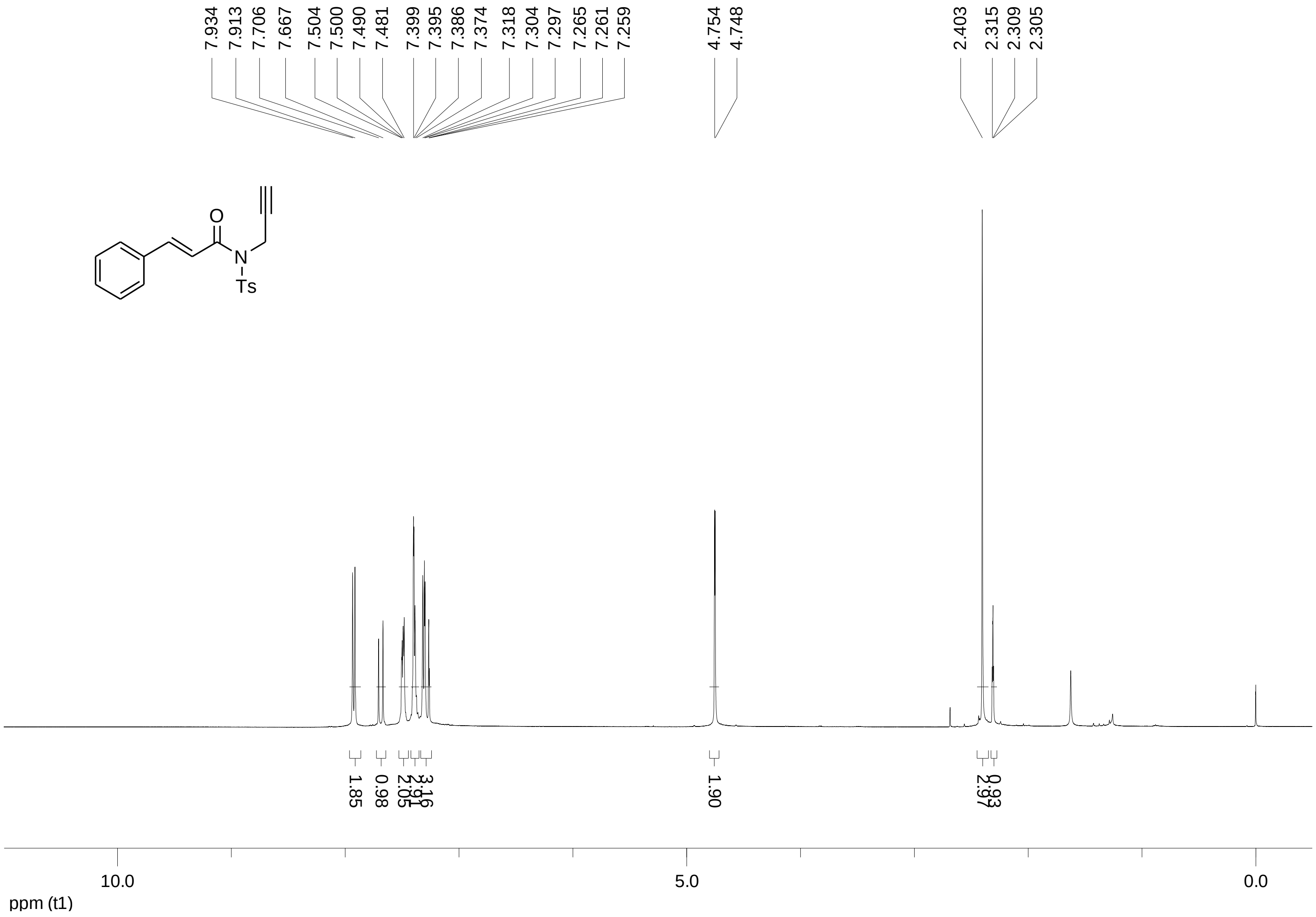

Supplementary Figure 20. ^{1}H NMR spectrum for 1u

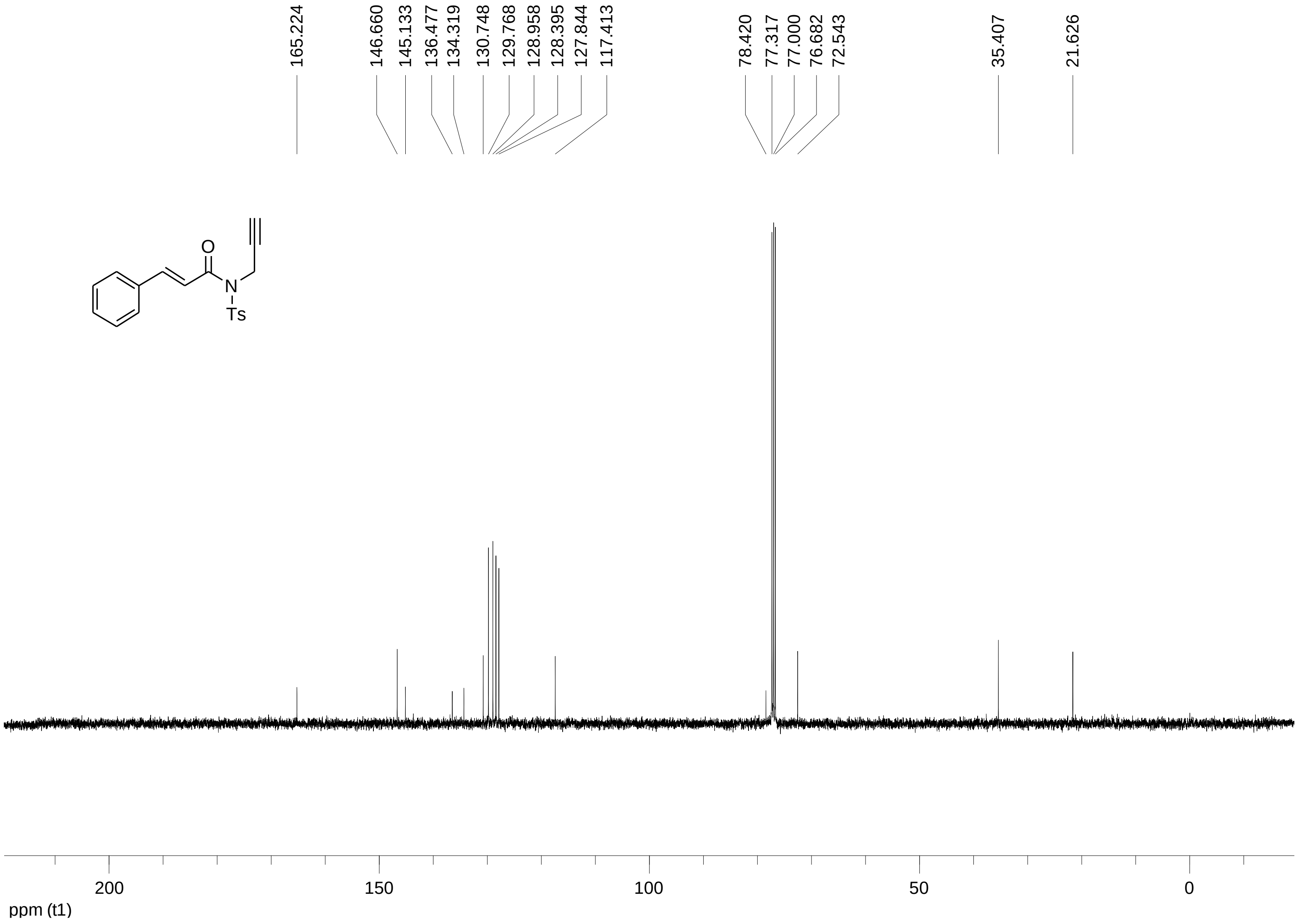

Supplementary Figure 21. ^{13}C NMR spectrum for 1u

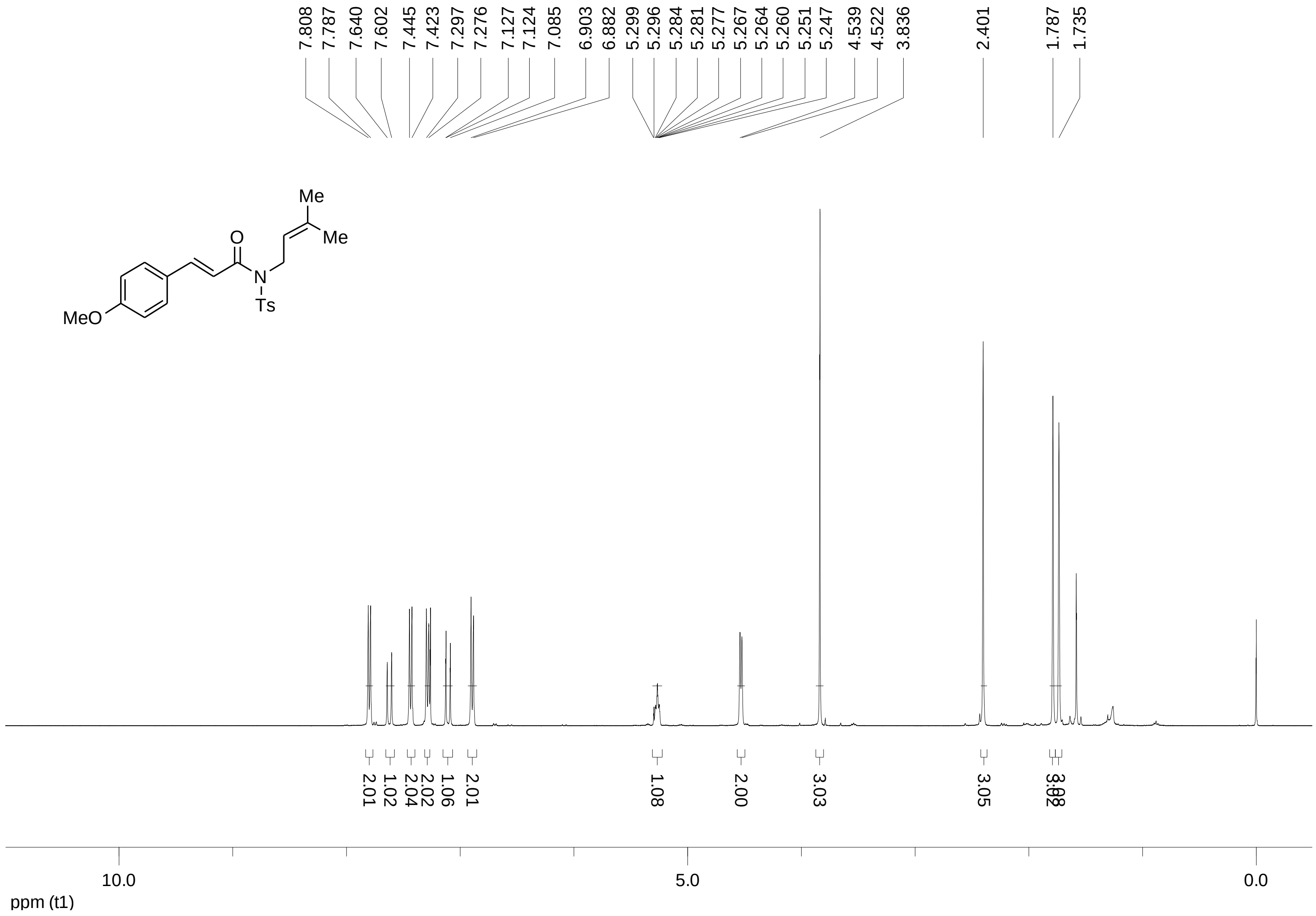

Supplementary Figure 22. ^{1}H NMR spectrum for 1v

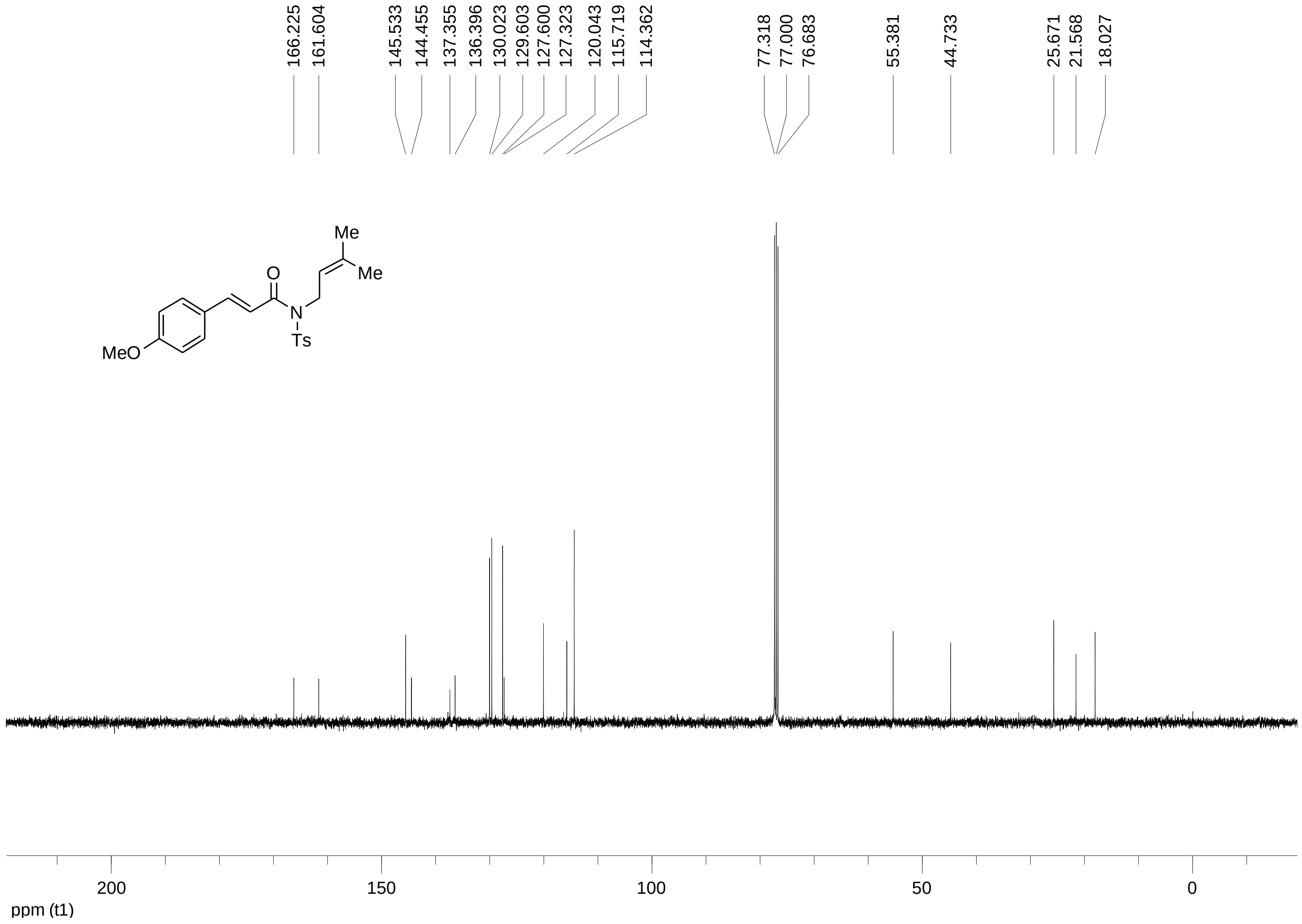

Supplementary Figure 23. ^{13}C NMR spectrum for 1v

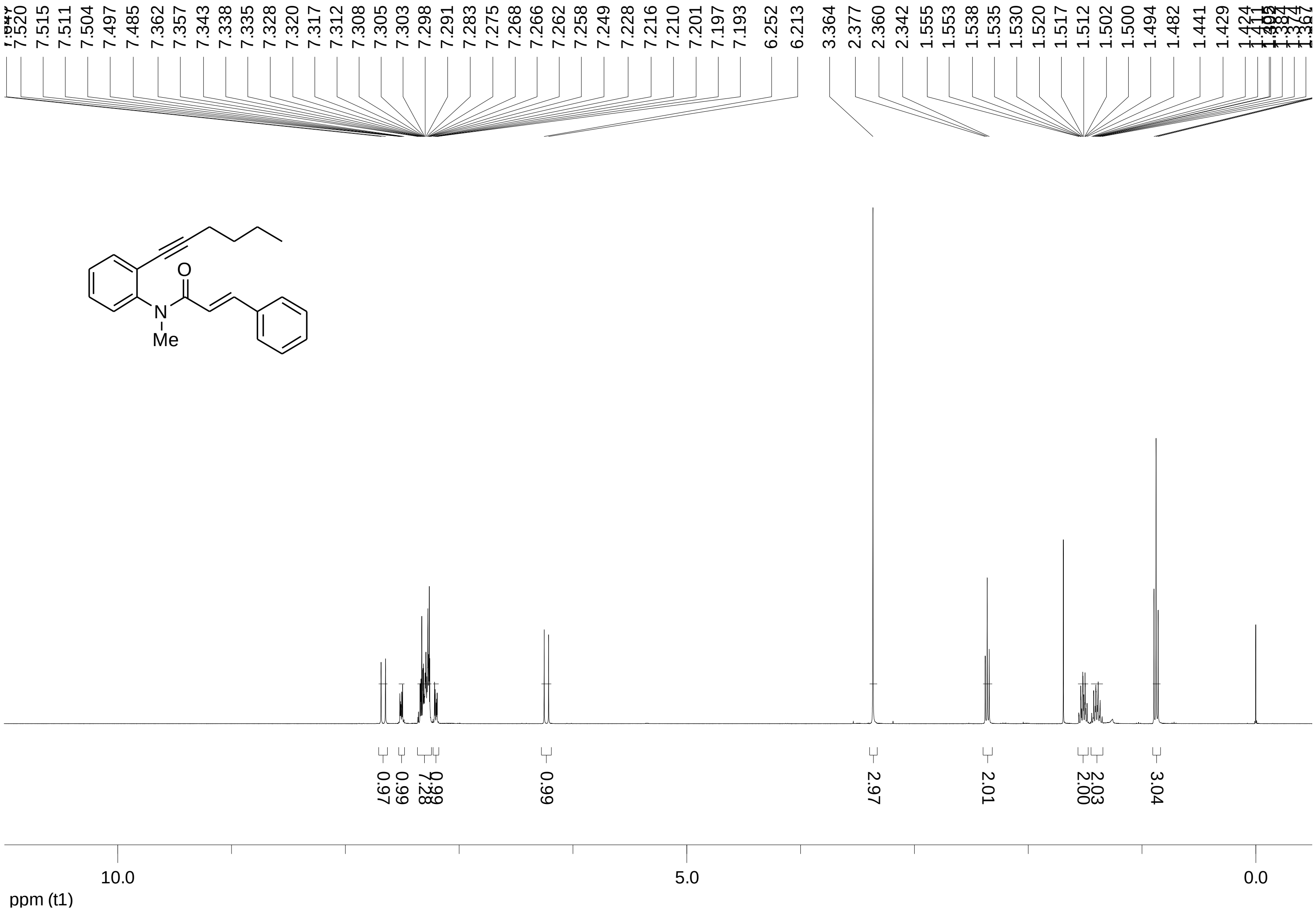

Supplementary Figure 24. ^{1}H NMR spectrum for 1w

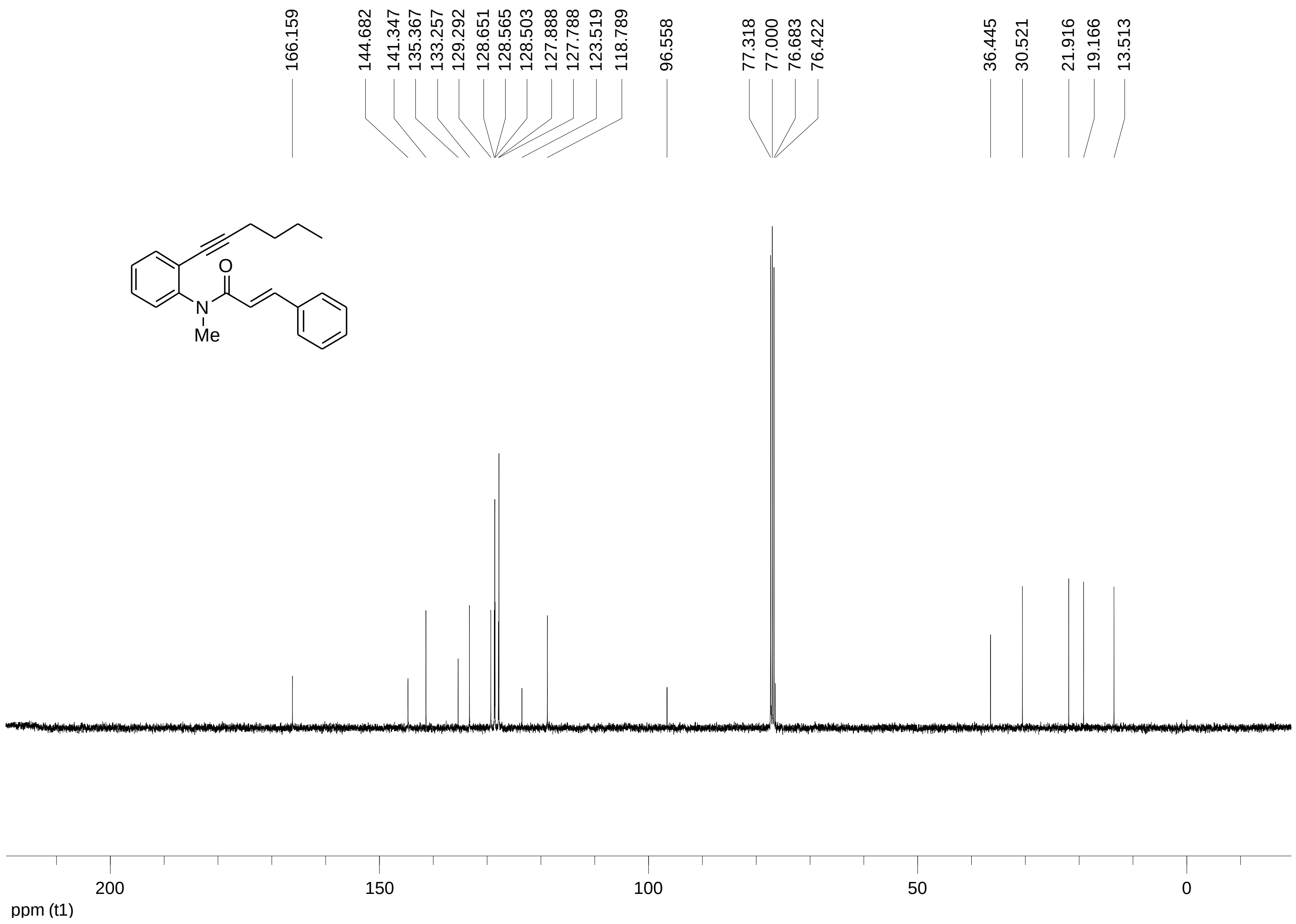

Supplementary Figure 25. ^{13}C NMR spectrum for 1w

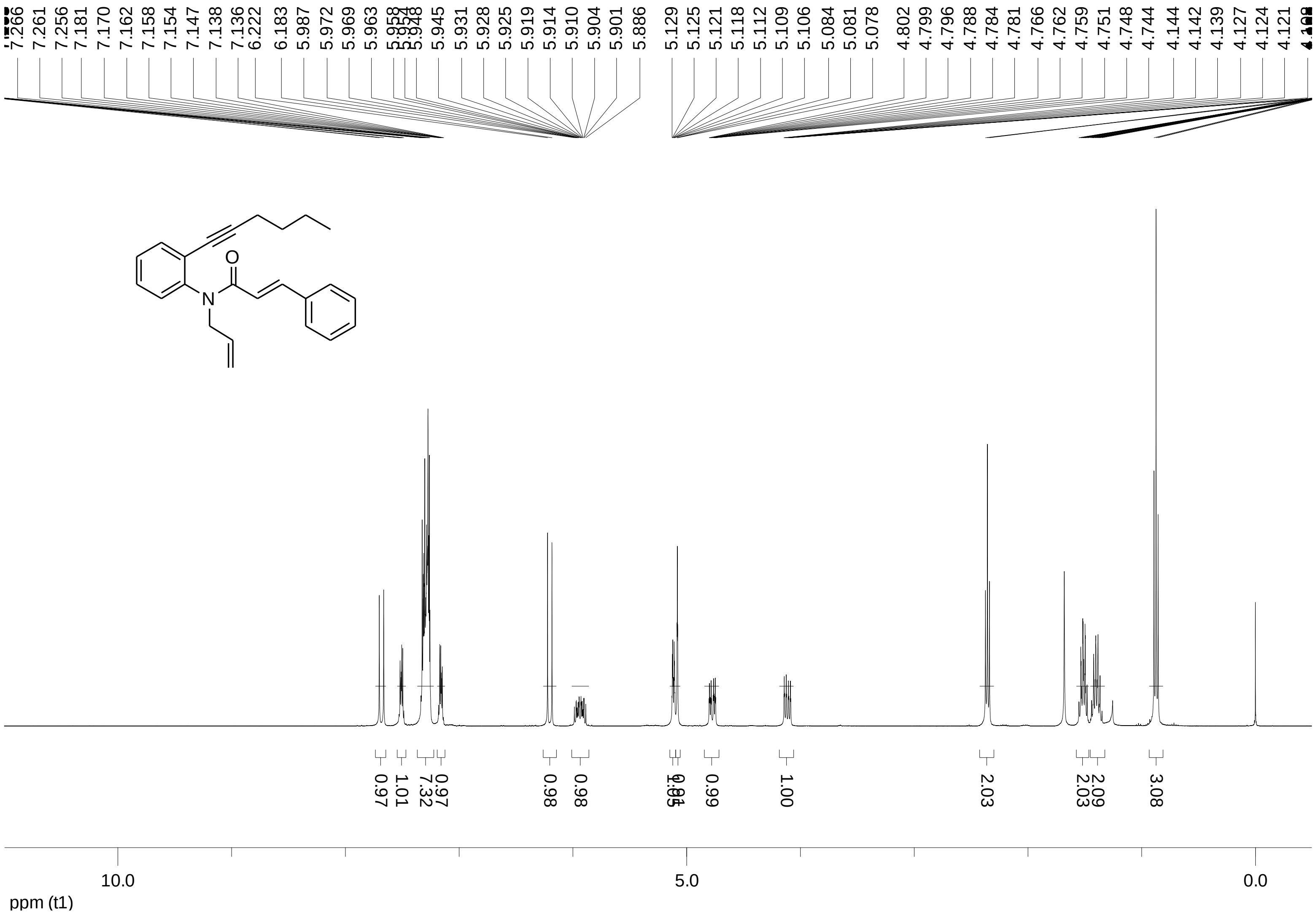

Supplementary Figure 26. ¹H NMR spectrum for 1x

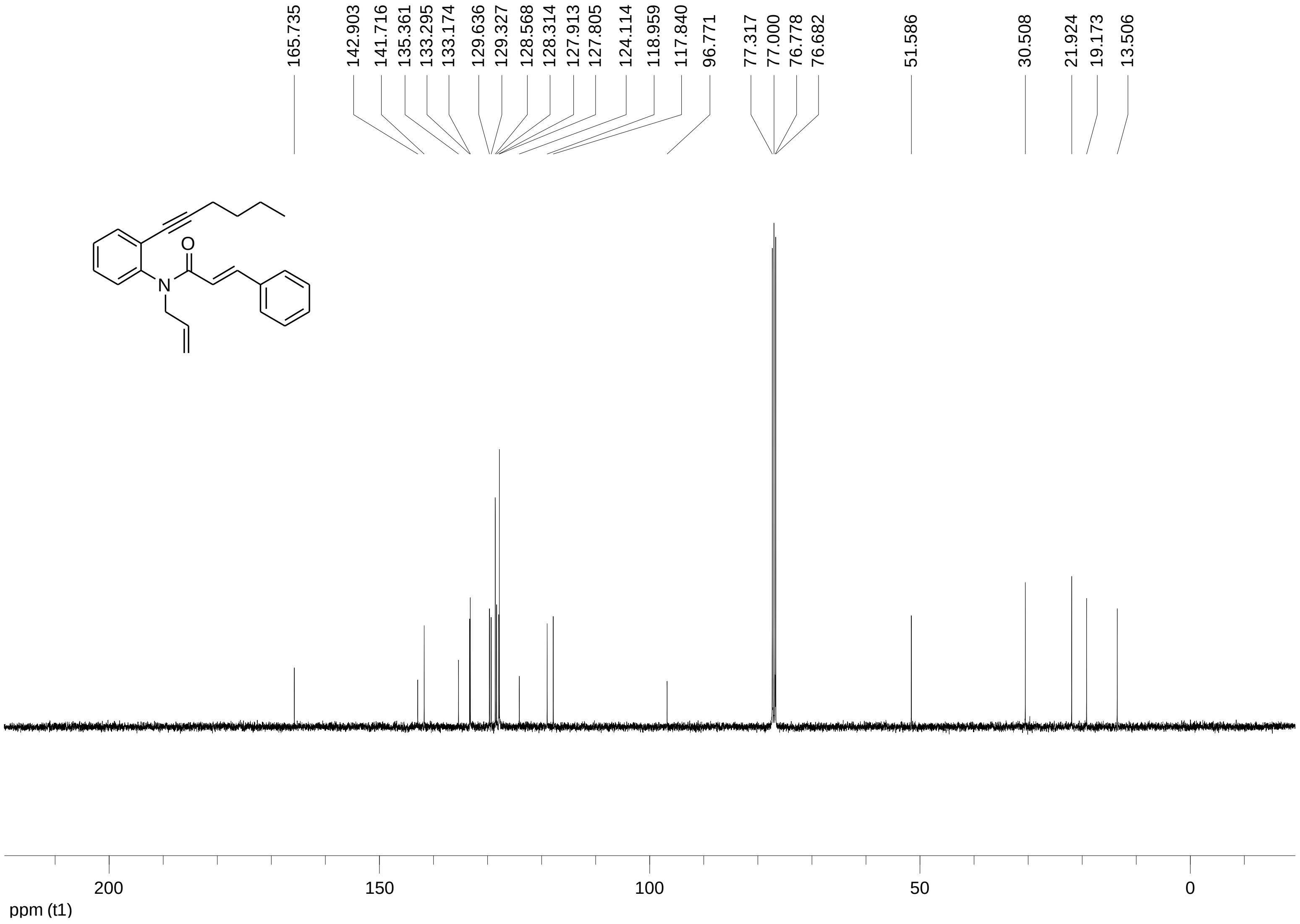

Supplementary Figure 27. ^{13}C NMR spectrum for 1x

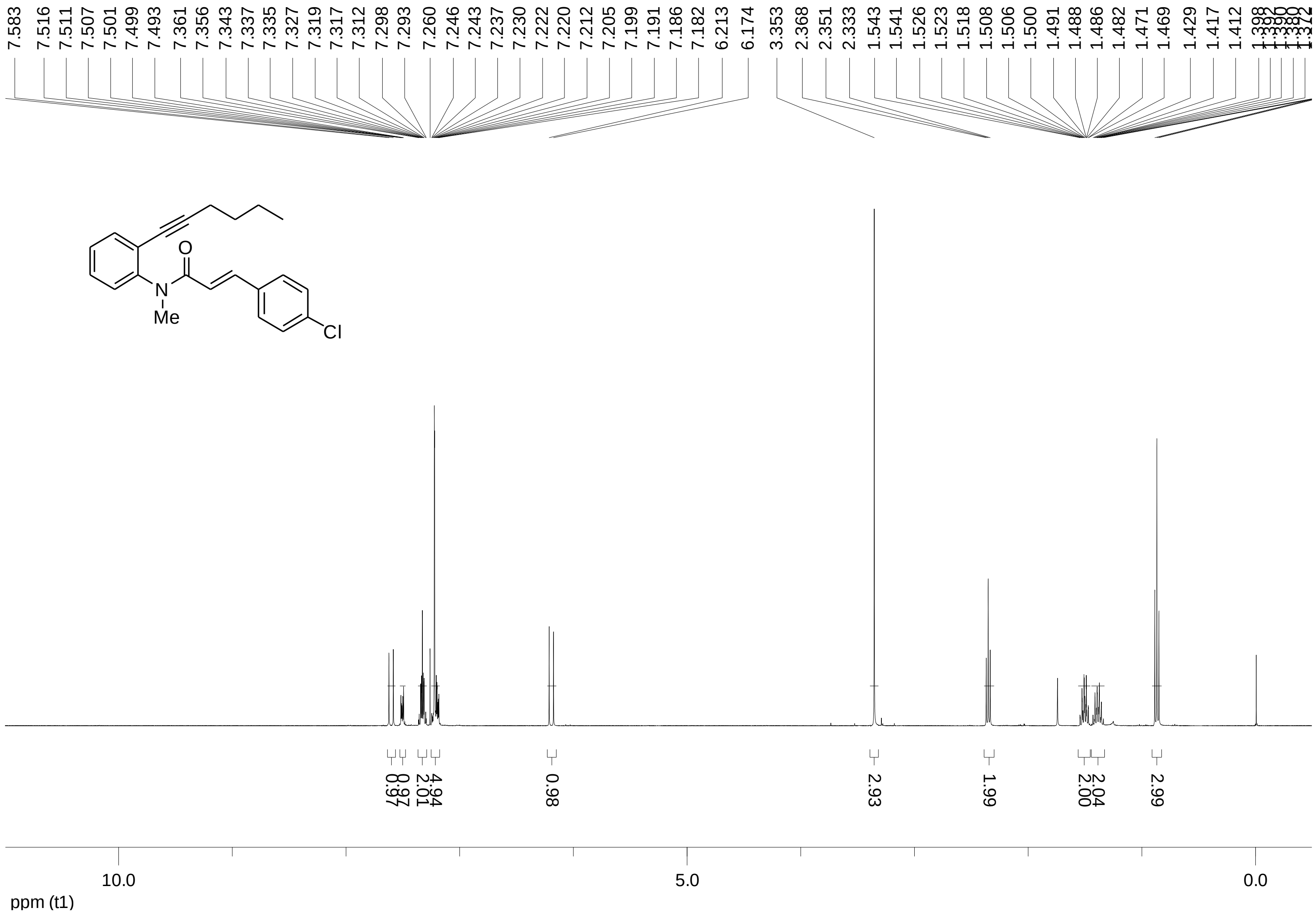

Supplementary Figure 28. ^{1}H NMR spectrum for 1y

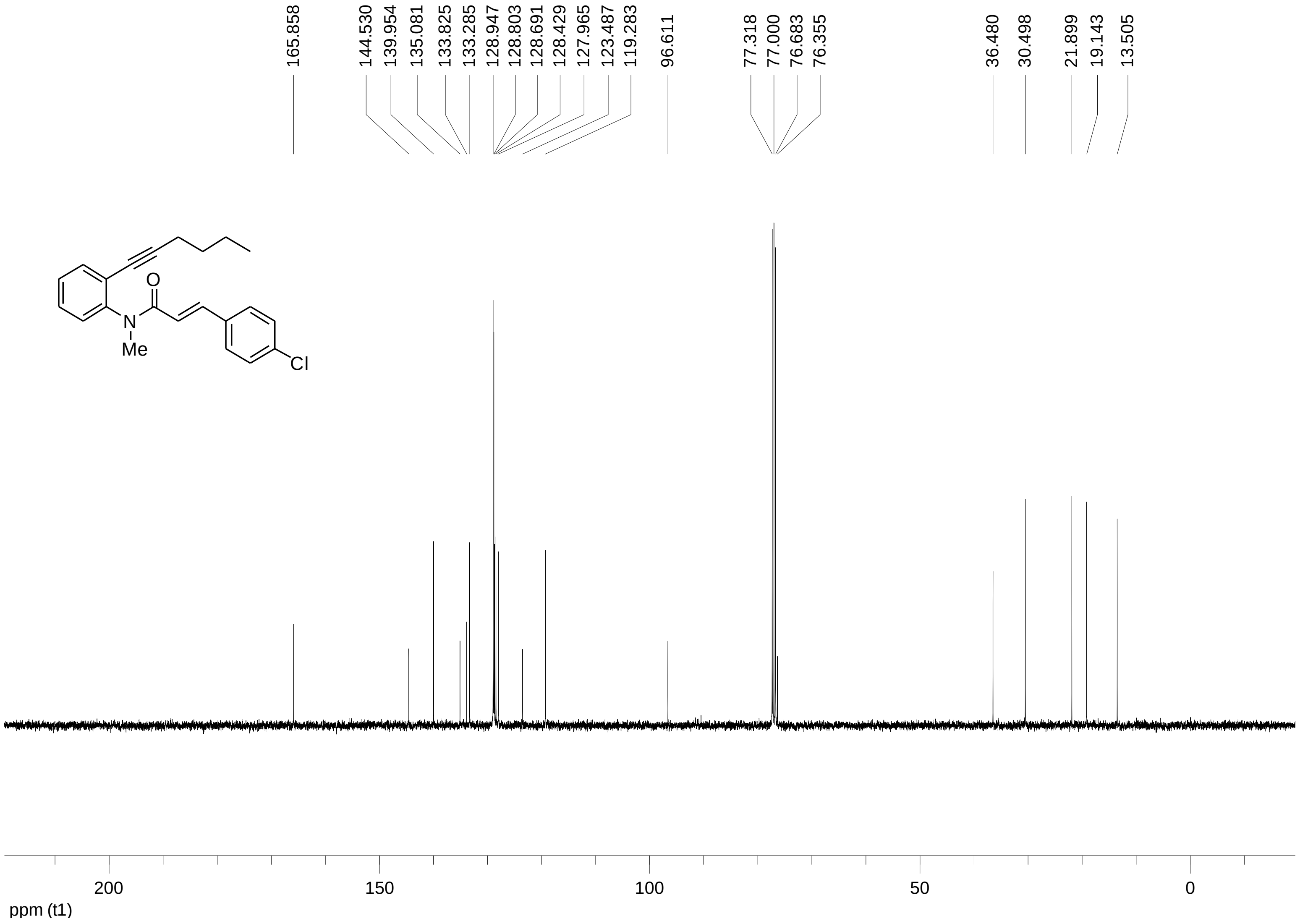

Supplementary Figure 29. ^{13}C NMR spectrum for 1y

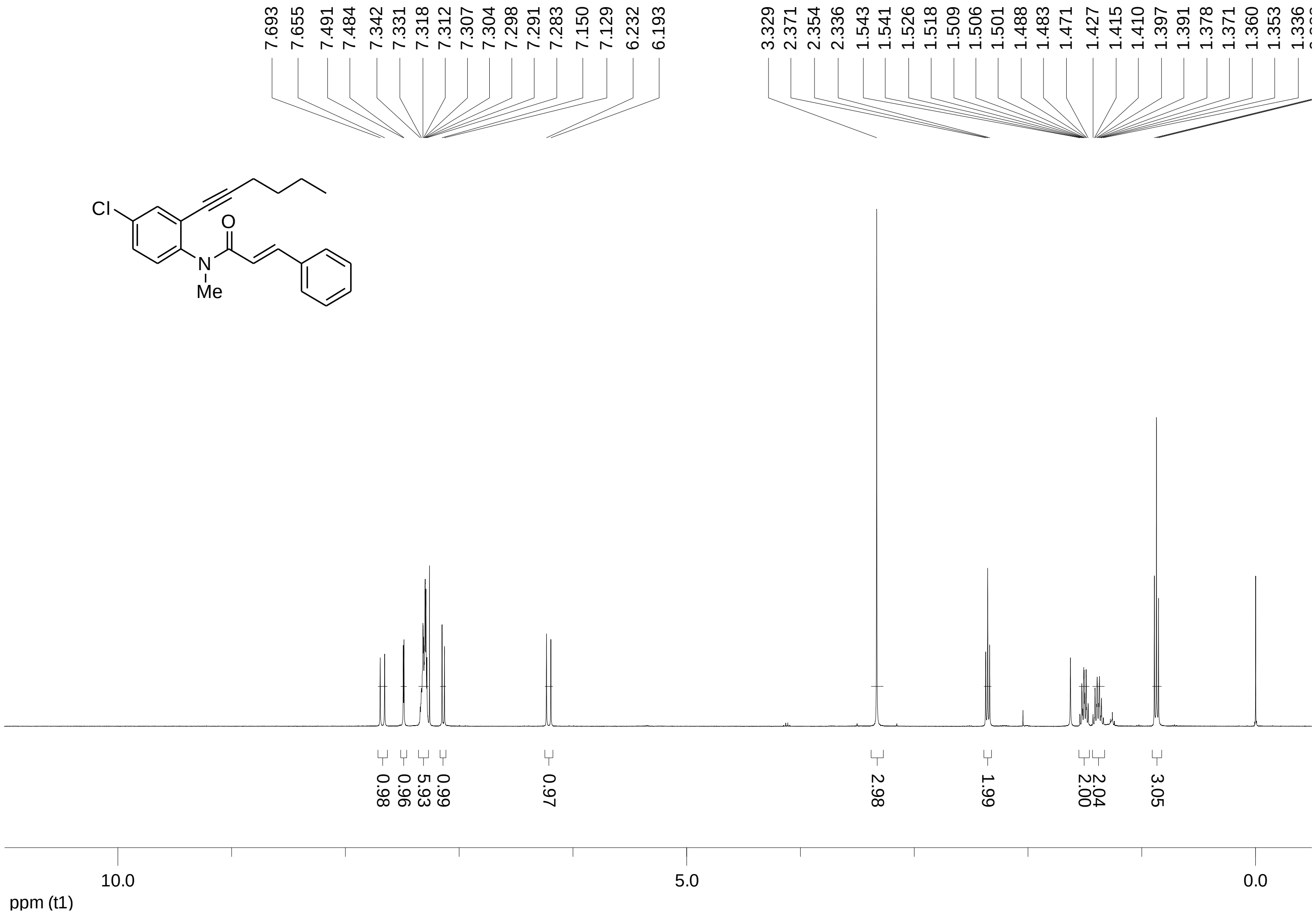

Supplementary Figure 30. ^{1}H NMR spectrum for 1z

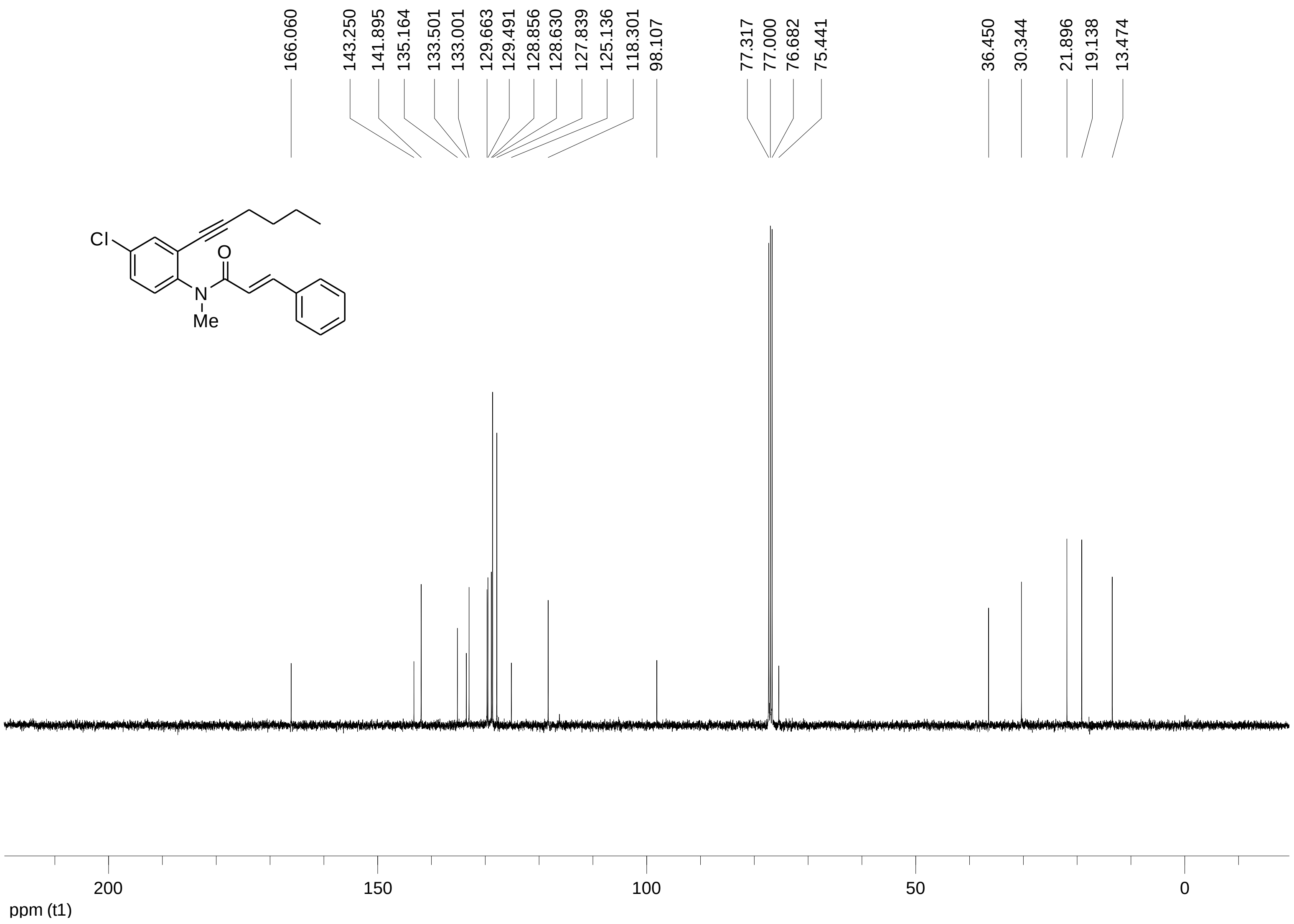

Supplementary Figure 31. ^{13}C NMR spectrum for 1z

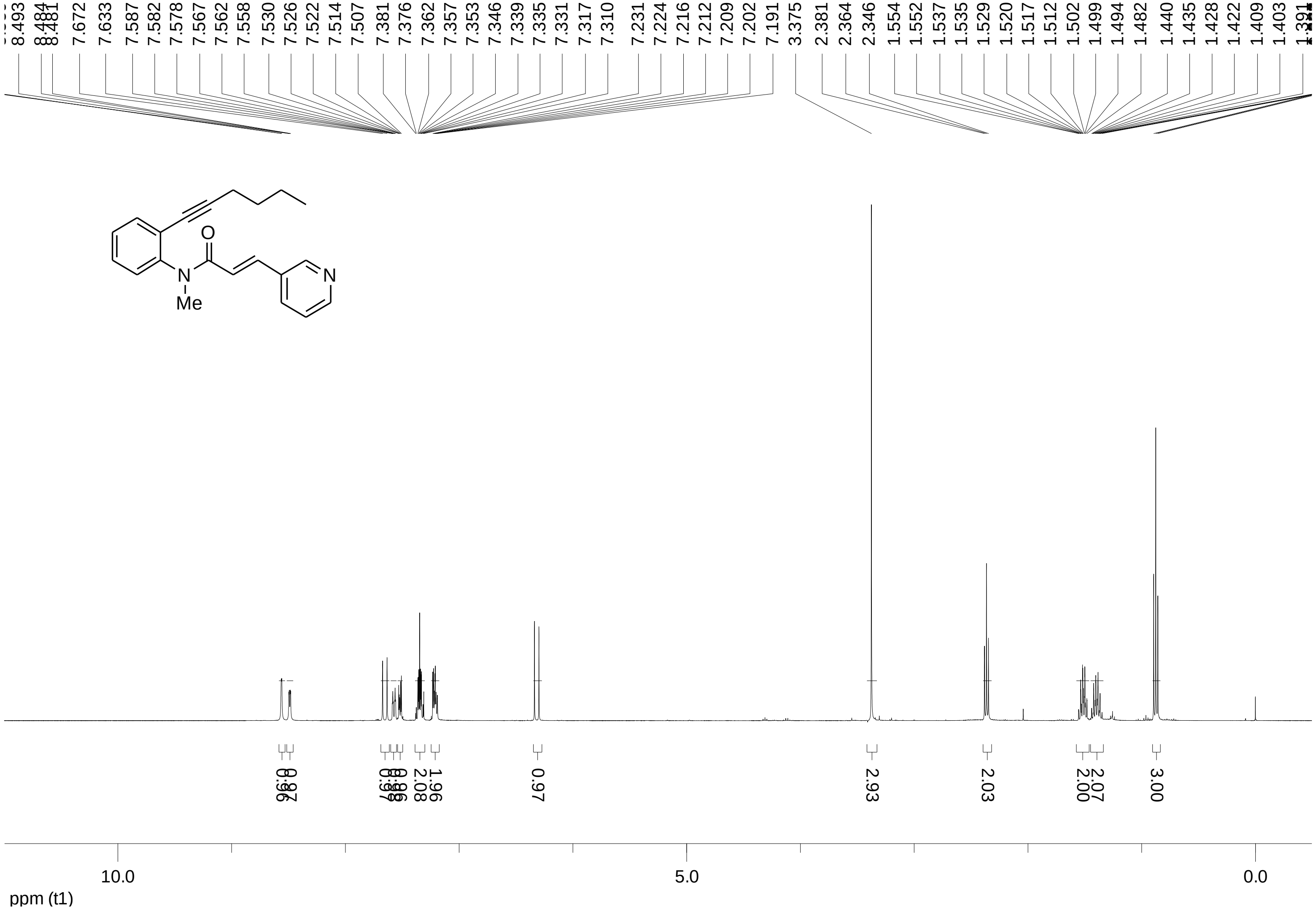

Supplementary Figure 32. ^{1}H NMR spectrum for 1aa

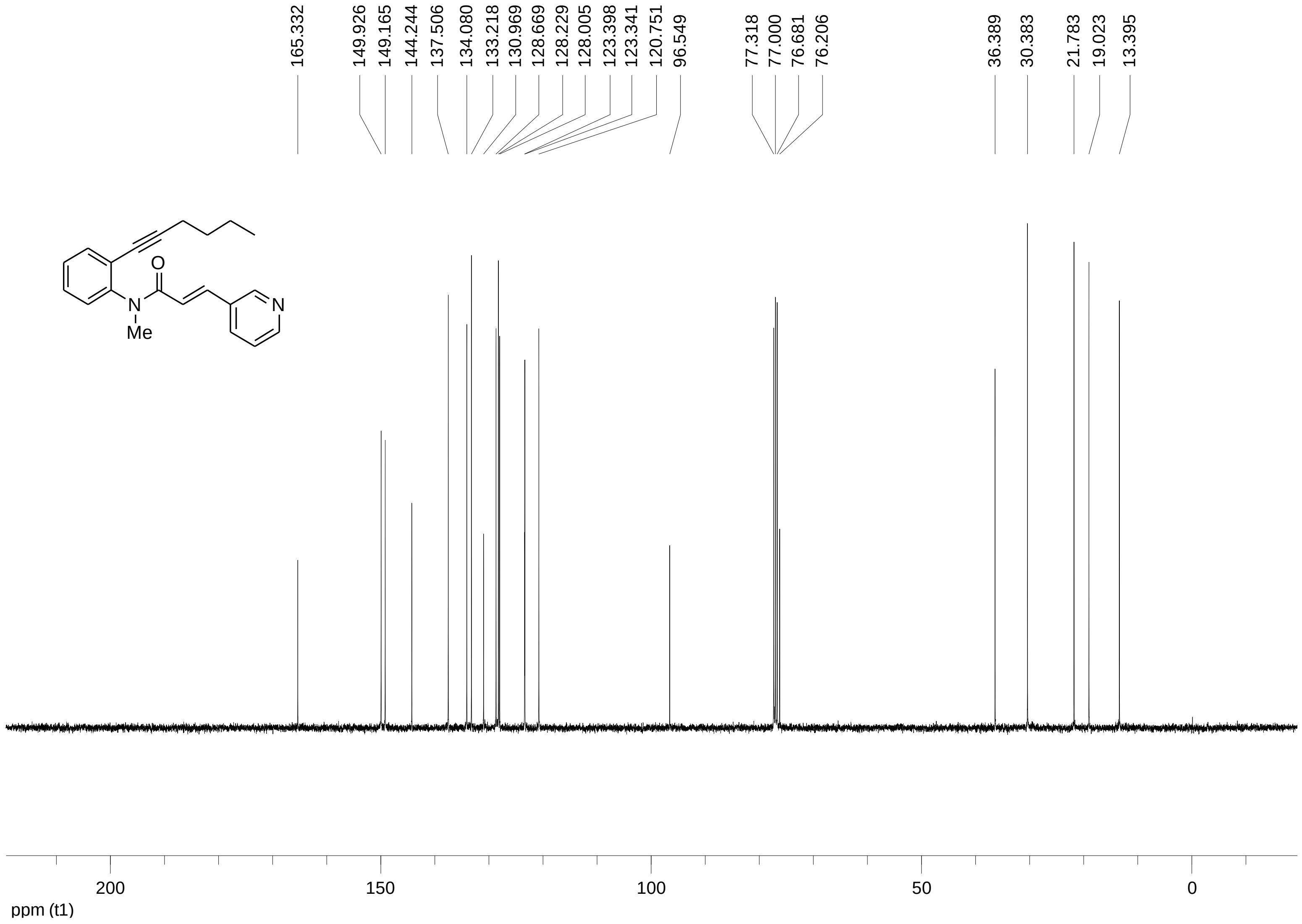

Supplementary Figure 33. ^{13}C NMR spectrum for 1aa

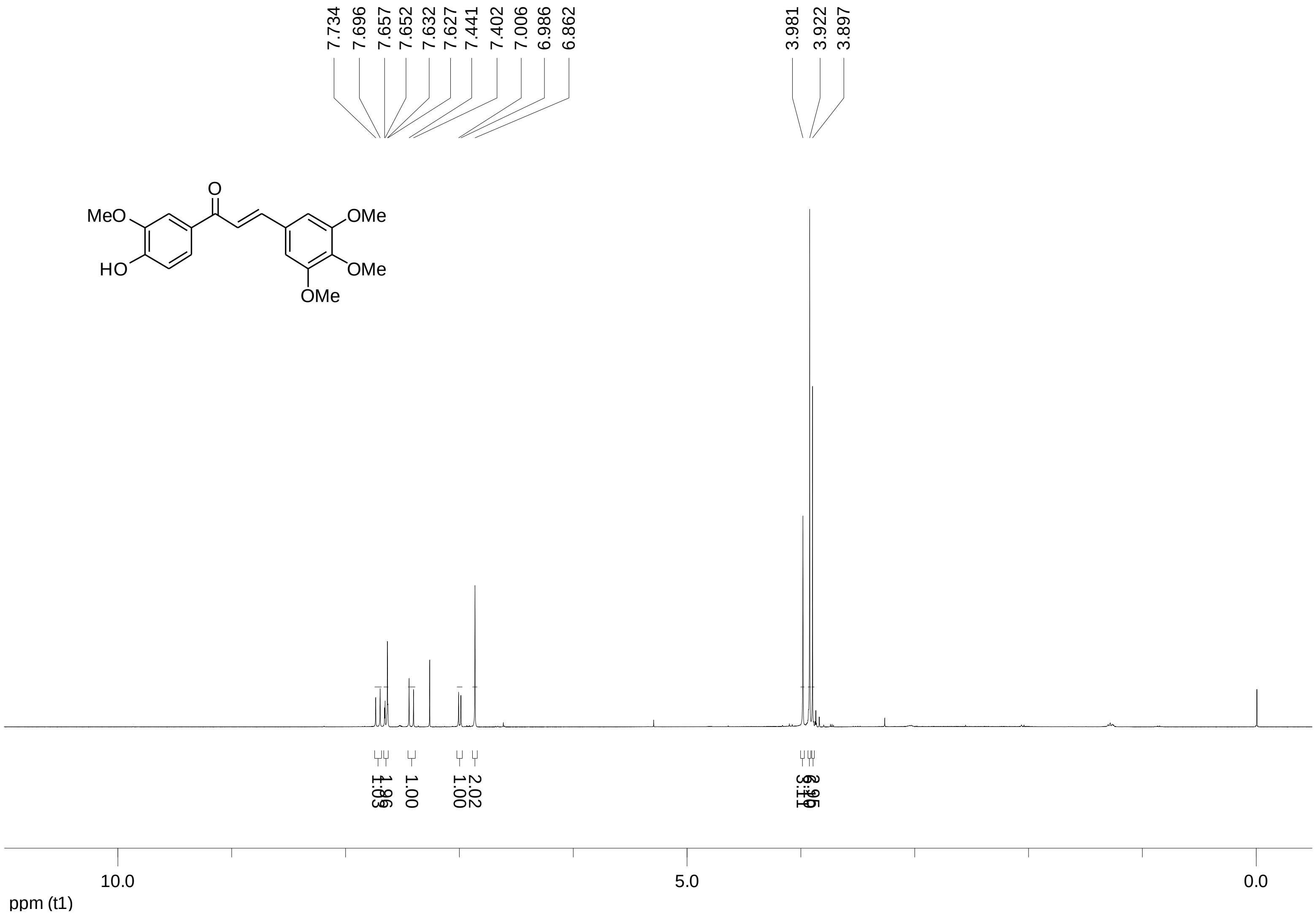

Supplementary Figure 34. ^{1}H NMR spectrum for 1ad

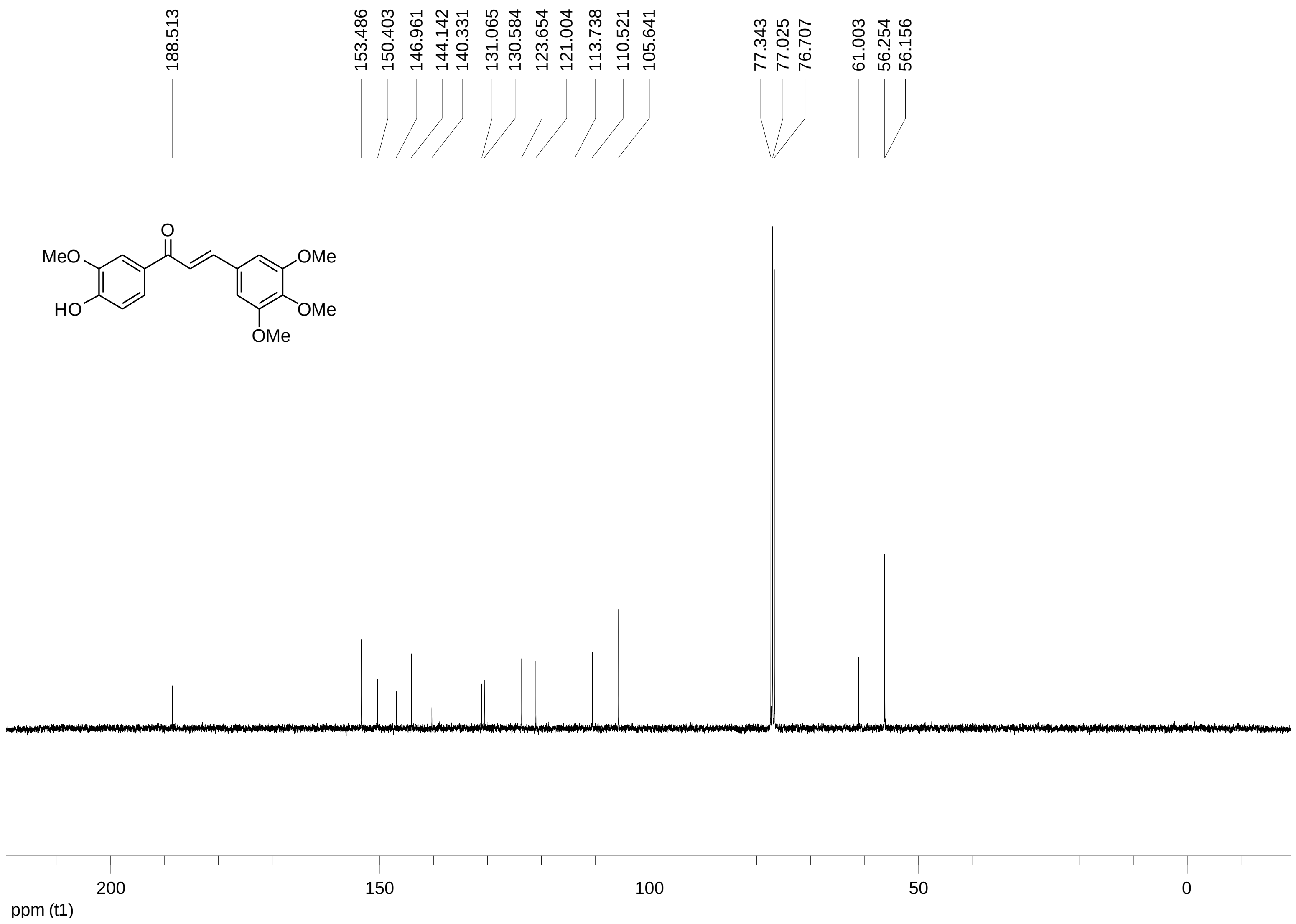

Supplementary Figure 35. ^{13}C NMR spectrum for 1ad

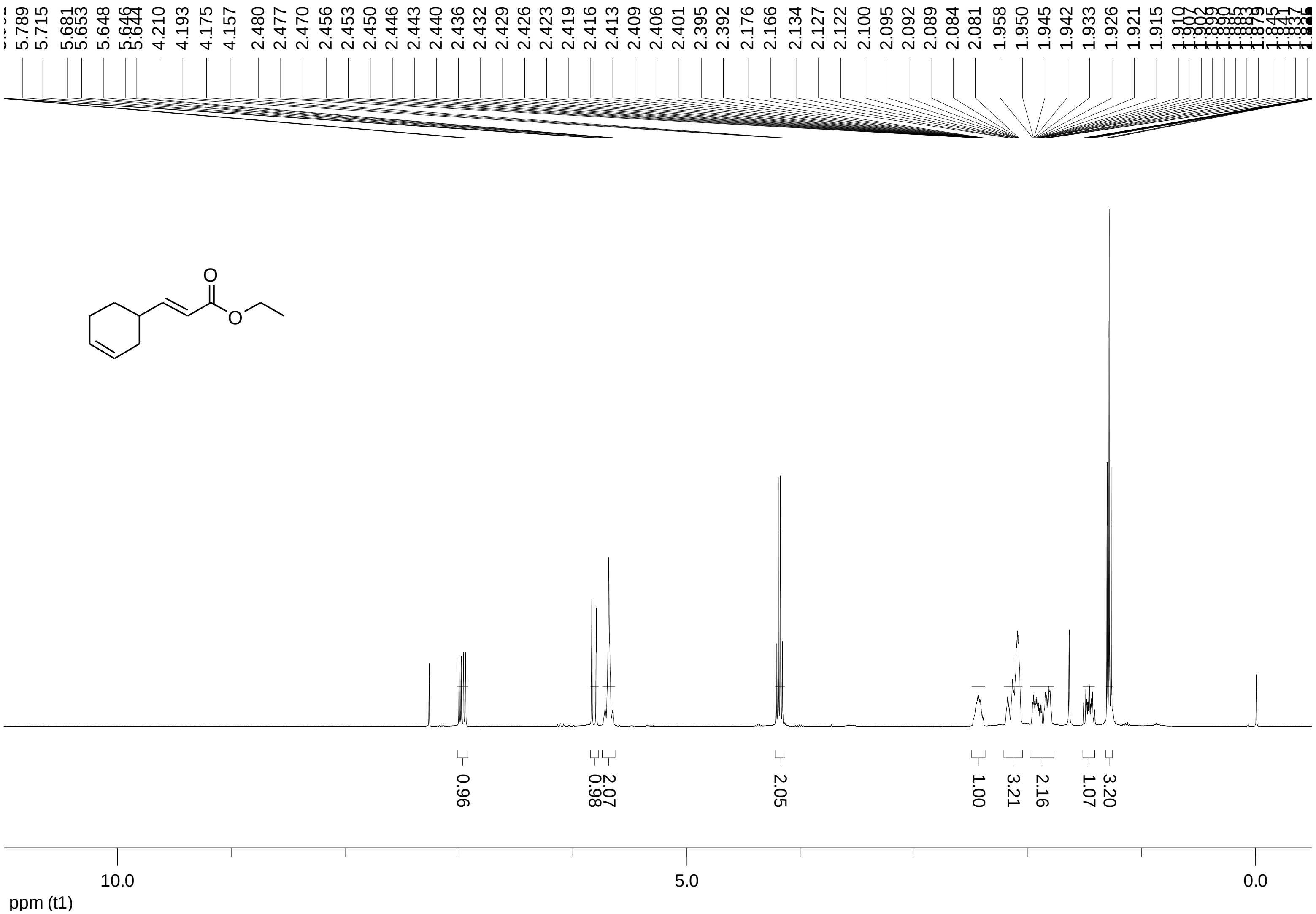

Supplementary Figure 36. ^{1}H NMR spectrum for 1aj

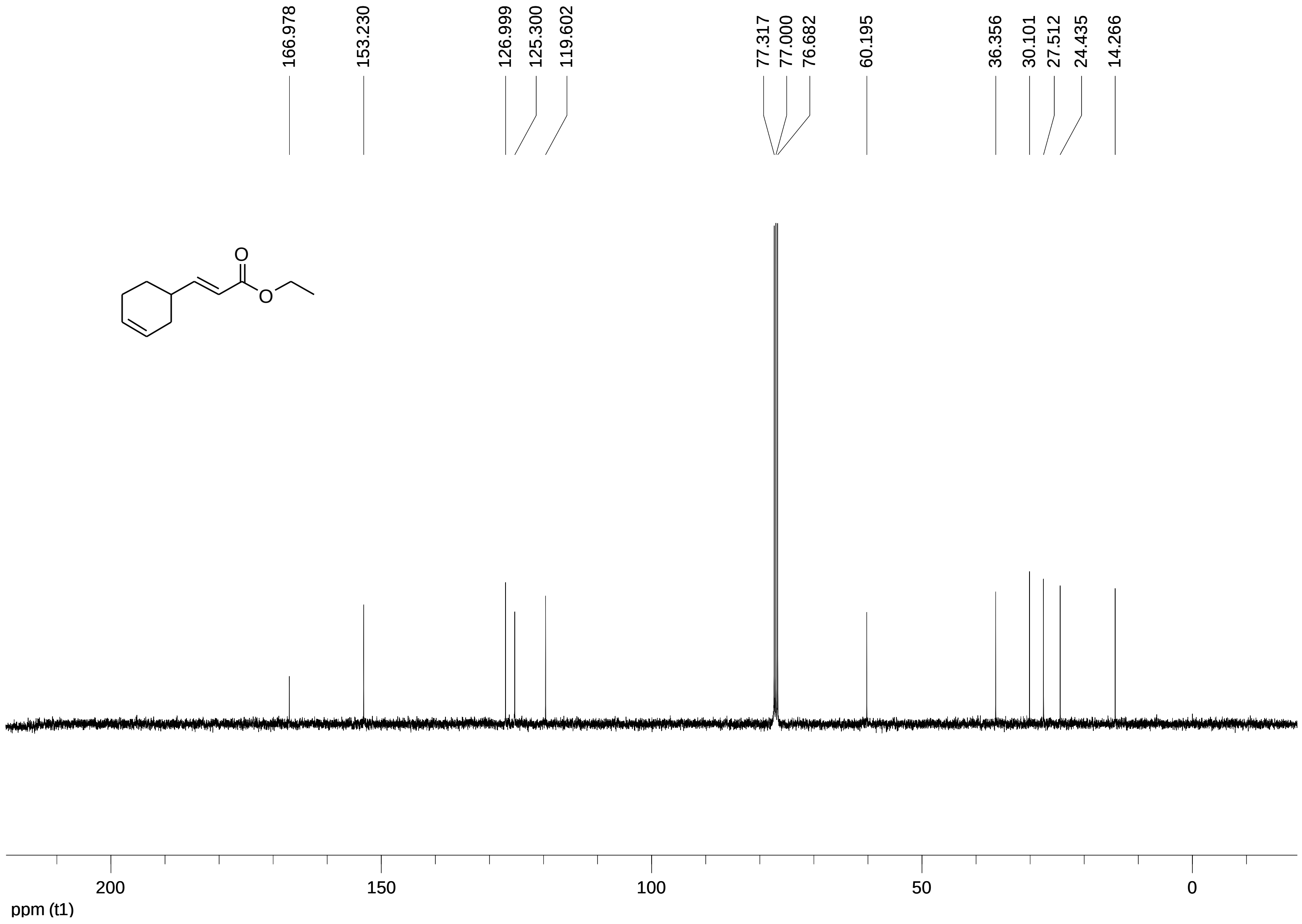

Supplementary Figure 37. ^{13}C NMR spectrum for 1aj

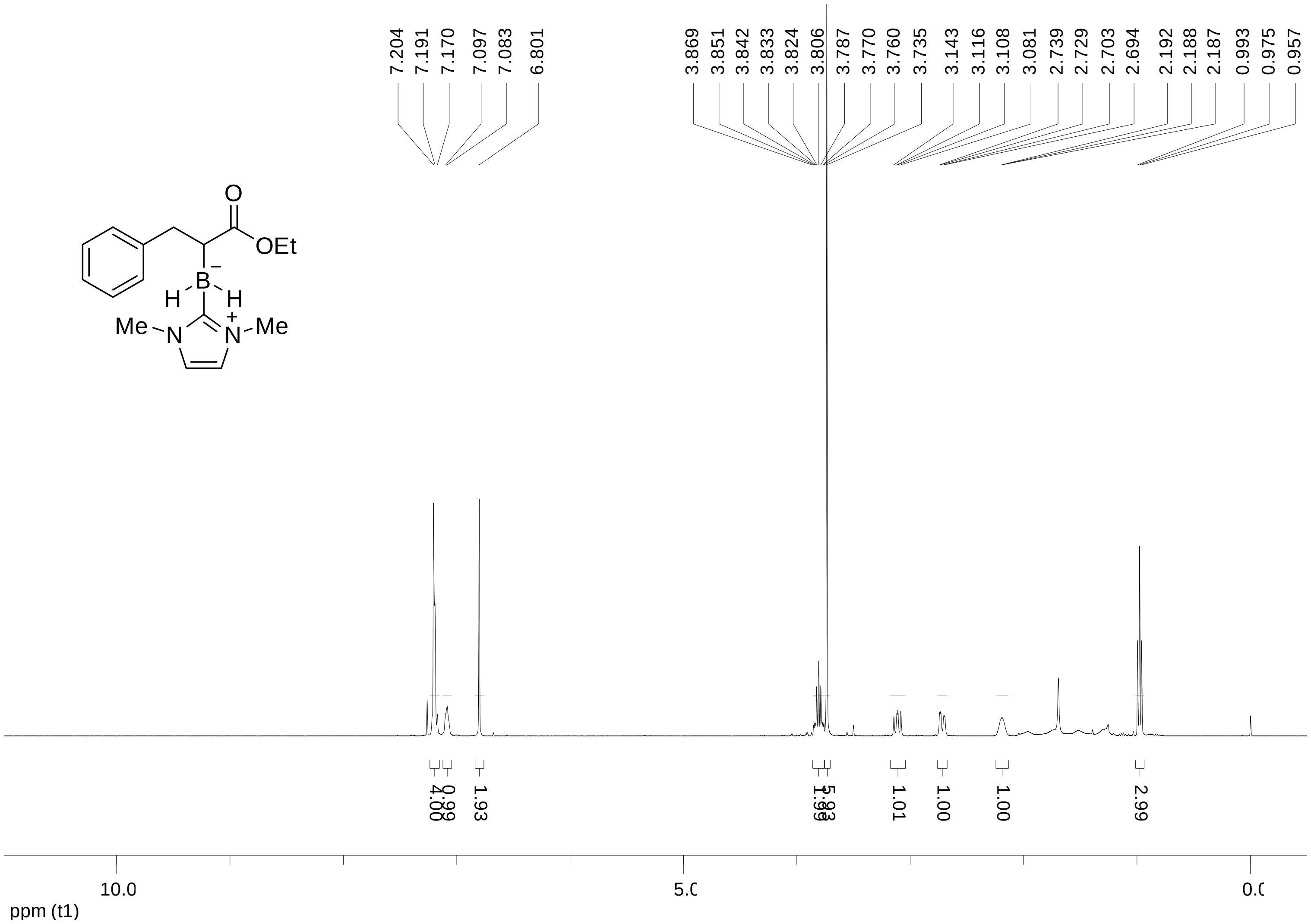

Supplementary Figure 38. ^{1}H NMR spectrum for 3a

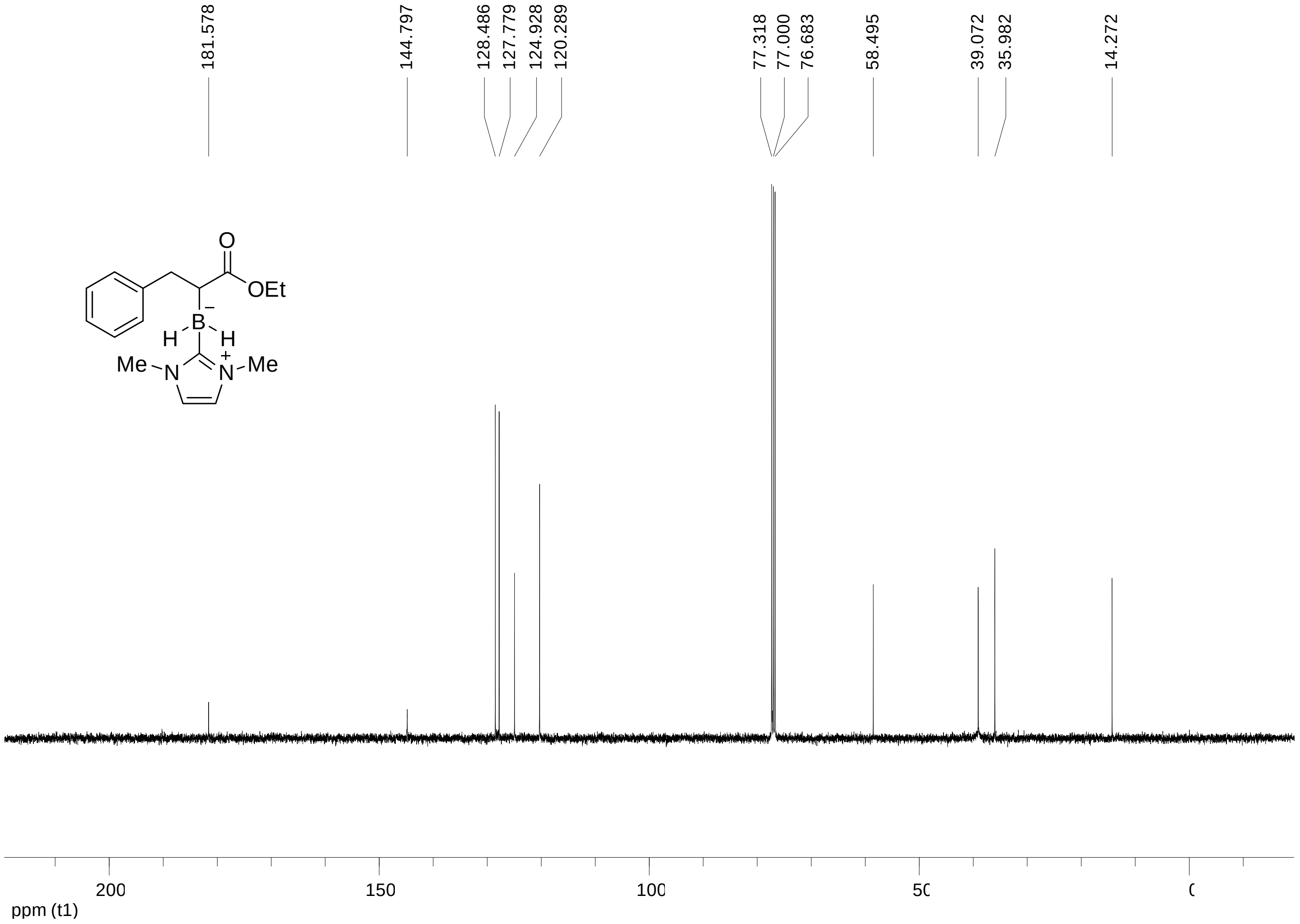

Supplementary Figure 39. ^{13}C NMR spectrum for 3a

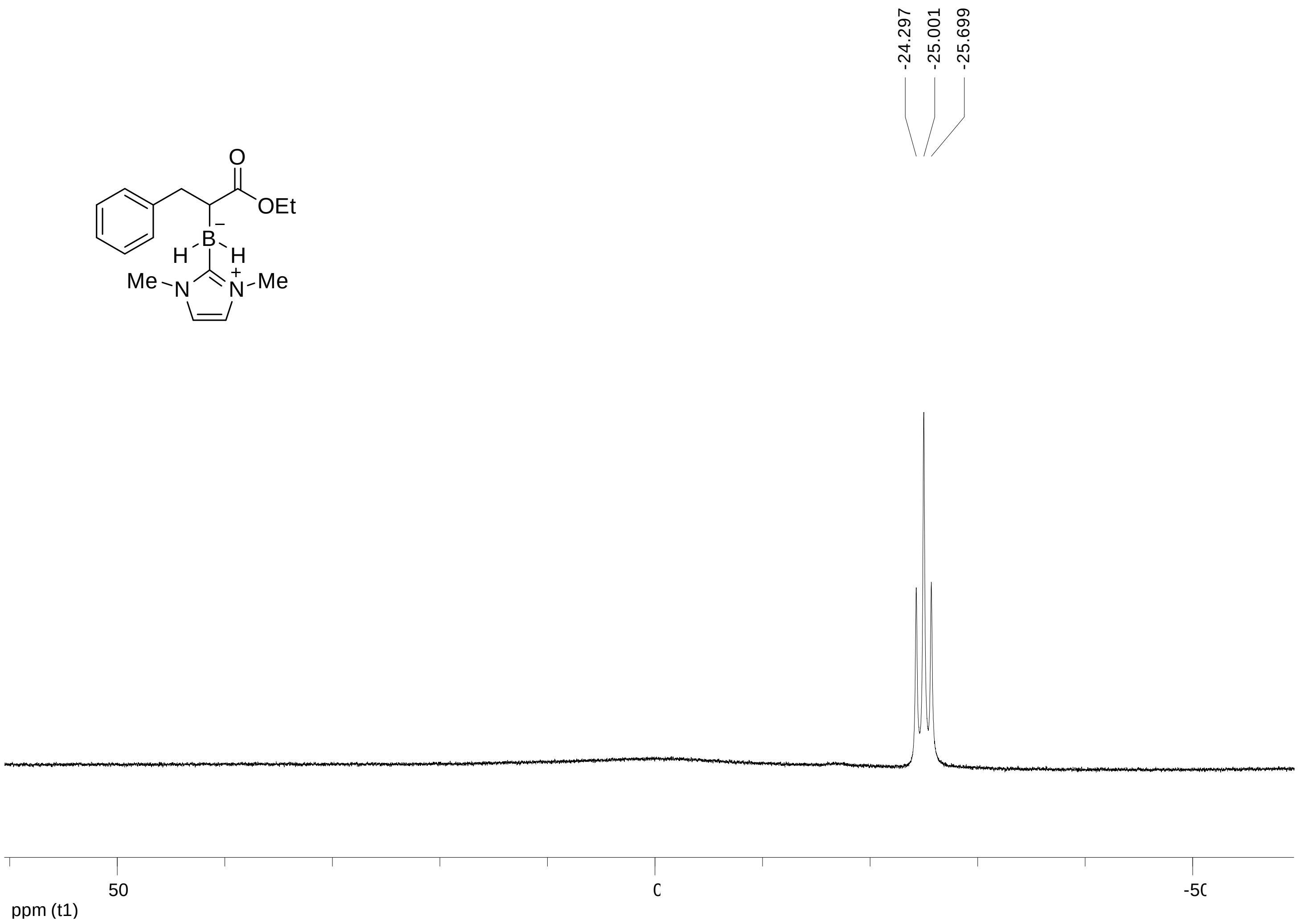

Supplementary Figure 40. ^{11}B NMR spectrum for 3a

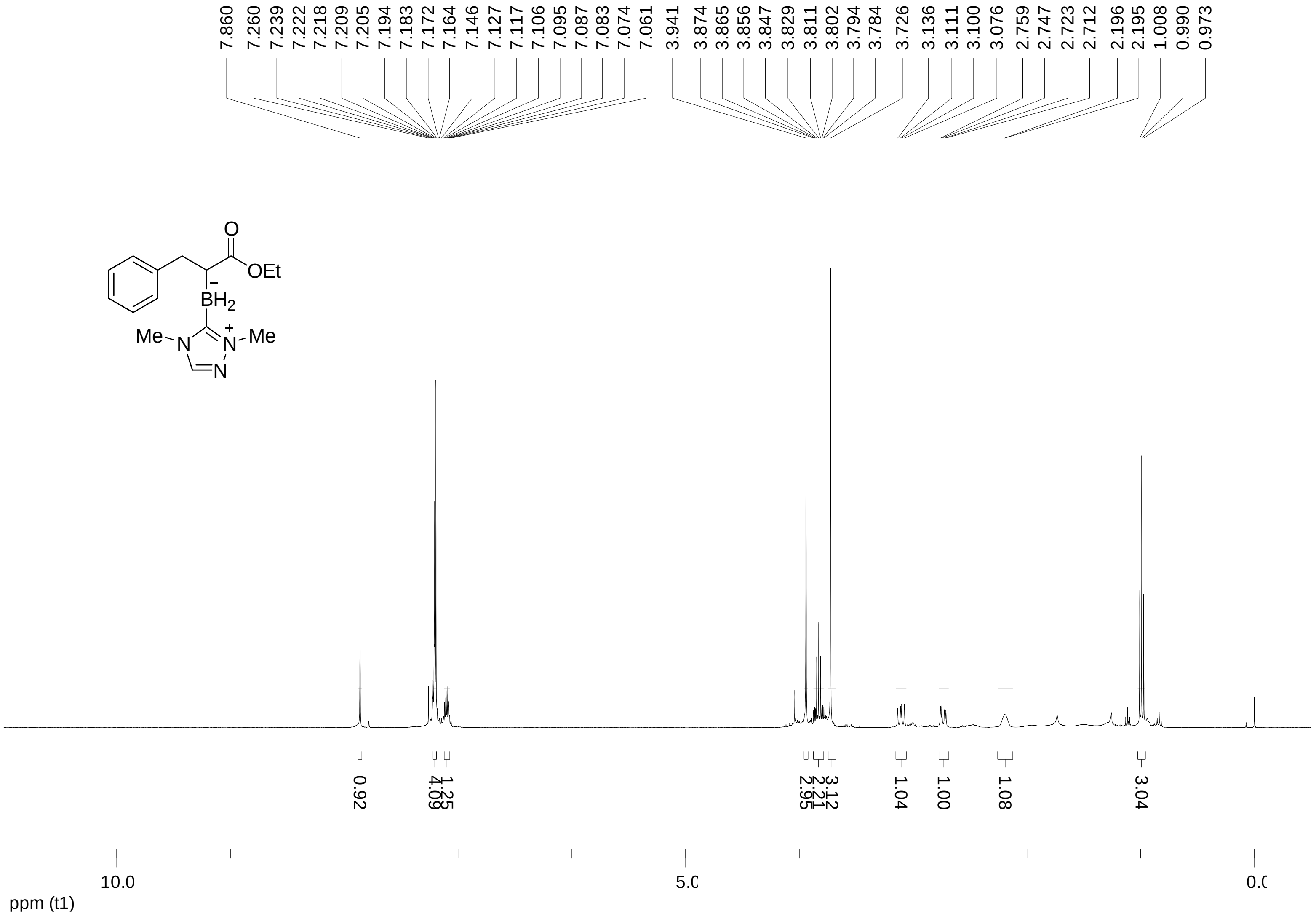

Supplementary Figure 41. ^{1}H NMR spectrum for 3a-B

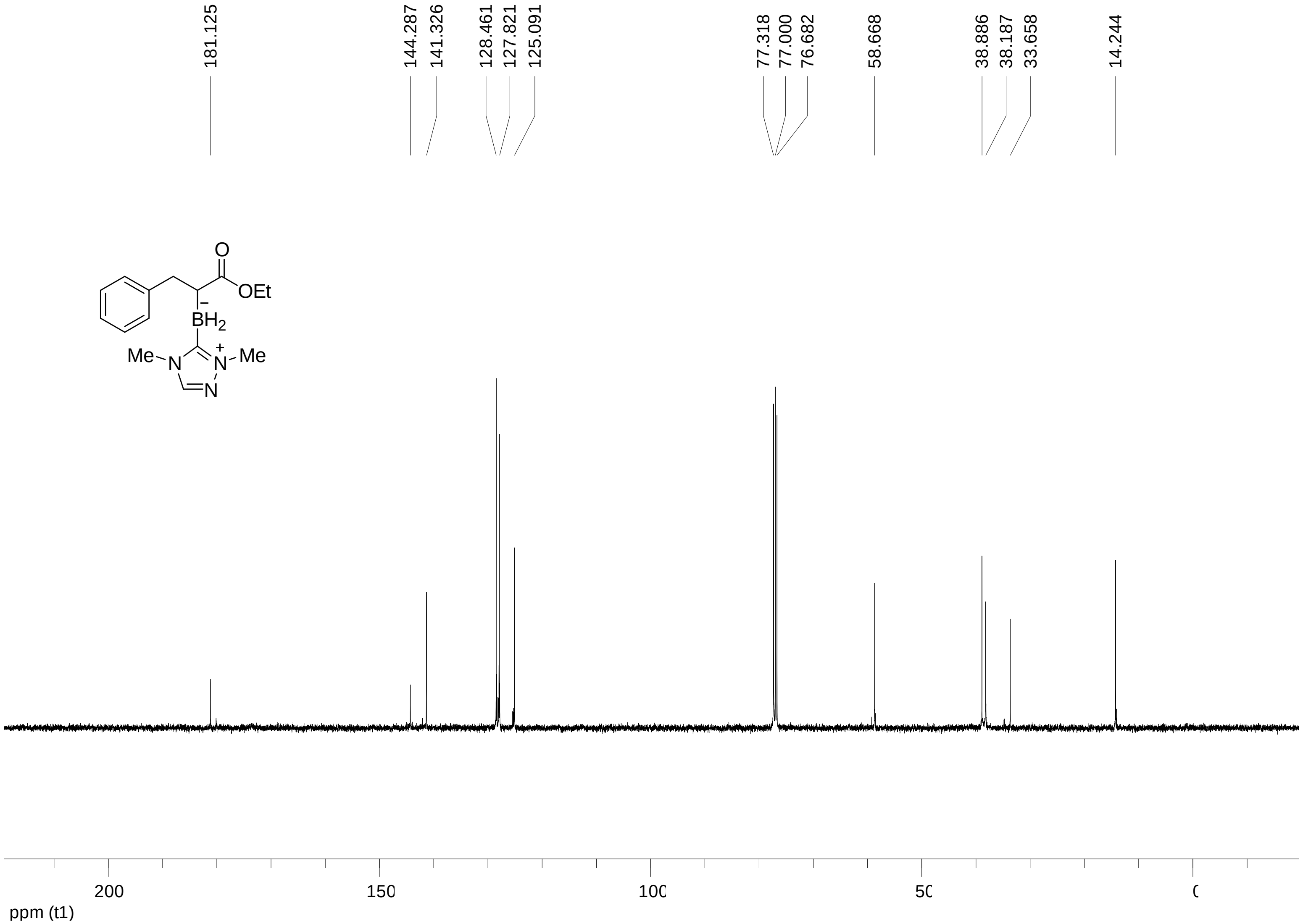

Supplementary Figure 42. ^{13}C NMR spectrum for 3a-B

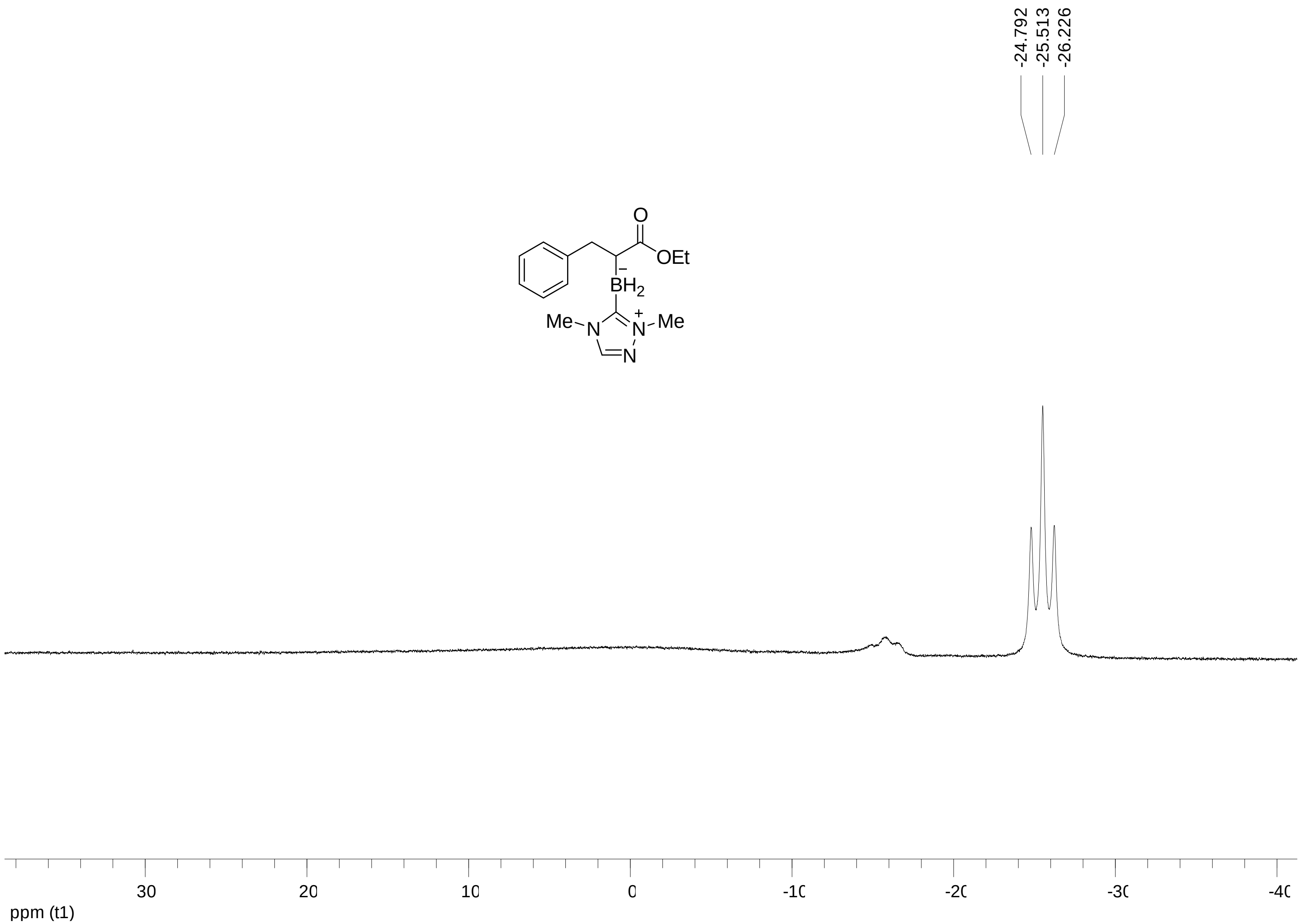

Supplementary Figure 43. ^{11}B NMR spectrum for 3a-B

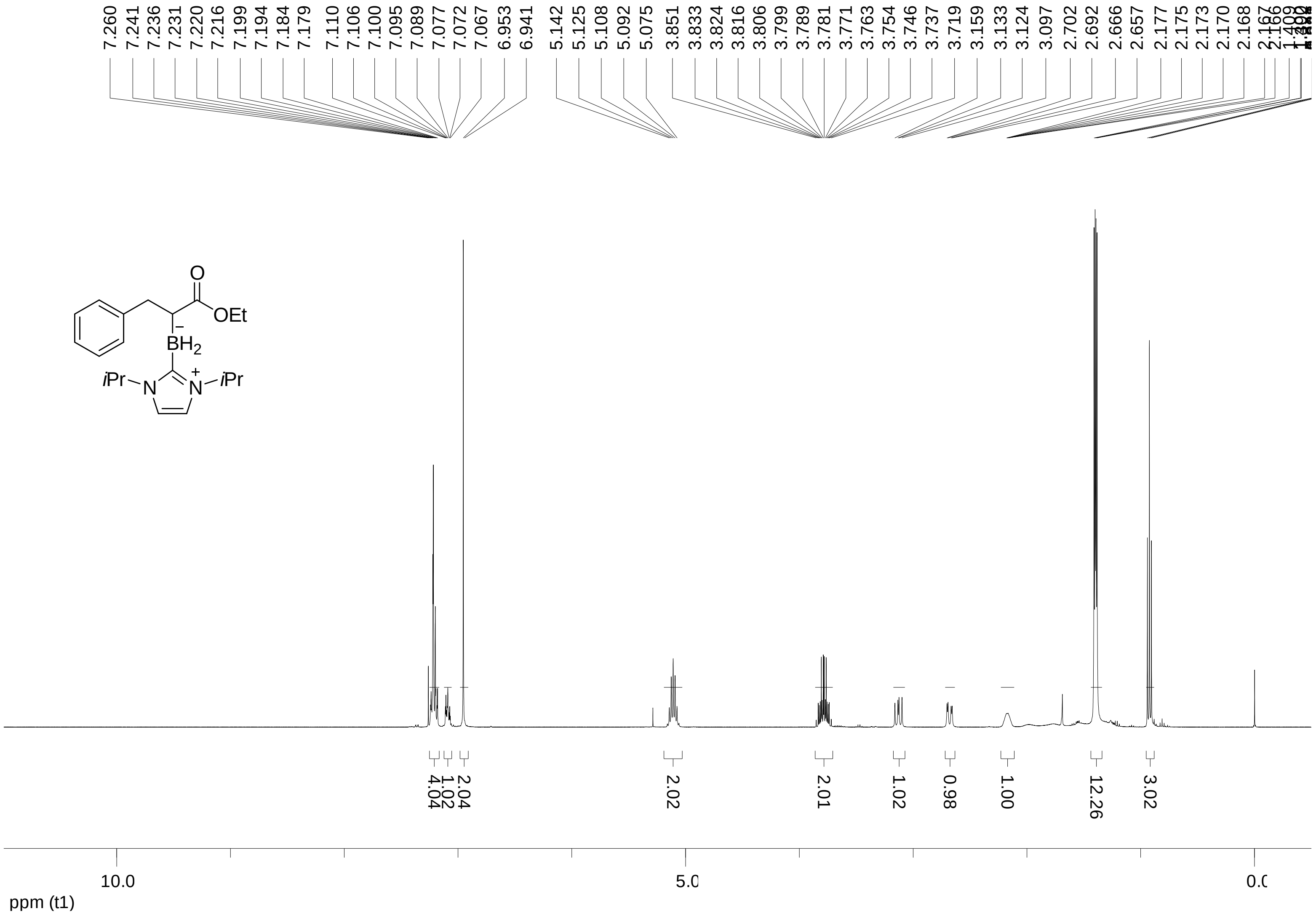

Supplementary Figure 44. ^{1}H NMR spectrum for 3-C

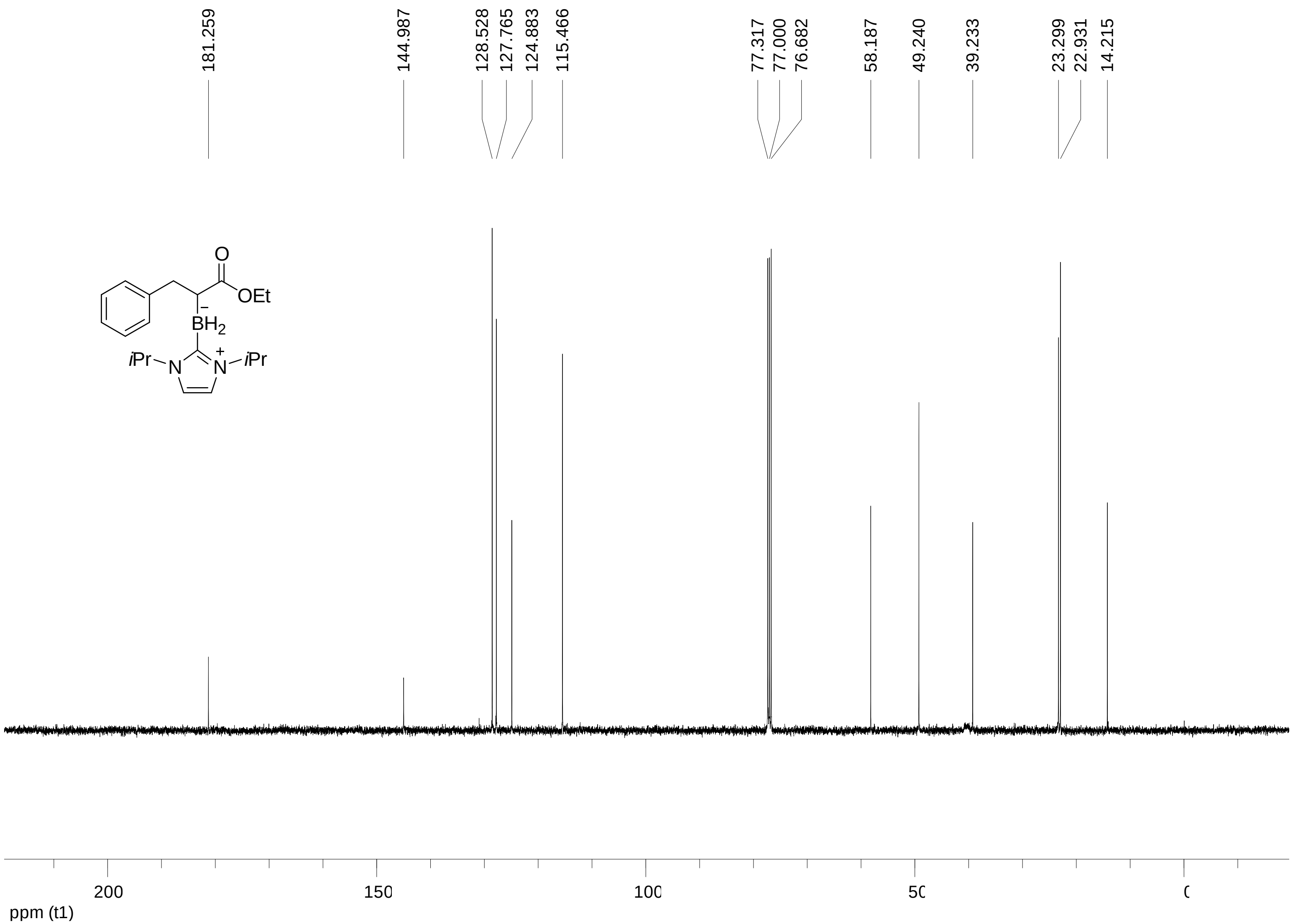

Supplementary Figure 45. ^{13}**C NMR spectrum for 3a-C**

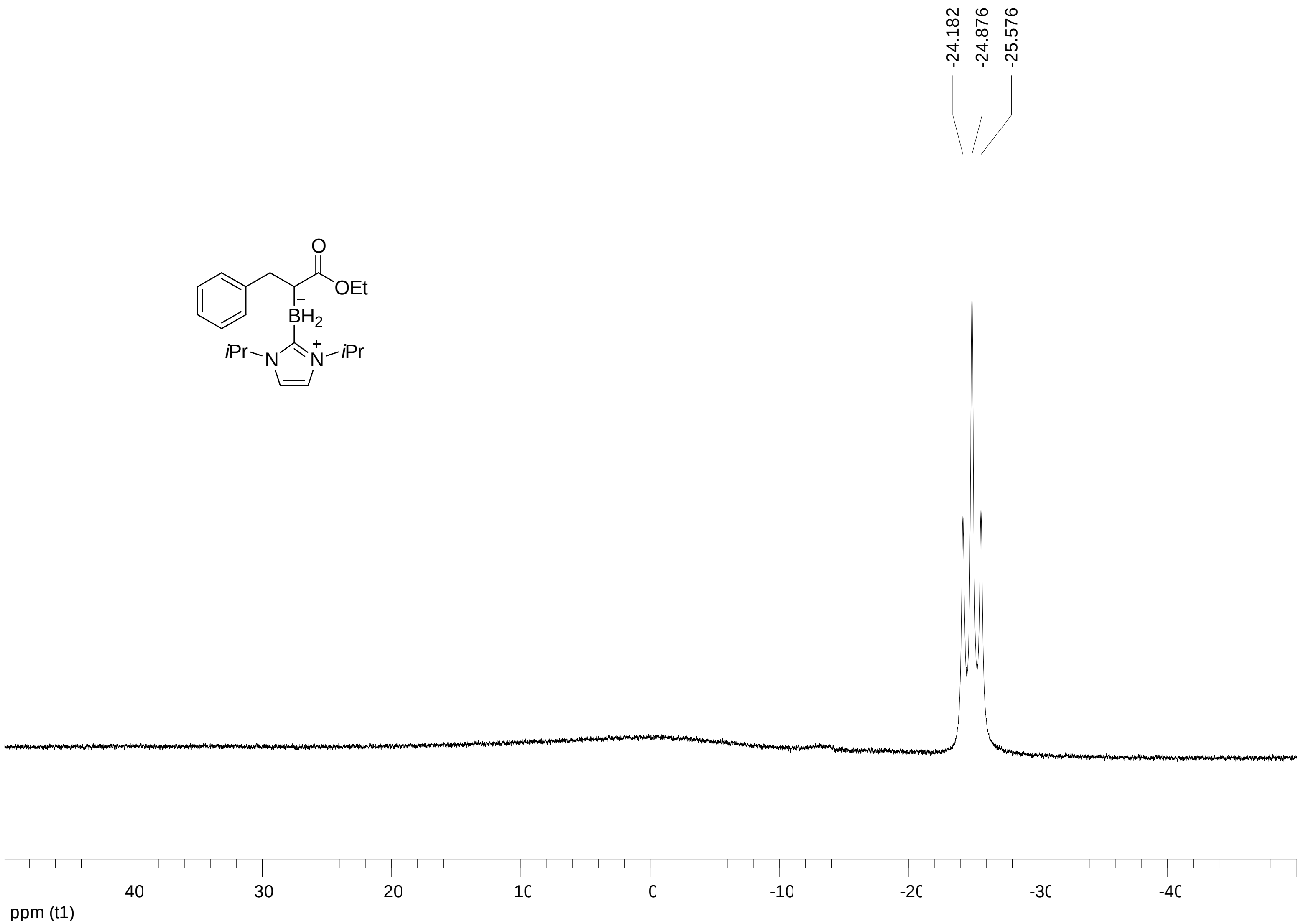

Supplementary Figure 46. ^{11}B NMR spectrum for 3a-C

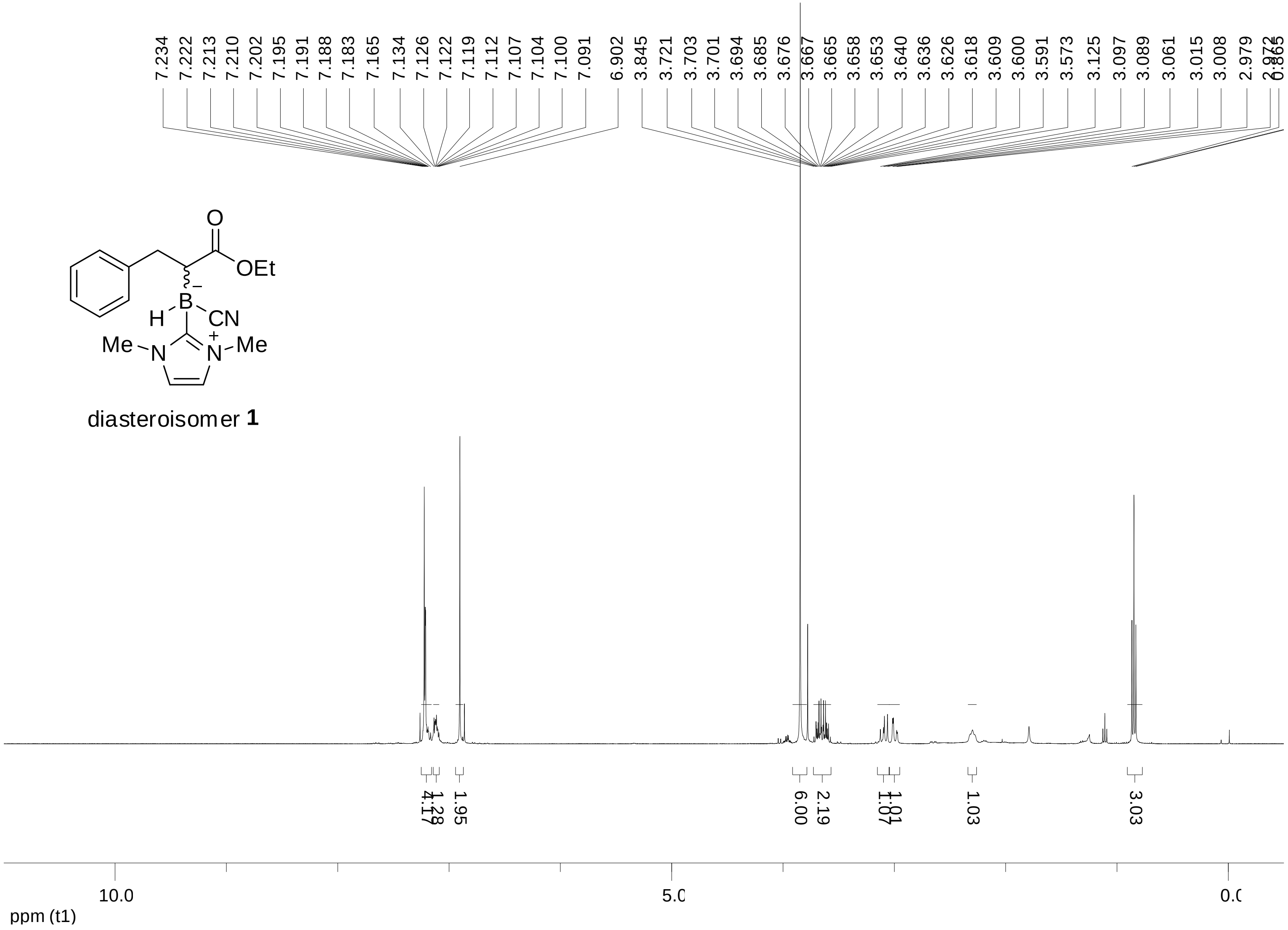

Supplementary Figure 47. ^{1}H NMR spectrum for 3a-D(diasteroisomer 1)

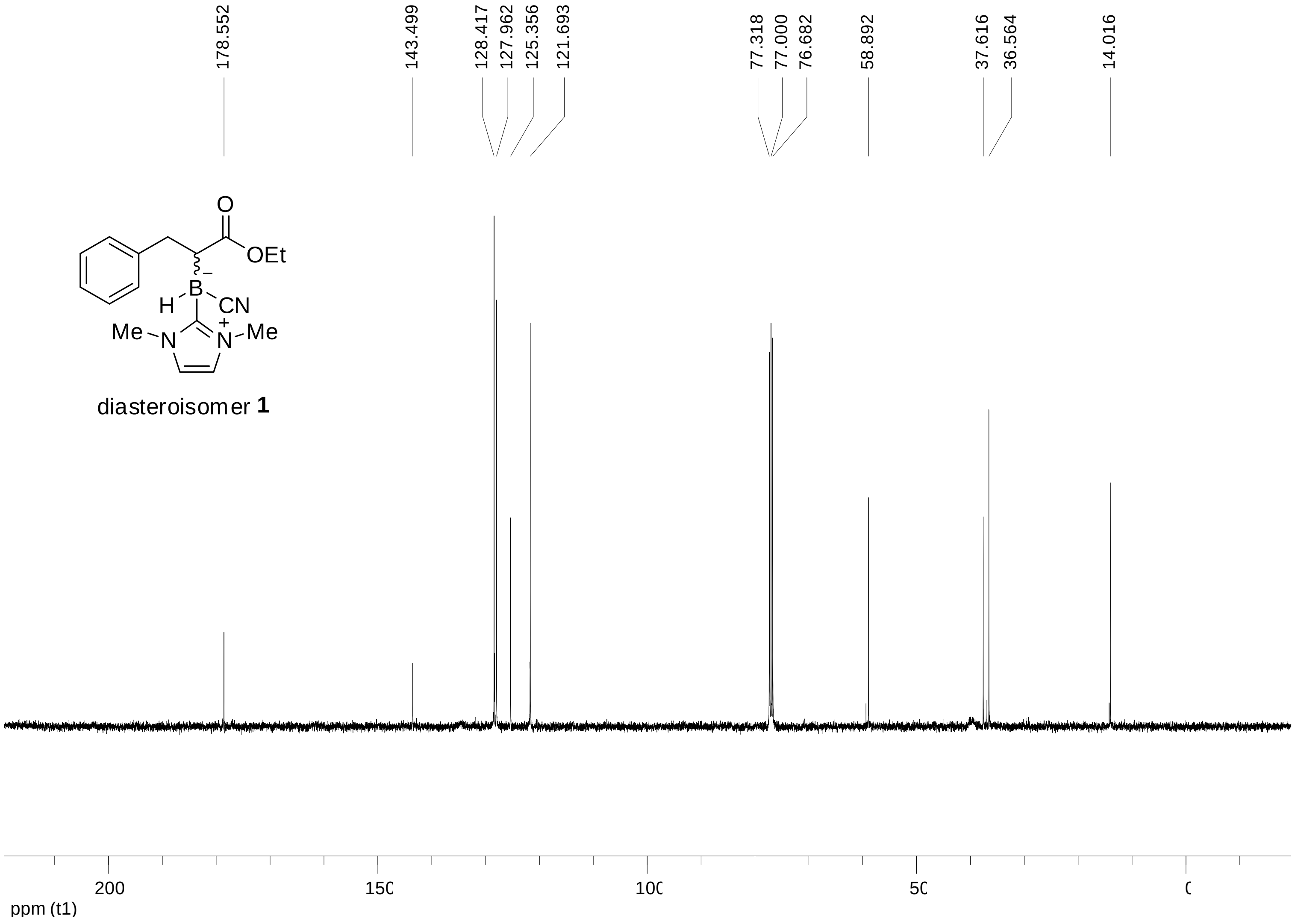

Supplementary Figure 48. ^{13}C NMR spectrum for 3a-D(diasteroisomer 1)

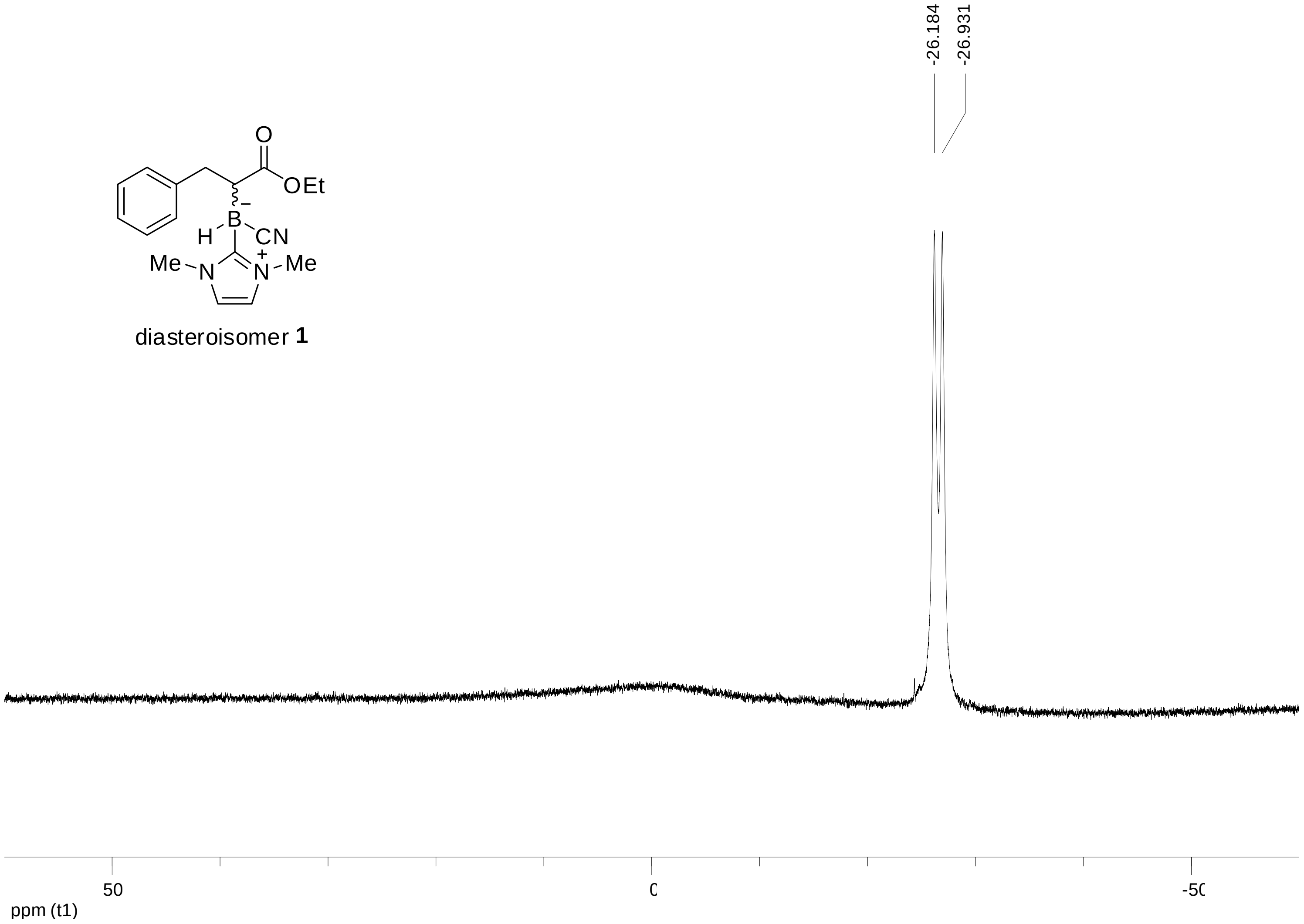

Supplementary Figure 49. ^{11}B NMR spectrum for 3a-D(diasteroisomer 1)

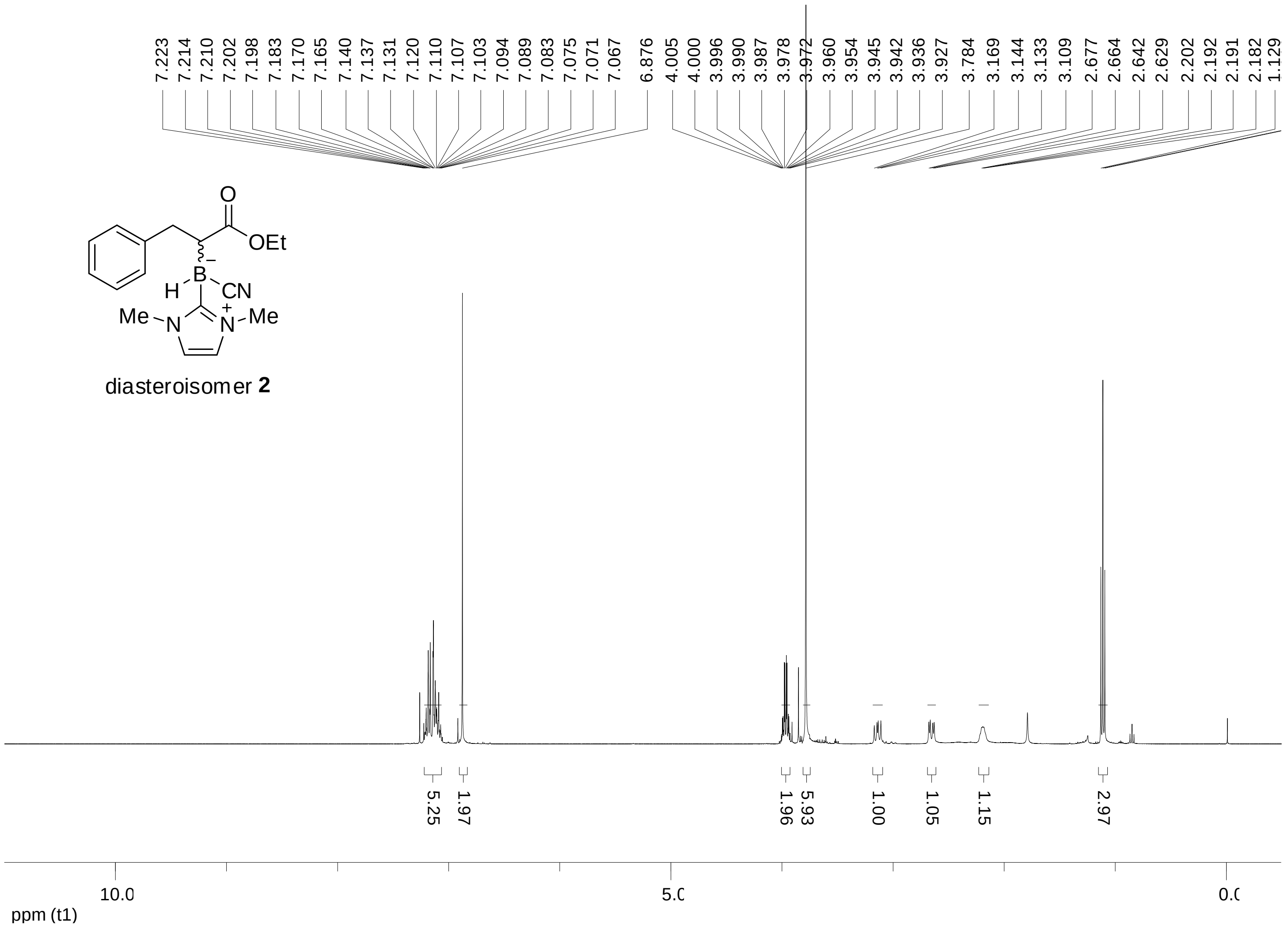

Supplementary Figure 50. ^{1}H NMR spectrum for 3a-D(diasteroisomer 2)

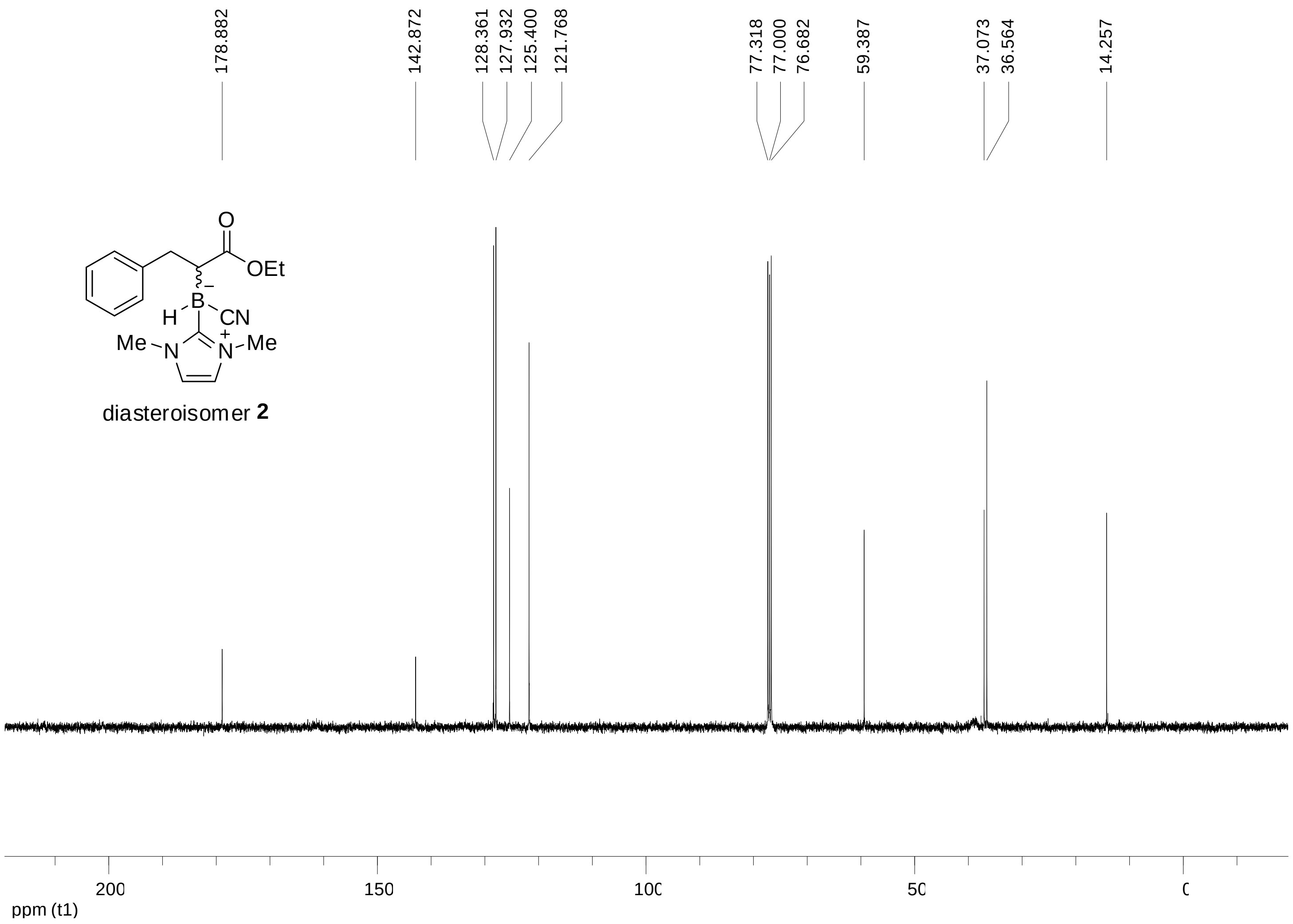

Supplementary Figure 51. ^{13}C NMR spectrum for 3a-D(diasterosiomer 2)

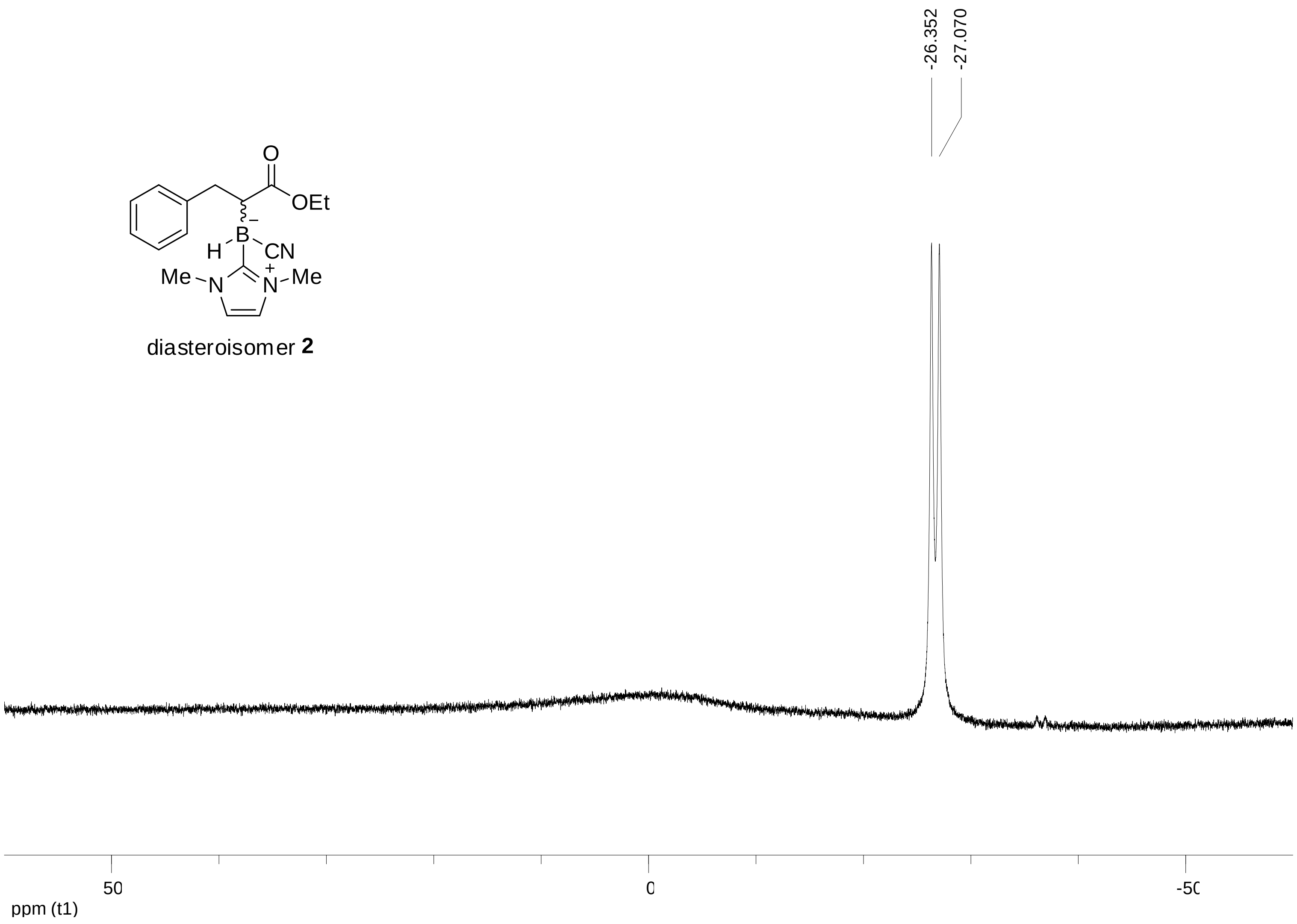

Supplementary Figure 52. ^{11}B NMR spectrum for 3a-D(diasteroisomer 2)

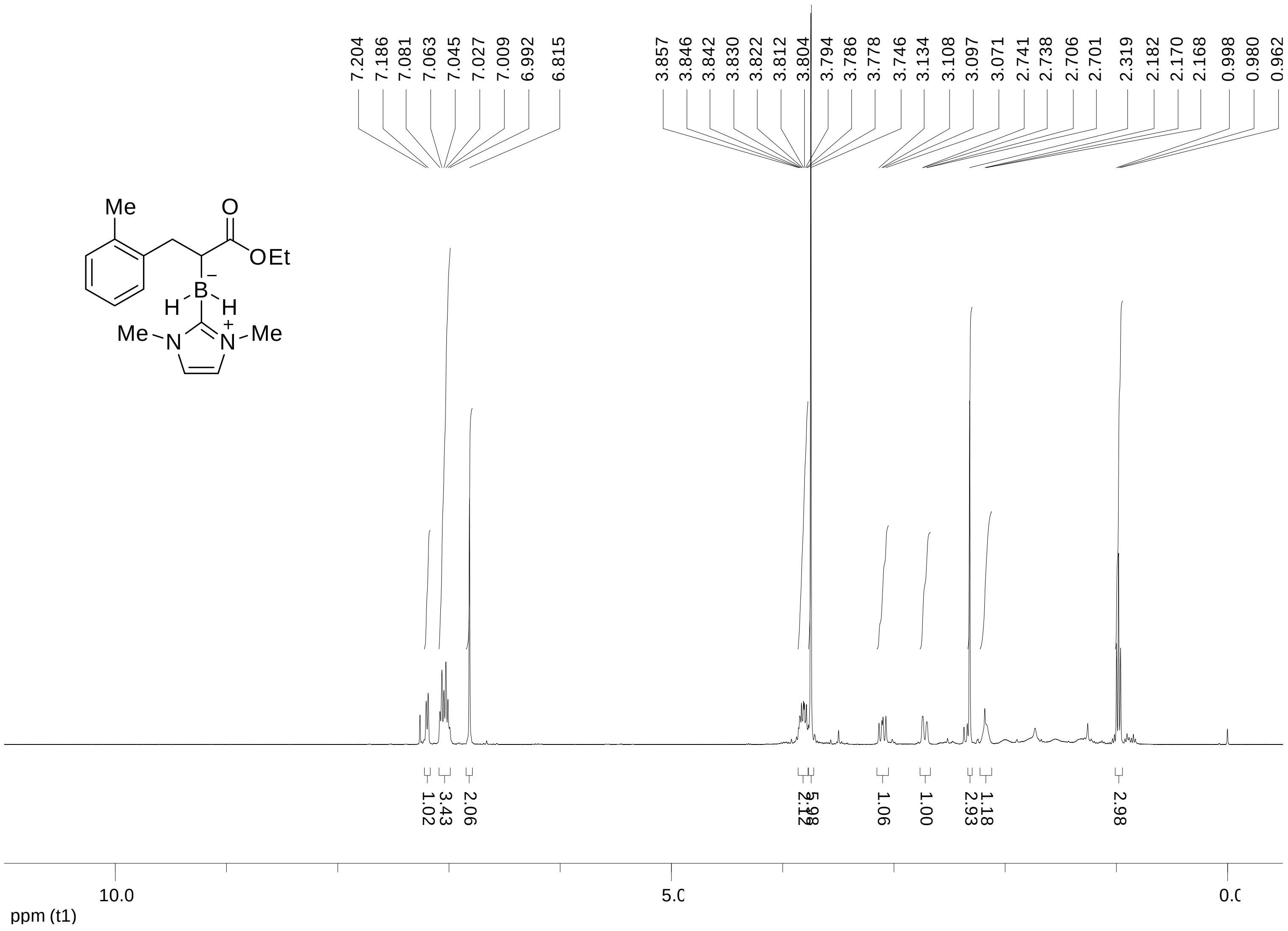

Supplementary Figure 53. ^{1}H NMR spectrum for 3b

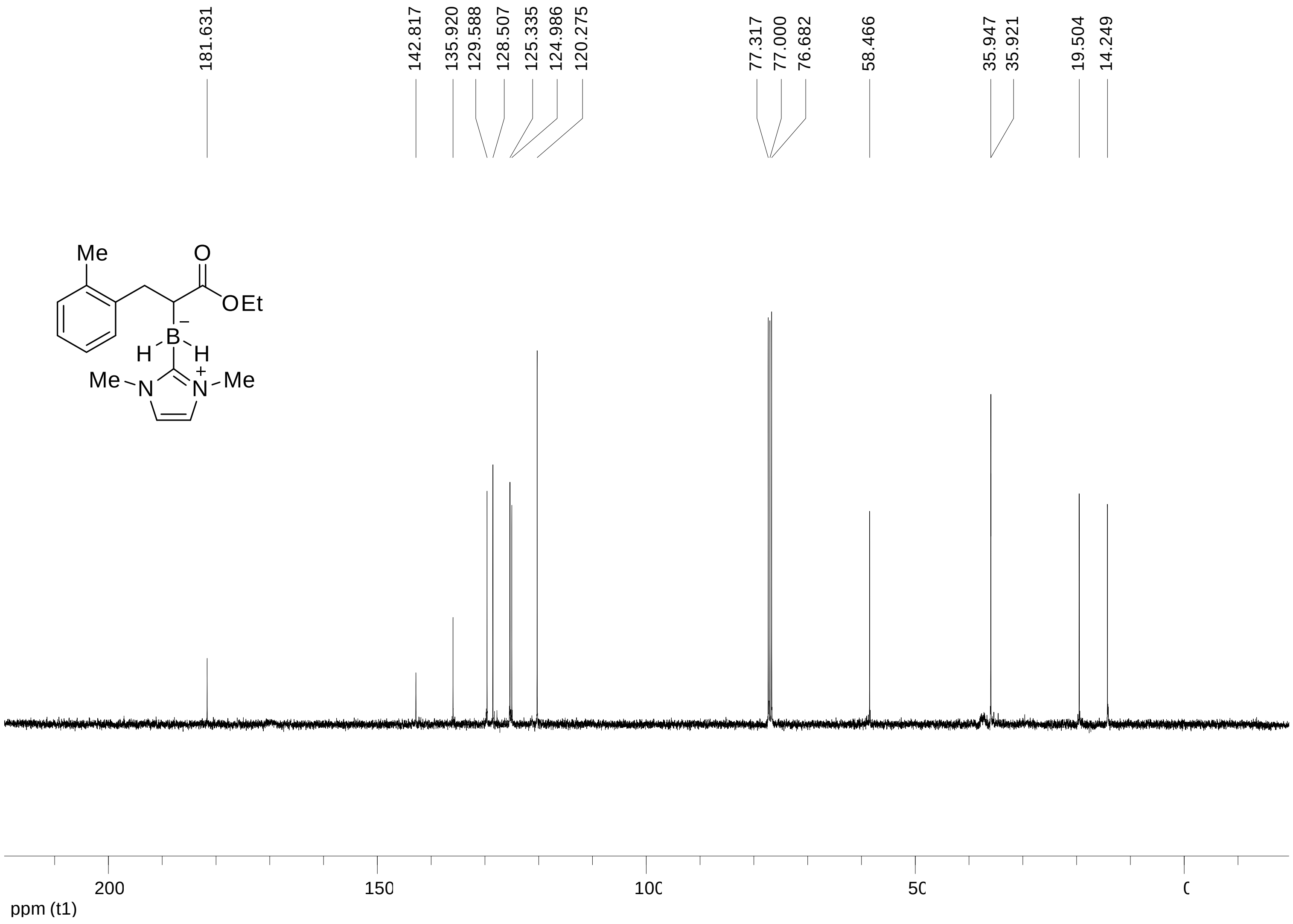

Supplementary Figure 54. ^{13}C NMR spectrum for 3b

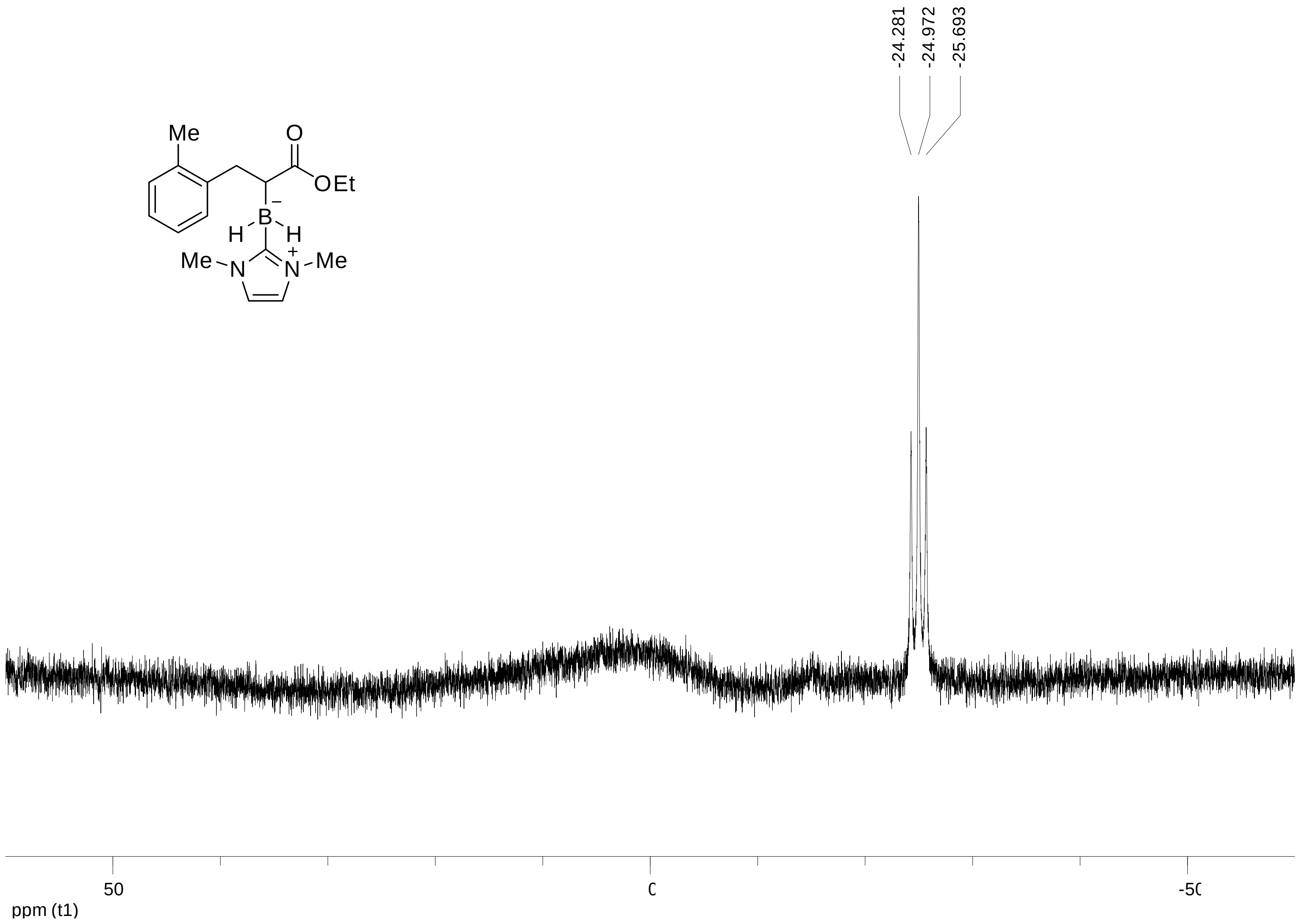

Supplementary Figure 55. ^{11}B NMR spectrum for 3b

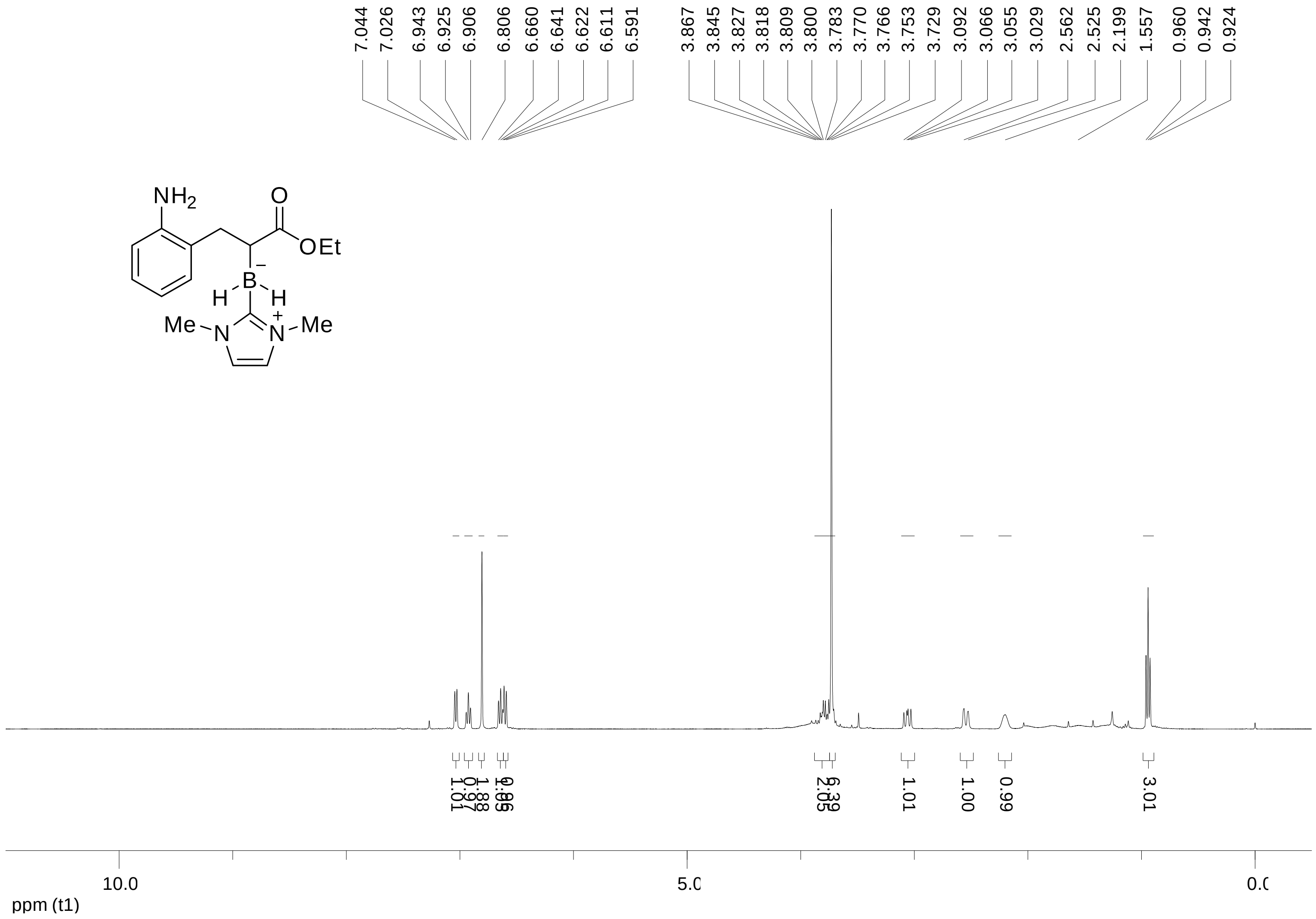

Supplementary Figure 56. ^{1}H NMR spectrum for 3c

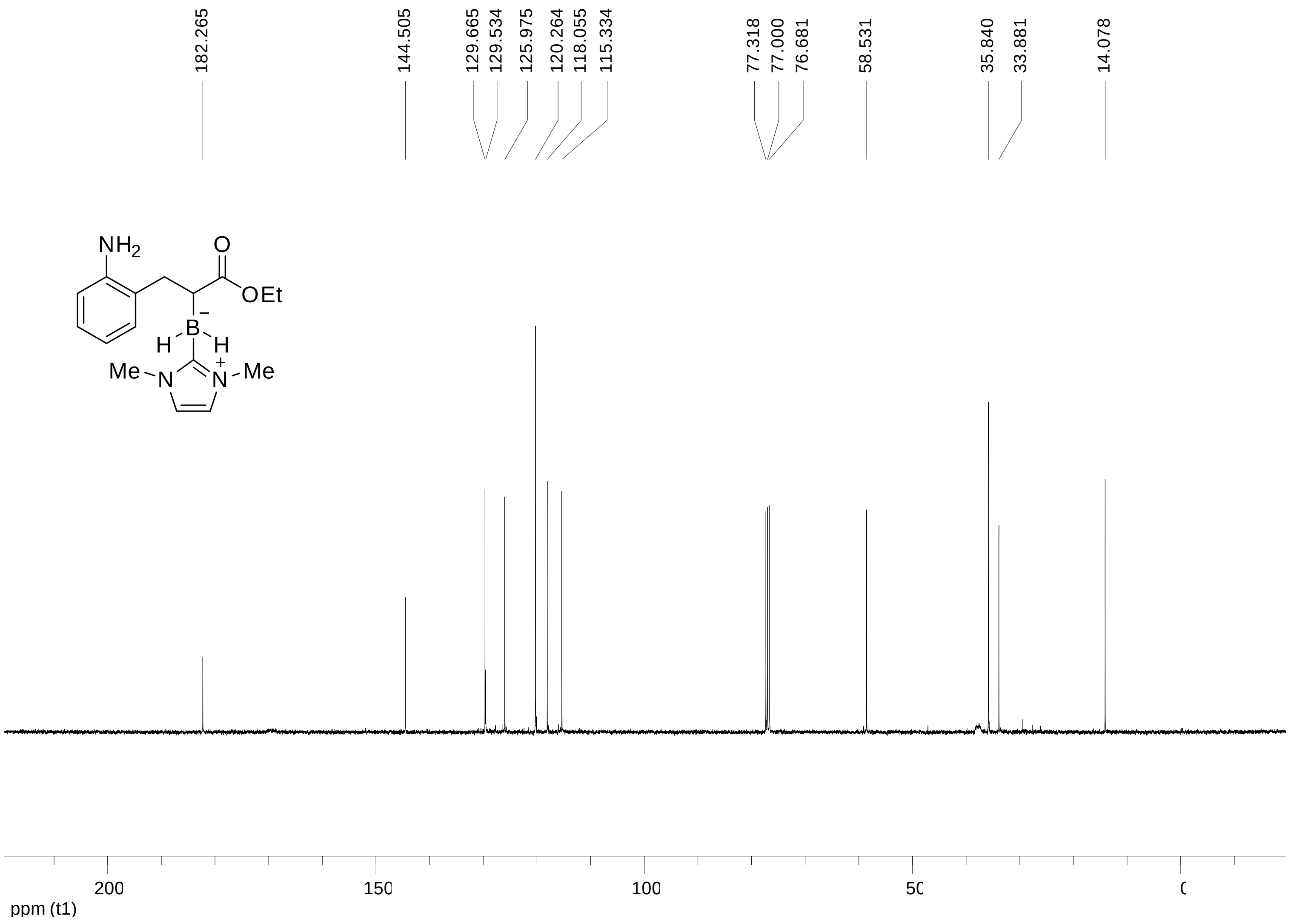

Supplementary Figure 57. ^{13}C NMR spectrum for 3c

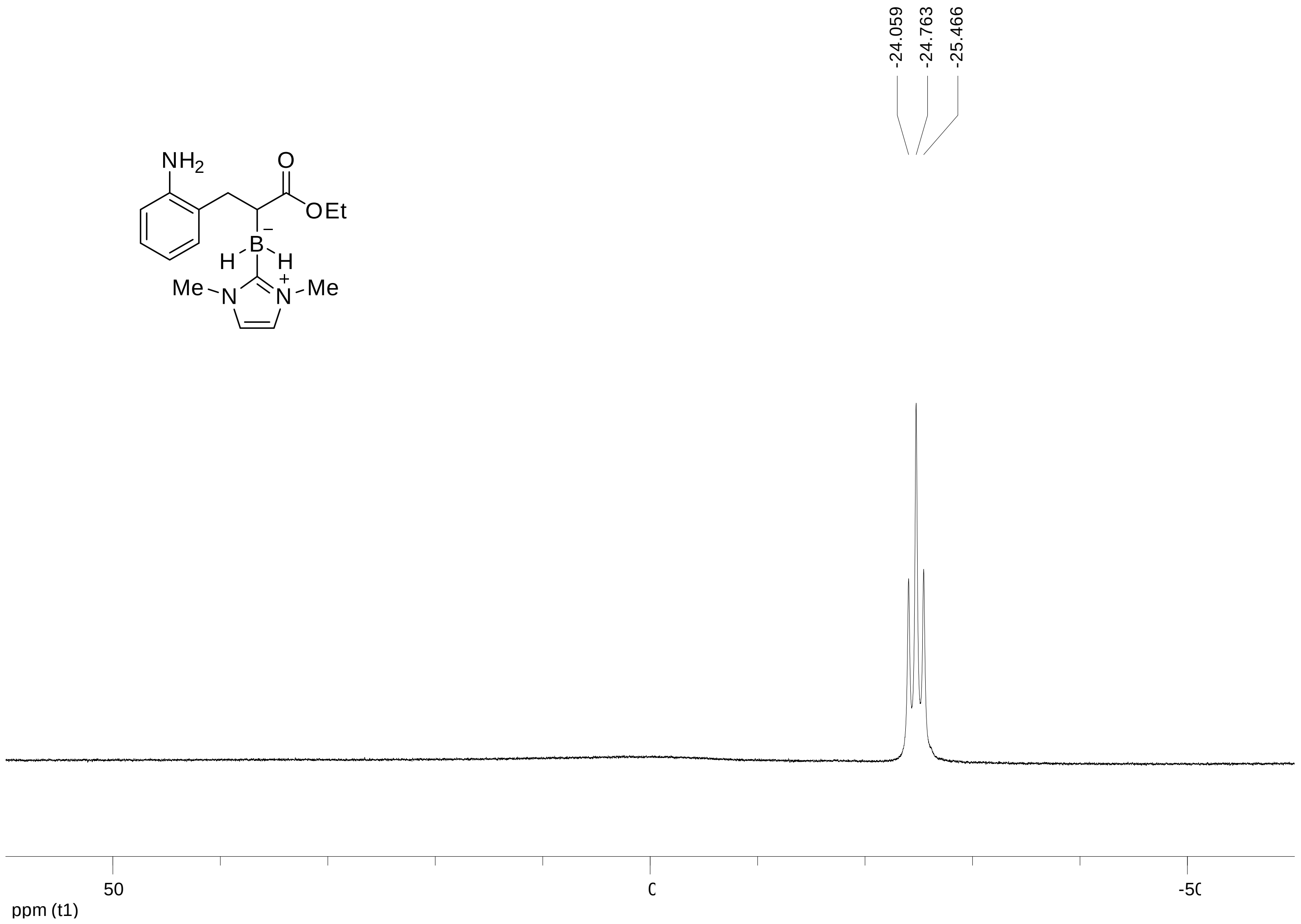

Supplementary Figure 58. ^{11}B NMR spectrum for 3c

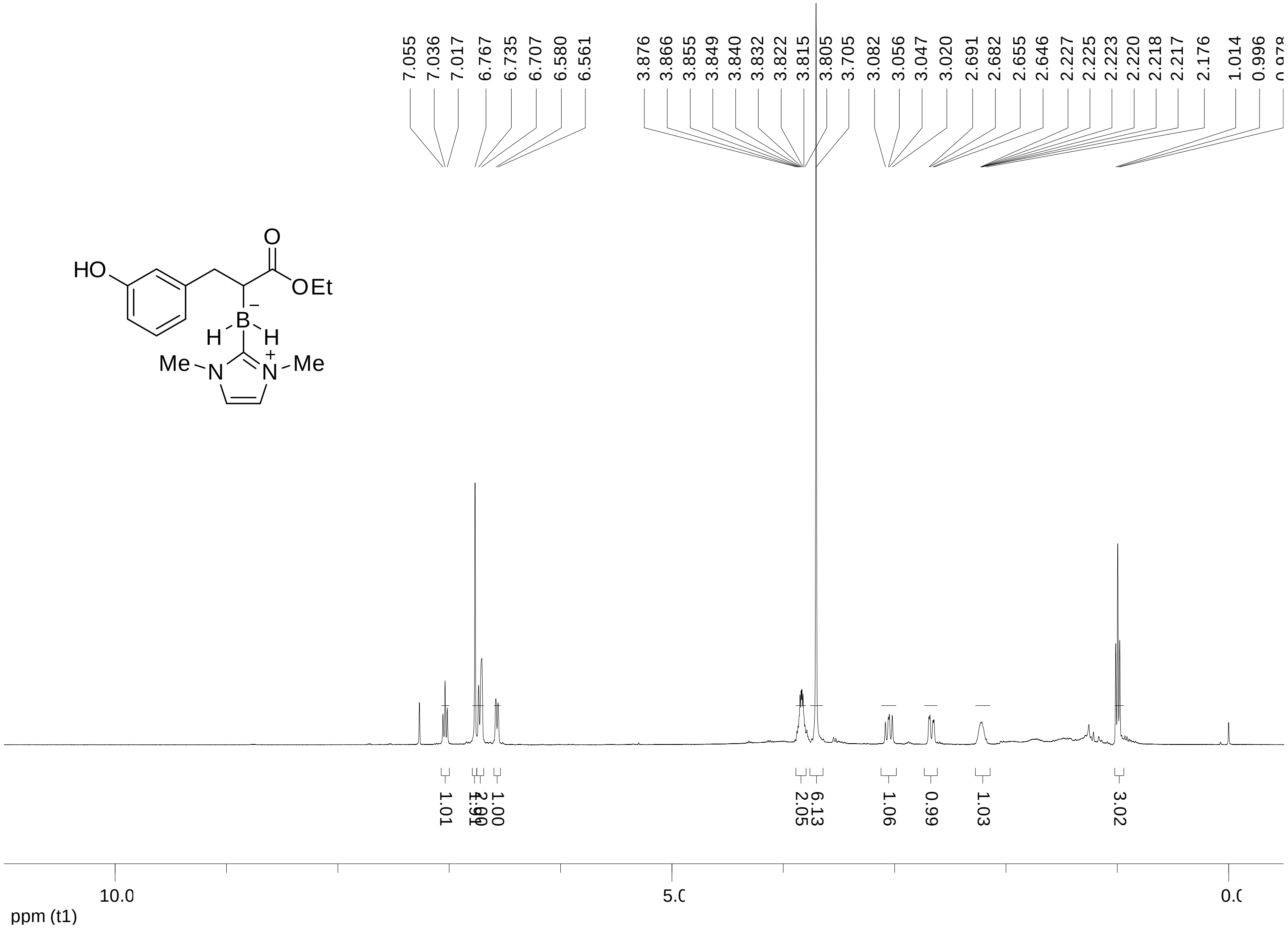

Supplementary Figure 59. ^{1}H NMR spectrum for 3d

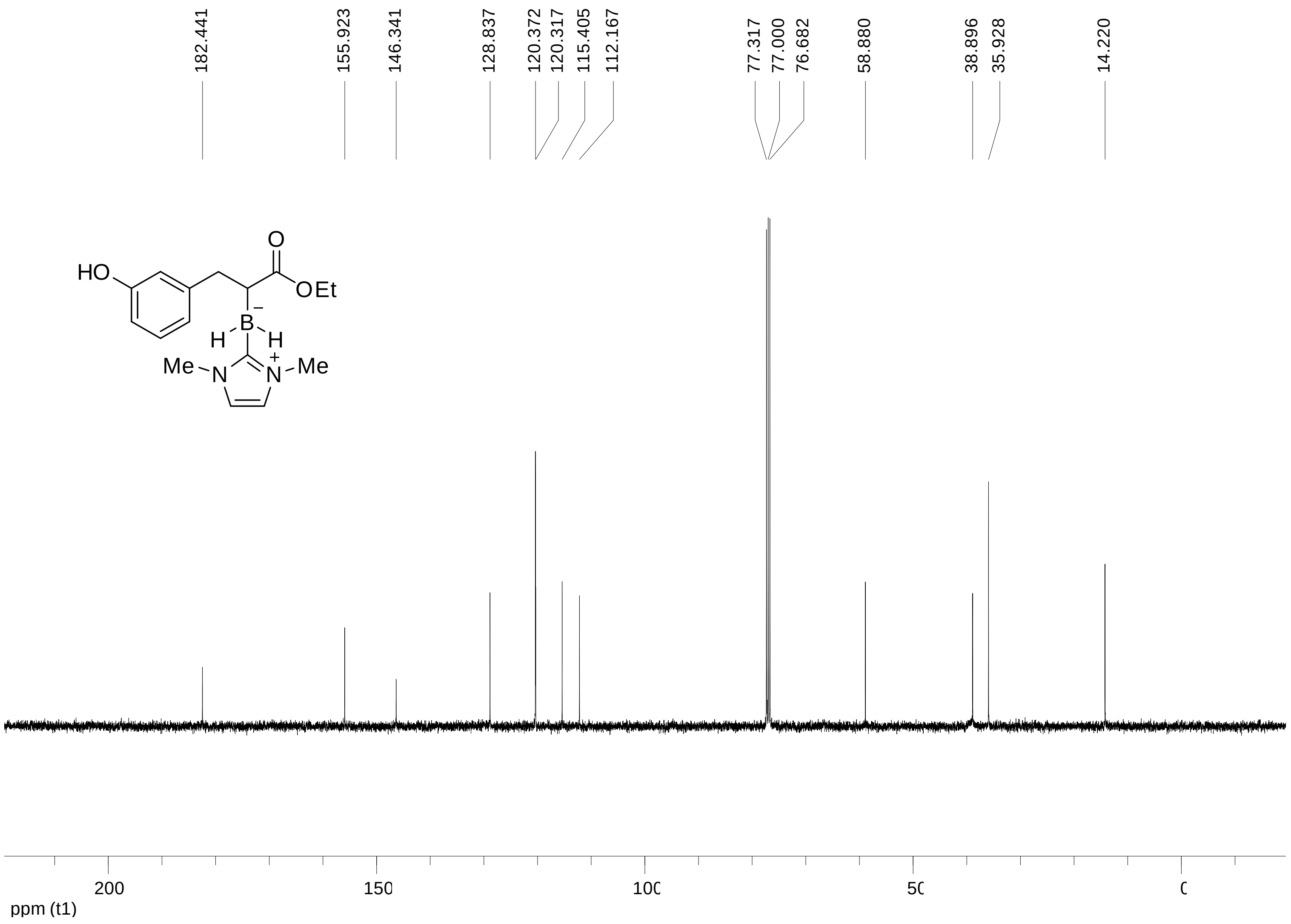

Supplementary Figure 60. ^{13}C NMR spectrum for 3d

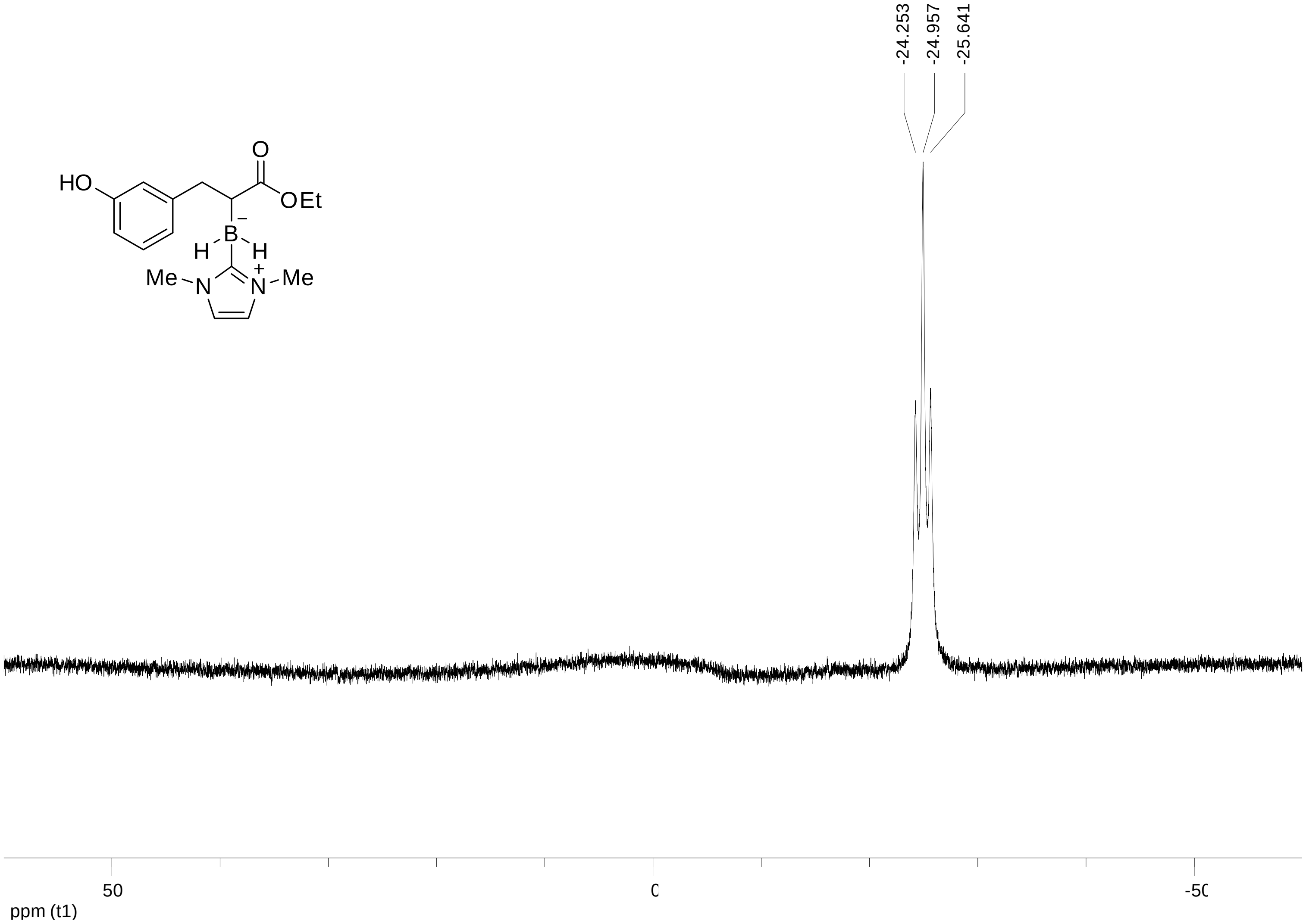

Supplementary Figure 61. ^{11}B NMR spectrum for 3d

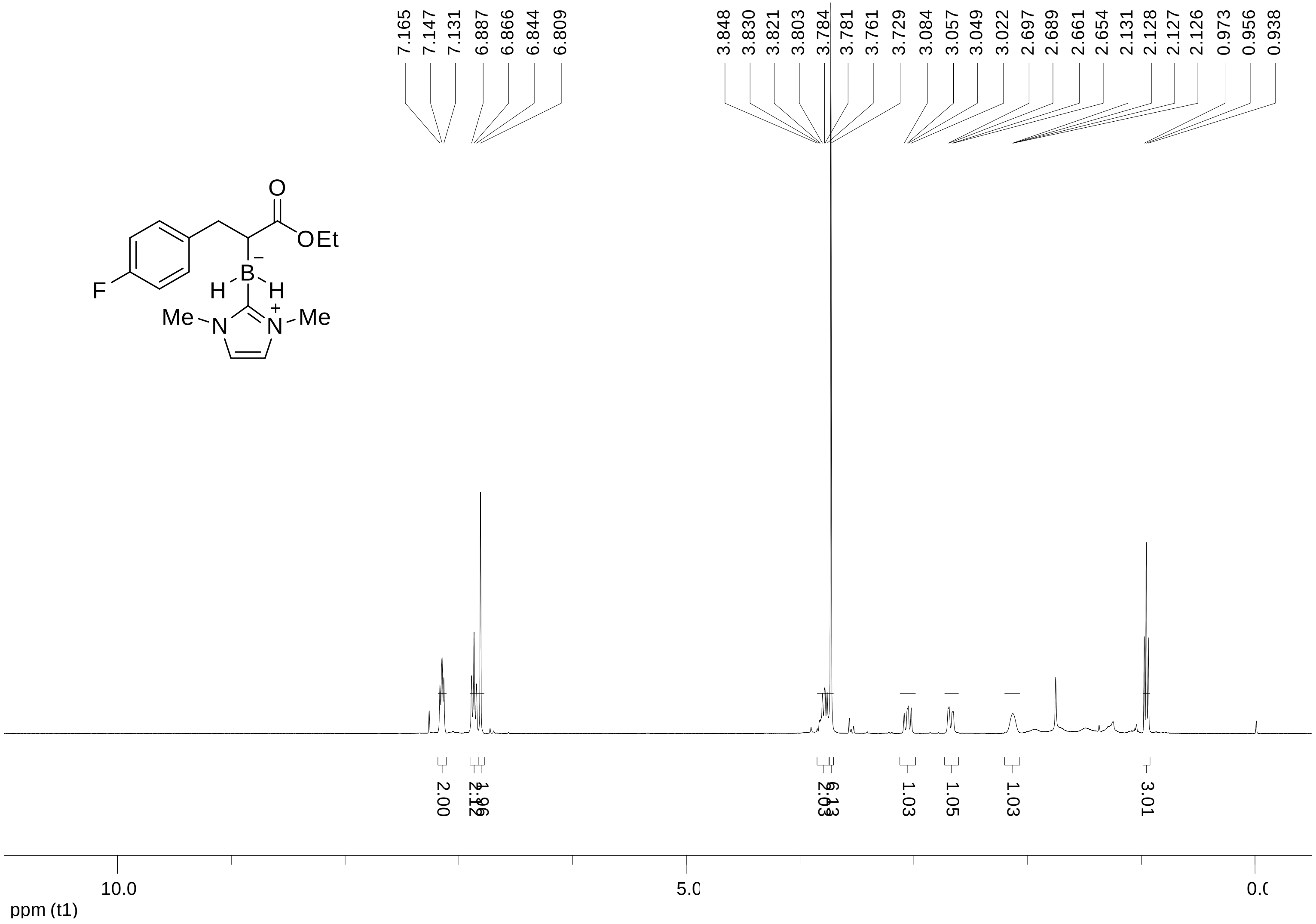

Supplementary Figure 62. ^{1}H NMR spectrum for 3e

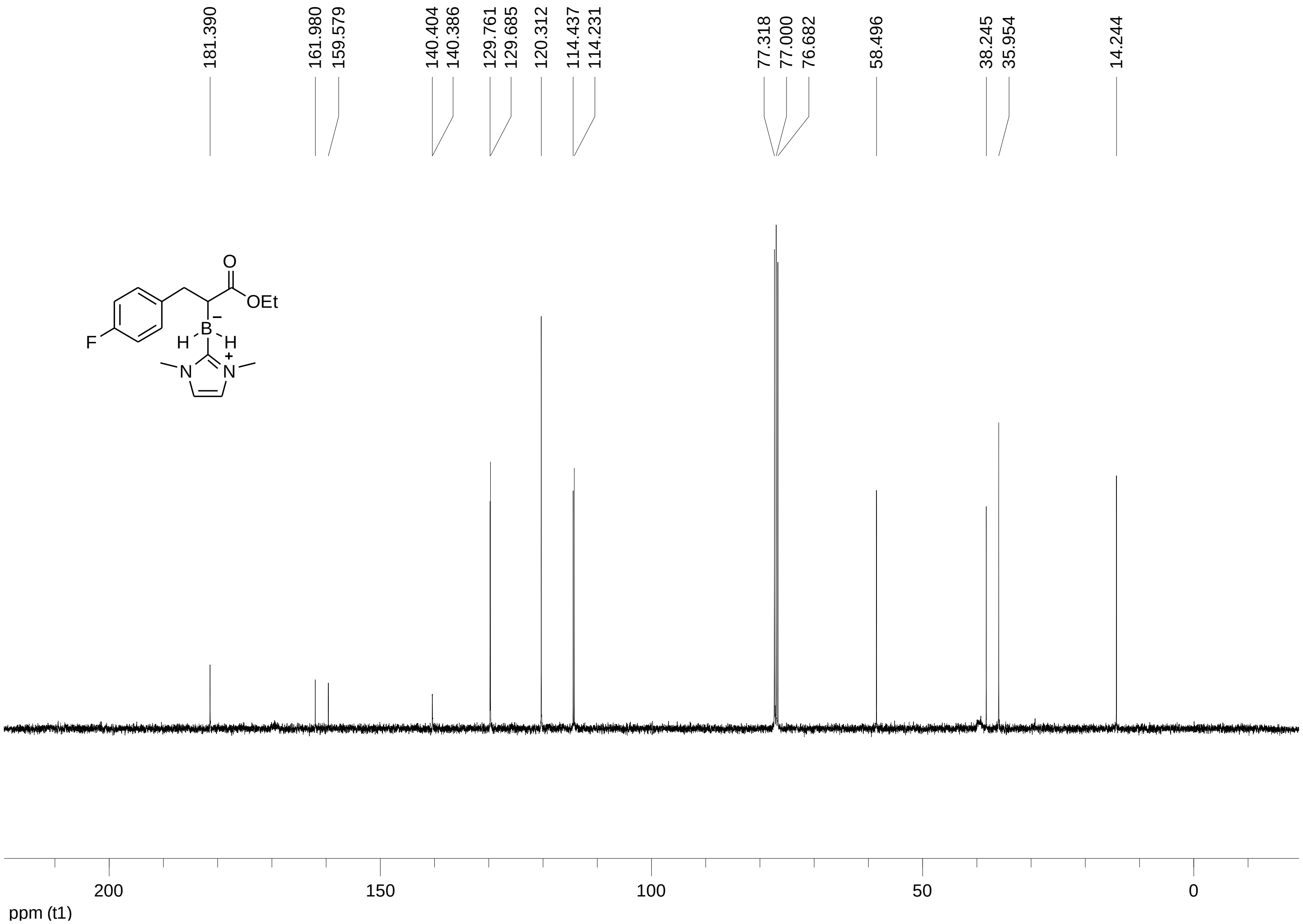

Supplementary Figure 63. ^{13}C NMR spectrum for 3e

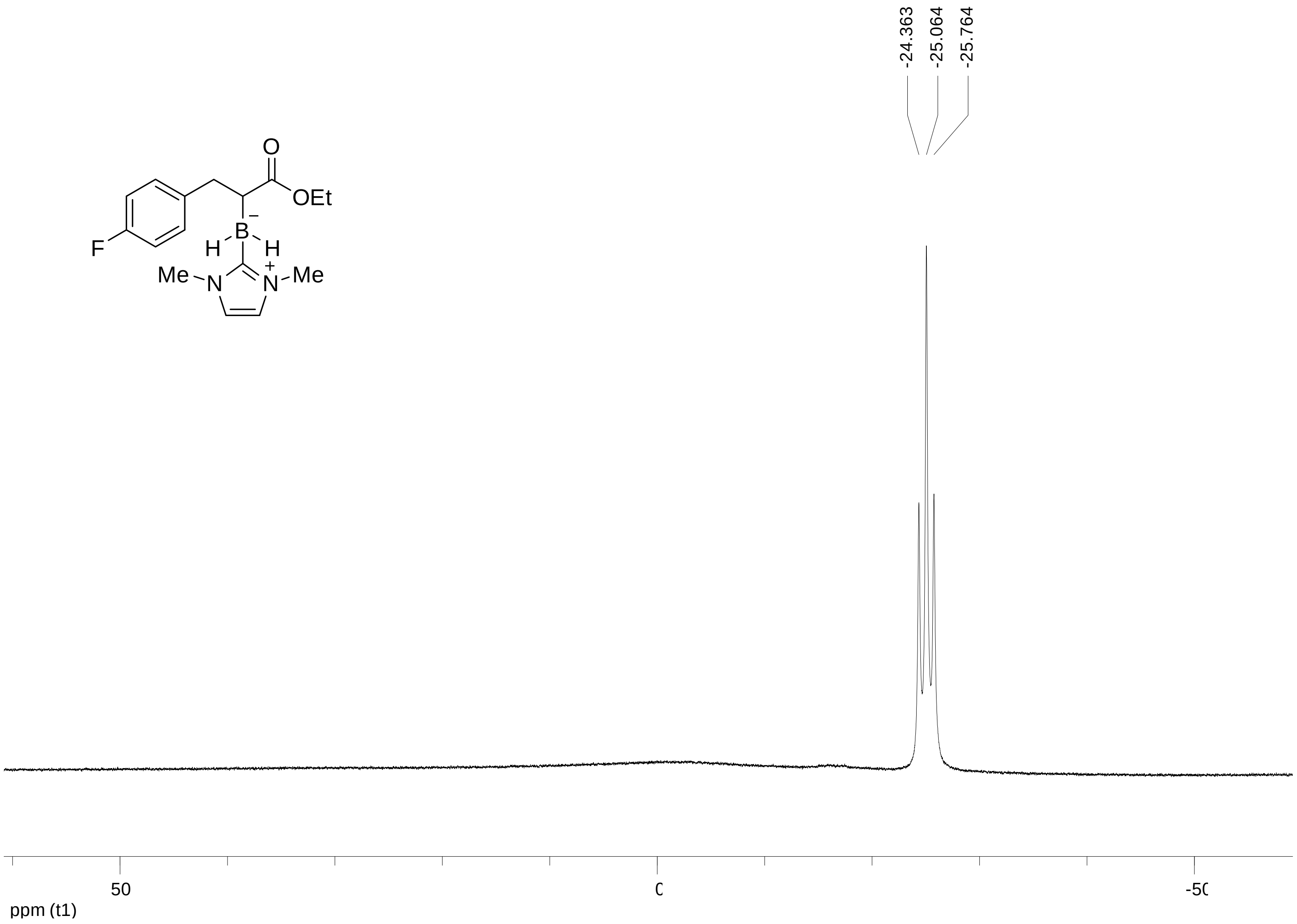

Supplementary Figure 64. ^{11}B NMR spectrum for 3e

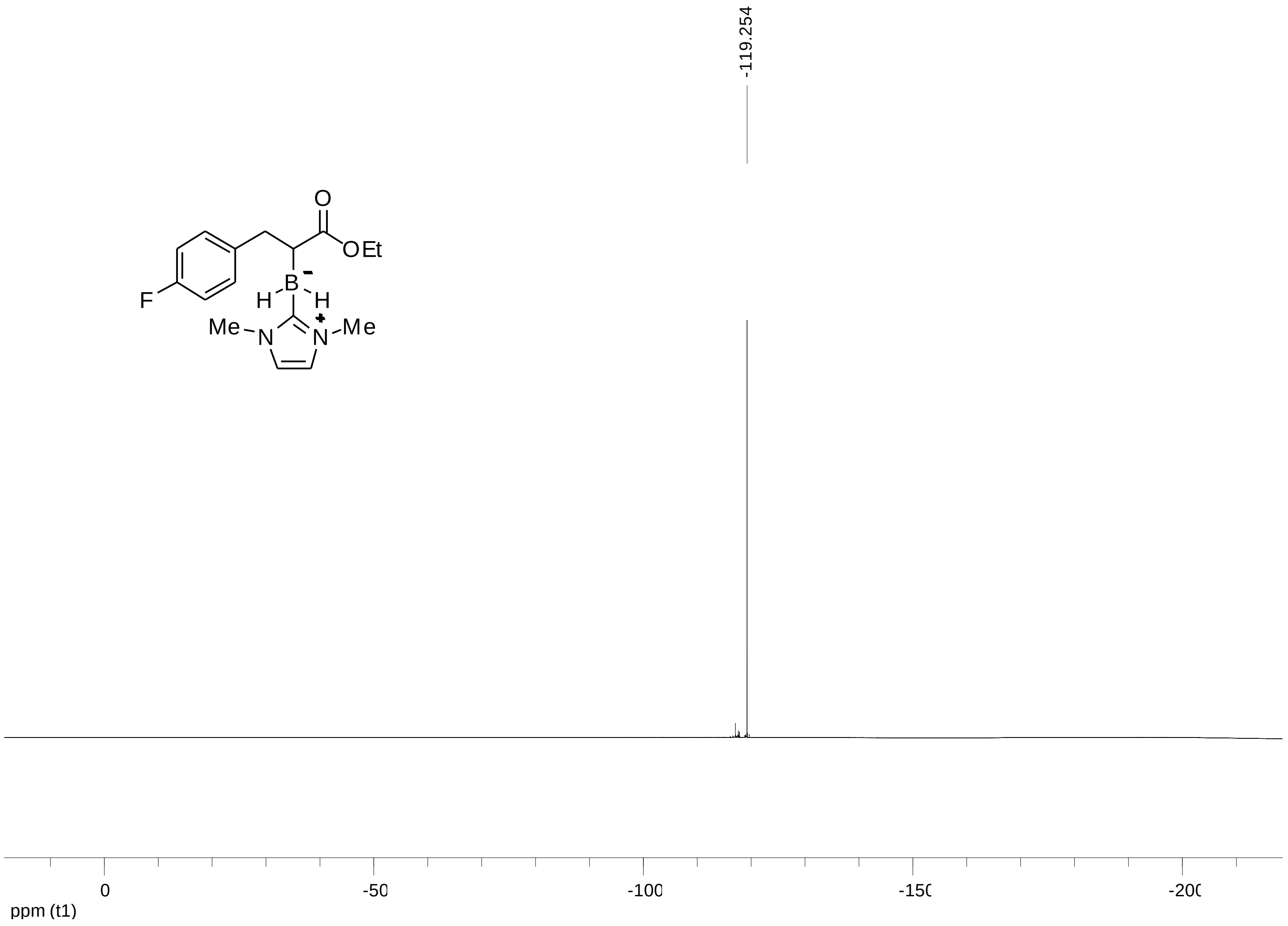

Supplementary Figure 65. ^{19}F NMR spectrum for 3e

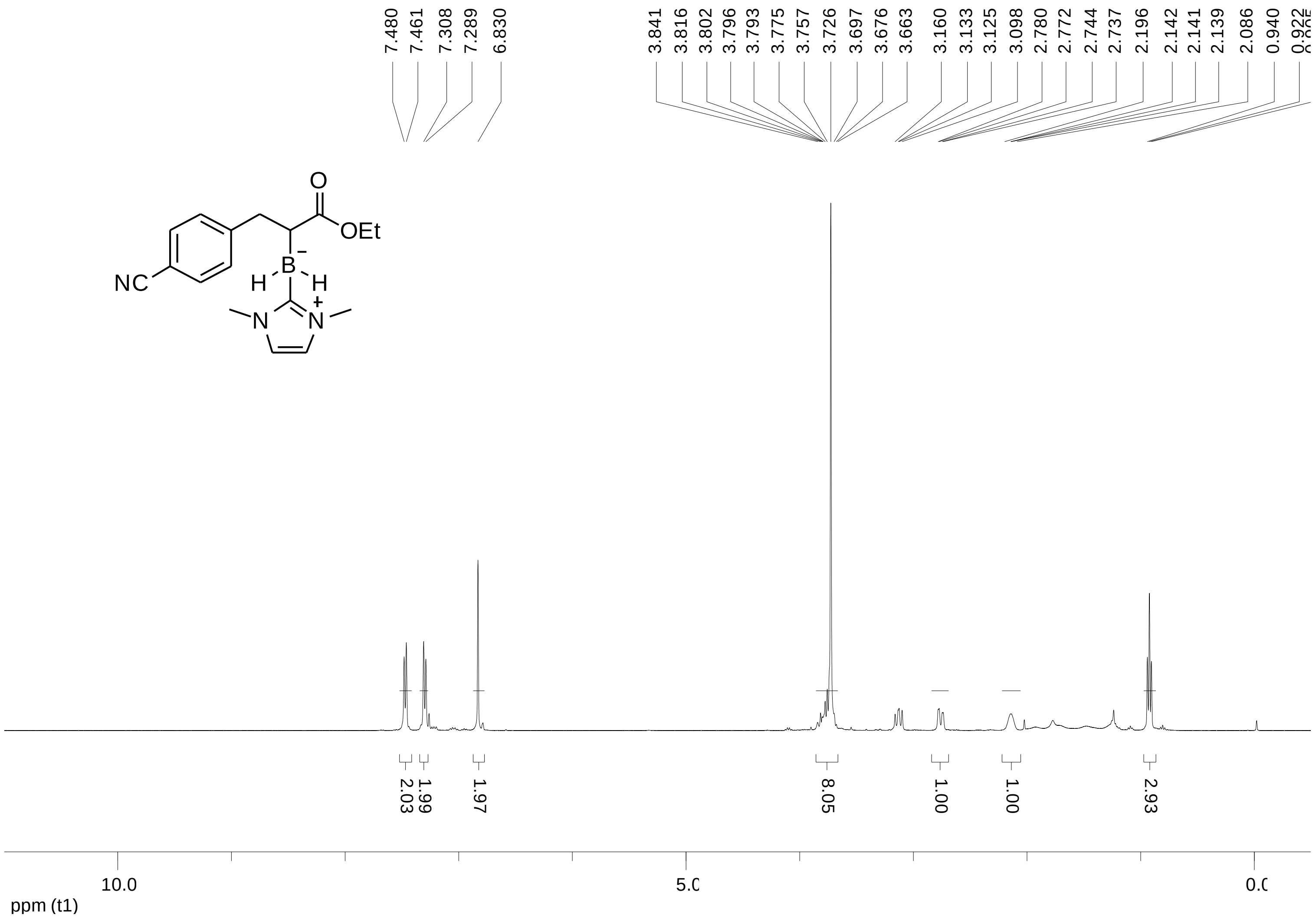

Supplementary Figure 66. ^{1}H NMR spectrum for 3f

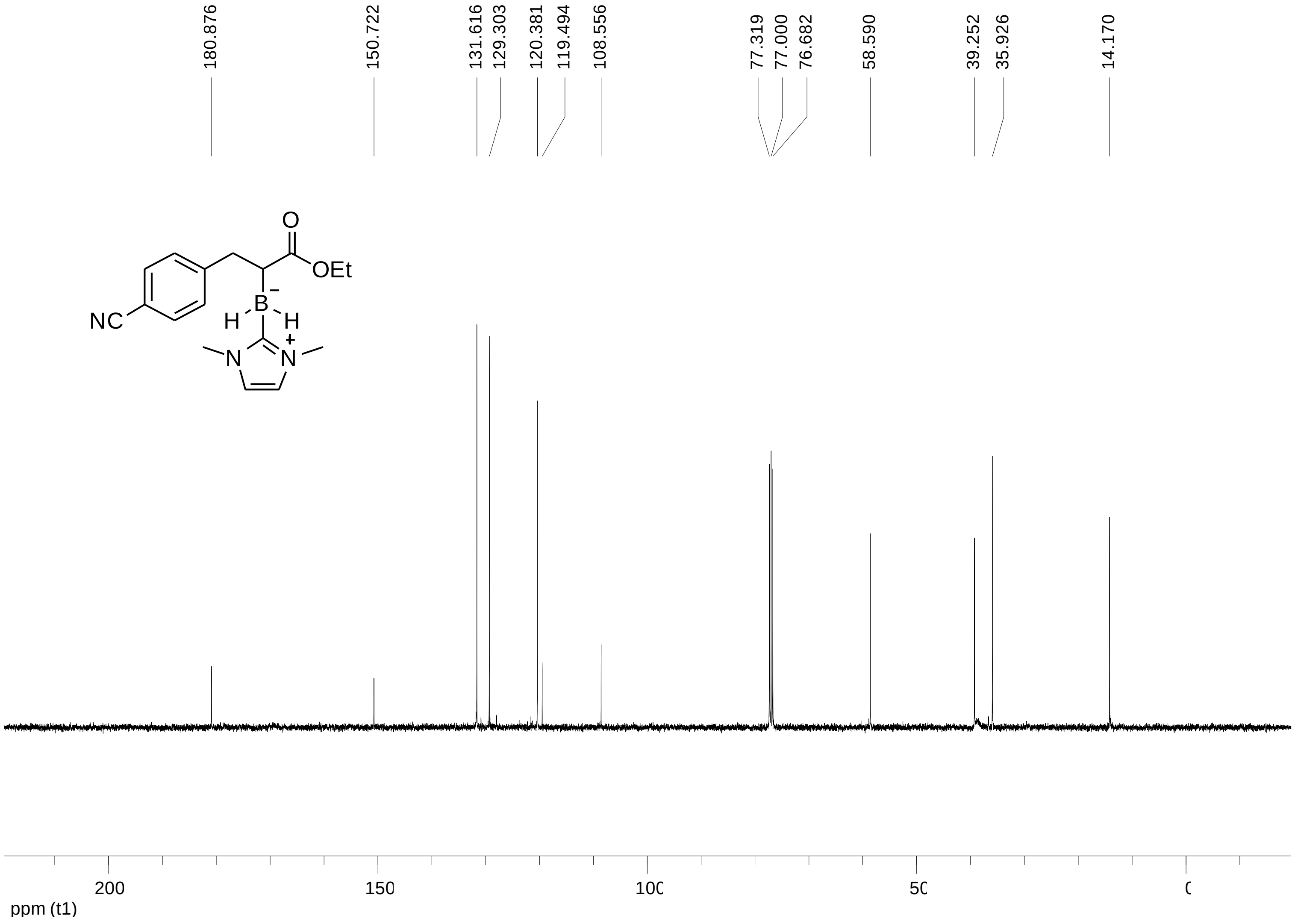

Supplementary Figure 67. ^{13}C NMR spectrum for 3f

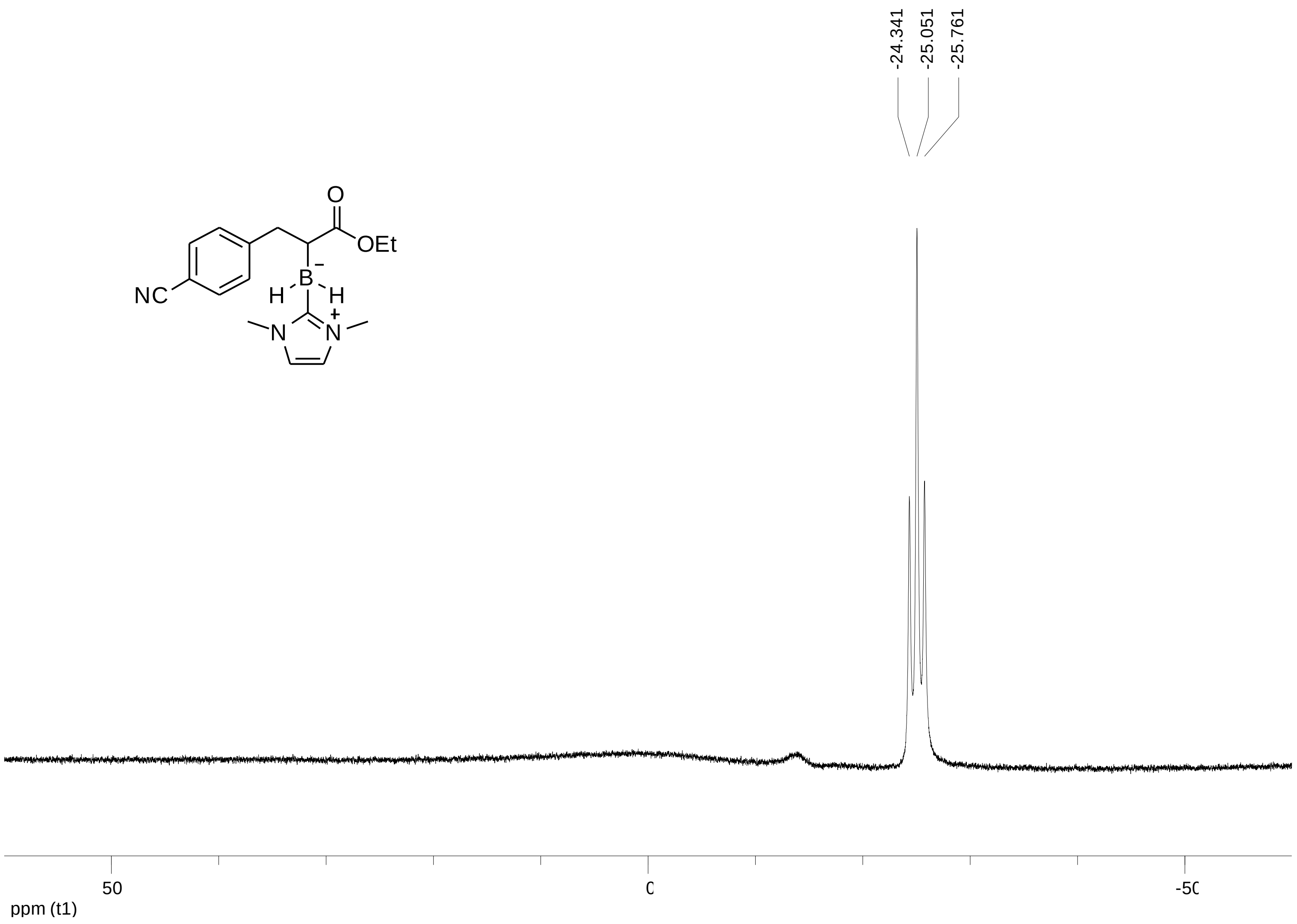

Supplementary Figure 68. ^{11}B NMR spectrum for 3f

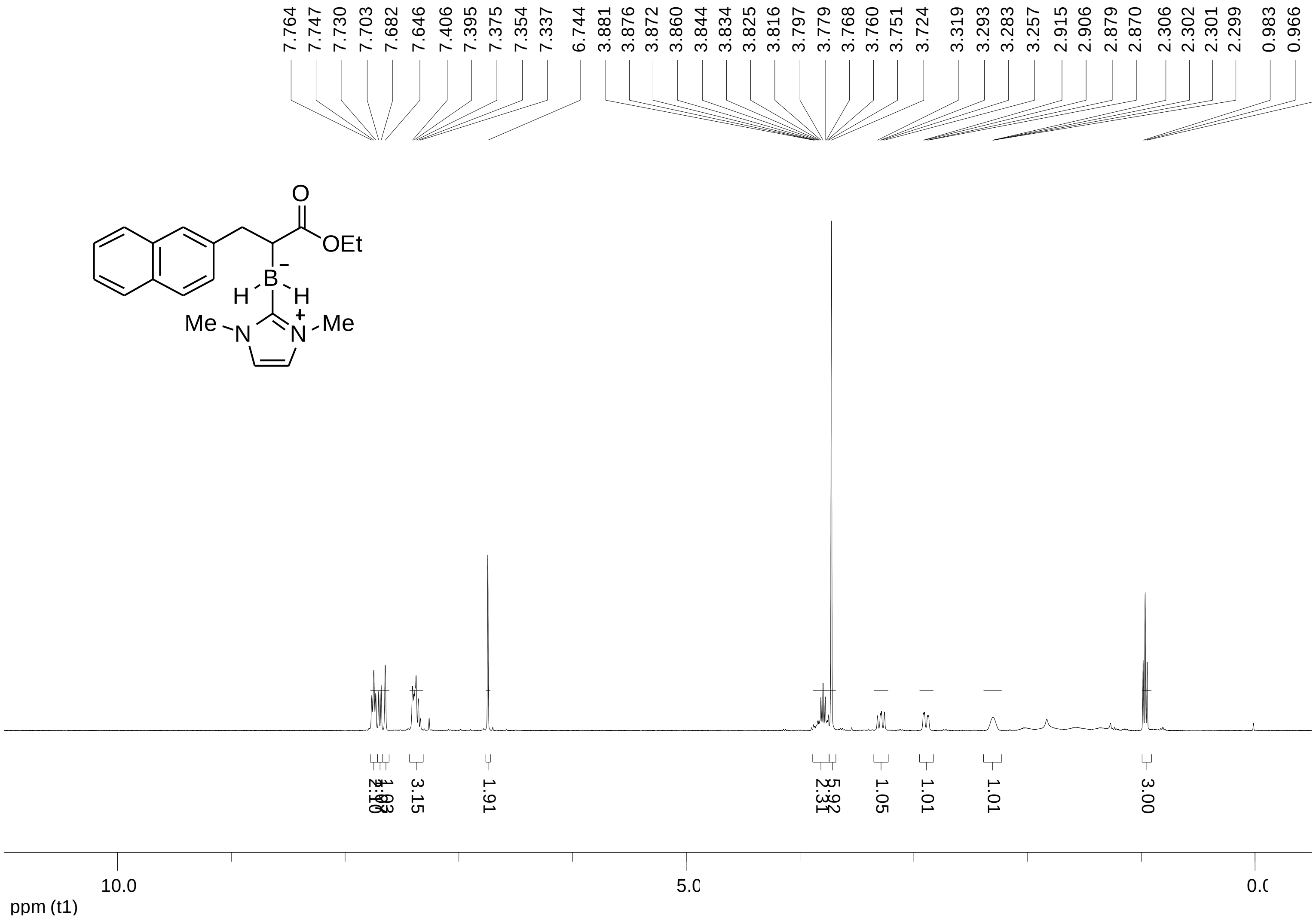

Supplementary Figure 69. ^{1}H NMR spectrum for 3g

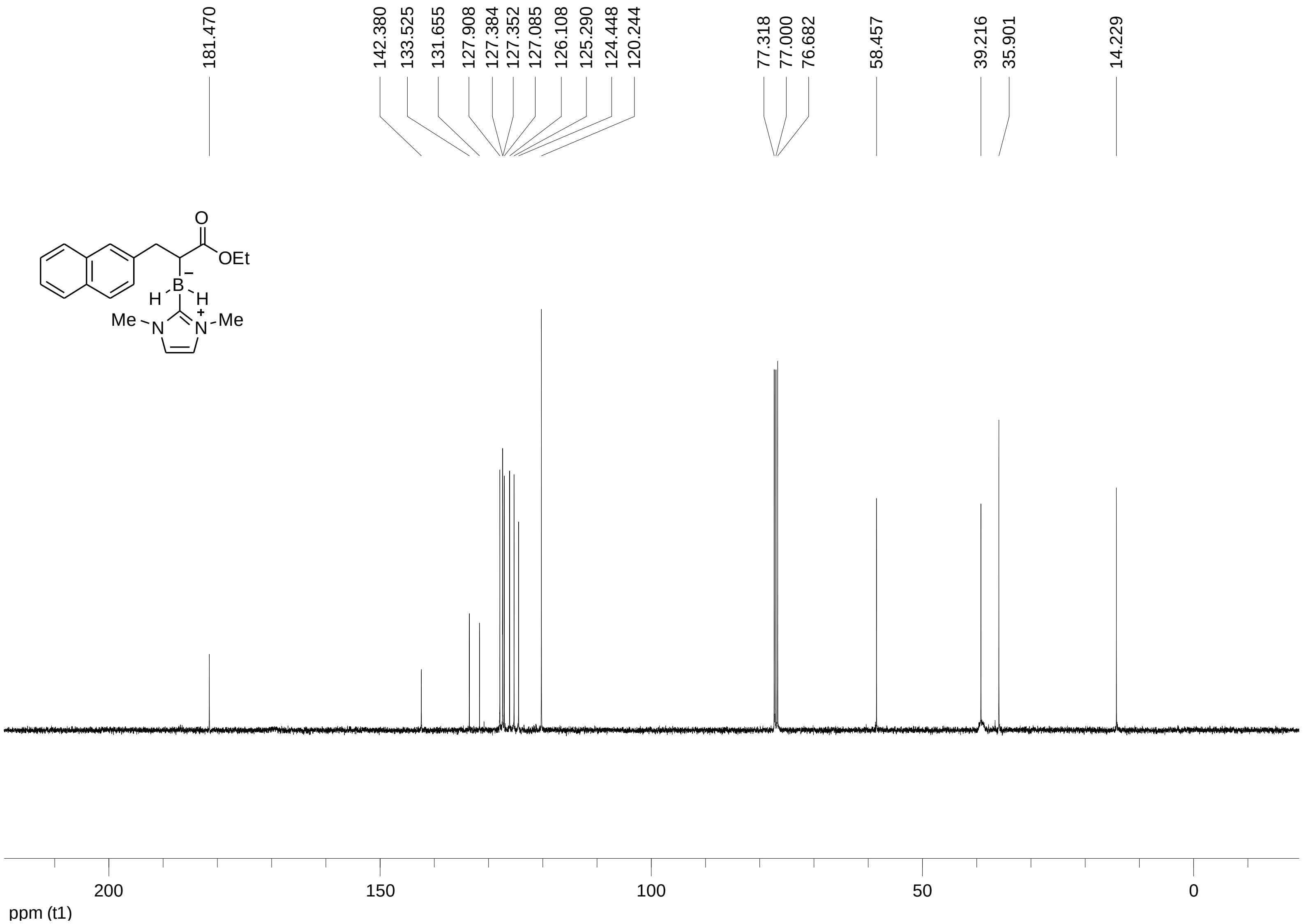

Supplementary Figure 70. ^{13}C NMR spectrum for 3g

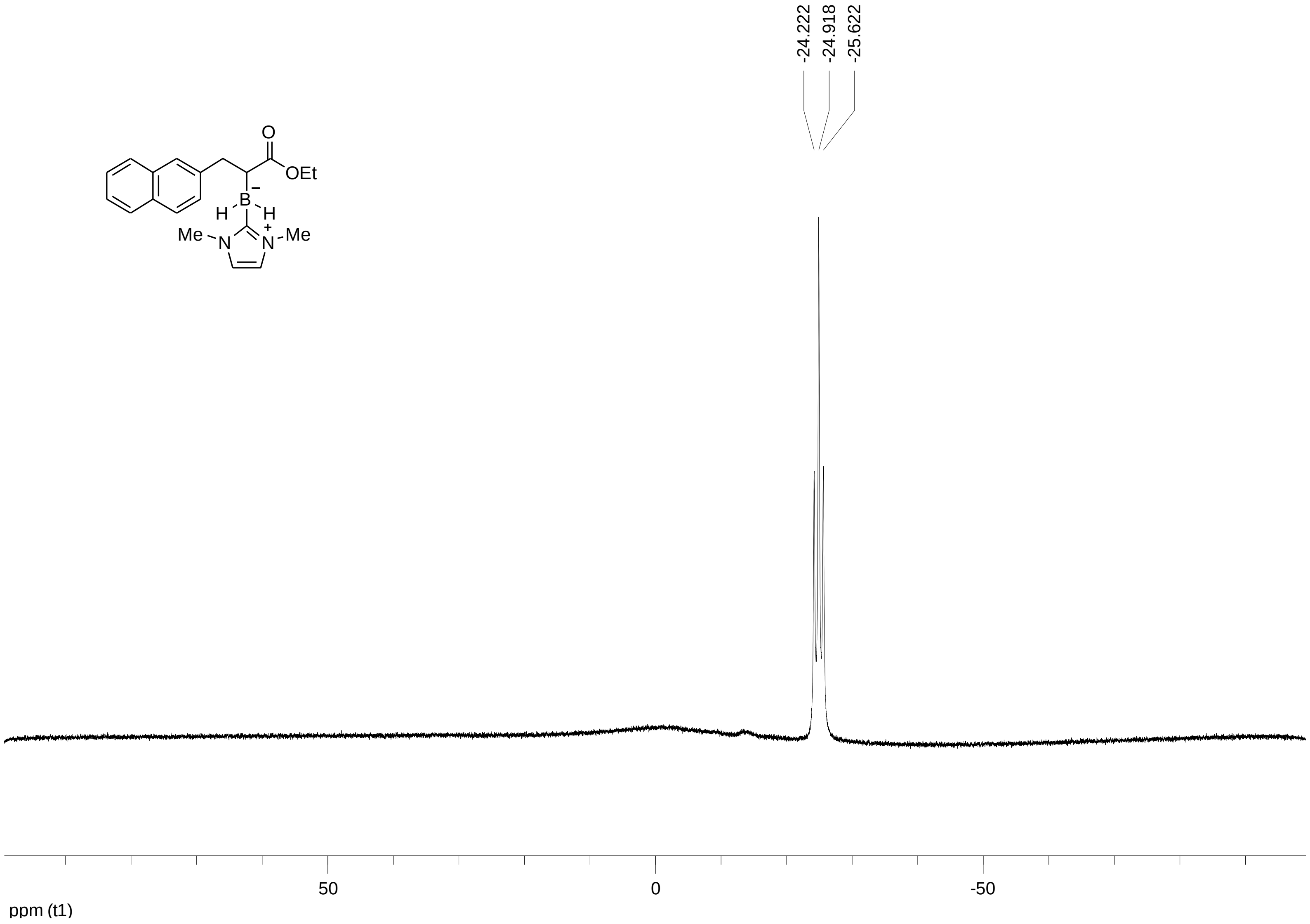

Supplementary Figure 71. ^{11}B NMR spectrum for 3g

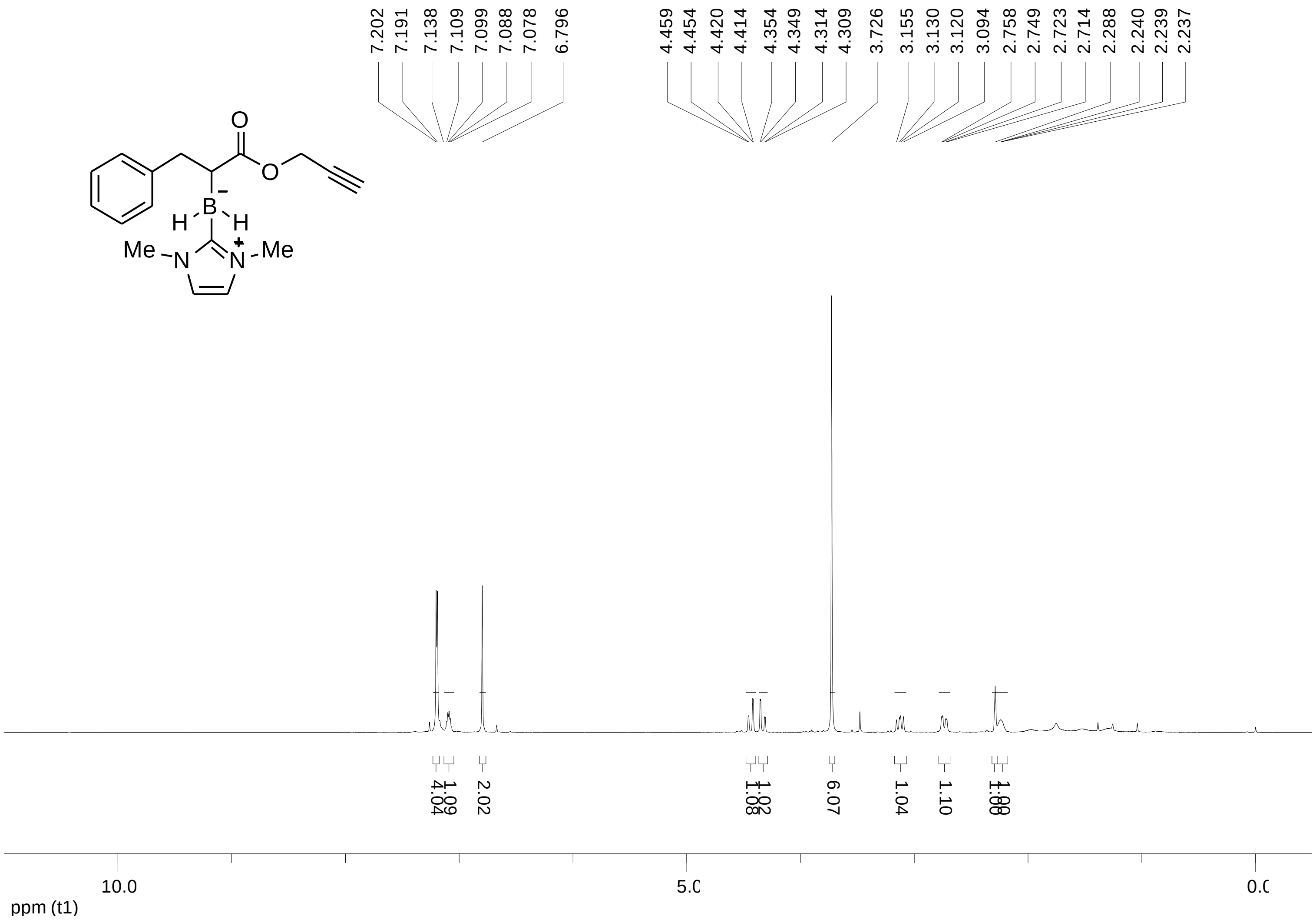

Supplementary Figure 72. ^{1}H NMR spectrum for 3h

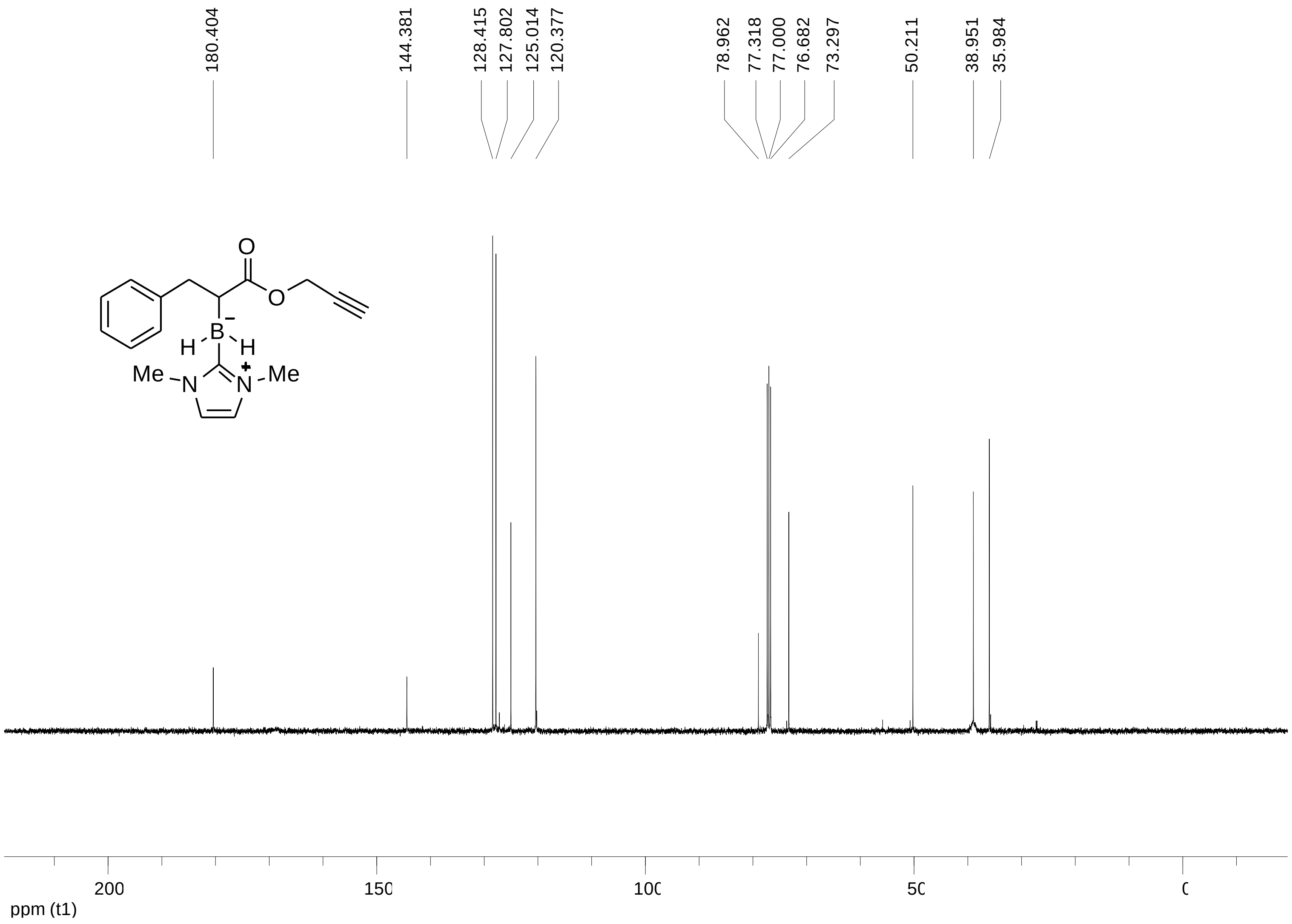

Supplementary Figure 73. ^{13}C NMR spectrum for 3h

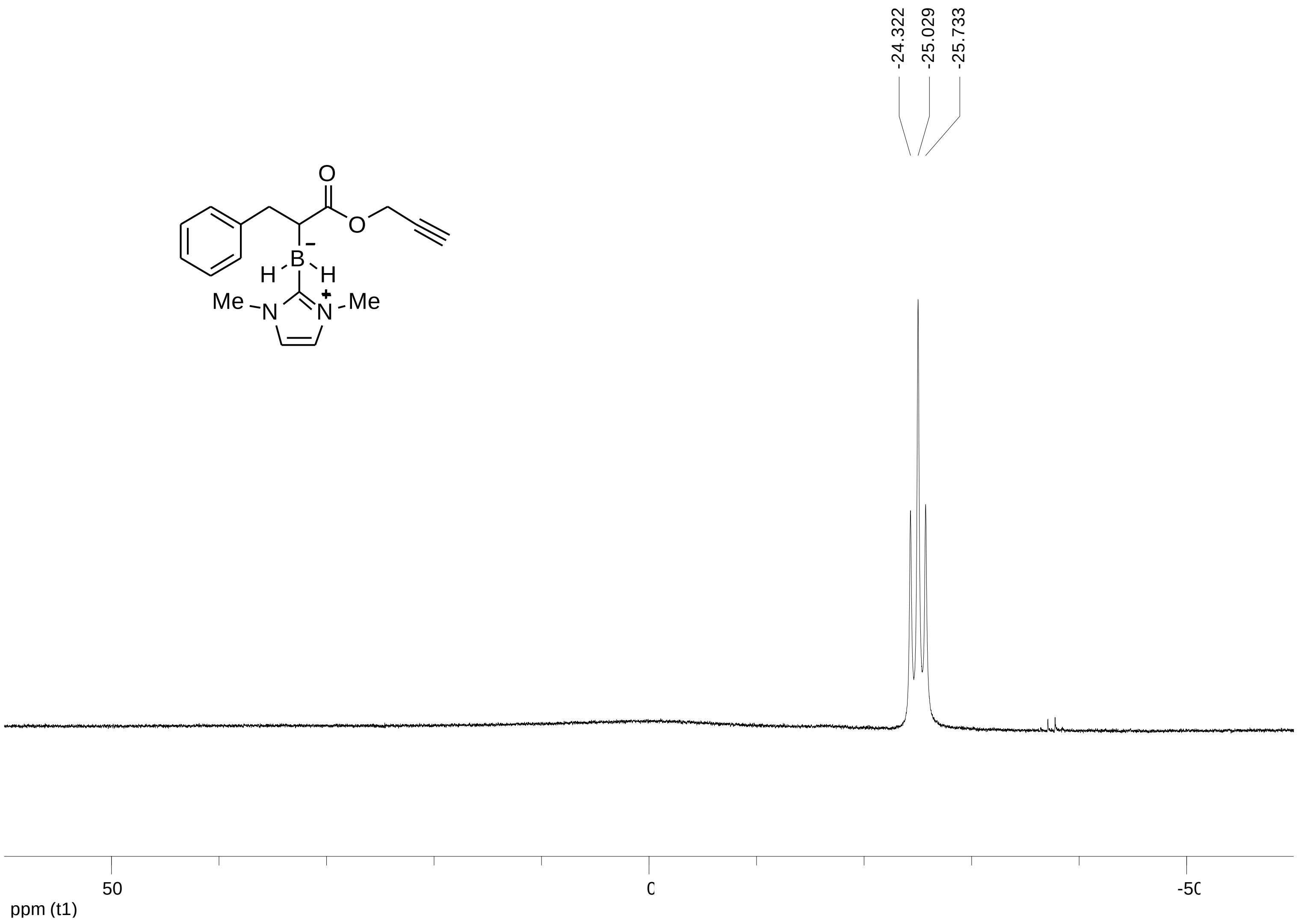

Supplementary Figure 74. ^{11}B NMR spectrum for 3h

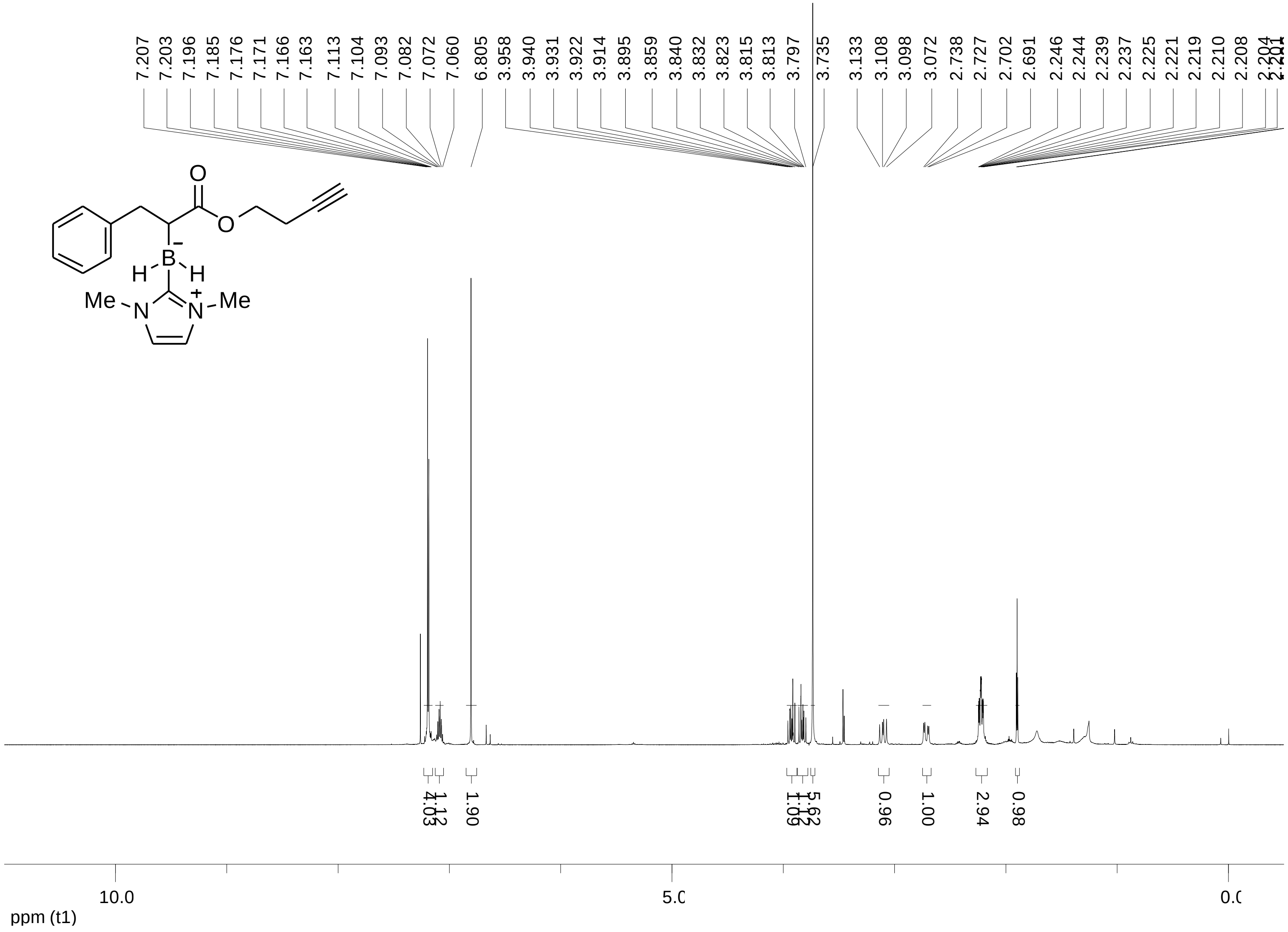

Supplementary Figure 75. ^{1}H NMR spectrum for 3i

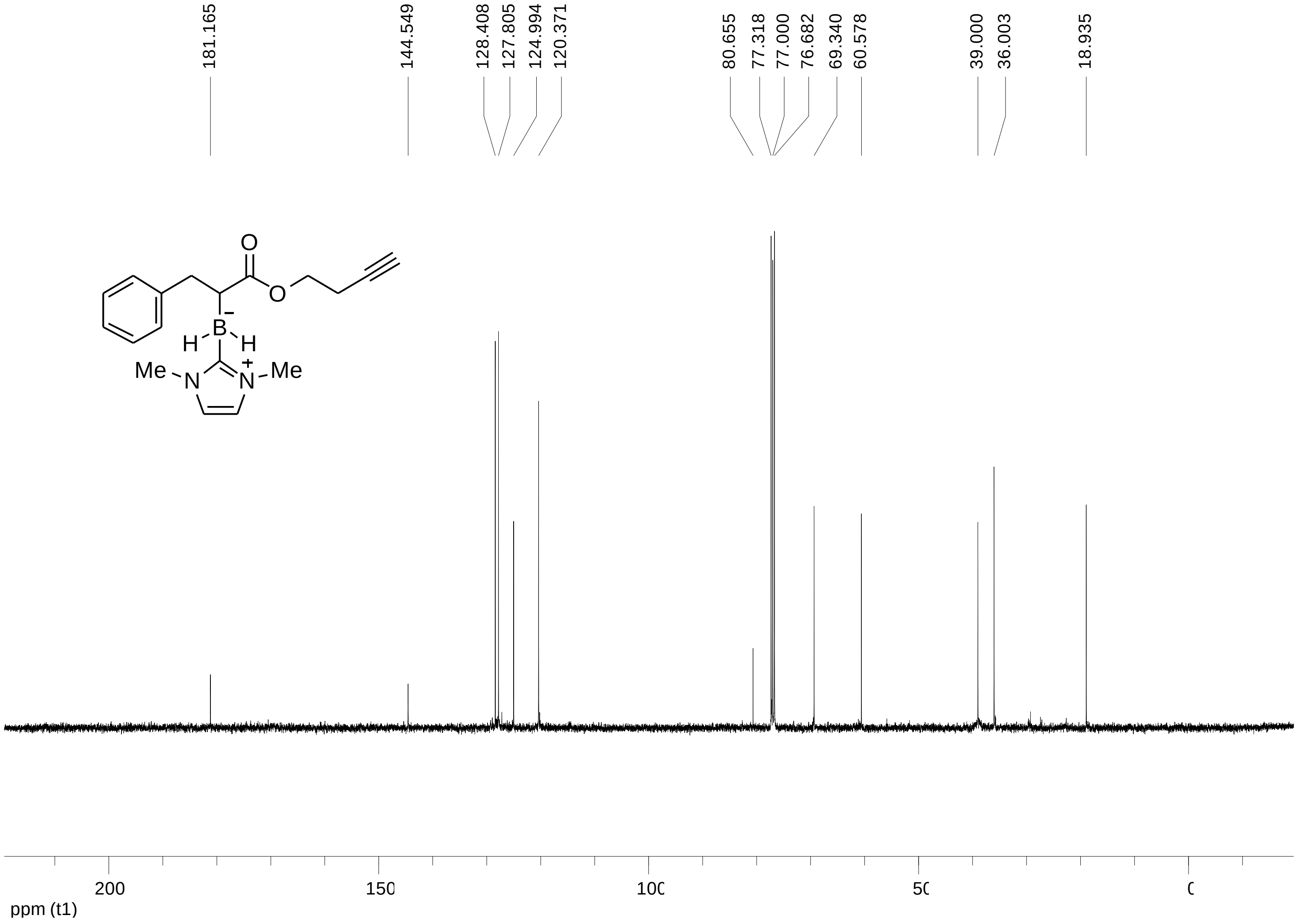

Supplementary Figure 76. ^{13}C NMR spectrum for 3i

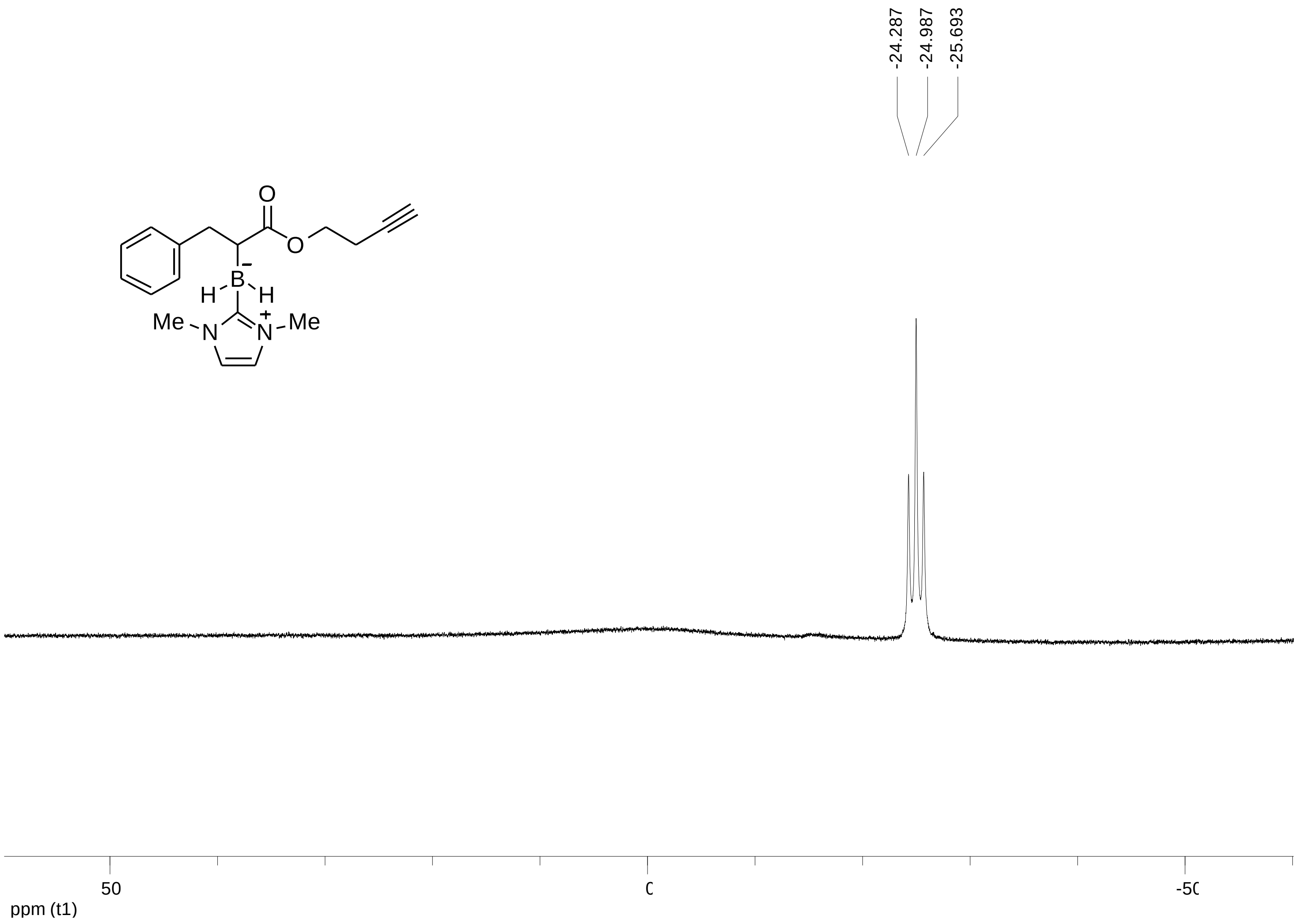

Supplementary Figure 77. ^{11}B NMR spectrum for 3i

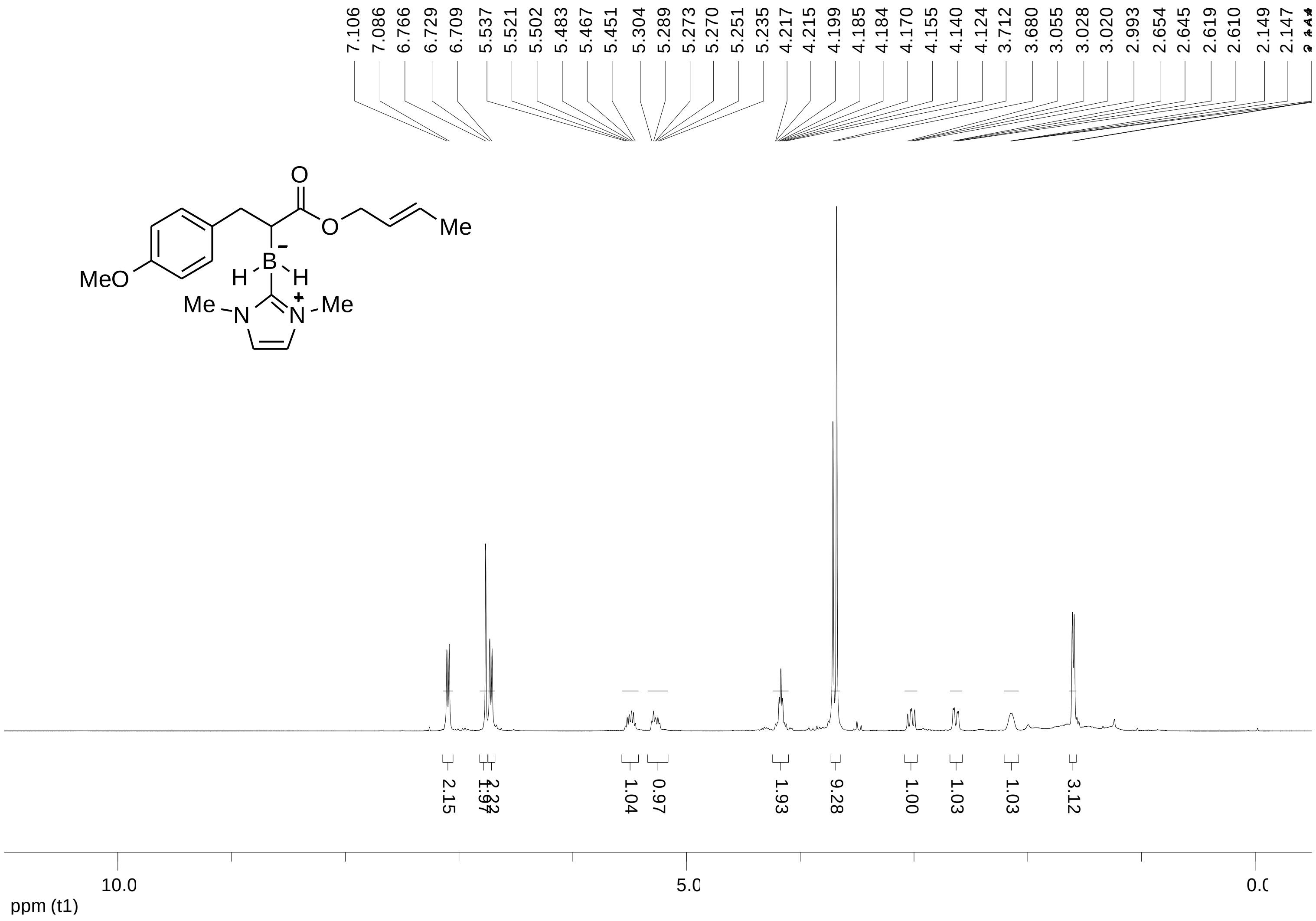

Supplementary Figure 78. ^{1}H NMR spectrum for 3j

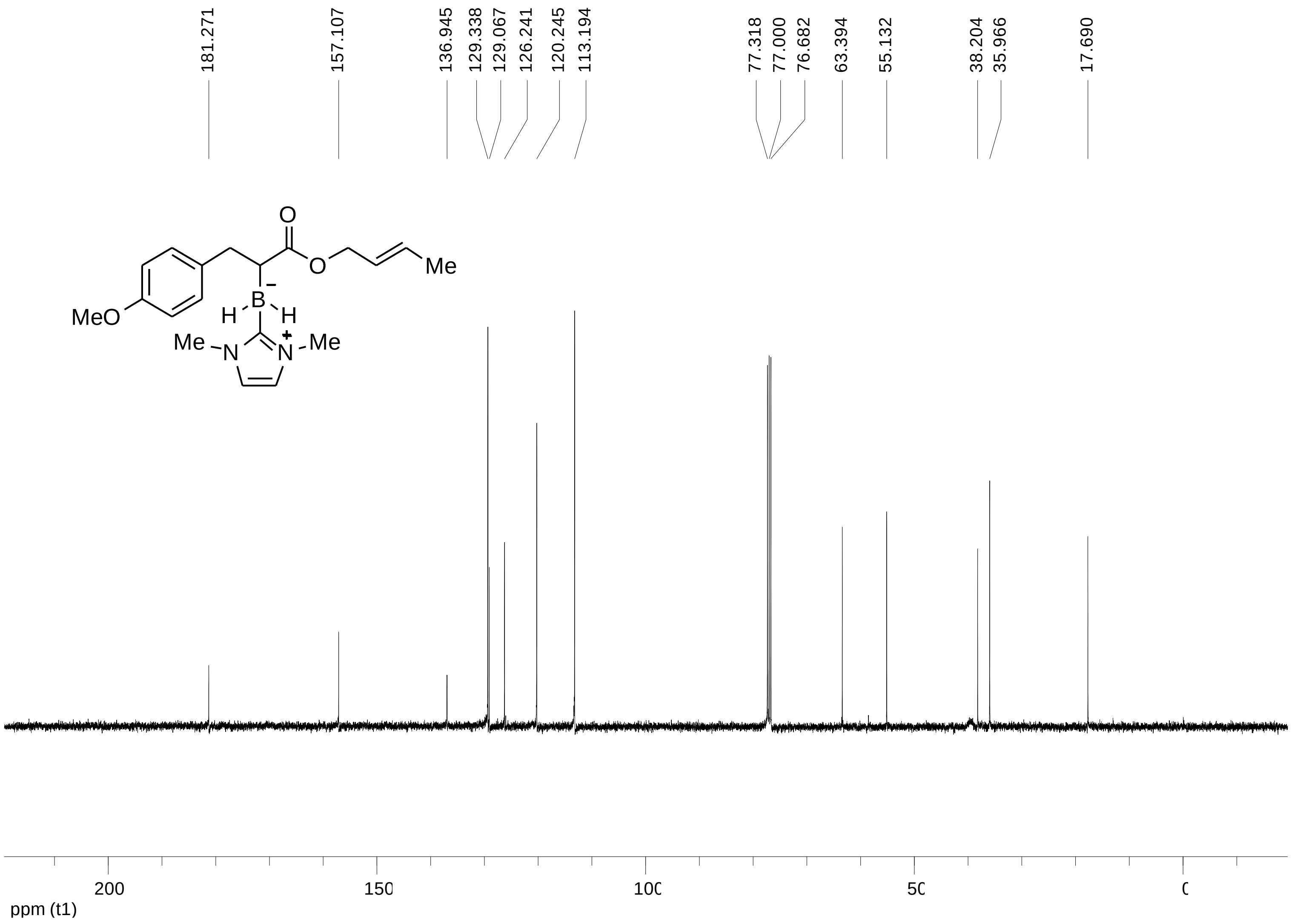

Supplementary Figure 79. ^{13}C NMR spectrum for 3j

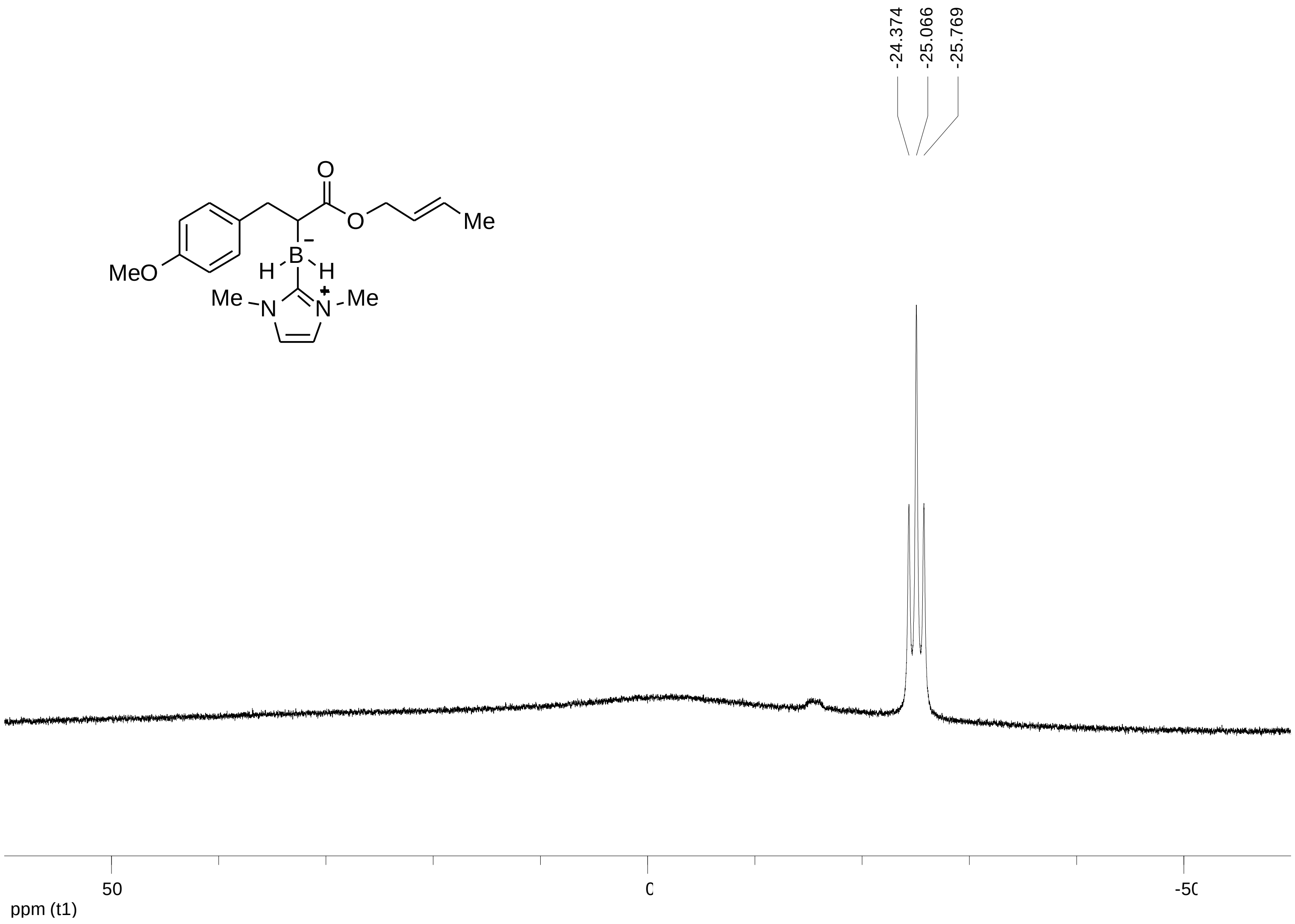

Supplementary Figure 80. ^{11}B NMR spectrum for 3j

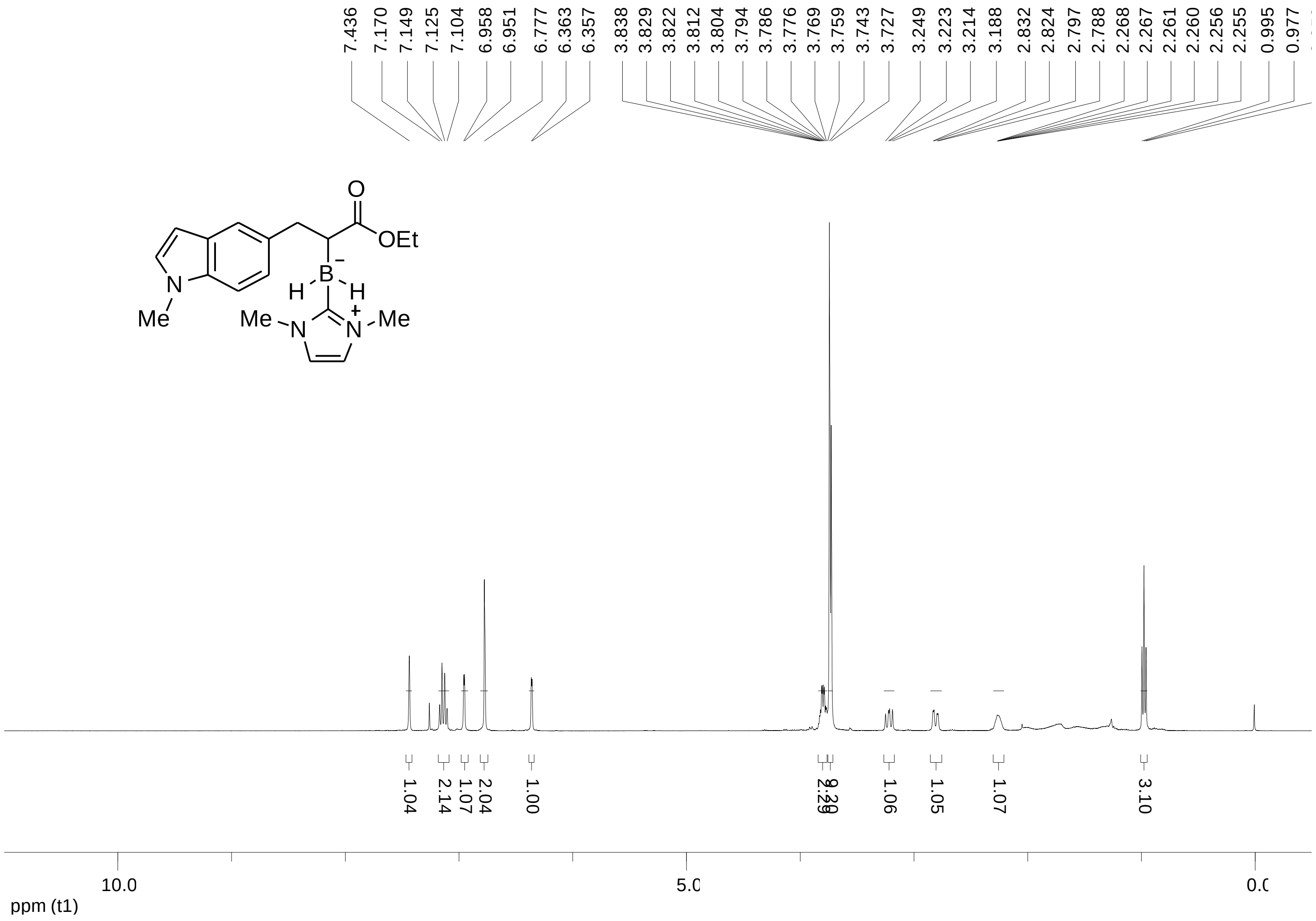

Supplementary Figure 81. ^{1}H NMR spectrum for 3k

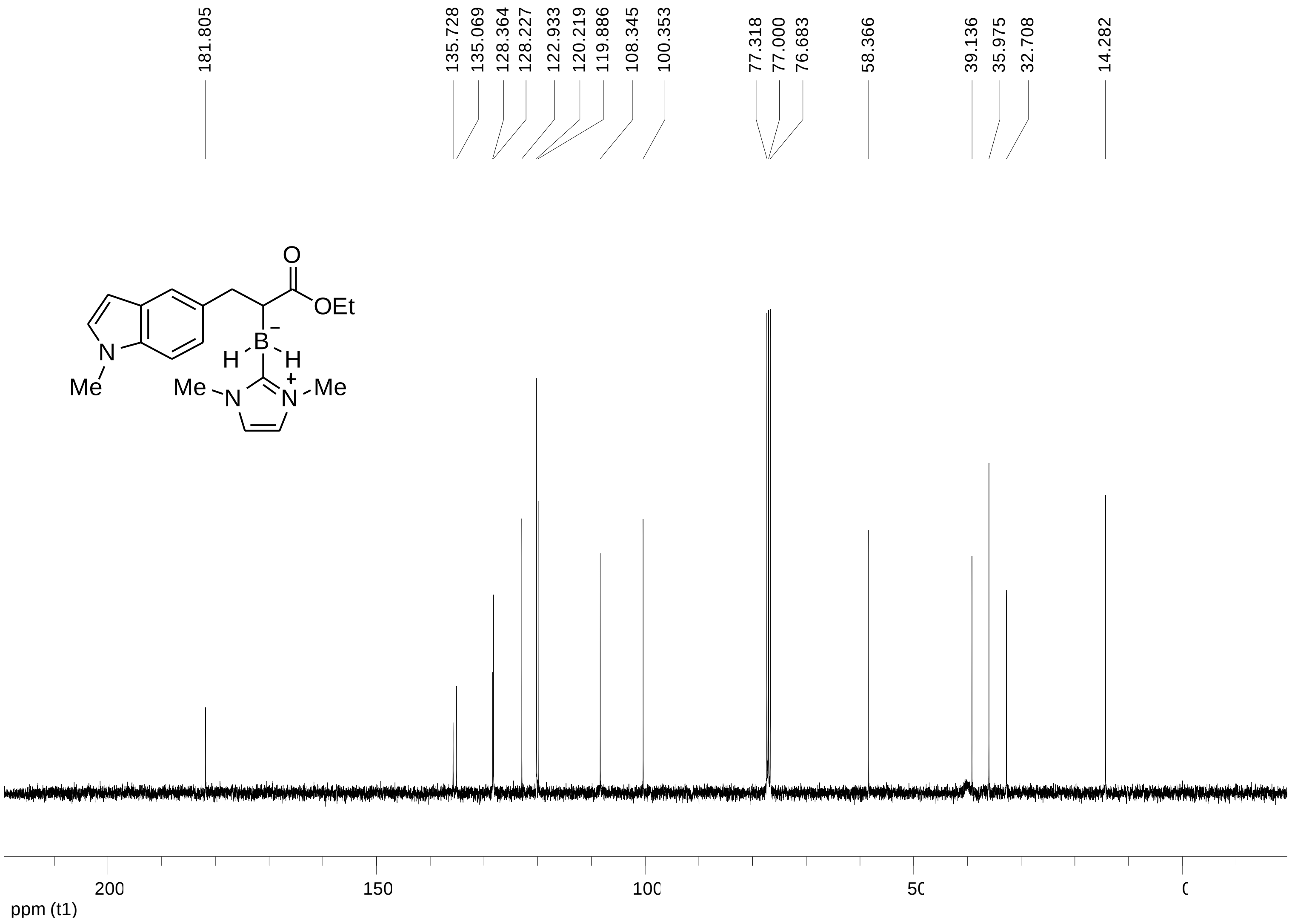

Supplementary Figure 82. ^{13}C NMR spectrum for 3k

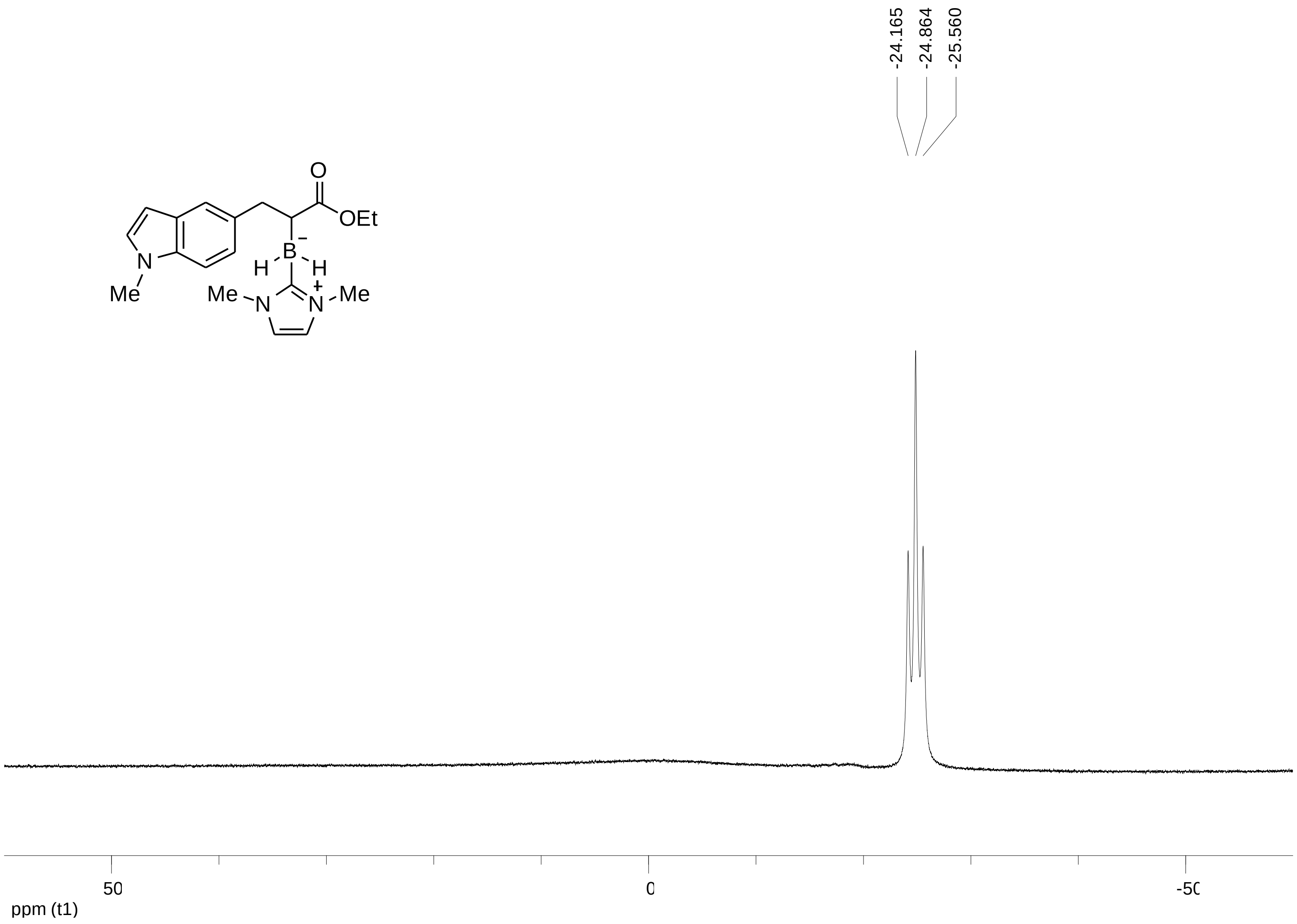

Supplementary Figure 83. ^{11}B NMR spectrum for 3k

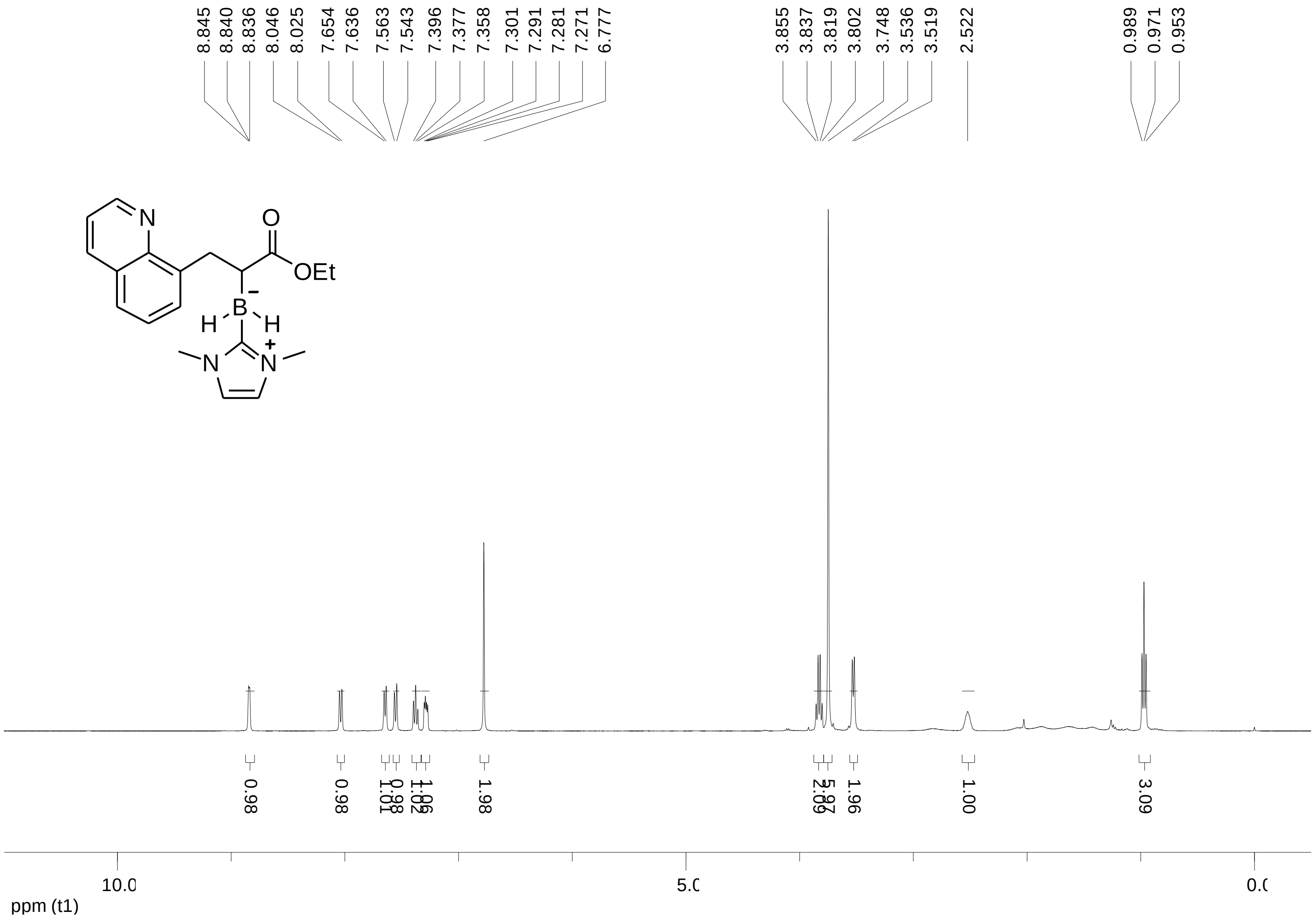

Supplementary Figure 84. ^{1}H NMR spectrum for 3l

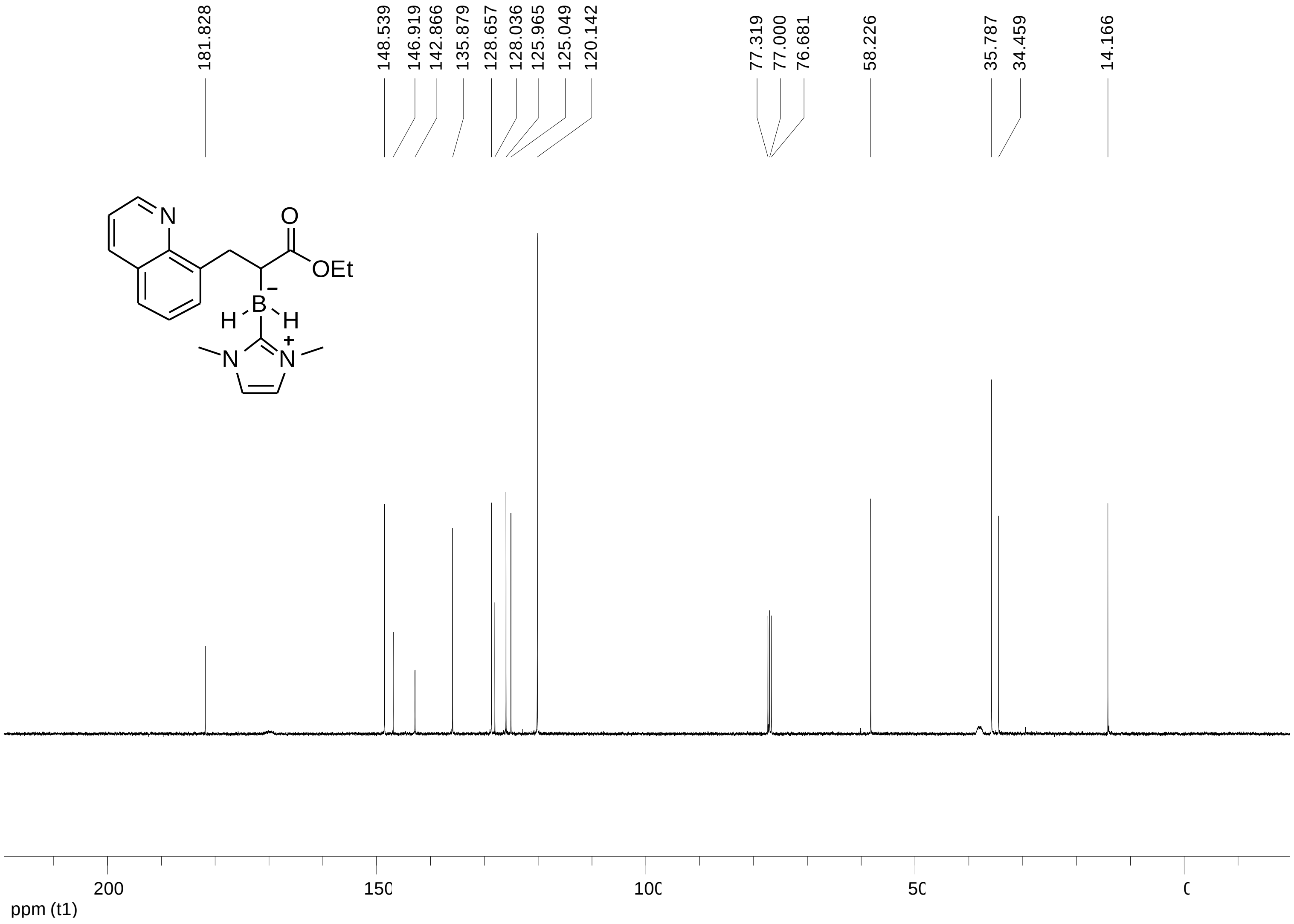

Supplementary Figure 85. ^{13}C NMR spectrum for 3l

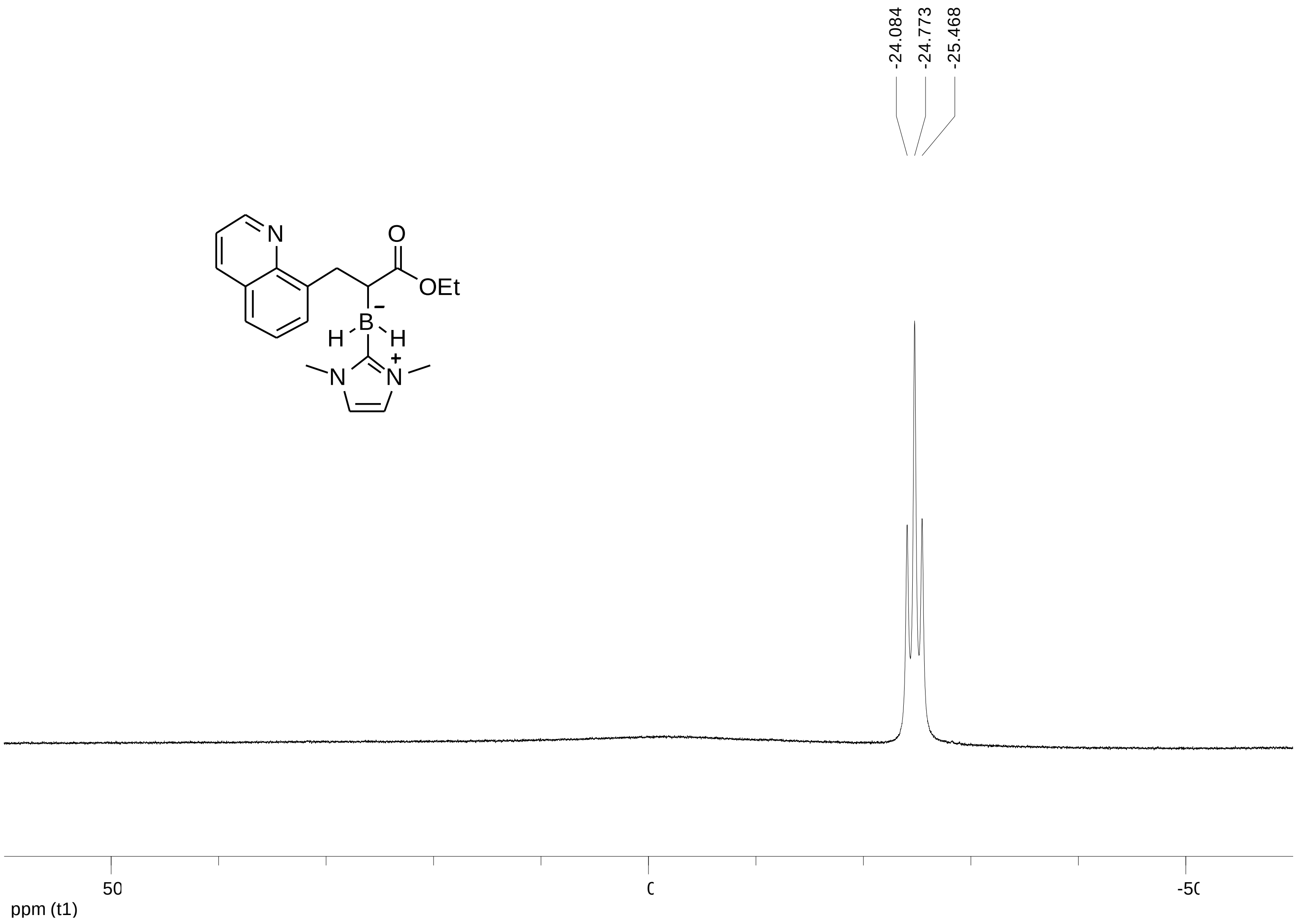

Supplementary Figure 86. ^{11}B NMR spectrum for 3l

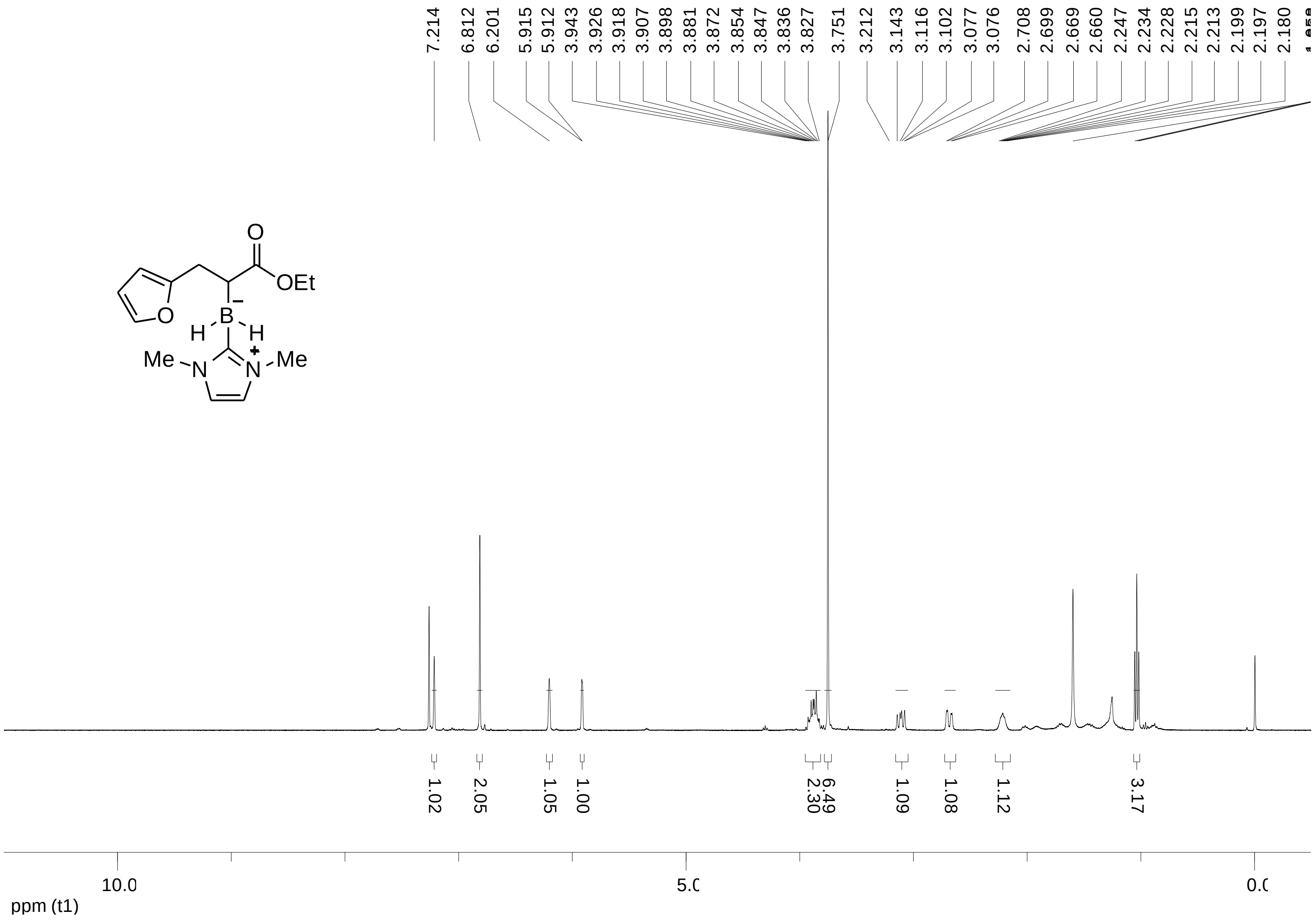

Supplementary Figure 87. ^{1}H NMR spectrum for 3m

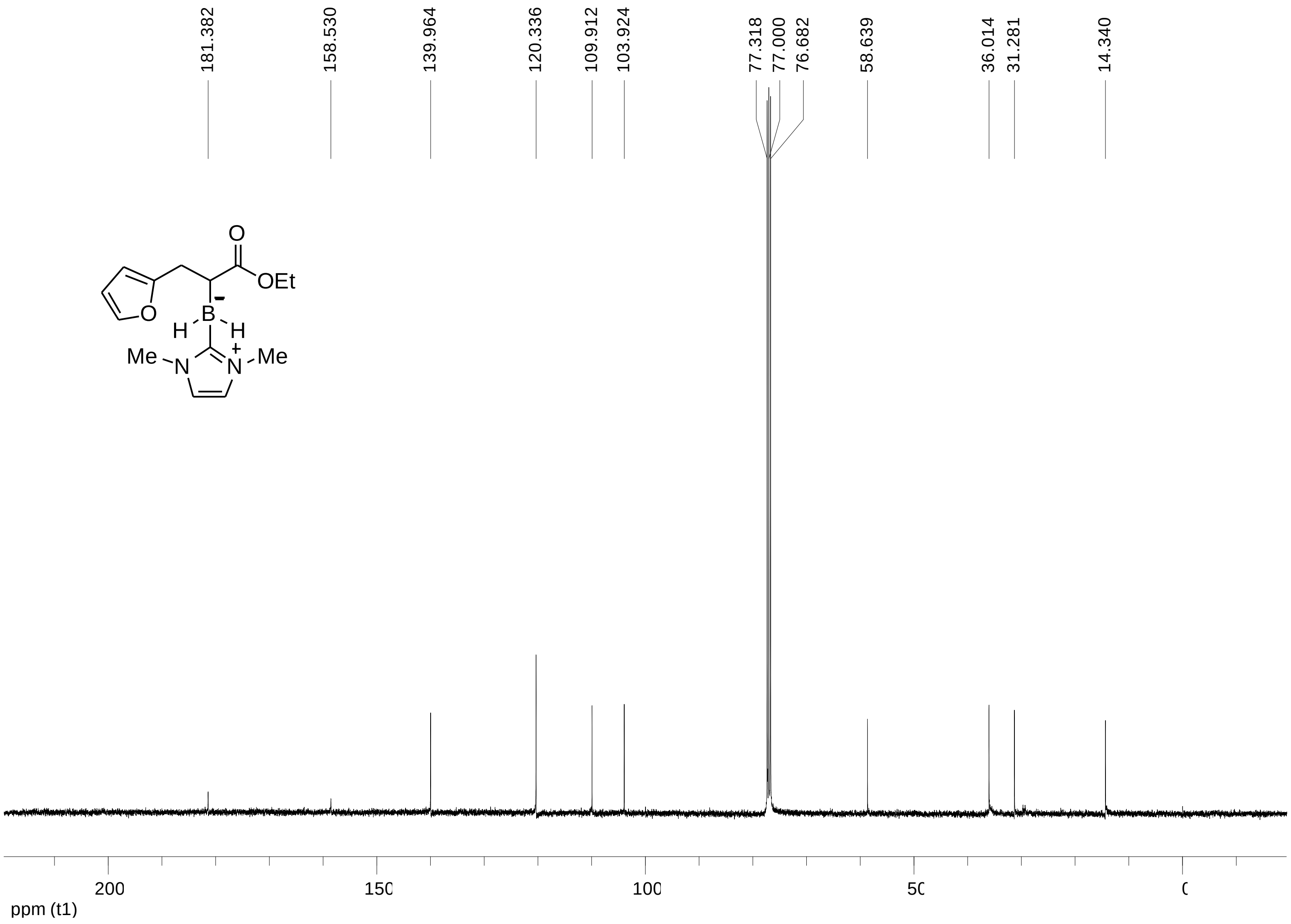

Supplementary Figure 88. ^{13}C NMR spectrum for 3m

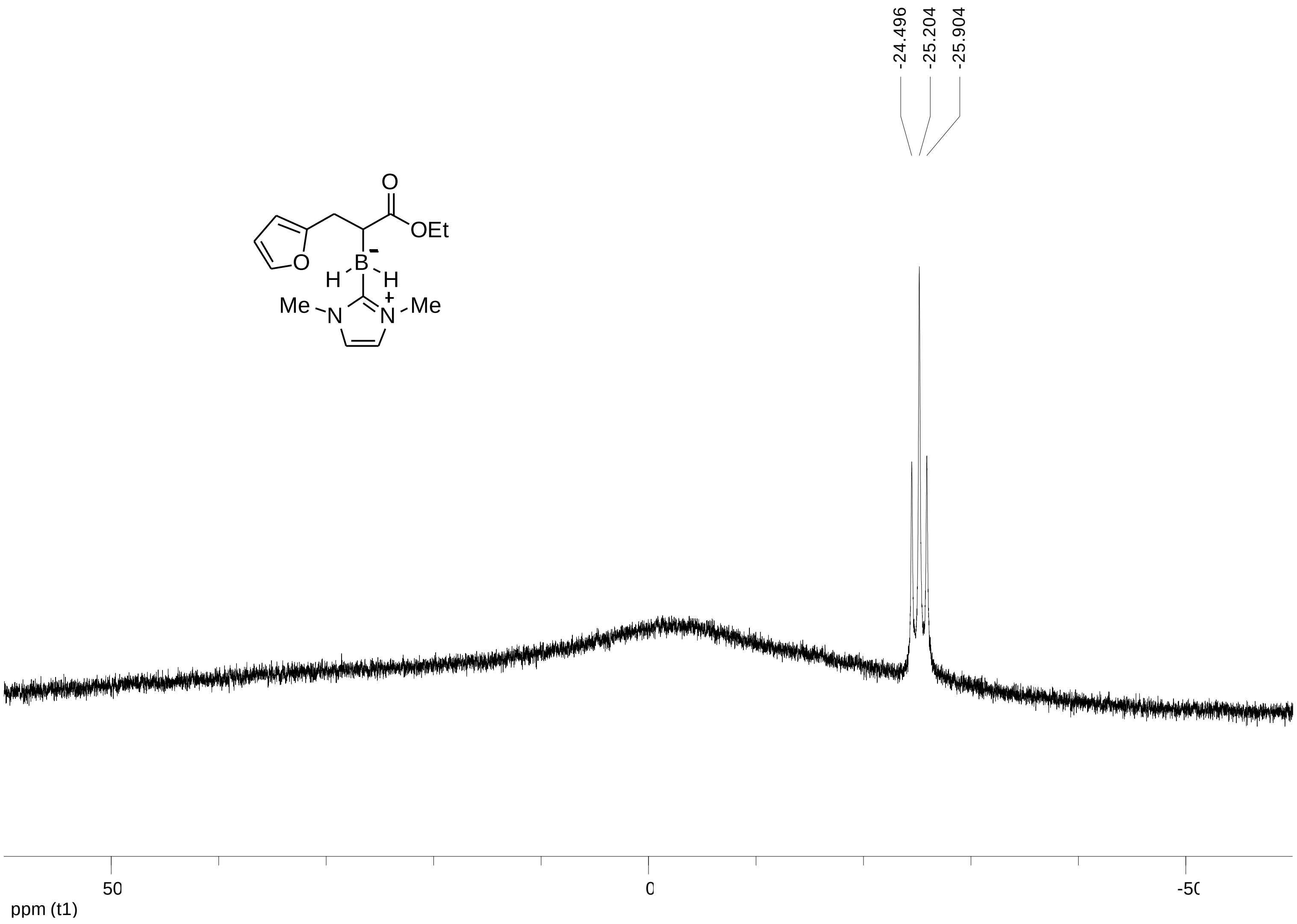

Supplementary Figure 89. ^{11}B NMR spectrum for 3m

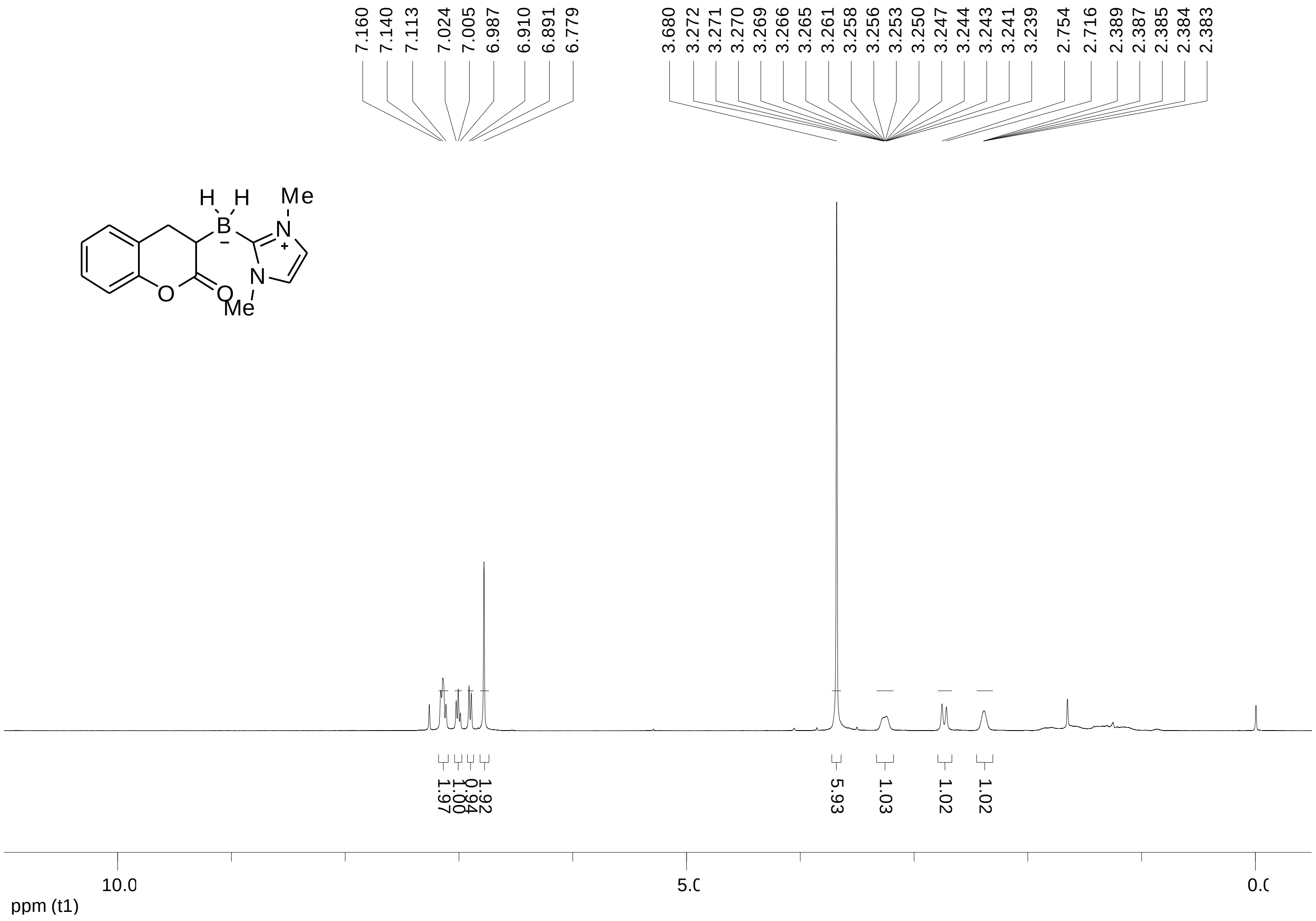

Supplementary Figure 90. ^{1}H NMR spectrum for 3n

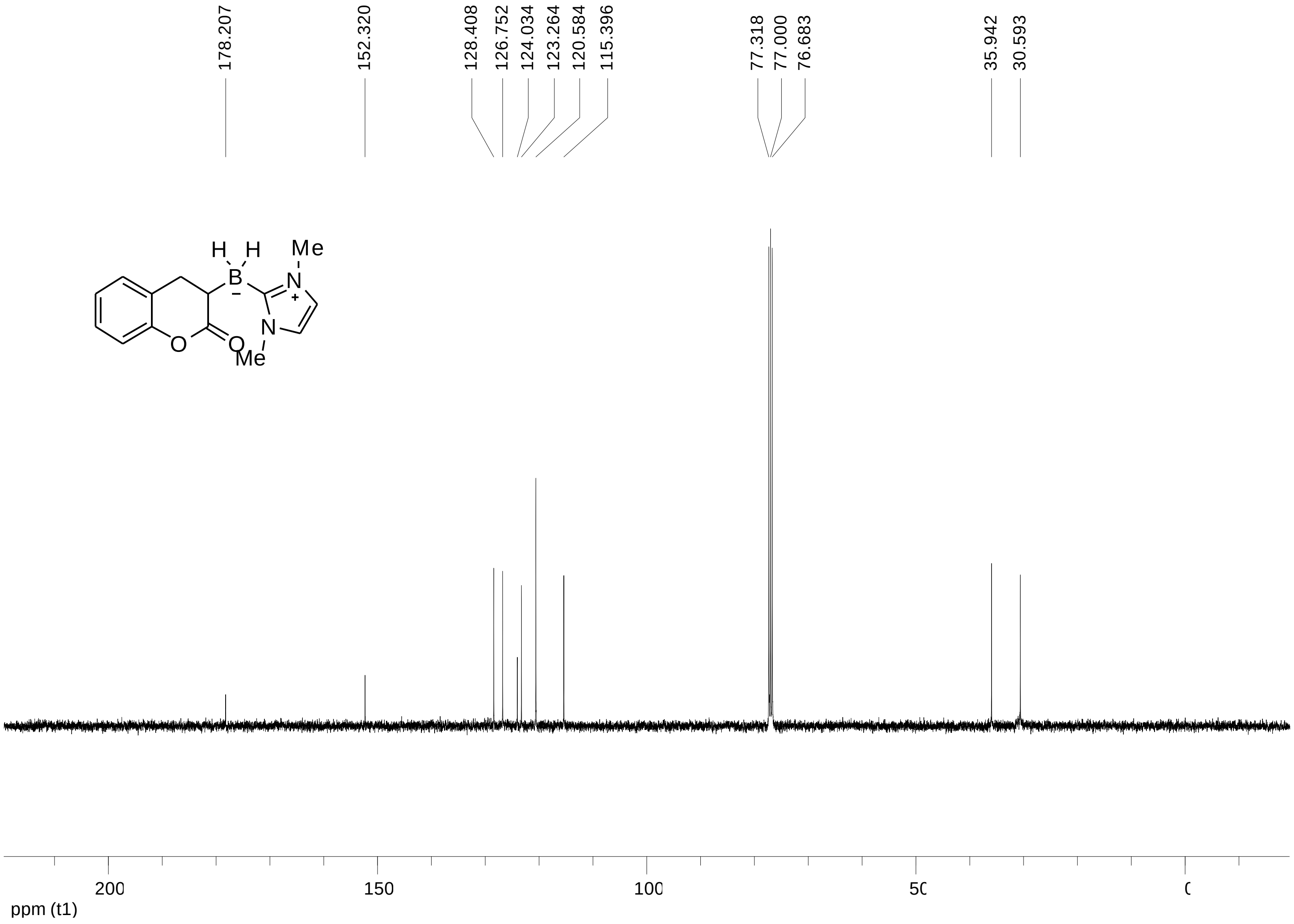

Supplementary Figure 91. ^{13}C NMR spectrum for 3n

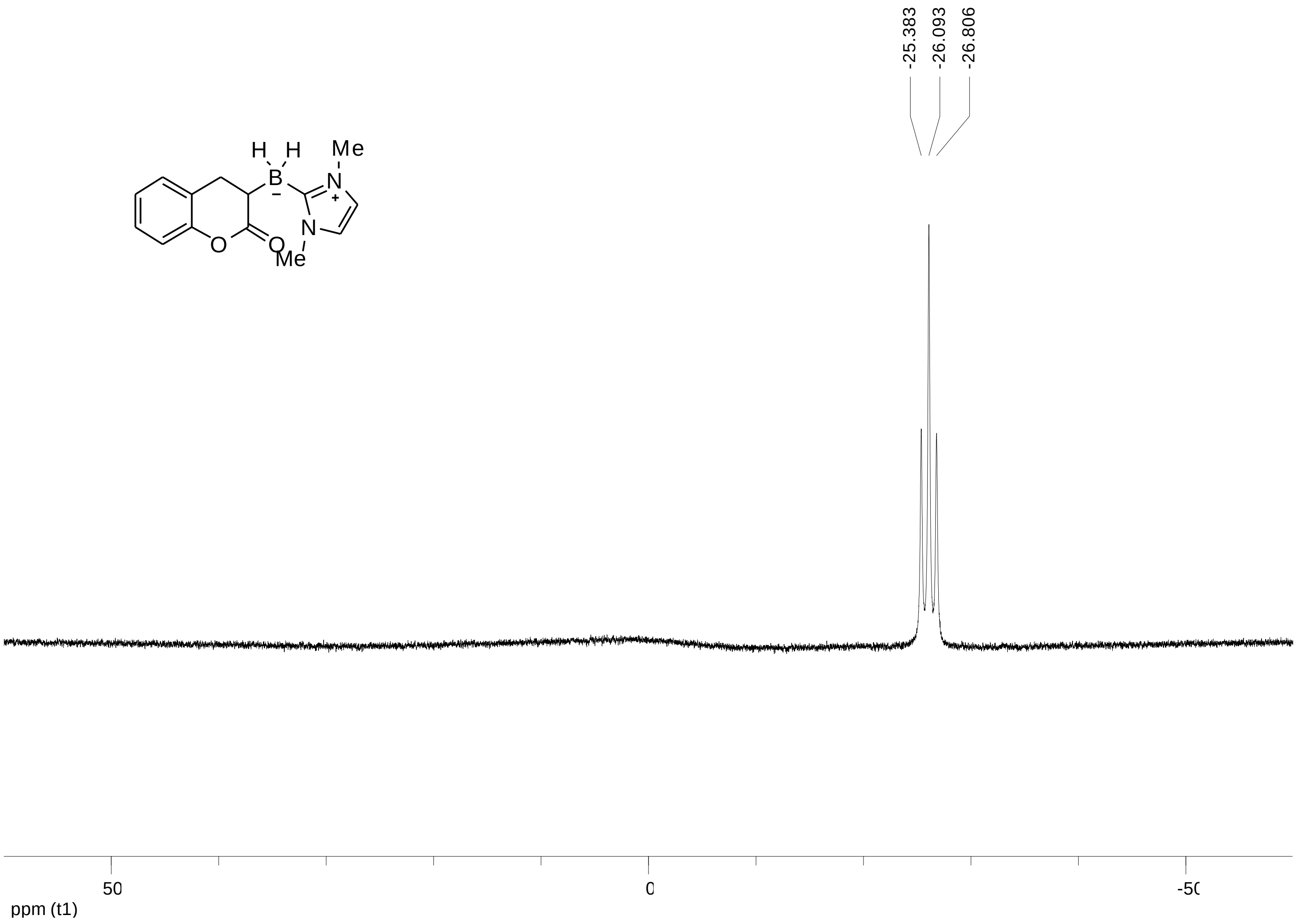

Supplementary Figure 92. ^{11}B NMR spectrum for 3n

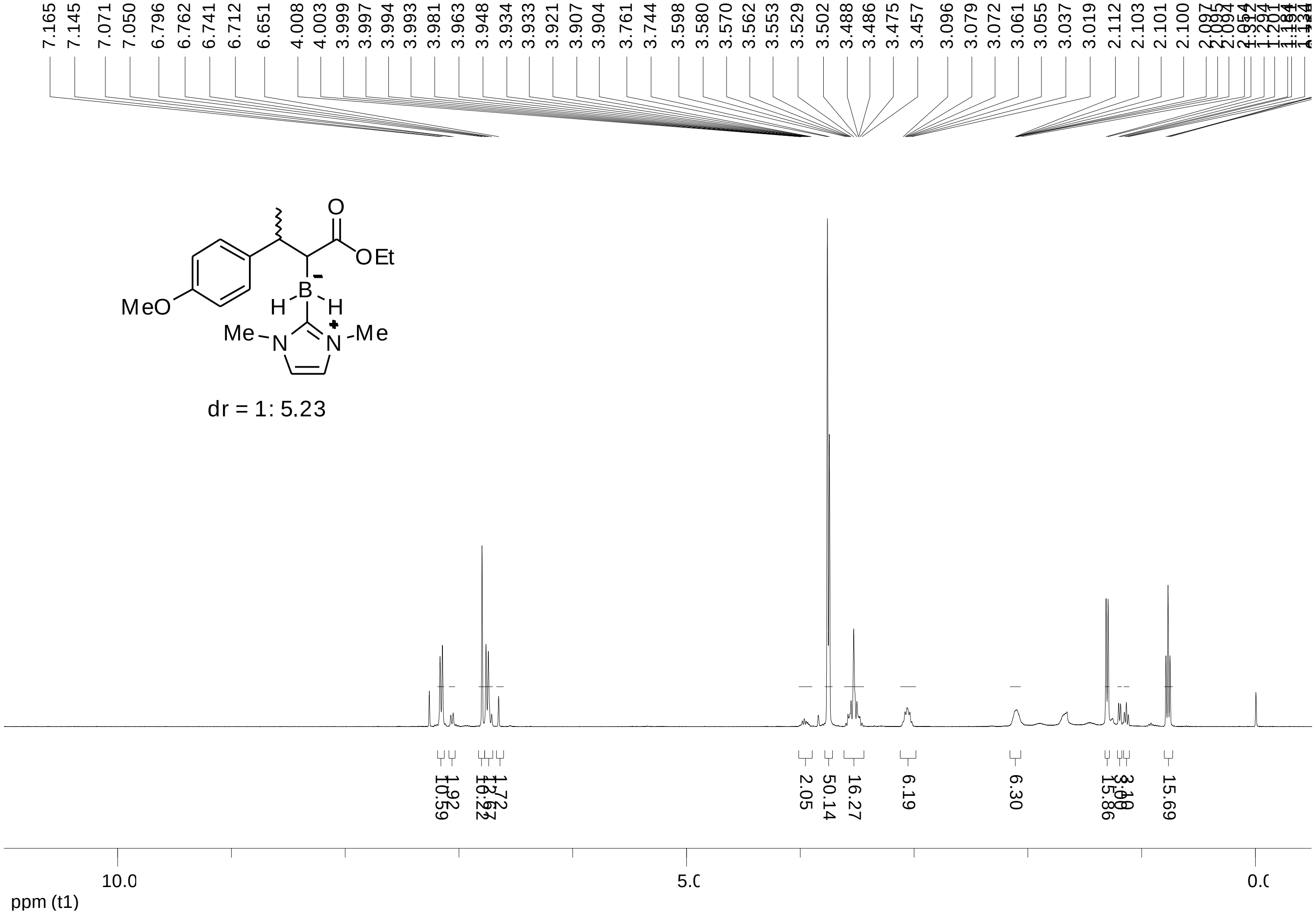

Supplementary Figure 93. ^{1}H NMR spectrum for 3o

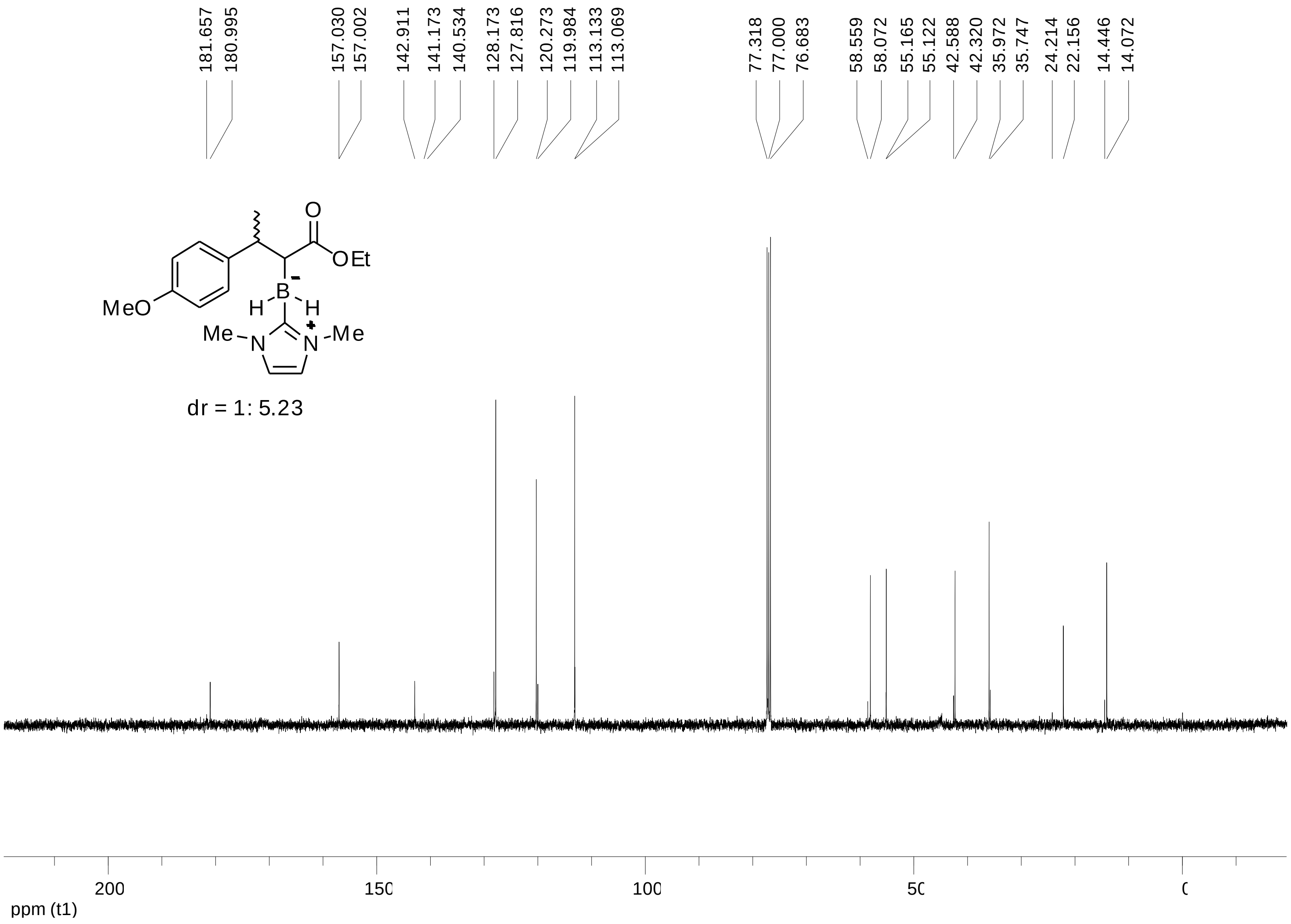

Supplementary Figure 94. ^{13}C NMR spectrum for 3o

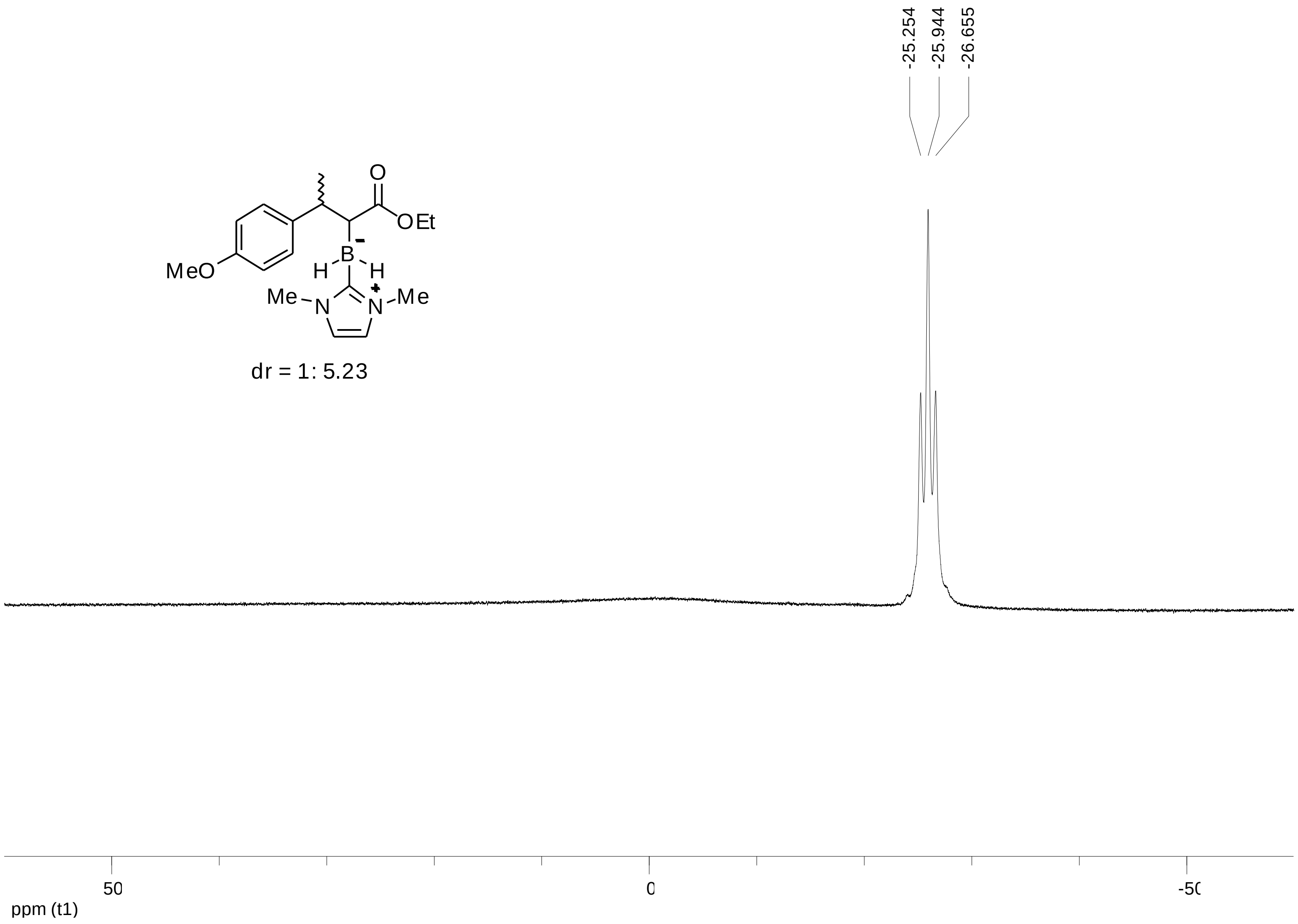

Supplementary Figure 95. ^{11}B NMR spectrum for 3o

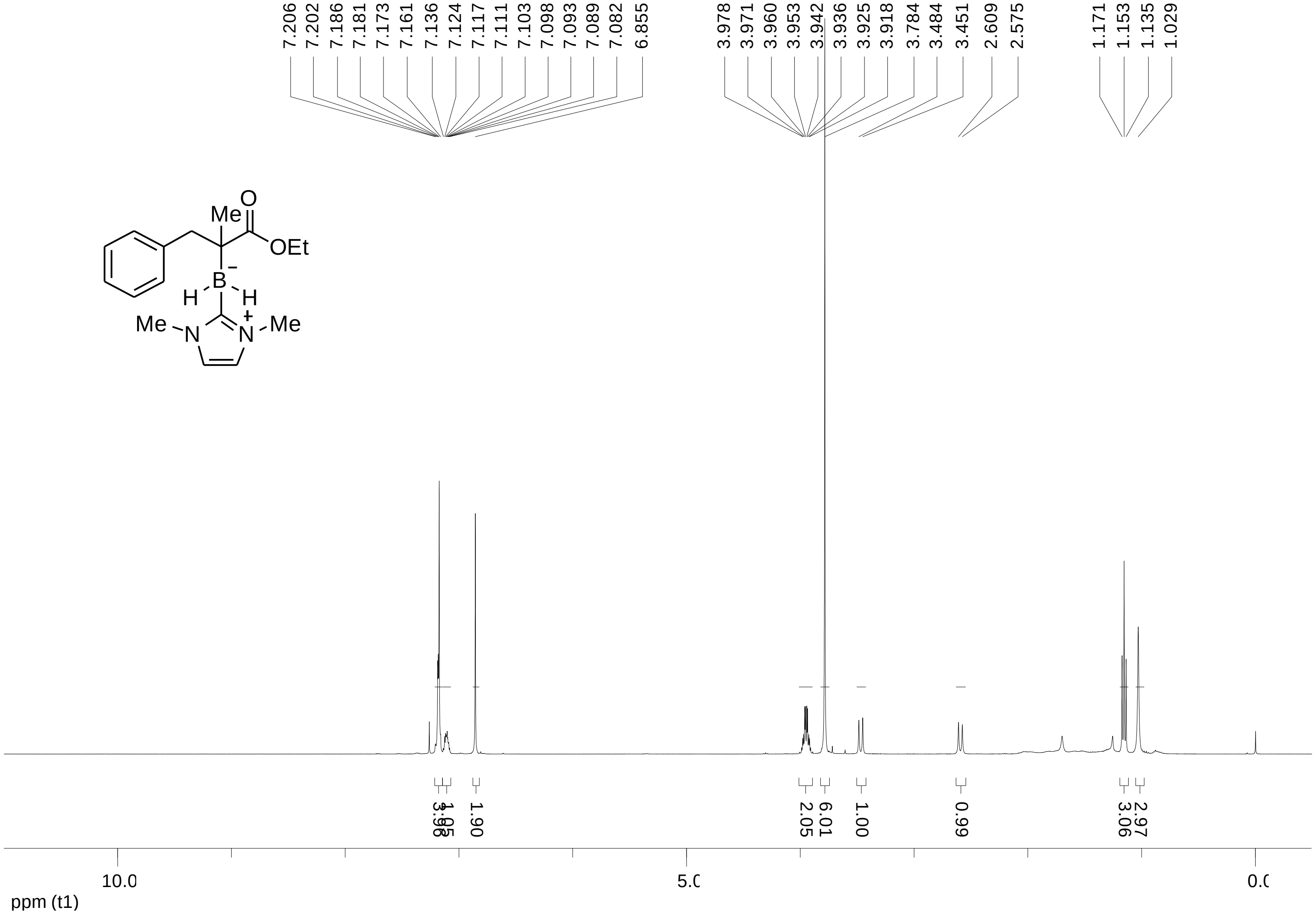

Supplementary Figure 96. ^{1}H NMR spectrum for 3p-α

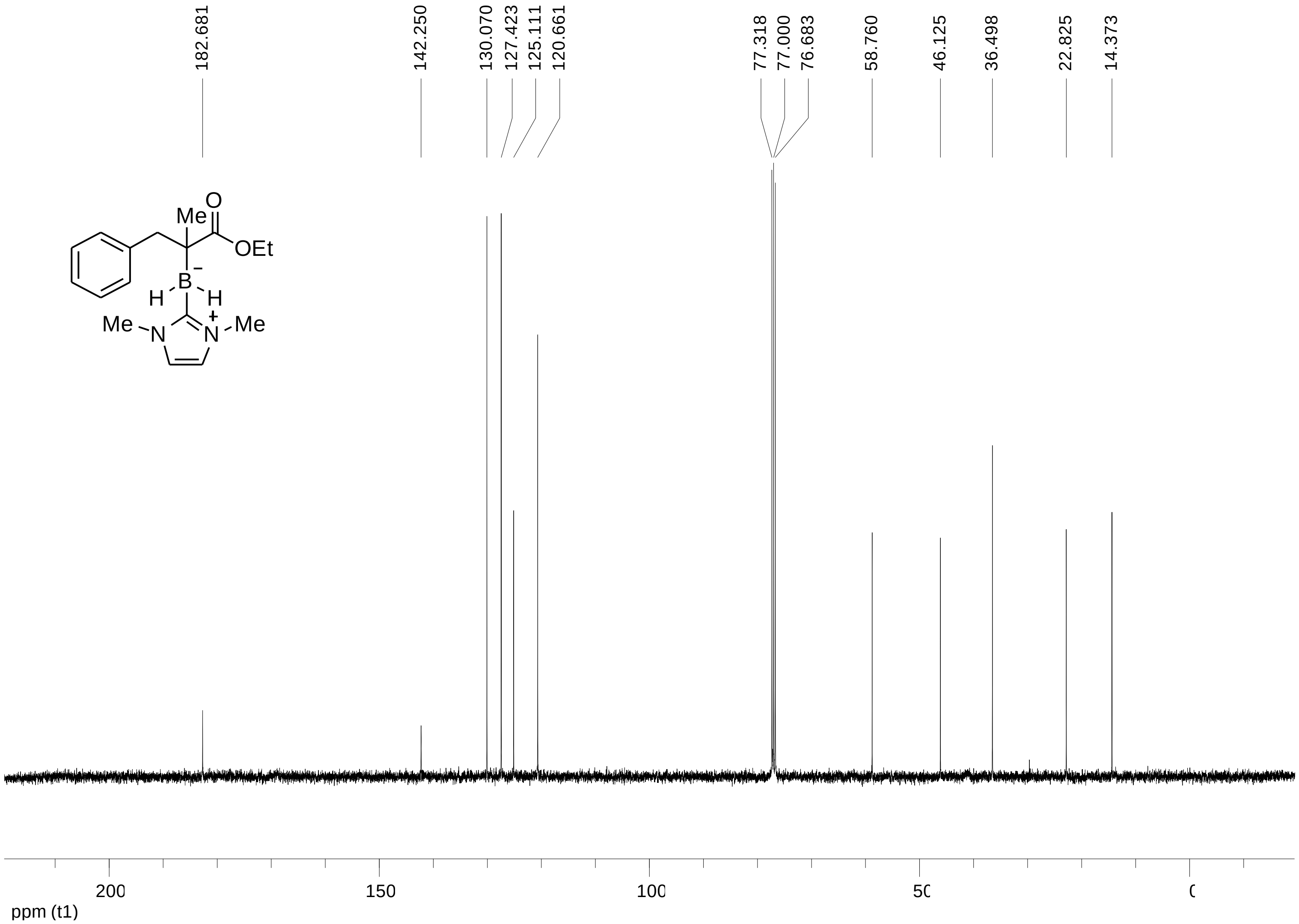

Supplementary Figure 97. ^{13}C NMR spectrum for 3p-α

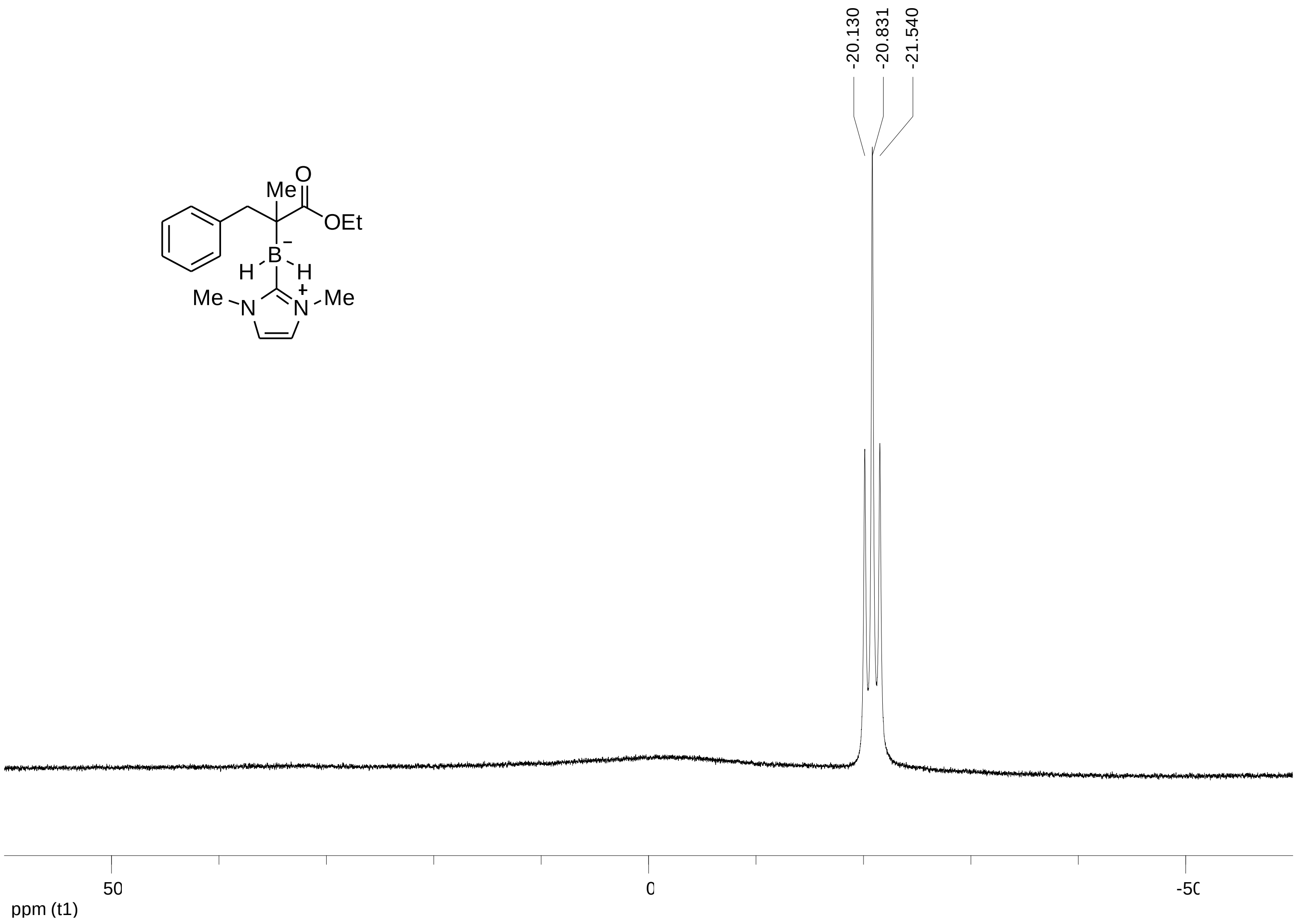

Supplementary Figure 98. ^{11}B NMR spectrum for 3p-α

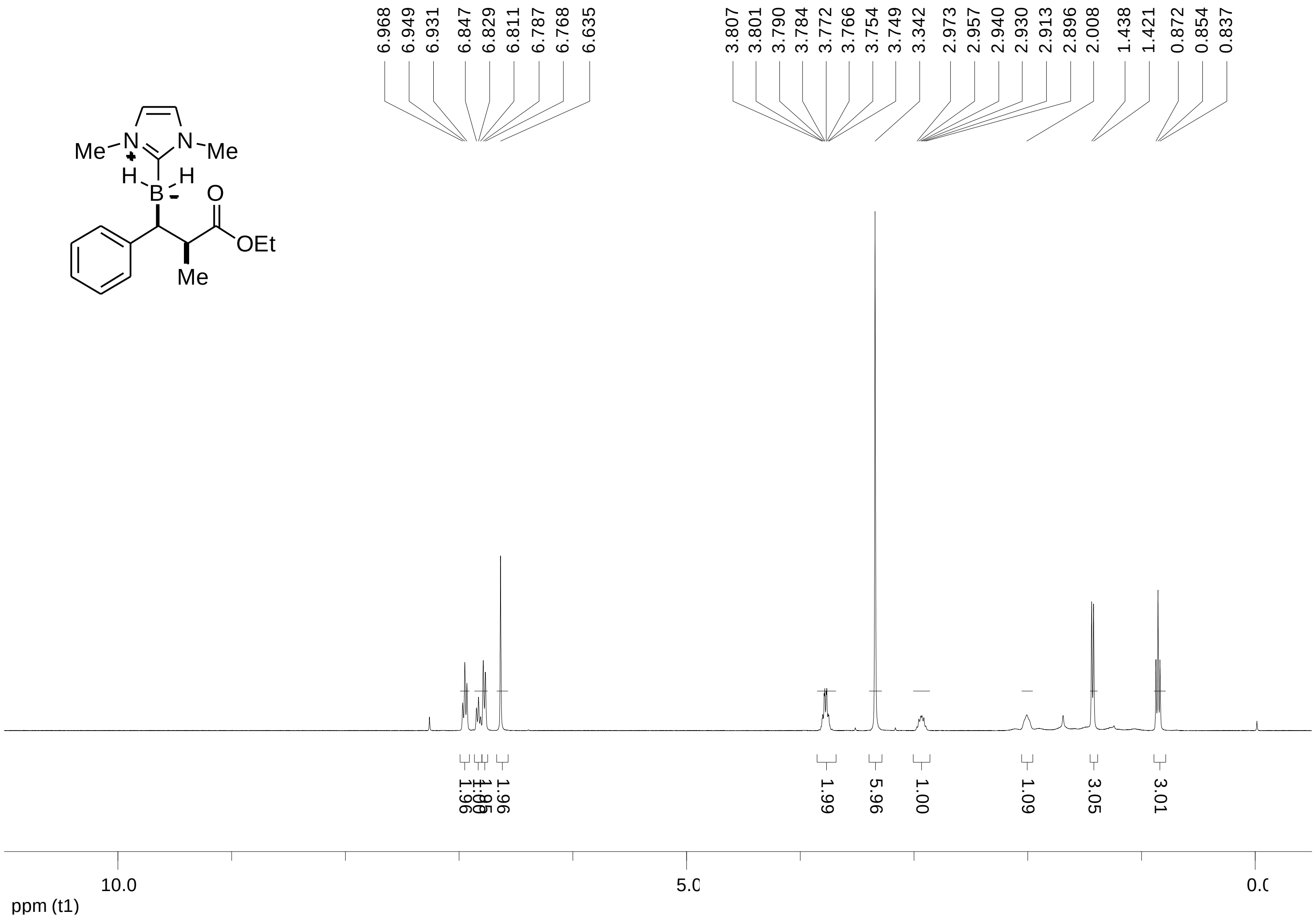

Supplementary Figure 99. ^{1}H NMR spectrum for 3p-β

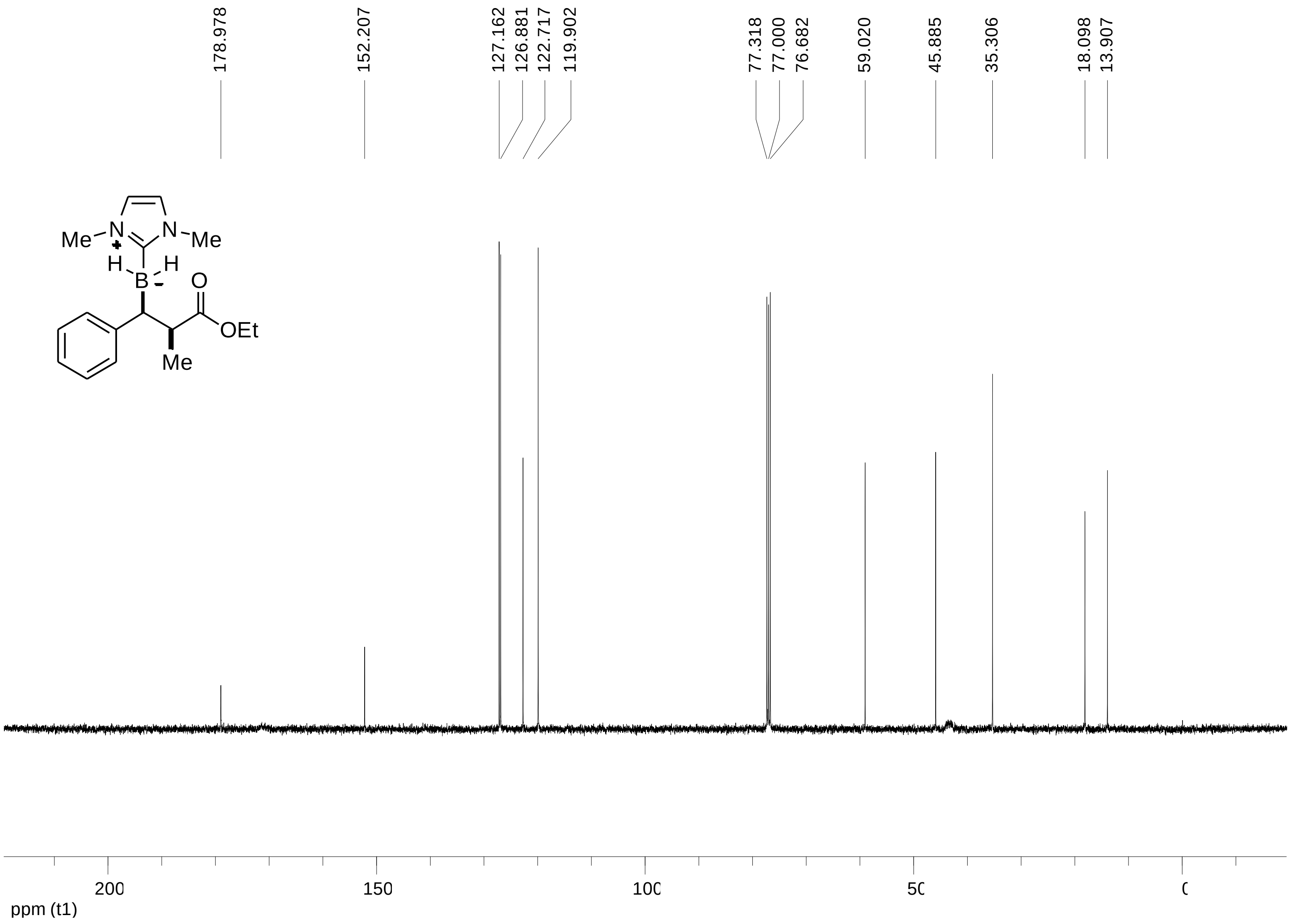

Supplementary Figure 100. ^{13}C NMR spectrum for 3p-β

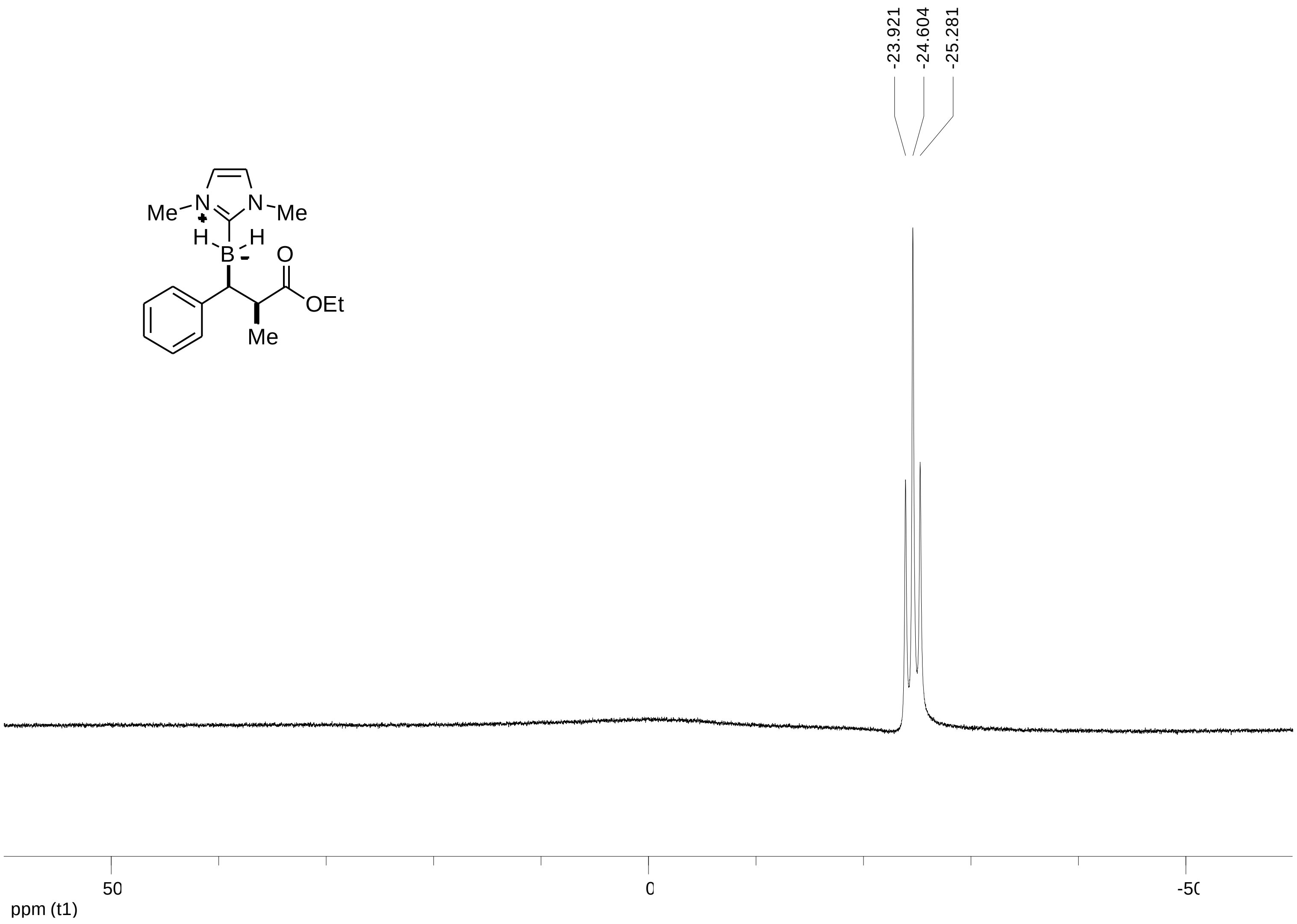

Supplementary Figure 101. ^{11}B NMR spectrum for 3p-β

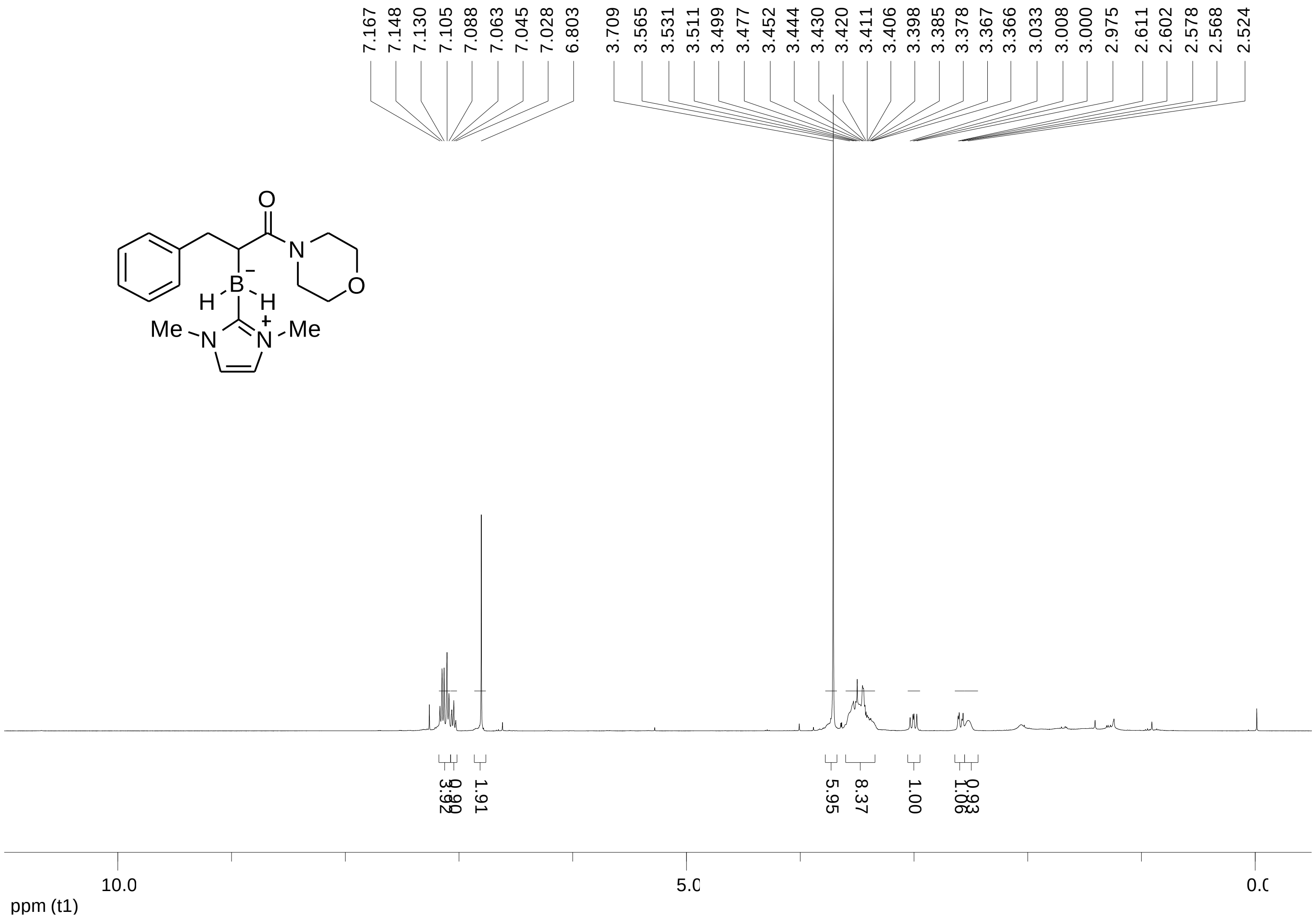

Supplementary Figure 102. ^{1}H NMR spectrum for 3q

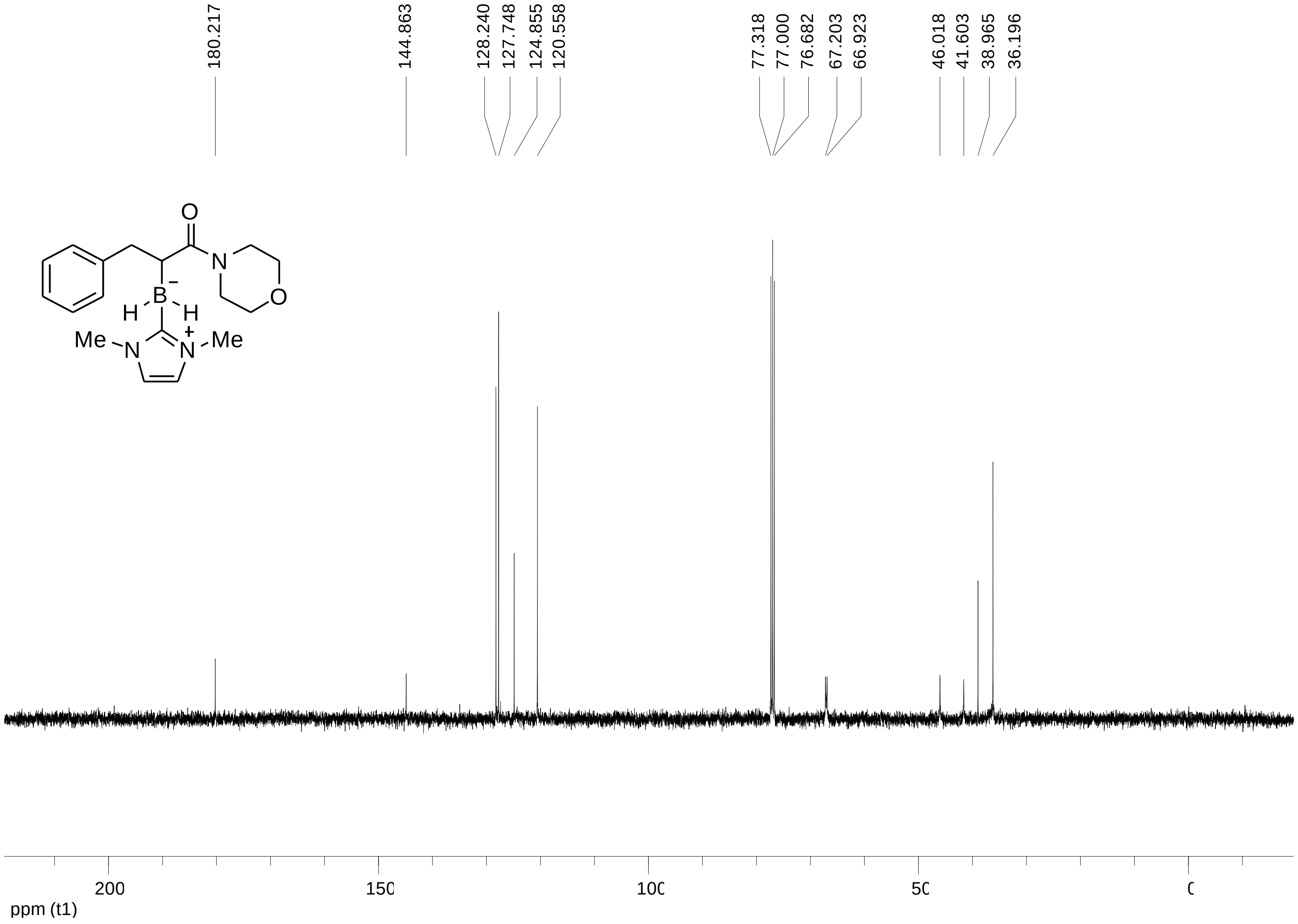

Supplementary Figure 103. ^{13}C NMR spectrum for 3q

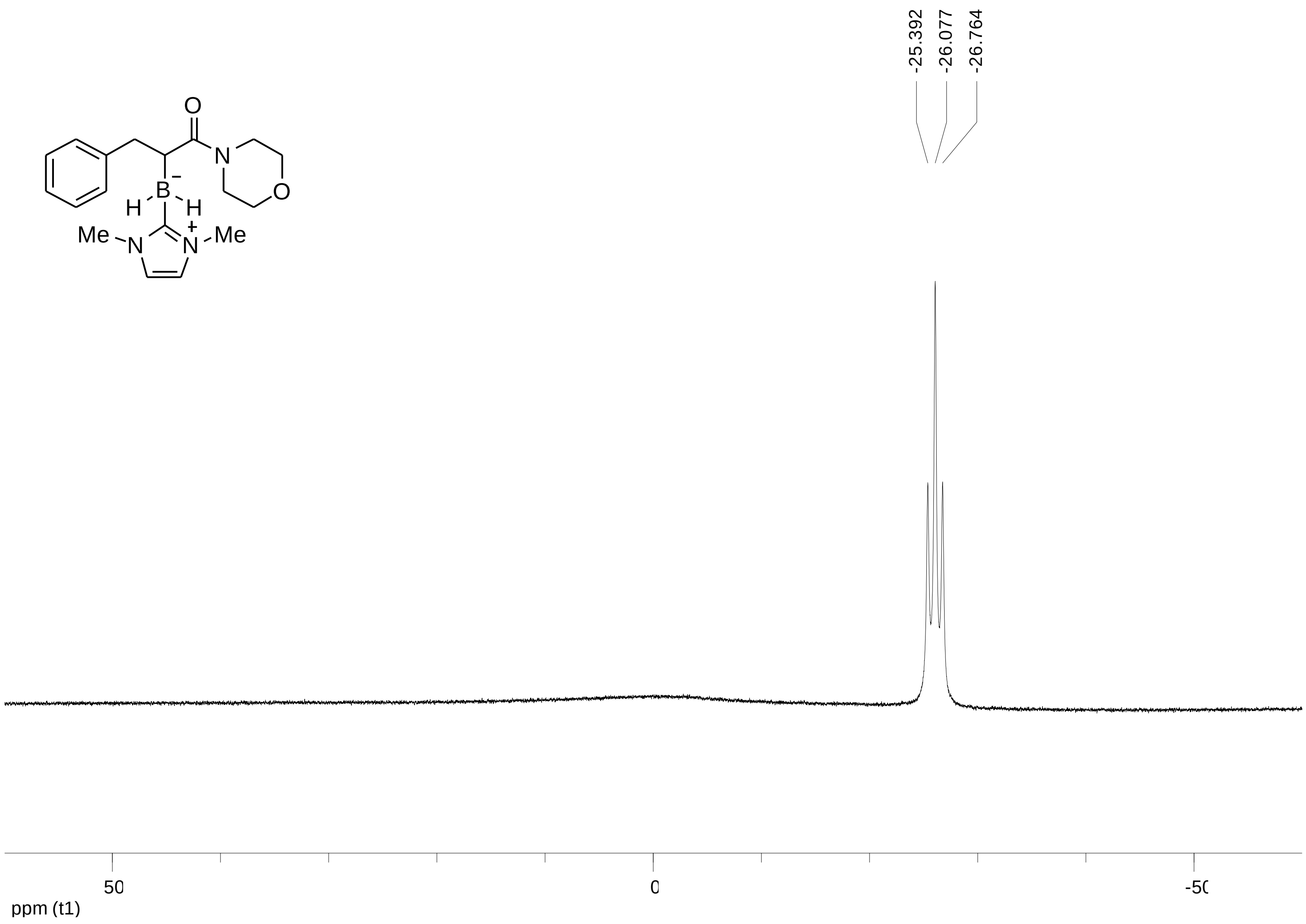

Supplementary Figure 104. ^{11}B NMR spectrum for 3q

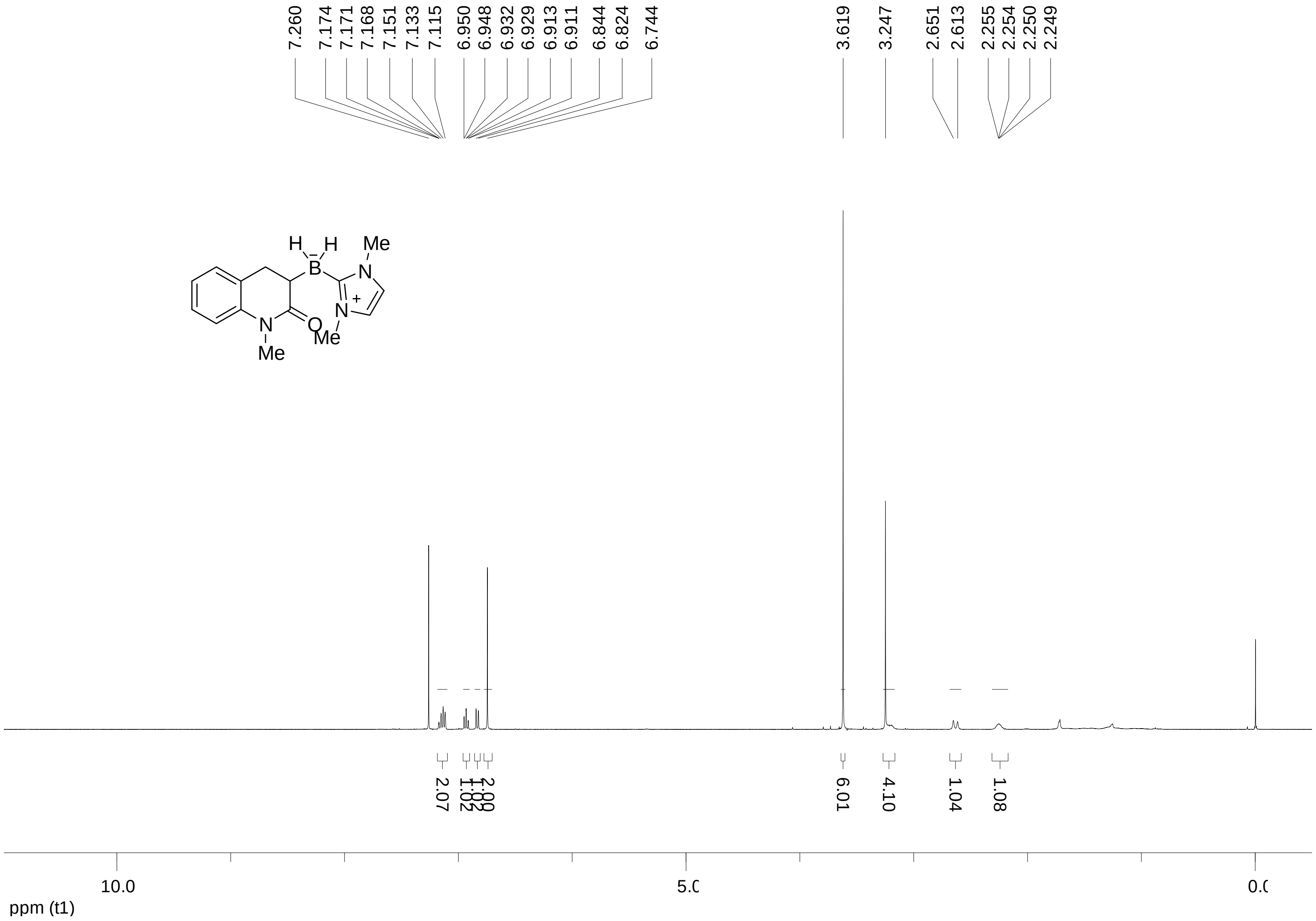

Supplementary Figure 105. ^{1}H NMR spectrum for 3r

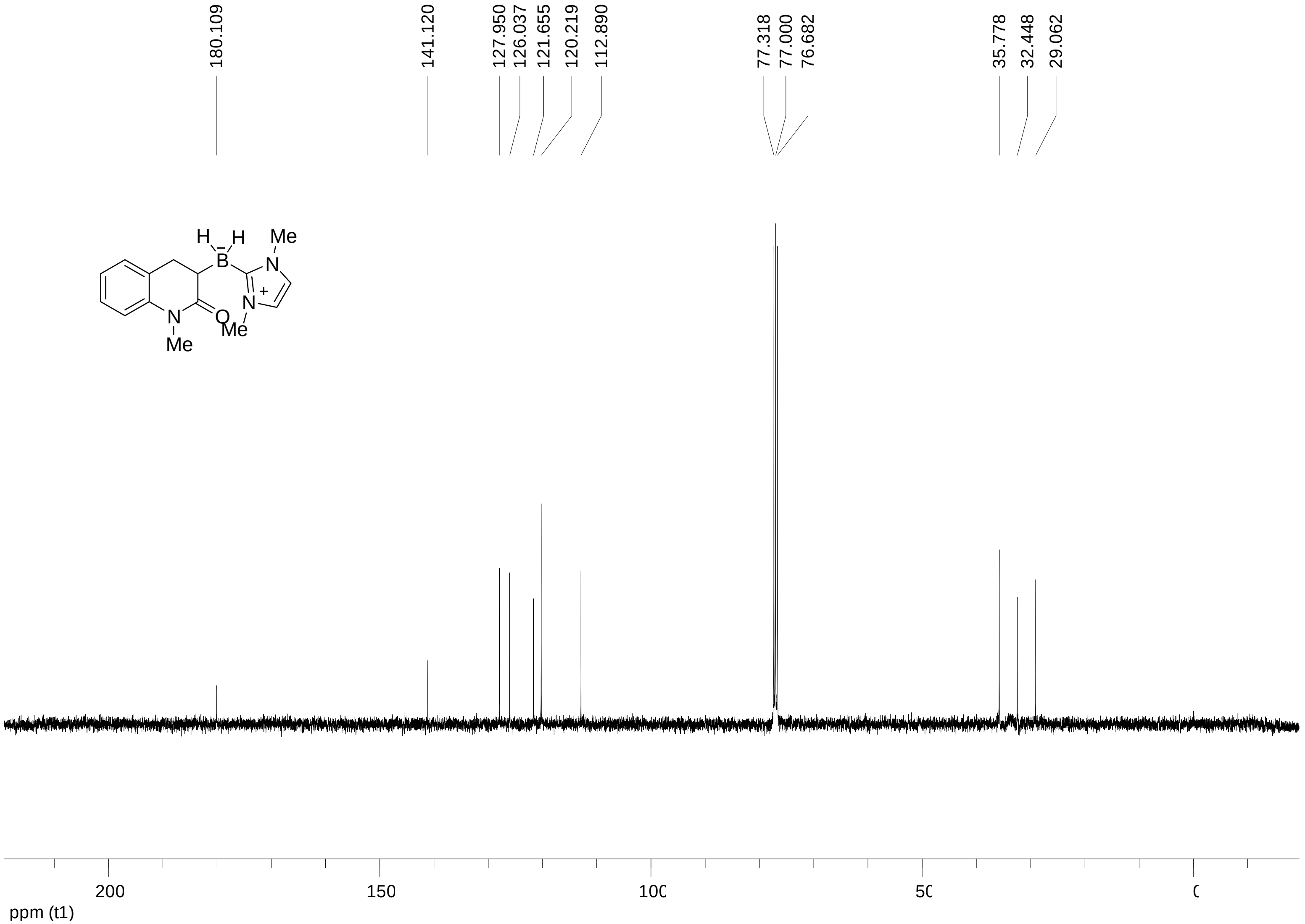

Supplementary Figure 106. ^{13}**C NMR spectrum for 3r**

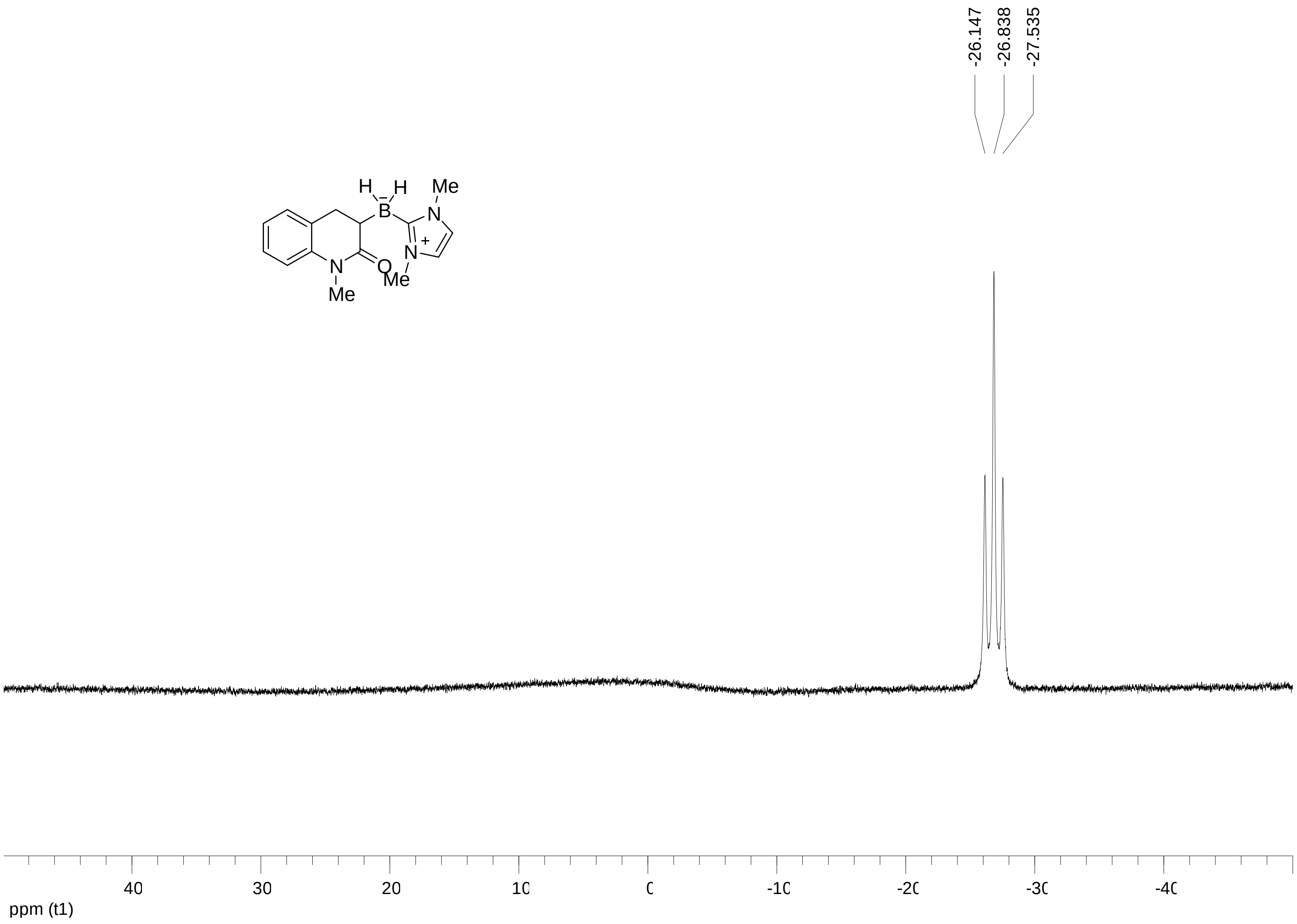

Supplementary Figure 107. ^{11}B **NMR spectrum for 3r**

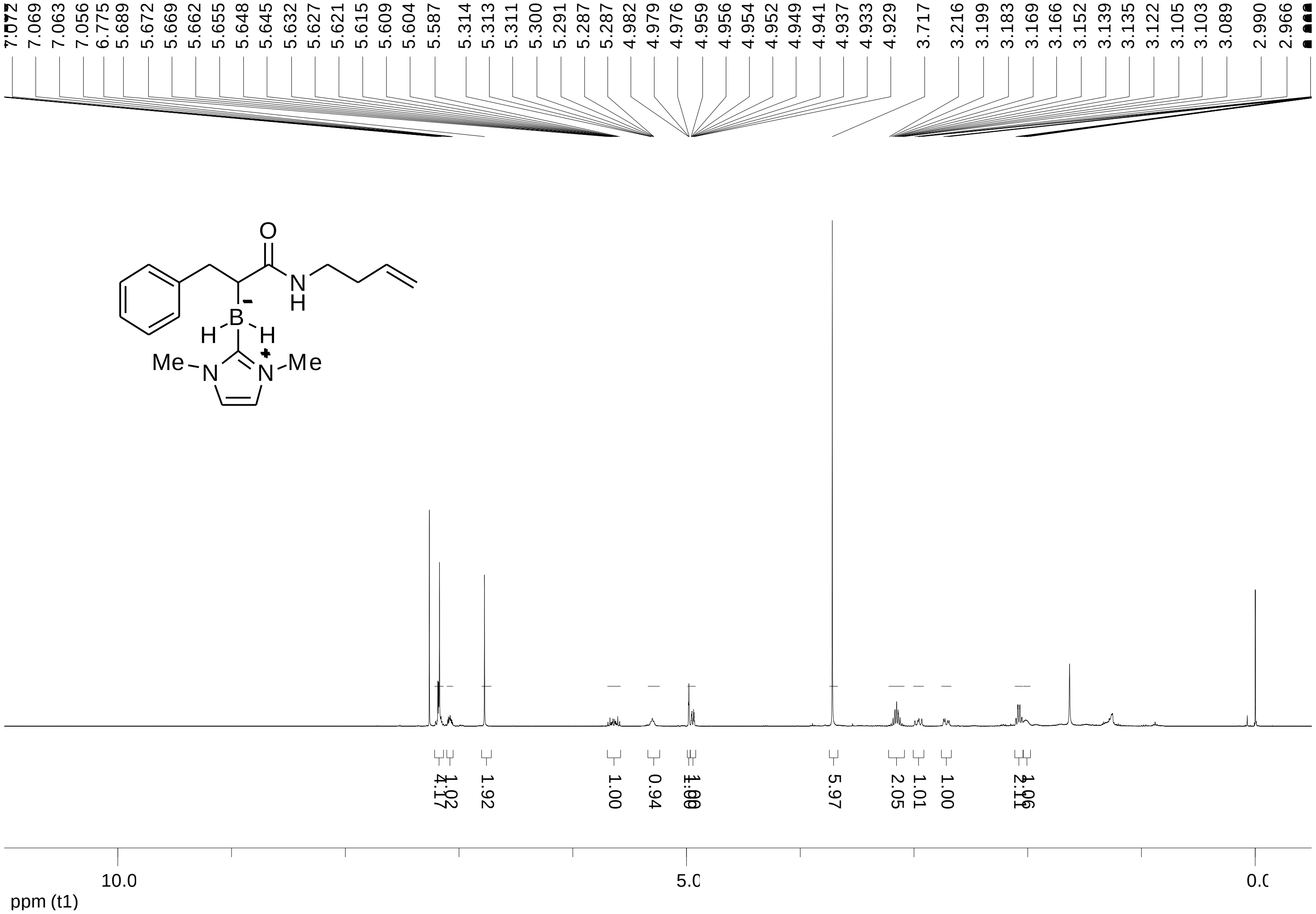

Supplementary Figure 108. ^{1}H NMR spectrum for 3s

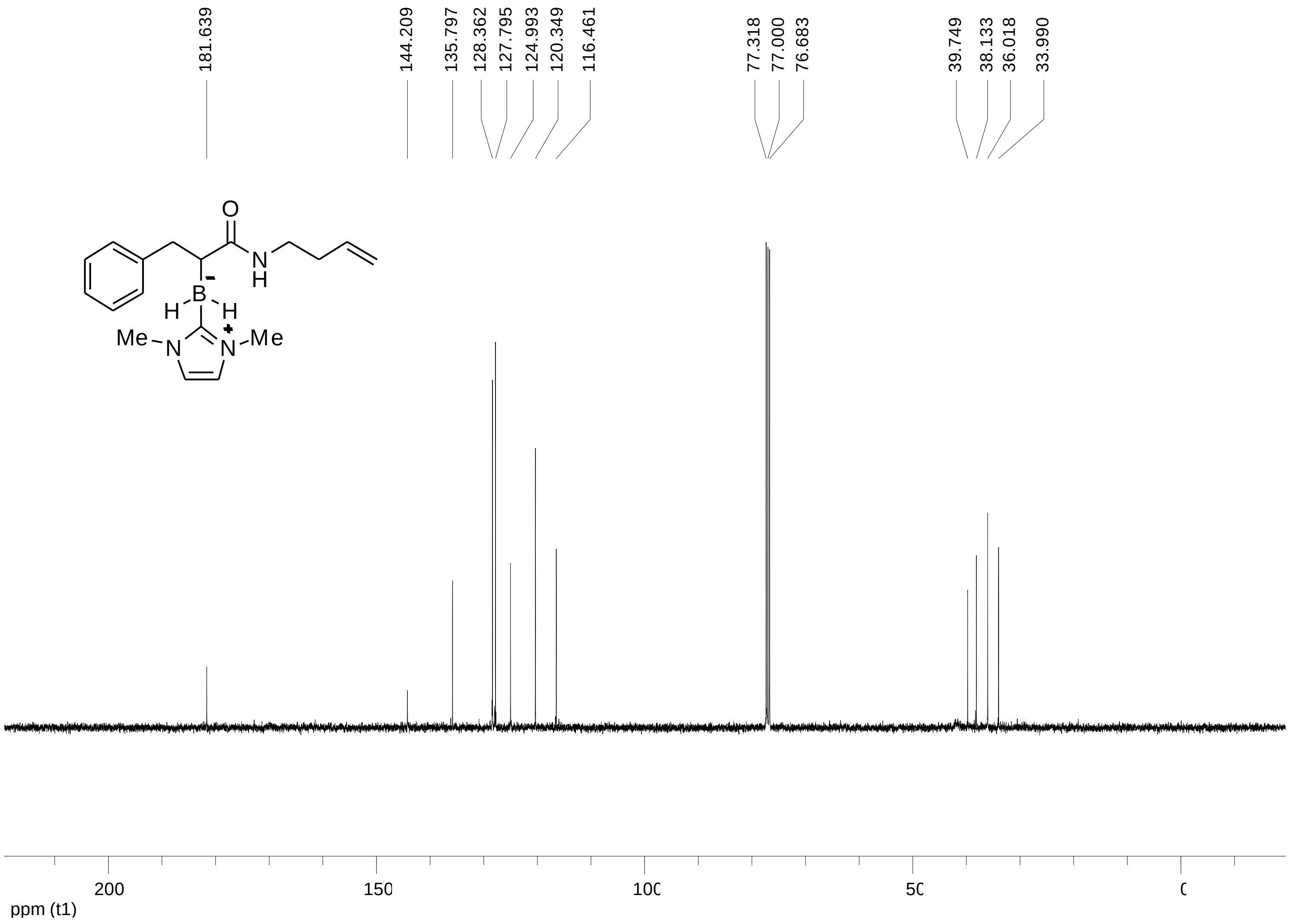

Supplementary Figure 109. ^{13}C NMR spectrum for 3s

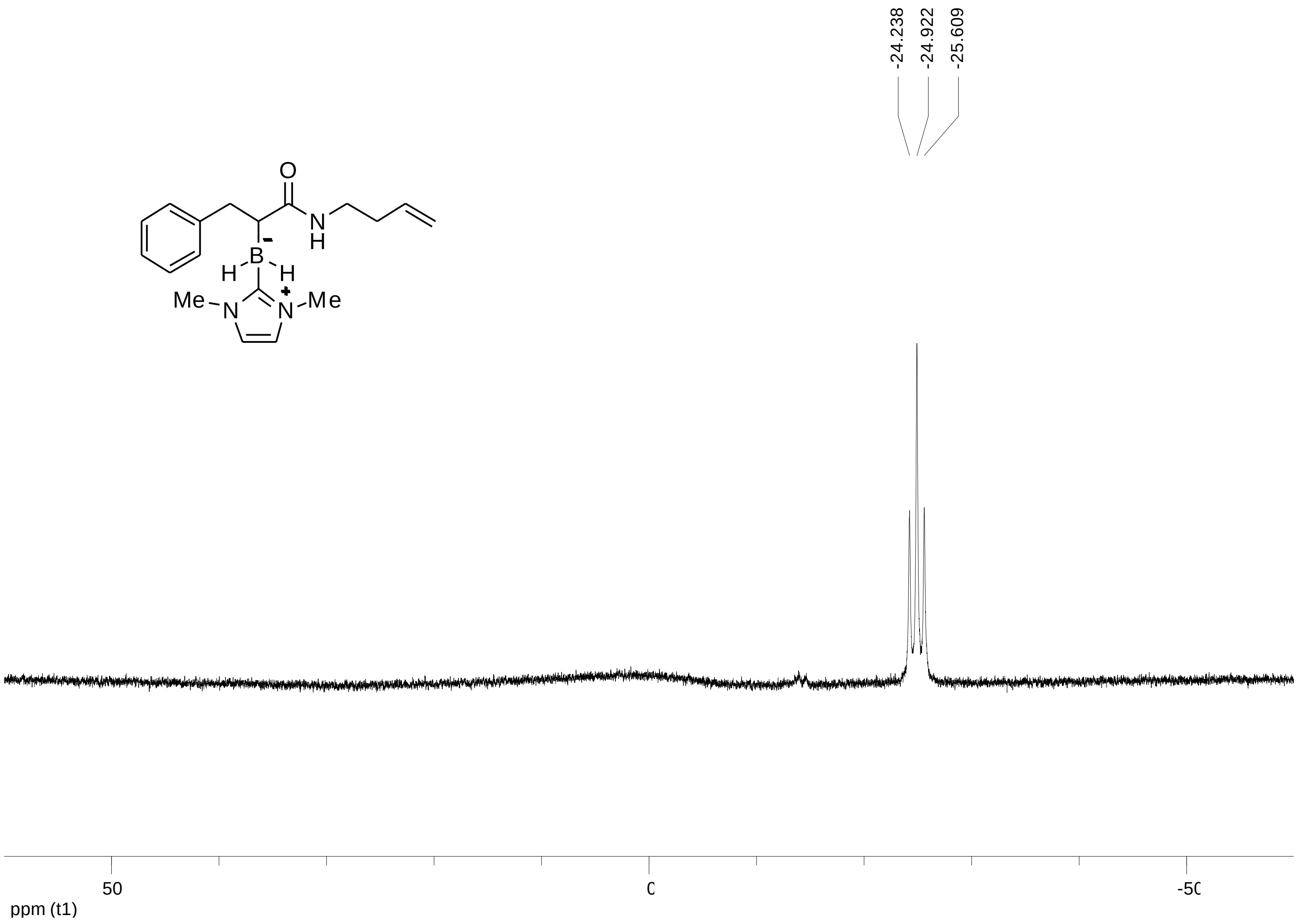

Supplementary Figure 110. ^{11}B NMR spectrum for 3s

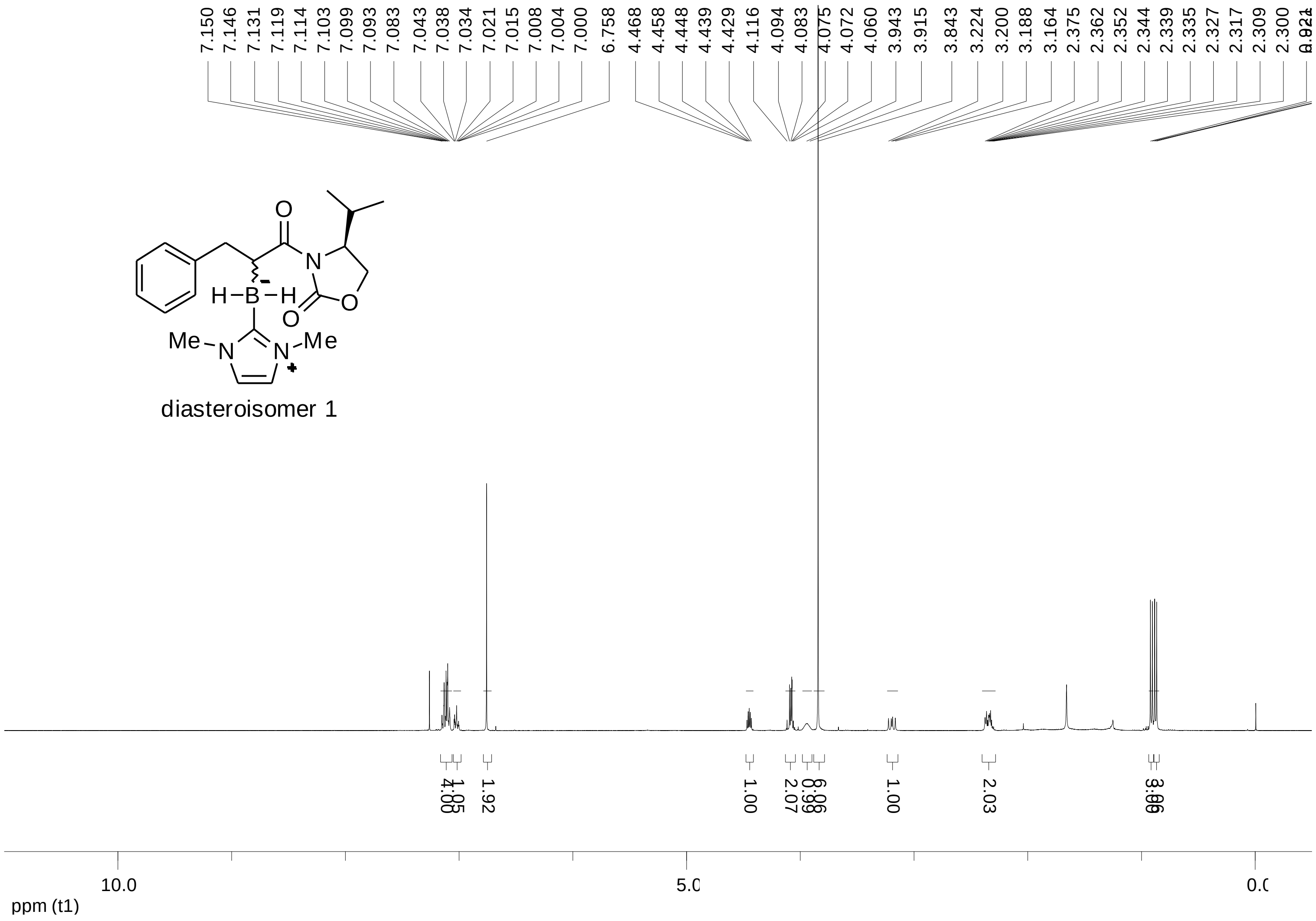

Supplementary Figure 111. ^{1}H NMR spectrum for 3t, diasteroisomer 1

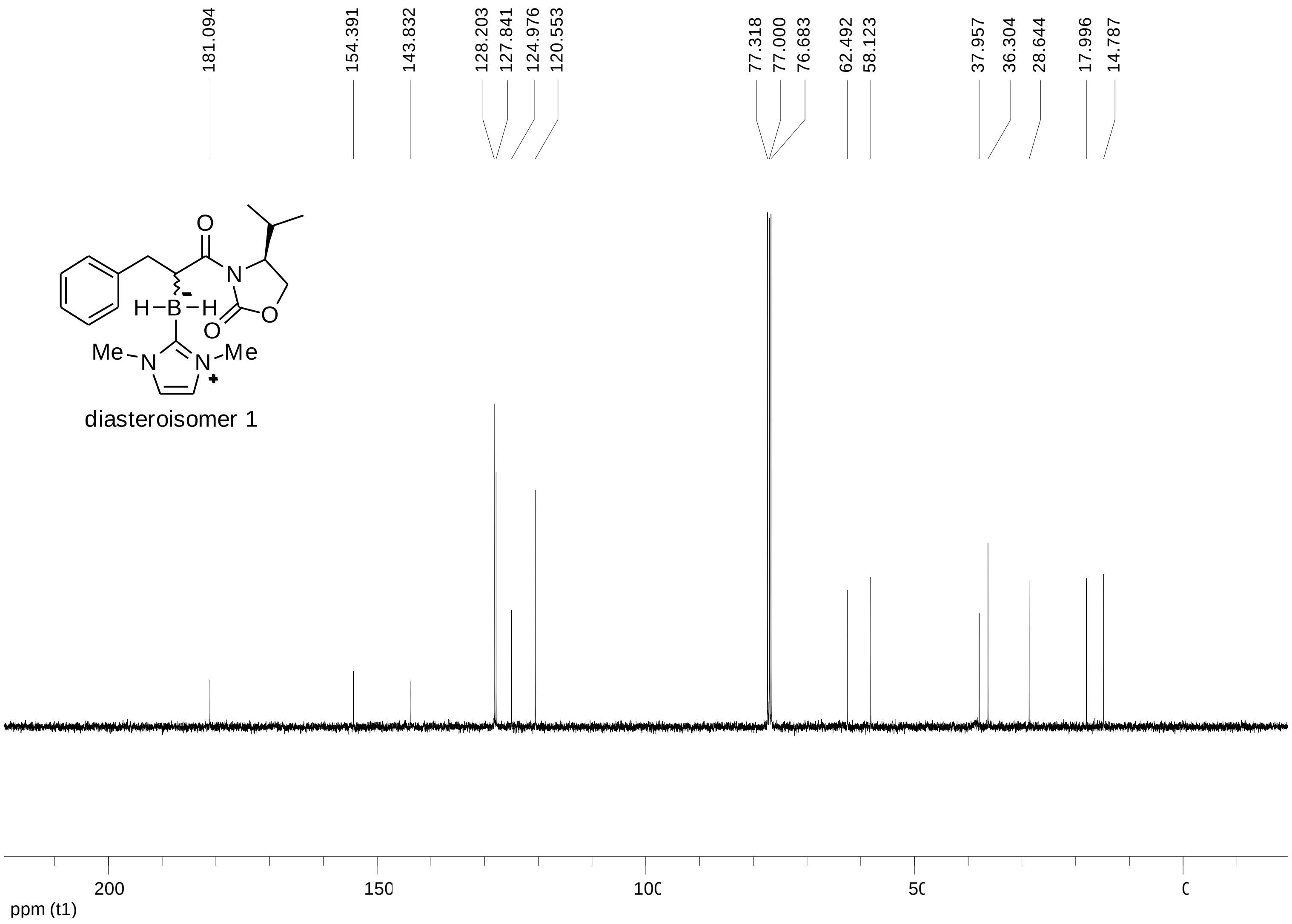

Supplementary Figure 112. ^{13}C NMR spectrum for 3t, diasteroisomer 1

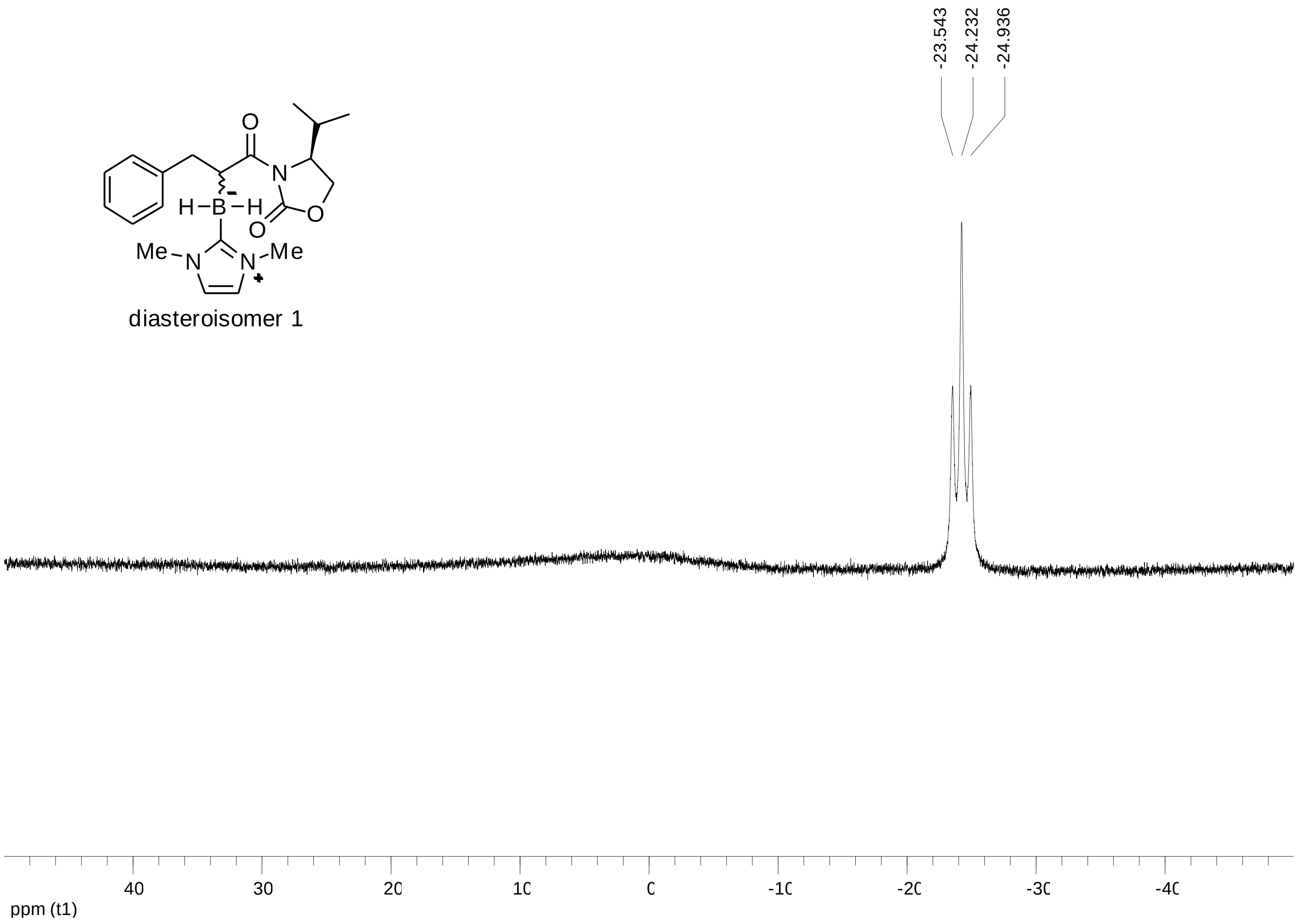

Supplementary Figure 113. ^{11}B NMR spectrum for 3t, diasteroisomer 1

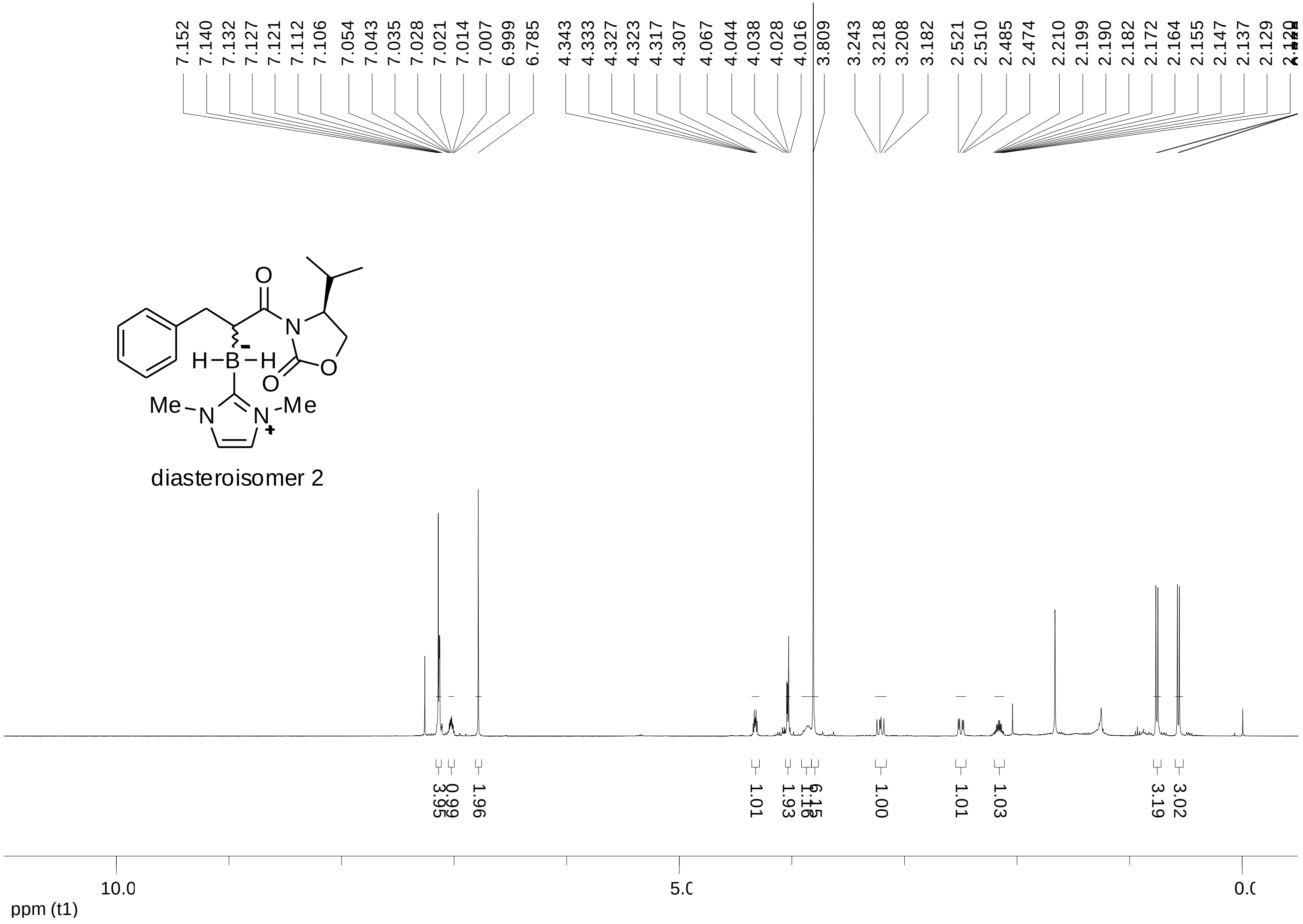

Supplementary Figure 114. ^{1}H NMR spectrum for 3t, diasteroisomer 2

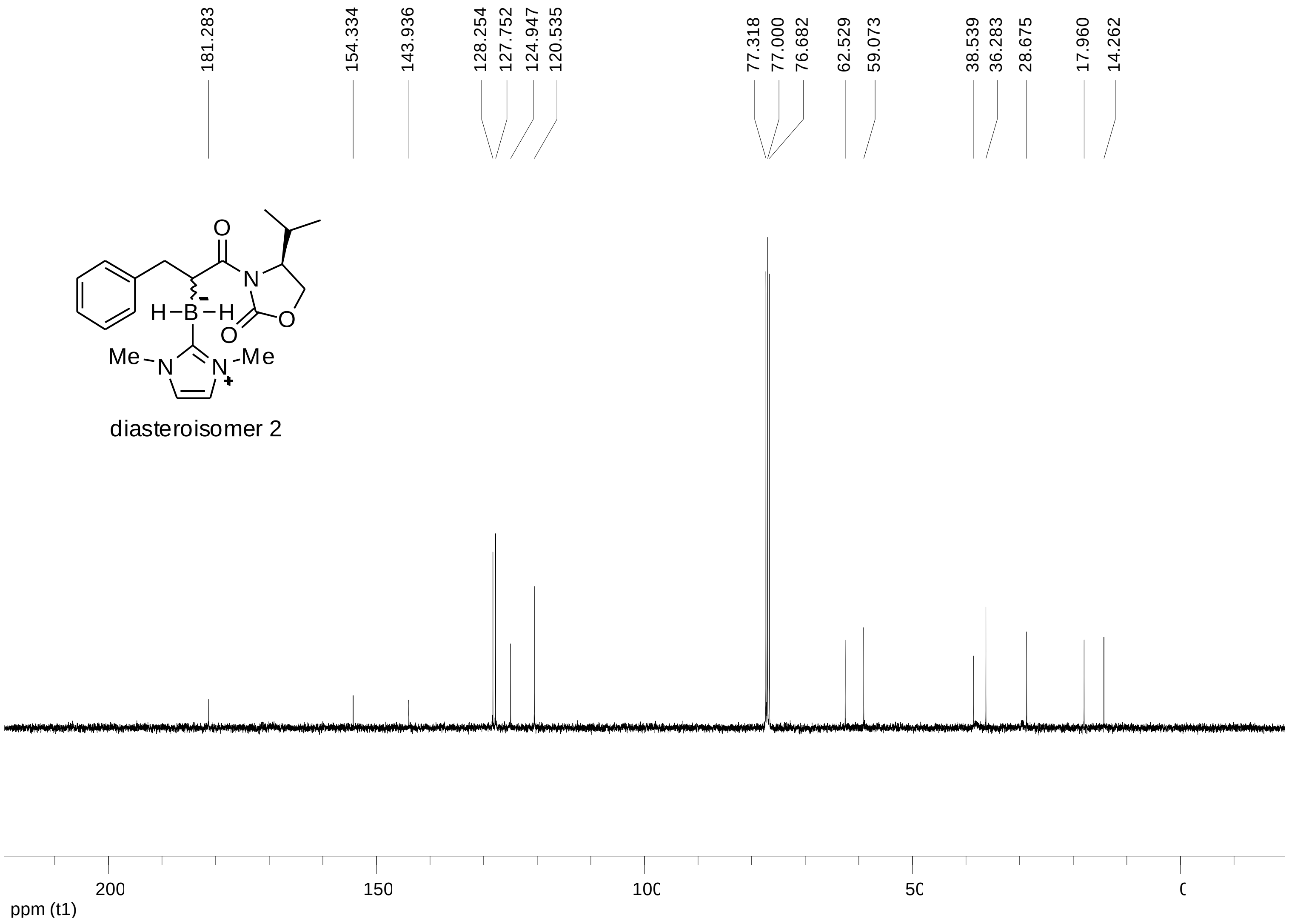

Supplementary Figure 115. ^{13}C NMR spectrum for 3t, diasteriisomer 2

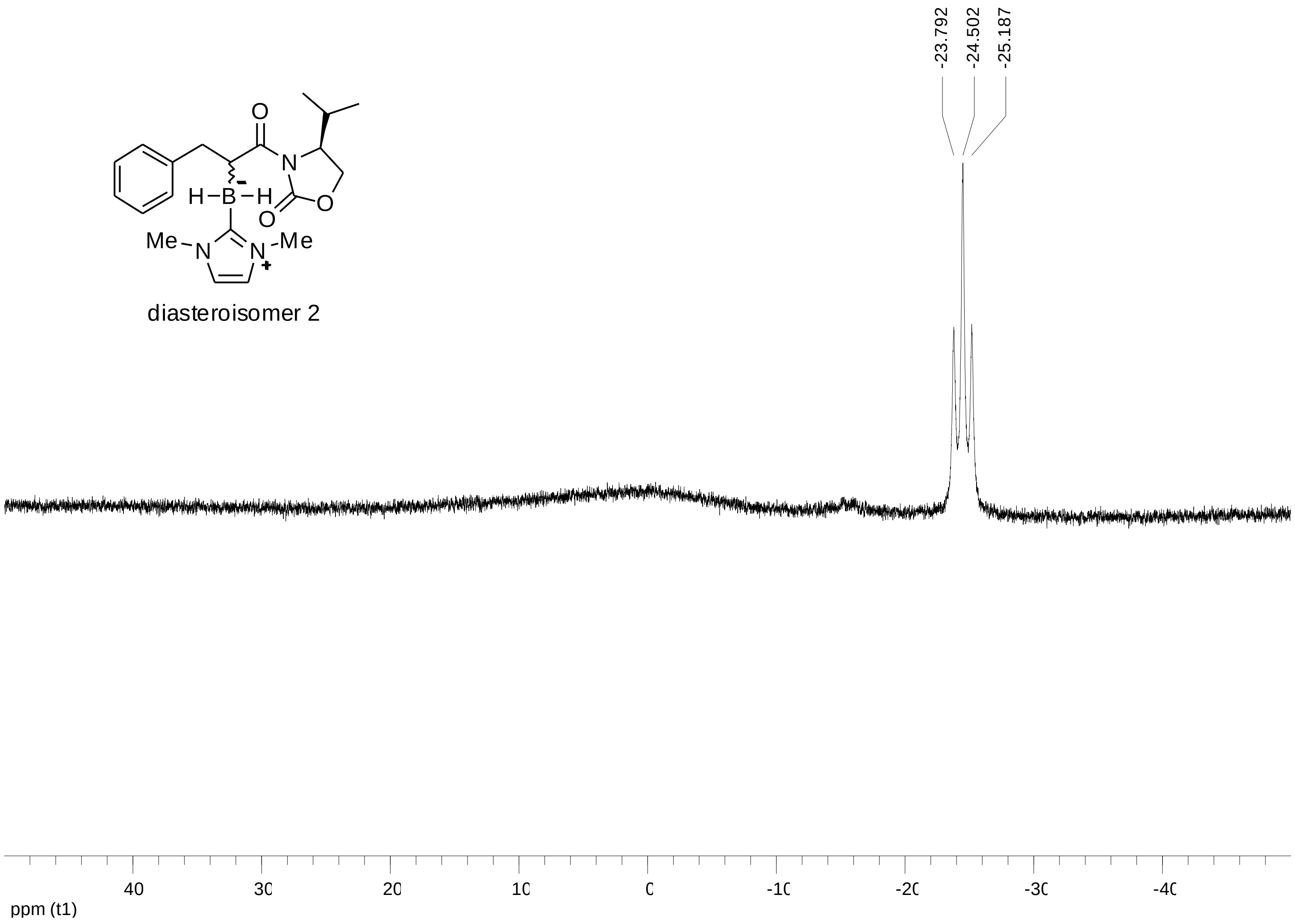

Supplementary Figure 116. ^{11}B NMR spectrum for 3t, diasteroisomer 2

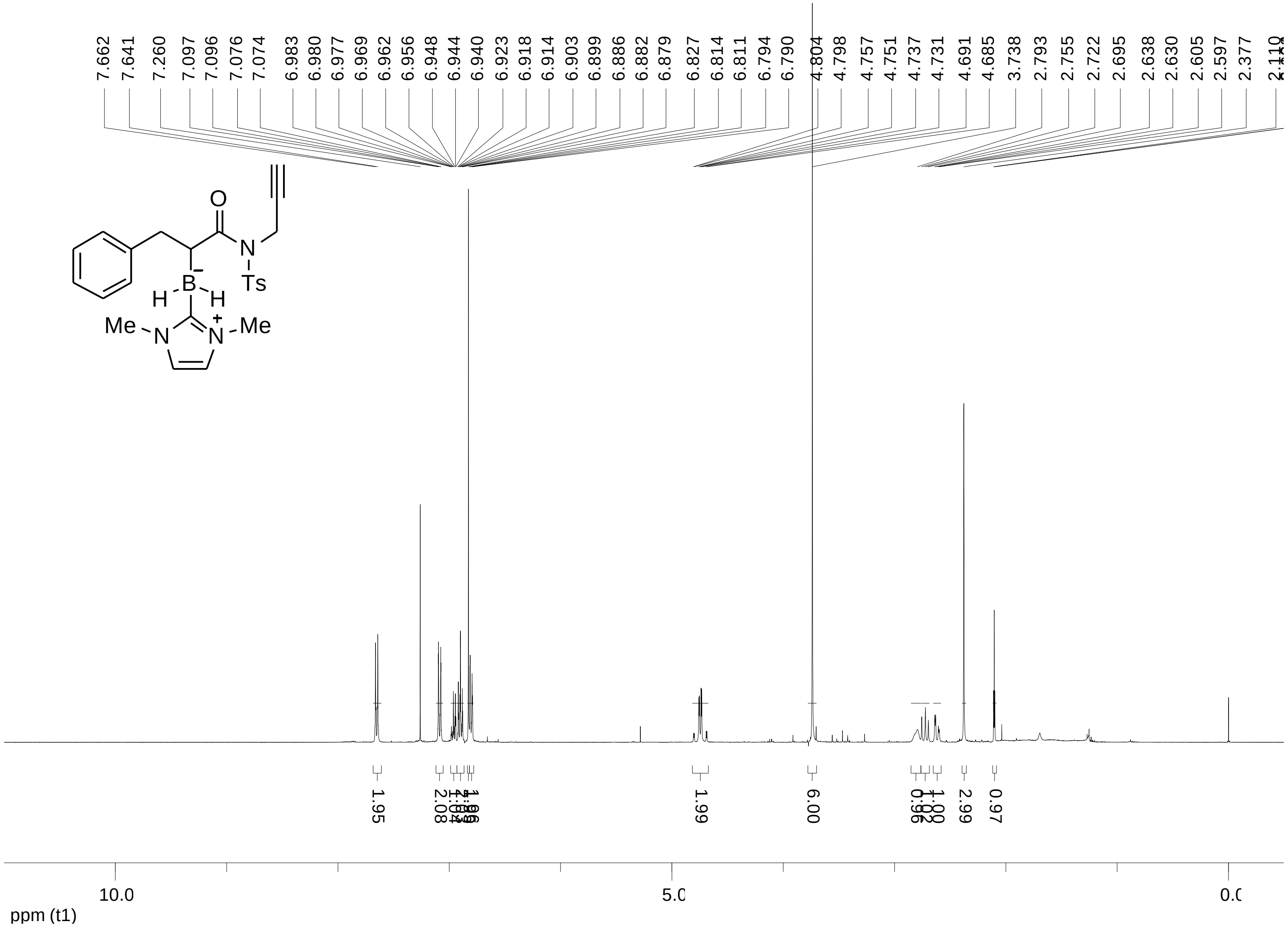

Supplementary Figure 117. ^{1}H NMR spectrum for 3u

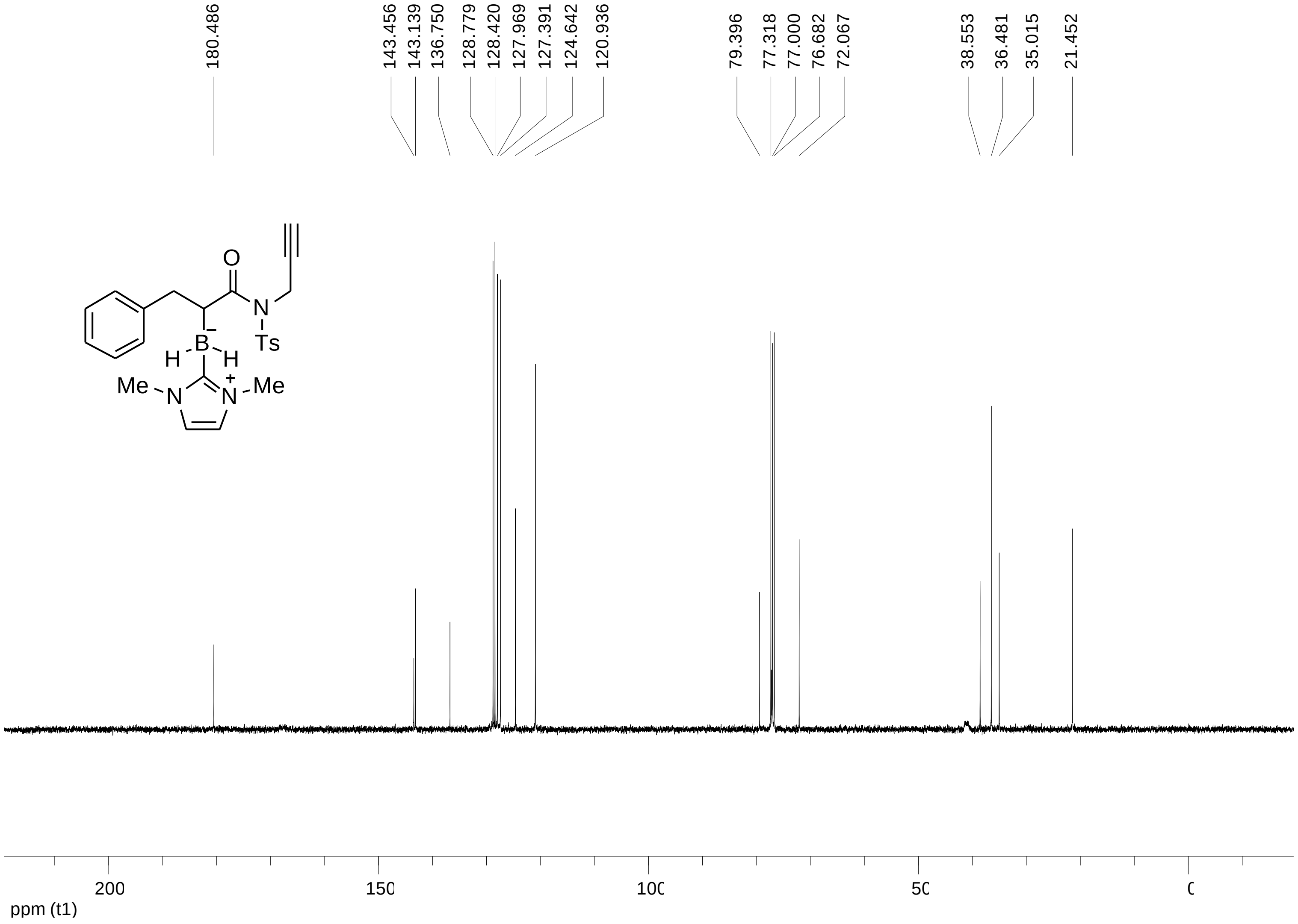

Supplementary Figure 118. ^{13}**C NMR spectrum for 3u**

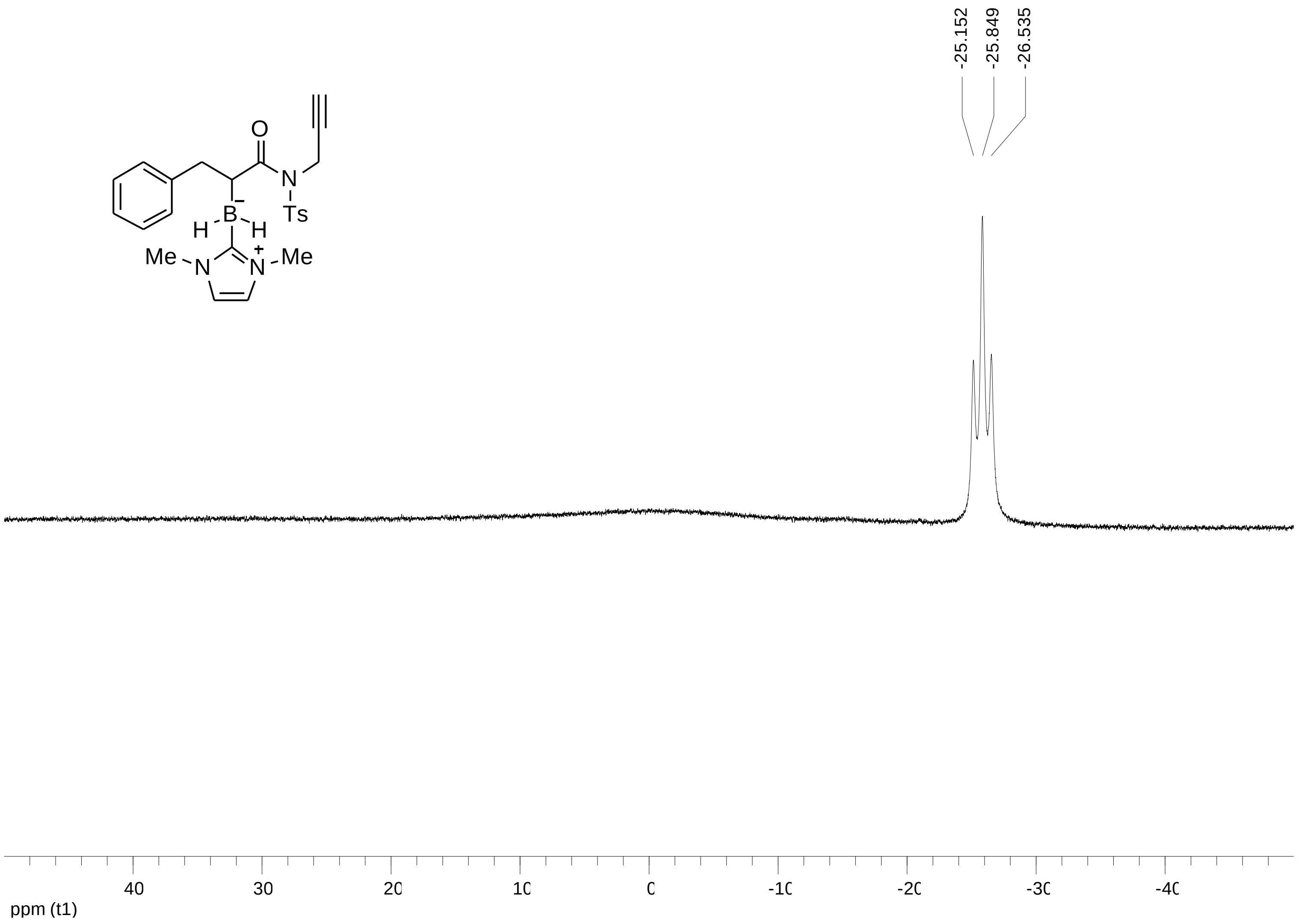

Supplementary Figure 119. ^{11}B NMR spectrum for 3u

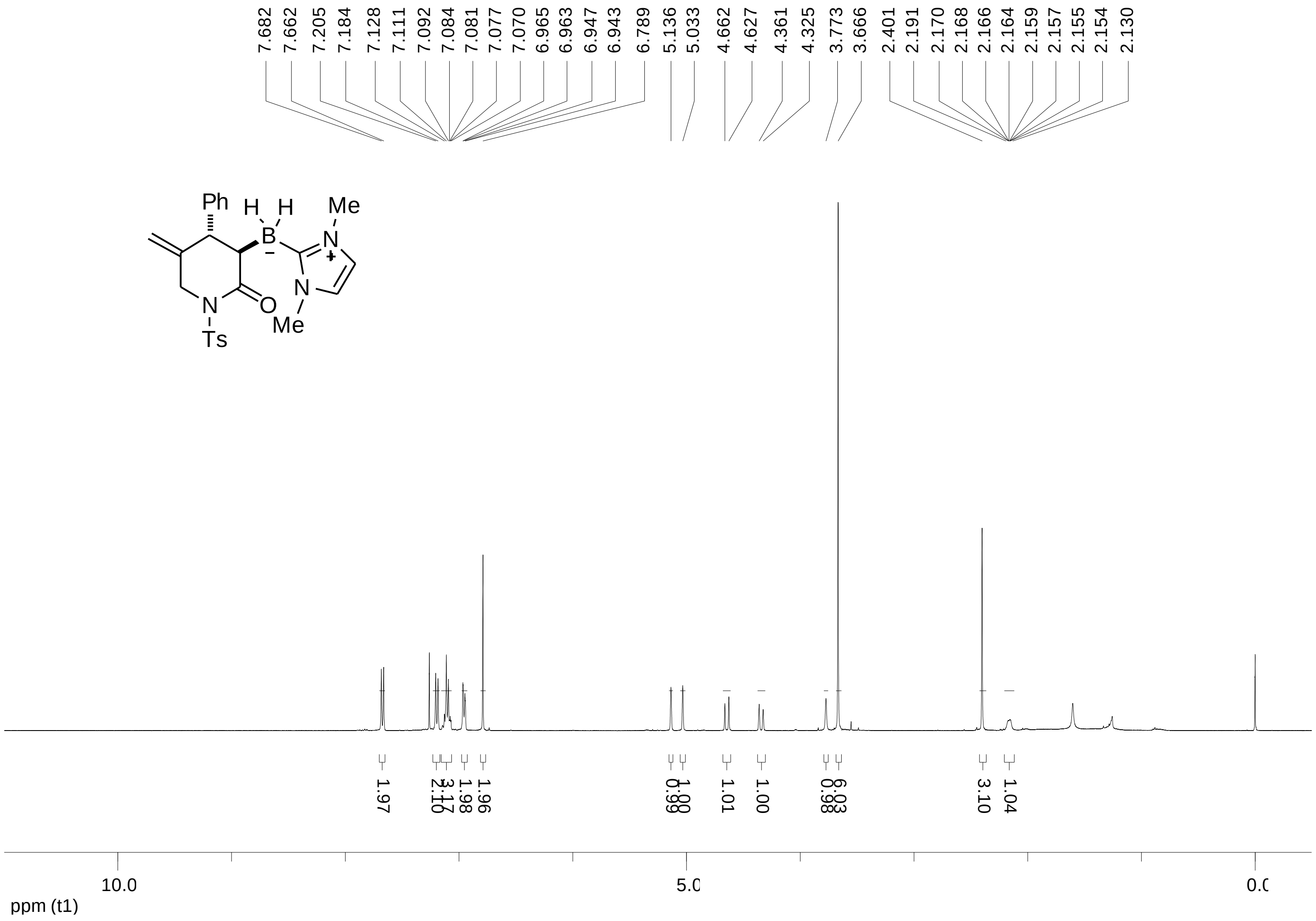

Supplementary Figure 120. ^{1}H NMR spectrum for 3 u′

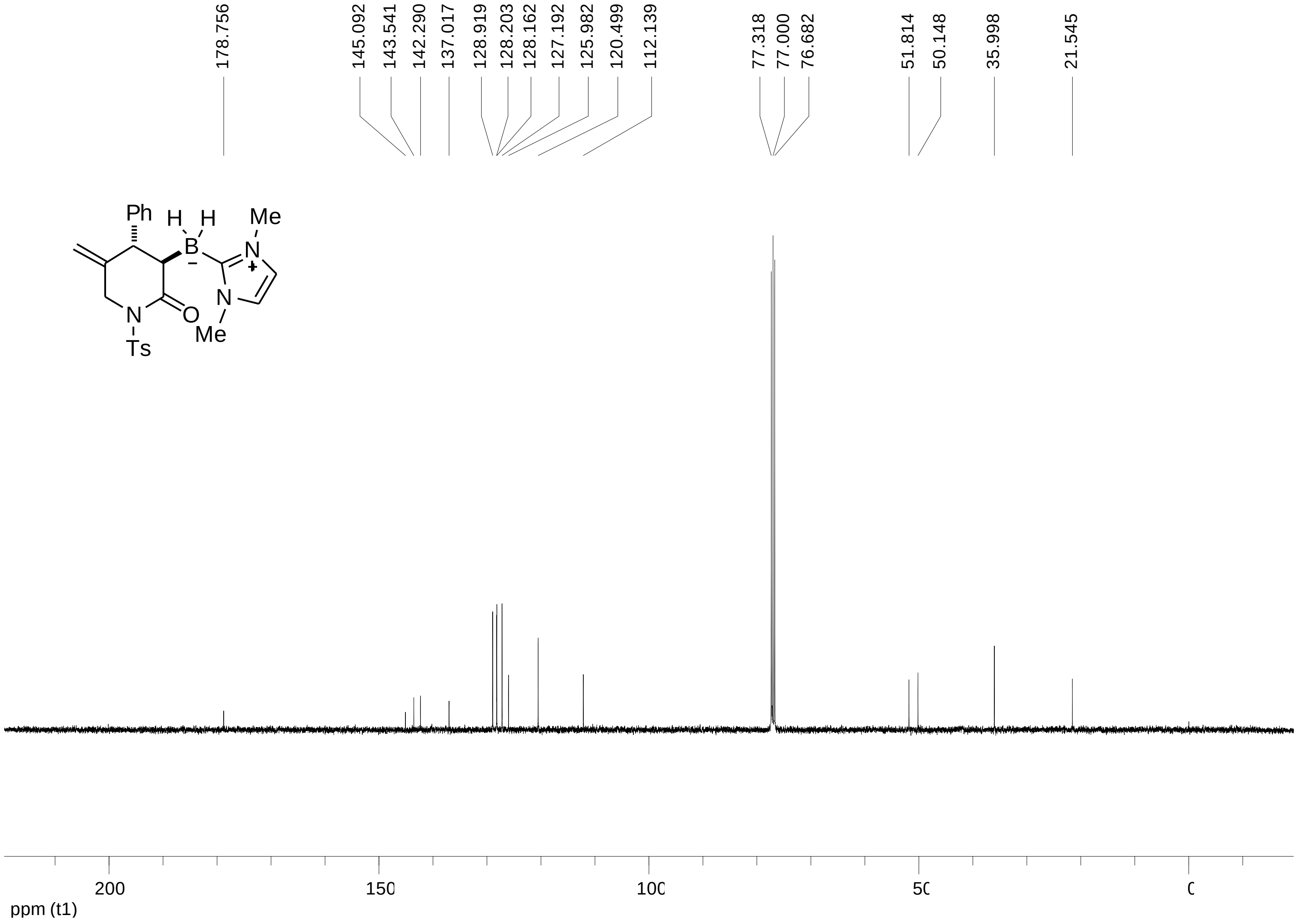

Supplementary Figure 121. ^{13}C NMR spectrum for 3u′

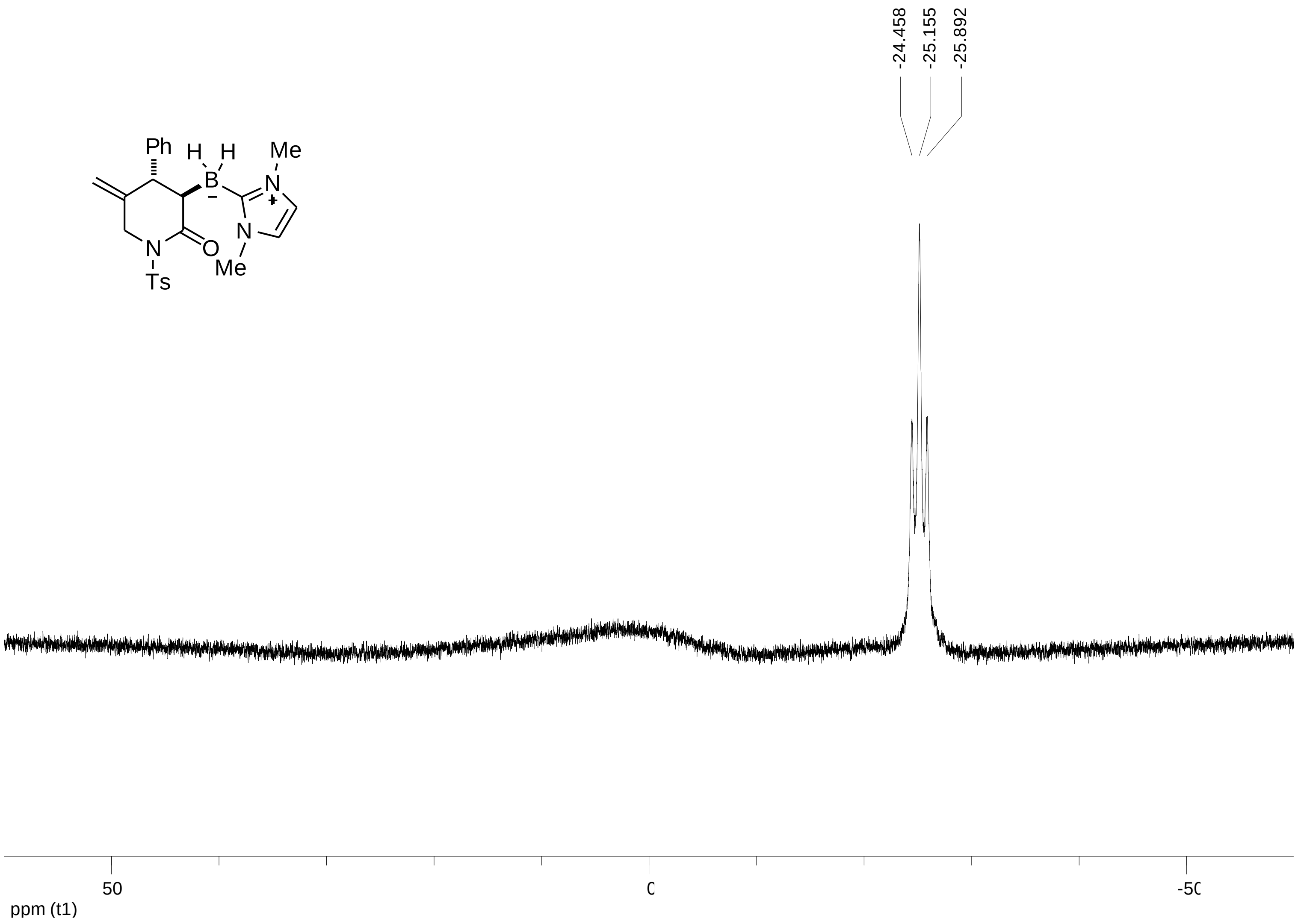

Supplementary Figure 122. ^{11}B NMR spectrum for 3u′

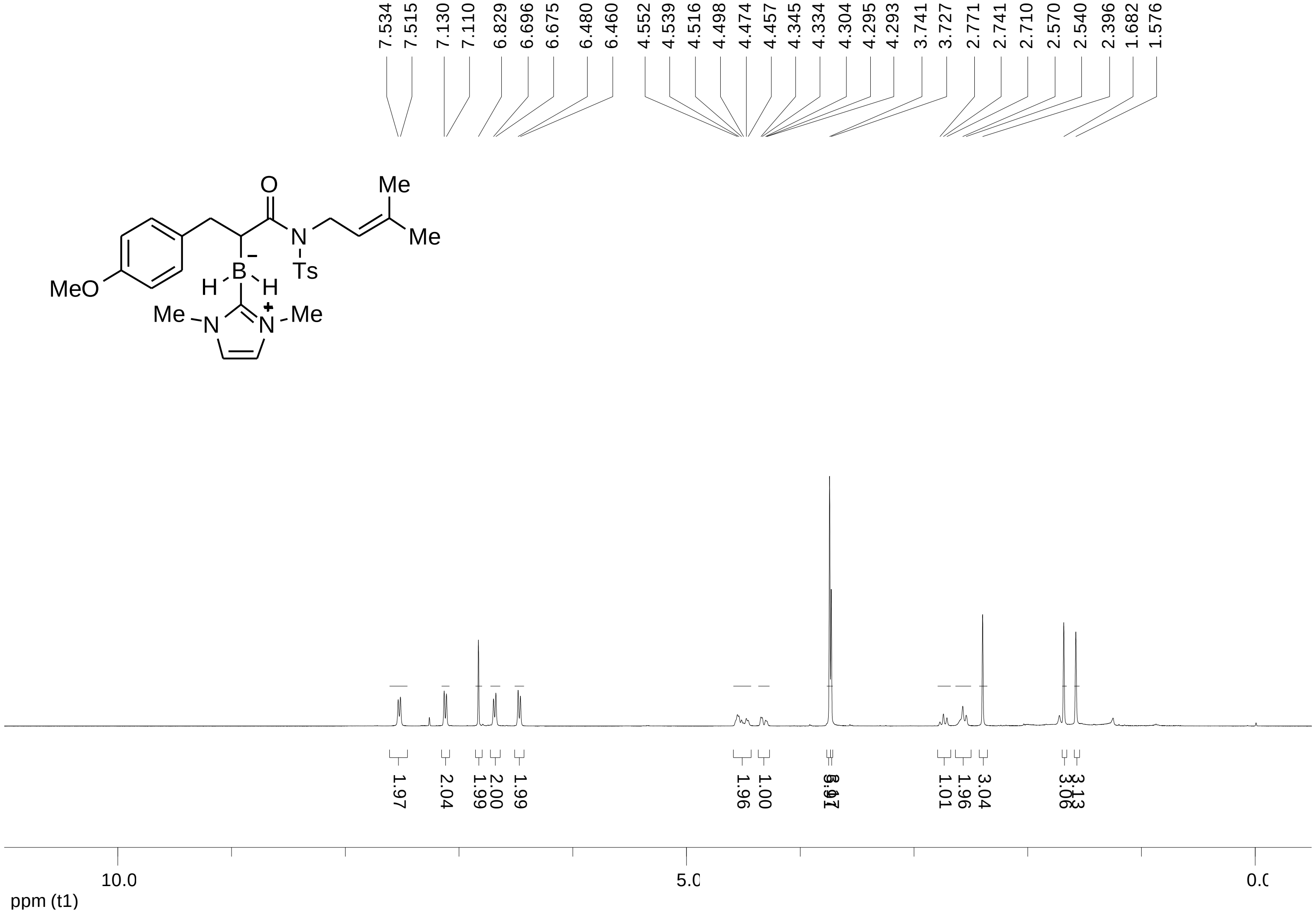

Supplementary Figure 123. ^{1}H NMR spectrum for 3v

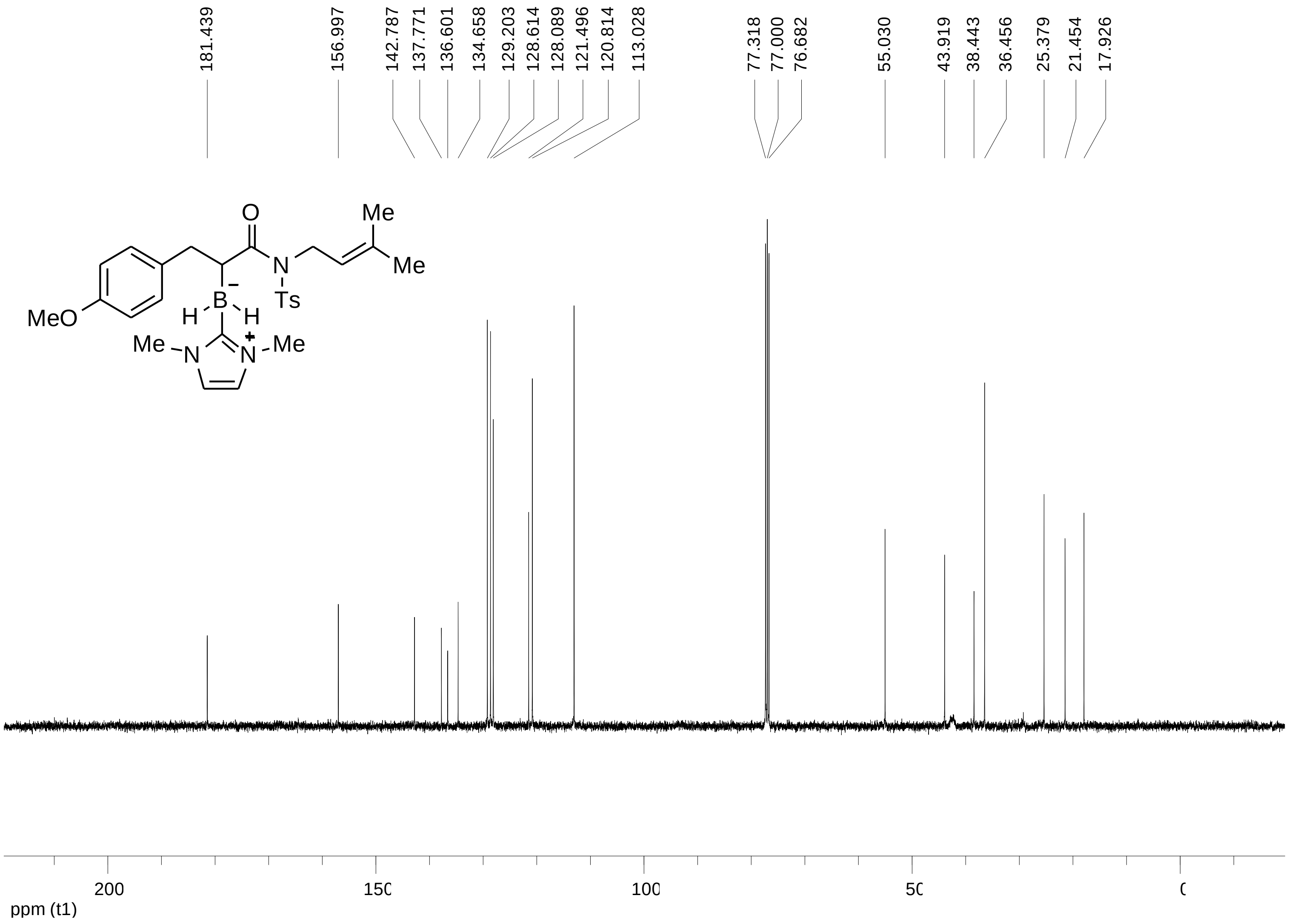

Supplementary Figure 124. ^{13}C NMR spectrum for 3v

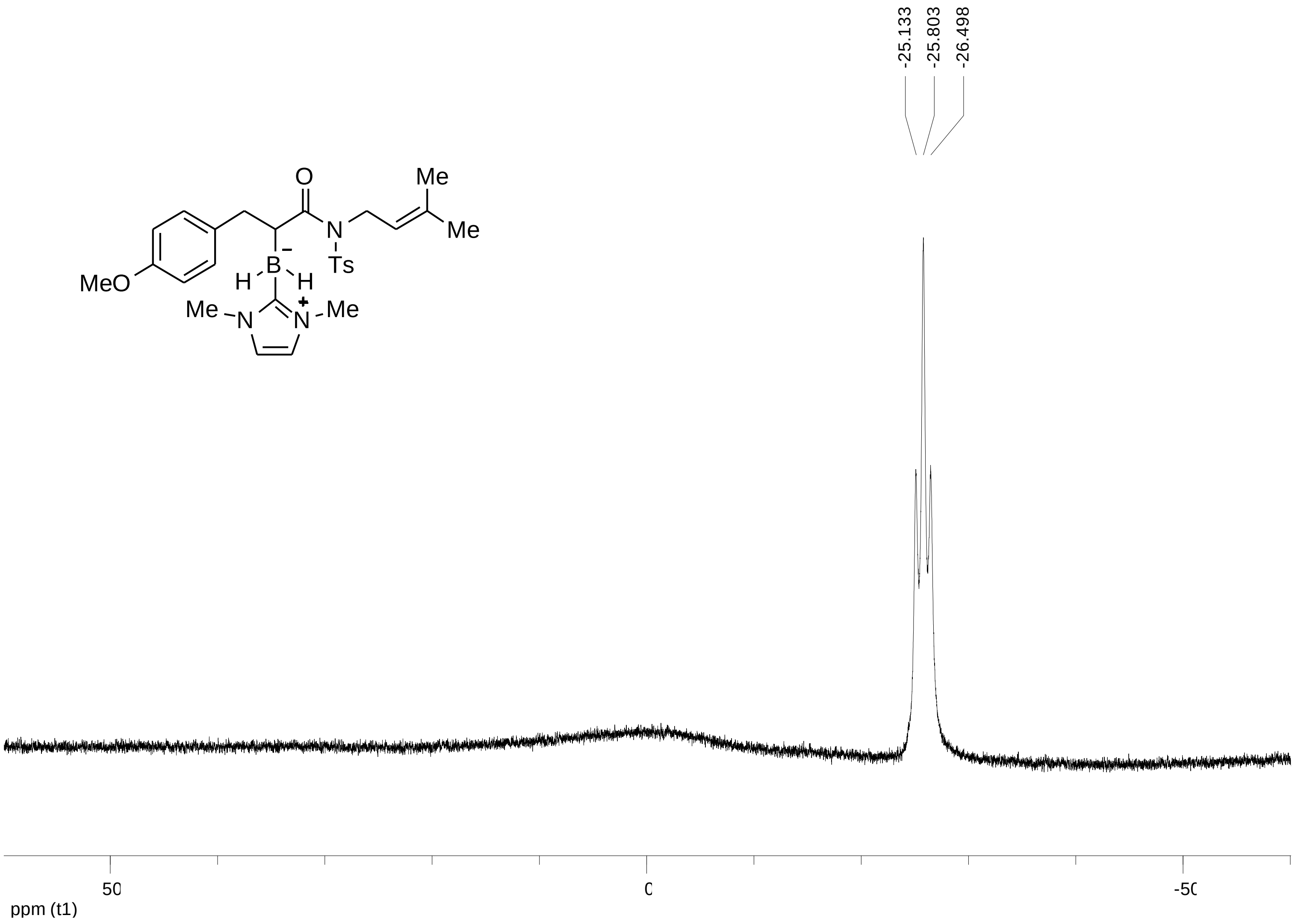

Supplementary Figure 125. ^{11}B NMR spectrum for 3v

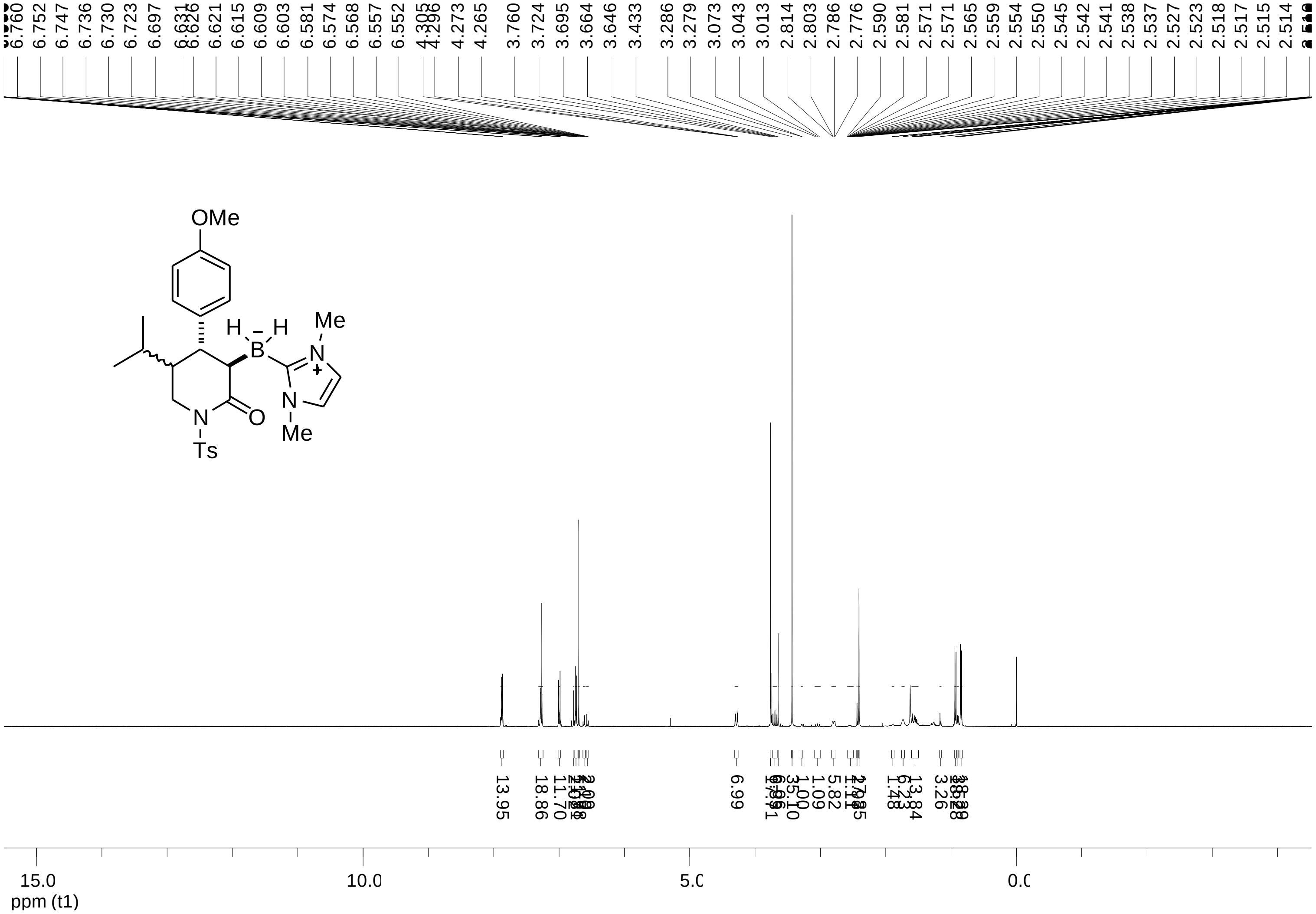

Supplementary Figure 126. ^{1}H NMR spectrum for 3 v′

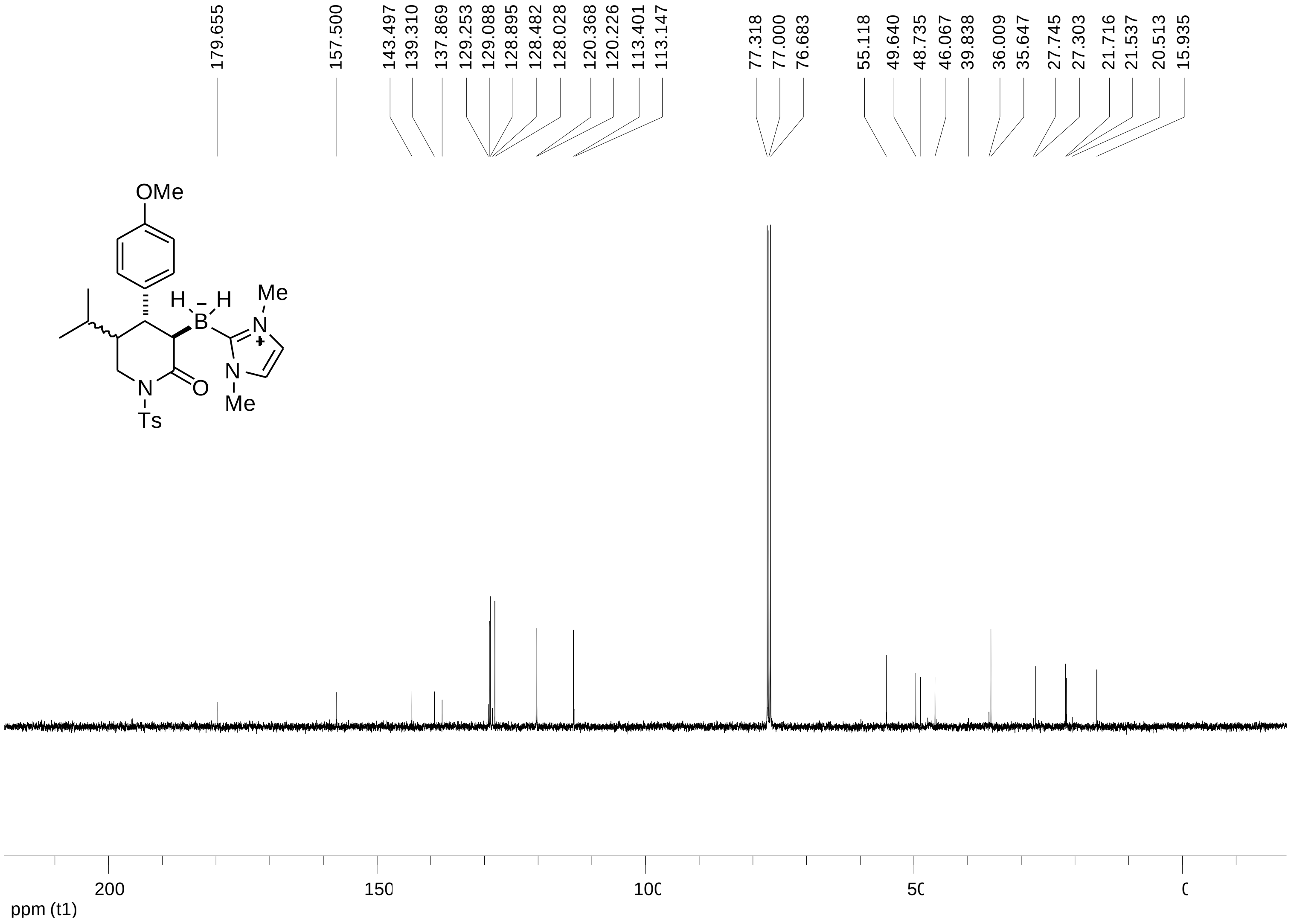

Supplementary Figure 127. ^{13}C NMR spectrum for 3v′

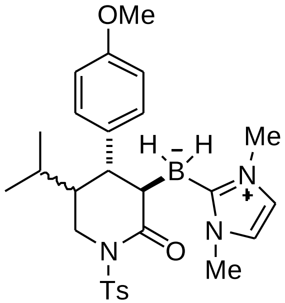

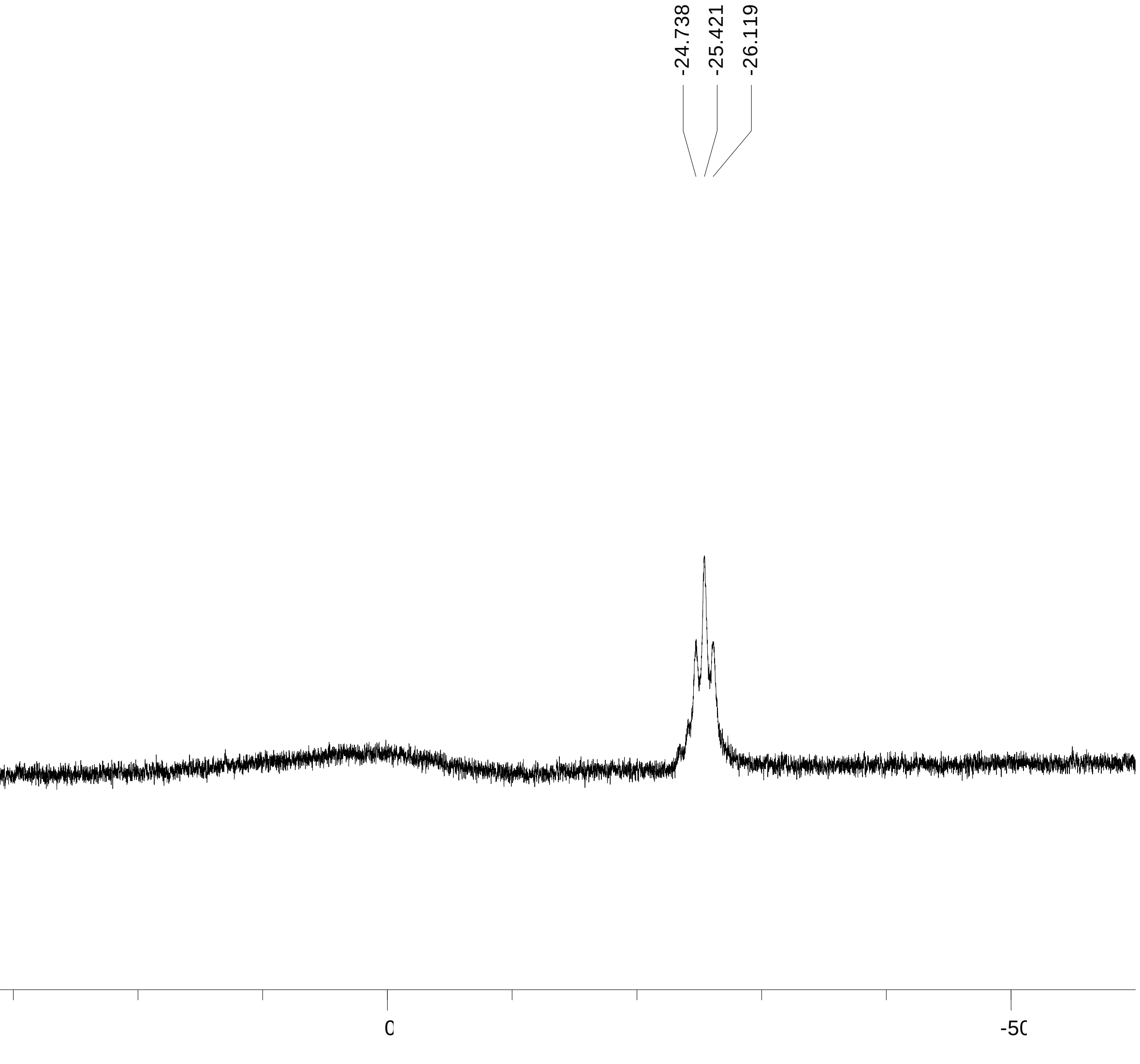

Supplementary Figure 128. ^{11}B NMR spectrum for 3v′

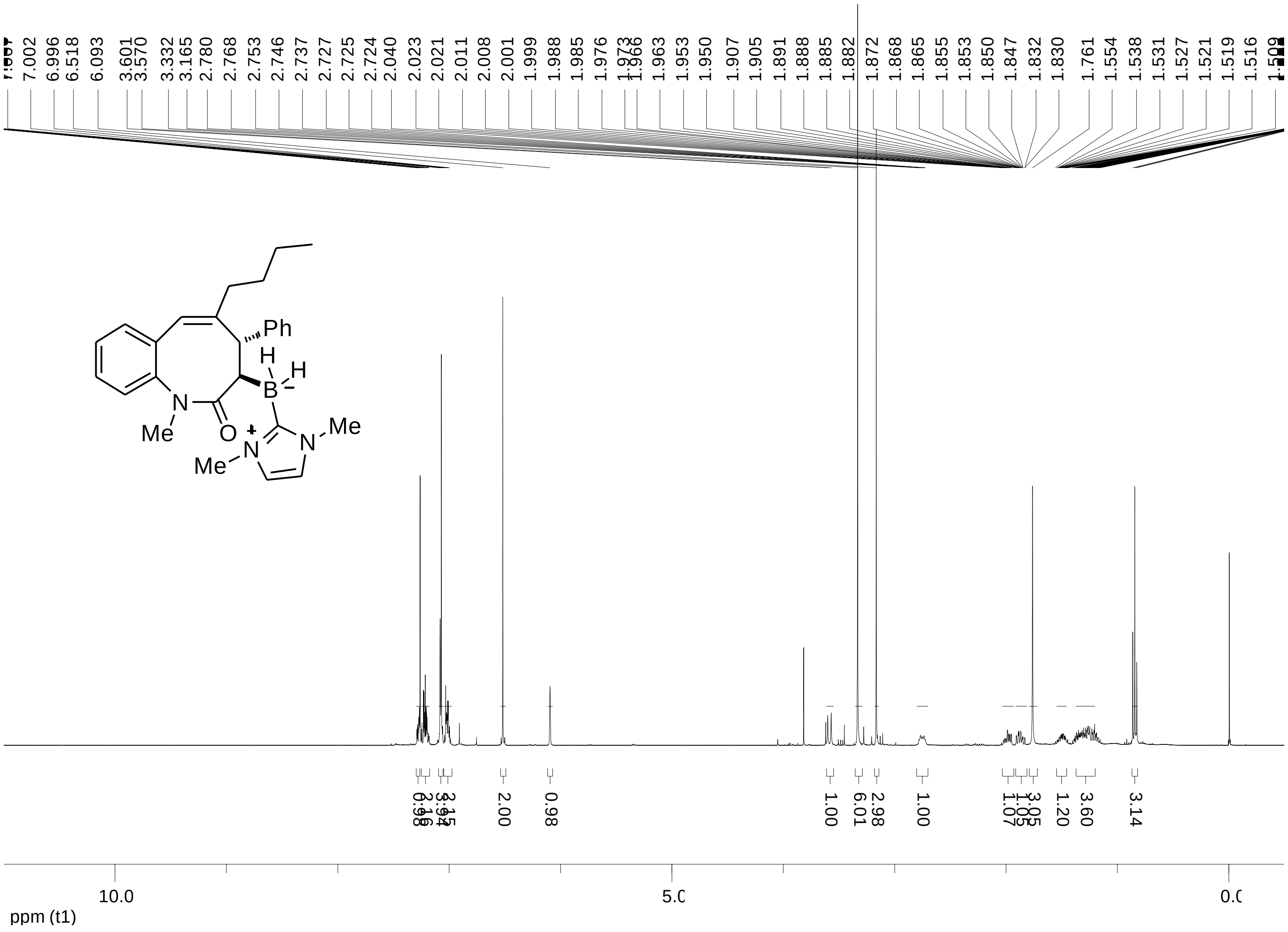

Supplementary Figure 129. ^{1}H NMR spectrum for 3w

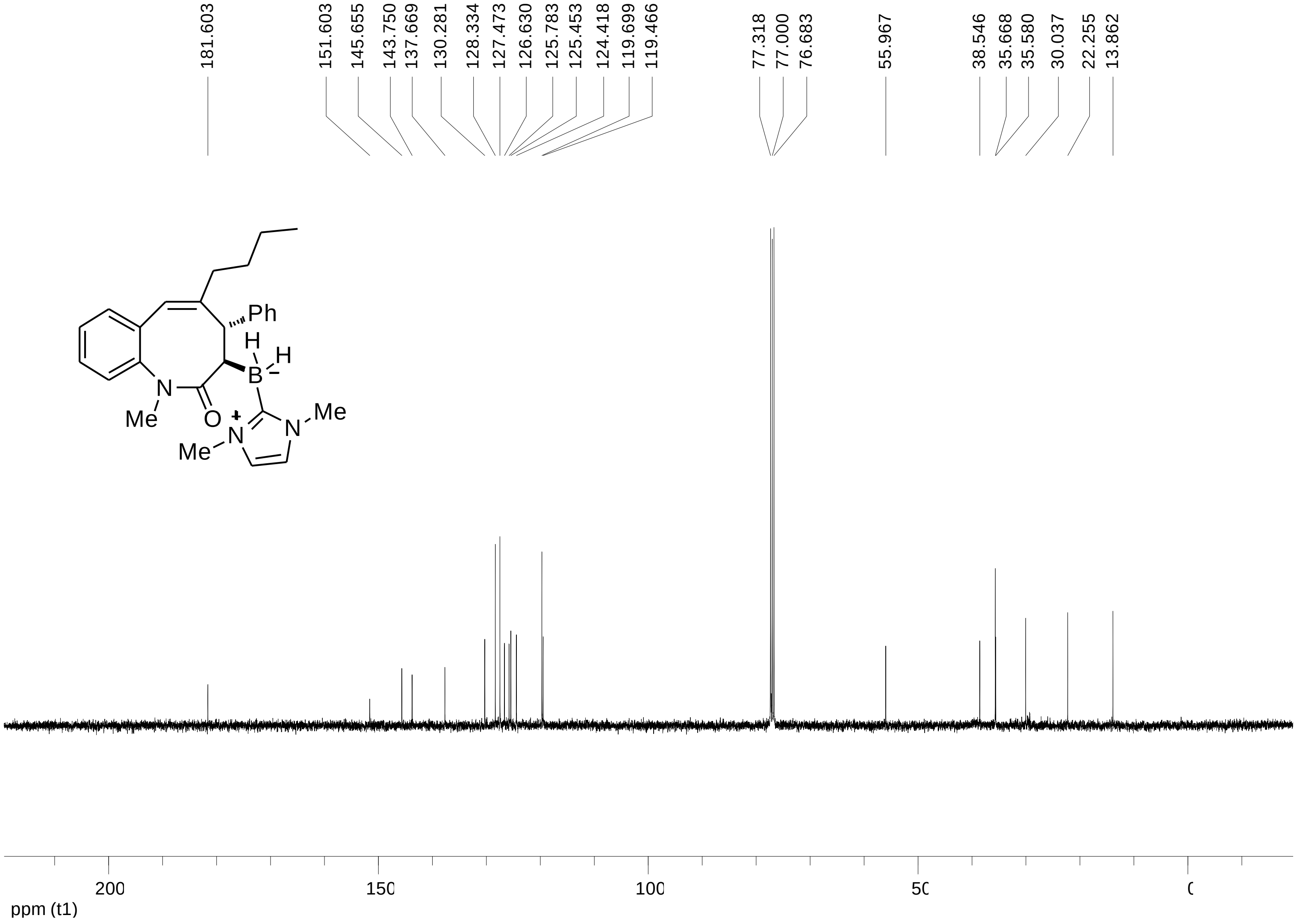

Supplementary Figure 130. ^{13}C NMR spectrum for 3w

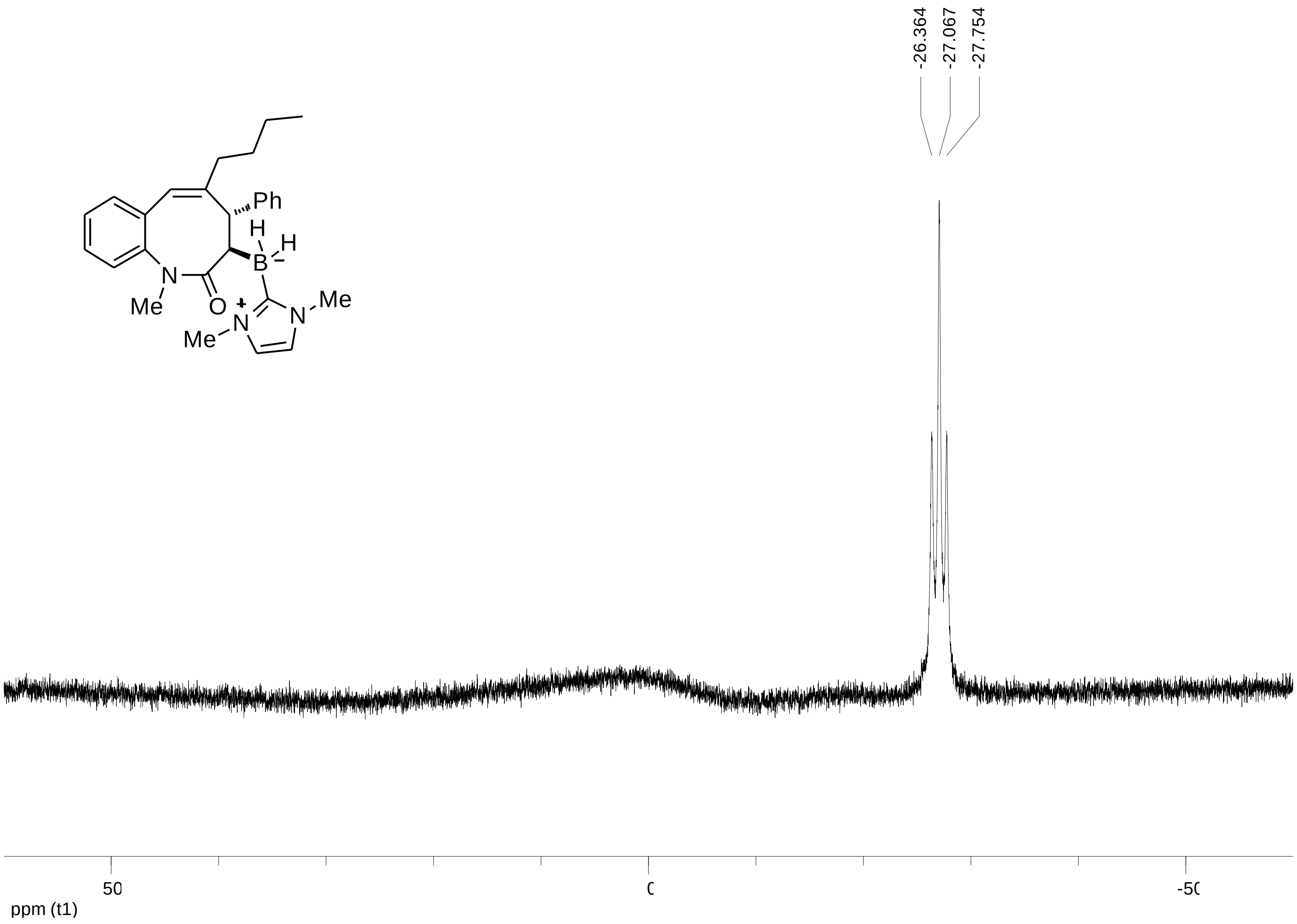

Supplementary Figure 131. ^{11}B NMR spectrum for 3w

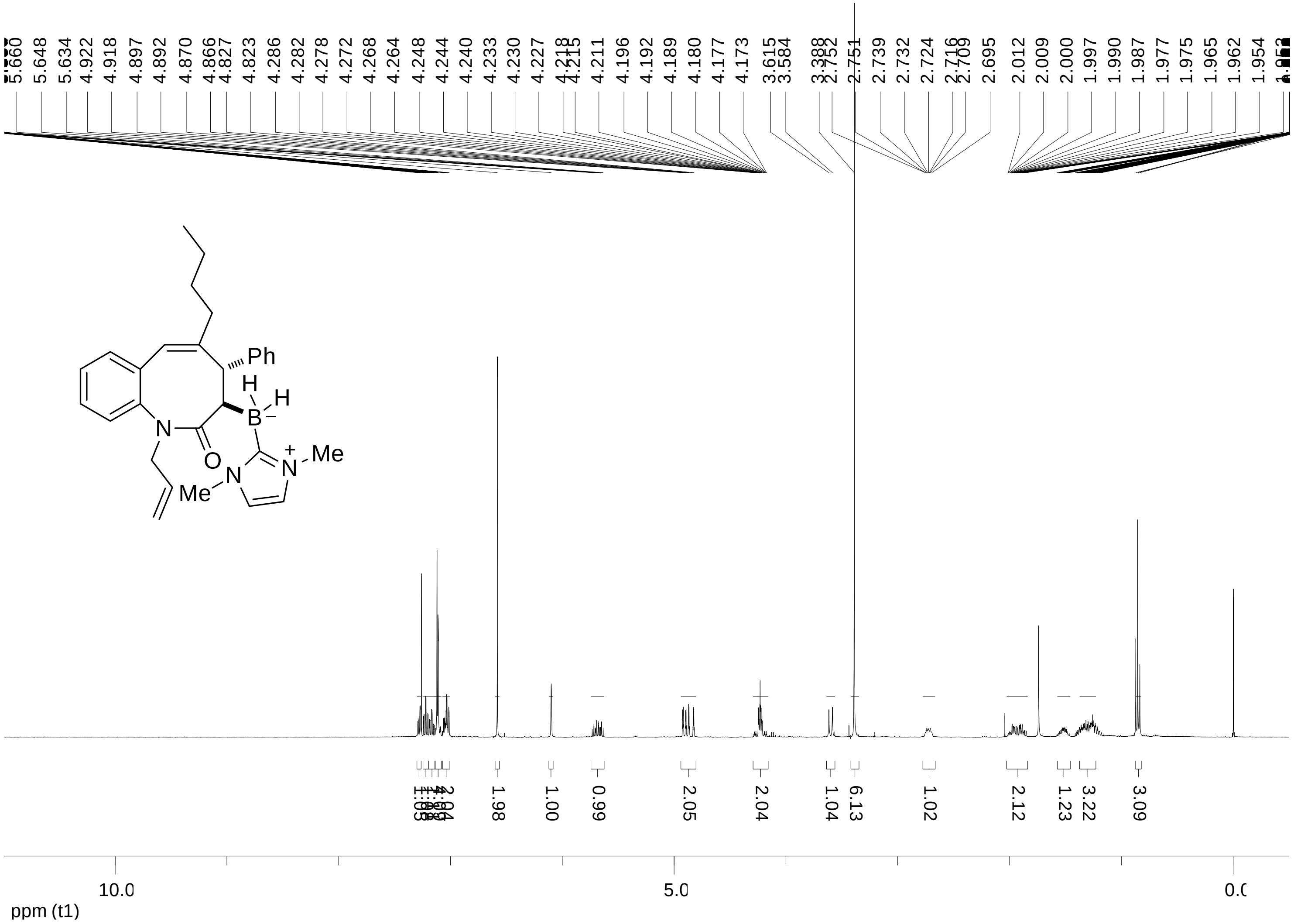

Supplementary Figure 132. ^{1}H NMR spectrum for 3x

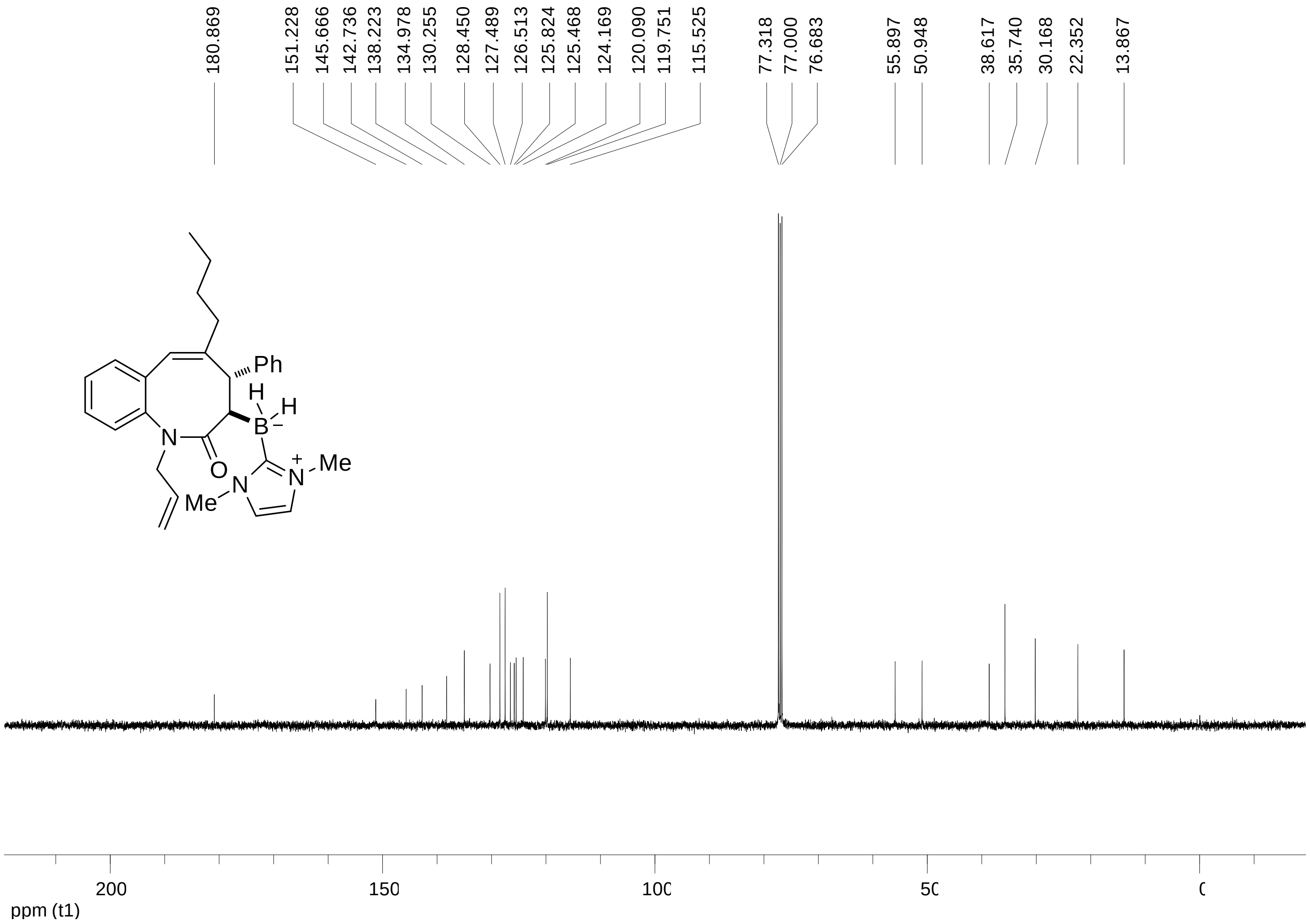

Supplementary Figure 133. ^{13}**C NMR spectrum for 3x**

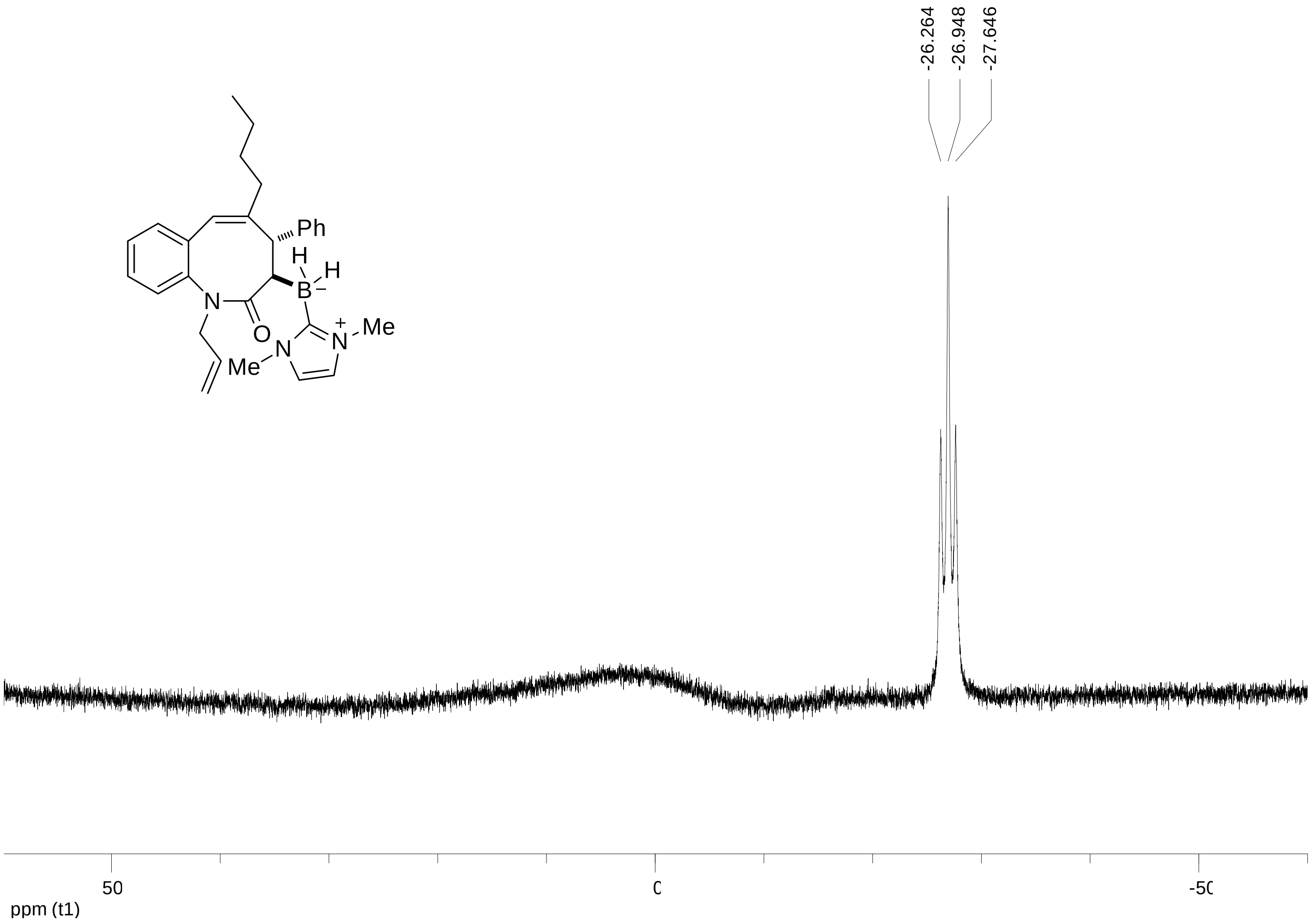

Supplementary Figure 134. ^{11}B NMR spectrum for 3x

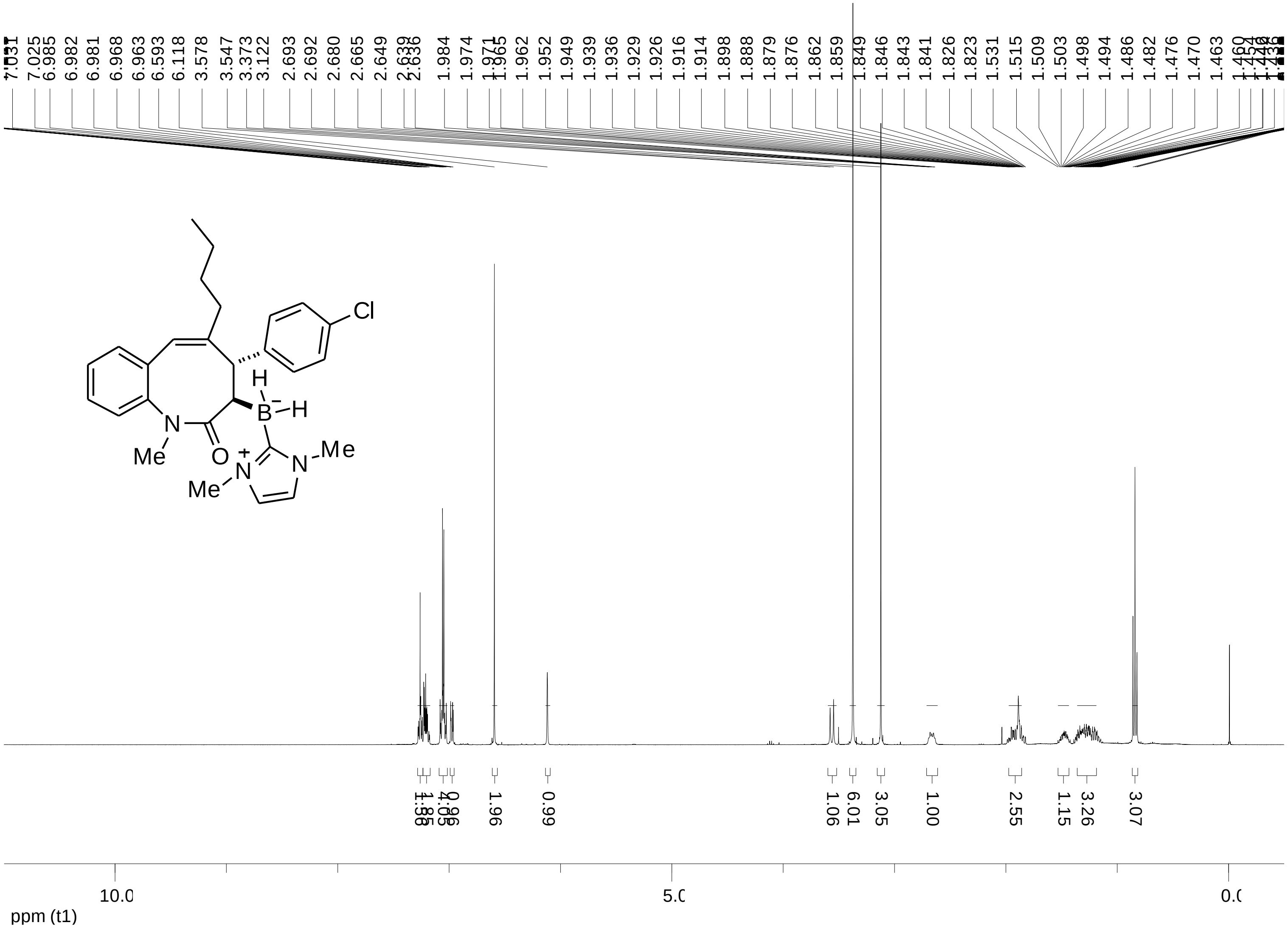

Supplementary Figure 135. ^{1}H NMR spectrum for 3y

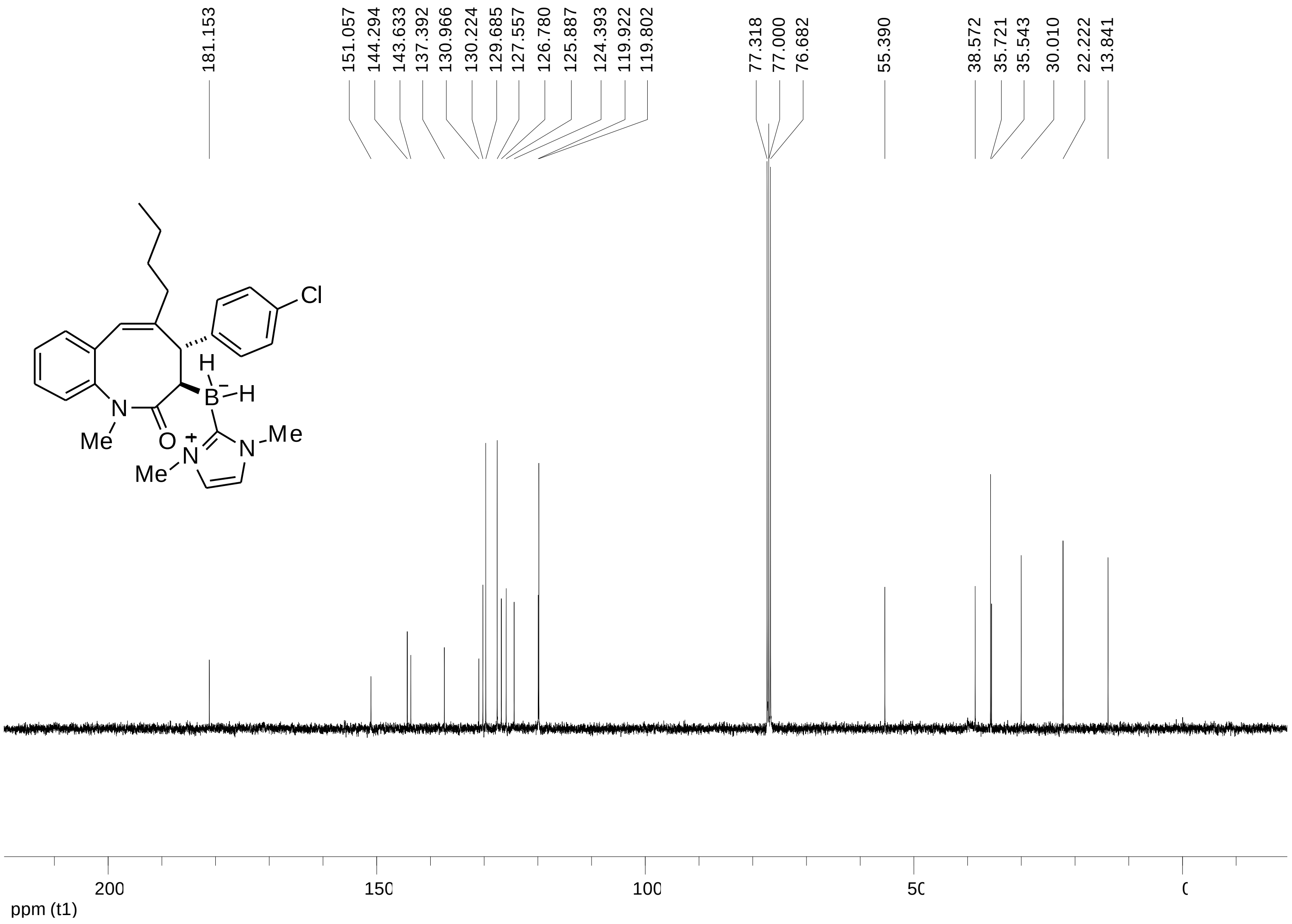

Supplementary Figure 136. ^{13}**C NMR spectrum for 3y**

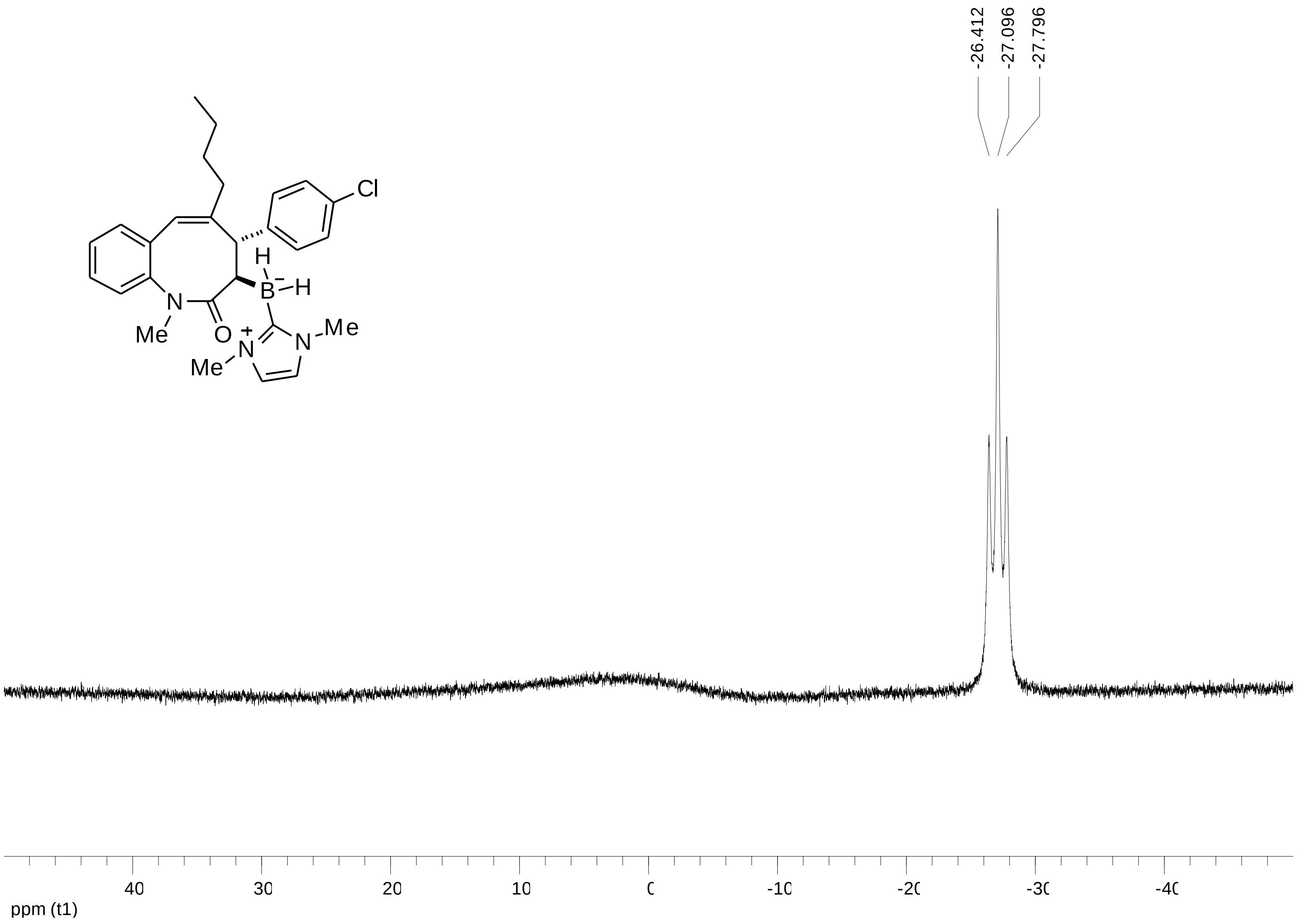

Supplementary Figure 137. ^{11}B NMR spectrum for 3y

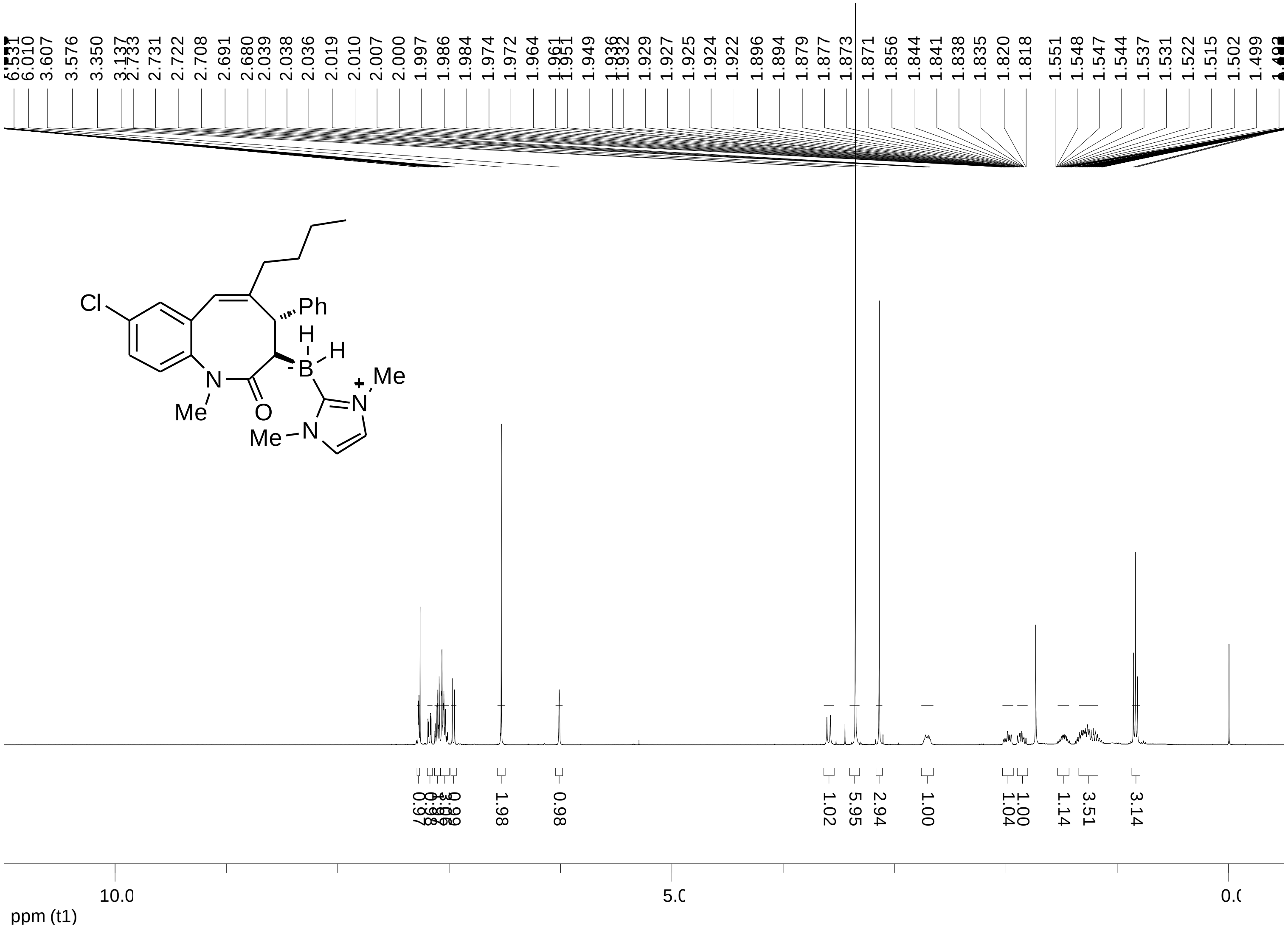

Supplementary Figure 138. ¹H NMR spectrum for 3z

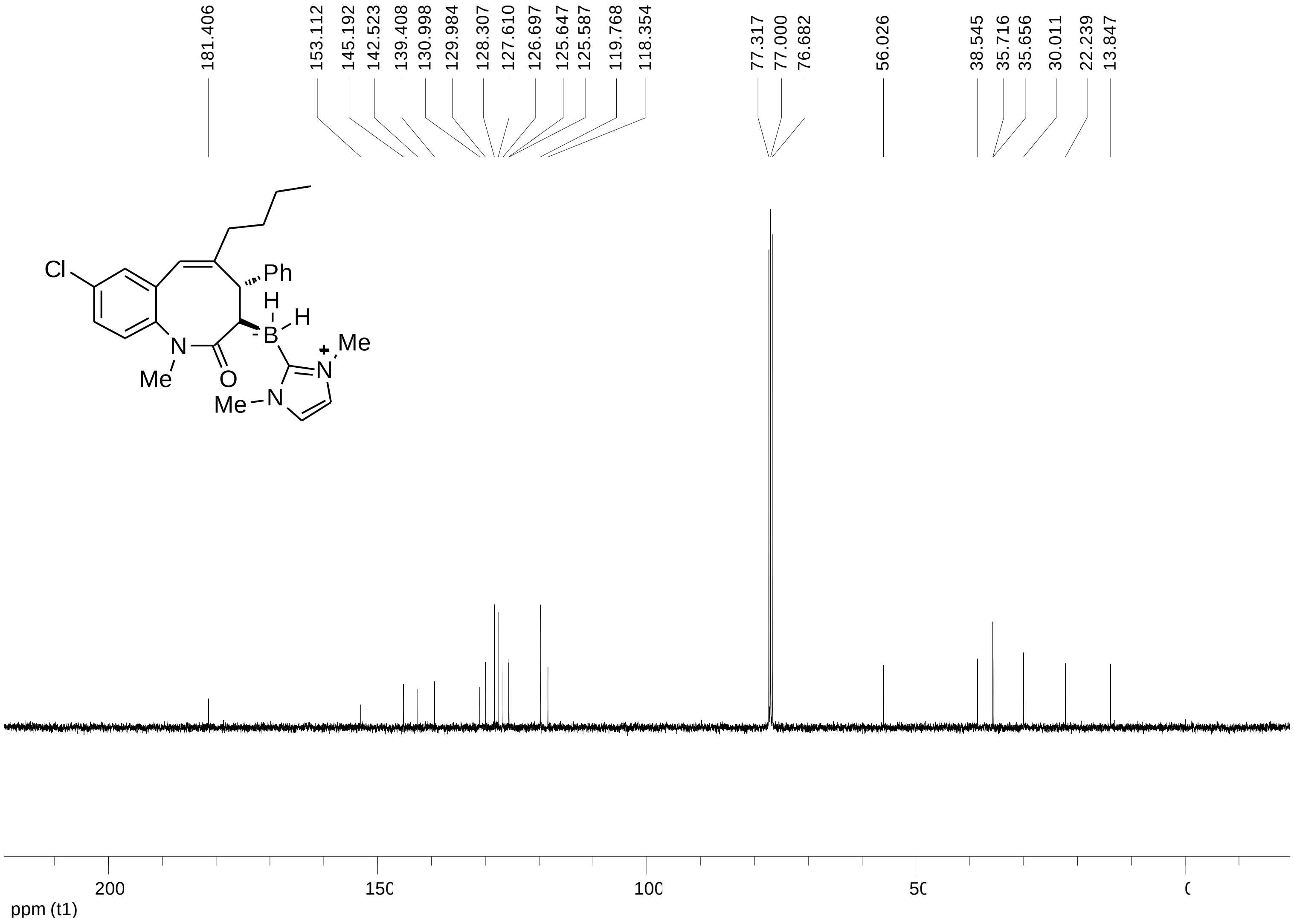

Supplementary Figure 139. ^{13}C NMR spectrum for 3z

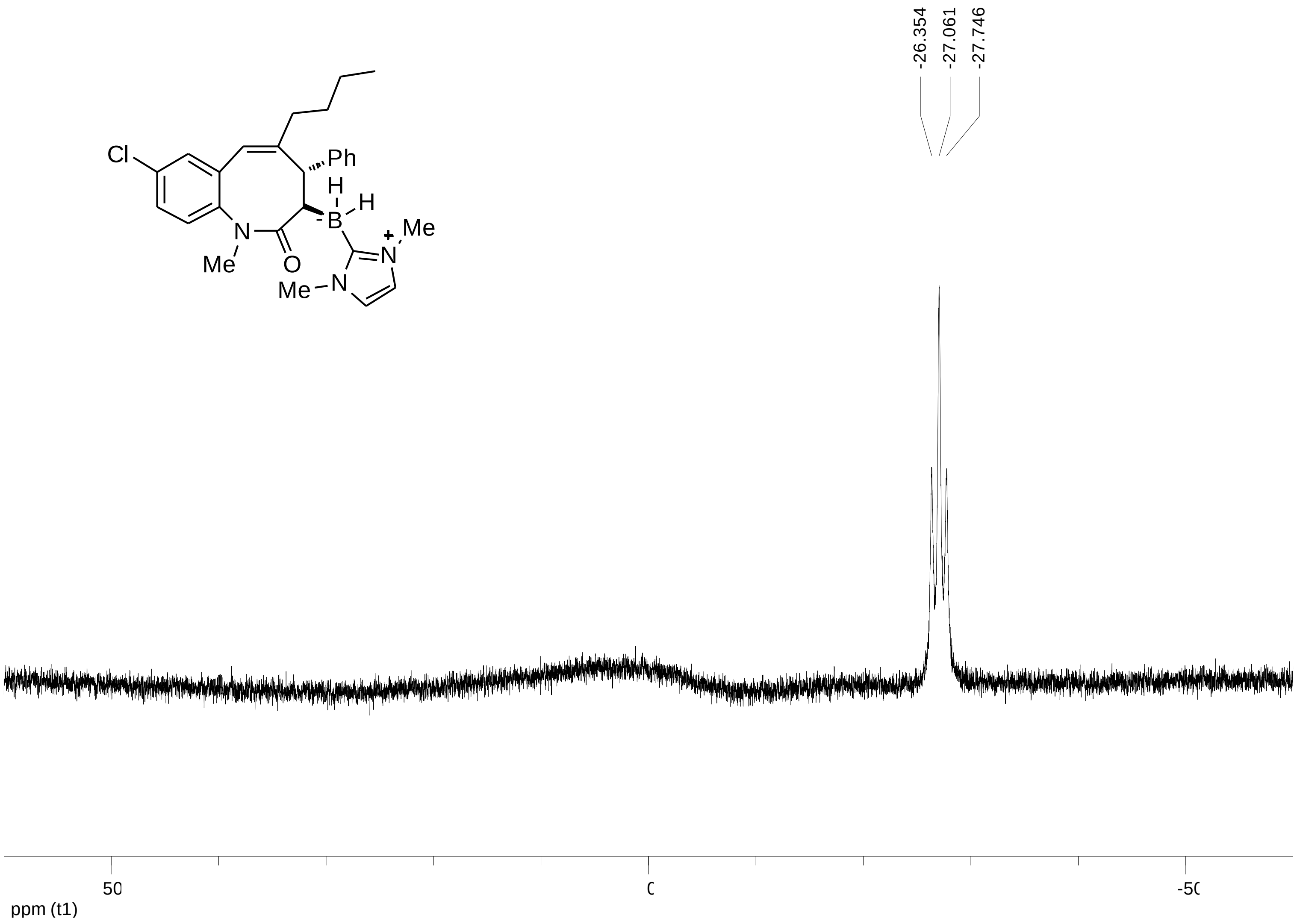

Supplementary Figure 140. ^{11}B NMR spectrum for 3z

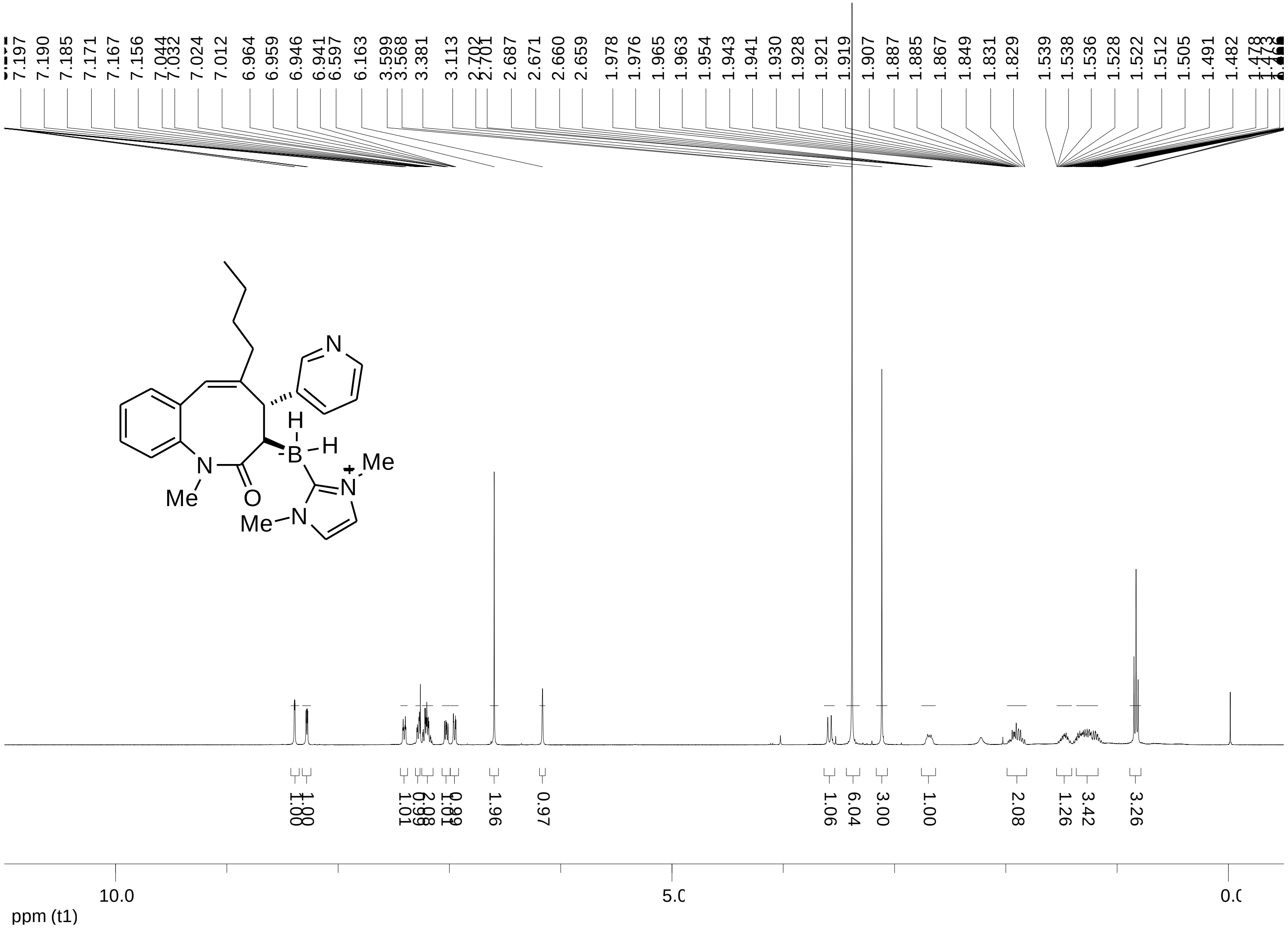

Supplementary Figure 141. ^{1}H NMR spectrum for 3aa

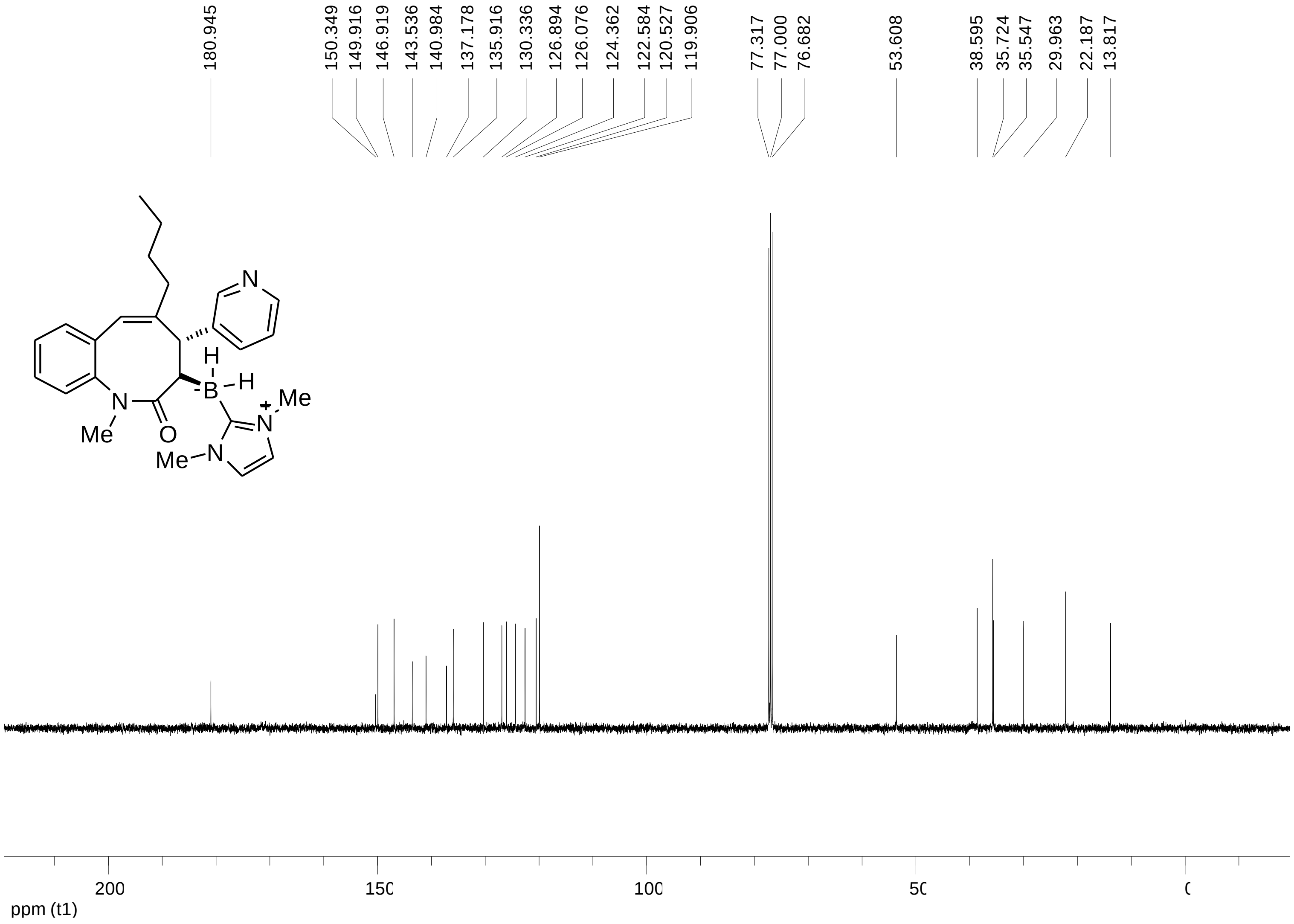

Supplementary Figure 142. ^{13}C NMR spectrum for 3aa

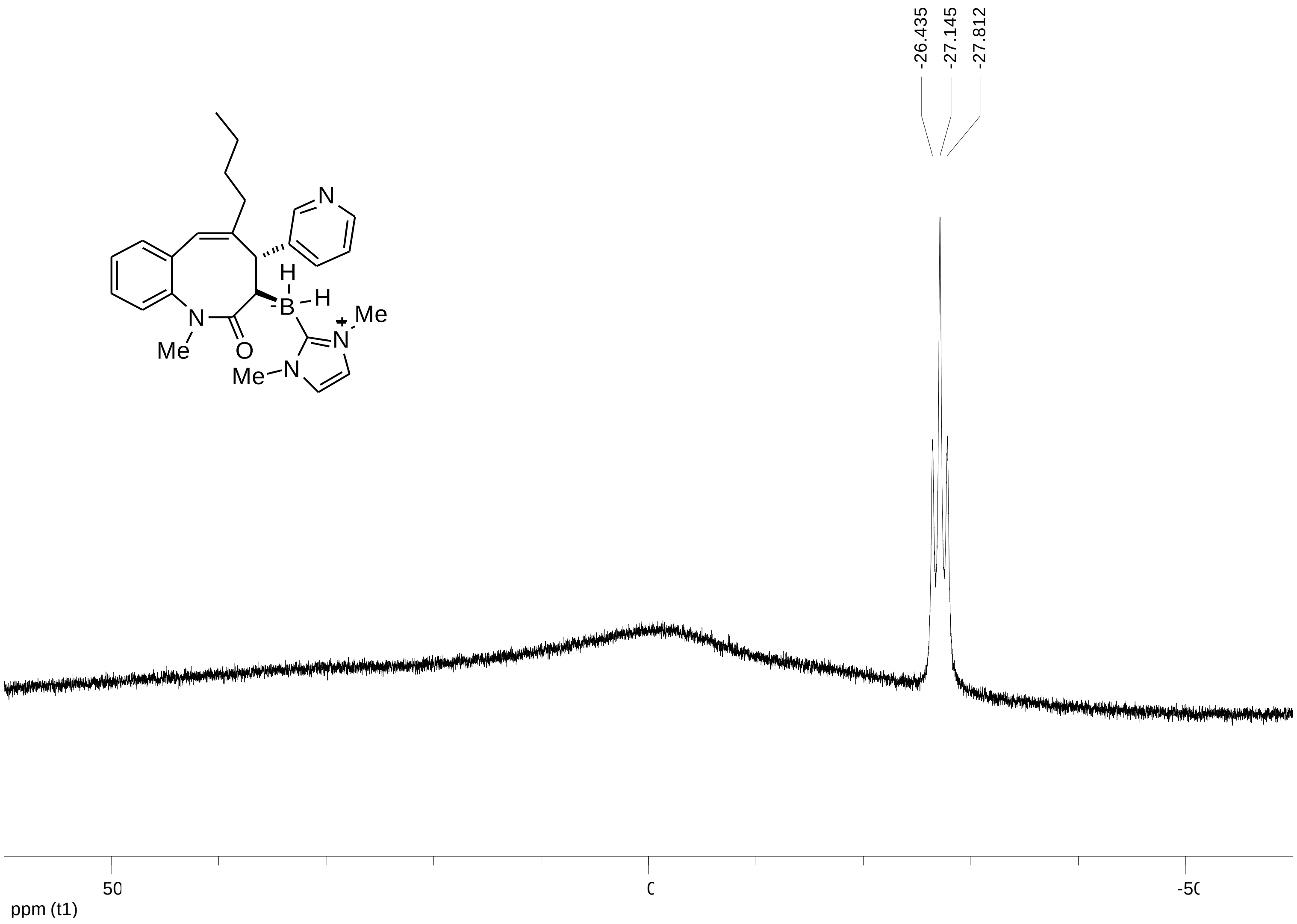

Supplementary Figure 143. ^{11}B NMR spectrum for 3aa

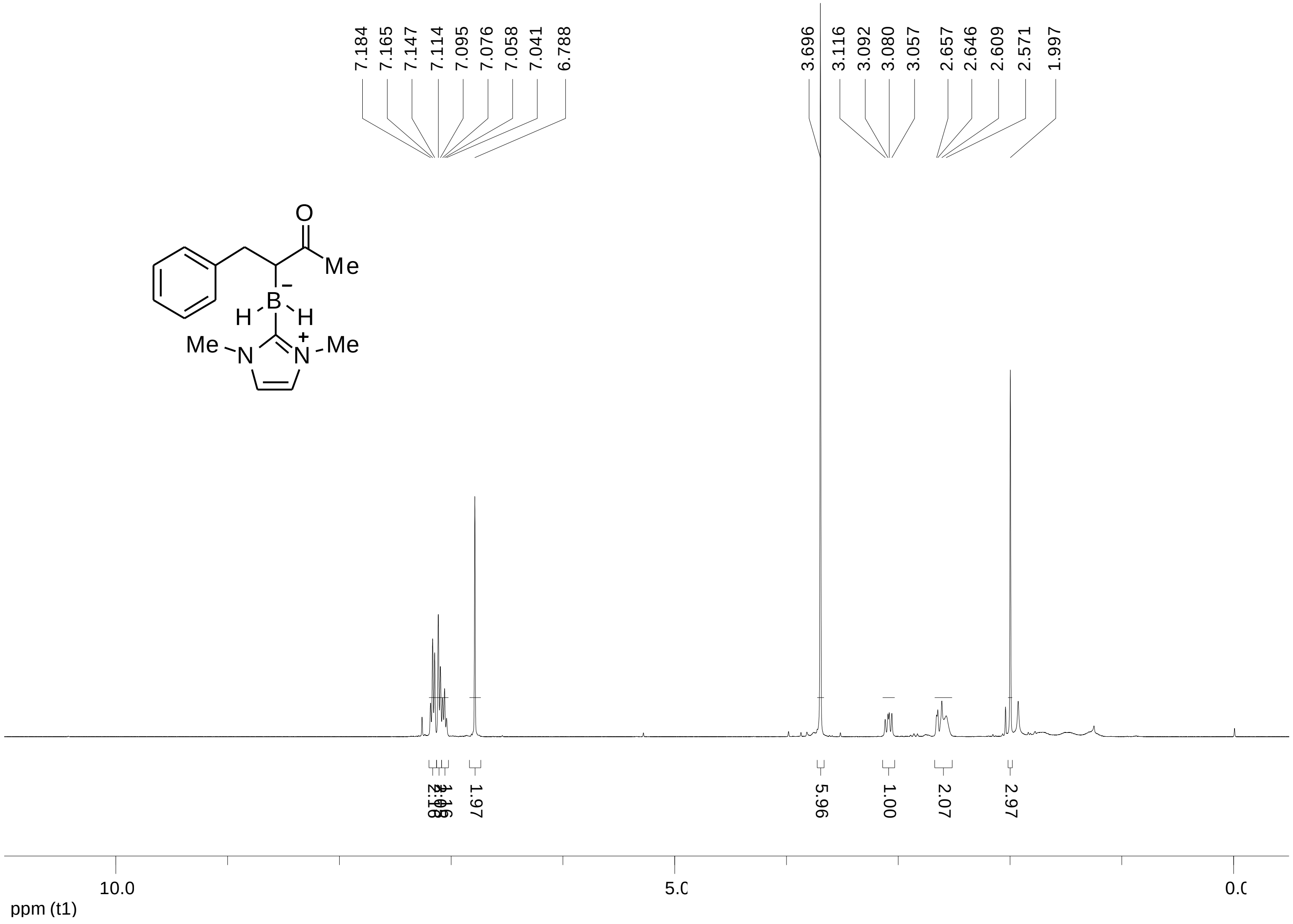

Supplementary Figure 144. ^{1}H NMR spectrum for 3ab

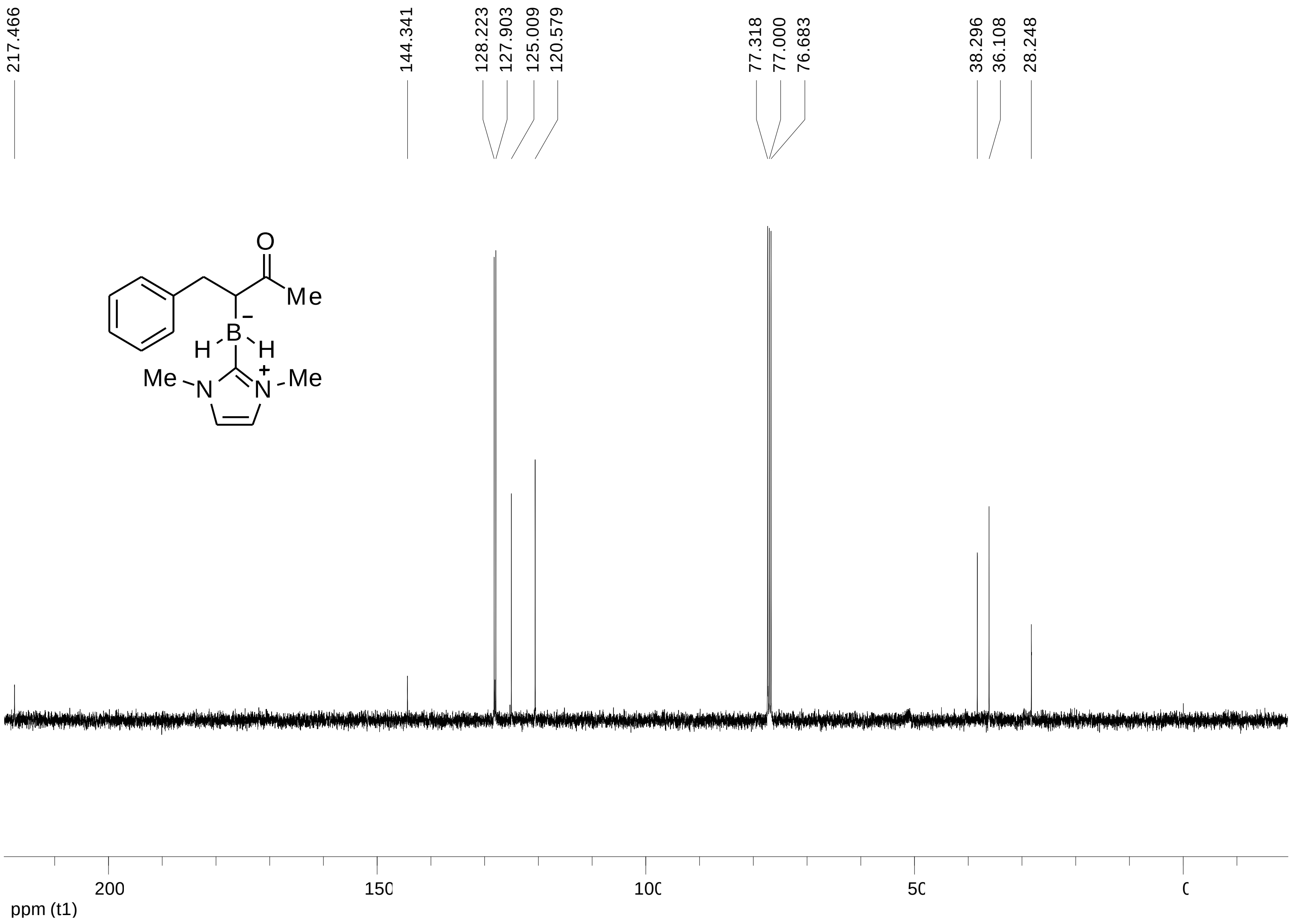

Supplementary Figure 145. ^{13}C NMR spectrum for 3ab

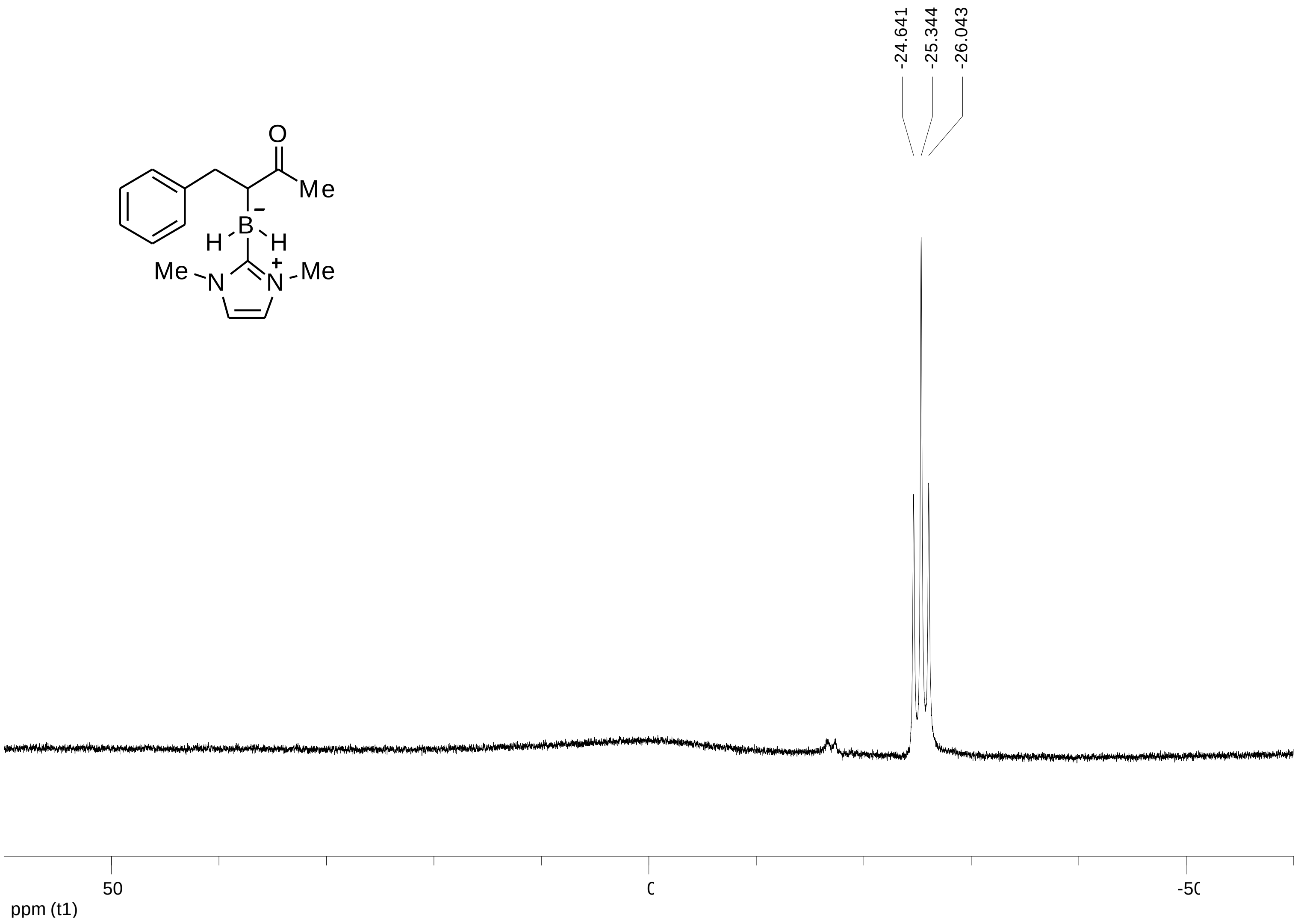

Supplementary Figure 146. ^{11}B NMR spectrum for 3ab

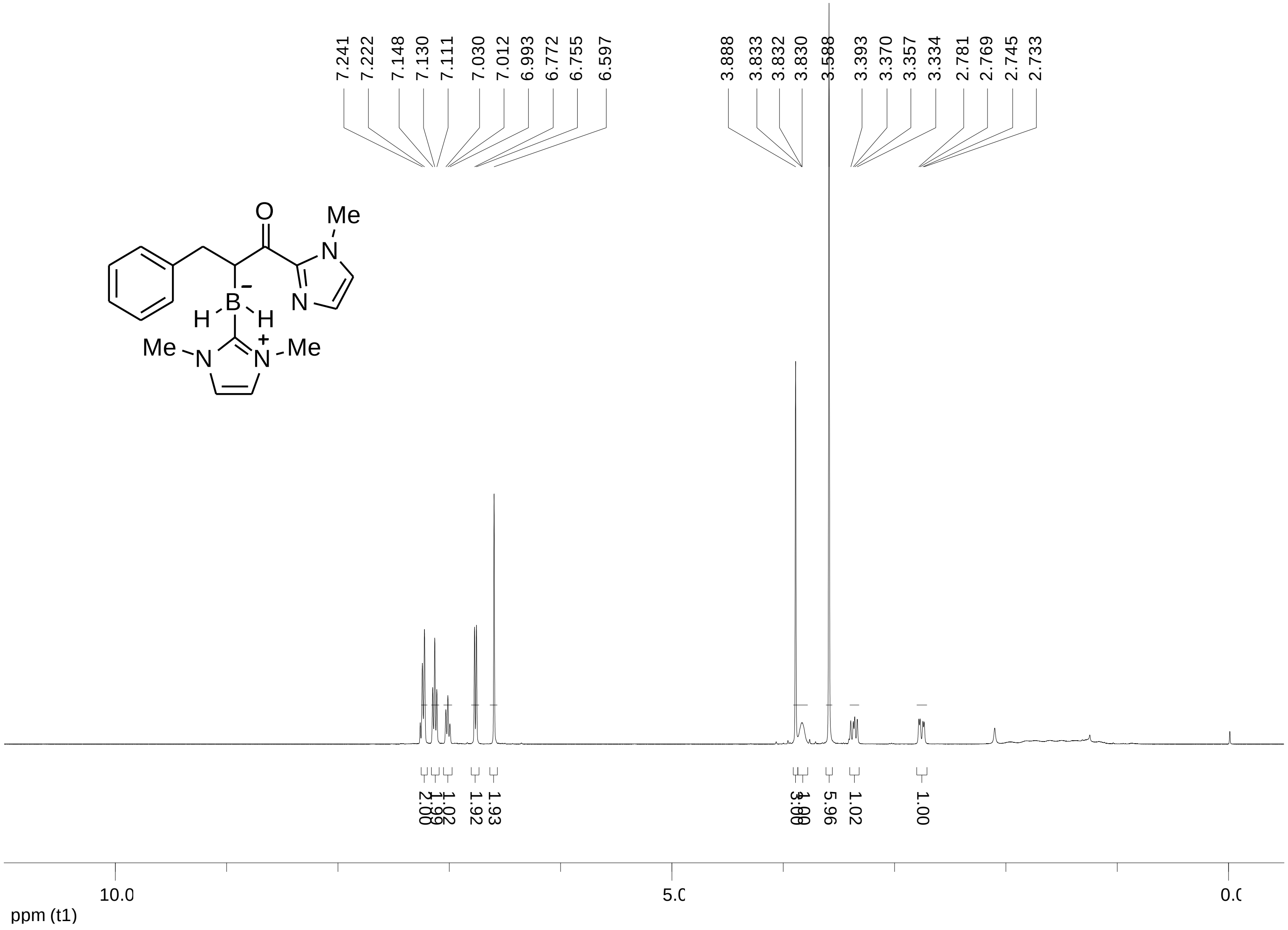

Supplementary Figure 147. ^{1}H NMR spectrum for 3ac

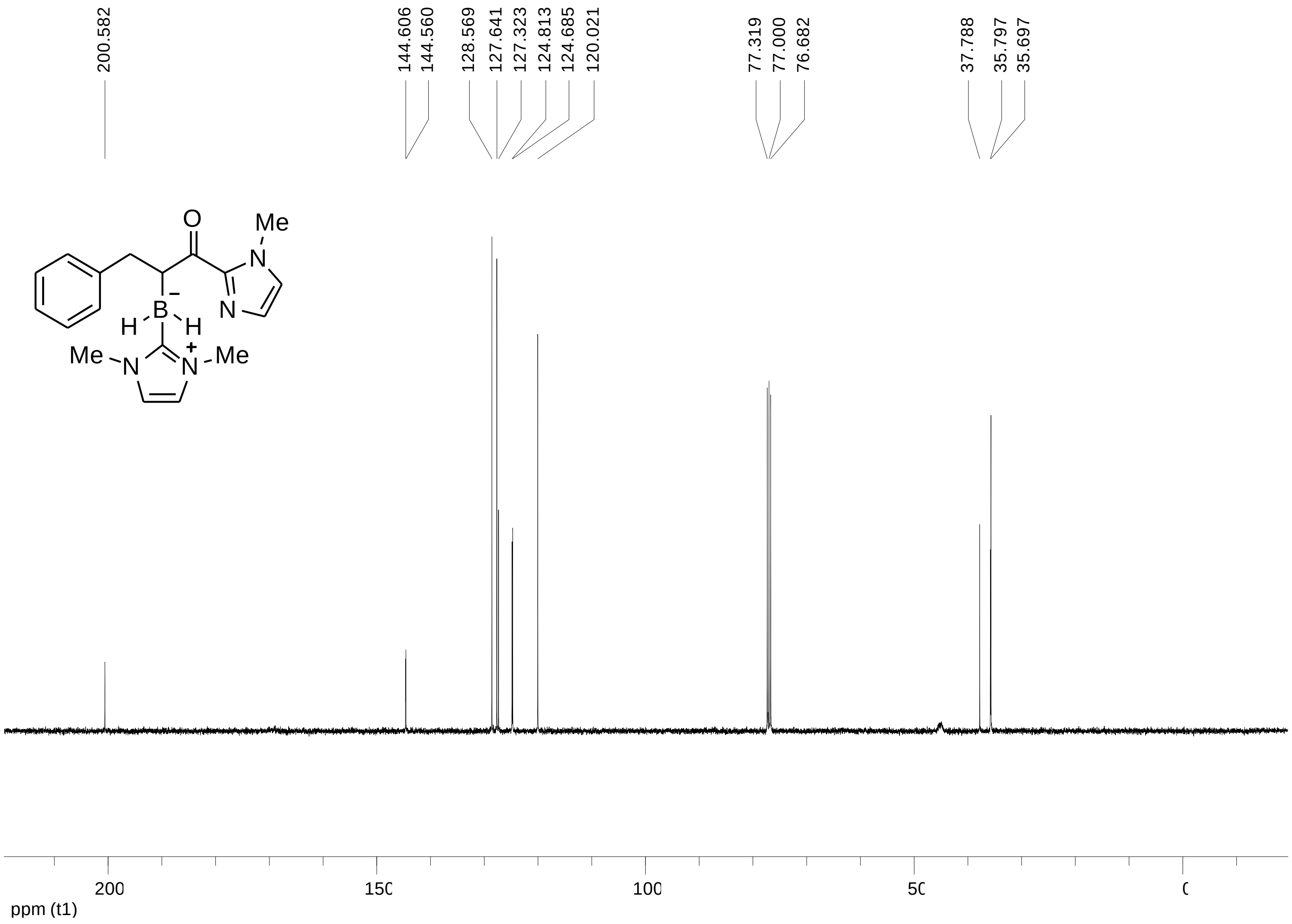

Supplementary Figure 148. ^{13}**C NMR spectrum for 3ac**

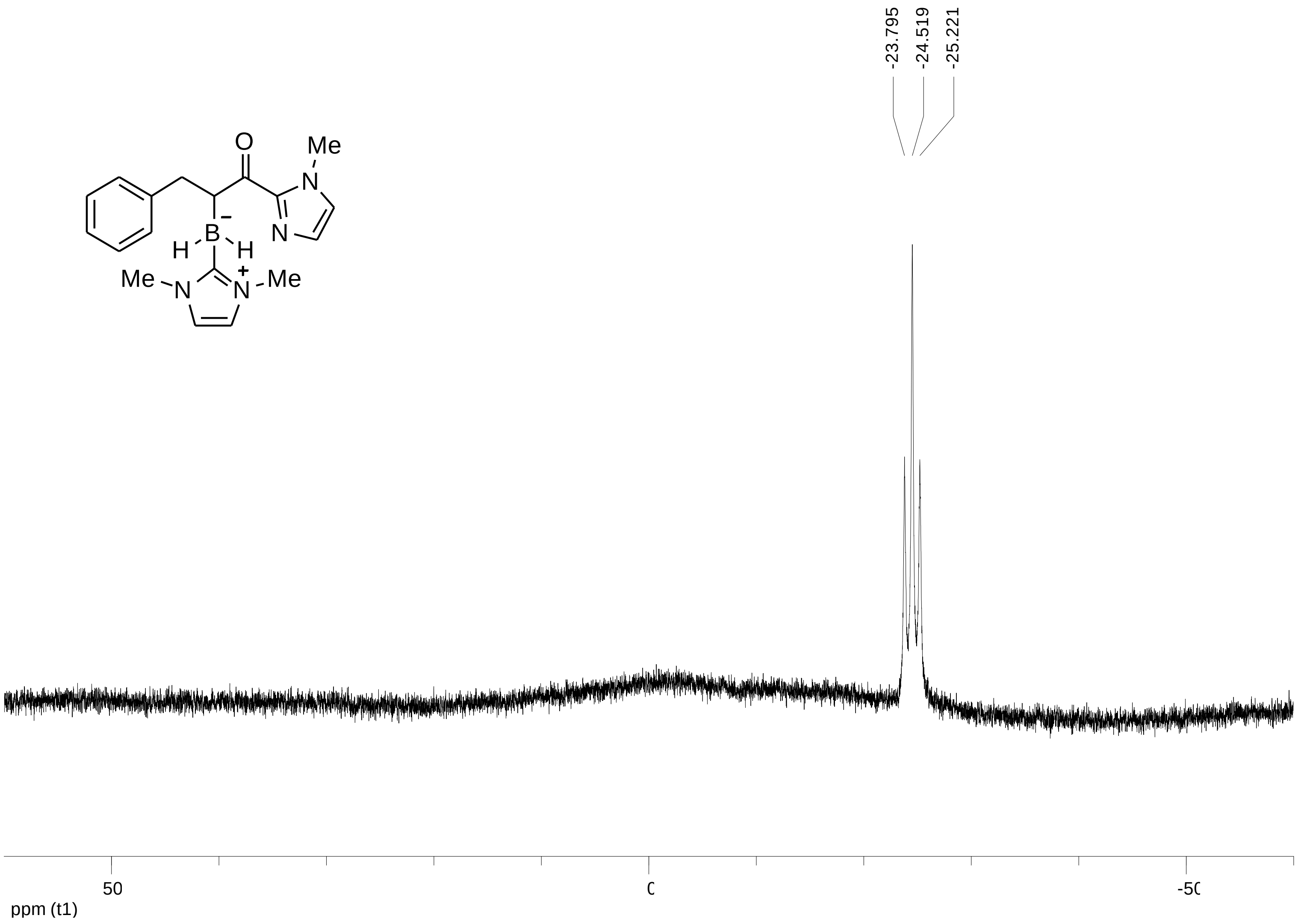

Supplementary Figure 149. ^{11}B NMR spectrum for 3ac

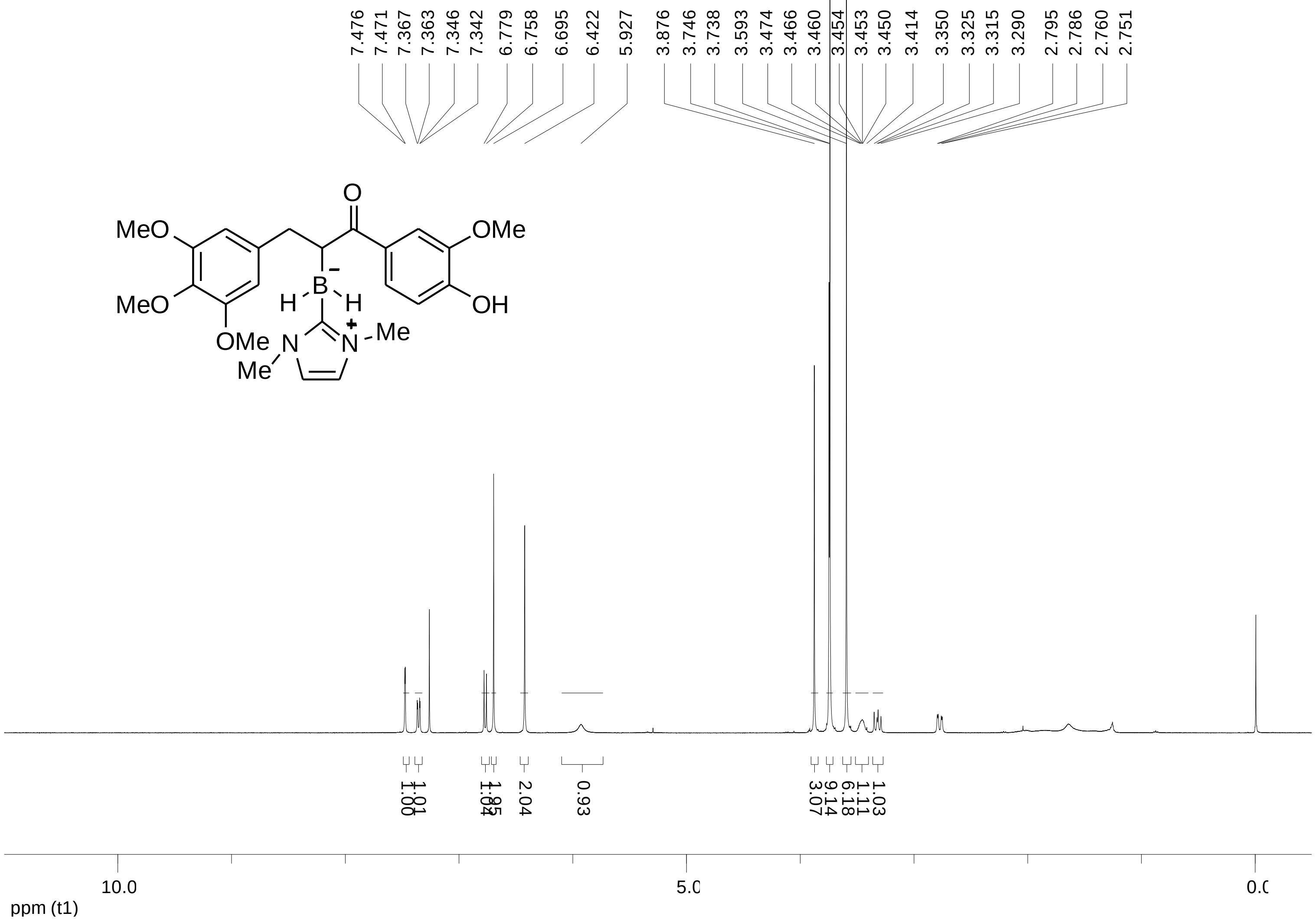

Supplementary Figure 150. ^{1}H NMR spectrum for 3ad

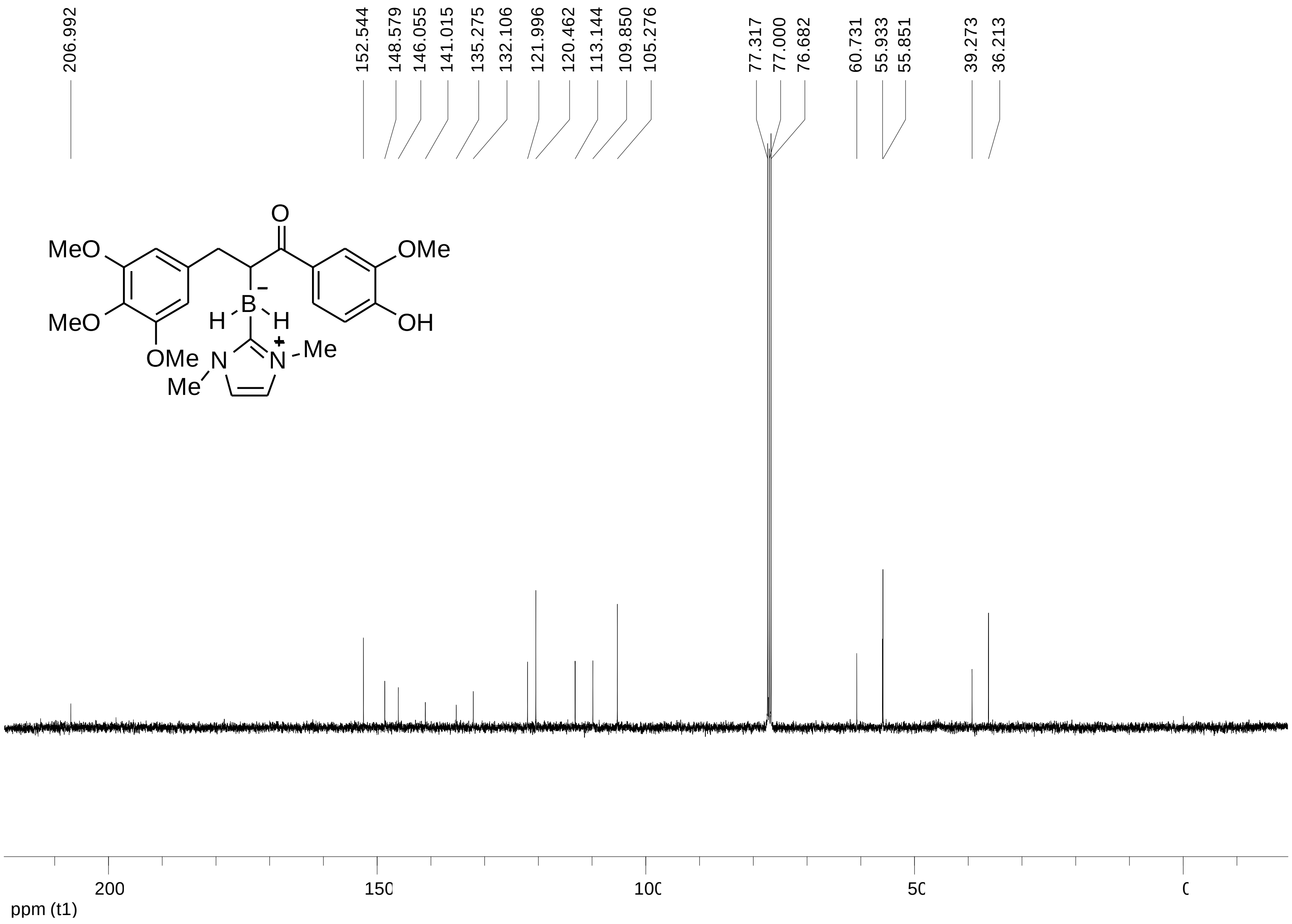

Supplementary Figure 151. ^{13}C NMR spectrum for 3ad

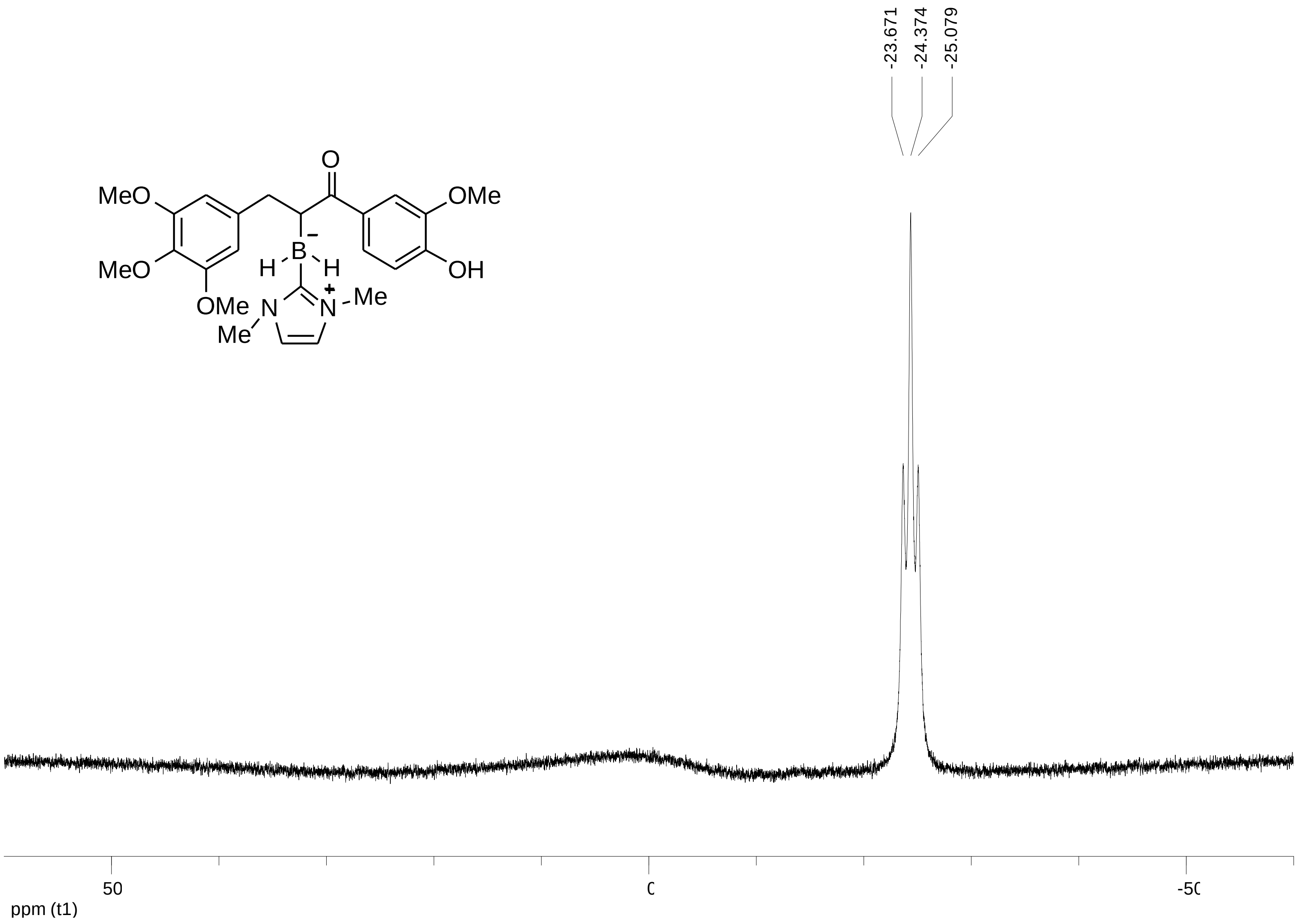

Supplementary Figure 152. ^{11}B NMR spectrum for 3ad

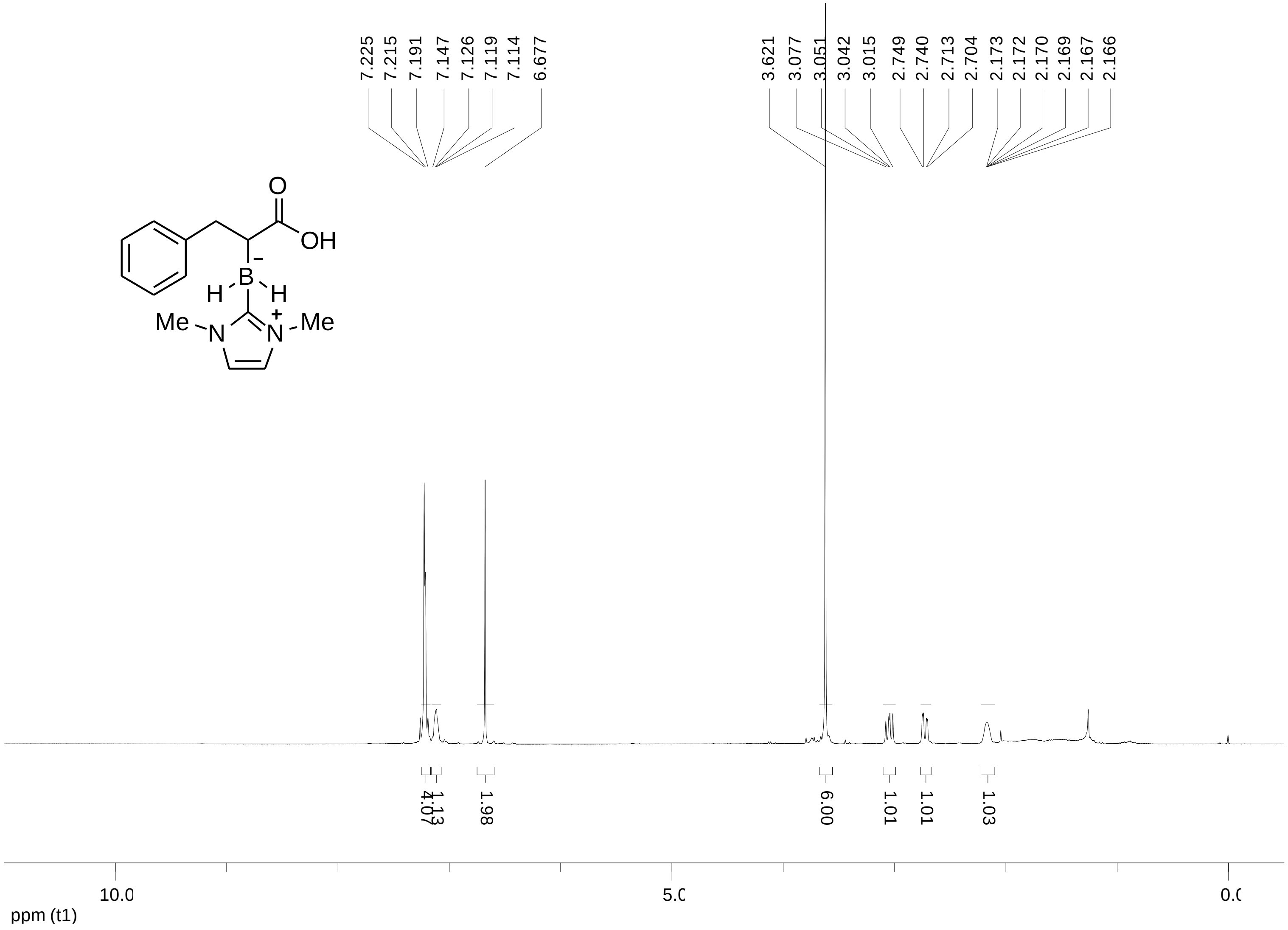

Supplementary Figure 153. ^{1}H NMR spectrum for 3ae

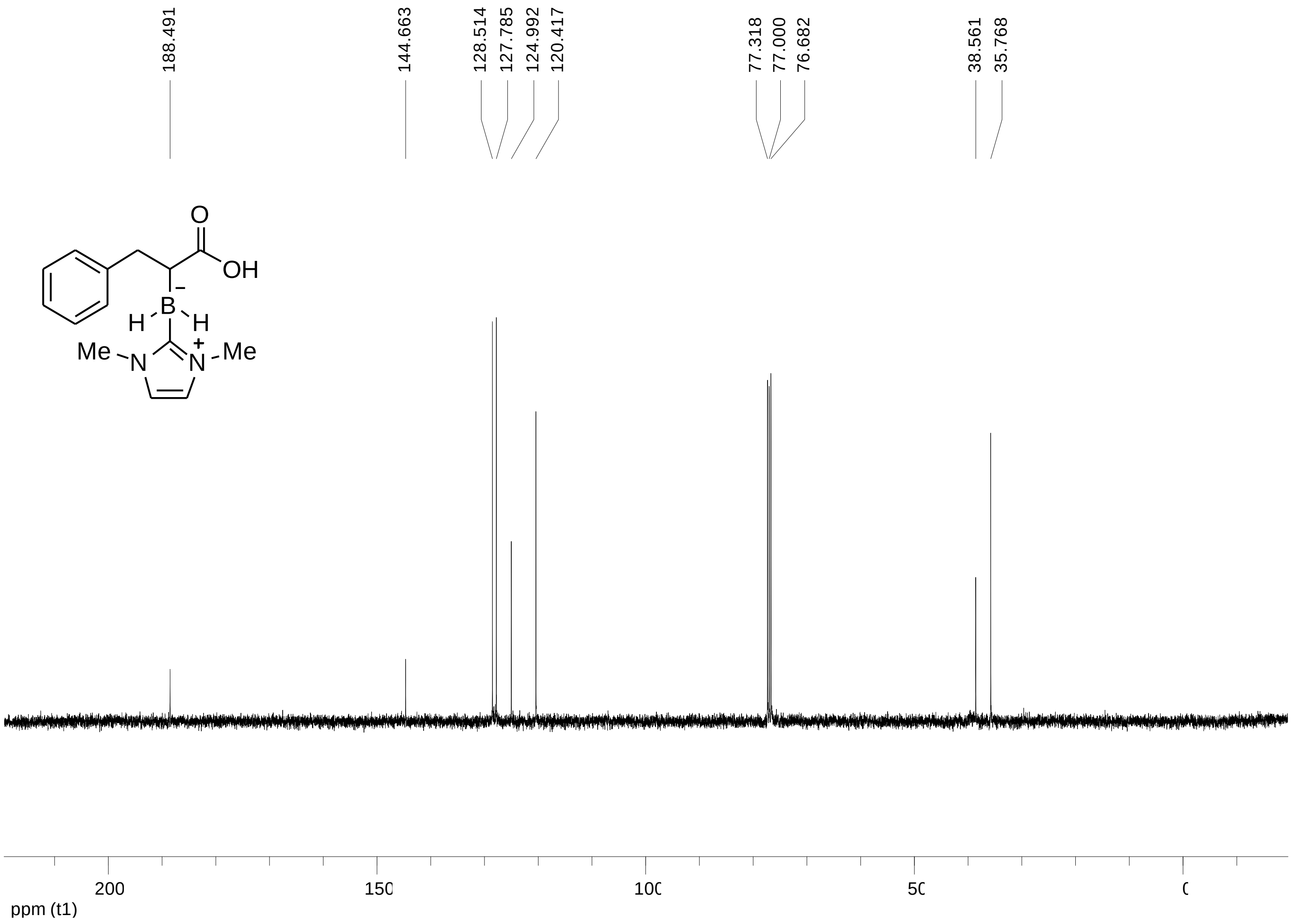

Supplementary Figure 154. ^{13}C NMR spectrum for 3ae

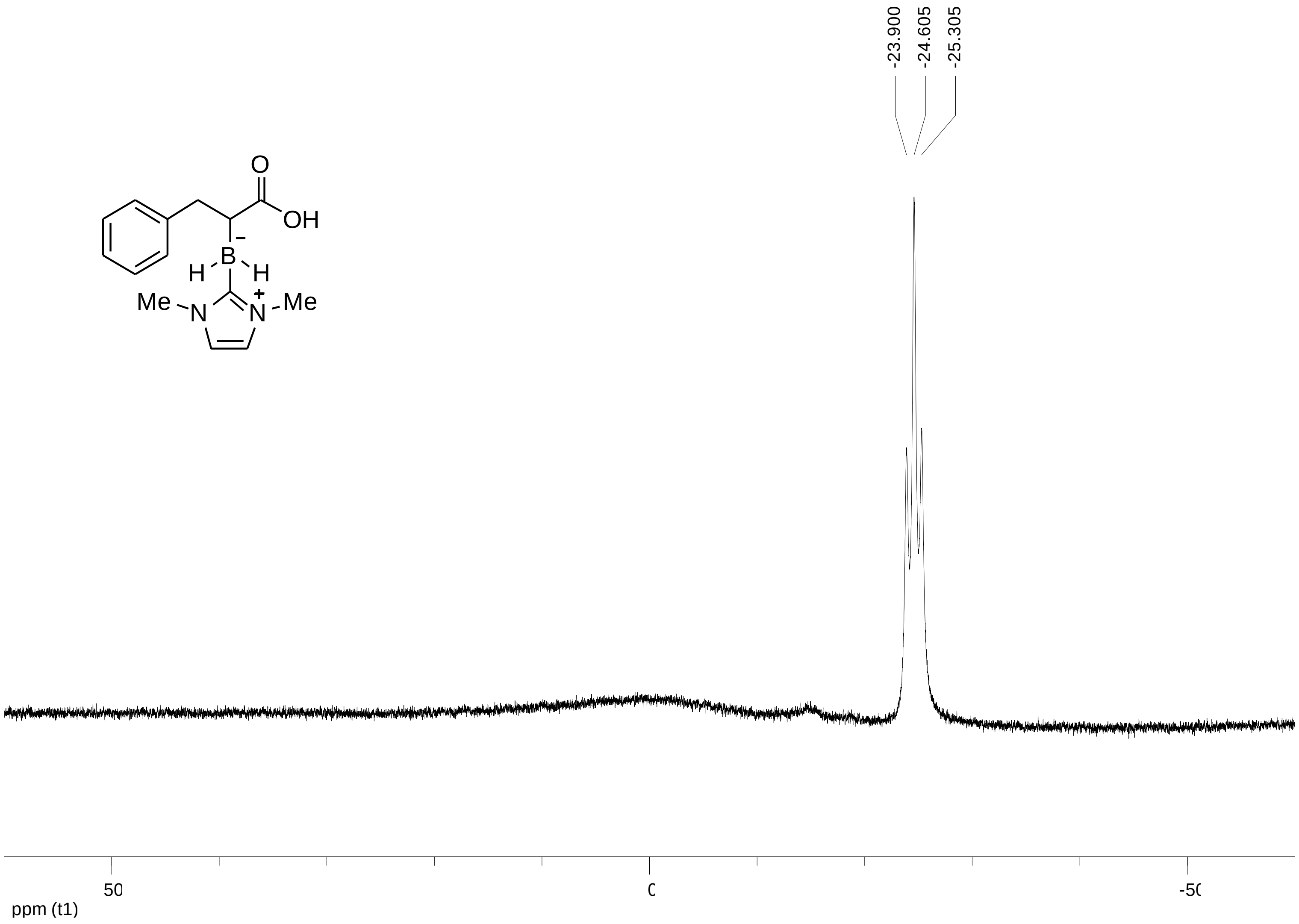

Supplementary Figure 155. ^{11}B NMR spectrum for 3ae

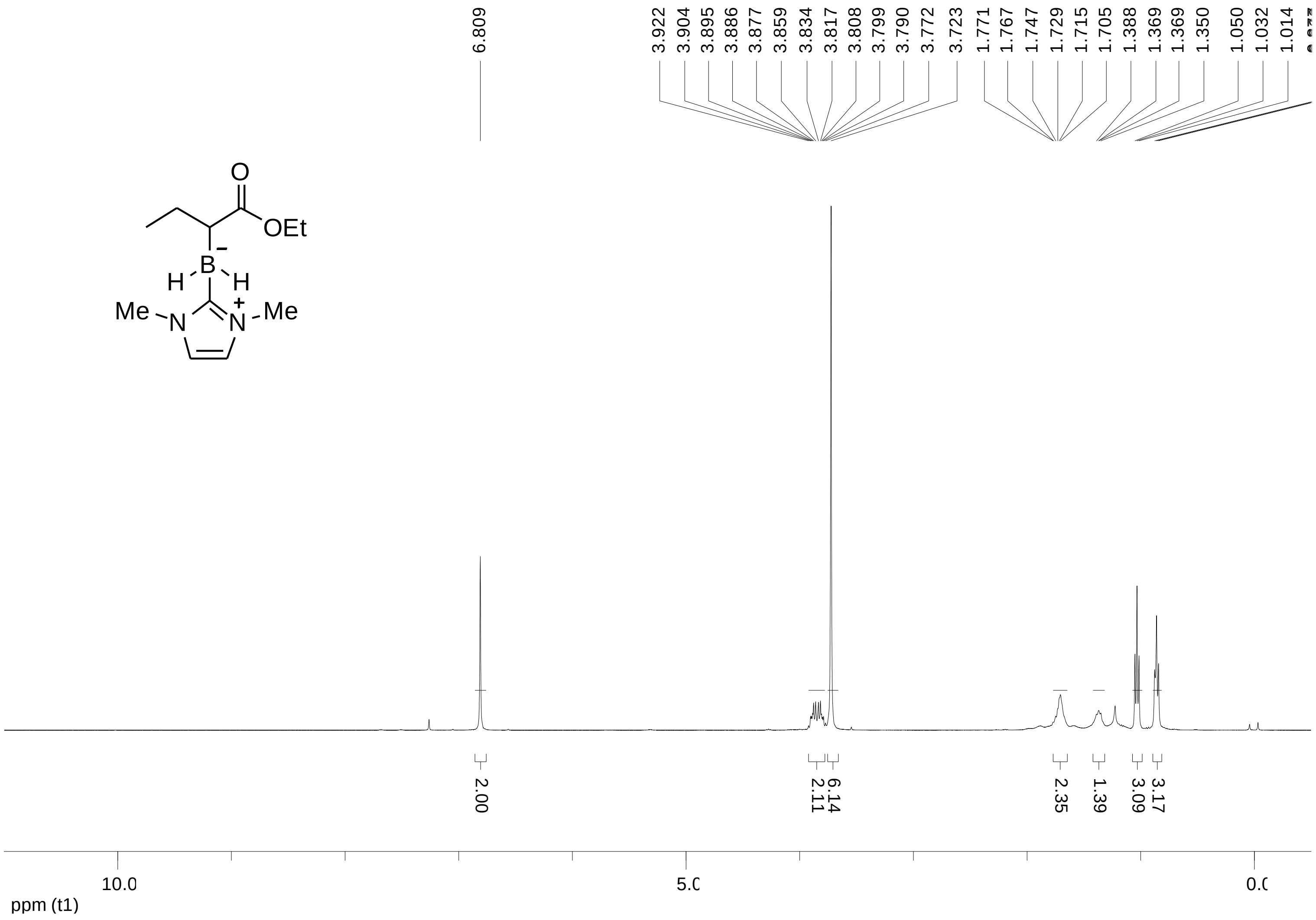

Supplementary Figure 156. ^{1}H NMR spectrum for 3f-α

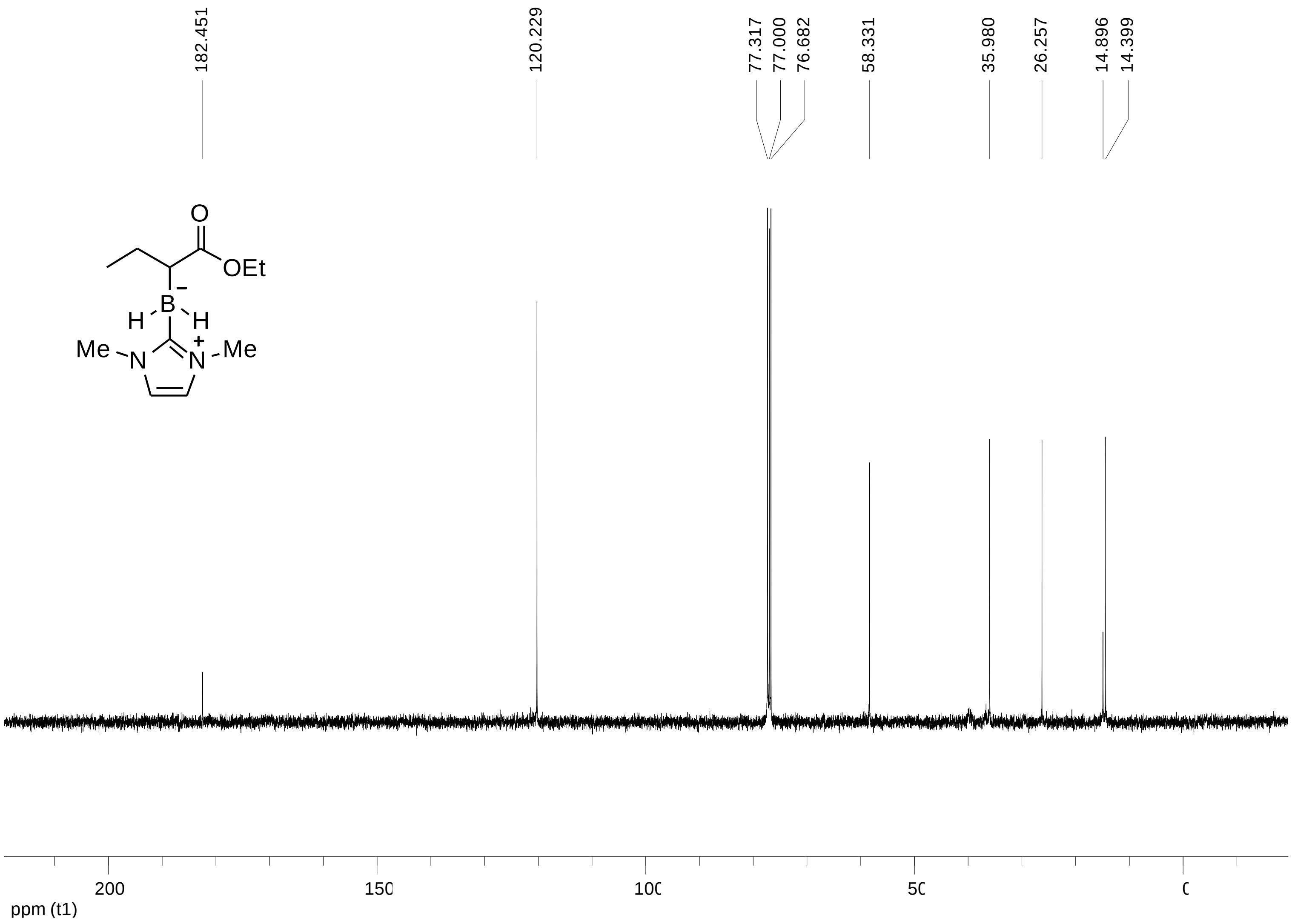

Supplementary Figure 157. ^{13}C NMR spectrum for 3af-α

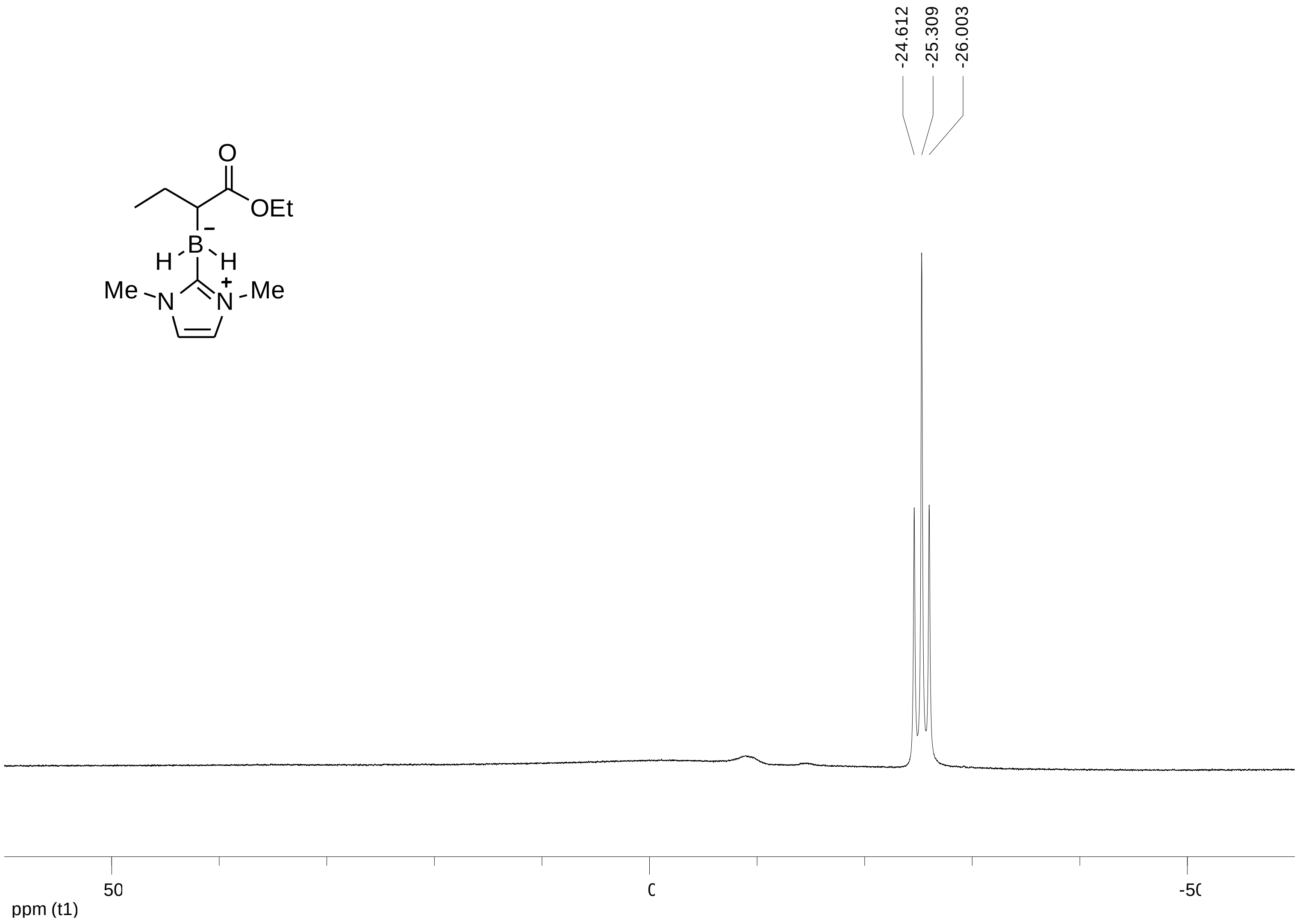

Supplementary Figure 158. ^{11}B NMR spectrum for 3af-α

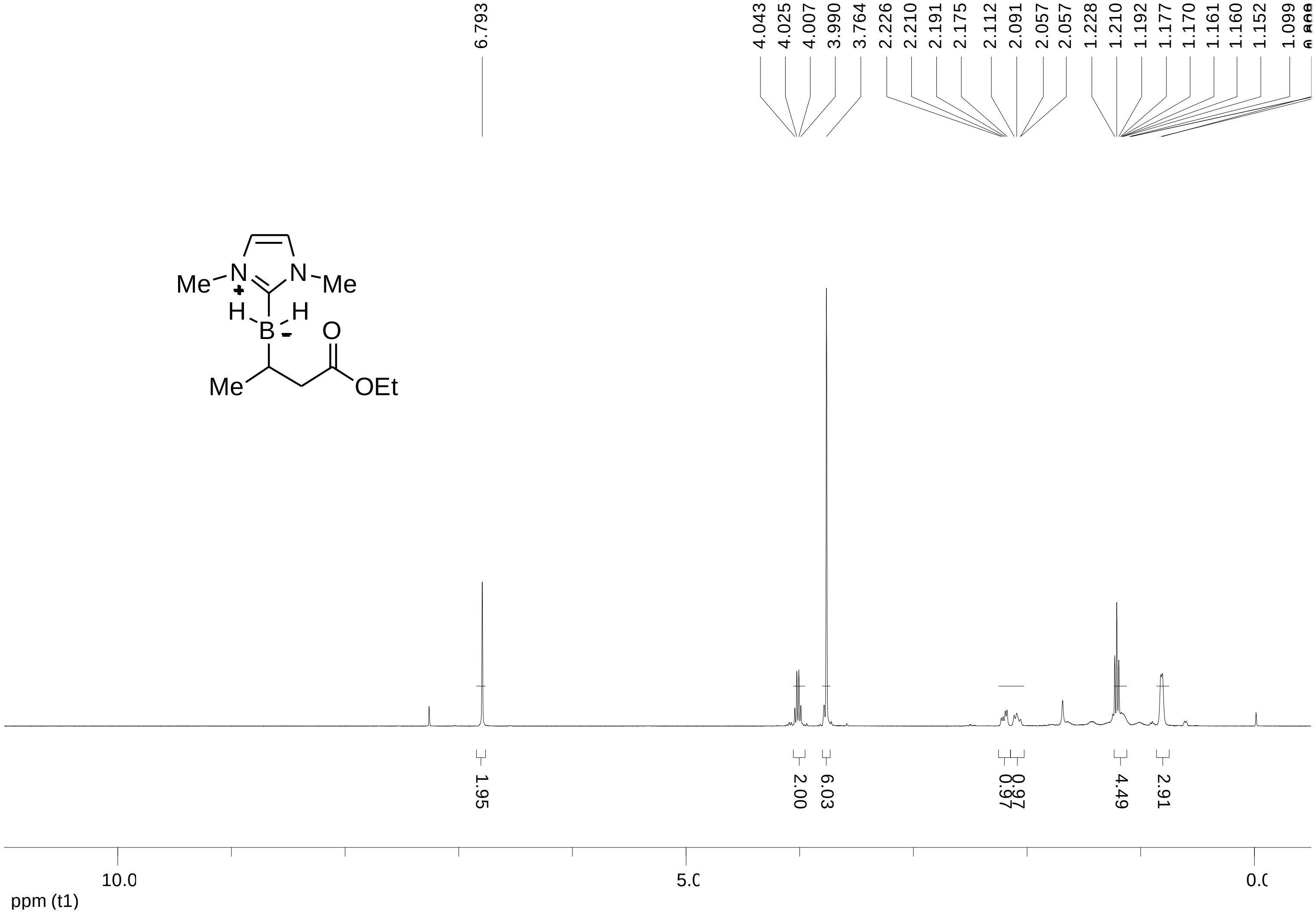

Supplementary Figure 159. ^{1}H NMR spectrum for 3af-β

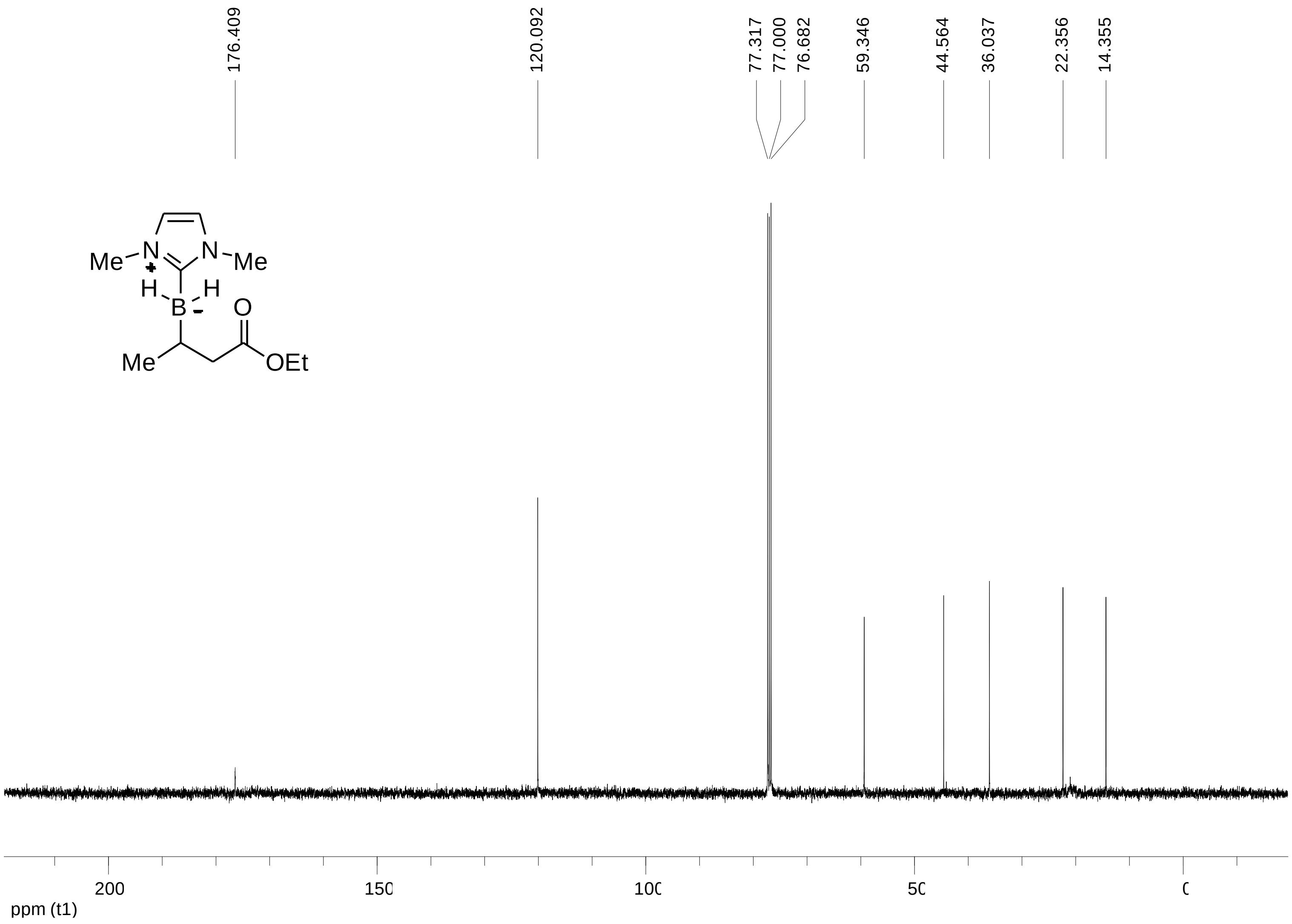

Supplementary Figure 160. ^{13}**C NMR spectrum for 3af-β**

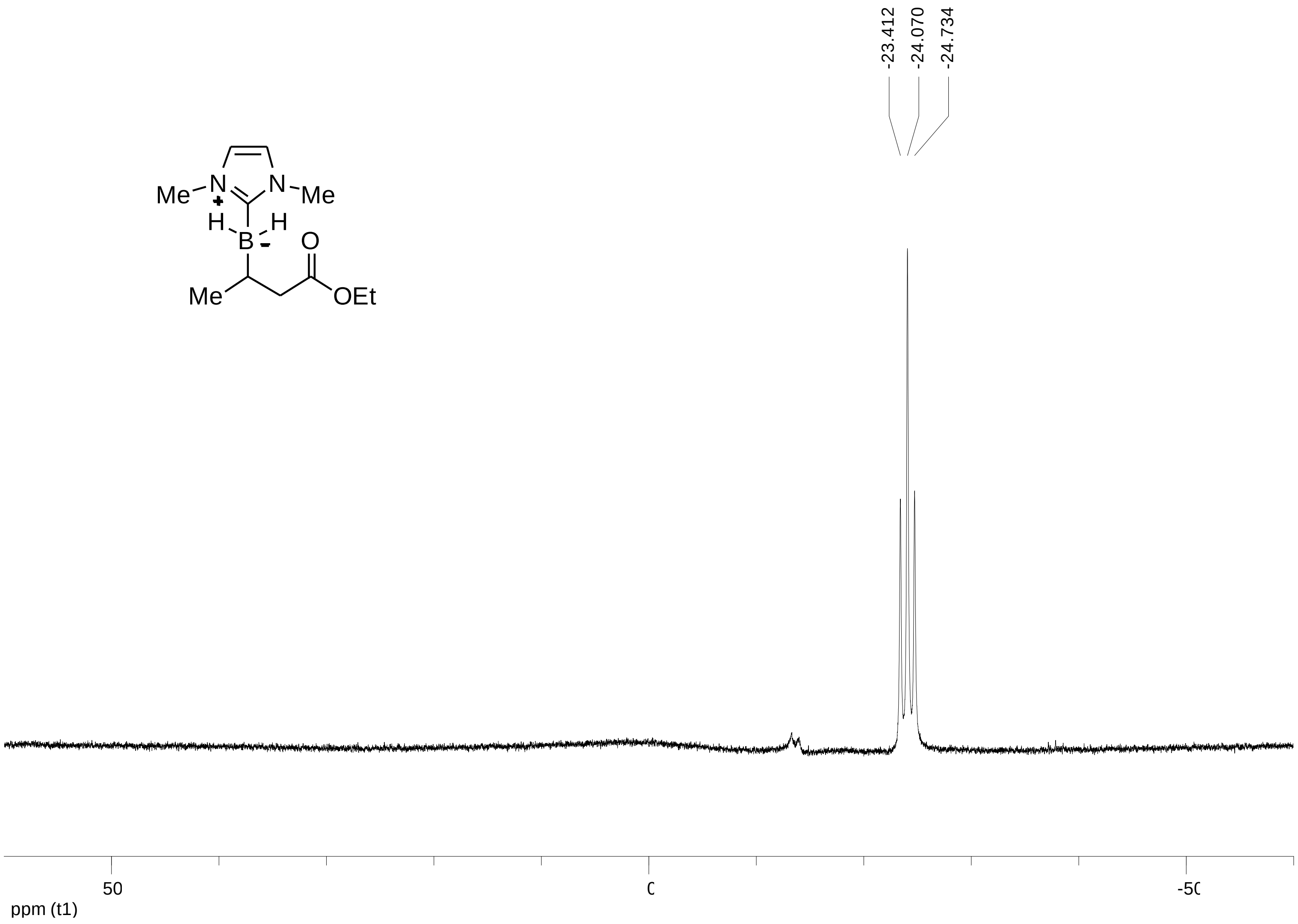

Supplementary Figure 161. ^{11}B NMR spectrum for 3af-β

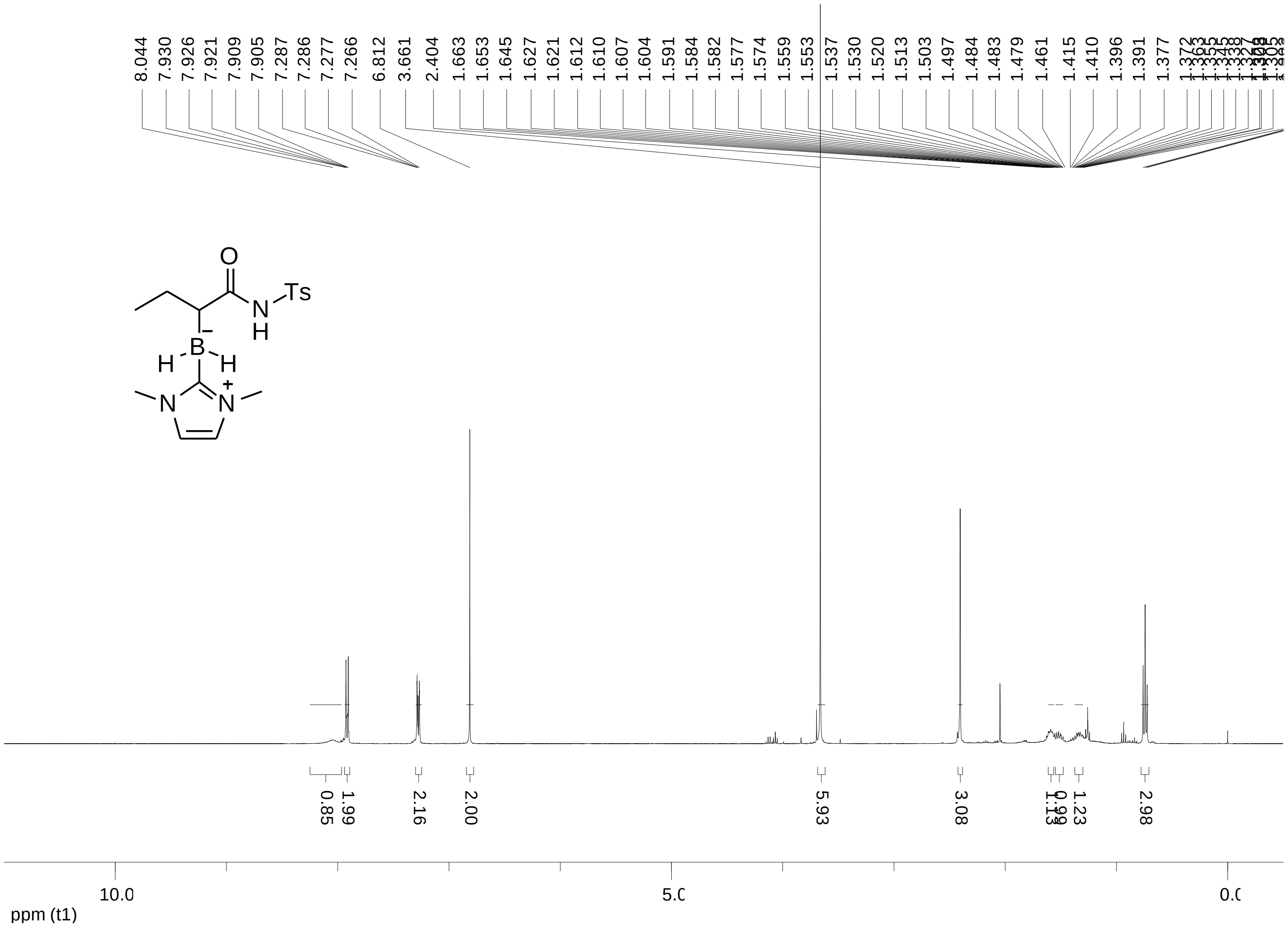

Supplementary Figure 162. ^{1}H NMR spectrum for 3ag-α

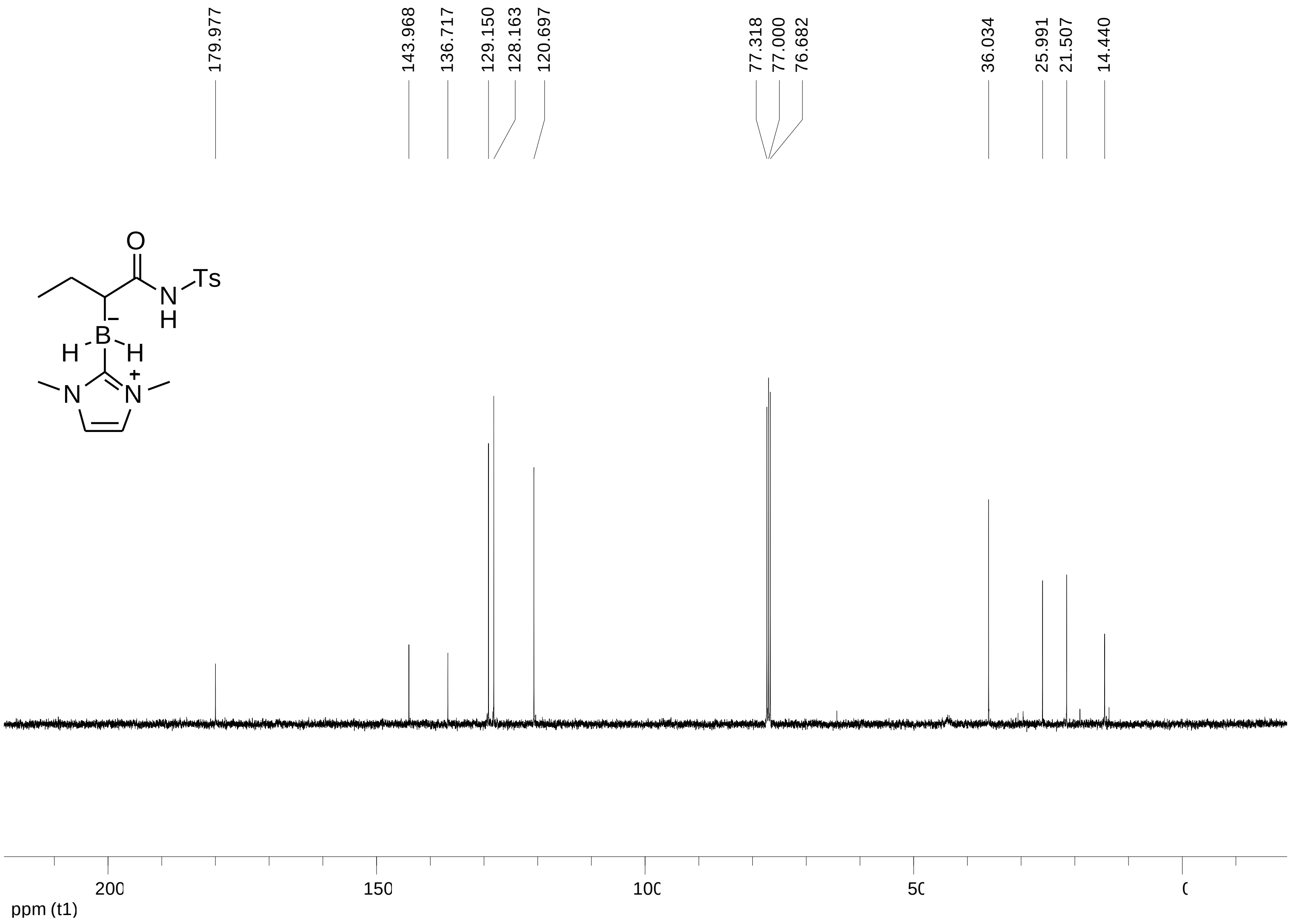

Supplementary Figure 163. ^{13}C NMR spectrum for 3ag-α

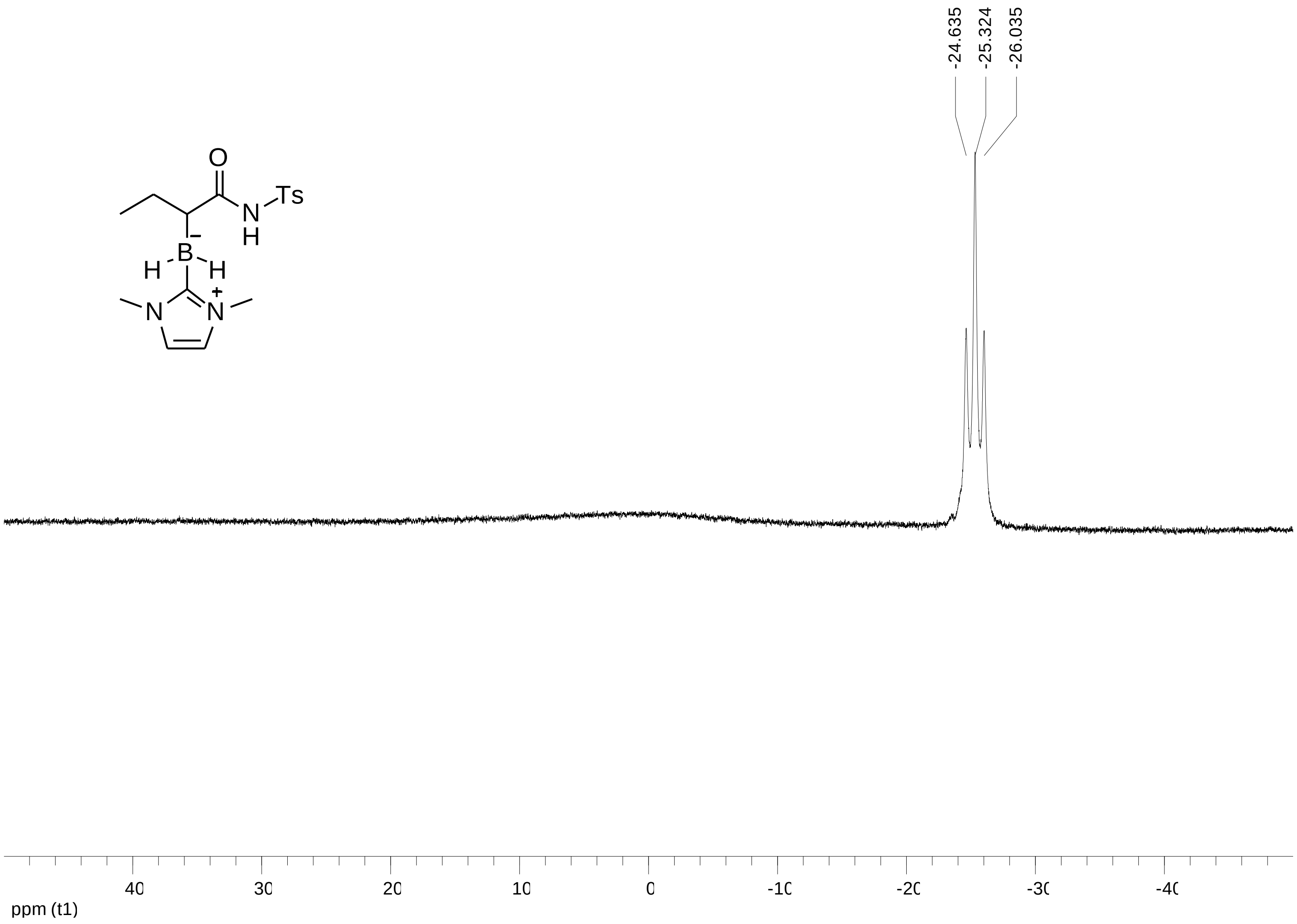

Supplementary Figure 164. ^{11}B NMR spectrum for 3ag-α

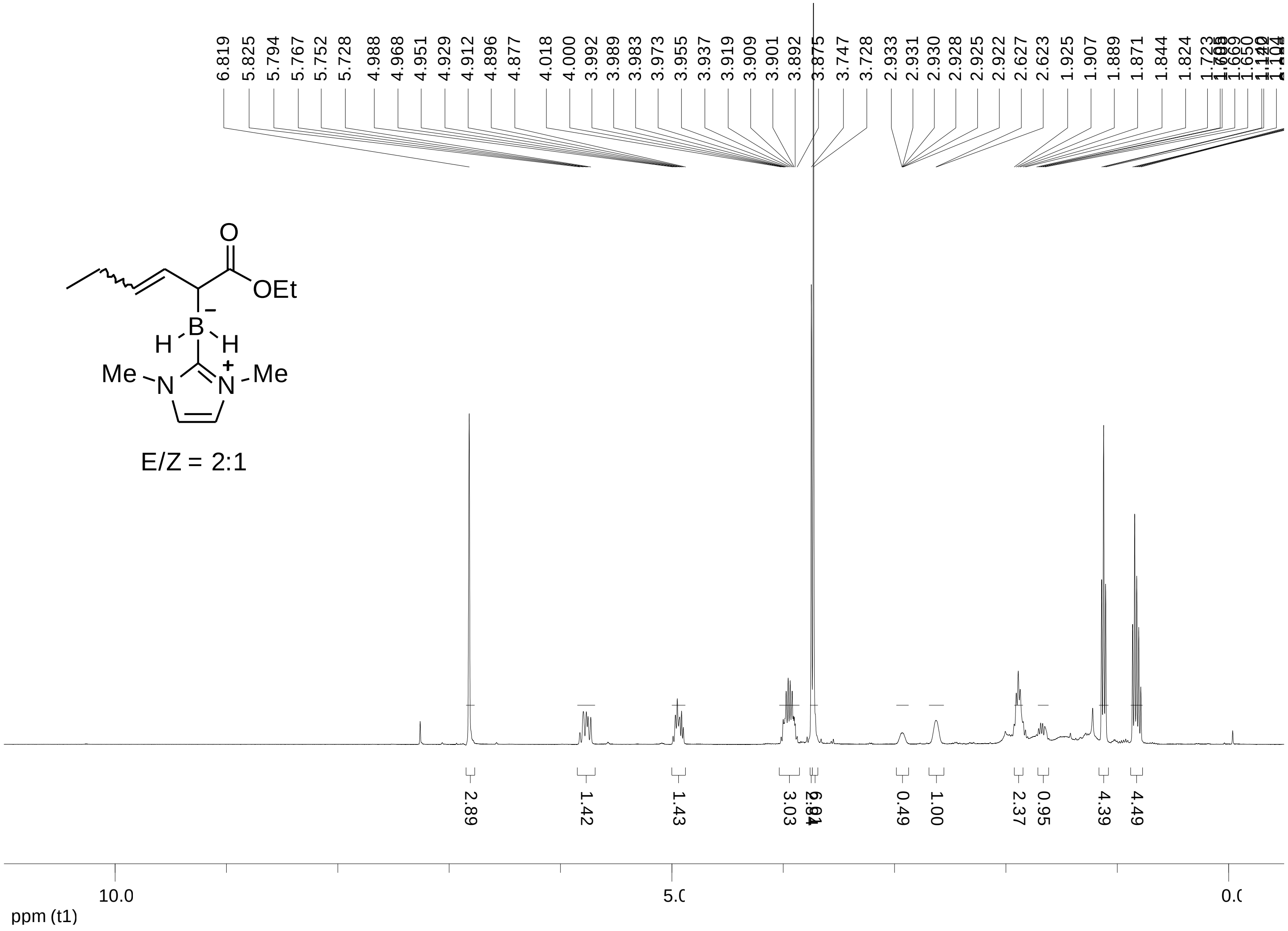

Supplementary Figure 165. ^{1}H NMR spectrum for 3ah

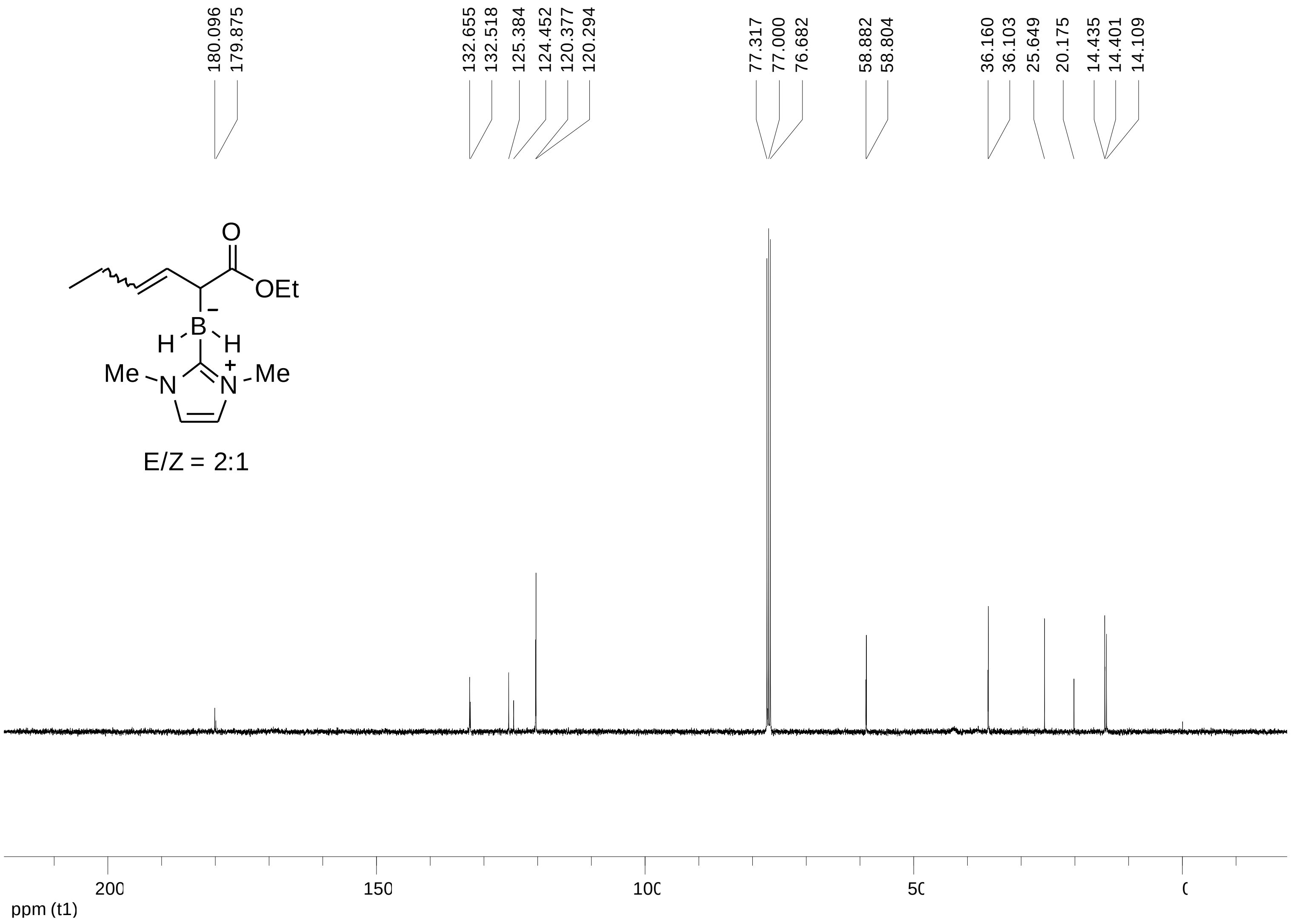

Supplementary Figure 166. ^{13}C NMR spectrum for 3ah

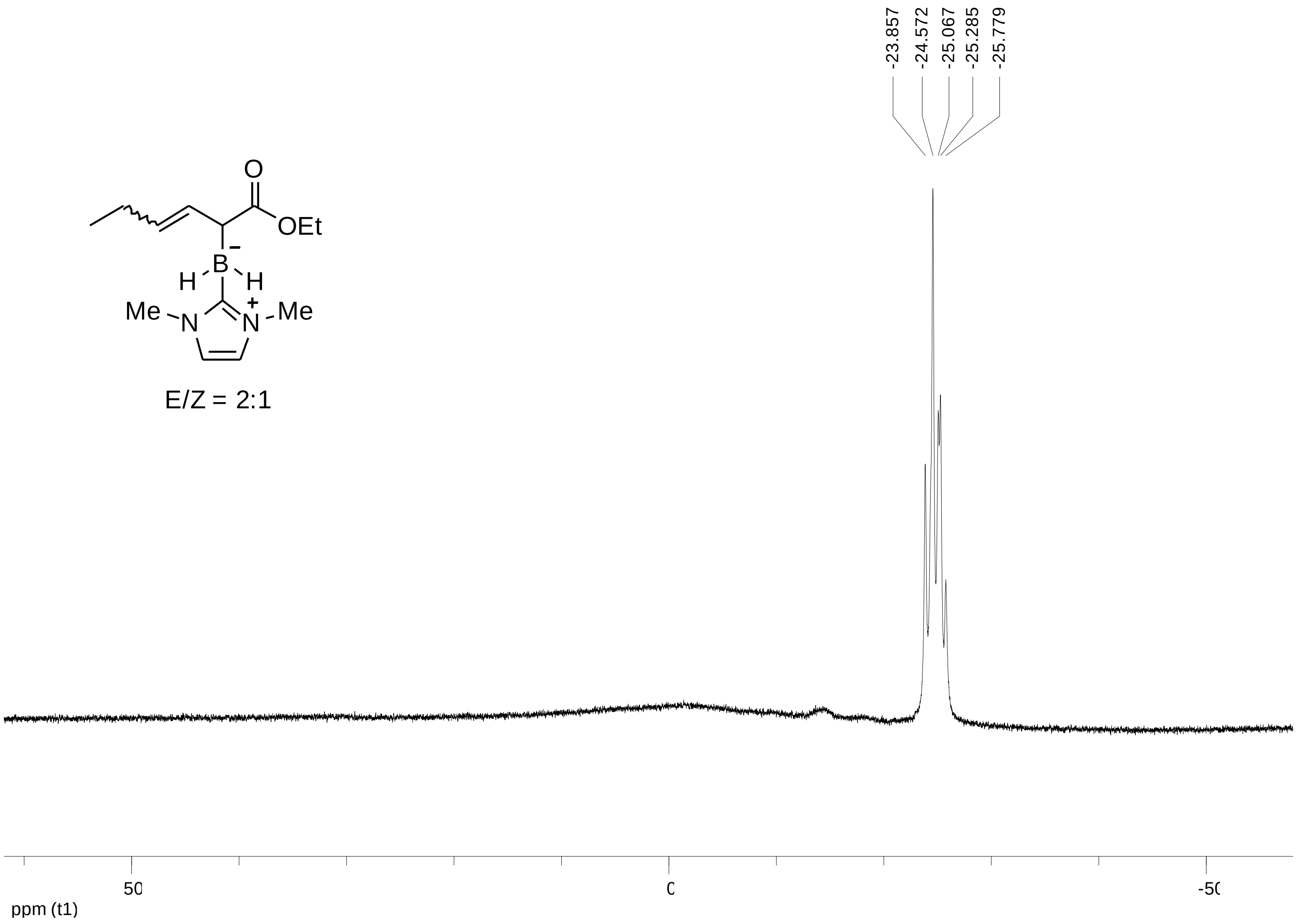

Supplementary Figure 167. ^{11}B NMR spectrum for 3ah

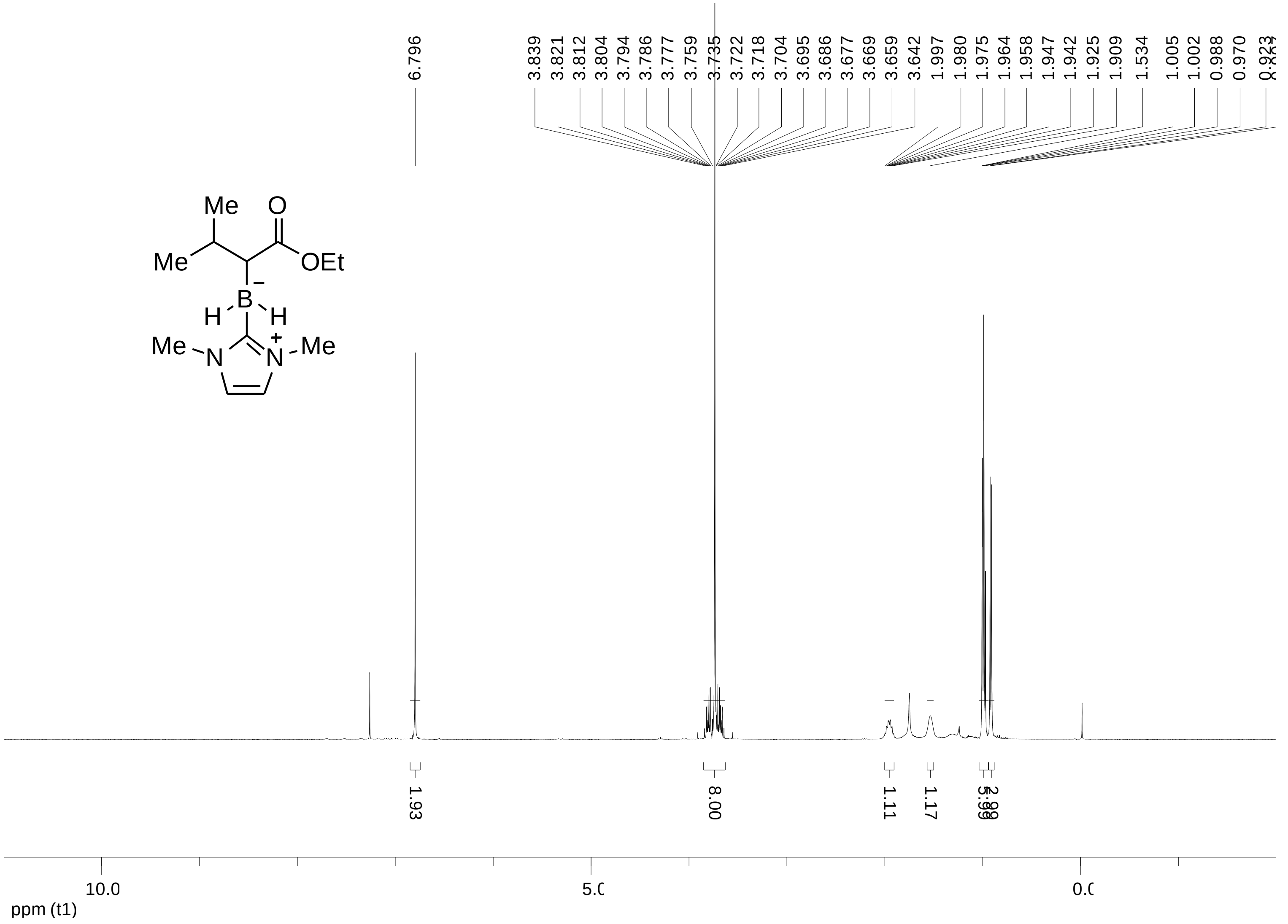

Supplementary Figure 168. ^{1}H NMR spectrum for 3ai

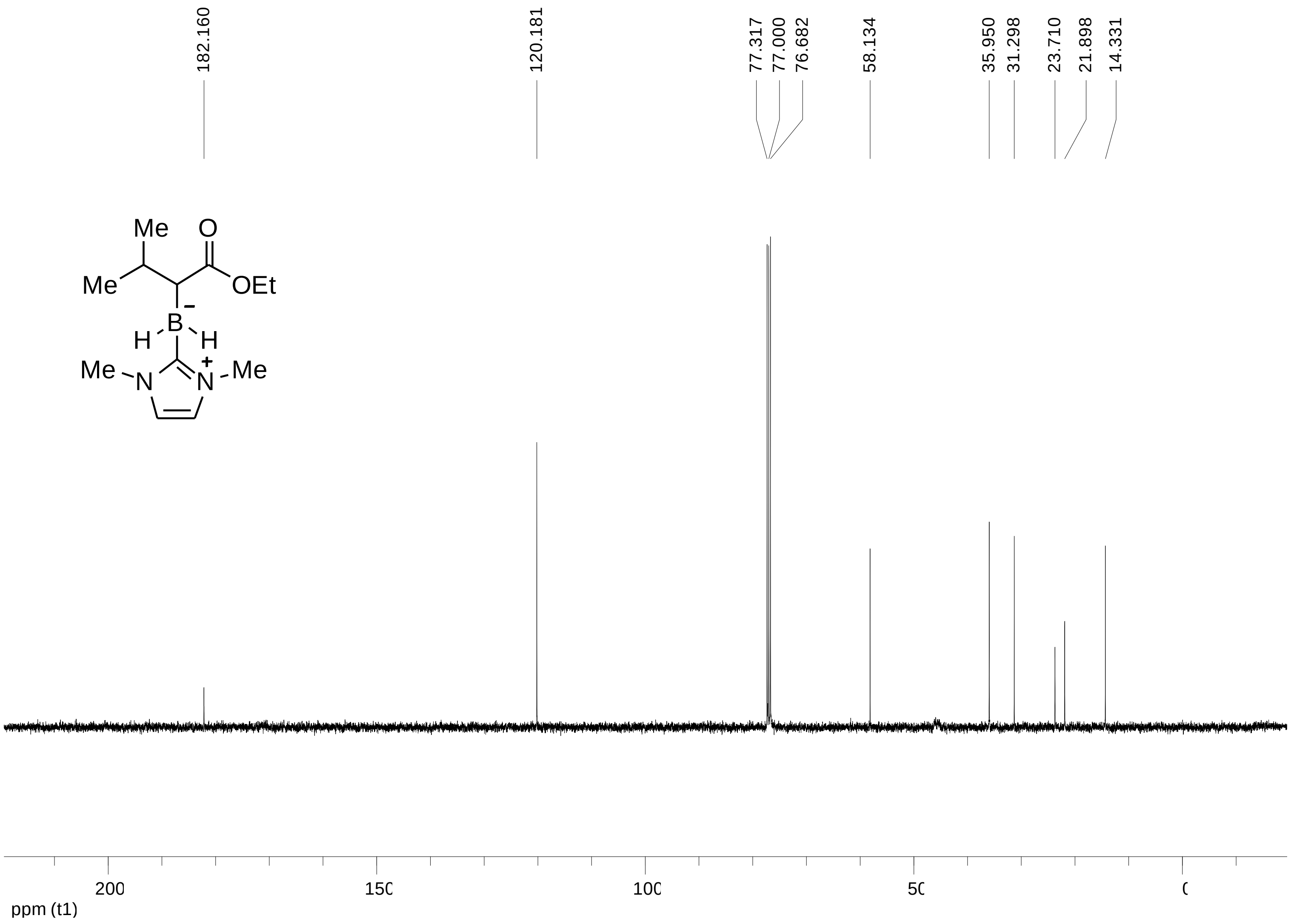

Supplementary Figure 169. ^{13}C NMR spectrum for 3ai

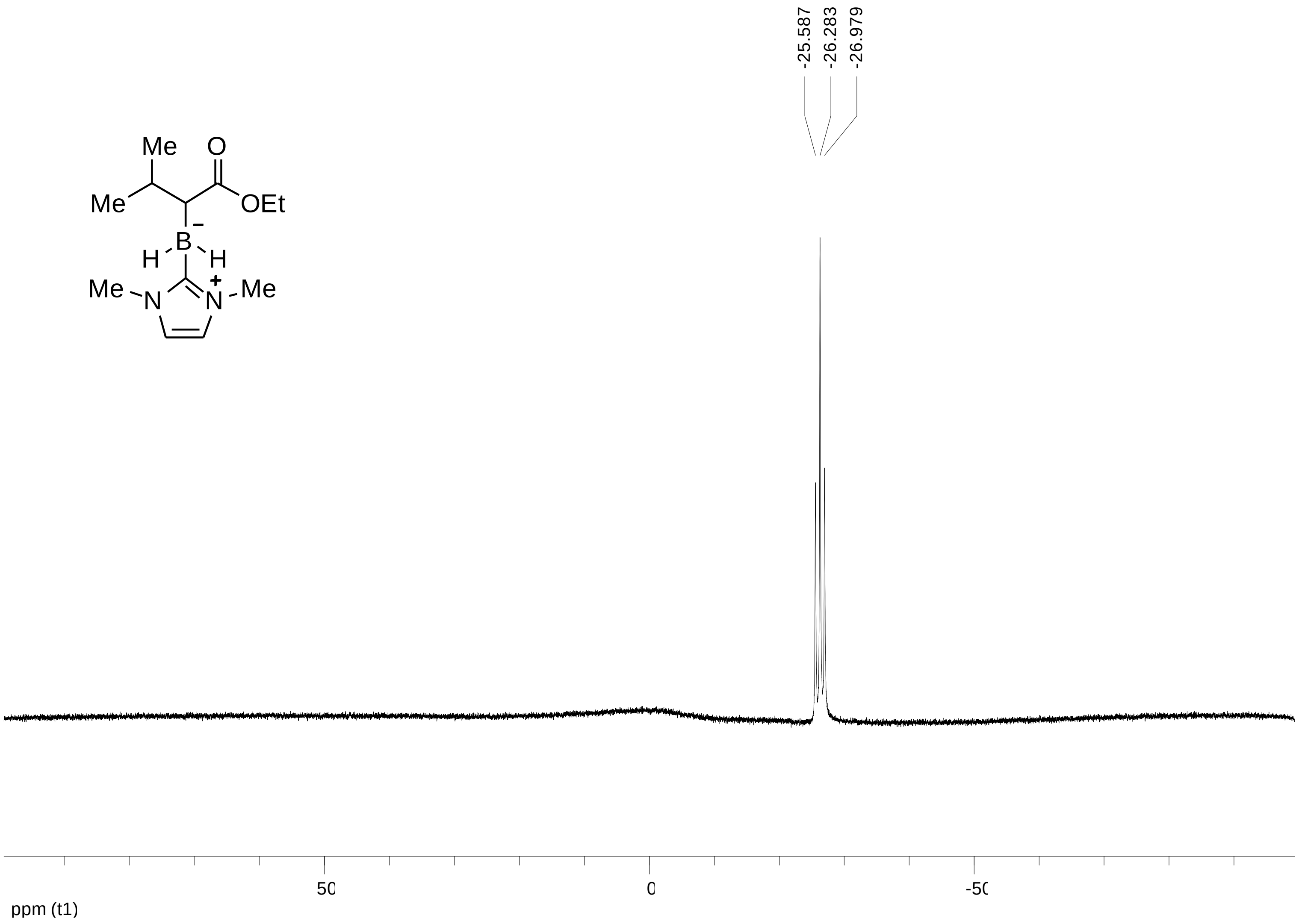

Supplementary Figure 170. ^{11}B NMR spectrum for 3ai

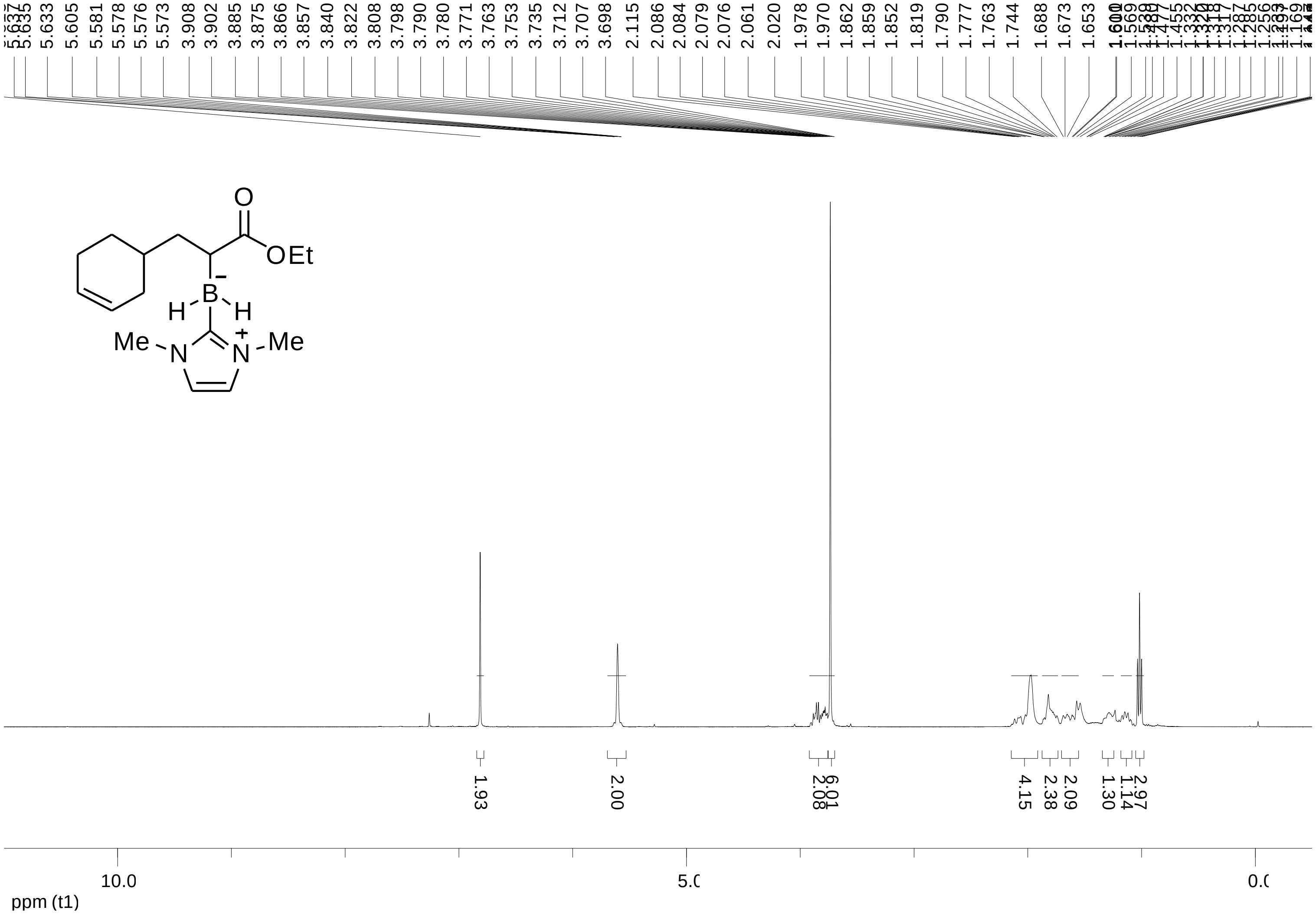

Supplementary Figure 171. ^{1}H NMR spectrum for 3aj

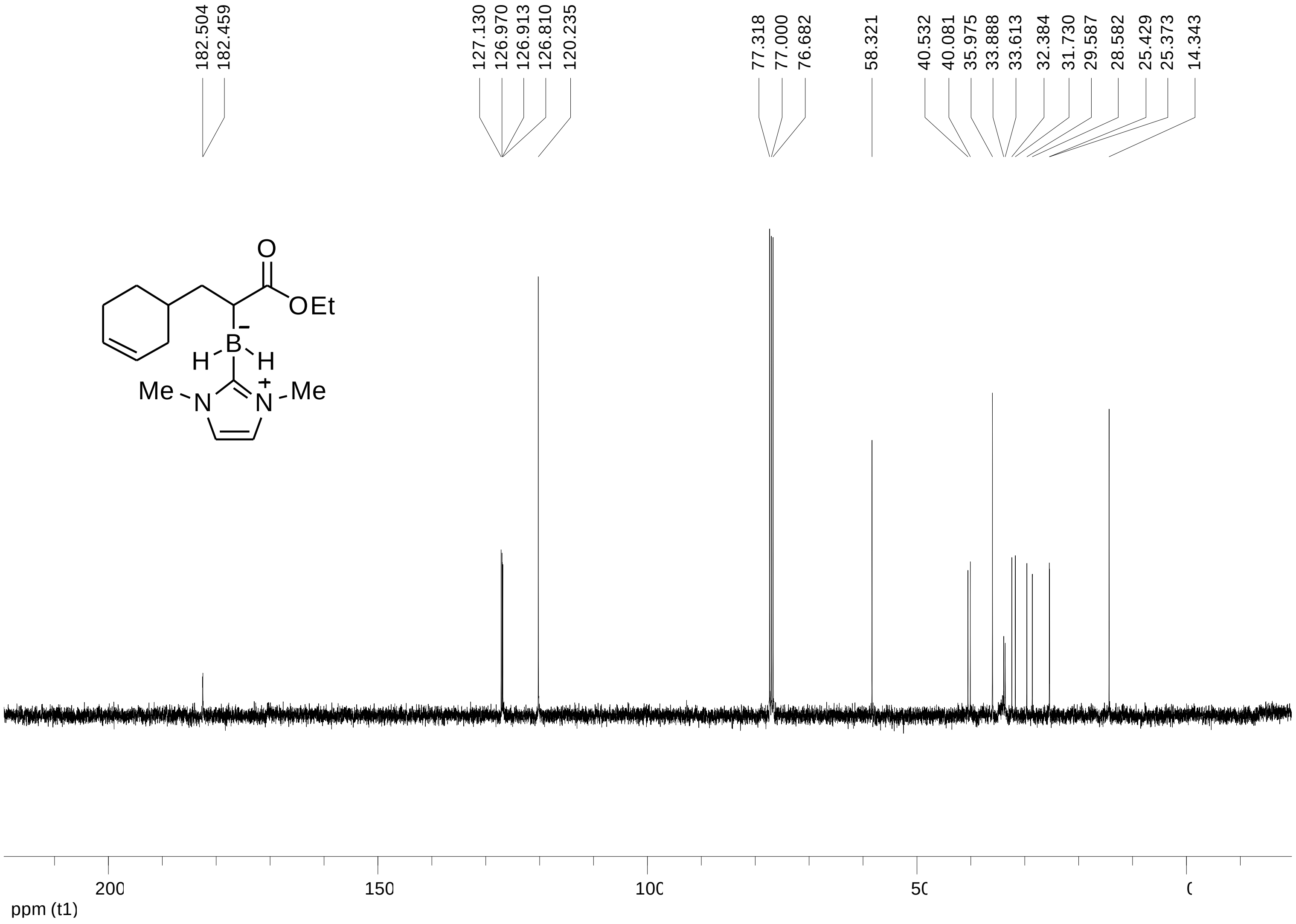

Supplementary Figure 172. ^{13}C NMR spectrum for 3aj

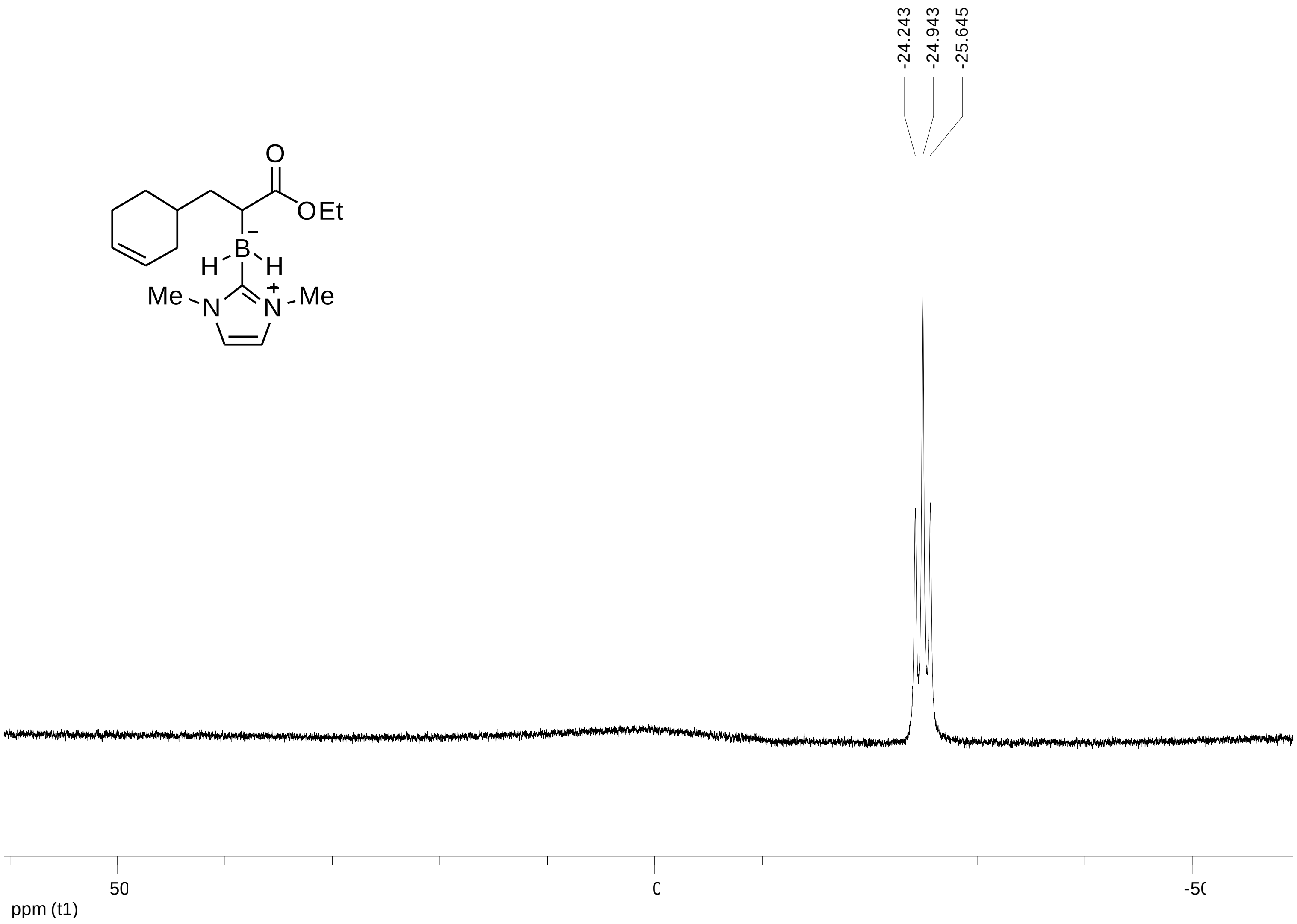

Supplementary Figure 173. ^{11}B NMR spectrum for 3aj

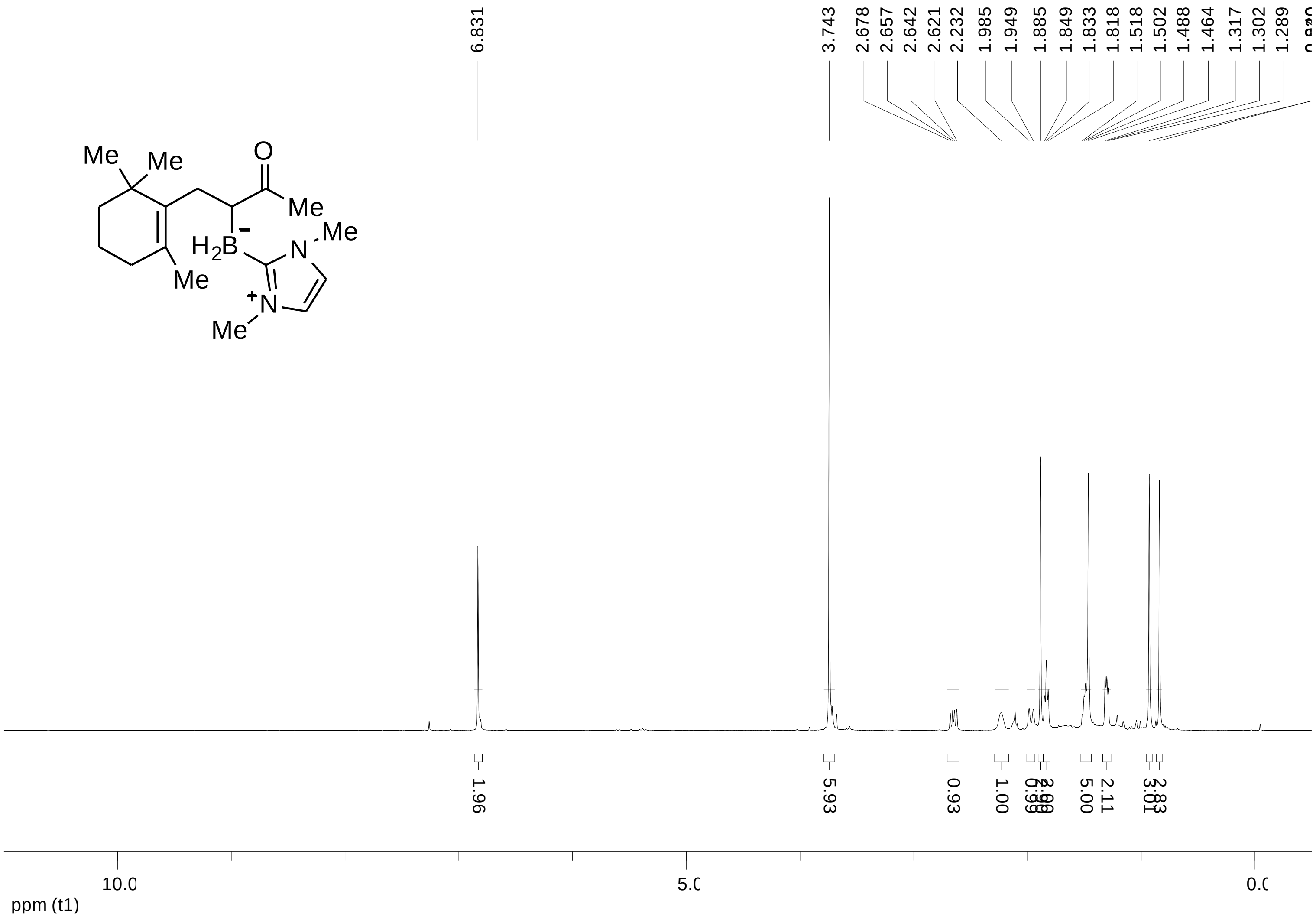

Supplementary Figure 174. ^{1}H NMR spectrum for 3ak

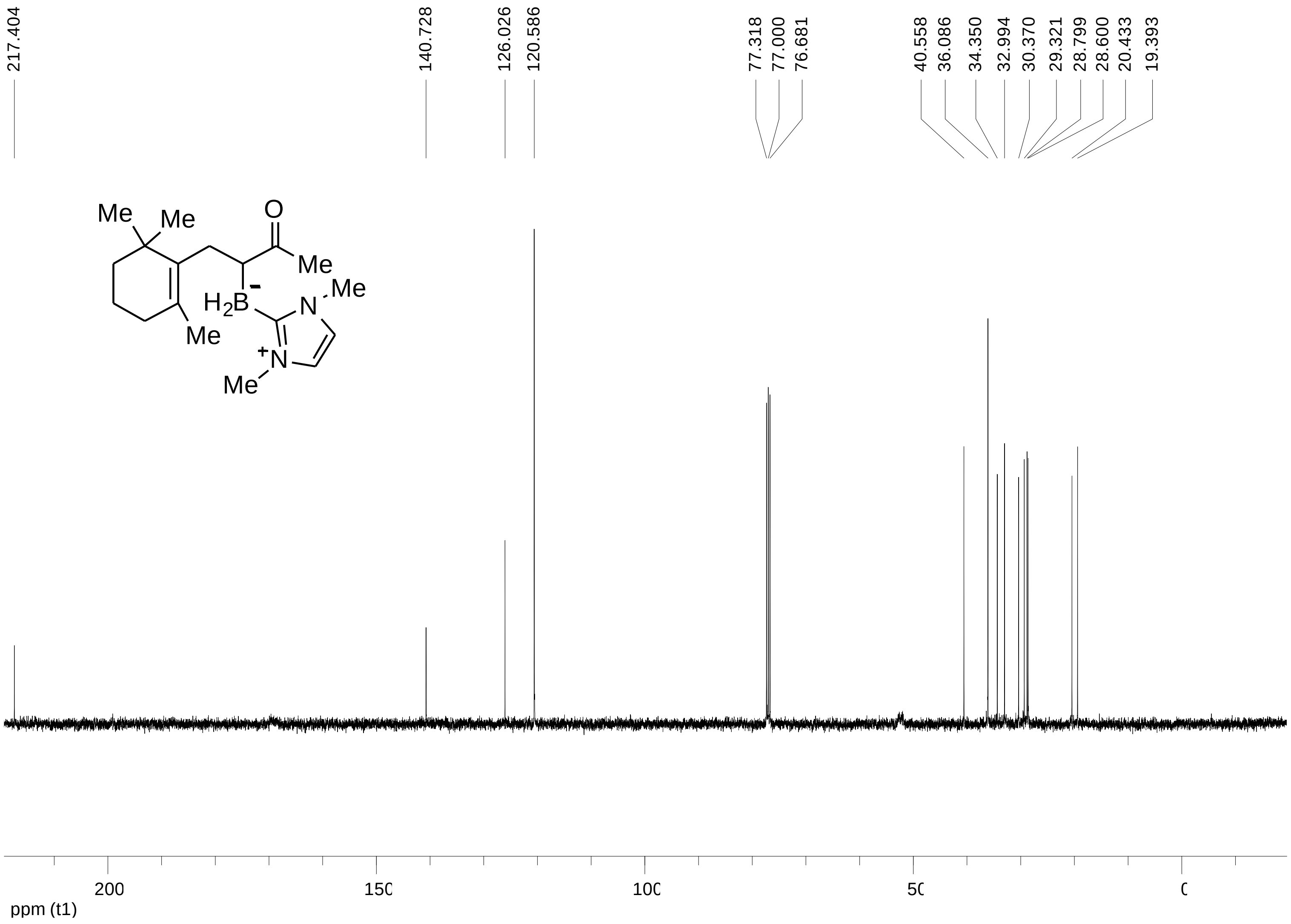

Supplementary Figure 175. ^{13}C NMR spectrum for 3ak

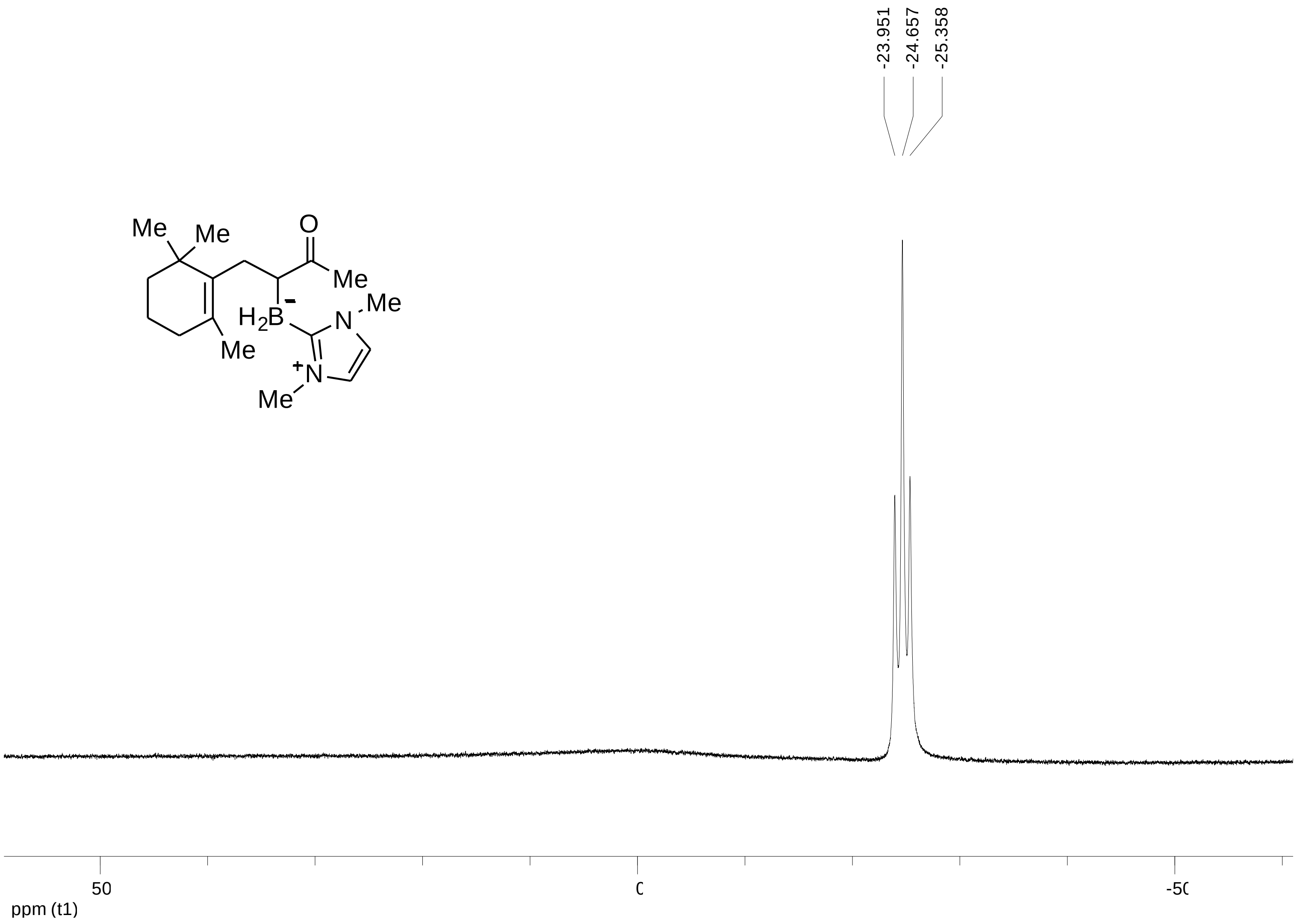

Supplementary Figure 176. ^{11}B NMR spectrum for 3ak

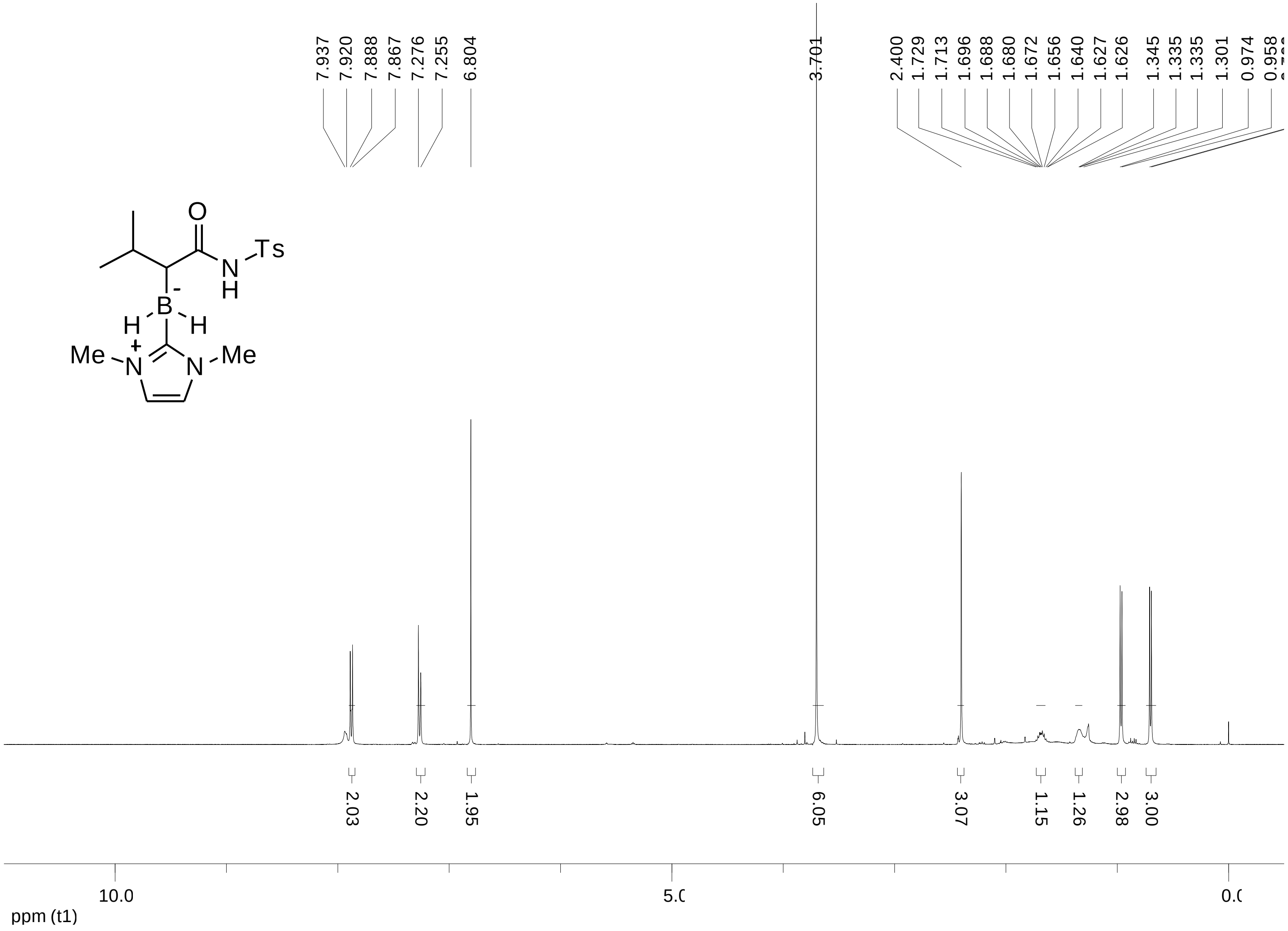

Supplementary Figure 177. ^{1}H NMR spectrum for 3al

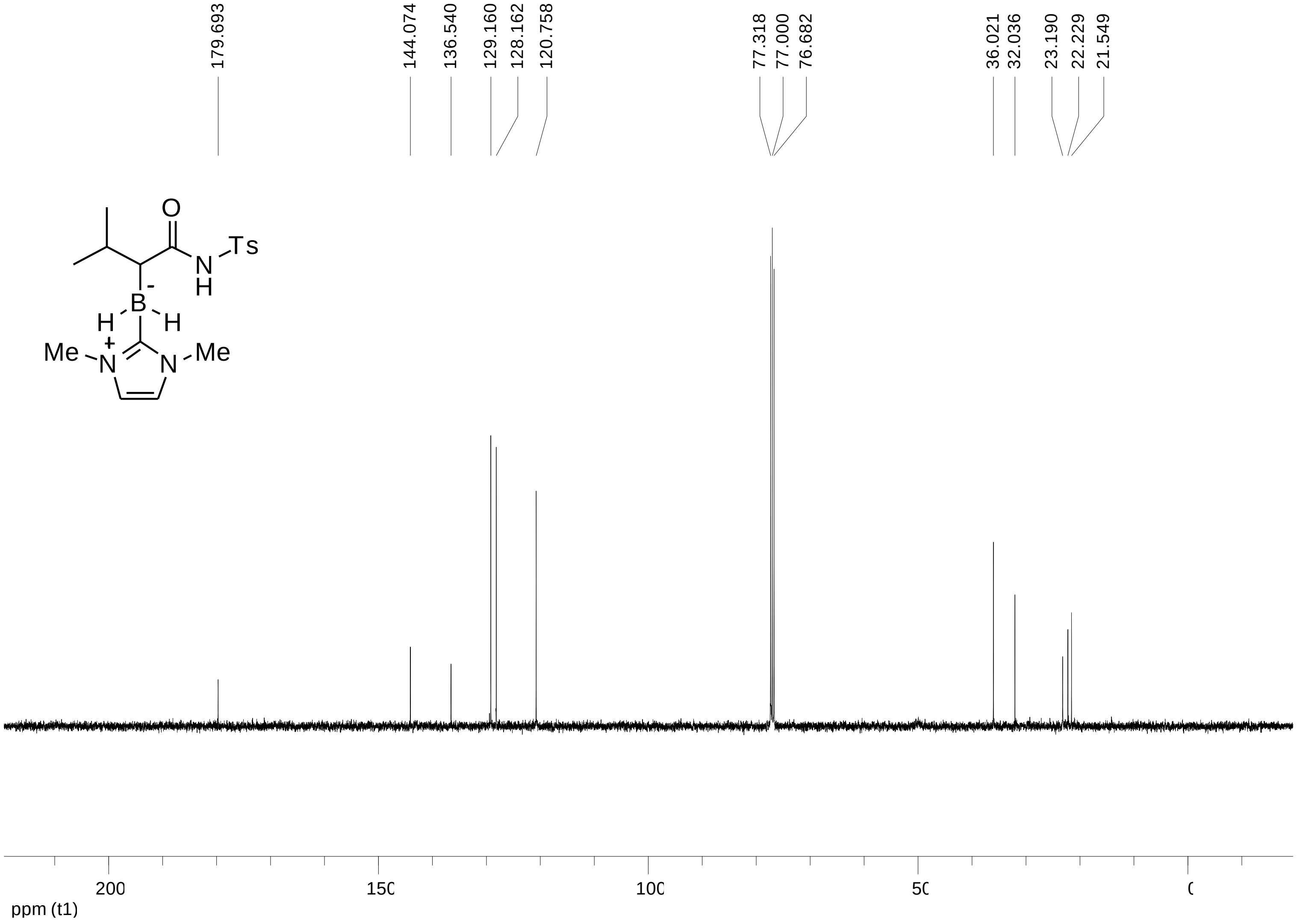

Supplementary Figure 178. ^{13}C NMR spectrum for 3al

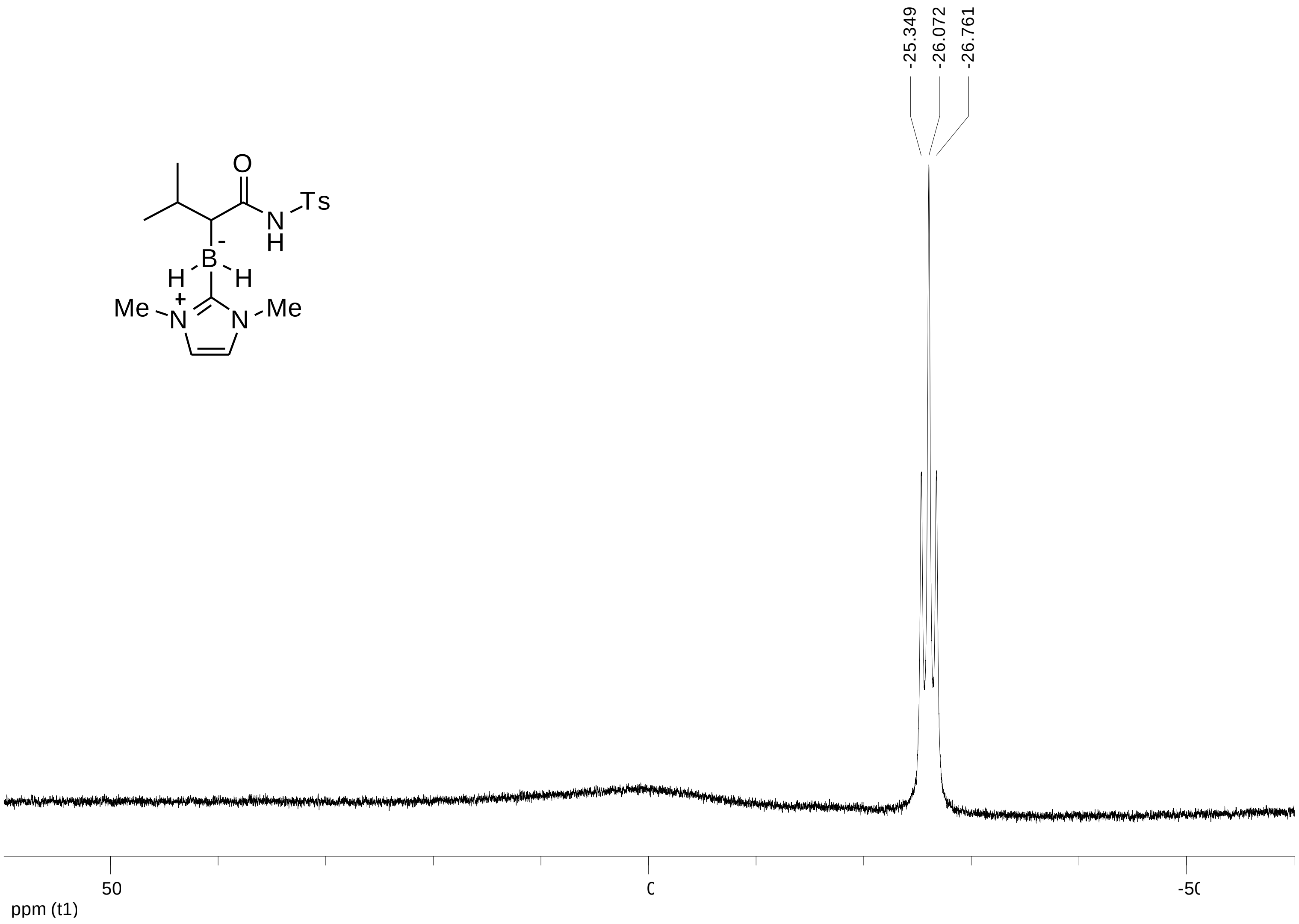

Supplementary Figure 179. ^{11}B NMR spectrum for 3al

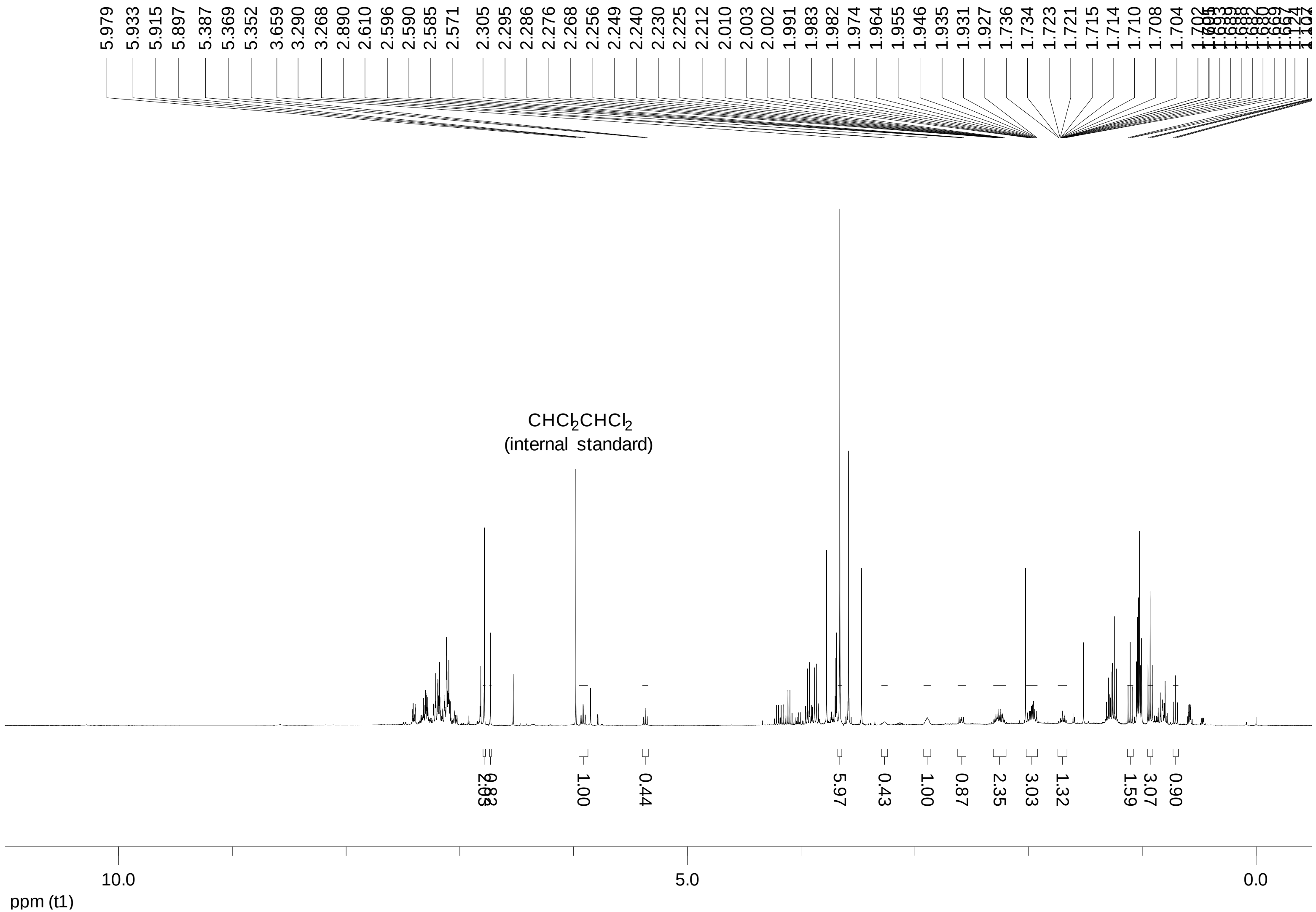

Supplementary Figure 180. ^{1}H NMR spectrum for crude 3am

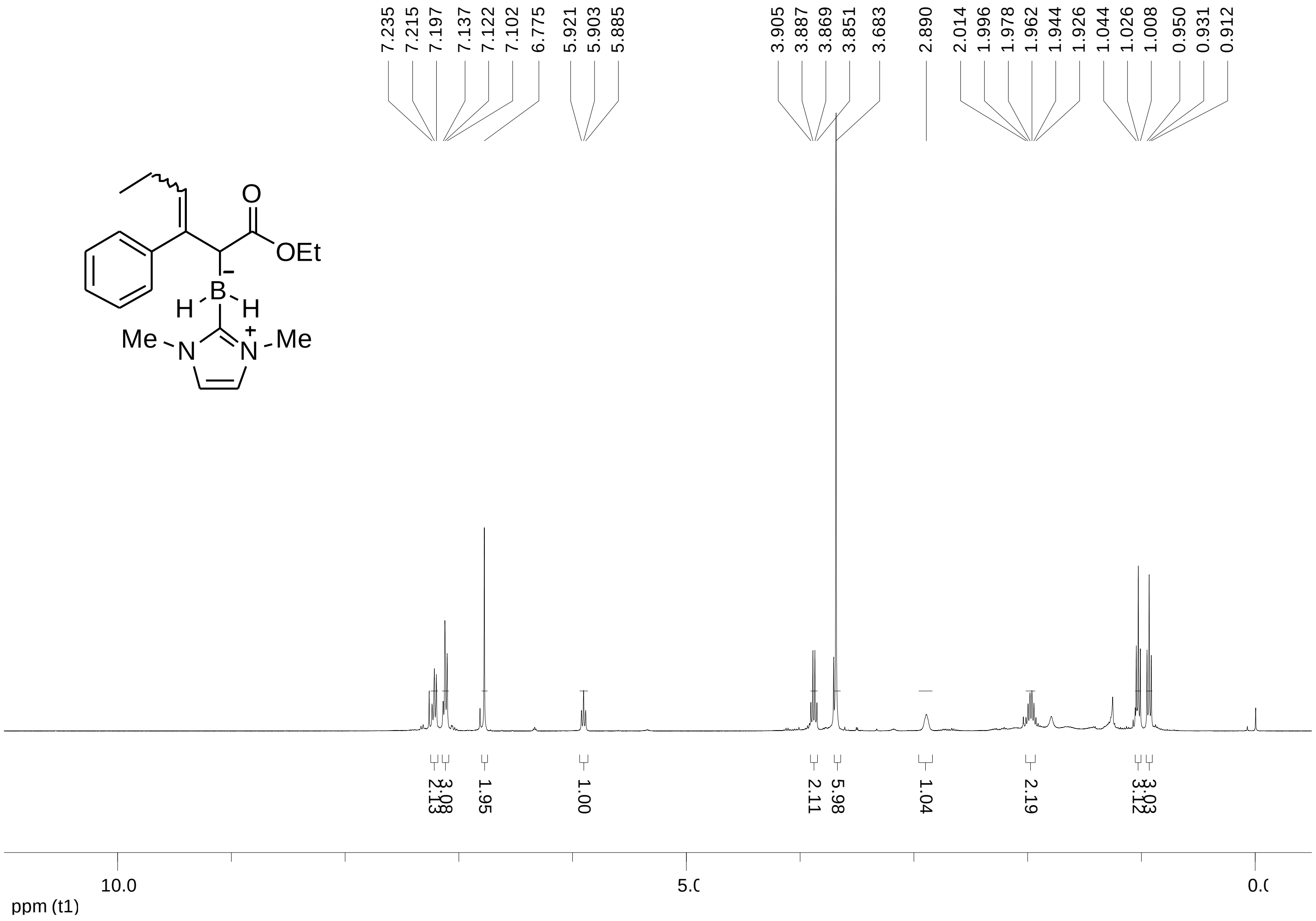

Supplementary Figure 181. ^{1}H NMR spectrum for 3am(one of the isomers)

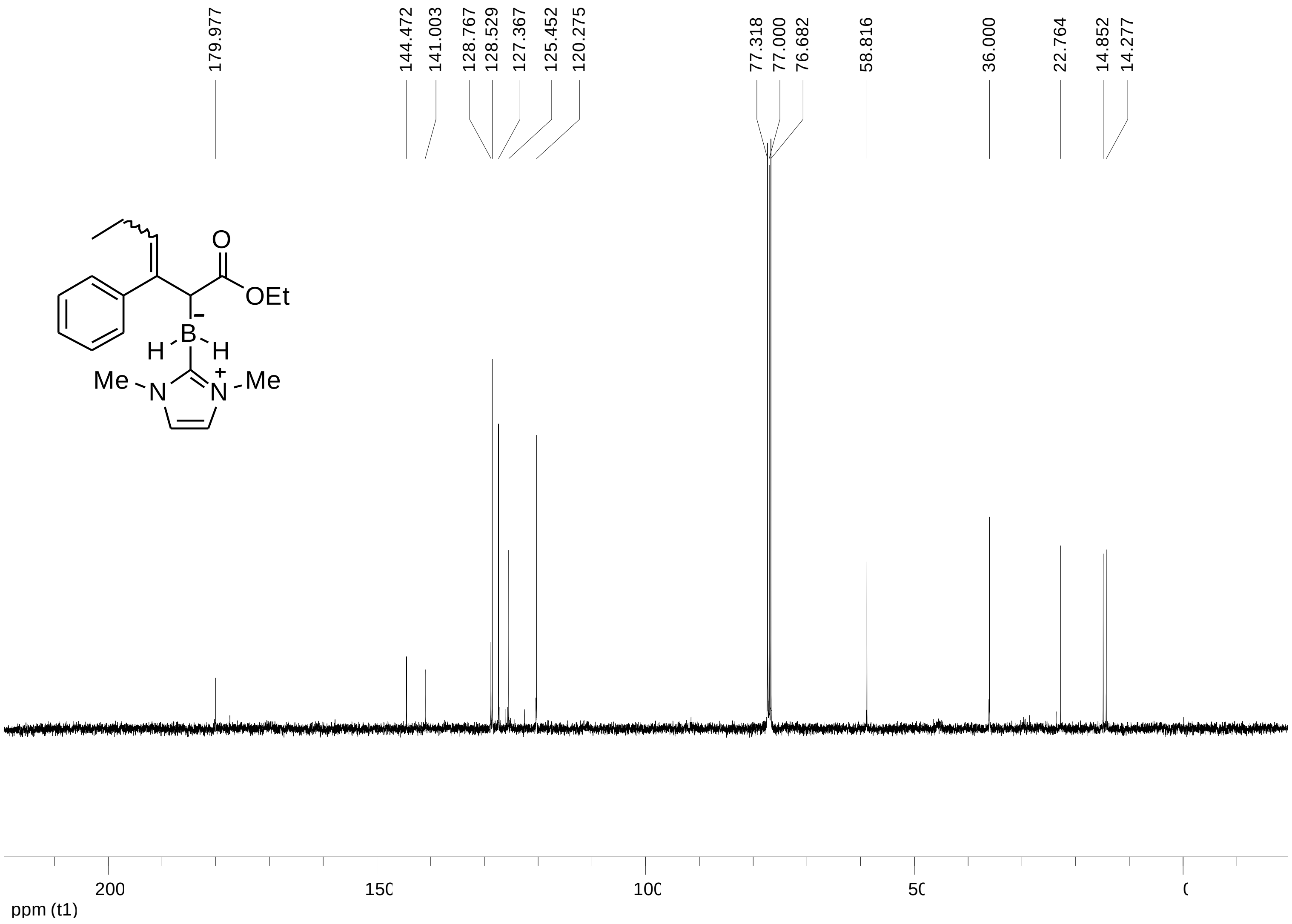

Supplementary Figure 182. ^{13}C NMR spectrum for 3am(one of the isomers)

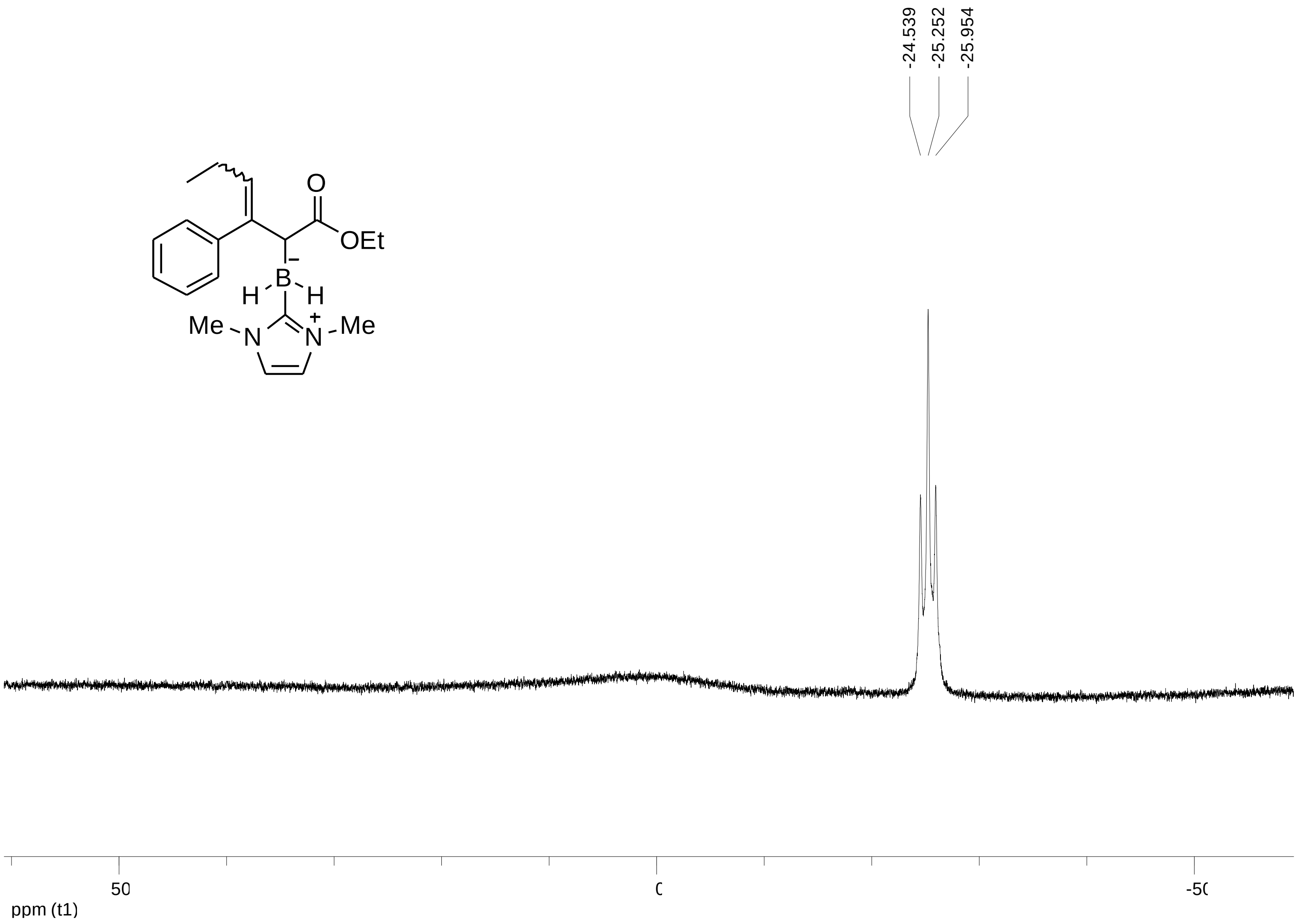

Supplementary Figure 183. ^{11}B NMR spectrum for 3am(one of the isomers)

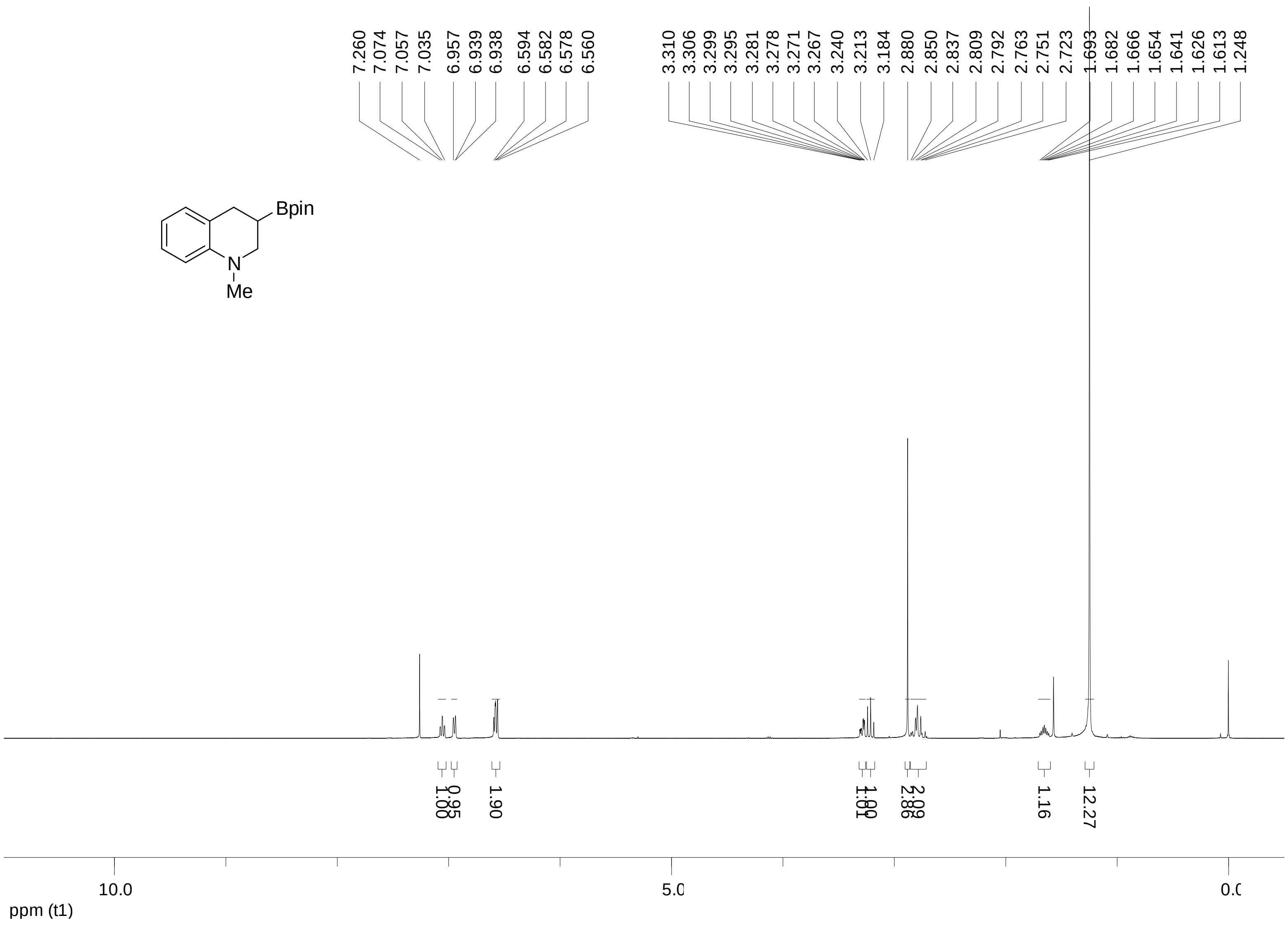

Supplementary Figure 184. ^{1}H NMR spectrum for 4

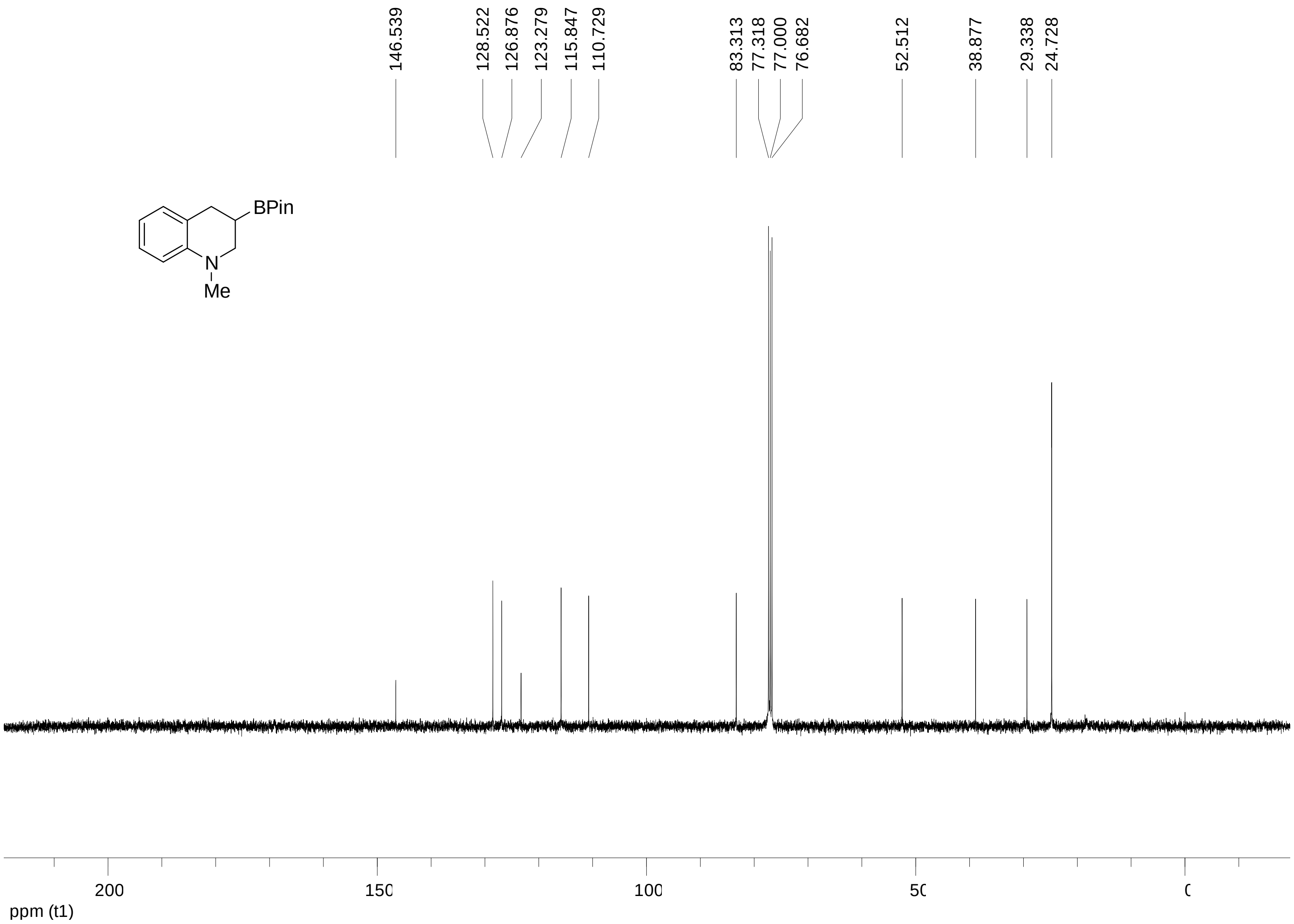

Supplementary Figure 185. ^{13}C NMR spectrum for 4

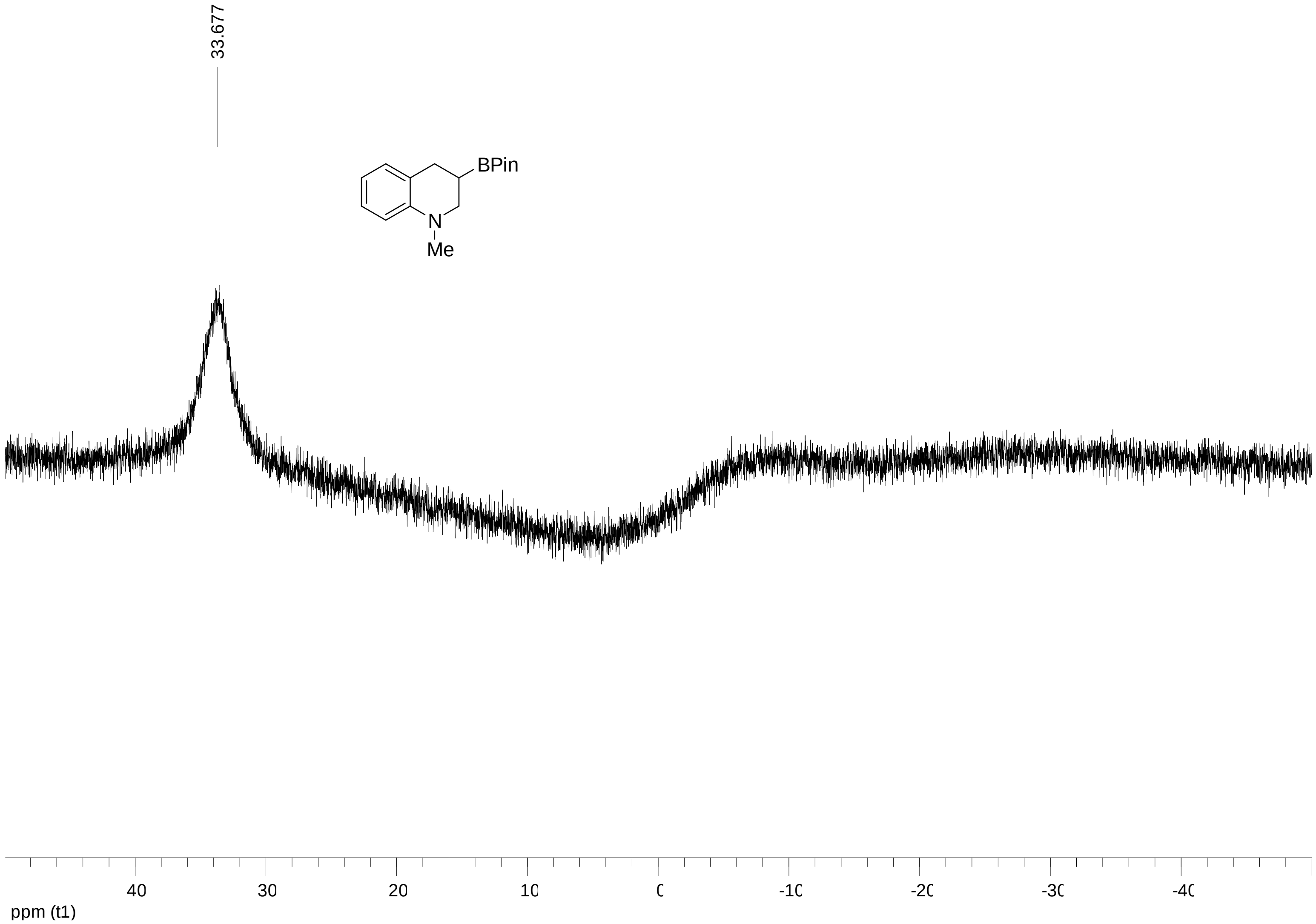

Supplementary Figure 186. ^{11}B NMR spectrum for 4

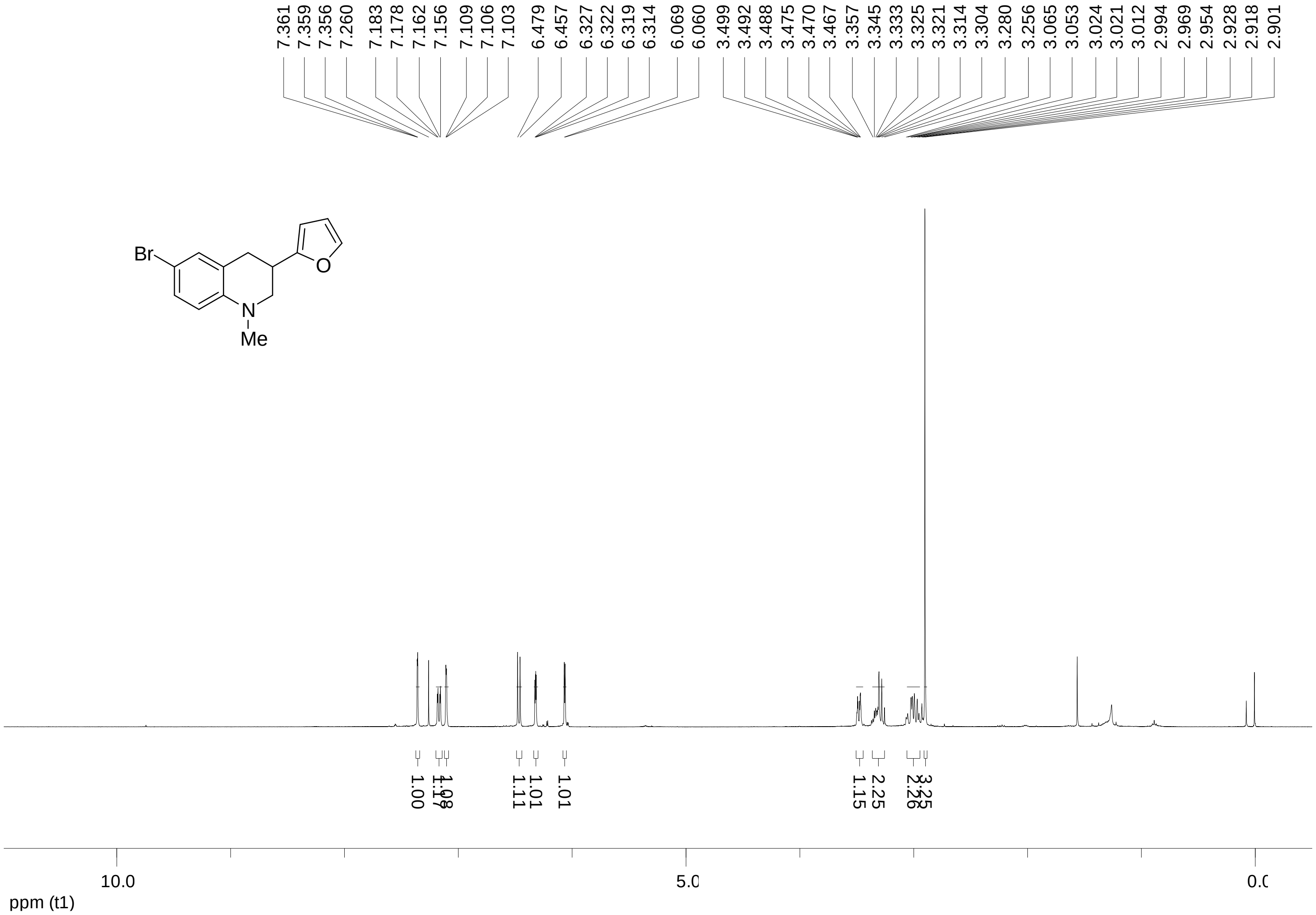

Supplementary Figure 187. ^{1}H NMR spectrum for 5

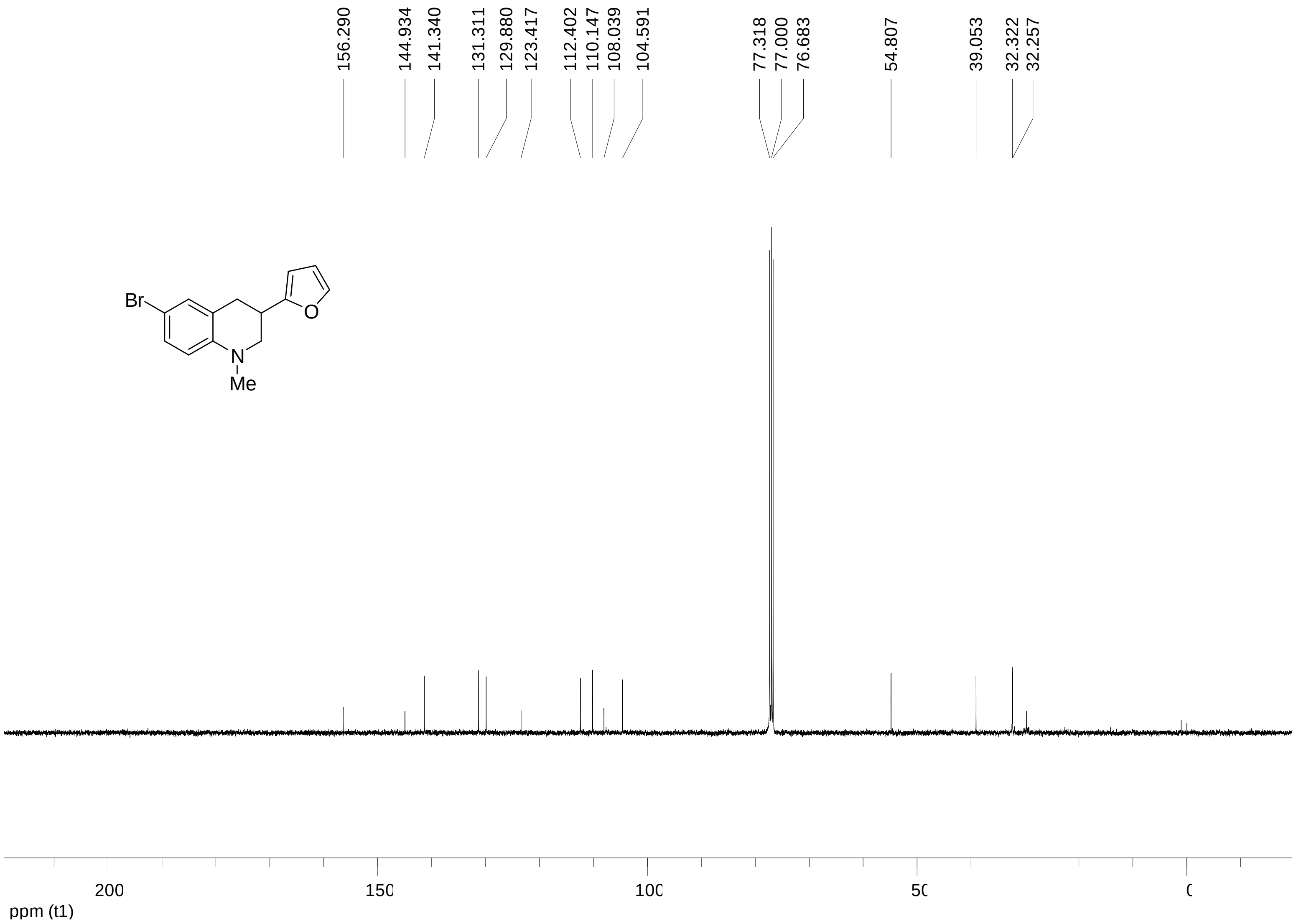

Supplementary Figure 188. ^{13}C NMR spectrum for 5

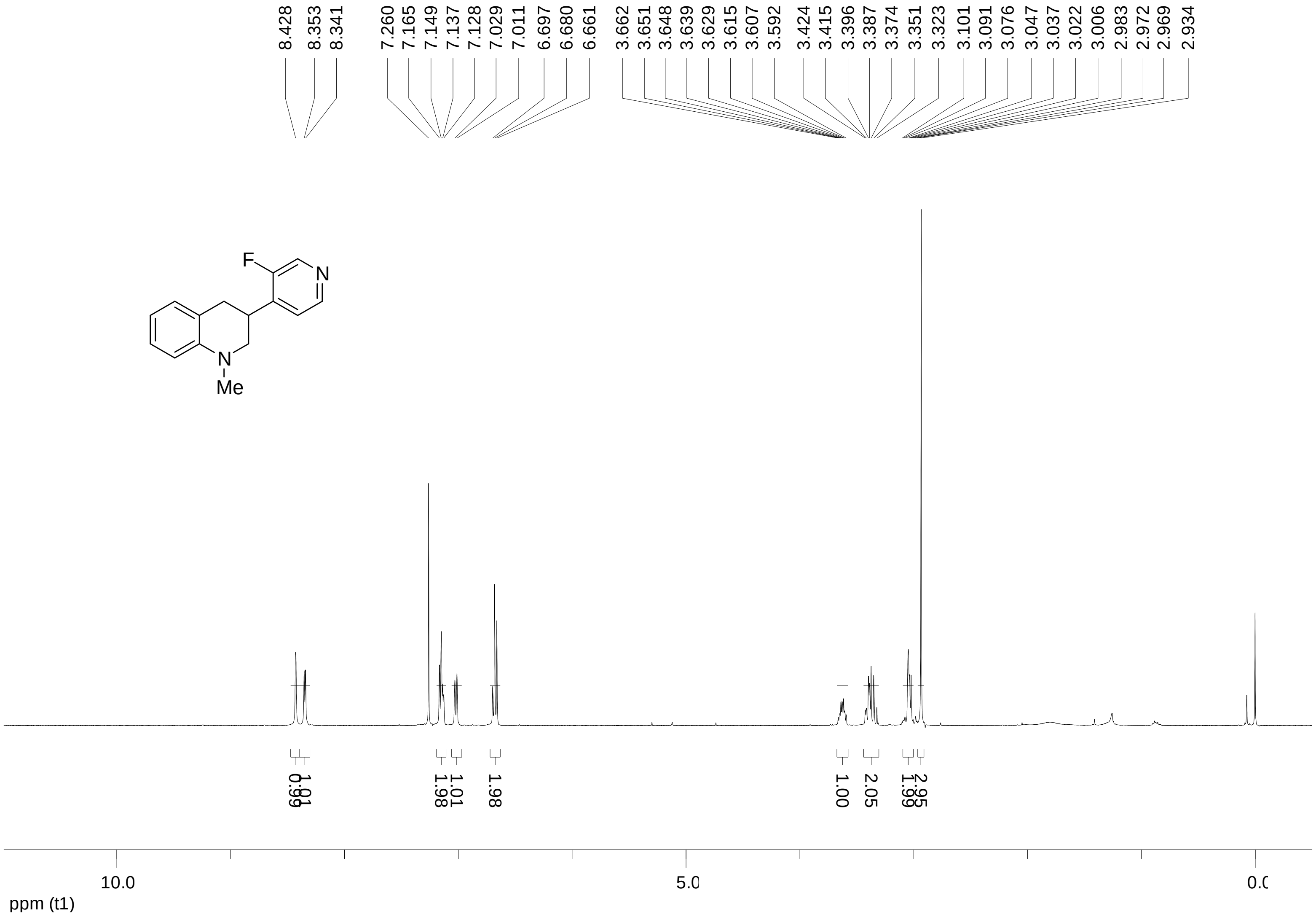

Supplementary Figure 189. ^{1}H NMR spectrum for 6

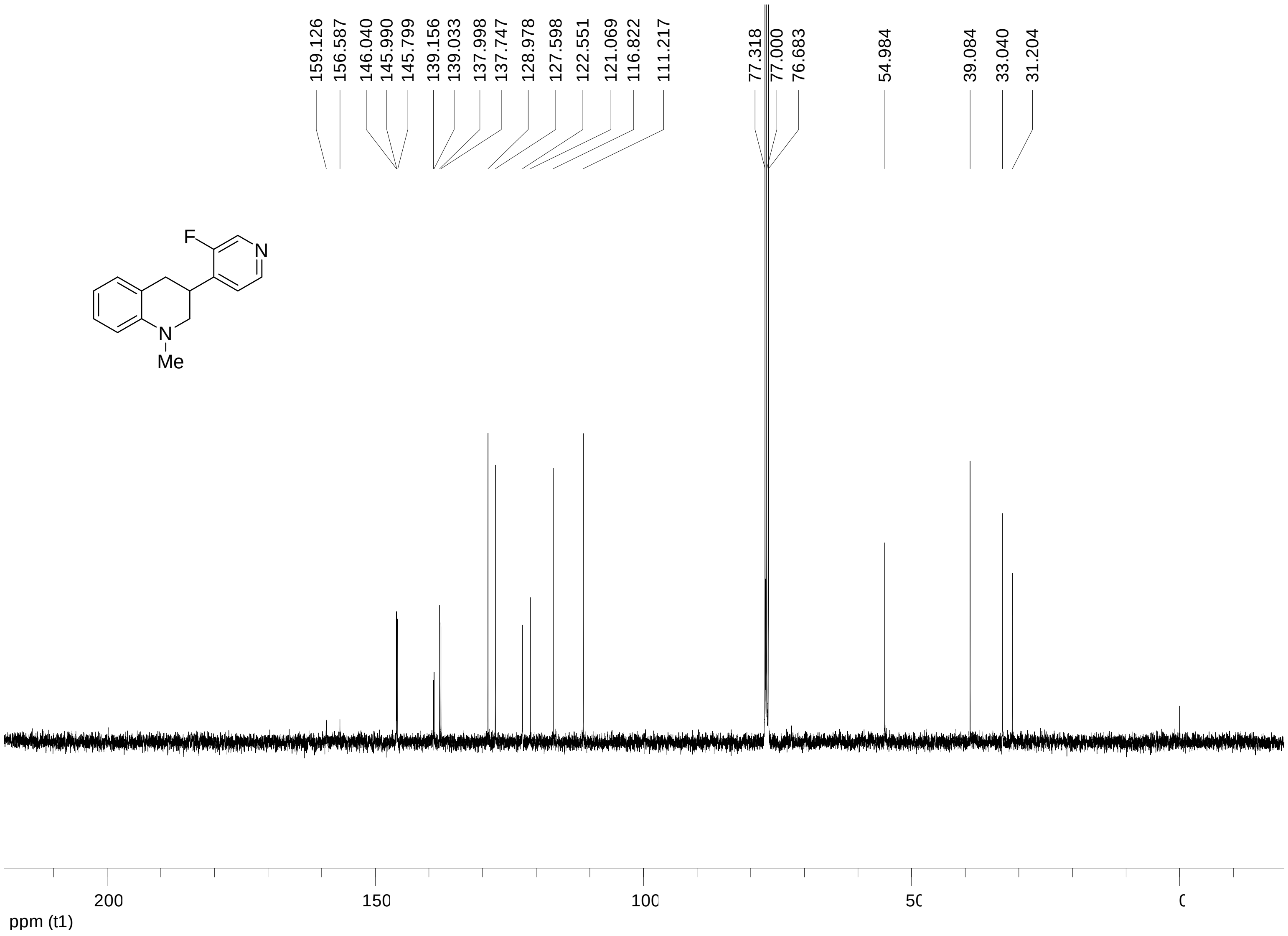

Supplementary Figure 190. ^{13}C NMR spectrum for 6

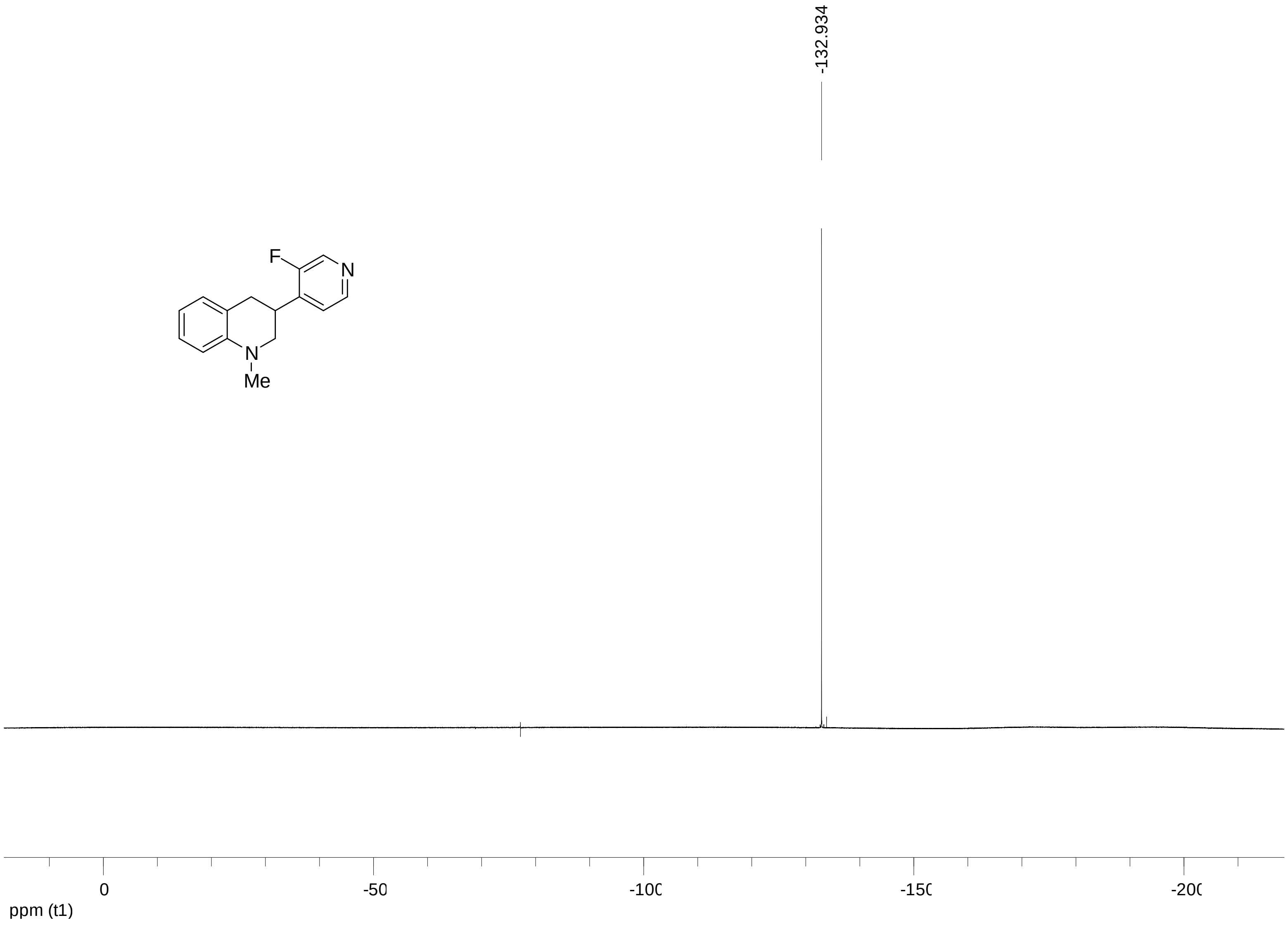

Supplementary Figure 191. ^{19}F NMR spectrum for 6

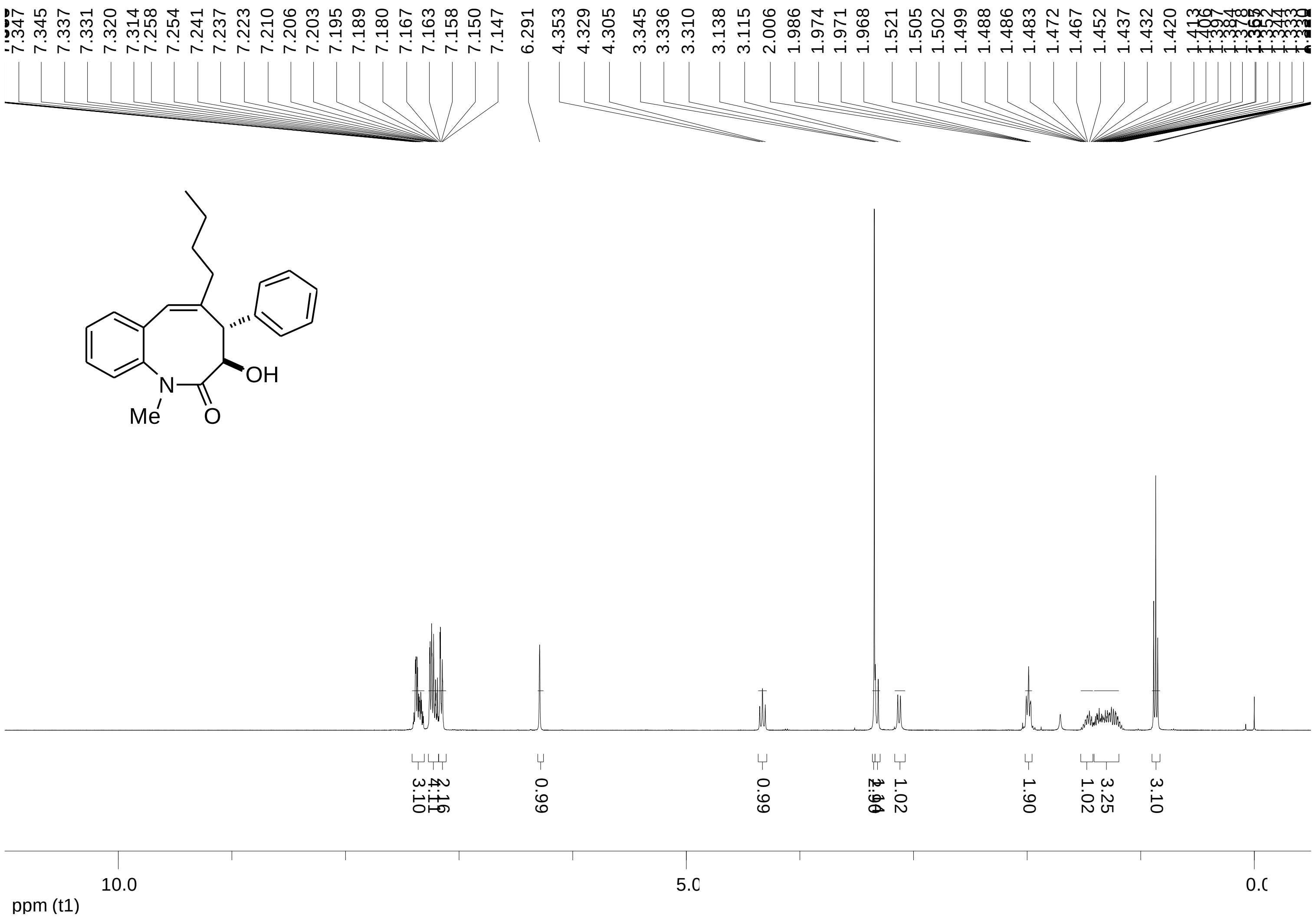

Supplementary Figure 192. ^{1}H NMR spectrum for 7

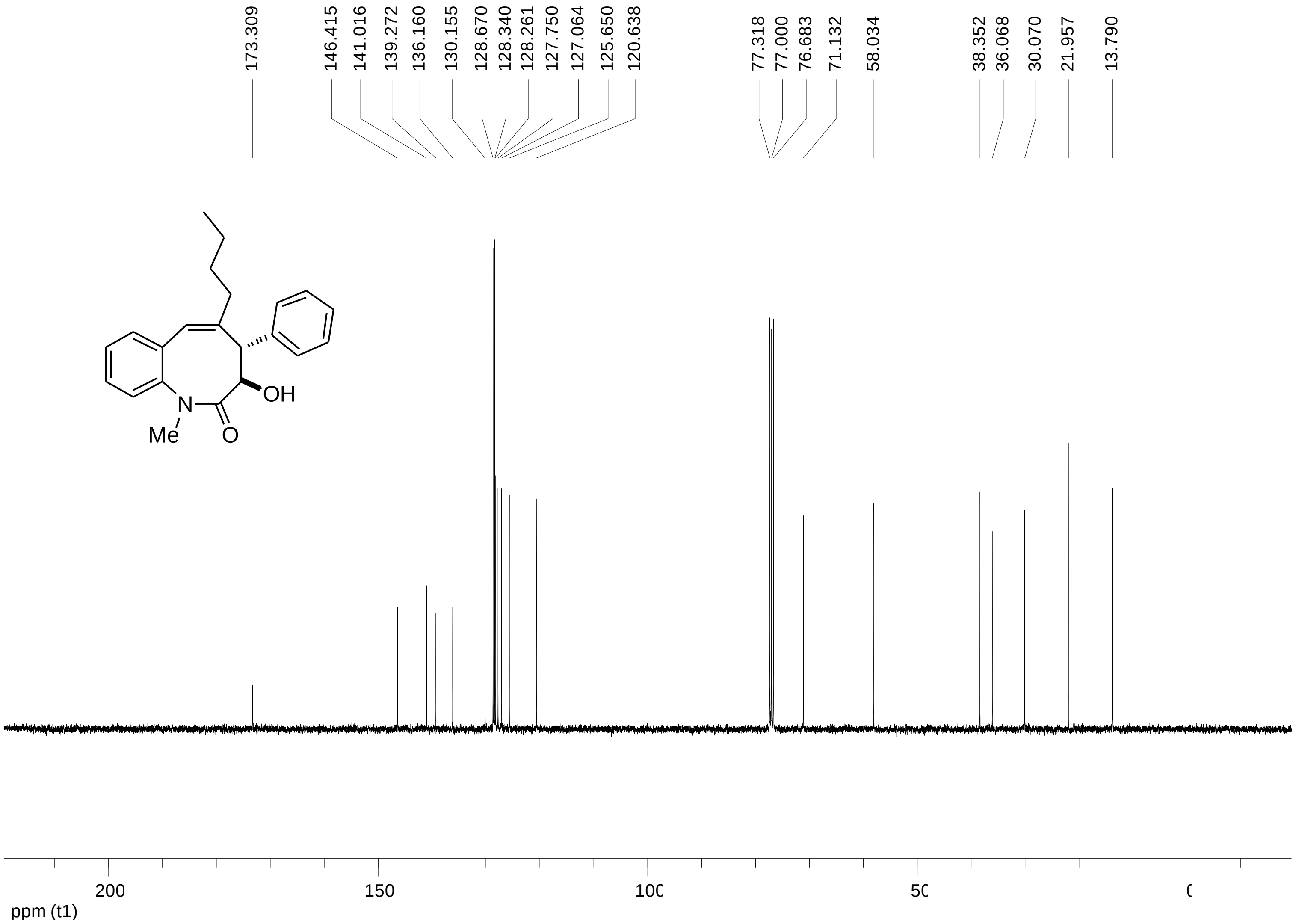

Supplementary Figure 193. ^{13}C NMR spectrum for 7

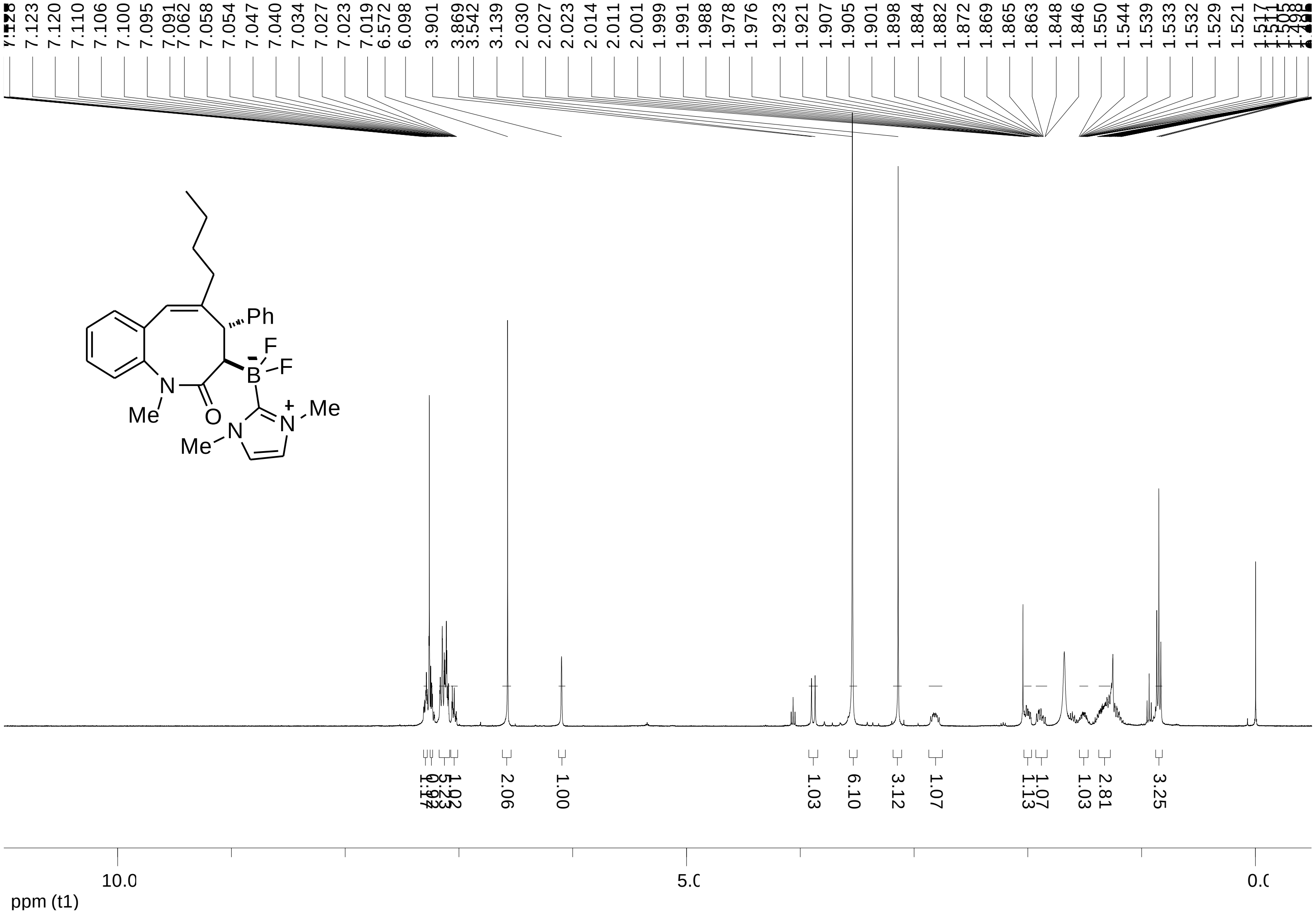

Supplementary Figure 194. ^{1}H NMR spectrum for 8

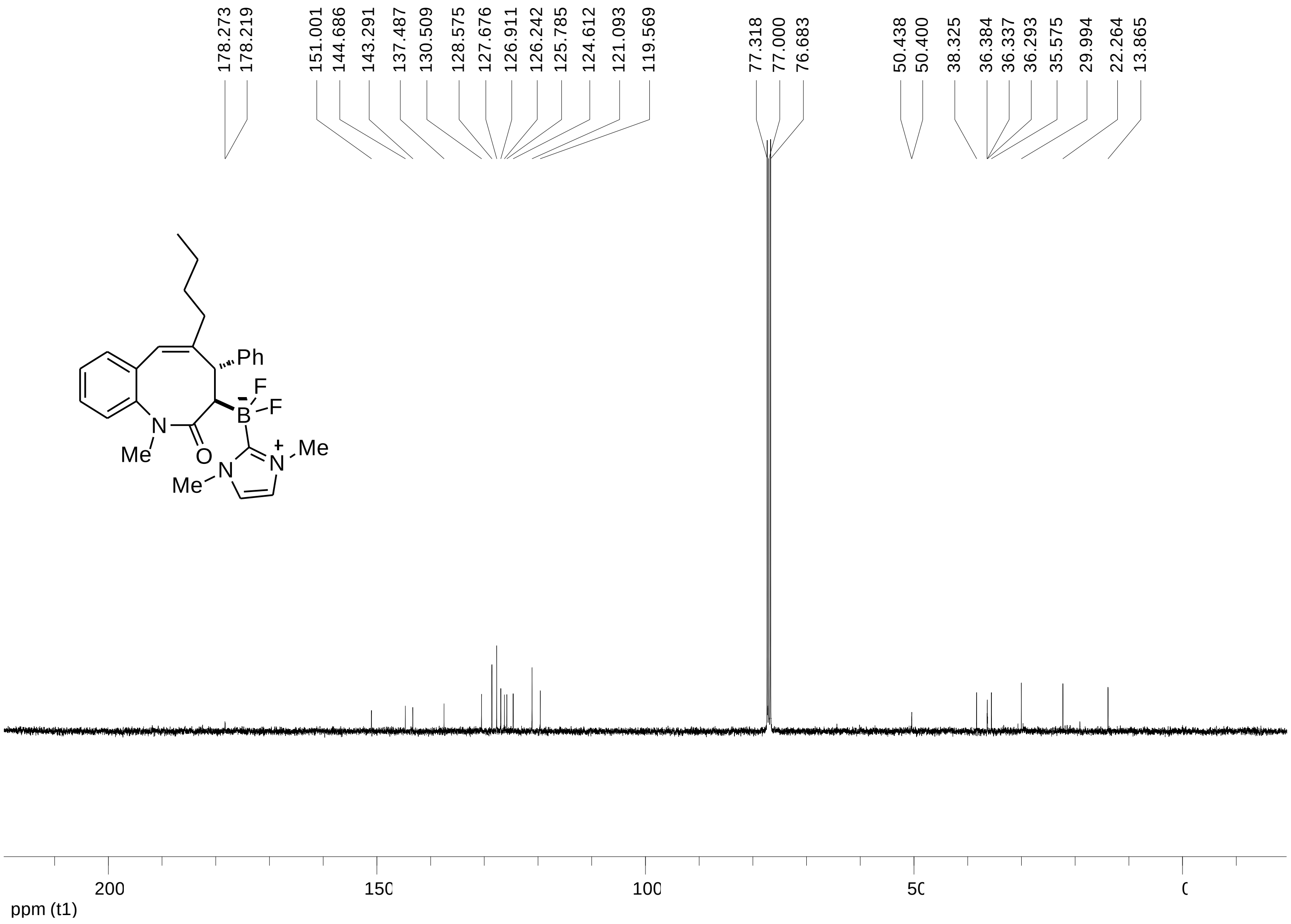

Supplementary Figure 195. ^{13}C NMR spectrum for 8

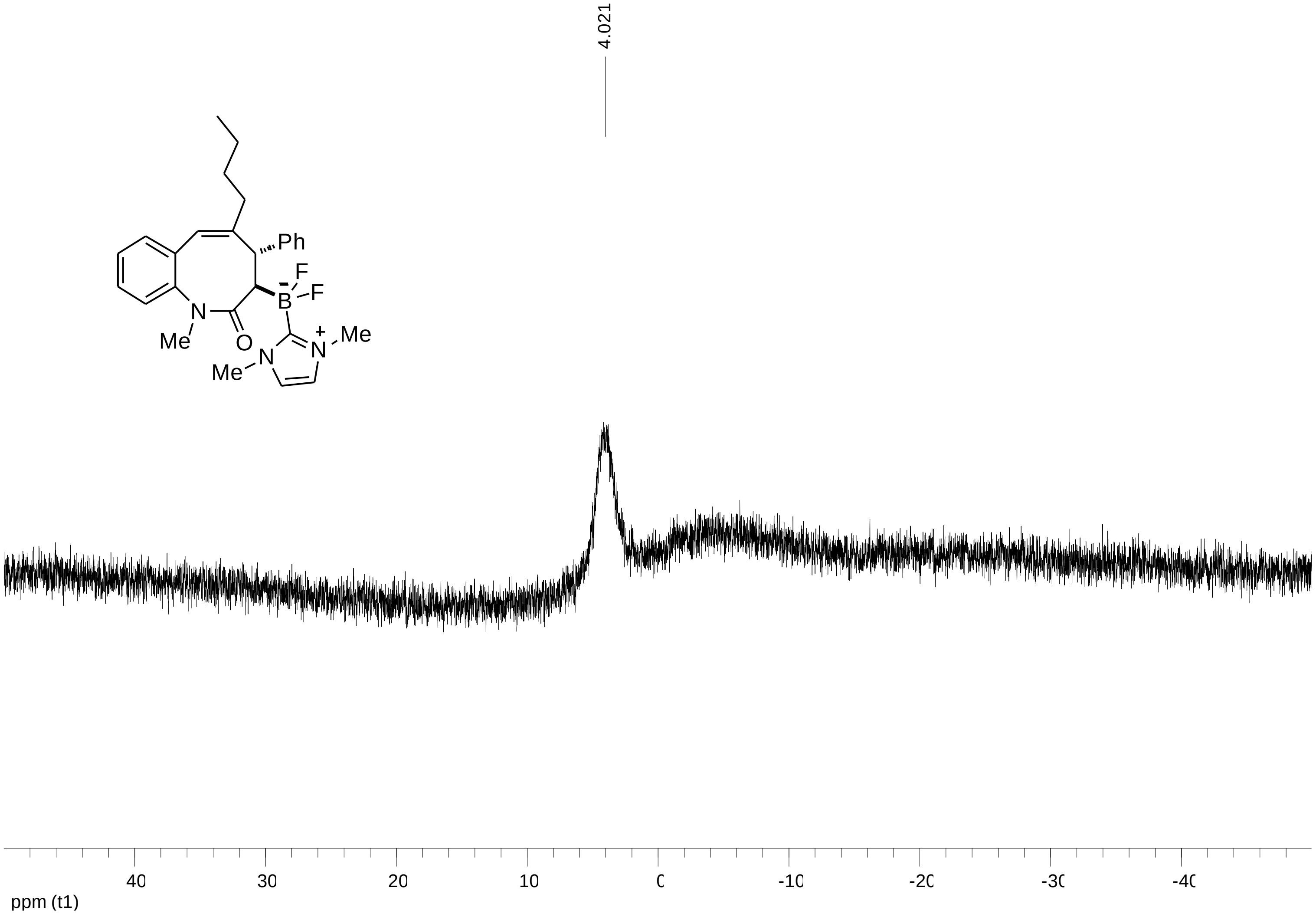

Supplementary Figure 195. ^{11}B NMR spectrum for 8

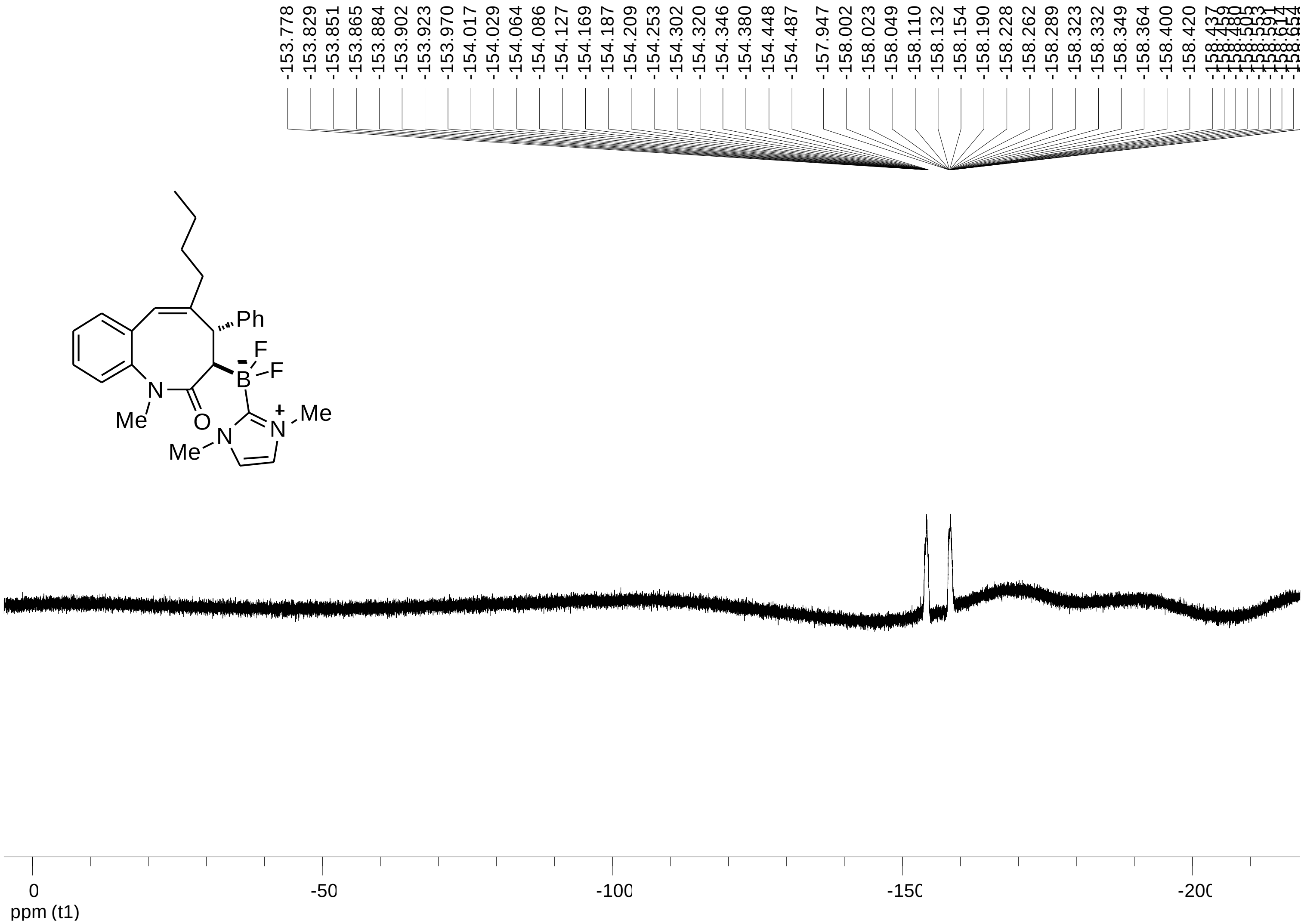

Supplementary Figure 197. ^{19}F NMR spectrum for 8

Supplementary References

(1) Gardner, S., Kawamoto, T., Curran, D. P. 1,3-Dimethylimidazoyl-2-ylidene borane. *Org. Synth.* **92**, 342 (2015).

(2) Barbero, M.; Dughera, S. Gold catalyzed Heck-coupling of arenediazonium o-benzenedisulfonimides. *Org. Biomol. Chem.* **16**, 295 (2018).

(3) Saunthwal, R. K., Patel, M., Kumar, S., Verma, A. K. Cu(II)-catalyzed tandem synthesis of 2-imino[1,3]benzothiazines from 2-aminoaryl acrylates via thioamidation and concomitant chemoselective thia-Michael addition. *Tetrahedron Lett.* **56**, 677 (2015).

(4) Zhang, B., Lv, C., Li, W., Cui, Z., Chen, D., Cao, F., Miao, F., Zhou, L. Ethyl cinnamate derivatives as promising high-efficient acaricides against p*soroptes cuniculi*: synthesis, bioactivity and structure–activity relationship. *Chem. Pharm. Bull.* **63**, 255 (2015).

(5) Song, C., Yang, C., Zeng, H., Zhang, W., Guo, S., Zhu, J. Rh(III)-catalyzed enaminone-directed C–H coupling with α-diazo- α-phosphonoacetate for reactivity discovery: fluoride-mediated dephosphonation for C–C coupling reactions. *Org. Lett.* **20**, 3819 (2018).

(6) Korytiaková, E., Thiel, N. O., Pape, F., Teichert, J. F. Copper(I)-catalysed transfer hydrogenations with ammonia borane. *Chem. Commun.* **53**, 732 (2017).

(7) Wang, X.-B., Zou, X.-L., Du, G.-F., Liu, Z.-Y., Dai, B. Nucleophilic carbene-catalyzed redox-esterification reaction of α-halo-α,β-unsaturated aldehyde. *Tetrahedron* **68**, 6498 (2012).

(8) Sharma, R., Kumar, R., Kumar, I., Sharma, U. Rh(III)-catalyzed dehydrogenative coupling of quinoline N-Oxides with alkenes: N-Oxide as traceless directing group for remote C–H activation. *Eur. J. Org. Chem.* **2015**, 7519 (2015).

(9) Dakarapu, U. S., Bokka, A., Asgari, P., Trog, G., Hua, Y., Nguyen, H. H., Rahman, N., Jeon, J. Lewis base activation of silyl acetals: iridium-catalyzed reductive Horner–Wadsworth–Emmons olefination. *Org. Lett.* **17**, 5792 (2015).

(10) Miaskiewicz, S., Reed, J. H., Donets, P. A., Oliveira, C. C., Cramer, N. Chiral 1,3,2-diazaphospholenes as catalytic molecular hydrides for enantioselective conjugate reductions. *Angew. Chem. In. Ed.* **57**, 4039 (2018).

(11) Liu, J., Liu, Q., Franke, R., Jackstell, R., Beller, M. Ligand-controlled palladium-catalyzed alkoxycarbonylation of allenes: regioselective synthesis of α,β- and β,γ-unsaturated

esters. *J. Am. Chem. Soc.* **137**, 8556 (2015).

(12) Chen, Y., Turlik, A., Newhouse, T. R. Amide α,β-dehydrogenation using allyl-palladium catalysis and a hindered monodentate anilide. *J. Am. Chem. Soc.* **138**, 1166 (2016).

(13) Evans, D. A., Chapman, K. T., Bisaha, Asymmetric Diels-Alder cycloaddition reactions with chiral α,β -unsaturated N-acyloxazolidinones. *J. Am. Chem. Soc.* **110**, 1238(1988).

(14) Lin, S.-X., Sun, G.-J., Kang, Q. A visible-light-activated rhodium complex in enantioselective conjugate addition of α-amino radicals with Michael acceptors. *Chem. Commun.* **53**, 7665 (2017).

(15) Homsi, F., Rousseau, G. 4-Endo-trig cyclization processes using bis(collidine)bromine(I) hexafluorophosphate as reagent: preparation of 2-Oxetanones, 2-azetidinones, and oxetanes. *J. Org. Chem.* **64**, 81 (1999).

(16) Sharma, V., Kelly, G. T., Watanabe, C. M. H., Exploration of the molecular origin of the azinomycin epoxide: timing of the biosynthesis revealed. *Org. Lett.* **10**, 4815 (2008).

(17) Chang, J. W. W.; Ton, T. M. U.; Tania, S.; Taylor, P. C.; Chan, P. W. H. Practical copper(I)-catalysed amidation of aldehydes. *Chem. Commun.* **46**, 922 (2010).

(18) (a) Lee, C. T., Yang, W. T., Parr, R. G. Development of the Colle-Salvetti correlation-energy formula into a functional of the electron density. *Phys. Rev. B* **37**, 785(1988).

(19) Grimme, S., Antony, J.; Ehrlich, S., Krieg, H. A consistent and accurate ab initio parametrization of density functional dispersion correction (DFT-D) for the 94 elements H-Pu. *J. Chem. Phys.***132**, 154104 (2010).

(20) Frisch, M. J., Trucks, G. W., Schlegel, H. B., Scuseria, G. E., Robb, M. A., Cheeseman, J. R., Scalmani, G., Barone, V., Mennucci, B., Petersson, G. A., Nakatsuji, H., Caricato, M., Li, X., Hratchian, H. P., Izmaylov, A. F., Bloino, J., Zheng, G., Sonnenberg, J. L., Hada, M., Ehara, M., Toyota, K., Fukuda, R., Hasegawa, J., Ishida, M., Nakajima, T., Honda, Y., Kitao, O., Nakai, H., Vreven, T., Jr. Montgomery, J. A., Peralta, J. E., Ogliaro, F., Bearpark, M., Heyd, J. J., Brothers, E., Kudin, K. N., Staroverov, V. N., Keith, T., Kobayashi, R., Normand, J., Raghavachari, K., Rendell, A., Burant, J. C., Iyengar, S. S., Tomasi, J., Cossi, M., Rega, N., Millam, J. M., Klene, M., Knox, J. E., Cross, J. B., Bakken, V., Adamo, C., Jaramillo, J., Gomperts, R., Stratmann, E., Yazyev, O., Austin, A. J., Cammi, R., Pomelli, C., Ochterski, J. W., Martin, R. L., Morokuma, K., Zakrzewski, V. G., Voth, G. A., Salvador, P., Dannenberg,

J. J., Dapprich, S., Daniels, A. D., Farkas, O., Foresman, J. B., Ortiz, J. V., Cioslowski, J., Fox, D. J. Gaussian 09, Revision D.01, Gaussian, Inc., Wallingford CT, **2013**.

(21) Marenich, A. V.; Cramer, C. J.; Truhlar, D. G. Universal solvation model based on solute electron density and on a continuum model of the solvent defined by the bulk dielectric constant and atomic surface tensions. *J. Phys. Chem. B* **113**, 6378 (2009).

(22) Fukui, K. The path of chemical reactions - the IRC approach. *Acc. Chem. Res.* **14**, 363 (1981).

(23) Fukui, K. A formulation of the reaction coordinate. *J. Phys. Chem.* **74**, 4161 (1970).

(24) Hehre, W. J.; Radom, L.; Schleyer, P. v. R.; Pople, J. A. *Ab Initio Molecular Orbital Theory*; Wiley: New York, 1986.

(25) Grande, M. d. C.; Bianchi, H. L.; Marschoff, C. M. On The density of pure acetonitrile. *J. Argent. Chem. Soc.* **92**, 109 (2004).

(26) Poh, J.-S., Makai, S., von Keutz, T., Tran, D. N., Battilocchio, C., Pasau, P., Ley, S. V. Rapid asymmetric synthesis of disubstituted allenes by coupling of flow-generated diazo compounds and propargylated amines. *Angew. Chem. Int. Ed.* **56**, 1864 (2017).

(27) Johnson, J. A., Petersen, B. M., Kormos, A., Echeverr á, E., Chen, Y.-S., Zhang, J. A new approach to non-coordinating anions: lewis acid enhancement of porphyrin metal centers in a zwitterionic metal-organic framework. *J. Am. Chem. Soc.* **138**, 10293 (2016).

(28) Saito, T., Nihei, H., Otani, T., Suyama, T., Furukawa, N., Saito, M. Thiocarbonyl induced heterocumulenic Pauson–Khand type reaction: expedient synthetic method for thieno[2,3-b]indol-2-ones. *Chem. Commun.* 172 (2008).

(29) Yin, G., Fan, L.; Ren, T., Zheng, C., Tao, Q., Wu, A., She, N. Synthesis of functionalized 2-aryl-4-(indol-3-yl)-4H-chromenes via iodine-catalyzed domino Michael addition–intramolecular cyclization reaction. *Org. Biomol. Chem.* **10**, 8877 (2012).

(30) Zhou, Q. H., Zhou, M. M., Wei, Y. X., Zhou, X. G., Liu, S. L., Zhang, S., & Zhang, B. Solvent effects on the triplet–triplet annihilation upconversion of diiodo-Bodipy and perylene. *Phys. Chem. Chem. Phys.* **19**, 1516-1525 (2017).

(31) Wei, Y., Zhou, M., Zhou, Q., Zhou, X., Liu, S., Zhang, S., & Zhang, B. Triplet–triplet annihilation upconversion kinetics of C60–Bodipy dyads as organic triplet photosensitizers. *Phys. Chem. Chem. Phys.* **19**, 22049 (2017).

(32) Tehfe, M.-A., Makhlouf Brahmi, M., Fouassier, J.-P., Curran, D. P., Malacria, M., Fensterbank, L., Lacôte, E., & Lalevée, J. N-Heterocyclic Carbenes−Borane Complexes: A New Class of Initiators for Radical Photopolymerization. *Macromolecules*. **43**, 2261-2267 (2010).
