# Supplementary material for: Regioselective radical α-borylation of α,β-unsaturated carbonyl compounds for direct synthesis of α-borylcarbonyl molecules
Source: Nat Commun. 2019 Apr 29;10:1934. doi: 10.1038/s41467-019-09825-3 (PMC6488649; doi:10.1038/s41467-019-09825-3)
Supplement: Supplementary file 2 — Description of Additional Supplementary Files [file 41467_2019_9825_MOESM2_ESM.pdf]

Description of Addition Supplementary Information

File Name: Supplementary Data 1

Description: Cartesian coordinates for all optimized geometries.
