# Supplementary material for: Regioselective radical α-borylation of α,β-unsaturated carbonyl compounds for direct synthesis of α-borylcarbonyl molecules
Source: Nat Commun. 2019 Apr 29;10:1934. doi: 10.1038/s41467-019-09825-3 (PMC6488649; doi:10.1038/s41467-019-09825-3)
Supplement: Supplementary file 3 — Supplementary Data 1 [file 41467_2019_9825_MOESM3_ESM.pdf]

Cartesian coordinates for all optimized geometries

1a

C	-3.58588500	1.54893100	0.00015600
C	-2.24574900	1.16663200	0.00030800
C	-1.88874600	-0.19909200	0.00011000
C	-2.91941300	-1.16105200	-0.00019100
C	-4.26208200	-0.77663100	-0.00034400
C	-4.59991200	0.58029400	-0.00018600
H	-3.84349500	2.60511600	0.00030900
H	-1.47576500	1.93292500	0.00061200
H	-2.65918500	-2.21722600	-0.00031100
H	-5.04144900	-1.53452700	-0.00058400
H	-5.64366000	0.88413400	-0.00030500
C	-0.50073500	-0.66689600	0.00019500
H	-0.37275600	-1.74925600	0.00034800
C	0.61760700	0.08793600	0.00002200
H	0.59294700	1.17326900	-0.00021200
C	1.95174900	-0.53868900	0.00012200
O	2.92348700	0.39901500	0.00014000
C	4.29912900	-0.07271500	0.00007800
H	4.45337600	-0.69428000	-0.88816500
H	4.45361500	-0.69364400	0.88873000
C	5.19755100	1.14703200	-0.00045700
H	6.24552400	0.82447500	-0.00052900
H	5.02572500	1.76169000	-0.89162500
H	5.02602700	1.76226500	0.89037400
O	2.18376500	-1.74269400	0.00016900

NHC-BH_2•

C	0.68220000	-1.51073800	0.00002600
C	-0.68220100	-1.51073800	0.00007300
H	1.38716400	-2.32900200	0.00008000
H	-1.38716500	-2.32900200	0.00017500
C	0.00000000	0.65631800	0.00002500
N	1.09442500	-0.19378300	0.00001300
N	-1.09442500	-0.19378300	0.00002600
C	2.47771400	0.25343500	-0.00011200
H	3.12883500	-0.62264100	-0.00065700
H	2.68069800	0.85692100	-0.89084400
H	2.68114600	0.85612700	0.89106500
C	-2.47771400	0.25343600	-0.00013700
H	-2.68063200	0.85703500	-0.89080500
H	-3.12883500	-0.62264000	-0.00083700
H	-2.68121200	0.85601200	0.89110400
B	0.00000100	2.16434300	0.00012400
H	-1.04262200	2.75908000	0.00030300
H	1.04262400	2.75907900	0.00026500

1a-TS-1

C	-2.97739300	-1.93297400	1.31518300
C	-1.65498400	-1.67453500	0.96241300
C	-1.28687500	-1.46393500	-0.39128600
C	-2.31481000	-1.53308700	-1.36498800
C	-3.63655300	-1.79614700	-1.00861900
C	-3.98016100	-1.99765600	0.33555200
H	-3.23163300	-2.08447000	2.36205000
H	-0.89997000	-1.62659900	1.74166600
H	-2.06041100	-1.36663800	-2.41000800
H	-4.40318100	-1.83959300	-1.77917800
H	-5.01080300	-2.19967000	0.61628500
C	0.06241400	-1.15133800	-0.80781000
H	0.21370100	-1.01411600	-1.87690500
C	1.14152900	-0.91956200	0.01962800
H	1.10282800	-1.13467800	1.08130400
C	2.47095000	-0.68719400	-0.54468300
O	3.44101600	-0.77956800	0.40259400

C	4.79834900	-0.50421900	-0.01912400
H	4.83637000	0.50439200	-0.44576300
H	5.08097000	-1.21795100	-0.80073400
C	5.68844500	-0.63013300	1.20155100
H	6.72822300	-0.42667200	0.91837900
H	5.39508800	0.08708400	1.97697100
H	5.63737800	-1.64141900	1.62199900
O	2.72882700	-0.46109300	-1.72776000
C	-2.54021300	1.92384800	0.31370000
C	-2.08770400	2.07042200	-0.96608800
H	-3.54354700	1.90496500	0.71162100
H	-2.62184800	2.20541600	-1.89445900
C	-0.28567500	1.82014100	0.37059800
N	-1.43365300	1.76819500	1.11945100
N	-0.71231300	1.99258000	-0.92018900
C	-1.47439900	1.55259700	2.56032300
H	-2.51378500	1.60212900	2.88778000
H	-1.06418200	0.56863600	2.80264300
H	-0.89284600	2.32444500	3.07115700
C	0.17351300	2.12938900	-2.07252500
H	-0.42644600	2.04856800	-2.98060000
H	0.67175300	3.10439200	-2.04982600
H	0.92397700	1.33758800	-2.05818800
B	1.15143800	1.59147300	0.83625600
H	2.05019200	1.89482100	0.11020000
H	1.35540200	1.37816400	1.99587600

1a-Int-1

C	2.82846700	-0.95697100	-1.84060400
C	1.56563300	-0.80443200	-1.27832700
C	1.14183000	-1.61461900	-0.17855700
C	2.07507700	-2.58794700	0.30128200
C	3.33286500	-2.73234500	-0.26958200
C	3.72681200	-1.91761400	-1.34672400
H	3.12208700	-0.31902400	-2.67163200
H	0.89882000	-0.04634100	-1.67686000
H	1.78478400	-3.21831100	1.13973500
H	4.01874500	-3.47990500	0.12347200
H	4.71300700	-2.02918500	-1.78998900
C	-0.11963500	-1.48036100	0.45131400
H	-0.32599800	-2.13069500	1.29894500
C	-1.13761900	-0.44955100	0.14563100
H	-1.10800800	-0.12401600	-0.89807800
C	-2.52932100	-0.89001700	0.47117000
O	-3.43851500	-0.33832100	-0.36572200
C	-4.83937000	-0.59769800	-0.08608000
H	-5.06652300	-0.24480200	0.92569800
H	-5.01174000	-1.67868200	-0.12069200
C	-5.65443600	0.13462300	-1.13315200
H	-6.72185500	-0.04141800	-0.95324100
H	-5.47084300	1.21451600	-1.08989200
H	-5.41142000	-0.22244000	-2.14083400
O	-2.85401100	-1.62274400	1.39816700
C	2.02467900	2.80984600	-0.50082700
C	2.68651000	2.19757600	0.51857600
H	2.37333000	3.46309700	-1.28653500
H	3.72888200	2.20612300	0.79965400
C	0.51287700	1.62630300	0.68049800
N	0.69504700	2.45015500	-0.38445100
N	1.74413900	1.47947500	1.23300100
C	-0.36041400	2.87868500	-1.30142000
H	0.08298900	3.53489600	-2.05193200
H	-0.80398700	2.01017400	-1.79343900
H	-1.13503600	3.41962200	-0.75357100
C	2.09177300	0.63597100	2.37554900
H	2.58742500	-0.27250900	2.02302900
H	2.76439300	1.19139700	3.03374300

H	1.18544800	0.36939000	2.91596000
B	-0.87579300	0.94351900	1.10756400
H	-0.85257000	0.62078400	2.27928300
H	-1.78141800	1.73215000	0.89746700

PhSH

C	-1.60271700	-1.20234600	0.00004700
C	-0.20513100	-1.21535400	0.00000700
C	0.50645700	-0.00487800	0.00016300
C	-0.19137900	1.21294000	0.00007800
C	-1.58968400	1.21325500	-0.00009100
C	-2.30240300	0.00949500	-0.00001700
H	-2.14313000	-2.14577600	-0.00000700
H	0.32429200	-2.16535100	-0.00000300
H	0.34727200	2.15726500	0.00004600
H	-2.11987200	2.16258600	-0.00018400
H	-3.38923000	0.01560900	-0.00010900
S	2.29789800	-0.08439600	-0.00008600
H	2.52344600	1.24733200	0.00052400

1a-TS-2

C	-1.36266900	-2.26321100	-1.40895900
C	-0.51968300	-1.24576900	-0.96814200
C	-0.65940700	-0.69527000	0.32883400
C	-1.70048400	-1.19609800	1.14877900
C	-2.54501600	-2.20953000	0.70372700
C	-2.38035600	-2.75262400	-0.57875200
H	-1.22974200	-2.67497500	-2.40660200
H	0.25485100	-0.87410000	-1.63058500
H	-1.83299100	-0.77749100	2.14384800
H	-3.33569800	-2.57680700	1.35354300
H	-3.03939500	-3.54358000	-0.92770400
C	0.18007700	0.38732400	0.81941200
H	0.06763000	0.60249700	1.88310400
C	1.54830400	0.65778400	0.27394000
H	1.57261100	0.59477300	-0.81733500
C	2.06781000	1.99909300	0.68749000
O	2.84672300	2.55482900	-0.26785600
C	3.49720100	3.81190900	0.05766400
H	4.11814900	3.66520100	0.94799500
H	2.72791700	4.55523900	0.29202300
C	4.32459000	4.21932700	-1.14481000
H	4.83111900	5.16886300	-0.93382700
H	5.08682500	3.46466800	-1.37116200
H	3.69146900	4.35561700	-2.02939400
O	1.86867700	2.53893600	1.76881700
C	2.17288100	-3.70721400	-1.04730100
C	1.63544300	-4.05826200	0.15346100
H	2.27928300	-4.25800900	-1.96967800
H	1.18198900	-4.97843600	0.48964400
C	2.36041600	-1.93312000	0.32877200
N	2.61713000	-2.40406900	-0.91833800
N	1.75759200	-2.95808600	0.98239000
C	3.19507500	-1.62746900	-2.01265300
H	3.92328800	-0.92157600	-1.61233000
H	3.68895200	-2.31302000	-2.70386800
H	2.40905200	-1.07932900	-2.54053300
C	1.31161500	-2.92646700	2.37586000
H	0.70363000	-3.81339100	2.55979100
H	2.17452800	-2.92717000	3.04667500
H	0.71159200	-2.03380400	2.55171400
B	2.67116200	-0.46375100	0.89325800
H	2.60846300	-0.46841200	2.10663700
H	3.78417700	-0.12370100	0.52479800
S	-1.43073700	2.77244400	-0.14580600
H	-0.59708200	1.57497200	0.34832500
C	-2.96896600	1.86408200	-0.23369700

C	-3.28613300	1.10351700	-1.37350700
C	-3.87608200	1.89631700	0.84143800
C	-4.47950700	0.37954100	-1.42956300
H	-2.59037400	1.07009000	-2.20688500
C	-5.07158500	1.17495500	0.77862800
H	-3.63820200	2.47914000	1.72714200
C	-5.37546200	0.41147600	-0.35434400
H	-4.70645100	-0.21396000	-2.31188700
H	-5.76202000	1.20471200	1.61828700
H	-6.30221500	-0.15513600	-0.39886100

3a

C	4.43837200	0.35345000	-1.39718300
C	3.30926000	-0.43792000	-1.16580300
C	2.93111900	-0.80010800	0.13866200
C	3.72324900	-0.35003000	1.20570800
C	4.85728800	0.43988100	0.98149600
C	5.21843000	0.79741800	-0.32185600
H	4.71174500	0.62069800	-2.41567900
H	2.71074800	-0.78047400	-2.00806800
H	3.44583700	-0.61709700	2.22338000
H	5.45548900	0.77696400	1.82524900
H	6.09829600	1.41140100	-0.49934800
C	1.69553600	-1.64332300	0.37062000
H	1.66717800	-1.97700900	1.41404100
C	0.38491200	-0.89514300	0.03174600
H	0.47795100	-0.42983500	-0.95739400
C	-0.77408100	-1.83397300	-0.01604700
O	-1.77859300	-1.35570000	-0.79489800
C	-3.02537800	-2.09480100	-0.81068000
H	-3.36292100	-2.24413200	0.22013800
H	-2.84791300	-3.07830200	-1.25985700
C	-4.02199700	-1.28122100	-1.61230100
H	-4.98199600	-1.81022100	-1.64860300
H	-4.18310600	-0.29997100	-1.15178600
H	-3.67166200	-1.13172400	-2.64041500
O	-0.87777200	-2.89163400	0.59682400
C	-3.23380300	2.11011000	0.32750900
C	-2.39344200	2.69893900	-0.56649700
H	-4.28766500	2.25087100	0.51495500
H	-2.56568000	3.45537200	-1.31743300
C	-1.18576500	1.21978800	0.63308500
N	-2.47553100	1.20511000	1.05092100
N	-1.14194400	2.14365900	-0.36034200
C	-3.00969700	0.35265800	2.11268900
H	-4.09814300	0.42581000	2.09331900
H	-2.71303000	-0.68299300	1.94405100
H	-2.63804800	0.68253800	3.08620800
C	0.03434000	2.45801900	-1.16864700
H	0.10430200	1.76798900	-2.01463800
H	-0.05856600	3.48105300	-1.53831800
H	0.93120600	2.36962900	-0.55504600
B	0.03262300	0.30304900	1.14623200
H	1.01596500	1.01554500	1.27458700
H	-0.25621300	-0.19592100	2.22186700
H	1.76628700	-2.55061600	-0.24683100

PhS•

C	1.53879100	1.21837900	0.00000300
C	0.14948600	1.22495400	0.00000800
C	-0.57919900	0.00000800	-0.00002500
C	0.14948400	-1.22495400	0.00000400
C	1.53877600	-1.21839000	0.00000600
C	2.23792700	0.00000100	-0.00000900
H	2.08498100	2.15769100	0.00000900
H	-0.39498800	2.16464100	0.00001400
H	-0.39501800	-2.16462500	0.00001200

H	2.08498800	-2.15768700	0.00001400
H	3.32462600	-0.00001700	-0.00000600
S	-2.30726100	0.00000000	0.00000200

1a-TS-1'

C	-3.16029700	-0.95246700	-1.71604900
C	-1.84887500	-0.87062100	-1.24602600
C	-1.47654000	-1.47400100	-0.02475700
C	-2.47505400	-2.16053100	0.70153600
C	-3.78485700	-2.24345600	0.23028100
C	-4.13807100	-1.63648500	-0.98314800
H	-3.42295300	-0.46972700	-2.65479100
H	-1.11447900	-0.31635400	-1.82294400
H	-2.21181600	-2.62550200	1.64941600
H	-4.53314300	-2.77893900	0.81021800
H	-5.15990500	-1.69429600	-1.34984600
C	-0.11029900	-1.42220300	0.50573400
H	0.04380300	-1.91242100	1.46442800
C	1.01336600	-1.12238800	-0.24044500
H	0.93718900	-0.72102400	-1.24579000
C	2.34327000	-1.23789000	0.30894700
O	3.29136300	-0.80608200	-0.57989900
C	4.66553500	-0.83084600	-0.13370900
H	4.94186100	-1.86073600	0.11982700
H	4.75657600	-0.22143200	0.77264900
C	5.52123900	-0.28454800	-1.26021500
H	6.57465800	-0.28779200	-0.95512200
H	5.42207300	-0.89901600	-2.16280900
H	5.23646200	0.74548300	-1.50530900
O	2.64839700	-1.65354300	1.43233600
C	-1.43327000	2.84672600	-0.78314100
C	-0.07382400	2.95486100	-0.82280300
H	-2.19675100	3.28120400	-1.41109700
H	0.57459100	3.50313500	-1.49008100
C	-0.60081200	1.59839200	0.90431400
N	-1.74412600	2.01158100	0.26709600
N	0.42588500	2.18624000	0.20775000
C	-3.10287100	1.69371700	0.69779900
H	-3.79709300	2.06771100	-0.05587900
H	-3.22316300	0.61527600	0.79615000
H	-3.31113500	2.17512900	1.65836900
C	1.84900500	2.11764700	0.53053100
H	2.40887800	1.79258500	-0.34896500
H	2.19887700	3.10650700	0.84494100
H	2.00438600	1.40600500	1.33820200
B	-0.50981300	0.58354100	2.04733500
H	0.52796300	0.42574500	2.61667900
H	-1.52407700	0.18756500	2.53857200

1a-Int-1'

C	2.84667800	-1.31053200	1.78975400
C	1.63753600	-0.94833500	1.18841700
C	1.24109900	-1.50467300	-0.04191800
C	2.10147700	-2.44098100	-0.64360800
C	3.31159500	-2.80651200	-0.04595000
C	3.69350100	-2.24056500	1.17602400
H	3.13011800	-0.85795900	2.73762800
H	1.01082000	-0.20581500	1.67590400
H	1.82022500	-2.88070500	-1.59877600
H	3.95597400	-3.53329200	-0.53600300
H	4.63509400	-2.51995300	1.64265800
C	-0.00903500	-1.06957800	-0.76606800
H	-0.25139800	-1.78129000	-1.56347100
C	-1.18701300	-0.83665300	0.06781800
H	-1.08767300	-0.45214200	1.07786700
C	-2.52673300	-0.97138700	-0.44030700
O	-3.46006700	-0.57282000	0.47706700

C	-4.84765500	-0.66947400	0.08021300
H	-5.07740100	-1.71407100	-0.15949200
H	-5.00095800	-0.07073800	-0.82481800
C	-5.68907000	-0.16261000	1.23510100
H	-6.75107300	-0.22363700	0.96838500
H	-5.52454300	-0.76593800	2.13561500
H	-5.45079100	0.88251800	1.46519000
O	-2.84426500	-1.39565300	-1.55745000
C	2.16684300	2.92990200	0.46931200
C	0.87781200	3.26920300	0.74450200
H	3.10140300	3.33693300	0.82484000
H	0.46147200	4.02985600	1.38794700
C	0.83394000	1.55933200	-0.72467400
N	2.12069300	1.88102200	-0.43008000
N	0.07559100	2.42201400	0.00120400
C	3.30942600	1.24893800	-1.00557700
H	4.18465400	1.62897100	-0.47658200
H	3.25350500	0.16757500	-0.88433100
H	3.38735400	1.49665000	-2.06720100
C	-1.38574000	2.46379700	0.04694200
H	-1.74634700	1.96949800	0.95243100
H	-1.70663600	3.50818300	0.04734600
H	-1.78887400	1.95678300	-0.82828500
B	0.33861200	0.36571200	-1.67708500
H	-0.66681200	0.69502500	-2.27631700
H	1.23152800	0.07228700	-2.44979200

1a-TS-2’

C	1.25431100	-2.55900600	-1.77634400
C	0.47624900	-1.55568300	-1.18974500
C	0.70155900	-1.14523300	0.13558000
C	1.73137200	-1.78028200	0.85292200
C	2.51152300	-2.78120900	0.27222800
C	2.27681900	-3.17894600	-1.04988600
H	1.05956700	-2.85538000	-2.80486000
H	-0.31243400	-1.09488000	-1.77755100
H	1.92366200	-1.47676500	1.87970500
H	3.30801800	-3.24609300	0.84901100
H	2.88361200	-3.95678100	-1.50726700
C	-0.13742000	-0.10532800	0.82769100
H	0.44169300	0.36294000	1.62968700
C	-0.72969800	0.94108400	-0.02916100
H	-1.31662800	0.63294100	-0.89293000
C	-1.18870800	2.19697400	0.59014000
O	-2.02242700	2.87487500	-0.23324400
C	-2.48766500	4.17255900	0.22321000
H	-1.61700700	4.81065900	0.40885600
H	-3.02519300	4.03806200	1.16792400
C	-3.38338200	4.73917500	-0.85930700
H	-3.75611400	5.72149900	-0.54545600
H	-2.83323600	4.86297100	-1.79933500
H	-4.24416000	4.08461300	-1.03900400
O	-0.82963700	2.62833500	1.68078100
C	-3.15575400	-3.43995200	-0.54695700
C	-3.94486200	-2.35279000	-0.76132500
H	-3.19956800	-4.44277500	-0.94472500
H	-4.81767300	-2.21525300	-1.38204900
C	-2.34931700	-1.75581000	0.71787500
N	-2.18729100	-3.05878600	0.36360800
N	-3.44029300	-1.33260200	0.02418500
C	-1.13669800	-3.96521400	0.82970800
H	-0.29084900	-3.94464600	0.13911500
H	-0.80536300	-3.65839300	1.82013500
H	-1.54939000	-4.97523000	0.87763200
C	-4.02899500	0.00655400	0.05611000
H	-3.73786500	0.56484300	-0.83692800
H	-5.11683400	-0.08996000	0.08896300

H	-3.68188600	0.53335900	0.94287400
B	-1.41730200	-0.88787900	1.69756900
H	-2.07708700	-0.03881500	2.26109300
H	-0.86921200	-1.61610800	2.50197800
S	1.56094800	2.10051800	-1.64510500
H	0.38592000	1.52712100	-0.84169600
C	2.83153600	1.47734000	-0.55635300
C	3.84484700	0.64568400	-1.06376900
C	2.83494300	1.81279100	0.80959800
C	4.84857000	0.16510900	-0.21869800
H	3.83751300	0.36493700	-2.11337500
C	3.82786400	1.30924600	1.65337700
H	2.05290100	2.45203000	1.20977600
C	4.84078700	0.48888500	1.14273300
H	5.62618700	-0.47880400	-0.62242300
H	3.81097800	1.56209100	2.71088100
H	5.61409900	0.10126600	1.80118800

3a’

C	-3.19342700	-0.83001800	-1.69442000
C	-1.88817900	-0.72787300	-1.20287000
C	-1.48939400	-1.40782100	-0.03578100
C	-2.45201500	-2.20757200	0.60871400
C	-3.75941400	-2.31312200	0.12323500
C	-4.14003300	-1.62050500	-1.03223000
H	-3.47238900	-0.28557700	-2.59431700
H	-1.17737300	-0.09166200	-1.72446200
H	-2.17128400	-2.74593600	1.51250600
H	-4.48034200	-2.93727400	0.64732800
H	-5.15595000	-1.69843300	-1.41227700
C	-0.11424700	-1.23428300	0.56409800
H	0.04896400	-2.07083100	1.25601400
C	0.99581500	-1.32907300	-0.50964800
H	1.01026100	-0.45276900	-1.16219200
C	2.36583700	-1.51349700	0.10170800
O	3.28355100	-0.69526900	-0.44801400
C	4.64326700	-0.77392400	0.06152300
H	5.02946600	-1.78150500	-0.12552400
H	4.61877500	-0.60712400	1.14330700
C	5.45273100	0.28737300	-0.65567400
H	6.48810500	0.26132000	-0.29564300
H	5.45913100	0.11228600	-1.73778800
H	5.04494000	1.28670600	-0.46403700
O	2.63917400	-2.32563600	0.97269800
C	-1.72825300	3.13665300	-0.15209200
C	-0.45749200	3.32729700	-0.59745700
H	-2.63521800	3.69633800	-0.32518700
H	-0.03020100	4.08526700	-1.23691200
C	-0.45204100	1.48458800	0.70853600
N	-1.70669000	2.00578200	0.64421100
N	0.31007500	2.30934600	-0.05762100
C	-2.87762700	1.50009000	1.36161500
H	-3.75880200	2.02089300	0.98338600
H	-2.98890500	0.43022200	1.19203500
H	-2.77134900	1.69230300	2.43266800
C	1.74446000	2.18214700	-0.31150900
H	1.91377400	1.82477700	-1.33018700
H	2.21347100	3.16131000	-0.18483200
H	2.17259500	1.47411900	0.39295800
B	-0.01408900	0.14452600	1.49989200
H	1.13755300	0.27887700	1.89514800
H	-0.76161100	0.02933600	2.46272400
H	0.83140800	-2.20667900	-1.15427300

1af

C	-2.45014800	0.14911800	0.00012200
H	-2.51581200	1.23762200	0.00042500

C	-1.23143100	-0.41544300	-0.00021800
H	-1.10632300	-1.49578000	-0.00054300
C	0.00057200	0.39911700	-0.00014100
O	1.09805500	-0.38726400	-0.00019700
C	2.39102200	0.27869700	-0.00008300
H	2.45390500	0.91602600	0.88809000
H	2.45418700	0.91569500	-0.88848200
C	3.45680300	-0.79768900	0.00028700
H	4.44672200	-0.32596500	0.00020900
H	3.37694700	-1.43089000	0.89147900
H	3.37705700	-1.43139900	-0.89055100
O	0.05576500	1.62339200	-0.00003500
C	-3.74123800	-0.60210900	0.00014500
H	-4.34126500	-0.32825500	-0.87912800
H	-3.58893000	-1.68680600	-0.00061200
H	-4.34051900	-0.32942300	0.88029700

1af-TS-1

C	-3.30976000	-0.18088100	-0.14542400
H	-3.44204700	0.10184600	-1.18892200
C	-2.04702700	-0.57274700	0.27603800
H	-1.92919800	-1.00336700	1.26663000
C	-1.01404800	-0.94603900	-0.69638800
O	-0.05163000	-1.71969800	-0.12748200
C	1.06399600	-2.10486700	-0.96588300
H	1.51861900	-1.20249500	-1.38616700
H	0.69145100	-2.71964100	-1.79277700
C	2.04184100	-2.86957100	-0.09644000
H	2.90372700	-3.17829800	-0.70023100
H	2.40328900	-2.24471800	0.72730200
H	1.57545200	-3.76842200	0.32382600
O	-0.99050200	-0.64264500	-1.88889900
C	2.38458800	1.41191900	-0.68133800
C	2.68360200	0.72556600	0.45920900
H	2.99966000	1.68241400	-1.52650800
H	3.60788600	0.28123200	0.79620400
C	0.49526500	1.28563900	0.55394500
N	1.04564900	1.74026800	-0.61883000
N	1.52344900	0.64853300	1.20220800
C	0.31566500	2.47979800	-1.64248000
H	-0.54148300	1.89253000	-1.97768900
H	-0.02829100	3.43860100	-1.24241300
H	0.98770300	2.65745100	-2.48380900
C	1.38991000	-0.02918200	2.48503000
H	0.65626600	-0.83465200	2.40194800
H	2.35932200	-0.44652100	2.76179000
H	1.06681500	0.67973700	3.25297600
B	-0.96849400	1.40367300	1.00360400
H	-1.21276200	1.19344500	2.15817600
H	-1.66241500	2.16555400	0.39361000
C	-4.43039500	0.11030700	0.80124800
H	-4.52384800	1.19556900	0.98399500
H	-5.39873000	-0.22014100	0.40098500
H	-4.27740000	-0.37100200	1.77536000

1af-Int-1

C	3.27256400	0.15663500	-0.10403200
H	3.58691900	0.83375200	-0.89651900
C	1.82127600	0.06449000	0.18671200
H	1.64166300	-0.31642300	1.20012900
C	1.08596800	1.35081000	0.00973200
O	-0.00421100	1.41887500	0.81563700
C	-0.92211300	2.52134500	0.60358600
H	-1.26434200	2.49608300	-0.43664700
H	-0.39051400	3.46439500	0.76924100
C	-2.07196700	2.34538500	1.57510700
H	-2.79458200	3.15895400	1.43836500

H	-2.58561700	1.39298400	1.40224800
H	-1.71727100	2.37009500	2.61223900
O	1.35523600	2.23640700	-0.79488900
C	-2.71839700	-0.91687300	-0.57931500
C	-2.41849200	-1.66378300	0.51804000
H	-3.66305700	-0.56382700	-0.96449700
H	-3.04754400	-2.09020400	1.28502300
C	-0.48225800	-1.19748600	-0.53863400
N	-1.51944300	-0.63677700	-1.21159400
N	-1.04448800	-1.82841600	0.52355300
C	-1.40687800	0.14701700	-2.44268400
H	-2.37713100	0.60319000	-2.64546000
H	-0.65752400	0.92954300	-2.31977500
H	-1.12503500	-0.49968700	-3.27758600
C	-0.31183000	-2.50722800	1.59004400
H	-0.05725200	-1.79541000	2.38074300
H	-0.94286500	-3.29858600	1.99978700
H	0.60162300	-2.94133000	1.18324000
B	1.08360700	-1.06492600	-0.87139000
H	1.62712300	-2.14070300	-0.68770500
H	1.22792800	-0.70093900	-2.02300800
C	4.20197300	-0.93886000	0.30583900
H	4.15666700	-1.80493300	-0.38125900
H	5.24660700	-0.60106700	0.31683400
H	3.95642000	-1.32587000	1.30549000

1af-TS-2

C	-0.05829400	-0.34827500	0.57247900
H	-0.42779900	-0.34835600	1.59829900
C	1.01485000	0.63264400	0.25746800
H	1.12865900	0.74334800	-0.82684300
C	0.76560700	1.97984900	0.86097600
O	1.17565200	2.98145300	0.04892700
C	1.07746500	4.33487500	0.56553000
H	1.67014400	4.40375700	1.48419200
H	0.03115700	4.54172100	0.81394600
C	1.59519500	5.26950600	-0.50926600
H	1.53412500	6.30478200	-0.15266300
H	2.64148800	5.04929200	-0.75129600
H	0.99778800	5.18478100	-1.42459500
O	0.28676600	2.18880800	1.96901100
C	3.79987800	-2.56166600	-1.48063400
C	3.65014100	-3.28808600	-0.33917900
H	4.13304700	-2.84383800	-2.46819000
H	3.82895400	-4.33212400	-0.13032300
C	3.05944600	-1.16387000	0.12481500
N	3.43847400	-1.26200900	-1.17474100
N	3.19645200	-2.41447700	0.63364900
C	3.42810100	-0.17030000	-2.14659000
H	2.47088100	-0.14555200	-2.67503400
H	3.58398100	0.77610200	-1.62838400
H	4.23617800	-0.33204100	-2.86283600
C	2.90308500	-2.80360600	2.01425000
H	1.86644500	-2.56264600	2.25773600
H	3.05852700	-3.87980300	2.10476000
H	3.57015900	-2.27935000	2.70187700
B	2.52693900	0.14671300	0.88341600
H	2.42009600	-0.08050800	2.07112700
H	3.31887300	1.05853400	0.69218000
C	-0.15001700	-1.64289200	-0.17811100
H	0.54371300	-2.39769400	0.22515200
H	-1.15782800	-2.07063400	-0.10575000
H	0.09454400	-1.51032100	-1.23958700
S	-2.45649100	1.11969500	-0.85073300
H	-1.37983600	0.48313700	-0.12934500
C	-3.71851800	-0.06480000	-0.38956400
C	-3.68993500	-0.72896100	0.84940600

C	-4.77905100	-0.31428100	-1.27710100
C	-4.69930100	-1.63650100	1.18321400
H	-2.88287500	-0.53870600	1.55107300
C	-5.79523700	-1.20914100	-0.92696600
H	-4.80897900	0.18548600	-2.24229800
C	-5.75859600	-1.87902500	0.30090000
H	-4.65888500	-2.14853000	2.14188300
H	-6.61083100	-1.38888600	-1.62351200
H	-6.54531200	-2.58015300	0.56744500

3af-α

C	3.30006000	0.12838900	-0.14603100
H	3.45747800	0.30634800	-1.21817600
C	1.78962700	0.01270800	0.14021300
H	1.63865600	-0.33404000	1.17164800
C	1.10371700	1.33008500	0.00205800
O	0.00578500	1.41872800	0.79903800
C	-0.85723800	2.56875100	0.62008900
H	-1.16763000	2.61554300	-0.42936000
H	-0.29236300	3.47818600	0.85211600
C	-2.04381600	2.39210800	1.54665900
H	-2.72696500	3.24274700	1.43471000
H	-2.59129700	1.47364900	1.30658500
H	-1.72123200	2.34450900	2.59348300
O	1.41628900	2.23533600	-0.76535400
C	-2.78830300	-0.89859000	-0.54392000
C	-2.47498300	-1.65496600	0.54284000
H	-3.73738000	-0.54197400	-0.91466800
H	-3.09425900	-2.08824800	1.31392900
C	-0.55123500	-1.17895800	-0.53351400
N	-1.59633800	-0.61344200	-1.18847200
N	-1.10059100	-1.81967200	0.52898300
C	-1.49946900	0.18380700	-2.41193900
H	-2.46989100	0.64876200	-2.59292700
H	-0.74278100	0.95995700	-2.29328100
H	-1.23598700	-0.45434400	-3.25933800
C	-0.35246300	-2.50847100	1.57794700
H	-0.05845500	-1.79847900	2.35648900
H	-0.98891500	-3.28294900	2.01083200
H	0.53901400	-2.96545300	1.14759800
B	1.01530800	-1.06204800	-0.88926600
H	1.51597300	-2.16896600	-0.75942100
H	1.13619400	-0.68765700	-2.04488200
C	4.09727500	-1.10532200	0.29205100
H	3.79005800	-2.00383300	-0.25459600
H	5.17121800	-0.95817600	0.11787300
H	3.95776200	-1.30338900	1.36389200
H	3.70713700	1.01008200	0.37206600

1af-TS-1’

C	-1.13295400	2.21443000	-0.35472600
H	-1.52204200	1.73863200	-1.25247300
C	0.18963000	2.00349400	-0.04569600
H	0.64836500	2.51558800	0.79698100
C	0.98880200	1.00612100	-0.72777900
O	2.21213000	0.84721000	-0.13415300
C	3.09868700	-0.13288900	-0.71908100
H	3.35638100	0.18173100	-1.73752200
H	2.57660000	-1.09321300	-0.78086700
C	4.32741400	-0.22189900	0.16508800
H	5.02772700	-0.95569300	-0.25203200
H	4.83903800	0.74579700	0.22799300
H	4.05721300	-0.54062700	1.17886500
O	0.65835500	0.35461900	-1.72271200
C	0.13389300	-2.24308400	0.57416600
C	-0.68677700	-2.54359500	-0.47273800
H	1.04647300	-2.70870200	0.91443600

H	-0.62061100	-3.31683700	-1.22353200
C	-1.55952600	-0.74627600	0.58203000
N	-0.39975700	-1.13727500	1.20432100
N	-1.71607700	-1.62746000	-0.45853600
C	0.13561500	-0.54156200	2.42344300
H	-0.48904300	-0.81105300	3.28201100
H	1.14823700	-0.91869000	2.57630200
H	0.16658400	0.54371800	2.32462600
C	-2.78207800	-1.55247800	-1.44800600
H	-2.68438700	-2.39995100	-2.12845200
H	-3.75697700	-1.59218100	-0.95441800
H	-2.70317500	-0.61979400	-2.01447500
B	-2.41717100	0.47492100	0.90776500
H	-3.42916100	0.64540800	0.28979800
H	-2.25432800	1.03430800	1.95362900
C	-1.90663200	3.38942900	0.18310200
H	-1.75318700	4.26441400	-0.46893700
H	-2.98323700	3.19492200	0.21445900
H	-1.57410200	3.66599000	1.19039700

1af-Int-1’

C	-0.95851700	2.10845100	0.04648100
H	-1.22091500	1.89496900	1.09108800
C	0.45721800	1.81828800	-0.17855900
H	0.95623600	2.22963400	-1.05383500
C	1.18970300	0.85716500	0.60226700
O	2.43043100	0.60307200	0.08018500
C	3.23343800	-0.38449500	0.76710200
H	2.67520300	-1.32673000	0.80974500
H	3.41146000	-0.04777900	1.79487700
C	4.52936300	-0.53700400	-0.00469000
H	5.16399600	-1.28247400	0.48953900
H	4.33755100	-0.87308500	-1.03050600
H	5.07807800	0.41131700	-0.04369400
O	0.78371600	0.29376300	1.62632500
C	-1.56687500	-2.44873600	0.60224800
C	-0.66083000	-2.55912700	-0.40716700
H	-1.87539500	-3.14987900	1.36305600
H	-0.01942200	-3.37519000	-0.70438000
C	-1.56340900	-0.49629600	-0.52427900
N	-2.10900700	-1.17996500	0.51303500
N	-0.67522200	-1.35455500	-1.08958900
C	-3.11797100	-0.64671300	1.42677300
H	-3.31962100	-1.39879500	2.19097100
H	-2.74580000	0.26347000	1.90127300
H	-4.03805900	-0.42322800	0.88162100
C	0.21017200	-1.06166000	-2.21642800
H	1.15535400	-0.64788300	-1.85418800
H	0.40212200	-1.99048200	-2.75759700
H	-0.27020800	-0.34367800	-2.88002000
B	-1.86868600	1.03258700	-0.91259100
H	-1.61577300	1.22699900	-2.08957900
H	-3.04602600	1.26924500	-0.68298800
C	-1.34604800	3.55052600	-0.31859900
H	-2.42252800	3.71061300	-0.18165800
H	-0.81372400	4.28641900	0.30131000
H	-1.11012600	3.76817100	-1.36915800

1af-TS-2’

C	1.38064900	-2.15819700	0.30844700
H	1.46317800	-2.23551700	-0.78347100
C	0.30896200	-1.19879700	0.65694600
H	0.06607600	-1.09112500	1.71530600
C	0.14938100	0.03191300	-0.13736700
O	-0.43012200	1.02341300	0.57828600
C	-0.63736700	2.28734300	-0.10360200
H	-1.25701800	2.10993500	-0.98766900

H	0.33642000	2.66720600	-0.43117500
C	-1.30988900	3.22551300	0.87700700
H	-1.48593300	4.19311500	0.39196100
H	-2.27519800	2.82266400	1.20294800
H	-0.67962700	3.39060700	1.75868400
O	0.47728600	0.15916100	-1.31379100
C	3.37422500	2.13031300	-0.05052700
C	3.74974500	1.52317400	-1.20962500
H	3.32312600	3.17289000	0.22483300
H	4.08421300	1.93063900	-2.15185300
C	3.19077800	-0.09574200	0.25417100
N	3.03109700	1.12228600	0.83363600
N	3.63555000	0.16144100	-1.00107900
C	2.58520700	1.35592300	2.20806800
H	2.40120800	2.42456500	2.32878400
H	1.66215700	0.80792900	2.39735300
H	3.35512100	1.03372200	2.91357300
C	3.84112900	-0.84239300	-2.04181600
H	2.88689000	-1.07620400	-2.52179200
H	4.53694100	-0.44085900	-2.78110700
H	4.25895600	-1.74715400	-1.59906600
B	2.85355800	-1.53204600	0.88854300
H	3.71716800	-2.33029000	0.55219400
H	2.80379900	-1.44032300	2.10248400
C	1.15768600	-3.54654200	0.92176900
H	0.24812500	-4.02657900	0.53208700
H	2.00244000	-4.20969900	0.70124100
H	1.05816400	-3.48495700	2.01379600
S	-2.33800400	-2.51226600	-0.07990700
H	-0.99629000	-1.89381600	0.26347900
C	-3.22583000	-0.96285800	-0.22526200
C	-3.16608300	-0.21724500	-1.41512400
C	-3.99534200	-0.48356500	0.84951400
C	-3.85697300	0.99294700	-1.52273700
H	-2.57186500	-0.58052300	-2.24861600
C	-4.68877500	0.72458200	0.73473800
H	-4.04361500	-1.05355600	1.77330400
C	-4.61914000	1.46755600	-0.44944700
H	-3.79608700	1.56569000	-2.44489700
H	-5.27832700	1.08765000	1.57314800
H	-5.15376500	2.41025000	-0.53450600

3af-β

C	2.36710600	0.61250200	-0.16985600
H	2.55061200	0.30666900	0.87141000
C	1.40519100	1.82587300	-0.11054400
H	1.10286000	2.12732300	-1.11945700
C	0.17907600	1.56338300	0.72930100
O	-0.97044900	1.76199500	0.05482300
C	-2.21225800	1.52762300	0.77660800
H	-2.28394000	2.26036000	1.58761800
H	-2.17615400	0.52641200	1.21415300
C	-3.34823900	1.66891000	-0.21526900
H	-4.30221600	1.50715300	0.30074300
H	-3.36219200	2.67104800	-0.65935200
H	-3.26089300	0.92986500	-1.01907800
O	0.20635900	1.21586600	1.90195200
C	-1.76086100	-1.90971200	-0.29468200
C	-1.11191000	-2.31920100	0.82907000
H	-2.79144700	-2.01074600	-0.59953600
H	-1.46212000	-2.84780200	1.70302000
C	0.39610200	-1.27234200	-0.48511000
N	-0.82255900	-1.26754400	-1.08524500
N	0.20681700	-1.92265400	0.69218400
C	-1.12341000	-0.66950300	-2.38571300
H	-2.18946600	-0.79547300	-2.57950900
H	-0.88018700	0.39377900	-2.36977400

H	-0.54975500	-1.16692800	-3.17068300
C	1.22755600	-2.10994900	1.71992100
H	1.27721500	-1.22208300	2.35579500
H	0.96315400	-2.98227100	2.32116100
H	2.19525200	-2.27431800	1.24387800
B	1.78190100	-0.65682200	-1.04190600
H	2.60701300	-1.57253500	-1.01673600
H	1.60548800	-0.31552200	-2.20561600
C	3.71116700	1.07864100	-0.75482200
H	4.15422800	1.90470700	-0.17670700
H	4.43969200	0.25712700	-0.77343200
H	3.59102900	1.43075300	-1.78997000
H	1.90812600	2.68773400	0.35540300

1ai

C	-2.15344800	-0.24337300	0.00072000
C	-0.85799000	-0.63577200	-0.00199300
H	-0.62029300	-1.69635900	-0.00404200
C	0.29781200	0.27003600	-0.00219600
O	1.45644600	-0.43608500	-0.00002000
C	2.69213300	0.32574400	-0.00013800
H	2.70799400	0.96842500	0.88665700
H	2.70961000	0.96547400	-0.88902200
C	3.83826000	-0.66566600	0.00260700
H	4.78970200	-0.12041100	0.00256800
H	3.80544600	-1.30218700	0.89451400
H	3.80709900	-1.30522500	-0.88719800
O	0.27528000	1.49685600	-0.00394600
C	-3.25046900	-1.27168000	-0.00036900
H	-3.89725800	-1.12929700	-0.87784000
H	-2.86898300	-2.29771200	-0.00523600
H	-3.89275600	-1.13601200	0.88144500
C	-2.60728600	1.19562200	0.00417700
H	-2.21276100	1.73199300	0.87454700
H	-2.23551600	1.72778700	-0.87897500
H	-3.70016600	1.25789600	0.01746600

1ai-TS-1

C	-3.06888400	-0.37099900	0.22087000
C	-1.73808000	-0.74783700	0.41245400
H	-1.51103200	-1.30871300	1.31449500
C	-0.75551300	-0.95503600	-0.65737700
O	0.25823000	-1.75846300	-0.22524600
C	1.34391700	-1.99758500	-1.15086500
H	1.73411700	-1.03575700	-1.49740900
H	0.95699400	-2.54396300	-2.01853400
C	2.40219200	-2.79565500	-0.41529400
H	3.23760500	-3.00709300	-1.09368700
H	2.78823100	-2.23607800	0.44355000
H	1.99822500	-3.75041500	-0.05831100
O	-0.77954300	-0.51152700	-1.80481200
C	2.42718200	1.62970500	-0.69950600
C	2.88558000	0.85022800	0.32194300
H	2.93340400	2.03320700	-1.56368200
H	3.86620700	0.44215500	0.51549400
C	0.68677200	1.23386100	0.69000600
N	1.08339200	1.85006500	-0.47212000
N	1.81591700	0.60787900	1.15963400
C	0.20623400	2.64345100	-1.32404700
H	0.76416800	2.94119200	-2.21371300
H	-0.65758300	2.04408600	-1.61898400
H	-0.13123700	3.53760600	-0.79029900
C	1.86154200	-0.20105700	2.36947700
H	2.87382500	-0.59020400	2.48952900
H	1.60044800	0.40786500	3.24045800
H	1.15854800	-1.03320200	2.28424400
B	-0.72708000	1.18622000	1.28296500

H	-0.83856600	0.82270100	2.42023400
H	-1.52576700	1.96275200	0.84101000
C	-3.98654700	-0.34927000	1.41127000
H	-4.14447600	0.68858900	1.75579800
H	-4.98137600	-0.74419500	1.16029700
H	-3.58998200	-0.92159800	2.25775900
C	-3.57703900	0.33862500	-1.00008500
H	-3.05625100	0.03953800	-1.91184600
H	-4.65503100	0.17429400	-1.12698900
H	-3.43386600	1.42985100	-0.88944100

1ai-Int-1

C	3.04033000	-0.16404300	0.19205300
C	1.55033700	-0.07928200	0.32007800
H	1.24139400	-0.48977200	1.28803500
C	0.94634000	1.28464700	0.20427900
O	-0.20792200	1.36402300	0.91993500
C	-1.00987400	2.56055000	0.76125800
H	-1.24186900	2.69079100	-0.30131800
H	-0.42773900	3.42645900	1.09456600
C	-2.26615300	2.37533400	1.58897700
H	-2.90513700	3.26105700	1.48883000
H	-2.83075000	1.49989700	1.24891400
H	-2.02208400	2.24458900	2.64987600
O	1.34771300	2.22599800	-0.47055700
C	-2.99679700	-0.75714700	-0.69661300
C	-2.77311100	-1.61880900	0.33272400
H	-3.90917900	-0.32563200	-1.07969600
H	-3.44990800	-2.08763000	1.03126800
C	-0.77744100	-1.14190500	-0.60315400
N	-1.76252700	-0.47331100	-1.25529100
N	-1.40821800	-1.84591900	0.37138500
C	-1.56826100	0.41659400	-2.40079400
H	-2.50957700	0.93401100	-2.59271800
H	-0.79138200	1.14874800	-2.17855500
H	-1.28171800	-0.16104600	-3.28344900
C	-0.74654000	-2.65805100	1.39000800
H	-0.48579200	-2.03754900	2.25253800
H	-1.42802900	-3.45145100	1.70334100
H	0.15913200	-3.09747300	0.97165400
B	0.80366300	-1.05561700	-0.86704700
H	1.28687700	-2.17312400	-0.77371400
H	1.00645000	-0.59389700	-1.97303500
C	3.72143600	-1.24549000	0.97893600
H	3.61094000	-2.23314000	0.48824700
H	4.80141000	-1.06228900	1.06596600
H	3.30782200	-1.34851400	1.99107900
C	3.73637800	0.27880300	-1.06165600
H	4.78837600	0.53023200	-0.86394900
H	3.74904900	-0.53253900	-1.81596300
H	3.25042200	1.14359200	-1.52094800

1ai-TS-2

C	-0.34113400	-2.56662800	0.29534900
C	0.52920600	-1.39233700	-0.03418900
H	0.52130800	-1.24592500	-1.11946800
C	0.17589900	-0.08476600	0.60501900
O	0.25993400	0.92965500	-0.28996600
C	0.10265000	2.27734000	0.22205300
H	0.86626500	2.45237300	0.98794200
H	-0.88321100	2.36423700	0.68791300
C	0.25622900	3.22506400	-0.95004300
H	0.14151300	4.25873600	-0.60209200
H	1.24636300	3.12273600	-1.40935200
H	-0.50707900	3.03159200	-1.71236000
O	-0.07033600	0.11555100	1.78789400
C	4.09513400	1.58403100	0.22990300

C	4.02887600	1.27591000	-1.09425800
H	4.52660700	2.42948400	0.74427900
H	4.38532700	1.80084700	-1.96789900
C	3.02822000	-0.39241800	0.04401200
N	3.47328200	0.55233000	0.91163600
N	3.37559600	0.06001700	-1.18749400
C	3.34156000	0.49489600	2.36944000
H	3.95061200	-0.31959000	2.76927800
H	3.68961000	1.44478100	2.77816000
H	2.29660700	0.34070100	2.64044100
C	3.02857700	-0.58000300	-2.45446800
H	2.06678600	-0.20176600	-2.81242100
H	3.80646200	-0.35342900	-3.18640000
H	2.96493000	-1.65852800	-2.30968700
B	2.15769300	-1.69920900	0.38804400
H	2.54615100	-2.63863600	-0.28281000
H	2.23862700	-1.94600300	1.57415100
C	-0.15890100	-3.77794300	-0.58279500
H	0.69228600	-4.38616500	-0.23371100
H	-1.04561800	-4.42526300	-0.55482300
H	0.03971600	-3.50377300	-1.62608900
C	-0.77733800	-2.85800100	1.70478400
H	-1.58967600	-3.59599600	1.71166000
H	0.05955100	-3.29178100	2.28077300
H	-1.10295500	-1.96116000	2.23560400
S	-2.96381300	-1.68730600	-1.22908600
H	-1.80868900	-2.05591300	-0.44384800
C	-3.26562200	-0.09059800	-0.46817200
C	-3.38204800	1.05145600	-1.27769600
C	-3.43005800	0.03242700	0.92132900
C	-3.66661300	2.29513600	-0.70489200
H	-3.24035800	0.96954200	-2.35214200
C	-3.69876300	1.28066400	1.48953200
H	-3.34048800	-0.84474100	1.55583100
C	-3.82368600	2.41556800	0.68009400
H	-3.75192200	3.17137200	-1.34315800
H	-3.81254400	1.36381500	2.56788200
H	-4.03570900	3.38452100	1.12507300

3ai

C	3.05250000	-0.06857600	0.21592300
C	1.50071800	-0.02552800	0.26325700
H	1.17978400	-0.46461100	1.21744100
C	0.96937800	1.37121700	0.24325600
O	-0.26630500	1.44597600	0.80836300
C	-0.95215000	2.72032500	0.75193300
H	-0.97015000	3.07065100	-0.28542000
H	-0.39349600	3.45001600	1.34894900
C	-2.35206200	2.50478900	1.29243100
H	-2.90416400	3.45217100	1.27007000
H	-2.89535700	1.77177000	0.68576700
H	-2.32304100	2.14684600	2.32833500
O	1.51107000	2.35918300	-0.24168500
C	-3.06326800	-0.95898000	-0.48536300
C	-2.68353700	-1.87421600	0.44718600
H	-4.03807700	-0.58919900	-0.76561000
H	-3.25882000	-2.46376300	1.14528800
C	-0.81715800	-1.13057400	-0.57909000
N	-1.90605500	-0.51481700	-1.10263400
N	-1.30406200	-1.96981700	0.37045400
C	-1.87852600	0.49363200	-2.16109000
H	-2.88688400	0.89380800	-2.27645200
H	-1.19678400	1.30084800	-1.89064100
H	-1.55413300	0.04474100	-3.10299000
C	-0.49544200	-2.80776000	1.25321100
H	-0.24144100	-2.25834500	2.16458400
H	-1.06881800	-3.70016500	1.51286900

H	0.41998300	-3.09830400	0.73763500
B	0.72966400	-0.88746500	-0.95508200
H	1.26601900	-1.98032100	-1.07015000
H	0.78452200	-0.29441900	-2.01844000
C	3.57357200	-1.38396600	0.81560400
H	3.24043800	-2.24823000	0.22738500
H	4.67166400	-1.39585000	0.83257400
H	3.22042900	-1.52258800	1.84625200
C	3.64393100	0.14733400	-1.18575900
H	4.73928200	0.21498800	-1.13241500
H	3.39445200	-0.68683500	-1.85304700
H	3.26644300	1.07030600	-1.63732600
H	3.42478300	0.74879100	0.85360300

1ai-TS-1'

C	0.96027500	-2.12422200	0.03474500
C	-0.37870500	-1.79835500	0.22002900
H	-0.86357500	-2.13592600	1.13281700
C	-1.17620500	-0.92941800	-0.61033300
O	-2.41395900	-0.71498500	-0.04469700
C	-3.30926800	0.16115100	-0.75904500
H	-3.50822900	-0.25690300	-1.75307400
H	-2.82621300	1.13566900	-0.89365800
C	-4.58201500	0.28049100	0.05767600
H	-5.28930700	0.94180700	-0.45758100
H	-5.05831700	-0.69845600	0.18849700
H	-4.37470900	0.70240900	1.04824900
O	-0.87444000	-0.41210500	-1.69252400
C	-0.02185000	2.51872600	0.17558700
C	1.02599600	2.61772700	-0.69121500
H	-0.92781300	3.09963400	0.26115700
H	1.20910700	3.30284100	-1.50545900
C	1.44983800	0.88521400	0.69442900
N	0.24251900	1.45363000	1.01183600
N	1.91575600	1.61385300	-0.37035600
C	-0.57870300	1.09538500	2.16459400
H	-1.56888200	1.53376500	2.03232700
H	-0.67209000	0.01308200	2.23515600
H	-0.12487100	1.48477300	3.08244000
C	3.21841100	1.43323100	-1.00413000
H	3.25511700	2.05652400	-1.89915400
H	4.01501900	1.73499000	-0.31641100
H	3.36071800	0.38944200	-1.28420800
B	2.07060400	-0.37115000	1.31770700
H	3.17375000	-0.68499100	0.97476800
H	1.65560800	-0.74199700	2.37766500
C	1.52644800	-3.25105700	0.87721100
H	1.35508400	-4.20965600	0.35948400
H	2.60758800	-3.15256000	1.02055100
H	1.04817200	-3.31285500	1.86052100
C	1.67197200	-1.91429400	-1.28473000
H	1.33400000	-2.67805100	-2.00489700
H	1.45493000	-0.94027200	-1.72304300
H	2.75401100	-2.03064400	-1.16617600

1ai-Int-1'

C	0.87670400	-2.00476900	0.21910800
C	-0.55553300	-1.67729500	0.29622200
H	-1.10279500	-2.03027300	1.16805500
C	-1.28414100	-0.76281800	-0.54542100
O	-2.56304000	-0.57701100	-0.07923200
C	-3.38345800	0.36981800	-0.79861800
H	-3.50296300	0.02795100	-1.83329600
H	-2.87206200	1.33918800	-0.81836800
C	-4.71644800	0.45680900	-0.08109800
H	-5.36709900	1.17016500	-0.60129000
H	-5.21796700	-0.51798600	-0.06368100

H	-4.58370400	0.80051900	0.95157300
O	-0.87582400	-0.17594200	-1.55420300
C	0.57794900	2.74671700	0.26693600
C	1.62104300	2.60644400	-0.59433600
H	-0.10124000	3.56881300	0.43591500
H	2.03535400	3.27939100	-1.33020900
C	1.45458900	0.69280400	0.58880900
N	0.48984100	1.56663500	0.98267100
N	2.14787600	1.34488600	-0.38047400
C	-0.46605000	1.35539300	2.07049000
H	-1.27338700	2.08165600	1.96114100
H	-0.87853000	0.34929500	2.02007600
H	0.02774500	1.49817400	3.03600200
C	3.29485100	0.83418800	-1.12950400
H	3.00348800	0.63359200	-2.16384100
H	4.08979900	1.58452200	-1.11341200
H	3.64822900	-0.08414200	-0.66552000
B	1.67696100	-0.79984800	1.14732000
H	2.86463200	-1.08246200	1.10187500
H	1.27868600	-0.84558300	2.29835900
C	1.14727100	-3.34718900	0.93152600
H	0.68419100	-4.18456500	0.38743500
H	2.22530700	-3.54242400	0.98959700
H	0.75095100	-3.35183200	1.95485700
C	1.45608700	-2.06530000	-1.20667100
H	0.95621500	-2.84843400	-1.79733800
H	1.33547100	-1.12295000	-1.74235400
H	2.52459300	-2.30919300	-1.16472300

1ai-TS-2’

C	1.35877700	-1.99469500	0.49826300
C	0.33952100	-0.99729800	0.92311200
H	0.24527400	-0.86041100	2.00128200
C	0.01457900	0.22554000	0.16578700
O	-0.63521400	1.11671400	0.95702900
C	-1.12322500	2.32291000	0.31763800
H	-1.82352600	2.03658700	-0.47317800
H	-0.27702300	2.84606500	-0.14025500
C	-1.79165900	3.16359100	1.38597900
H	-2.18848300	4.07994600	0.93282200
H	-2.62301200	2.62033000	1.84843200
H	-1.07699400	3.44615100	2.16794100
O	0.24303800	0.44150700	-1.02000900
C	3.28541100	2.33602700	-0.23154500
C	3.68012600	1.71732300	-1.37679500
H	3.20020100	3.38232900	0.02094300
H	4.00567800	2.11418900	-2.32675900
C	3.16870500	0.11024800	0.12026900
N	2.97594100	1.33804500	0.67433100
N	3.60772300	0.35641800	-1.14082800
C	2.58687400	1.60147900	2.06101600
H	1.75366900	0.96302000	2.34841800
H	3.43343100	1.41808000	2.72837100
H	2.28058000	2.64607600	2.13568900
C	3.95231500	-0.63854000	-2.15480600
H	3.17824300	-0.66590800	-2.92628000
H	4.90873200	-0.36401500	-2.60713400
H	4.03566300	-1.61714700	-1.68634900
B	2.89872000	-1.30595200	0.83299800
H	3.70964600	-2.12666200	0.43361300
H	3.00592000	-1.15359800	2.03624100
C	1.25360200	-3.24487800	1.39474300
H	0.31368500	-3.78693700	1.20909800
H	2.07881700	-3.93725900	1.18873600
H	1.28806200	-2.98713100	2.46056300
C	1.24579900	-2.42438500	-0.97385200
H	1.35604700	-1.58377800	-1.65946500

H	2.01634700	-3.16907300	-1.20658600
H	0.26441600	-2.88344800	-1.16711400
S	-2.38957900	-2.33245900	0.65673900
H	-1.00313000	-1.70055100	0.75657800
C	-3.19740300	-0.94014500	-0.12870300
C	-3.03986900	-0.70732400	-1.50637000
C	-4.01652100	-0.07998400	0.62352100
C	-3.68828200	0.36920600	-2.11764300
H	-2.40458900	-1.36427500	-2.09351300
C	-4.66965100	0.99017200	0.00607400
H	-4.13398800	-0.24624500	1.69077400
C	-4.50729600	1.21877700	-1.36507300
H	-3.55295700	0.54284200	-3.18251200
H	-5.29851900	1.64968200	0.59911200
H	-5.01159400	2.05507900	-1.84265000

3ai’

C	0.26725300	-0.37589000	-0.16814700
C	1.48991800	-0.66689400	-1.09943300
H	1.59759300	0.15302800	-1.81744300
C	2.80001200	-0.84709200	-0.37211100
O	3.51316800	0.29741800	-0.30860100
C	4.76057900	0.26360300	0.43609400
H	5.42178100	-0.47996900	-0.02134300
H	4.54532800	-0.05123200	1.46269100
C	5.35674500	1.65579700	0.38702800
H	6.30179600	1.66709100	0.94321600
H	5.56102900	1.95990300	-0.64618900
H	4.68001400	2.38865800	0.84151400
O	3.18807200	-1.89173900	0.13444600
C	-4.14777900	1.18461200	0.58759000
C	-4.37082300	-0.15279900	0.68812500
H	-4.72635500	2.03107400	0.92605200
H	-5.18000000	-0.71021200	1.13587300
C	-2.41744400	0.13018000	-0.41182100
N	-2.94802600	1.33962700	-0.08556100
N	-3.30429300	-0.78226700	0.06846900
C	-2.40105600	2.64190500	-0.46918300
H	-2.94940000	3.41395800	0.07326900
H	-1.34495400	2.69893800	-0.21070100
H	-2.52181500	2.79631000	-1.54484100
C	-3.19728500	-2.23719000	-0.02890800
H	-2.91502000	-2.65801600	0.93971400
H	-4.16570900	-2.64127500	-0.33436300
H	-2.44311800	-2.49296000	-0.77042900
B	-1.02428900	-0.13695900	-1.18708600
H	-1.17082700	-1.12792300	-1.89738900
H	-0.81063000	0.83639600	-1.90222400
C	0.03348000	-1.57038400	0.77639000
H	0.89279700	-1.74971300	1.43843300
H	-0.83853400	-1.38861500	1.41761400
H	-0.15156200	-2.49677400	0.21767000
C	0.54972100	0.87238900	0.69079100
H	1.38556900	0.71712700	1.39016600
H	0.79965100	1.74193600	0.06964500
H	-0.33015400	1.13236600	1.29417100
H	1.30033900	-1.59167400	-1.65528100

1ag

C	-5.12020800	-2.20396400	-0.38023200
H	-5.93342700	-2.13992100	0.35648700
H	-5.44249000	-1.71577100	-1.30608100
H	-4.96611300	-3.27418900	-0.57769900
C	-3.87430900	-1.59720300	0.17524100
H	-3.48634400	-2.02903200	1.09814600
C	-3.20528100	-0.57008600	-0.37458000
H	-3.55172600	-0.10388600	-1.29548500

C	-1.97671000	-0.03632900	0.25397600
O	-1.46679000	-0.46026600	1.28903800
N	-1.41412700	1.02944900	-0.44560700
H	-1.78941300	1.30726300	-1.35123800
S	0.02668500	1.83147200	-0.01851800
O	-0.10445600	2.37185700	1.33853600
O	0.23722500	2.74736900	-1.15243200
C	1.28411900	0.56714000	-0.03291700
C	1.73002700	0.07815900	-1.26576500
C	1.79638800	0.08864400	1.17367900
C	2.71153400	-0.91032400	-1.27779500
H	1.31958400	0.45908300	-2.19624900
C	2.78452000	-0.89631500	1.13795800
H	1.42678700	0.47269400	2.11786100
C	3.25678200	-1.40899600	-0.08079700
H	3.06360800	-1.29858900	-2.23038600
H	3.19020300	-1.27437000	2.07301300
C	4.34620800	-2.45064900	-0.11236800
H	5.32333600	-1.97666700	-0.27961900
H	4.40257200	-3.00058000	0.83296400
H	4.18911900	-3.16787400	-0.92572200

1ag-TS-1

C	1.74940500	4.66008700	-0.16306200
H	2.61017900	4.91218100	0.48037900
H	2.13847400	4.47763600	-1.17235400
H	1.11409600	5.55607900	-0.19403500
C	1.00970300	3.47575700	0.37205100
H	0.45471200	3.60046100	1.30043700
C	1.15775600	2.19931000	-0.13944700
H	1.58854600	2.07321600	-1.12989500
C	0.33599300	1.09351600	0.36800800
O	-0.30897700	1.09764100	1.42037500
N	0.28750400	-0.02416500	-0.48493800
H	0.74557900	0.00233100	-1.39308500
S	-0.70267800	-1.37309300	-0.26333800
O	-0.39751800	-2.02827000	1.01331300
O	-0.52760600	-2.12498800	-1.52045300
C	-2.36509700	-0.72102800	-0.19454300
C	-2.92301500	-0.18099200	-1.35917800
C	-3.07708400	-0.76244400	1.00307700
C	-4.21983600	0.32341000	-1.31048600
H	-2.35583300	-0.15227100	-2.28502700
C	-4.37937300	-0.25707000	1.02930800
H	-2.62194600	-1.17421100	1.89721700
C	-4.96885400	0.29177900	-0.11915500
H	-4.66131700	0.74688200	-2.20964800
H	-4.94132300	-0.28692600	1.95946200
C	-6.37635000	0.83276700	-0.09438100
H	-7.03350900	0.23337800	-0.73811200
H	-6.79257700	0.82449200	0.91812300
H	-6.40739700	1.86211000	-0.47266700
C	3.40490900	-1.93317700	-0.98411700
H	3.61870000	-2.49131900	-1.88338300
C	2.93092200	-2.33768800	0.22907900
H	2.64585200	-3.31550800	0.58615500
C	2.35290800	-1.20314300	2.40112000
H	1.44673200	-0.60004300	2.47477000
H	2.12836100	-2.22847000	2.69667700
H	3.12538800	-0.79330400	3.05822700
C	4.09894800	0.26767100	-1.99315700
H	4.22437100	-0.35435100	-2.88092500
H	3.39564600	1.07497200	-2.21174000
H	5.06413000	0.69973600	-1.71164000
C	3.24283800	-0.10255400	0.33353200
H	2.94769600	1.56576800	1.95463100
H	3.84570900	2.16151400	0.18607000

N	2.83100700	-1.21500000	1.02270400
N	3.58563400	-0.56579100	-0.91291900
B	3.21274900	1.35551000	0.80641200

1ag-Int-1

C	2.19424800	4.51224700	-0.24515900
H	3.18173000	4.54884800	0.25230000
H	2.39354900	4.36028700	-1.31577400
H	1.74121900	5.50417200	-0.11815300
C	1.32341100	3.43444500	0.31213800
H	0.79477200	3.62281700	1.24463700
C	0.39910200	1.12163300	0.31807000
O	-0.28526400	1.24135000	1.33184700
N	0.20418200	0.03197800	-0.54208800
H	0.71849600	-0.03058700	-1.41813100
S	-0.77738200	-1.31178400	-0.22717100
O	-0.43749500	-1.88624500	1.08003700
O	-0.62039400	-2.13068600	-1.44360100
C	-2.44060800	-0.66631700	-0.16307700
C	-3.06482700	-0.29634300	-1.35985200
C	-3.08726000	-0.54330700	1.06614100
C	-4.36266800	0.20620000	-1.31118000
H	-2.54804700	-0.39856800	-2.30951600
C	-4.39117800	-0.04350900	1.09202600
H	-2.58149400	-0.82625100	1.98270600
C	-5.04686300	0.33780300	-0.08842600
H	-4.85628900	0.49743800	-2.23541100
H	-4.90277700	0.05450700	2.04625900
C	-6.45969000	0.86454000	-0.06208200
H	-7.15089400	0.13821700	-0.51015900
H	-6.79540300	1.06372100	0.96073200
H	-6.54512900	1.79111500	-0.64261900
C	1.52041400	2.02170000	-0.09106300
H	1.69021100	1.94471900	-1.17376200
H	3.87623800	2.04957300	0.21555100
H	2.84959600	1.51426000	1.85414500
C	3.14262800	-0.14898300	0.26673900
C	3.09810400	-2.40000500	0.34230400
C	3.47275700	-2.04039700	-0.91586800
H	2.95705200	-3.37065700	0.79318700
H	3.72399000	-2.63237300	-1.78323000
C	2.45777500	-1.17591200	2.44601600
H	3.21699600	-0.68632700	3.05971200
H	1.51537900	-0.63158900	2.51314300
H	2.30946200	-2.19872700	2.79446400
C	3.77042500	0.13100700	-2.14204600
H	2.84173000	0.29989600	-2.69594300
H	4.20329800	1.08981100	-1.85618700
H	4.47502800	-0.41722700	-2.77053400
N	3.49851500	-0.65682900	-0.94174300
N	2.89929100	-1.22894100	1.05110300
B	2.94121600	1.39877000	0.64841600

RSH

C	2.73065200	-0.32048300	-0.17387800
C	3.06827800	0.73765700	-1.24657200
H	2.58312800	1.69409300	-1.04253400
H	4.15181500	0.90855300	-1.27663300
H	2.75475900	0.40213500	-2.24313300
C	3.17333300	0.20610500	1.20199500
H	2.68479100	1.15338400	1.44808500
H	2.95716400	-0.51440600	1.99873300
H	4.25609500	0.38407000	1.19811500
C	3.56292100	-1.58479300	-0.49393400
H	3.42391600	-2.35649900	0.27305900
H	3.27440500	-2.01462100	-1.46141700
H	4.63161500	-1.34284700	-0.53960400

C	1.23651100	-0.77602700	-0.25061000
H	1.03532300	-0.93494400	-1.31707000
H	1.18189100	-1.77166300	0.21201900
C	0.00029000	-0.02128400	0.33996100
C	-1.23853200	-0.79078200	-0.22828300
H	-1.19163500	-1.76910400	0.27050500
H	-1.03633300	-0.99028400	-1.28753300
C	-2.72959700	-0.32217700	-0.17273200
C	-3.57121700	-1.59107300	-0.44642300
H	-3.43904600	-2.33454200	0.34913200
H	-4.63798300	-1.34277400	-0.50257500
H	-3.28508600	-2.05913600	-1.39672100
C	-3.17174300	0.26052500	1.18056900
H	-2.97769700	-0.43722400	2.00287500
H	-2.66537100	1.20529400	1.39878500
H	-4.25061800	0.45996900	1.16140900
C	-3.05527600	0.69582300	-1.28727000
H	-2.74228600	0.31818600	-2.26879300
H	-4.13713800	0.87499500	-1.32722200
H	-2.56260400	1.65550800	-1.12005000
C	0.00810400	-0.05586200	1.88121900
H	0.04206300	-1.09918200	2.21477900
H	0.87217800	0.45809600	2.30103700
H	-0.88484100	0.40185700	2.30554400
S	-0.00773500	1.79899700	-0.10796400
H	0.00291100	1.64136600	-1.44763400

1ag-TS-2

C	0.84992100	3.19469900	1.54644400
H	1.90521500	3.10866100	1.82933600
H	0.78400700	3.79554000	0.63175100
H	0.33897300	3.75491200	2.34591700
C	0.22481500	1.84207500	1.36432100
H	0.36327400	1.13905100	2.18688700
C	-1.54451800	0.31410600	0.53003300
O	-1.42604500	-0.54705600	1.39790600
N	-2.22700000	0.03467600	-0.65743500
H	-2.25567900	0.71603100	-1.41290900
S	-2.98592200	-1.43681500	-1.02022800
O	-3.44039300	-1.24188500	-2.40937100
O	-3.96708600	-1.76104800	0.02174100
C	-1.66835100	-2.63992300	-0.98156700
C	-1.58490400	-3.54038300	0.08111800
C	-0.72508400	-2.63460300	-2.01324200
C	-0.52899100	-4.45126300	0.10605300
H	-2.32210800	-3.51974300	0.87609500
C	0.32702400	-3.54703800	-1.96424500
H	-0.80264600	-1.92767800	-2.83390200
C	0.44675000	-4.46085500	-0.90337400
H	-0.45226000	-5.15278200	0.93311700
H	1.07174100	-3.54320600	-2.75643400
C	1.63031400	-5.38958700	-0.81915000
H	1.37881500	-6.31523500	-0.29013600
H	2.01329400	-5.64515400	-1.81312300
H	2.44707600	-4.90539000	-0.26551300
C	-1.08267300	1.73345600	0.65353200
H	-1.01203200	2.19659900	-0.33960500
H	-2.36726200	2.16972100	2.63946300
H	-2.04671600	3.75455300	1.45203000
C	-3.71465400	2.29609900	0.75360900
C	-5.32345800	2.23090500	-0.82312600
C	-5.69488400	1.39037500	0.18087900
H	-5.80733400	2.49083400	-1.75274700
H	-6.56782400	0.76687700	0.30102800
C	-4.69383600	0.64452600	2.36457800
H	-3.81256700	0.00230000	2.38574200
H	-4.69630600	1.30142000	3.23704300

H	-5.59307000	0.02719700	2.36881800
C	-3.32378400	3.66812800	-1.30698800
H	-2.69838400	4.30974600	-0.68627200
H	-2.69087300	3.07786500	-1.97729500
H	-4.00791000	4.28088000	-1.89757400
N	-4.69646500	1.44201700	1.13622600
N	-4.10830400	2.77936400	-0.45215700
B	-2.31298400	2.56721500	1.49301600
C	3.04643300	-1.40419100	2.08012200
C	1.81799400	-2.04294700	1.41140400
H	2.00010300	-2.28877400	0.36267700
H	1.56009500	-2.97531300	1.93282200
H	0.94558600	-1.38407900	1.44602900
C	4.22736300	-2.39849400	2.02722100
H	4.47873600	-2.69979500	1.00680900
H	5.12871700	-1.96820000	2.48433600
H	3.97456900	-3.31122900	2.58372700
C	2.70810800	-1.17062600	3.57162700
H	3.54755200	-0.70153900	4.10303500
H	1.83274700	-0.51675900	3.67993900
H	2.48228000	-2.12105800	4.07295800
C	3.43822000	-0.00058400	1.51866000
H	2.74471000	0.73084000	1.94900900
H	4.41675000	0.23107800	1.96540100
C	3.57003800	0.36192000	0.00332600
C	4.35586000	1.71664200	-0.00248200
H	5.37891800	1.43286000	0.28660900
H	3.97110900	2.31807700	0.83181600
C	4.48181000	2.70018000	-1.21114200
C	5.71696100	3.58150700	-0.91226700
H	6.63925700	2.98543900	-0.90268100
H	5.82938600	4.36471700	-1.67388400
H	5.62432300	4.07485200	0.06487600
C	4.70513600	2.01431200	-2.57008600
H	5.58629200	1.36122900	-2.55224900
H	3.83768500	1.41724300	-2.86991500
H	4.87112100	2.77374300	-3.34652400
C	3.26166900	3.63967700	-1.31226400
H	3.11501100	4.19069300	-0.37451500
H	3.41687400	4.37658900	-2.11256900
H	2.34352200	3.08810300	-1.52661900
C	4.33029400	-0.71955000	-0.78500200
H	5.33089300	-0.85444000	-0.35450500
H	3.80993400	-1.67895100	-0.74694600
H	4.45162200	-0.45210000	-1.83604100
S	1.87954500	0.48470100	-0.80560300
H	1.14885500	1.15764400	0.31631500

3ag-α

C	-4.35825900	-0.68714800	0.75961400
H	-4.87831200	-0.83482200	-0.19643300
C	-2.92097800	-0.19576500	0.49500500
H	-2.50860600	0.21441700	1.42855500
C	-2.03920400	-1.31749600	0.04325700
O	-2.41702800	-2.34079400	-0.52275900
C	0.32332700	3.00468800	-0.03454200
C	0.65016900	2.42244500	-1.22004400
H	0.87381400	3.67582800	0.60758600
H	1.54660300	2.47973900	-1.81743100
C	-1.43436000	1.74917800	-0.68753300
N	-0.95896100	2.58305000	0.27350100
N	-0.43637900	1.65446500	-1.60209700
C	-1.66157400	2.94826800	1.50174900
H	-1.37385400	3.96350500	1.78268400
H	-2.73682900	2.90774900	1.32869400
H	-1.39518500	2.25718800	2.30682600
C	-0.47331900	0.82888700	-2.81025600

H	-1.30561200	1.13438900	-3.44723800
H	0.46624400	0.97053000	-3.34625000
H	-0.58156700	-0.22267000	-2.53893300
B	-2.85811200	0.99650100	-0.68620700
H	-3.05526400	0.50229200	-1.78286200
H	-3.72204800	1.82672300	-0.44644500
C	-5.16245800	0.27491800	1.64113500
H	-5.25603300	1.26548100	1.18158400
H	-6.17566100	-0.10978100	1.81593800
H	-4.68157900	0.40465500	2.62042300
N	-0.66830600	-1.08536500	0.24664100
H	-0.37176900	-0.32412400	0.85361400
S	0.56813300	-2.16673200	-0.19882000
O	0.56893600	-3.32790200	0.70911700
O	0.48666000	-2.40657700	-1.64824500
C	1.98723600	-1.14023100	0.15278200
C	2.48313700	-1.07879500	1.45921800
C	2.56158800	-0.40176400	-0.88392500
C	3.57722900	-0.25606500	1.72161000
H	2.02632700	-1.66168400	2.25332800
C	3.66175600	0.40772500	-0.60082100
H	2.15481200	-0.45374400	-1.88836100
C	4.18518200	0.49411700	0.69911300
H	3.96895900	-0.19881200	2.73427000
H	4.11491500	0.98644400	-1.40174200
C	5.39102900	1.35018100	0.99273000
H	6.30443500	0.73965000	0.98253000
H	5.51300900	2.14116200	0.24524700
H	5.31931500	1.81183600	1.98391400
H	-4.33170800	-1.67246400	1.24687800

RS•

C	2.72836900	-0.27834700	-0.18674500
C	3.02574800	1.20313500	0.10030500
H	2.50210800	1.56011800	0.99431100
H	4.09946800	1.34627000	0.27603300
H	2.74117900	1.83857900	-0.74613300
C	3.20110000	-1.13884400	1.00513400
H	2.75013600	-0.83433900	1.95154800
H	2.96503000	-2.19748200	0.84211300
H	4.28875000	-1.05283200	1.11869800
C	3.55586700	-0.70720700	-1.42163600
H	3.36648200	-1.75685200	-1.67858800
H	3.30842300	-0.09482600	-2.29702000
H	4.62891800	-0.59769700	-1.22444700
C	1.23886900	-0.54655100	-0.56360600
H	1.09629700	-0.21499300	-1.59807400
H	1.11015400	-1.63782600	-0.57025600
C	0.02211400	0.01913000	0.24881000
C	-1.22442600	-0.73214500	-0.36154100
H	-1.11467000	-1.75358300	0.02681800
H	-1.06437500	-0.79510000	-1.44403400
C	-2.71376500	-0.31780400	-0.16519200
C	-3.54784700	-1.52676800	-0.65296800
H	-3.36383900	-2.41112800	-0.03136300
H	-4.61898700	-1.29798600	-0.60654100
H	-3.30323600	-1.78479400	-1.69065100
C	-3.09845400	-0.02581700	1.29537600
H	-2.84009100	-0.86256100	1.95387100
H	-2.60429200	0.87586900	1.67127500
H	-4.18070000	0.13707200	1.36868000
C	-3.09474300	0.89326000	-1.03789600
H	-2.82344300	0.73006400	-2.08732900
H	-4.17557500	1.07371500	-0.98816000
H	-2.60039300	1.81315600	-0.70236200
C	0.15247400	-0.25029900	1.75694400
H	0.30005600	-1.32312700	1.92946400

H	1.00453000	0.28279100	2.18145300
H	-0.73598800	0.06423200	2.30204700
S	-0.13798600	1.83392500	-0.06582800

1ag-TS-1'

C	2.41344600	3.78101100	-0.71415600
H	3.14309000	4.27179100	-0.06306200
H	2.91812400	3.48458900	-1.64099200
H	1.65330500	4.53456100	-0.97710900
C	1.75124300	2.61024200	-0.03842200
H	1.43646300	2.75081000	0.99305100
C	1.15700400	1.59007700	-0.74604400
H	1.38503300	1.45180200	-1.80179300
C	0.33103000	0.60329400	-0.08878100
O	-0.05400300	0.64031500	1.09030200
N	-0.01782400	-0.50025300	-0.90934400
H	0.14647900	-0.42885800	-1.91183500
S	-1.23858000	-1.60853700	-0.53876700
O	-0.87555900	-2.34739000	0.67626400
O	-1.42038700	-2.34433600	-1.80540800
C	-2.70657600	-0.64342800	-0.21011900
C	-3.40025700	-0.08506200	-1.29016700
C	-3.12842500	-0.44885300	1.10510500
C	-4.53726400	0.67816400	-1.03570800
H	-3.06264800	-0.24821300	-2.30937100
C	-4.27456600	0.31457800	1.33822600
H	-2.57172700	-0.88310900	1.92784000
C	-4.99238000	0.89025600	0.27882100
H	-5.08486200	1.11240600	-1.86897300
H	-4.61241500	0.46565800	2.36052300
C	-6.21825300	1.73161600	0.53159500
H	-7.05285900	1.41345200	-0.10494000
H	-6.53785600	1.67231900	1.57696300
H	-6.01818400	2.78585200	0.29782500
C	3.47202000	-1.72524200	-0.65542900
H	3.59970900	-2.33506600	-1.53725400
C	2.91237500	-2.00928500	0.55624400
H	2.45339700	-2.91179300	0.92986900
C	2.46944000	-0.72771800	2.67314100
H	1.59823900	-0.06714800	2.65414900
H	2.17183000	-1.71547600	3.02943400
H	3.23628100	-0.31751500	3.33436100
C	4.53075500	0.30265100	-1.70684800
H	4.57960100	-0.36201300	-2.57066800
H	3.94691100	1.19043200	-1.96055500
H	5.54230400	0.60488900	-1.42071900
C	3.61868600	0.13415600	0.61813300
H	3.55361300	1.92028700	2.15051100
H	4.54954900	2.27065700	0.36316200
N	3.00138000	-0.86643800	1.32200000
N	3.89041300	-0.41250900	-0.60874700
B	3.82720900	1.59264500	1.03193900

1ag-Int-1'

C	-4.94084900	-1.39958600	0.12086600
H	-5.05187900	-2.31934400	-0.47116700
H	-5.07909000	-1.66540700	1.17774200
H	-5.75350400	-0.71818400	-0.15793800
C	-2.45278300	-1.57977000	0.29320600
H	-2.55905500	-2.20345200	1.17998900
C	-1.16094200	-1.51841400	-0.33251300
O	-0.88041500	-0.86727300	-1.35145800
N	-0.16690000	-2.29455100	0.31448900
H	-0.33680400	-2.65827500	1.24993200
S	1.43019100	-2.46036600	-0.19181800
O	1.44832800	-2.93505200	-1.58128100
O	2.03318800	-3.28887500	0.86949800

C	2.17101500	-0.83414500	-0.13451400
C	2.63936300	-0.34870500	1.09133300
C	2.30029100	-0.09112300	-1.30892600
C	3.25730800	0.89960000	1.12763600
H	2.52979400	-0.93588200	1.99777600
C	2.92434000	1.15595600	-1.25040100
H	1.91839300	-0.47882300	-2.24646000
C	3.41622400	1.66716800	-0.03955300
H	3.62184200	1.28495900	2.07688300
H	3.02501900	1.74140700	-2.16092900
C	4.10438500	3.00767000	0.01671000
H	5.19496100	2.87992400	0.05296600
H	3.86940700	3.61649700	-0.86258900
H	3.81368100	3.56486600	0.91481900
C	-3.57407700	-0.73519300	-0.10727200
H	-3.46309900	-0.42808800	-1.15501300
H	-4.46651200	1.35091000	0.54453500
H	-3.48247600	0.40641100	2.01541300
C	-2.16175200	1.52945000	0.47265200
C	-0.68593000	2.84209000	-0.61253600
C	-0.08138500	2.39350500	0.52084200
H	-0.32933200	3.48323600	-1.40456700
H	0.90769500	2.56195300	0.91544500
B	-3.47934300	0.68755500	0.82961300
N	-0.99855900	1.58628800	1.16964300
C	-0.73274000	0.90018600	2.43406200
H	-1.35927100	1.31465100	3.22751700
H	-0.93619800	-0.16677000	2.32884900
H	0.31923100	1.04477100	2.68306800
C	-2.91363600	2.48081100	-1.71599700
H	-2.87317700	1.61959700	-2.38869200
H	-3.92205800	2.57889300	-1.31213900
H	-2.64991500	3.38624300	-2.26581500
N	-1.96108300	2.30548900	-0.62216600

1ag-TS-2’

C	1.17828000	3.52722900	-2.32327000
H	2.18329700	3.44487900	-1.88947600
H	1.23228500	3.16824400	-3.36055500
H	0.91227300	4.59040400	-2.35449900
C	0.42675700	1.27189800	-1.50927800
H	0.50195200	0.81229700	-2.49916700
C	-0.24787400	0.42339800	-0.49769000
O	-0.72372500	0.83652900	0.55821800
N	-0.27796000	-0.93390300	-0.82329900
H	0.10675700	-1.25281100	-1.70986300
S	-0.68446000	-2.19964900	0.23276700
O	0.04699300	-2.04039000	1.49392400
O	-0.46275600	-3.39647300	-0.59760100
C	-2.43062600	-2.03548200	0.55944700
C	-3.34179700	-2.55300200	-0.36622300
C	-2.85398700	-1.45334700	1.75612600
C	-4.70318000	-2.49119500	-0.07286900
H	-2.99621900	-3.00747700	-1.28938800
C	-4.22035800	-1.40364000	2.02981400
H	-2.13031200	-1.05359400	2.45761900
C	-5.16287000	-1.92037400	1.12617100
H	-5.41890100	-2.89531500	-0.78451600
H	-4.55926400	-0.95419500	2.95996200
C	-6.63911600	-1.81919900	1.41302300
H	-7.19845900	-2.62578200	0.92691000
H	-6.84118200	-1.84985400	2.48918900
H	-7.03488200	-0.86736400	1.03167800
C	0.13938900	2.73516100	-1.51719600
H	0.11641500	3.10212600	-0.48374400
H	-1.53479800	4.20488000	-2.26489600
H	-1.41999900	2.50460300	-3.31877200

C	-2.57726100	2.39062800	-1.31361200
C	-4.06618600	2.09136000	0.35088000
C	-4.26145700	1.09861000	-0.55849000
H	-4.55828600	2.30625200	1.28753500
H	-4.95682100	0.27519500	-0.57521300
B	-1.38310800	2.99033000	-2.20055700
N	-3.33638800	1.29461300	-1.56851300
C	-3.20385200	0.42416000	-2.73722200
H	-3.45636900	0.97274100	-3.64751000
H	-2.18101300	0.05033600	-2.80836400
H	-3.88869000	-0.41619800	-2.61463200
C	-2.44508800	3.98905400	0.60466100
H	-1.60541700	3.62992800	1.20607300
H	-2.09331100	4.74638600	-0.09680600
H	-3.20710400	4.41976900	1.25727900
N	-3.03493900	2.87563700	-0.13234500
C	3.92047700	-2.44495300	-1.00502100
C	2.89524600	-2.36927600	-2.15647400
H	2.77993300	-1.34914700	-2.53107000
H	3.21499300	-3.00795700	-2.99156900
H	1.91463700	-2.72986300	-1.82294400
C	5.32038400	-2.11179000	-1.54947000
H	5.36174800	-1.10433600	-1.97658100
H	6.08575500	-2.18886700	-0.76759200
H	5.58735300	-2.82105400	-2.34489200
C	3.93904600	-3.90853900	-0.50588600
H	4.71218300	-4.05853200	0.25950800
H	2.97178800	-4.18908900	-0.06778800
H	4.14540100	-4.59934800	-1.33445300
C	3.47480400	-1.58171300	0.21992800
H	2.39861400	-1.75402300	0.33604200
H	3.93906900	-2.02876600	1.11177600
C	3.71864100	-0.04571800	0.39861800
C	2.75909700	0.36708000	1.56080300
H	2.85441800	-0.43046900	2.31236400
H	1.74178600	0.26736400	1.17415600
C	2.82233200	1.70445500	2.36363800
C	1.50684700	1.75046700	3.17849500
H	1.41301400	0.86969500	3.82878900
H	1.47825900	2.64331100	3.81732000
H	0.63336700	1.77280300	2.51629000
C	3.98889700	1.73176300	3.37525800
H	3.99910400	0.82208100	3.99055700
H	4.96594100	1.82378600	2.89375200
H	3.87737900	2.59001600	4.05180400
C	2.88061900	2.96754700	1.48744800
H	2.05668400	2.99112400	0.76658300
H	2.80354300	3.86195500	2.12199500
H	3.81484100	3.03212800	0.92248900
C	5.19287700	0.24392200	0.74086800
H	5.45976300	-0.28323800	1.66609000
H	5.87294800	-0.09195100	-0.04346600
H	5.36456200	1.31154100	0.89354600
S	3.39051600	0.94881100	-1.15807300
H	1.80027500	1.08436700	-1.17594500

3ag-β

C	-3.61589000	0.24119900	1.30142500
H	-4.09969800	0.67899200	0.41514500
C	-2.37316300	1.11654800	1.60634600
H	-1.72439900	0.61686600	2.33622700
C	-1.59827900	1.50933300	0.36353000
O	-2.08279000	2.17795800	-0.54451300
C	-0.25388400	-2.16525100	-1.19910600
C	-1.12633200	-1.68304900	-2.12404400
H	0.75323500	-2.53894100	-1.29666600
H	-1.03021100	-1.54313000	-3.18989100

C	-2.14395400	-1.58662200	-0.11150500
N	-0.89188100	-2.09294300	0.02763900
N	-2.27713000	-1.33505100	-1.43989100
C	-0.26563100	-2.47634100	1.29223800
H	0.77520700	-2.73530100	1.09125100
H	-0.30206800	-1.64066000	1.99400900
H	-0.77929200	-3.33682800	1.72706500
C	-3.44313000	-0.71181600	-2.06118800
H	-3.37090700	0.37592800	-1.97593300
H	-3.47322700	-0.99836200	-3.11403900
H	-4.34897000	-1.05878200	-1.56185400
B	-3.24848700	-1.34170000	1.03800000
H	-4.26779600	-1.94056300	0.68884400
H	-2.82876700	-1.84487400	2.07409000
C	-4.60930200	0.36167900	2.46972600
H	-4.92859000	1.40157400	2.64034200
H	-5.51054600	-0.23767500	2.28340900
H	-4.16350000	-0.00343600	3.40649200
H	-2.67956000	2.07647900	2.04976200
N	-0.28141700	1.07028200	0.31452900
H	0.10404800	0.56901900	1.11262300
S	0.89138000	1.69561200	-0.77463600
O	0.54922700	1.30013700	-2.14640300
O	1.08277900	3.12585400	-0.47759100
C	2.31085400	0.77028600	-0.21376400
C	2.74492300	-0.32824400	-0.95712700
C	2.95096400	1.15596000	0.97006300
C	3.84297300	-1.05714700	-0.49694200
H	2.23549400	-0.60806300	-1.87251200
C	4.04077000	0.41092600	1.41356000
H	2.60525600	2.01761500	1.53366200
C	4.50340100	-0.70376200	0.69007100
H	4.18831800	-1.91417600	-1.06961000
H	4.54374600	0.69883400	2.33357300
C	5.70023100	-1.48064400	1.17626200
H	6.62393000	-0.91473700	0.99445200
H	5.64056000	-1.66361700	2.25553500
H	5.79005600	-2.44339900	0.66304000
